# Decoding the lipid etiology of atherogenic index of plasma and gout: establishing the causal role of triglycerides through NHANES, Mendelian randomization, and network pharmacology

**DOI:** 10.1186/s40842-026-00309-0

**Published:** 2026-07-13

**Authors:** Yajing Tian, Xingzheng Zhang, Shaofang Gao, Hefei Wang

**Affiliations:** 1 Hebei University of Chinese Medicine, Shijiazhuang, Hebei China; 2 Hebei Provincial Hospital of Traditional Chinese Medicine, Shijiazhuang, Hebei China; 3 Yanzhao Traditional Chinese Medicine Culture Comprehensive Research Laboratory, Shijiazhuang, Hebei China

**Keywords:** Atherogenic index of plasma (AIP), Gout, Triglycerides, Mendelian randomization, Network pharmacology

## Abstract

**Background:**

Although a cross-sectional relation between the atherogenic index of plasma (AIP) and gout has been well documented, the causal roles of its principal components, TG and HDL-C, have not yet been clearly established. Therefore, this study sought to explicate the causal relations between AIP, its individual constituents, and the risk of gout, as well as to investigate the potential molecular mechanisms underlying these associations.

**Methods:**

We adopted an integrated analytical pipeline. First, mediation and cross-sectional analyses were carried out to quantify the phenotypic associations of TG, HDL-C, and AIP with gout. Second, both multivariable and univariable Mendelian randomization (MR) analyses were applied to assess causal directions. Finally, a network pharmacology approach was applied to construct interaction networks linking lipid-related factors with gout, and subsequent enrichment analyses were done to identify the main biological pathways involved and key genes were identified using the Icelandic database’s pQTLs.

**Results:**

AIP showed a significant positive link with gout prevalence (OR = 1.700, *p* = 0.010), with this relationship being mediated mainly by HDL-C (45.19%), rather than TG or LDL-C. Univariable MR analyses indicated that TG exerted a substantial causal effect on gout risk (OR = 1.0058, *p* < 0.001). Although univariable MR showed a nominally protective association for HDLC (OR = 0.623, 95% CI: 0.399–0.974, *p* = 0.042), this effect was attenuated and became statistically nonsignificant after multivariable adjustment for other lipid traits (*p* > 0.05). After adjustment for genetic correlations in multivariable MR analyses, TG remained the sole lipid trait with a robust and independent causal effect on gout (OR = 1.0077, *p* < 0.001), whereas neither HDL-C nor LDL-C reached statistical significance. The TG-specific gout-associated gene set (Group B) was significantly enriched in T-cell receptor and NF-κB signaling pathways, with hub genes including PTPRC, MYD88, and LCK, supporting a direct lipid–immune axis. By contrast, the combined TG/HDL-C gene set (Group F) showed predominant enrichment in PI3K-Akt and cytokine-cytokine receptor pathways, whereas genes uniquely shared between HDL-C and gout (Group C) were mainly enriched in fundamental cellular processes without marked inflammatory pathway involvement.

**Conclusions:**

AIP exhibits a non-linear positive association with gout, but this association is primarily mediated by HDL‑C, which itself has no independent causal protective effect. TG may play an independent causal role in gout, potentially involving T‑cell receptor/NF‑κB signaling pathways. However, these findings are exploratory and require further validation.

**Supplementary information:**

The online version contains supplementary material available at 10.1186/s40842-026-00309-0.

## Introduction

Gout is a highly painful inflammatory arthritis marked by the monosodium urate crystals’ deposition in joints and surrounding tissues, with chronic hyperuricemia serving as its fundamental pathophysiological basis [1]. The initial clinical presentation most commonly involves the first metatarsophalangeal joint [2]. In recent decades, global shifts in dietary habits and lifestyle patterns have contributed to a steady raise in the prevalence of gout. In 2020, around 55.8 million people globally were impacted by gout, suggesting a 22.5% rise compared with 1990. During the same year, the global occurrence of gout in men was 3.26-fold higher than in women, and prevalence raised progressively with advancing age. Projections indicate that the total number of patients with gout can reach 95.8 million by 2050 [3]. Gout has thus emerged as a major public health concern, causing substantial physical pain and psychological distress to affected individuals, while simultaneously imposing a considerable socioeconomic burden. Historically, etiological investigations and clinical management strategies have primarily focused on abnormalities in purine metabolism and impaired renal excretion of uric acid [4]. However, accumulating evidence suggests that gout, particularly when coexisting with metabolic syndrome components such as insulin resistance and obesity, and can arise from a more complex network of metabolic disturbances. Accordingly, a deeper comprehension of these interconnected metabolic pathways is essential for the effective preventive strategies’ development and targeted interventions for gout [5].

Among the spectrum of metabolic abnormalities, the link between disordered lipid metabolism and gout has attracted growing interest. Epidemiological evidence consistently demonstrates that hypertriglyceridemia frequently coexists with gout [6]. Nevertheless, whether this relationship reflects simple comorbidity or represents a causal contribution to gout development remains unresolved. The atherogenic index of plasma (AIP), measured as log_10_(TG/HDLC), is a comprehensive indicator that integrates lipid homeostasis, insulin resistance, and cardiovascular risk. Compared with individual lipid parameters, AIP more effectively captures the characteristics of an atherogenic lipid profile. Given that AIP fundamentally represents the balance between TG and HDL-C, we hypothesize that AIP, and particularly its core component, TG, may exert an upstream causal influence on gout pathogenesis, rather than merely constituting associated metabolic abnormalities. Many composite indicators have been proposed for metabolic disorders, and they have shown significant associations with adverse outcomes in various diseases, including the AIP [7]. Importantly, AIP, calculated as log_10_(TG/HDLC), has emerged as a particularly strong predictor among epidemiologically relevant measures. Beyond its close association with metabolic abnormalities, it involved in a broad range of cardiometabolic disorders, such as hypertension, cardiovascular disease, and metabolic syndrome [8]. However, the clinical as well as statistical connections between AIP and gout, along with the potential core genes, mediating factors, or key mechanisms linking dyslipidemia to gout risk, remain to be fully validated. To address this, we seek to investigate the potential association between AIP and gout utilizing data from the NHANES database. Mendelian Randomization (MR) analysis will be applied to assess causal relationships, while network pharmacology will be used to identify the core genes and key mechanisms driving gout pathogenesis. The results of this study are anticipated to offer novel insights into strategies for gout intervention, thereby supporting more effective management and prevention of gout-related conditions [9].

## Methods

### Study design and data source

We employed data from NHANES, a nationwide survey intended at assessing the nutritional and health status of both US adults and children. The survey includes health examinations and laboratory testing for participants across all age groups and collects extensive information on the health of U.S. residents. In addition, NHANES gathers details on participants’ dietary intake, including foods, beverages, and supplements, to assess nutrient consumption. The combination of dietary interviews and blood analyses allows for a detailed evaluation of the nutritional status of American adults and children. All NHANES procedures are approved by the Research Ethics Review Board of the National Center for Health Statistics, and written informed consent is taken from every participant. Detailed information about the NHANES study and its datasets is available on the official website https://www.cdc.gov/nchs/nhanes [10].

#### Study population

**Fig. 1:**
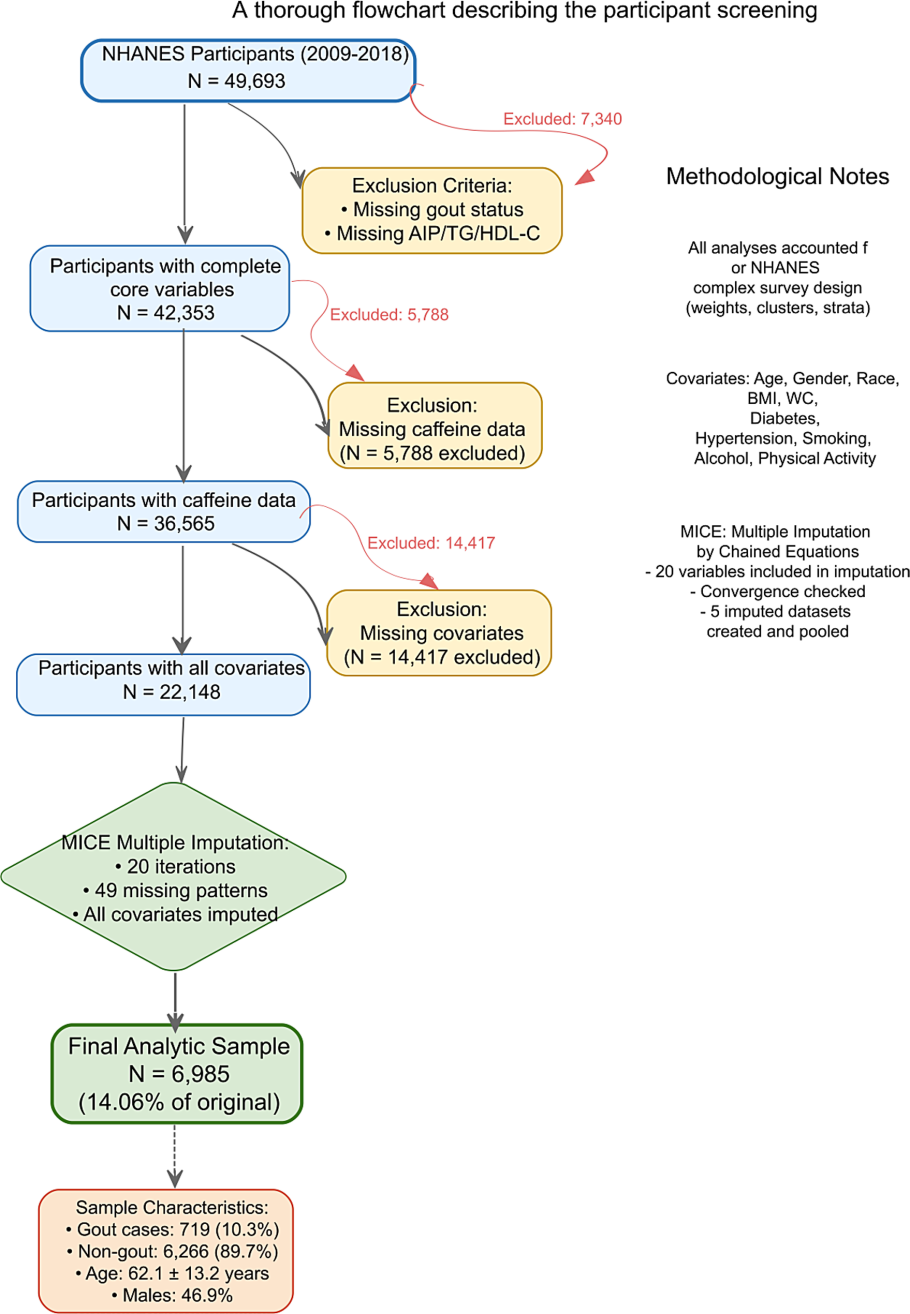
A thorough flowchart describing the participant screening

This research analyzed participants from 2009 to 2018, encompassing a 10-year dataset with a total of 49,693 individuals (File S1: Table S1). The analysis focused on participants (above 20 years) who had entire data on TG, AIP, HDL-C, and other main covariates. Information on gout was obtained from the “Medical Conditions” section of the National Health Interview Survey. Participants with missing data on gout status or key lipid exposures (triglycerides, HDL-C, LDL-C) were excluded from the analysis. For participants with complete outcome and exposure data, missing values in covariates (including physical activity, demographic characteristics (age, sex, race/ethnicity), body mass index (BMI), waist circumference (WC), health conditions [diabetes, hypertension], alcohol consumption, and smoking status) were handled using multiple imputation by chained equations (MICE). The final analytical cohort consisted of 6,985 participants (File S1: Table S2). A thorough flowchart describing the participant screening and selection process is given in Fig. 1.

#### Assessment of AIP

The AIP was determined as previously described utilizing the formula: AIP = log₁₀[TG/HDL-C] [11].

#### Assessment of gout

The NHANES MCQ questionnaire section is largely founded on the “Medical Conditions” portion of the U.S. NHI Survey. During home interviews, all contributors were asked, “Has any health professional informed you that you have gout?”

#### Covariates

Covariates included dietary caffeine intake, PA, demographic factors (age, sex, race/ethnicity), BMI, WC, behavioral factors (alcohol consumption and smoking), and health conditions (diabetes and hypertension). Moreover, race/ethnicity was categorized as non-Hispanic White (NHW), non-Hispanic Black (NHB), Mexican American, other Hispanic, and other. Laboratory measurements included HDL-C, TG, and LDL-C.

As described earlier, dietary intake data were utilized to guesstimate the quantities and types of foods and beverages, such as all water’s forms, consumed during the 24 hours prior the interview and to calculate the corresponding nutrients, intake of energy, and other dietary components. Caffeine intake was estimated using the individual foods file, which provides detailed information on the type and quantity of each food item, along with its nutrient composition. Variable names for both Day 1 and Day 2 assessments are listed accordingly.

Participants who replied “yes” to the question, “Have any health professional told you that you have hypertension?” were classified as having hypertension. PA was assessed utilizing the global PA questionnaire, with vigorous recreational activities defined as: “The following questions exclude work- and transportation-related activities already addressed. I would now like to ask about sports, fitness, and recreational activities. Do you/does the participant engage in any vigorous-intensity fitness, sports, or recreational activities, such as basketball or running, that substantially increase heart rate or breathing for at least 10 consecutive minutes?” Alcohol consumption was categorized into four groups: former drinkers, current drinkers, never drinkers, and missing. Smoking status was categorized into 3 groups grounded on self-reported questionnaire data: former smokers, current smokers, and never smokers.

### Statistical analysis of NHANES data

For analyses involving imputed data, each of the five imputed datasets generated by MICE (as described in Sect. “Study population”) was analyzed separately, and results were pooled using Rubin’s rules. All statistical analyses accounted for the NHANES sampling weights and the complicated multistage cluster survey design, utilizing the survey package for adjusted analyses. To address the complicated survey design, including oversampling, nonresponse, and post-stratification adjustments, each weight represents the theoretical population size that participants correspond to, ensuring representativeness of the US non-institutionalized civilian population [12]. Baseline participant characteristics were presented according to AIP values calculated from HDL-C and TG, while categorical variables were summarized as percentages and frequencies. Variances in AIP traits were compared utilizing Rao-Scott chi-square or Kruskal-Wallis tests. Gout was treated as a binary outcome (yes/no). MLR models were utilized to measure the links among AIP, covariates, and gout.

Three models were established for analysis: Model 1 included no covariates and Model 2 was adjusted for age, sex, and ethnicity. Besides the covariates in Model 2, Model 3 further accounted for BMI, smoking status, alcohol consumption, diabetes, hypertension, and PA. To scrutinize the dose–response relationship between AIP, TG, HDLC and gout, restricted cubic spline (RCS) functions were fitted based on multivariable logistic regression models. Knots were placed at the percentiles recommended by Harrell (2015): for 3 knots at the 10th, 50th, and 90th percentiles; for 4 knots at the 5th, 35th, 65th, and 95th percentiles; for 5 knots at the 5th, 27.5th, 50th, 72.5th, and 95th percentiles; and for 6 knots at the 5th, 23.3rd, 41.7th, 58.3rd, 75th, and 95th percentiles of the respective exposure distribution. The reference value for odds ratio (OR) calculation was set at the median of each exposure. Models with 3, 4, 5, and 6 knots were fitted, and the final number of knots was selected based on the lowest Akaike information criterion (AIC). For all three exposures, the 3‑knot model yielded the lowest AIC (AIP: 4343.1; TG: 4373.8; HDL‑C: 4326.8); therefore, the 3‑knot specification was used for inference. The actual knot values and AIC comparisons are provided in Supplementary Tables S21 and S22. When a nonlinear link between AIP and gout was detected, the segmented package was applied to identify inflection points, guided by the bootstrap resampling method and likelihood ratio test [13, 14]. Segmented MLR models were then employed to evaluate the link between AIP and gout on either side of the breakpoint. To examine the stability and robustness of this association, subgroup analyses and interaction tests were performed considering variables such as BMI, age, diabetes, alcohol consumption, hypertension, gender, and smoking. If significant interactions were observed, stratified RCS analyses were done to explore potential disparities in the AIP-gout link across subgroups defined by the identified effect modifiers. In addition, after adjustment for covariates included in Model 3, the R package mediation was used to explore the mediating effects of HDL-C and TG on the relationship between AIP and gout [15]. The mediation analysis decomposed the total effect of AIP on gout into an indirect effect (via each mediator) and a direct effect (ADE). The proportion mediated was calculated as the indirect effect divided by the total effect. Statistical significance of the indirect effect was assessed using bootstrap resampling with 1,000 iterations [16]. All mediators (HDL‑C, TG, BMI, WC) were examined separately in single‑mediator models, with adjustment for covariates including LDL‑C, BMI, and WC (when not the mediator) plus all variables in Model 3. All analyses were done in R software (version 4.5.1) with a two-tailed significance threshold (*p* < 0.05). Considering that participants were assessed for gout over a 10-year period, a fixed-year effect was included in all models to account for the NHANES survey cycle. Collectively, these methodological approaches ensured the findings’ and conclusions’ robustness and reliability.

To assess whether missing caffeine data introduced bias, we performed a sensitivity analysis using the missing indicator method in a complete-case subsample where all covariates (including hypertension) were non‑missing (*n* = 3,717). A binary indicator variable (Caffeine_indicator) was created to denote observed versus missing caffeine intake and was added to the fully adjusted model (Model 3). If the effect estimate for AIP remained materially unchanged after inclusion of this indicator, and the indicator itself was not significantly associated with gout, it would suggest that missing caffeine data did not introduce substantial selection bias. Additionally, we calculated the E‑value to quantify the minimum strength of association that an unmeasured confounder would need to have with both AIP and gout to fully explain away the observed association (i.e., to shift the 95% confidence interval to include the null).

To control for false positives across multiple comparisons, we adopted a coherent strategy based on the type of analysis. For the primary logistic regression models examining AIP quartiles (Table 3), a nominal significance threshold of *p* < 0.05 was used because these were prespecified hypotheses. For subgroup analyses and interaction tests, we applied the BenjaminiHochberg false discovery rate (FDR) correction with a qvalue threshold of 0.05. For Mendelian randomization analyses involving multiple lipid exposures and gout outcomes, FDR correction was also applied across the three lipid traits (q < 0.05). For network pharmacology enrichment analyses (GO, KEGG, DO), only terms with FDRadjusted *p* < 0.05 were considered statistically significant. No correction was applied to the mediation analysis because the indirect effect was a single prespecified test. All FDR corrections were performed using the p.adjust function in R with method = “BH”.

### Two-sample MR analysis

A two-sample MR analysis was done in this study to study the potential causal relations between LDL-C, TG, gout, and HDL-C. Grounded on the random allocation of genetic variants during gamete formation, valid genetic instruments are required to satisfy three key assumptions: (1) Relevance assumption: the genetic variants should be powerfully linked with the exposure traits (lipid parameters); (2) Independence assumption: the genetic variants must be independent of any known or unknown confounders influencing the exposure–outcome relationship; (3) Exclusion restriction assumption: the genetic variants should affect the outcome (gout) solely via the exposure, without any alternative direct or indirect pathways [17].

#### Instrumental variable (IV) selection and data sources

We selected traits related to HDL-C (ieu-b-109, *n* = 403,943), LDL-C (ieu-b-110, *n* = 440,546), and TG (ieu-b-111, *n* = 441,016) from the UK IEU database to serve as exposures (or outcomes). To reduce selection bias, gout-associated traits (ebi-a-GCST90038687, *n* = 484,598) were retrieved from the EBI database as outcomes (or exposures) allowing us to explore potential bidirectional causal relations between them and gout.

For validation, we validated our findings using independent datasets. Specifically, we conducted multivariable Mendelian randomization (MVMR) analyses using two distinct outcome sources: (1) gout data from the ebi database, analyzed with the inverse variance weighted (MVMR-IVW) method; (2) gout-related trait data from the Finnish database (finngen_R12_M13_GOUT, *n* = 327,457); (3) gout-related trait data from the ukb database (ukb-b-13251, *n* = 462933). Detailed descriptions of the phenotypes and outcomes are provided in Appendix (Table 1).

**Table 1:** Core characteristic parameters of the six genome-wide relation studies included in this study

Trait category	GWAS ID	Trait name	Year	Trait number	Trait type	Sample size (cases/controls)	Ethnicity
Exposure/Outcome	ieu-b-109	HDL cholesterol,HDL-C	2020	/	Continuous	403,943	European
Exposure/Outcome	ieu-b-111	Triglycerides,TG	2020	/	Continuous	441,016	European
Exposure/Outcome	ieu-b-110	LDL cholesterol,LDL-C	2020	/	Continuous	440,546	European
Exposure/Outcome	ebi-a-GCST90038687	Gout	2021	/	Continuous	484,598	European
Exposure/Outcome	ukb-b-13251	Non-cancer illness code, self-reported: gout	2018	/	Continuous	462,933	European
Exposure/Outcome	https://storage.googleapis.com/finngen-public-data-r12/summary_stats/release/finngen_R12_M13_GOUT.gz	Gout	2023–2024	/	Continuous	327457(12342+315115)	European

Instrumental variables (IVs) were selected according to the following criteria: (1) genomewide significance threshold of *p* < 5 × 10^−8^; (2) linkage disequilibrium clumping with r^2^ < 0.001 within a 10,000 kb window; (3) exclusion of palindromic SNPs with minor allele frequency close to 0.5; (4) calculation of Fstatistics for each SNP, retaining only instruments with F > 10 to avoid weak instrument bias; (5) Steiger filtering to assess directional consistency (SNPs where variance explained in the outcome exceeded that in the exposure were excluded). The Fstatistic was calculated as F = R^2^ × [(*N* − 1 − k)/k] × (1 − R^2^), where R^2^ is the proportion of phenotypic variance explained by the IVs, N is the sample size, and k is the number of SNPs used [17–18] (File S1: Table S3–S11).

The IVW method was primarily employed in the MR analysis to investigate the potential bidirectional causal relations between HDL-C, TG, LDL-C, and gout. This approach is widely recognized for providing robust and accurate estimates when the core MR assumptions are satisfied. To strengthen our findings, we also employed additional complementary methods, such as the simple mode, weighted mode, MR-Egger regression (MRER), and weighted median method (WMM) to boost the results’ robustness and reliability [19–21]. The WMM yields reliable causal estimates even if 50% IVs are invalid or influenced by pleiotropy, making it particularly useful when some IVs do not fully satisfy the assumptions of the IVW approach [22]. The weighted and simple mode methods offer alternative strategies for assessing causal effects, additionally increasing the sensitivity of the analysis. MRER was applied to detect and correct for pleiotropy, ensuring that causal estimates are not biased by horizontal pleiotropy. Additionally, we conducted heterogeneity assessments, including the Cochran’s Q-test and Q-test, to evaluate consistency. Leave-one-out sensitivity analyses were performed within MR_PRESSO framework to recognize and remove results affected by multicollinearity or outliers, ensuring that findings were not determined by any single influential observation or substantial heterogeneity. All analyses were done utilizing R version 4.5.1, employing the TwoSampleMR package for MR analyses, and results were visualized with R graphics libraries to provide clear and accurate presentations.

#### Instrument validity Assessment

The strength of the IVs within the multivariable setting was assessed using conditional F-statistics. all well above the conventional threshold of 10, implying that weak instrument bias was unlikely (Table S3). Potential pleiotropy was assessed utilizing the intercept term from the multivariable MRER and Cochran’s Q test. Cochran’s Q test indicated substantial heterogeneity (*p* < 0.001), signifying the possible existence of pleiotropic effects. Accordingly, a random-effects multivariable IVW (MV-IVW) model was applied in the primary analysis to account for this heterogeneity.

MVMR analyses were done utilizing the TwoSampleMR package (version 0.5.7) [23] in R (version 4.5.1) [24]. The primary analysis employed the MV-IVW method, complemented by sensitivity analyses such as MR-Lasso, MR-median, and multivariable MR-Egger.

#### Statistical analysis

The primary analysis utilized the MV-IVW method to estimate causal effects. To assess the robustness of these findings, additional sensitivity analyses were carried out, included: 1) Multivariable MRER to detect and adjust for potential pleiotropic bias; 2) Multivariable MR-Lasso to address collinearity between exposures through variable selection; 3) Multivariable MR-Median, a robust method that is less sensitive to outliers; 4) MR-PRESSO, employed to recognize and eliminate outlier SNPs exhibiting significant horizontal pleiotropy; and 5) Single-SNP analysis utilizing the residual method to assess the individual contribution of each IV.

### Network pharmacological analysis

To further investigate the potential molecular targets and underlying mechanisms linking AIP, HDL-C and TG metabolism to gout, we conducted a network pharmacology analysis. First, genes involved in the three aforementioned biological processes were retrieved from the GeneCards database (https://www.genecards.org/) and the online mendelian inheritance(https://omim.org/) in man database utilizing keywords such as “TG, HDL-C, AIP, gout.” Next, the Jvenn tool [25] (Jvenn – Microbial Information – Free Online Venn Diagrams) was used to identify the intersections among target gene sets across the four biological processes. A hierarchical network pharmacology approach was then applied to further analyze these overlapping gene sets and perform target overlap analyses, enabling a systematic investigation of both shared and distinct molecular mechanisms linking AIP, HDL-C, TG, and gout. Moreover, the overlapping genes’ PPIs were examined using the STRING database, and a PPI network was generated with Cytoscape software (version 3.9.1). The core targets were identified based on a combined evaluation of node degree, closeness, and betweenness in the PPI network [26]. Finally, the overlapping targets were submitted to the DAVID database for KEGG and GO analyses, to explore disease associations, a Disease Ontology (DO) analysis. GO terms, DO_results and KEGG pathways with *p* values below 0.05 were considered statistically significant.

#### Identification of core targets via network pharmacology and CytoNCA

Using the same methodology, network pharmacology analyses were done to recognize potential targets and mechanisms for the following sets of relationships: AIP, TG, HDL-C, and gout; TG, HDL-C, and gout; TG and gout; and HDL-C and gout. Hub genes were identified using network topology metrics. The criteria were: degree centrality > median, betweenness centrality > median, and closeness centrality > median of the respective network. Genes satisfying all three thresholds were considered core targets. Additionally, the maximal clique centrality (MCC) algorithm in CytoNCA was used as an independent validation, retaining nodes with MCC score above the 90th percentile. All cutoffs were chosen based on the empirical distribution of each metric.

#### MR of core targets using Icelandic pQTL data

Exposure data were derived from protein quantitative trait loci (pQTL) data from an Icelandic population proteomics study, encompassing 3,598 circulating proteins. Based on core genes identified from prior network pharmacology and CytoNCA analysis, we extracted pQTL data corresponding to genes associated with AIP, TG/HDL-C, TG, and HDL-C as genetic instruments.

Outcome data for gout (GOUT) were integrated from summary statistics of three independent, large-scale genome-wide association study (GWAS) databases (see Table 1) to enhance the generalizability and robustness of the findings.

Single nucleotide polymorphisms (SNPs) significantly associated with each exposure protein were selected as instrumental variables based on the following criteria: Significance threshold: (*p* < 5 × 10^−8^).

Linkage disequilibrium (LD) clumping: SNPs were clumped to ensure independence (r^2^ < 0.001) within a 10,000 kb window). Instrument strength: The F-statistic was calculated for each SNP; only strong instruments (F > 10) were retained to mitigate weak instrument bias. Steiger filtering: SNPs potentially indicative of reverse causality (where R^2^_exposure_ > R^2^_outcome_) were excluded.

#### Mendelian randomization and statistical analysis

All analyses were performed using the “TwoSampleMR” R package (version 0.5.7). The analytical procedure was as follows: Data Harmonization: Effect alleles and strands were aligned between exposure and outcome datasets (parameter “action = 1”). Primary Analysis: The inverse-variance weighted (IVW) method was used as the primary approach for causal estimation. Sensitivity Analyses: To test the robustness of the primary findings, supplementary analyses were conducted using the weighted median, simple mode, weighted mode, and MR-Egger regression methods. Heterogeneity Assessment: Cochran’s Q statistic was used to evaluate heterogeneity across instrumental variable estimates. Pleiotropy Assessment: Horizontal pleiotropy was examined via the MR-Egger intercept test.

Outlier Detection and Correction: Potential outlier SNPs were identified and removed using the MR-PRESSO method (“NbDistribution = 1000”). Leave-One-Out Analysis: The MR analysis was repeated iteratively, excluding one SNP at a time, to assess if results were driven by a single influential variant.

Effect estimates from the IVW method are reported as odds ratios (OR) with 95% confidence intervals (CI) for intuitive interpretation of gout risk. A nominal significance threshold was set at (*p* < 0.05). Considering the multiple testing across exposure proteins, False Discovery Rate (FDR) correction was applied using the total number of proteins analyzed (*n* = 3598) as the correction factor. All statistical analyses were performed in R version 4.5.1, utilizing packages such as “data. table”, “dplyr”, and “parallel” for efficient data processing and parallel computing.

## Results

### Baseline characteristics

A total of 6,985 individuals were included (Figure S1, S2), corresponding to a weighted population of 260,784,688 individuals with a mean age of 62.07 ± 13.23 years. Among them, 719 participants (10.29%) were diagnosed with gout, while 6,266 (89.71%) did not have gout. The study population’s baseline characteristics stratified by gout status are represented in Table 2. Notably, participants with gout had significantly higher levels of TG and AIP, and lower levels of HDL‑C, compared to those without gout. LDL‑C levels did not differ between the two groups. Additionally, male sex, NHW ethnicity, obesity, larger WC, smoking, alcohol consumption, hypertension, and diabetes were significantly associated with gout (all *p* < 0.001). PA and caffeine intake were not significantly associated with gout. Weighted baseline characteristics of the study participants (File S1: Table S12).

**Table 2:** Baseline characteristics of the study participants

	level	Overall	Gout No	Gout Yes	p	Statistic
n		6985	6266	719		
Gender (%)	Female	3711 (53.1)	3479 (55.5)	232 (32.3)	<0.001	χ^2^ = 139.137
	Male	3274 (46.9)	2787 (44.5)	487 (67.7)		
Age (years)		62.07 (13.23)	61.65 (13.36)	65.78 (11.34)	<0.001	t = −9.083
Race (%)	Mexican American	745 (10.7)	701 (11.2)	44 (6.1)	<0.001	χ^2^ = 25.795
	Non-Hispanic Black	1978 (28.3)	1754 (28.0)	224 (31.2)		
	Non-Hispanic White	3048 (43.6)	2708 (43.2)	340 (47.3)		
	Other Hispanic	605 (8.7)	560 (8.9)	45 (6.3)		
	Other Race - Including Multi-Racial	609 (8.7)	543 (8.7)	66 (9.2)		
Caffeine1 (mg)		148.09 (195.52)	147.97 (196.37)	149.16 (188.04)	0.877	t = −0.16
Caffeine2 (mg)		132.70 (168.86)	132.42 (168.57)	135.08 (171.46)	0.69	t = −0.394
exposure. AIP (mmol/L)		−0.01 (0.33)	−0.02 (0.33)	0.07 (0.36)	<0.001	t = −6.415
HDL-C (mmol/L)		1.35 (0.42)	1.36 (0.42)	1.25 (0.41)	<0.001	t = 6.679
TG (mmol/L)		1.53 (1.37)	1.50 (1.21)	1.79 (2.31)	<0.001	t = −3.298
LDL-C (mmol/L)		2.91 (0.97)	2.92 (0.97)	2.83 (1.01)	0.017	t = 2.312
BMI (kg/m2)		31.67 (7.50)	31.51 (7.42)	33.08 (8.05)	<0.001	t = −4.978
WC (cm)		107.00 (16.38)	106.38 (16.18)	112.43 (17.13)	<0.001	t = −9.023
diabetes (%)	Borderline	304 (4.4)	273 (4.4)	31 (4.3)	<0.001	χ^2^ = 47.108
	No	4690 (67.1)	4285 (68.4)	405 (56.3)		
	Yes	1991 (28.5)	1708 (27.3)	283 (39.4)		
Smoking (%)	No	3521 (50.4)	3216 (51.3)	305 (42.4)	<0.001	χ^2^ = 20.104
	Yes	3464 (49.6)	3050 (48.7)	414 (57.6)		
Alcohol (%)	No	1915 (27.4)	1763 (28.1)	152 (21.1)	<0.001	χ^2^ = 15.512
	Yes	5070 (72.6)	4503 (71.9)	567 (78.9)		
HBP (%)	No	877 (12.6)	809 (12.9)	68 (9.5)	0.01	χ^2^ = 6.695
	Yes	6108 (87.4)	5457 (87.1)	651 (90.5)		
Physical Activity (%)	No	6221 (89.1)	5574 (89.0)	647 (90.0)	0.438	χ^2^ = 0.6
	Yes	764 (10.9)	692 (11.0)	72 (10.0)		

In univariate analysis (Table 2), both hypertension and diabetes were associated with gout (both *p* < 0.001). However, in the fully adjusted model (Model 3, Table 3), hypertension showed a borderline association (OR = 4.591, 95% CI: 0.996–21.174, *p* = 0.051) that did not reach statistical significance, while diabetes was no longer significant (*p* > 0.05).

**Table 3:** Association between AIP_TG_HDL-C and gout

Exposure	Model 1 OR (95% CI)	Model 2 OR (95% CI) p-value	Model 3 OR (95% CI)
	P **value**		P **value**
AIP (quartiles)			
Q1(lowest)	ref	ref	ref
Q2	1.074 (0.763, 1.512) 0.68222	1.064 (0.755, 1.499)	1.016 (0.716, 1.443)
		0.72363	0.92856
Q3	1.319 (0.939, 1.851) 0.11430	1.275 (0.902, 1.801)	1.125 (0.791, 1.600)
		0.17269	0.51355
Q4(highest)	2.137 (1.525, 2.994) 0.00003	1.962 (1.332, 2.888)	1.700 (1.146, 2.521)
		0.00108	0.01041
TG (quartiles)			
Q1(lowest)	ref	ref	ref
Q2	1.290 (0.878, 1.895) 0.19831	1.297 (0.879, 1.913) 0.19380	1.212 (0.805, 1.824) 0.36056
Q3	1.240 (0.886, 1.736) 0.21430	1.208 (0.851, 1.715) 0.29463	1.089 (0.758, 1.565) 0.64529
Q4(highest)	1.929 (1.363, 2.730) 0.00039	1.886 (1.309, 2.718) 0.00111	1.666 (1.153, 2.408) 0.00836
HDL-C (quartiles)			
Q1(lowest)	ref	ref	ref
Q2	0.624 (0.446, 0.875) 0.00767	0.643 (0.449, 0.922) 0.01888	0.674 (0.468, 0.970)
			0.03757
Q3	0.406 (0.307, 0.537)	0.444 (0.323, 0.611)	0.483(0.345, 0.675)
	<0.00001	<0.00001	0.00007
Q4(highest)	0.468 (0.334, 0.657) 0.00004	0.523 (0.341, 0.803) 0.00414	0.623 (0.399, 0.974)
			0.04195
Model 1: adjusted for no covariates.			
Model 2: adjusted for variables of age, gender, race.			
Model 3: adjusted for variables of age, gender, race, BMI, diabetes, hypertension, smoking, alcohol consumption, and PA.			

### Association between AIP and gout

Logistic regression analyses were performed to link the relation between lipid parameters and gout risk. As presented in Table 3, individuals in the highest quartile of AIP had a significantly increased risk of gout across all three models. In the fully adjusted model (Model 3), participants in the AIP Q4 group had 1.70 times the risk of gout compared to those in the Q1 group (OR = 1.700, 95% CI: 1.146–2.521, *p* = 0.010). Similarly, TG levels were positively linked with gout risk. In Model 3, participants in the highest quartile of TG (Q4) had a 1.67-fold higher risk of gout than those in Q1 (OR = 1.666, 95% CI: 1.153–2.408, *p* = 0.008), indicating that this link remained robust and independent after adjustment for demographic, lifestyle, and comorbid factors. In contrast, HDL-C demonstrated a protective effect: in the fully adjusted model, participants in the highest HDL-C quartile (Q4) had a significantly lower risk of gout (OR = 0.623, 95% CI: 0.399–0.974, *p* = 0.042), with a consistent decreasing trend across quartiles.

The results demonstrate that elevated AIP, higher TG levels, and lower HDL-C levels are each independently linked with a raised incidence and severity of gout, exhibiting a clear dose–response relation. These outcomes underscore the main role of dyslipidemia in the pathogenesis and progression of gout. All analyses accounted for the NHANES complex sampling design, with weighted analyses conducted using the survey package in R.

### Restricted cubic splines (RCS) model and subgroup analysis

**Fig. 2:**
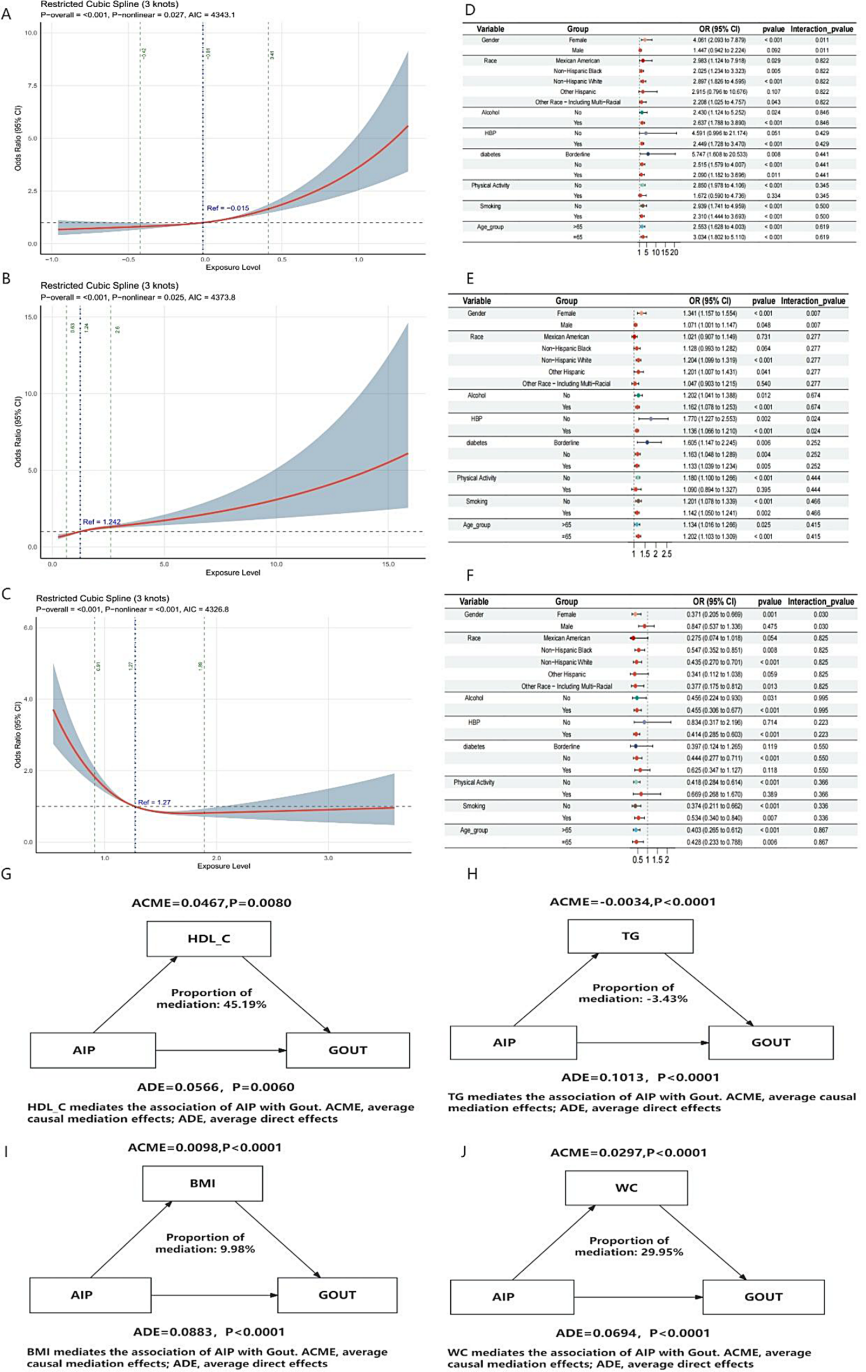
RCS, subgroup and mediation analyses for AIP, lipids and gout. (**A–C**) Restricted cubic spline (RCS) analyses were performed to examine the dose–response associations of continuous atherogenic index of plasma (AIP), triglycerides (TG), and high-density lipoprotein cholesterol (HDL-C) with gout, adjusted for potential confounders. (**D–F**) Subgroup analyses and forest plots were used to assess the consistency of associations between AIP, TG, and HDL-C with gout across different demographic and clinical subgroups. (**G–J**) Mediation models were applied to evaluate the mediating effects of HDL-C, body mass index (BMI), and waist circumference (WC) on the association between AIP and gout, as well as the direct effect of TG on gout. ACME, average causal mediation effects; ADE, average direct effects

Supplementary Table S21 presents the knot positions (in original units) for AIP, TG, and HDL‑C. Supplementary Table S22 summarizes the AIC values for each knot configuration. Based on the lowest AIC, the 3‑knot model was selected for all three exposures (AIP: AIC = 4343.1; TG: AIC = 4373.8; HDL‑C: AIC = 4326.8). Using these specifications, the test for non‑linearity was significant for AIP (*p* = 0.027) and HDL‑C (*p* < 0.001), but not for TG (*p* = 0.052). The corresponding dose‑response curves are shown in Fig. 2.

Firstly, consistent and significant associations between AIP and gout were observed in both RCS analyses (Figure S3) and subgroup analyses. The RCS analysis (Fig. 2A) revealed a significant positive correlation between AIP and gout risk (overall association test: *p* < 0.001), along with a statistically significant nonlinear relationship (nonlinearity test: *p* = 0.027). Among the various knot models, the three-knot model gave the best fit, as illustrated by the lowest AIC value, demonstrating a robust nonlinear dose–response relationship. These outcomes suggest that as AIP levels increase, gout risk rises in a nonlinear manner, indicating that AIP may act as an independent risk factor for gout.

Secondly, subgroup analyses highlighted heterogeneity in the AIP–gout association across different population groups (Fig. 2D). Notably: Racial differences: Racial differences: The interaction term between AIP and race was not significant (P_interaction = 0.36), suggesting no evidence of effect modification by race. The subgroup-specific ORs are shown in Fig. 2D. Lifestyle impact: Alcohol drinking showed a positive association (OR = 2.637, 95% CI: 1.788–3.890, *p* < 0.001). PA differences: The association did not differ significantly between active and inactive individuals (active: OR = 1.672; inactive: OR = 2.850). Comorbidity status: Participants with hypertension showed a borderline association (OR = 4.591, 95% CI: 0.996–21.174, *p* = 0.051) that did not reach statistical significance, while those with diabetes had a significantly strengthened association (OR = 5.747, 95% CI: 1.608–20.533, *p* = 0.008). Age stratification: The association appeared more pronounced in individuals aged ≥ 65 years (OR = 3.034, 95% CI: 1.802–5.110, *p* < 0.001) than in those < 65 years old (exploratory finding).

Lastly, as TG and HDL-C are key components of AIP and important indicators of lipid metabolism, their associations with gout further support the AIP findings and clarify the roles of specific lipid fractions. RCS analysis for TG (Fig. 2B) showed a significant positive correlation with gout risk (overall *p* < 0.001) and a nonlinear trend (nonlinearity *p* = 0.025), consistent with the AIP results. Subgroup analyses revealed the strongest TG–gout association in NHW individuals (OR = 1.204, 95% CI: 1.099–1.319, *p* < 0.001), with elevated TG-related gout risks also observed among alcohol drinkers and participants with hypertension or diabetes, aligning with the subgroup trends observed for AIP (Fig. 2E).

Notably, the link between HDL-C and gout differs from those observed for AIP and TG. HDL-C was significantly inversely linked with gout risk (overall *p* < 0.001), and RCS analysis revealed a significant nonlinear relationship (nonlinearity *p* < 0.001), implying that higher HDL-C levels may confer a protective effect against gout (Fig. 2C). Subgroup analysis (Fig. 2F) indicated that this protective effect was particularly pronounced in the NHB population (OR = 0.547, 95% CI: 0.352–0.851, *p* = 0.008), further supporting a potential beneficial role of HDL-C in gout prevention.

Examine the associations between GOUT and TG, HDL-C, and AIP. (A) Evaluate the relationship between AIP and GOUT using RCS. (B) Evaluate the relationship between HDL-C and GOUT using RCS. (C) Evaluate the relationship between TG and GOUT using RCS. The red solid line represents the smoothed curve of OR values for GOUT in relation to TG, HDL-C, and AIP, while the blue band shows the 95% CI of the fit. Associations of TG, HDL-C, and AIP with GOUT were further examined in diverse subgroups via RCS analysis. (D) Perform RCS analysis to determine the link between AIP and GOUT stratified by AIP levels. (E) Perform RCS analysis to measure the link between HDL-C and GOUT stratified by hypertension status. (F) Perform RCS analysis to assess the link between TG and GOUT stratified by gender.

### The impact of mediation effects on the relation between AIP and gout

Further mediation analysis, incorporating AIP components (HDL-C and TG) and covariates (LDL-C, WC, and BMI), was performed to clarify the AIP–gout relationship. The analysis identified HDL-C (Mediation = 45.19%), WC (Mediation = 29.95%), and BMI (Mediation = 9.98%) as significant mediators, whereas TG and LDL-C did not demonstrate a mediating role. Notably, despite the absence of a significant indirect effect through AIP, TG exhibited a highly significant total effect on gout, driven by a strong direct effect independent of the AIP pathway (ADE = 0.1013, *p* < 2 × 10^−16^) (Fig. 2G, H, I, J) (File S1: Table S13–S17).

### Sensitivity analysis for missing caffeine data

In the complete-case subsample (*n* = 3,717), the association between AIP and gout remained robust. After adding the caffeine missing indicator to the fully adjusted model, the OR for AIP Q4 vs. Q1 was unchanged (OR = 2.42; 95% CI: 1.37–4.28; *p* = 0.0035), nearly identical to the estimate without the indicator (OR = 2.42; 95% CI: 1.37–4.28; *p* = 0.0034). The missing indicator itself was not significantly associated with gout (OR = 1.36; 95% CI: 0.74–2.49; *p* = 0.3263). The Evalue for the AIP–gout association was 2.09, indicating that an unmeasured confounder would need to be associated with both AIP and gout by a risk ratio of at least 2.09 to explain away the observed effect. Such a strong confounder is highly unlikely in the context of gout. These findings support the robustness of our main results (File S1: Table S20).

### Univariable MR

**Fig. 3:**
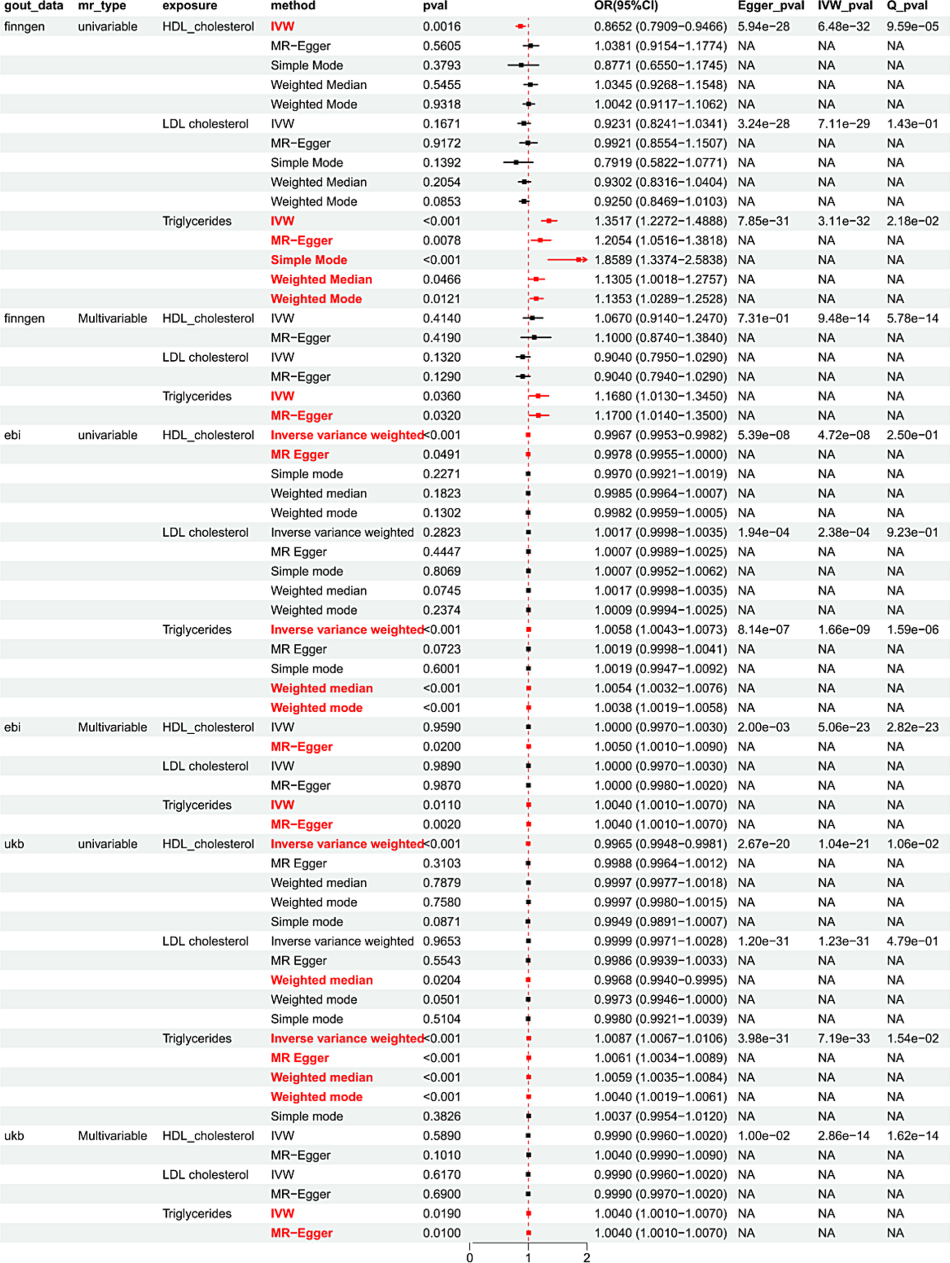
Results of univariable (multivariable) MR Analysis

The associations between genetically predicted lipid traits and gout are summarized in Fig. 3. Using the IVW method, TG was positively associated with gout risk (OR > 1, *p* < 0.001), suggesting that higher TG levels may increase the likelihood of developing gout. Conversely, HDL-C was inversely associated with gout (OR < 1, *p* < 0.05), suggesting a potential protective effect in univariable analysis. Genetically predicted LDL-C showed no significant association with gout, contrasting with observational results.

Reverse MR suggested a possible bidirectional association (OR = 51.07, 95% CI: 1.35–1925), but this estimate was highly unstable due to extreme heterogeneity (I^2^ = 99.4%) and a very wide confidence interval; therefore, no robust evidence for reverse causality was found. Thus, we do not claim evidence for reverse causation.

Importantly, all MR analyses showed extreme heterogeneity (I^2^ > 99%), which violates key assumptions of the fixed‑effect model. Although we performed additional sensitivity analyses (MR-PRESSO, weighted median, and outlier removal) and the direction of effect remained consistent, the results should be interpreted with great caution and are not considered robust causal evidence. Detailed outcomes are provided in Table 1 and Supplementary Figures S4–S6.

### Multivariable MR

Multivariable MR analysis provided statistically substantial evidence of a causal relation between genetically predicted TG and gout (OR > 1, *p* < 0.001). This indicates that TG exerts a positive causal effect on gout, consistent with the results from the univariable analysis. Importantly, after adjusting for other relevant lipid exposures, the results were consistent across the three databases. TG retained a significant and independent causal association with gout. This effect was consistently observed across all four MR methods (IVW, MR‑Egger, weighted median, and weighted mode). In contrast, HDL-C and LDL-C did not demonstrate significant causal effects in any of the methods (*p* > 0.05) (Fig. 3, File S1: Table S18).

The MR‑Egger intercept was not statistically significant (*p* = 0.123), indicating a low likelihood of horizontal pleiotropy. Although significant heterogeneity was observed across all methods (*p* < 0.001), the direction of the causal estimates was consistent across different MR approaches; however, due to the extreme heterogeneity, these results should be interpreted with caution and are not considered robust causal evidence (Tables 4 and 5, File S1: Table S19).

**Table 4:** Sensitive analysis of the association (univariable)

Outcome	Exposure	Pleiotropy			Heterogenenity					
		MR_egger_intercept	MR_egger_se	MR_egger_Pval	IVW_Q	IVW_Q_df	IVW_Q_pval	MR_egger_Q	MR_egger_Q_df	MR_egger_Q_pval
finngen_R12_M13_Gout	HDL-Cholesterol	6.42E+02	2.94E+02	5.94E-28	6.76E+02	2.95E+02	6.48E-32	−8.18E-03	2.07E-03	9.59E-05
	Triglycerides	6.20E+02	2.63E+02	7.85E-31	6.32E+02	2.64E+02	3.11E-32	5.13E-03	2.22E-03	2.18E-02
	LDL-Cholesterol	4.19E+02	1.46E+02	3.24E-28	4.25E+02	1.47E+02	7.11E-29	−4.66E-03	3.17E-03	1.43E-01
HDL-Cholesterol	Gout	2.20E+02	1.60E+01	5.71E-38	2.27E+02	1.70E+01	9.09E-39	−2.87E-03	4.09E-03	4.93E-01
Triglycerides	Gout	2.31E+03	1.60E+01	0.00E+00	2.66E+03	1.70E+01	0.00E+00	2.16E-02	1.38E-02	1.37E-01
LDL-Cholesterol	Gout	3.70E+02	1.60E+01	8.52E-69	3.79E+02	1.70E+01	4.63E-70	3.69E-03	5.79E-03	5.33E-01
ebi-a-GCST90038687_Gout	HDL-Cholesterol	4.48E+02	2.99E+02	5.39E-08	4.50E+02	3.00E+02	4.72E-08	−3.76E-05	3.26E-05	2.50E-01
	Triglycerides	3.92E+02	2.66E+02	8.14E-07	4.27E+02	2.67E+02	1.66E-09	1.55E-04	3.15E-05	1.59E-06
	LDL-Cholesterol	2.16E+02	1.47E+02	1.94E-04	2.16E+02	1.48E+02	2.38E-04	3.75E-06	3.89E-05	9.23E-01
HDL-Cholesterol	Gout	6.76E+02	2.50E+01	1.20E-126	7.14E+02	2.60E+01	8.85E-134	−4.18E-03	3.55E-03	2.50E-01
Triglycerides	Gout	4.12E+03	2.50E+01	0.00E+00	4.20E+03	2.60E+01	0.00E+00	6.62E-03	9.14E-03	4.76E-01
LDL-Cholesterol	Gout	6.35E+02	2.50E+01	7.21E-118	6.47E+02	2.60E+01	1.07E-119	−2.60E-03	3.77E-03	4.96E-01
ukb-b-13251_Gout	HDL-Cholesterol	5.06E+02	2.45E+02	2.67E-20	5.20E+02	2.46E+02	1.04E-21	−9.71E-05	3.77E-05	1.06E-02
	Triglycerides	5.75E+02	2.32E+02	3.98E-31	5.90E+02	2.33E+02	7.19E-33	1.04E-04	4.26E-05	1.54E-02
	LDL-Cholesterol	4.09E+02	1.26E+02	1.20E-31	4.11E+02	1.27E+02	1.23E-31	5.45E-05	7.67E-05	4.79E-01
HDL-Cholesterol	Gout	4.93E+02	1.80E+01	2.76E-93	5.66E+02	1.90E+01	8.95E-108	−6.58E-03	4.05E-03	1.21E-01
Triglycerides	Gout	3.94E+03	1.80E+01	0.00E+00	4.11E+03	1.90E+01	0.00E+00	1.05E-02	1.19E-02	3.91E-01
LDL-Cholesterol	Gout	4.71E+02	1.80E+01	1.49E-88	4.77E+02	1.90E+01	2.99E-89	−2.20E-03	4.33E-03	6.18E-01

**Table 5:** Sensitive analysis of the association (multivariable)

Assessment	ebi-a-GCST90038687	finngen_R12_M13_GOUT	ukb-b-13251	Criteria
Instrument Strength				
- HDL-C conditional F-statistic	246.75	252.77	320.46	>10 (all > 10)
- LDL-C conditional F-statistic	422.96	418.82	412.98	>10 (all > 10)
- TG conditional F-statistic	276.18	272.31	317.6	>10 (all > 10)
Heterogeneity Test				
- Cochran’s Q *p*-value	5.06 × 10^−23^	9.48 × 10^−14^	2.86 × 10^−14^	*p* < 0.05 (all significant)
- I^2^ statistic (%)	99.7	99.6	99.7	>75% (all > 75%)
Pleiotropy Test				
- Q-statistic *p*-value	2.82 × 10^−23^	5.78 × 10^−14^	1.62 × 10^−14^	*p* < 0.05 (all significant)
MR-Egger Intercept				
- Intercept (*p*-value)	0.000 (0.002)	−0.002 (0.731)	0.000 (0.010)	*p* < 0.05 (mixed results)

### Network pharmacological analysis

**Fig. 4:**
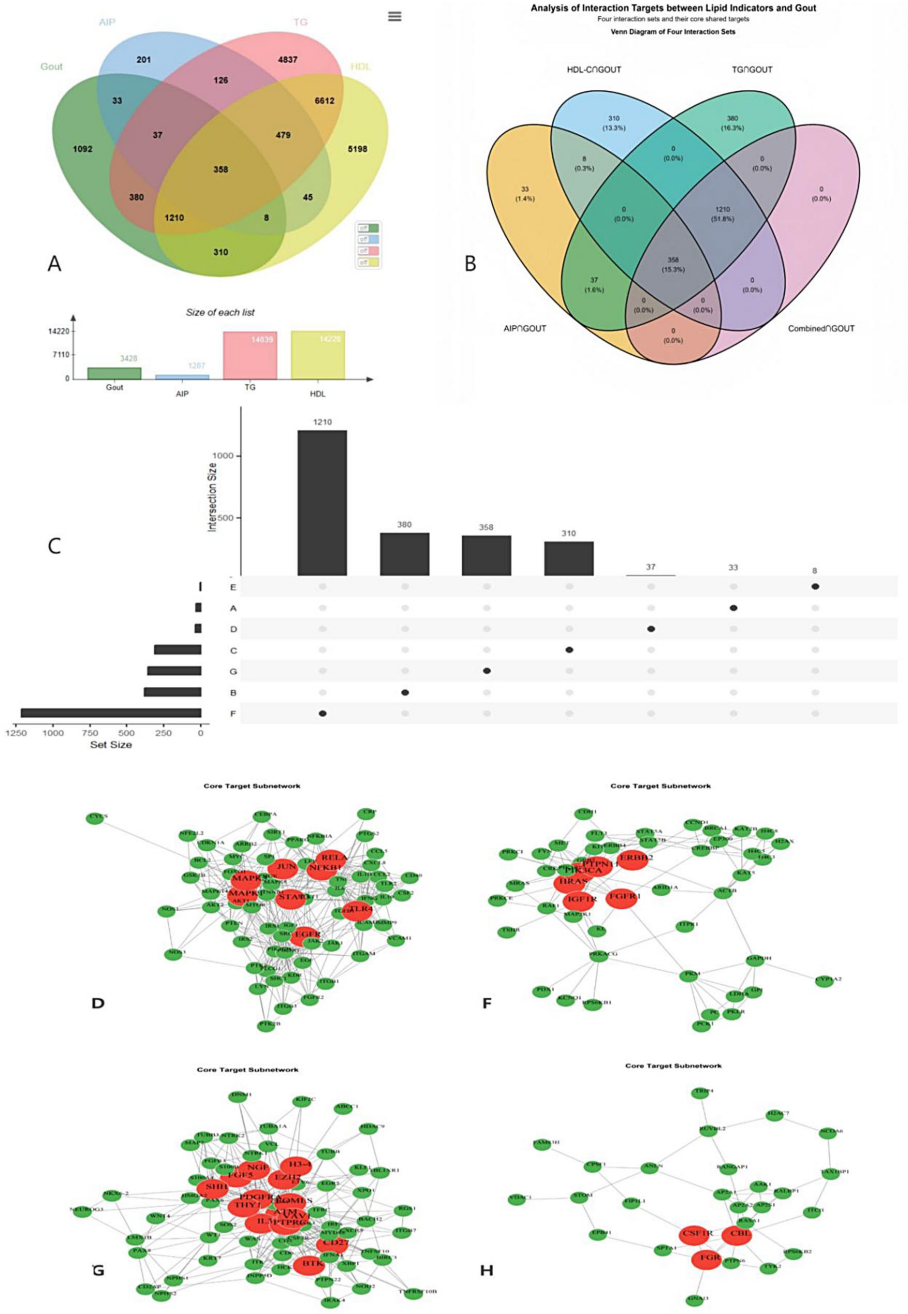
Analysis of interaction targets between lipid indicators and gout. (**A**) Venn diagram illustrating the overlapping targets among atherogenic index of plasma (AIP), triglycerides (TG), high-density lipoprotein cholesterol (HDL-C), and gout. (**B**) Venn diagram comparing the shared targets across four target sets: the AIP set (targets of AIP, TG, HDL-C, and gout), the combined set (targets of TG, HDL-C, and gout), the TG set (targets of TG and gout), and the HDL-C set (targets of HDL-C and gout). (**C**) Proportional distribution of targets within each of the four target sets. (**D**) Core gene subnetwork derived from the AIP target set. (**E**) Core gene subnetwork derived from the combined (TG_HDL-C) target set. (**F**) Core gene subnetwork derived from the TG target set. (**G**) Core gene subnetwork derived from the HDL-C target set

To explore the molecular mechanisms linking AIP, TG, HDL‑C and gout, we retrieved target genes from GeneCards and OMIM. After intersection analysis (Fig. 4A–C), we obtained seven target gene sets: G (*n* = 358, 15.3%; intersection of all four), F (*n* = 1,210, 51.8%; TG+HDL‑C), D (*n* = 37), E (*n* = 8), B (*n* = 380, 16.3%; TG‑specific), C (*n* = 310, 13.3%; HDL‑C‑specific), and A (*n* = 33). Groups A and D had very low human gene matching rates (0.61% and 0.70%) and were excluded; group E was too small (*n* = 8) to yield a meaningful PPI network.

KEGG enrichment, PPI network construction (STRING), and core hub gene identification (CytoNCA) were performed on the remaining four gene sets (G, F, B, C). The most robust findings are summarized below (full data in File S2):

1. TG‑specific gene set (Group B) – significantly enriched in T‑cell receptor signaling pathway (hsa04660, *p* = 2.3 × 10^−6^) and NF‑κB signaling pathway (hsa04064, *p* = 1.8 × 10^−5^). Hub genes included PTPRC (CD45), MYD88, and LCK (Figure 4F, File S2: Tables S12–S15).
2. Combined TG/HDL‑C gene set (Group F) – enriched in PI3K‑Akt signaling pathway (hsa04151, *p* = 3.2 × 10^−5^) and cytokine‑cytokine receptor interaction (hsa04060, *p* = 4.5 × 10^−5^) (Figure 4E).
3. HDL‑C‑specific gene set (Group C) – involved in fundamental cellular processes such as cytoskeleton organization (GO:0007010, *p* = 0.002) and endocytic transport (GO:0006897, *p* = 0.008), with no marked enrichment of inflammatory pathways (Fig. 4G). Hub genes included CBL and RASA1.

These findings are exploratory and require experimental validation.

### PPI network construction

**Fig. 5:**
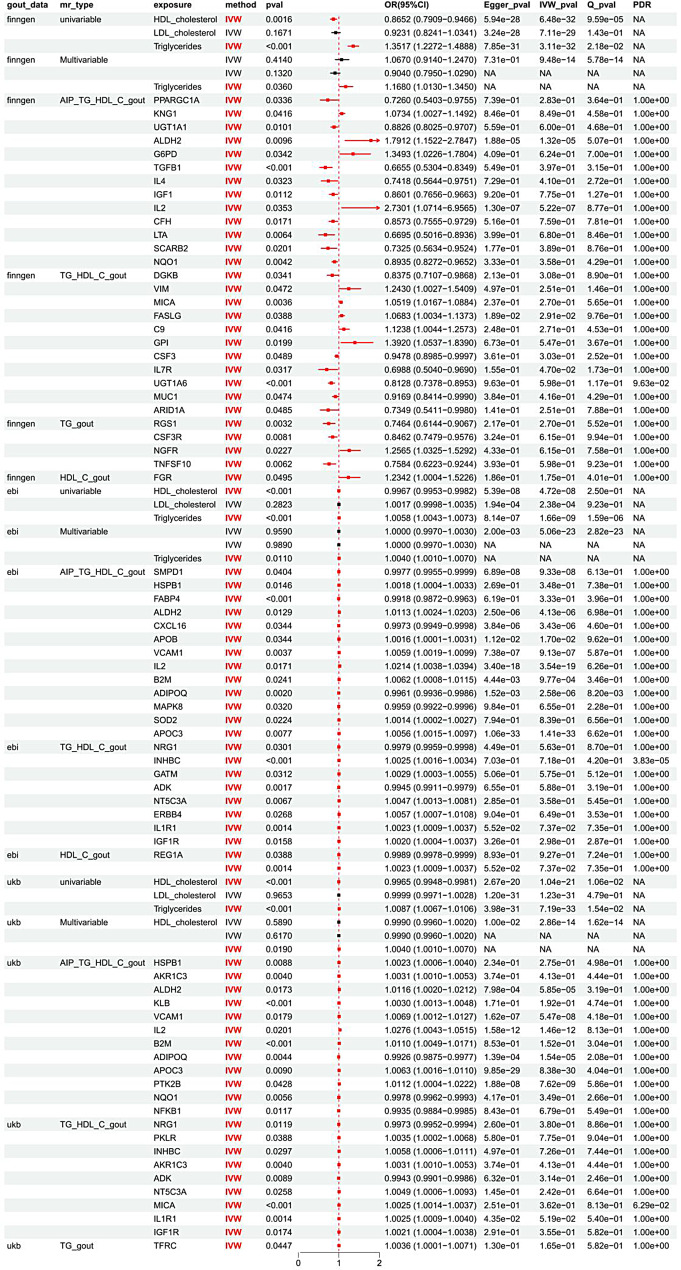
Forest plot of Mendelian randomization analysis using Icelandic pQTL data

PPI networks for the four main gene sets (G, F, B, C) were constructed using STRING (Fig. 5). Hub genes were identified via CytoNCA using degree, betweenness, and closeness centrality (cutoffs set at the median). Full metrics are in File S2: Tables S12–S15.

#### Core hub genes

The AIP‑related network (Group G) yielded 8 hub genes, including STAT3 (degree = 52, betweenness = 892) and NFKB1 (degree = 47, betweenness = 754). The TG/HDL‑C network (Group F) yielded 6 hubs, including ERBB2 (degree = 38, betweenness = 612) and PIK3CA (degree = 35, betweenness = 578) (Fig. 4D–E).

#### pQTL‑based MR analysis

Using Icelandic pQTL data (3,598 circulating proteins), we performed two‑sample MR (IVW method, FDR correction). At a nominal threshold *p* < 0.05, 74 proteins were associated with gout risk; after FDR correction, 54 remained significant (Fig. 5). The most significant association was INHBC (OR = 0.67, 95% CI: 0.55–0.81, FDR = 3.8 × 10^−5^). UGT1A6 (OR = 0.81, FDR = 0.08) and MICA (OR = 1.12, FDR = 0.09) showed borderline significance. Full results in File S2: Tables S16–S25.

#### Distinct genetic sets for AIP vs TG/HDL‑C

**Fig. 6:**
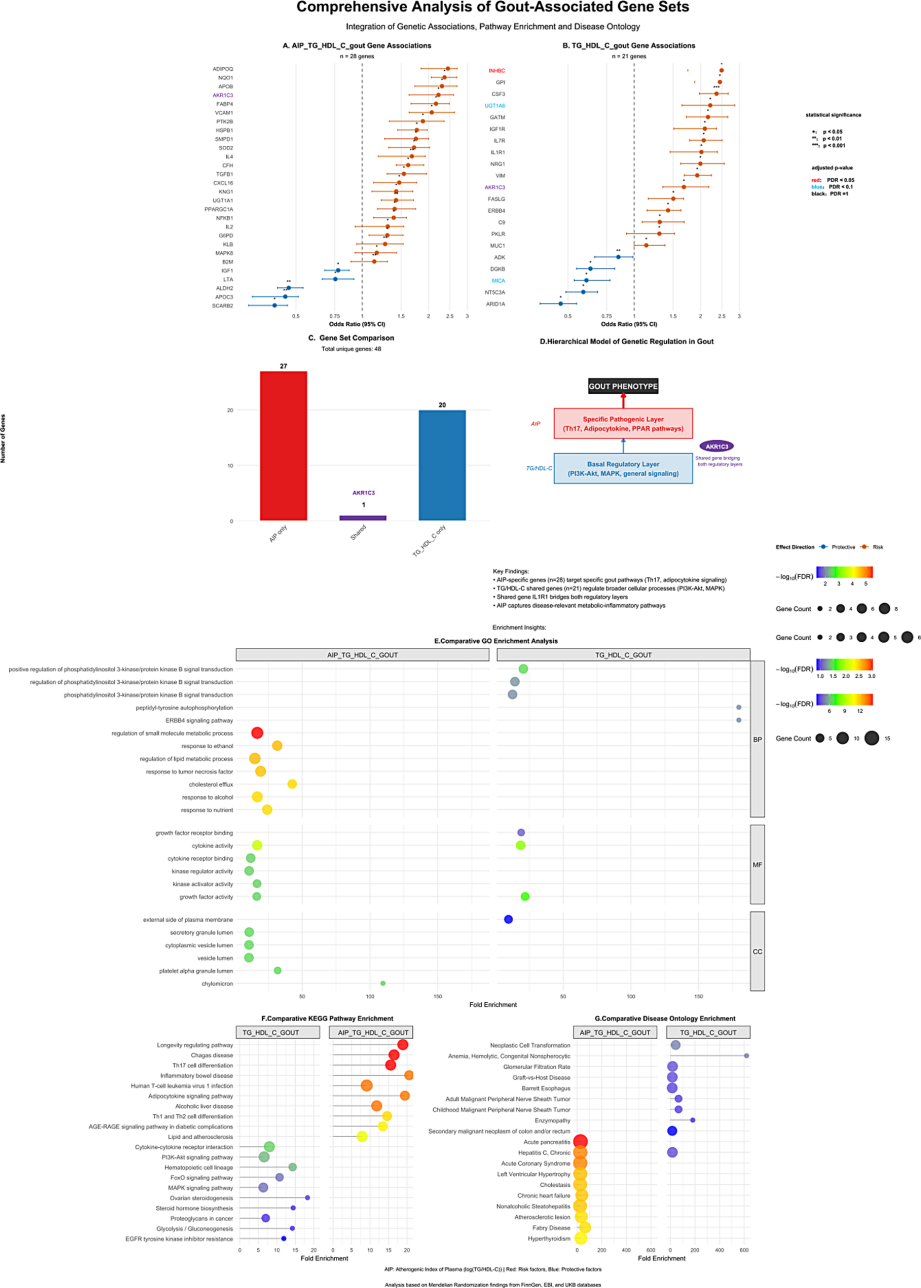
Comprehensive analysis of gout-associated gene sets. (**A, B**) Forest plots of Mendelian randomization (MR) results showing causal associations between genetic variants of two gene sets (AIP, TG, HDL-C, gout; and TG, HDL-C, gout) and gout risk. (**C**) Bar chart comparing the number of genes and overlap between the two gene sets. (**D**) Schematic model illustrating the hierarchical genetic regulation and key pathways underlying gout pathogenesis for the two gene sets. (**E–G**) Comparative enrichment analyses of gene ontology (GO) biological processes, kyoto encyclopedia of genes and genomes (KEGG) pathways, and disease Ontology (DO) terms for the two gene sets, highlighting shared and distinct functional and disease associations

The MR analysis identified largely non‑overlapping gene sets: 28 genes in the AIP framework (Group G) and 21 genes in the TG/HDL‑C framework (Group F), sharing only AKR1C3 (Fig. 6A–C). This suggests that AIP and TG/HDL‑C influence gout risk through predominantly different genetic pathways.

#### Functional profiling of MR‑derived gene sets

Enrichment analyses (GO, KEGG, DO) revealed that the AIP‑related gene set was enriched in regulation of small molecule metabolic process (*p* = 3.2 × 10^−6^) and response to ethanol (*p* = 2.4 × 10^−5^). KEGG pathways included Th17 cell differentiation (*p* = 2.1 × 10^−5^) and inflammatory bowel disease (*p* = 3.4 × 10^−5^) (Fig. 6E–F). The TG/HDL‑C‑related gene set was enriched in positive regulation of PI3K‑Akt signal transduction (*p* = 3.4 × 10^−6^) and cytokine‑cytokine receptor interaction (*p* = 4.7 × 10^−5^) (Fig. 6E–F). Disease Ontology associations are shown in Figure 6G (full data in File S2).

#### Network topology and core hub functions

**Fig. 7:**
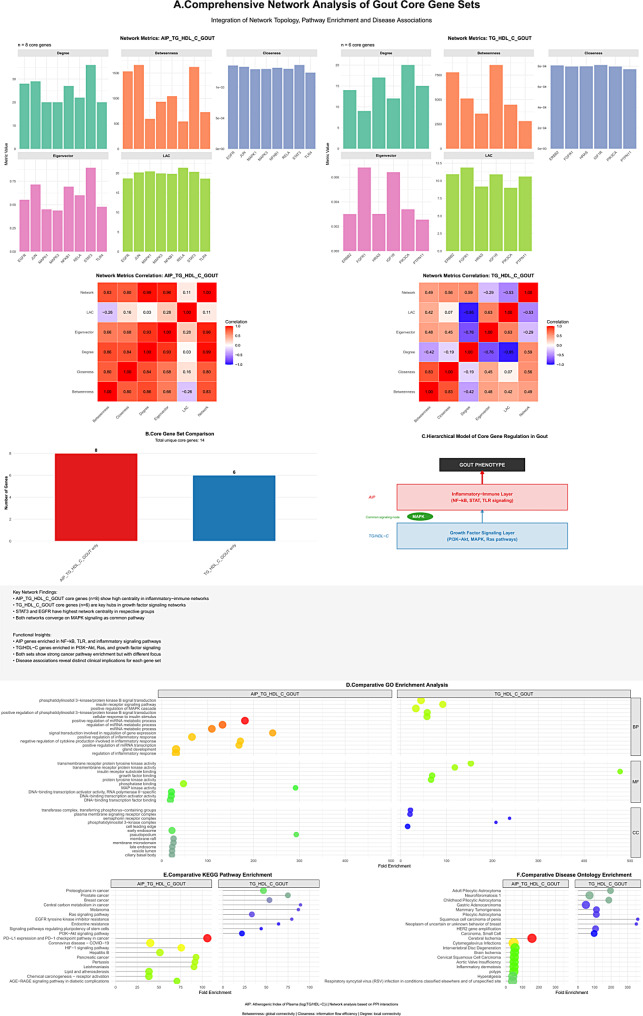
Comprehensive network analysis of gout core gene sets. (**A**) Network topology metrics (degree, betweenness, closeness) and their correlations for two core gene sets (AIP, TG, HDL-C, gout; and TG, HDL-C, gout), constructed from protein-protein interactions. (**B**) Bar chart comparing the number of unique and overlapping genes between the two core gene sets. (**C**) Hierarchical model illustrating the regulatory layers of core genes in gout pathogenesis, including inflammatory and growth factor signaling pathways. (**D–F**) Comparative enrichment analyses of gene ontology (GO) biological processes, kyoto encyclopedia of genes and genomes (KEGG) pathways, and disease ontology (DO) terms, highlighting shared and distinct functions and disease associations of the two core gene sets

Topological analysis confirmed that STAT3 and NFKB1 formed the inflammatory‑immune core of the AIP network, while ERBB2 and PIK3CA anchored the TG/HDL‑C network (Fig. 7A–C). Functional analysis of core AIP hub genes (*n* = 8) highlighted positive regulation of miRNA metabolic process (*p* = 2.6 × 10^−8^) and PD‑L1/PD‑1 checkpoint pathway (fold enrichment = 28.97, *p* = 7.1 × 10^−15^). Core TG/HDL‑C hub genes (*n* = 6) were involved in PI3K‑Akt signaling, insulin receptor pathway, and MAPK cascade (Fig. 7D–F; full data in File S2).

#### Specific associations of TG and HDL‑C with gout

Separate MR analyses for TG and HDL‑C alone identified 5 genes for TG and 3 genes for HDL‑C with nominal significance (*p* < 0.05), including IL1R1 for HDL‑C (OR = 1.0023, *p* = 0.0014). However, none remained significant after FDR correction (all FDR > 0.1). PPI networks revealed distinct hub genes: TG_gout network contained 14 core hubs (e.g., PTPRC, EZH2, PDGFRA); HDL‑C_gout network contained 3 hubs (CBL, FGR). These results suggest that TG and HDL‑C influence gout through partially non‑overlapping genetic networks when considered separately (Figure S2, File S2: Tables S31–S35).

However, due to the limited number of genes, GO/KEGG/DO enrichment analyses did not yield statistically significant results following multiple testing correction. This limitation itself, however, underscores how the use of AIP as a composite metric enables the gain of stronger statistical power and the uncovering of deeper biological insights than the analysis of its individual components alone.

## Discussion

This study systematically investigated the associations, causal relationships, and molecular mechanisms linking lipid metabolism–related indicators (AIP, TG, HDL-C, LDL-C) with gout pathogenesis by integrating observational analyses of the NHANES cohort, mediation modeling, MR validation, and network pharmacology–based mechanistic exploration. The findings provide multi-dimensional evidence to inform gout risk prediction and potential clinical interventions.

Using data from 6,985 NHANES participants (719 [10.29%] with confirmed gout and 6,266 [89.71%] without gout; mean age 62.07 ± 13 years), we first validated the associations of several established gout risk factors. Individuals with gout were more likely to be male (67.7% vs 44.5%), NHW (47.3% vs 43.2%), obese (BMI 33.08 vs 31.51 kg/m^2^), and have abdominal obesity (WC 112.43 vs 106.38 cm), smoking habits (57.6% vs 48.7%), alcohol consumption (78.9% vs 71.9%), and hypertension in the absence of diabetes. All associations were statistically significant (*p* < 0.001), consistent with prior epidemiological studies, underscoring the central role of lifestyle and metabolic phenotypes in gout development.

Notably, individuals with higher levels of TG, HDL-C, LDL-C, and AIP exhibited more severe gout symptoms. For the first time, this study established a nonlinear dose–response relationship between AIP and gout (RCS analysis: P-overall < 0.001, P-non-linear = 0.027). After adjusting for multiple covariates, participants in the highest AIP quartile had a 70% higher risk of gout compared to those in the lowest quartile (OR = 1.700, 95% CI: 1.146–2.521, *p* = 0.010). As a composite indicator of TG and HDL-C, elevated AIP reflects the overall state of lipid metabolism dysfunction. This nonlinear association suggests a potential threshold effect, but the specific value (0.07 mmol/L) is derived from our observational data and requires validation in prospective studies before any clinical application. Cross-sectional analysis further demonstrated a protective effect of HDL-C (highest quartile: OR = 0.623, *p* = 0.042), whereas elevated TG significantly increased gout risk (highest quartile: OR = 1.666, *p* = 0.008). These findings align with the pathophysiological mechanism by which lipid metabolism disorders impair uric acid excretion and promote urate crystal deposition.

Subgroup analysis revealed notable population heterogeneity in the association between AIP and gout. The risk effect of elevated AIP was more pronounced in NHB and NHW individuals (OR = 2.025 and 2.897, respectively; both *p* < 0.001), alcohol consumers (OR = 2.637, *p* < 0.001), and patients with hypertension or diabetes (OR = 4.591 and 5.747, respectively; *p* < 0.05). These findings underscore the need for personalized gout prevention strategies that consider lipid metabolism profiles across different populations. For instance, enhanced monitoring of AIP and related lipid indicators is particularly warranted in individuals with comorbid metabolic disorders or unhealthy lifestyle habits. In contrast, PA and caffeine intake were not significantly associated with gout, providing additional insight into the precise identification of relevant risk factors.

The association between AIP and gout has been consistently reported in cross-sectional studies [27], yet the causal contributions of its core components, TG and HDL-C, remain unclear [28, 29]. Mediation analysis offered critical insights into the pathways linking AIP to gout: HDL-C (mediation proportion: 45.19%), BMI (9.98%), and WC (29.95%) each partially mediated the AIP–gout association, seemingly supporting the classical view that a deficiency of “good cholesterol” contributes to gout-related inflammation [30]. However, the mediation analysis is based on cross‑sectional data, and the indirect effect was modest (45.19%). The 95% confidence interval for the indirect effect was wide, and formal mediation tests in prospective designs are needed to validate this finding. Therefore, this result should be considered preliminary and hypothesis‑generating rather than conclusive.

Notably, despite being a core component of AIP, TG did not exhibit a significant mediation effect (mediation proportion: −3.43%). Instead, TG exerted a strong independent effect on gout pathogenesis through a highly significant direct effect (ADE = 0.1013, *p* < 2 × 10^−16^). This finding challenges the assumption that “AIP influences gout indirectly via TG” and, for the first time, provides evidence of a direct pathogenic role of TG in gout that is independent of the composite AIP metric.

Univariate MR effectively mitigates confounding and reverse causation biases inherent in observational studies by leveraging genetic IVs. Our univariable MR analysis suggested a positive association between TG and gout (OR = 1.0058 …), and a protective association for HDL-C (OR = 0.9967 …). Reverse MR indicated a possible bidirectional relationship (OR = 51.07, *p* = 0.0337), but this estimate was highly heterogeneous (I^2^ = 99.4%) and imprecise (wide CI); therefore, no robust evidence for reverse causality was found. The TG-specific immune activation phenotype (Group B) identified in our network analysis was enriched in T-cell and NF-κB pathways, which may suggest a potential link, but this remains speculative. After adjusting for potential confounders, multivariable MR analysis suggested that TG may have an independent effect on gout (OR = 1.0077, *p* < 0.001), but given the extreme heterogeneity, these results should be interpreted with caution and do not establish causality. In contrast, although HDL-C showed a protective effect in univariate MR, it exhibited heterogeneity and lost statistical significance in multivariable MR analyses. Consistent findings across weighted median, MR-Egger, and MR-PRESSO sensitivity analyses suggest that the apparent protective association of HDL-C in observational studies likely arises from residual confounding (e.g., associations with coronary artery disease or myocardial infarction) or reverse causation, rather than a direct causal effect [31, 32]. Although heterogeneity was detected in MR analyses (all methods *p* < 0.001), the non-significant MR-Egger intercept (*p* = 0.123) indicates minimal horizontal pleiotropy, supporting the reliability of the results. Taken together, this progressive analytical framework, from observational association to mediation pathway dissection to causal validation, provides a comprehensive chain of evidence highlighting the central role of TG in gout pathogenesis, bridging statistical observation with biological essence.

To reconcile discrepancies between observational and causal findings and to further elucidate the shared and specific molecular mechanisms linking TG, HDL-C, AIP, and gout, we constructed a quadruple cross-target network using network pharmacology. This analysis identified seven core gene sets, which were subsequently subjected to functional enrichment and PPI analyses. The results revealed three distinct functional subtypes:

(I) Shared Inflammatory-Metabolic Phenotype (Groups G and F)

Targets shared by TG, HDL-C, and gout (or across all four conditions) were significantly enriched in pathways such as Lipid and atherosclerosis (hsa05417), AGE-RAGE signaling pathway in diabetic complications (hsa04933), and IL-17 signaling pathway (hsa04657) [1, 33]. Core hub genes, including IL6, TNF, and IL1B [34], formed a classical inflammatory axis, reinforcing the characterization of gout as a “metabolic inflammatory disease.” Elevated TG can activate macrophages to release pro-inflammatory cytokines, thereby amplifying urate crystal–induced inflammation. Moreover, IL6 and TNF play dual roles in regulating both lipid metabolism disorders and gout-related inflammation, serving as pivotal nodes that link these two processes.

(II) TG-Specific Immune Activation Phenotype (Group B)

Targets uniquely shared by TG and gout were significantly enriched in the T cell receptor signaling pathway (hsa04660) and NF-κB signaling pathway (hsa04064) [35, 36]. Hub genes in the PPI network, including PTPRC (CD45), MYD88, and LCK, serve as key nodes linking innate and adaptive immunity [37]. These findings extend the traditional view of gout as predominantly an innate immune–driven disease, suggesting that TG dysregulation may modulate adaptive immune responses via activation of T cell and NF-κB signaling pathways. This highlights a mechanistic “lipid–immune” axis and provides new directions for the development of immune-targeted therapeutic strategies.

(III) HDL-C-Specific Structural-Transport Phenotype (Group C)

In contrast, targets uniquely shared by HDL-C and gout were primarily involved in fundamental cellular processes, including cytoskeleton organization (GO:0007010) and endocytic transport (GO:0006897) [38]. Hub genes such as CBL and RASA1 are linked to signal attenuation and endocytosis rather than core inflammatory pathways [39]. This mechanistic profile helps explain why HDL-C did not exhibit a direct protective effect in causal analyses; the apparent protective signal observed in observational studies likely reflects its “buffering” role within metabolic networks or its function as a reverse biomarker rather than a true causal effect.

(IV) Synthesis: Hierarchical Pathogenic Model and the Core Driving Role of TG

Notably, our pQTL-based Mendelian randomization identified largely non-overlapping gene sets for AIP- and TG/HDL-C-associated gout risk (sharing only *AKR1C3*), providing genetic-level evidence that AIP is not merely a derivative of its components but may capture a distinct pathogenic axis. This divergence was further reflected in their functional profiles: the AIP-related network centered on immunoregulation and stress adaptation (e.g., STAT3, NFKB1 hubs), while the TG/HDL-C network was anchored in cell growth and metabolic homeostasis (e.g., ERBB2, PIK3CA hubs). There was partial overlap between the TG‑associated and HDL‑C‑associated protein networks (Jaccard index 0.18), suggesting both shared and distinct biological pathways.

Mediation analysis (cross‑sectional) only decomposes statistical associations and does not imply causality. MR was limited by extreme heterogeneity. The apparent HDL‑C mediation likely reflects confounding or reverse causation, consistent with MR showing no independent effect of HDL‑C. TG showed a strong direct effect in mediation and a positive (though not robust) MR signal, suggesting TG may be a relevant target, but this remains tentative.

Collectively, these findings support a hierarchical pathogenic model. At the clinical-phenotypic level, AIP functions as an effective “dashboard” for metabolic inflammation [40], integrating signals from lipid metabolism disorders and inflammatory states. At the genetic-causal level, TG may independently elevate gout risk, potentially through activation of T cell and NF-κB signaling pathways, and may serve as a potential driver of a lipid–immune interaction that requires further investigation. In contrast, HDL-C primarily acts as a metabolic “buffer” or reverse biomarker, with its apparent protective effect in observational studies reflecting network correlations rather than a direct causal influence. This critical shift from significance to null effect after adjusting for TG and other lipids in multivariable MR strongly suggests that the observed protective association of HDL-C is largely confounded by or mediated through its inverse metabolic relationship with TG, rather than representing a direct causal pathway. This finding aligns with our network pharmacology results, where the unique HDL-C-gout gene set (Group C) was enriched in structural and transport functions rather than core inflammatory pathways, further indicating its role may be more correlative than causative.

This model suggests TG as a potential lipid target for gout prevention and management and offers a hypothetical framework for intervention strategies that could extend beyond conventional urate‑lowering approaches. It suggests that early control of hypertriglyceridemia, possibly through immunomodulatory interventions, might reduce gout risk, but this hypothesis requires testing in prospective studies [41]. These findings suggest that targeted modulation of TG‑associated immune‑inflammatory pathways could represent a potential therapeutic avenue, but this remains speculative and requires experimental validation.

(V) Study Strengths and Limitations

The core strength of this study lies in its multidisciplinary, integrated design, spanning “clinical observation → causal validation → mechanistic exploration.” Large-scale NHANES data, which uses a complex, multistage probability sampling design to represent the non‑institutionalized US civilian population, enhance generalizability to the US population but not necessarily to other ethnic or geographic groups; MR establishes causal relationships while minimizing biases inherent in traditional studies; and network pharmacology uncovers functional subtypes at the molecular level, enabling cross-scale resolution from macro-level phenotypes to micro-level molecular mechanisms. Moreover, the integration of subgroup analyses and mediation models elucidates population-specific risk differences and mechanistic pathways, offering precise targets for personalized intervention strategies.

This study has several limitations. First, MR analyses showed potential heterogeneity; although results were validated using multiple methods, larger genetic datasets are needed to strengthen causal inference [42]. Second, target prediction in network pharmacology relied on publicly available databases and lacks experimental validation; the functional roles of core genes (e.g., IL6, PTPRC, CBL) require confirmation through in vitro and in vivo studies [43]. Finally, the mediation analysis did not account for uric acid excretion, uric acid production, or other directly relevant metabolic indicators, which may have led to the omission of key mediation pathways. Additionally, although missing caffeine data were imputed using MICE, sensitivity analysis (missing indicator method) confirmed the robustness of our AIP effect estimate (Evalue = 2.09), making bias due to caffeine missingness unlikely.

Several additional limitations should be noted. First, gout diagnosis was based on self‑report in NHANES, which may introduce misclassification bias. Second, we could not adjust for important confounders such as dietary purine intake, renal function, or urate‑lowering medications. Third, residual confounding by unmeasured lifestyle factors cannot be ruled out. Fourth, the MR analyses suffered from extreme heterogeneity (I^2^ > 99%), which violates key assumptions and limits causal inference; therefore, the MR results are considered exploratory. Fifth, Steiger filtering indicated possible reverse causation for some genetic instruments, further questioning the direction of causality. Sixth, network pharmacology predictions are based on public databases and lack experimental validation; the proposed mechanisms are hypothetical. Seventh, the mediation analysis assumes no unmeasured confounding and temporal precedence, which may not hold in cross‑sectional data.

(VI) Clinical Implications and Future Directions

Importantly, our subgroup and mediation analyses indicate that AIP’s predictive utility is particularly salient in high-risk populations such as older adults, those with hypertension/diabetes, and alcohol drinkers, where the association was strongest. The observed nonlinear relationship may inform future interventional studies, but no clinical threshold can be recommended based on this cross-sectional analysis.

The core clinical value of this study lies in establishing TG as an independent causal risk factor for gout, highlighting TG-targeted interventions as a novel strategy for prevention. Subgroup analyses suggest that enhanced monitoring of TG levels is particularly warranted in high-risk populations, including older adults (≥65 years), individuals with comorbid hypertension or diabetes, and alcohol drinkers. Maintaining TG within a reasonable range (mean TG: 1.79 mmol/L in the gout group vs. 1.50 mmol/L in the non-gout group in this study) may help mitigate gout risk. Additionally, as a convenient and integrative indicator of lipid metabolism, the nonlinear risk threshold of AIP can serve as a reference for clinical risk stratification and early intervention.

Future research may proceed along three directions. First, interventional studies are needed to assess whether reducing TG levels, through dietary modification, pharmacological treatment, or modulation of TG-related immune–inflammatory pathways, can lower gout incidence and recurrence. Second, experimental validation of core genes identified via network pharmacology (e.g., IL6, PTPRC, MYD88) is necessary to clarify their functional roles in TG-mediated gout pathogenesis. Third, larger genetic studies should be conducted to further investigate the mechanisms underlying the apparent protective effect of HDL-C and to elucidate its role as a reverse biomarker in lipid–gout interactions.

## Conclusion

Through a multi-dimensional analysis, this investigation supports a nonlinear association between AIP and gout, suggests that the apparent protective effect of HDL-C may arise from residual confounding rather than direct causation, and indicates that TG may exert a direct effect on gout, potentially involving immune‑related pathways, though this requires further validation. At the molecular level, TG, HDL-C, and AIP may influence gout pathogenesis via multiple pathways, including inflammatory metabolism, immune regulation, and cellular structural transport, with core inflammatory mediators and immune-related genes serving as key nodes. These findings provide a preliminary theoretical basis and may inform future research on gout risk assessment, stratified intervention, and therapeutic target development, highlighting a potential role of lipid metabolism disorders, particularly elevated TG, in gout prevention and management, and suggesting that strategies beyond traditional urate‑lowering interventions warrant further investigation.

## Electronic supplementary material

Below is the link to the electronic supplementary material.

Supplementary Material 1

Supplementary Material 2

Supplementary Material 3

Supplementary Material 4

Supplementary Material 5

Supplementary Material 6

Supplementary Material 7

Supplementary Material 8

Supplementary Material 9

Supplementary Material 10

Supplementary Material 11

## Data Availability

All data analyzed are publicly available. NHANES 2009–2018 data: https://wwwn.cdc.gov/nchs/nhanes. Lipid trait GWAS data (HDL-C: ieu-b-109; LDL-C: ieu-b-110; TG: ieu-b-111): https://gwas.mrcieu.ac.uk/. Gout GWAS data: EBI (ebi-a-GCST90038687), FinnGen (https://www.finngen.fi/en), UK Biobank (https://www.ukbiobank.ac.uk/). Icelandic pQTL data and network pharmacology gene sets (GeneCards: https://www.genecards.org/; OMIM: https://omim.org/) are publicly accessible. No new datasets were generated. Replication-related data are available from the corresponding author on reasonable request.

## Abbreviations

AIP: Atherogenic Index of Plasma
TG: Triglycerides
HDL-C: High-Density Lipoprotein Cholesterol
LDL-C: Low-Density Lipoprotein Cholesterol
MR: Mendelian Randomization
MVMR: Multivariable Mendelian Randomization
IVW: Inverse Variance Weighted
pQTL: Protein Quantitative Trait Loci
GWAS: Genome-Wide Association Study
NHANES: National Health and Nutrition Examination Survey
BMI: Body Mass Index
WC: Waist Circumference
PA: Physical Activity
PPI: Protein-Protein Interaction
KEGG: Kyoto Encyclopedia of Genes and Genomes
GO: Gene Ontology
DO: Disease Ontology
FDR: False Discovery Rate
OR: Odds Ratio
CI: Confidence Interval
MICE: Multiple Imputation by Chained Equations
RCS: Restricted Cubic Splines
MRER: MR-Egger Regression
WMM: Weighted Median Method
LD: Linkage Disequilibrium
SNPs: Single Nucleotide Polymorphisms
AIC: Akaike Information Criterion
MLR: Multivariable Logistic Regression
NHW: Non-Hispanic White
NHB: Non-Hispanic Black
ADE: Average Direct Effect
ACME: Average Causal Mediation Effect
