# Supplementary figures and images for: Decoding the lipid etiology of atherogenic index of plasma and gout: establishing the causal role of triglycerides through NHANES, Mendelian randomization, and network pharmacology
Source: Cardiovasc Diabetol Endocrinol Rep. 2026 Jul 13;12:40. doi: 10.1186/s40842-026-00309-0 (PMC13362044; doi:10.1186/s40842-026-00309-0)

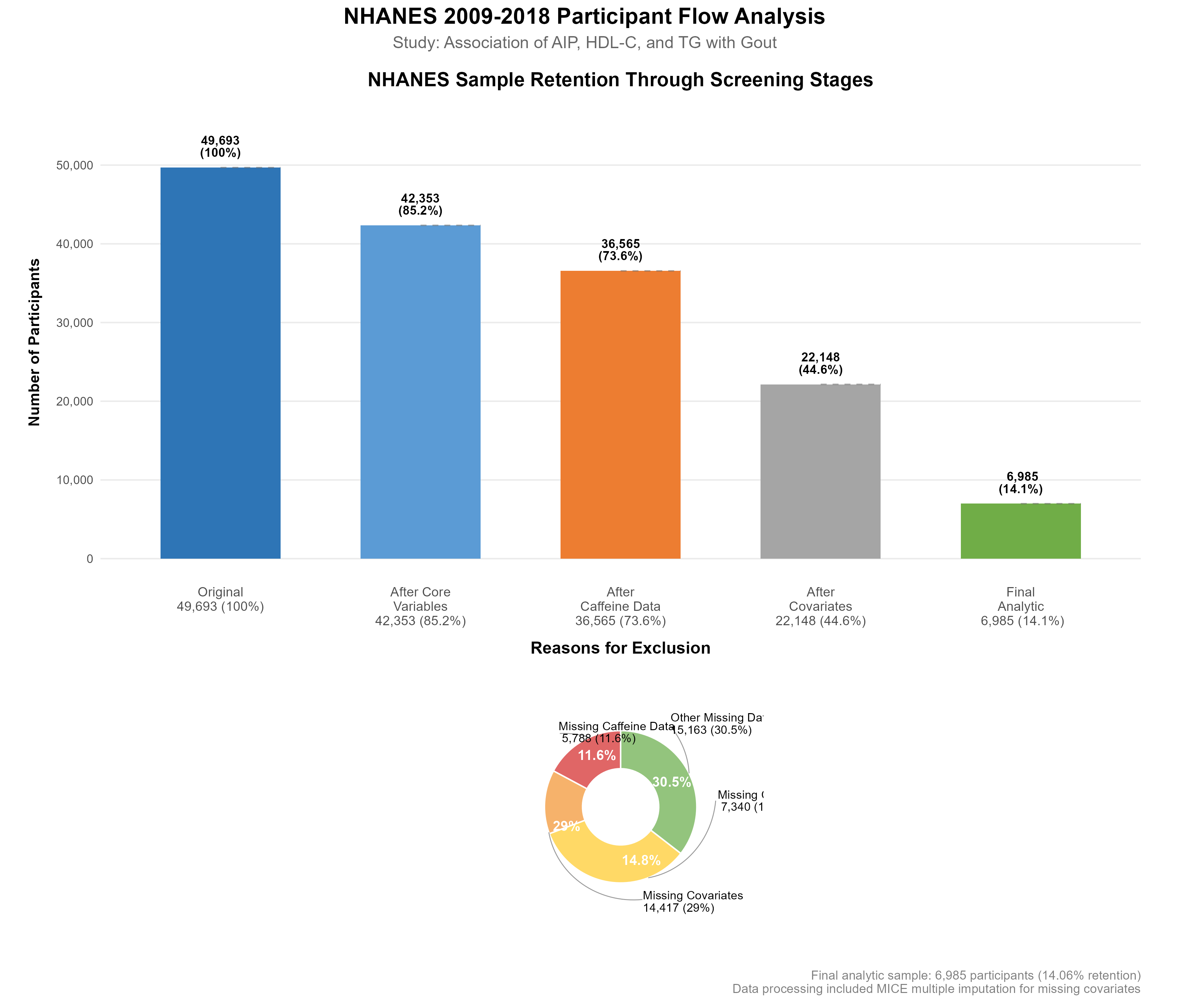

Supplement: Supplementary file 1 — Supplementary Material 1 [file 40842_2026_309_MOESM1_ESM.zip › 40842_2026_309_MOESM1_ESM.png]

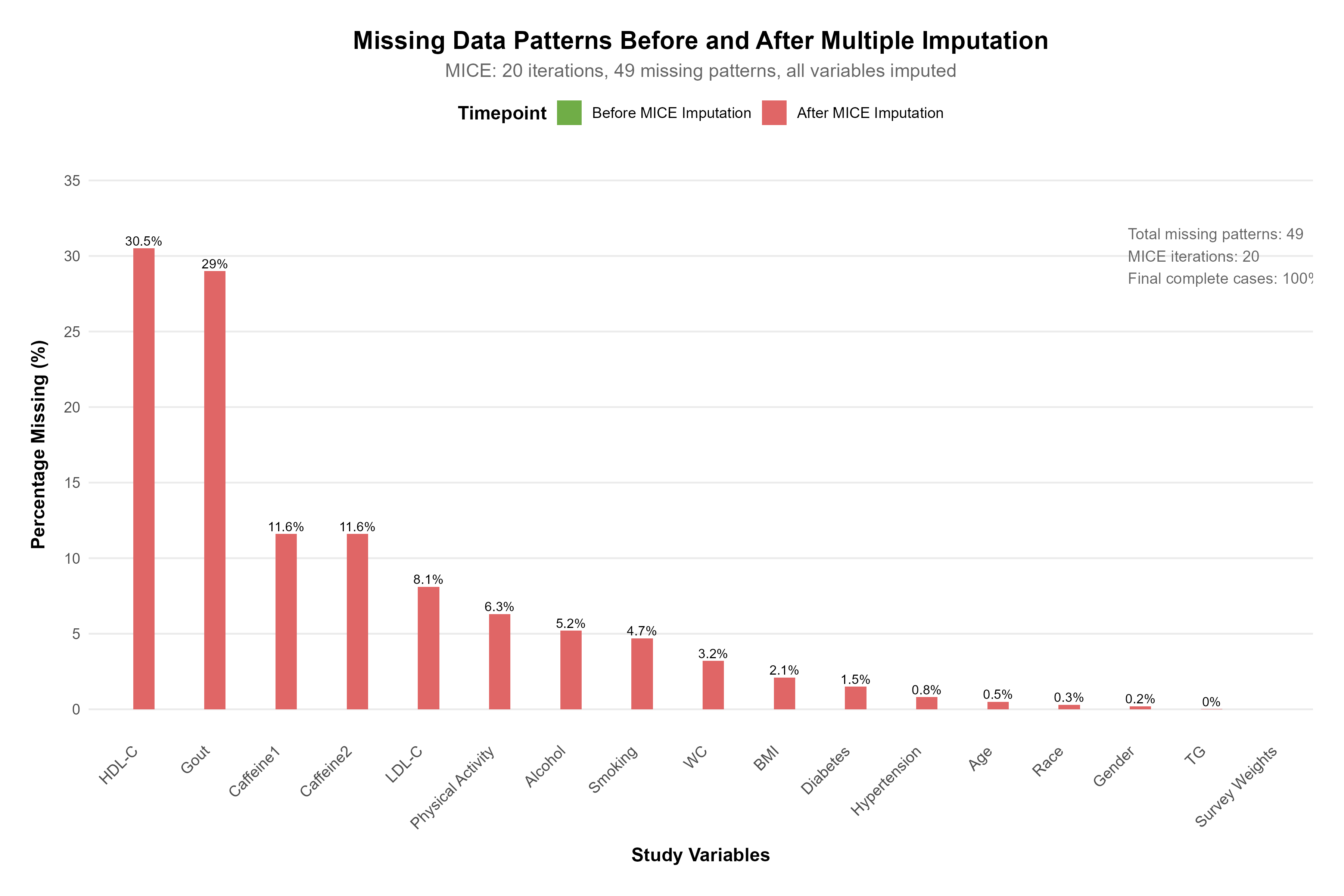

Supplement: Supplementary file 2 — Supplementary Material 2 [file 40842_2026_309_MOESM2_ESM.zip › 40842_2026_309_MOESM2_ESM.png]

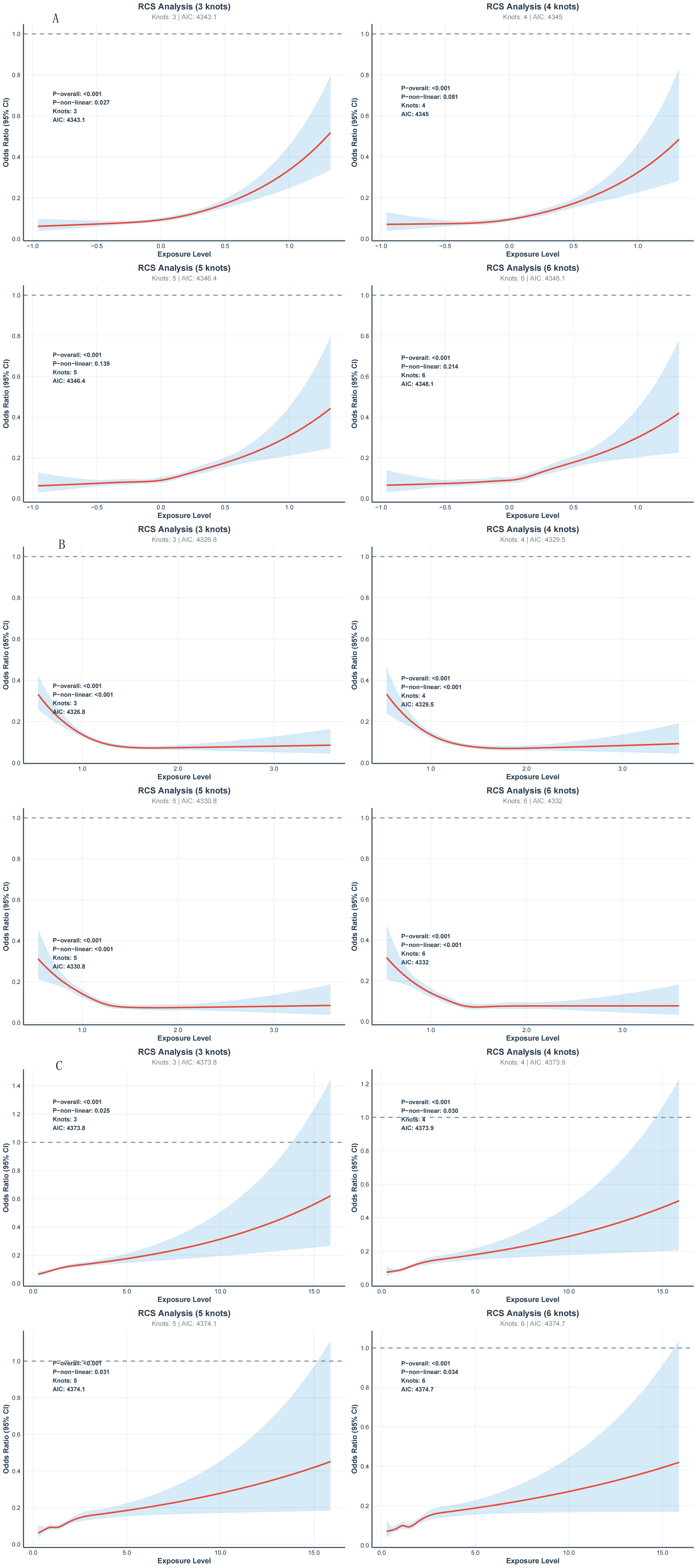

Supplement: Supplementary file 3 — Supplementary Material 3 [file 40842_2026_309_MOESM3_ESM.zip › 40842_2026_309_MOESM3_ESM.png]
