# Supplementary material for: Decoding the lipid etiology of atherogenic index of plasma and gout: establishing the causal role of triglycerides through NHANES, Mendelian randomization, and network pharmacology
Source: Cardiovasc Diabetol Endocrinol Rep. 2026 Jul 13;12:40. doi: 10.1186/s40842-026-00309-0 (PMC13362044; doi:10.1186/s40842-026-00309-0)
Supplement: Supplementary file 4 — Supplementary Material 4 [file 40842_2026_309_MOESM4_ESM.pdf]

FINNGEN
GOUT_109

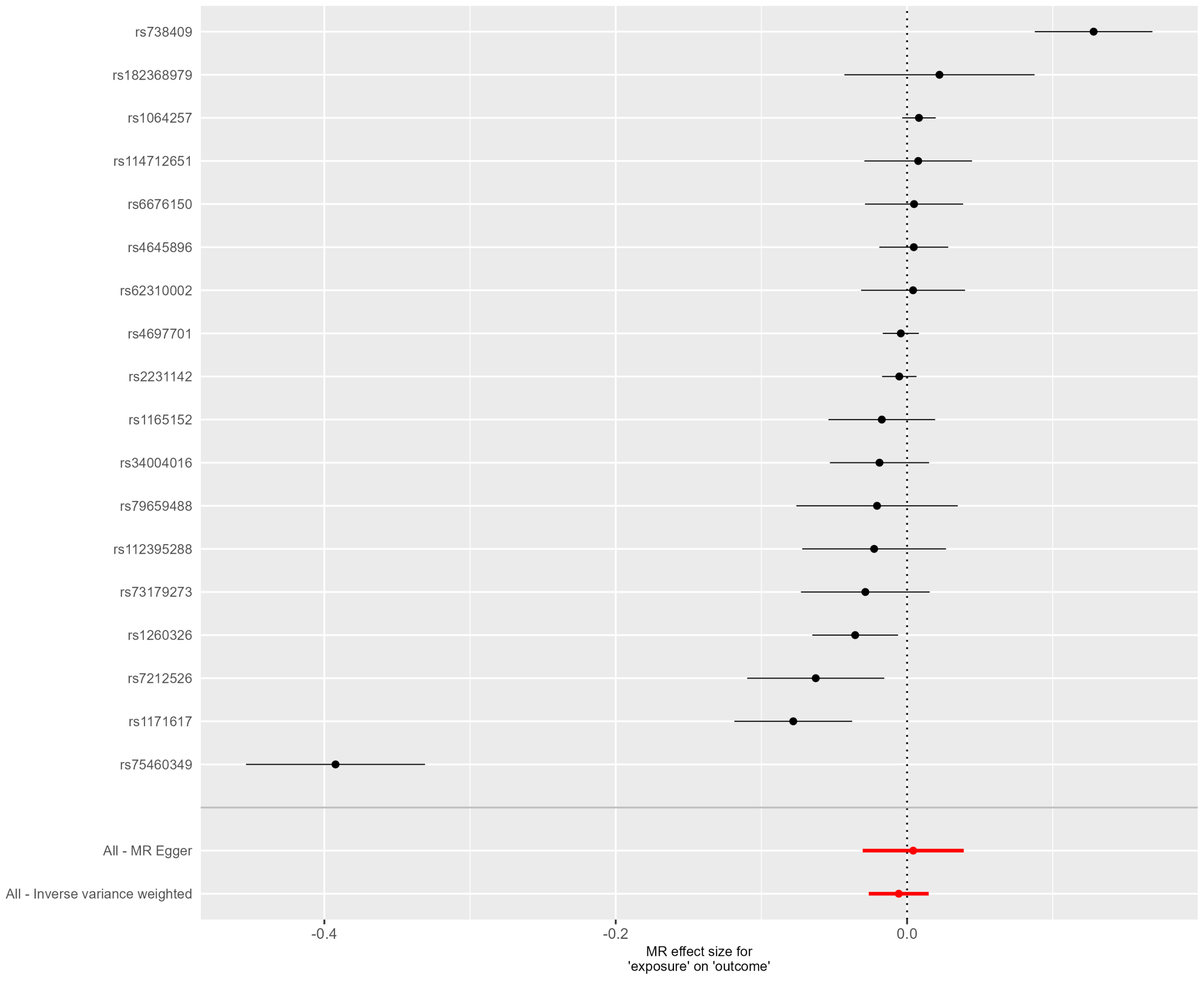

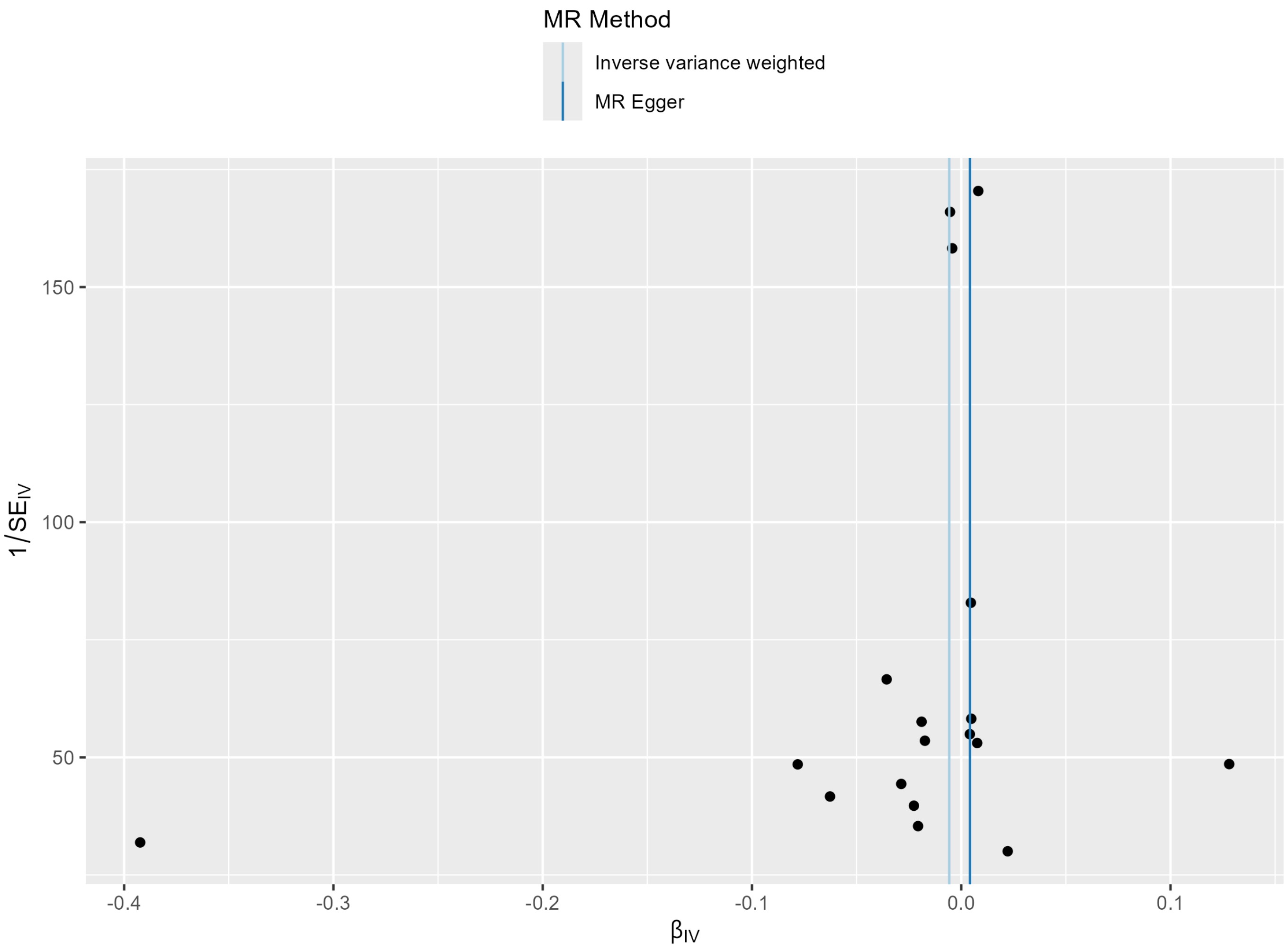
MR Method
Inverse variance weighted
MR Egger
150
100
50
1/SE_IV
-0.4
-0.3
-0.2
-0.1
0.0
0.1
β_IV

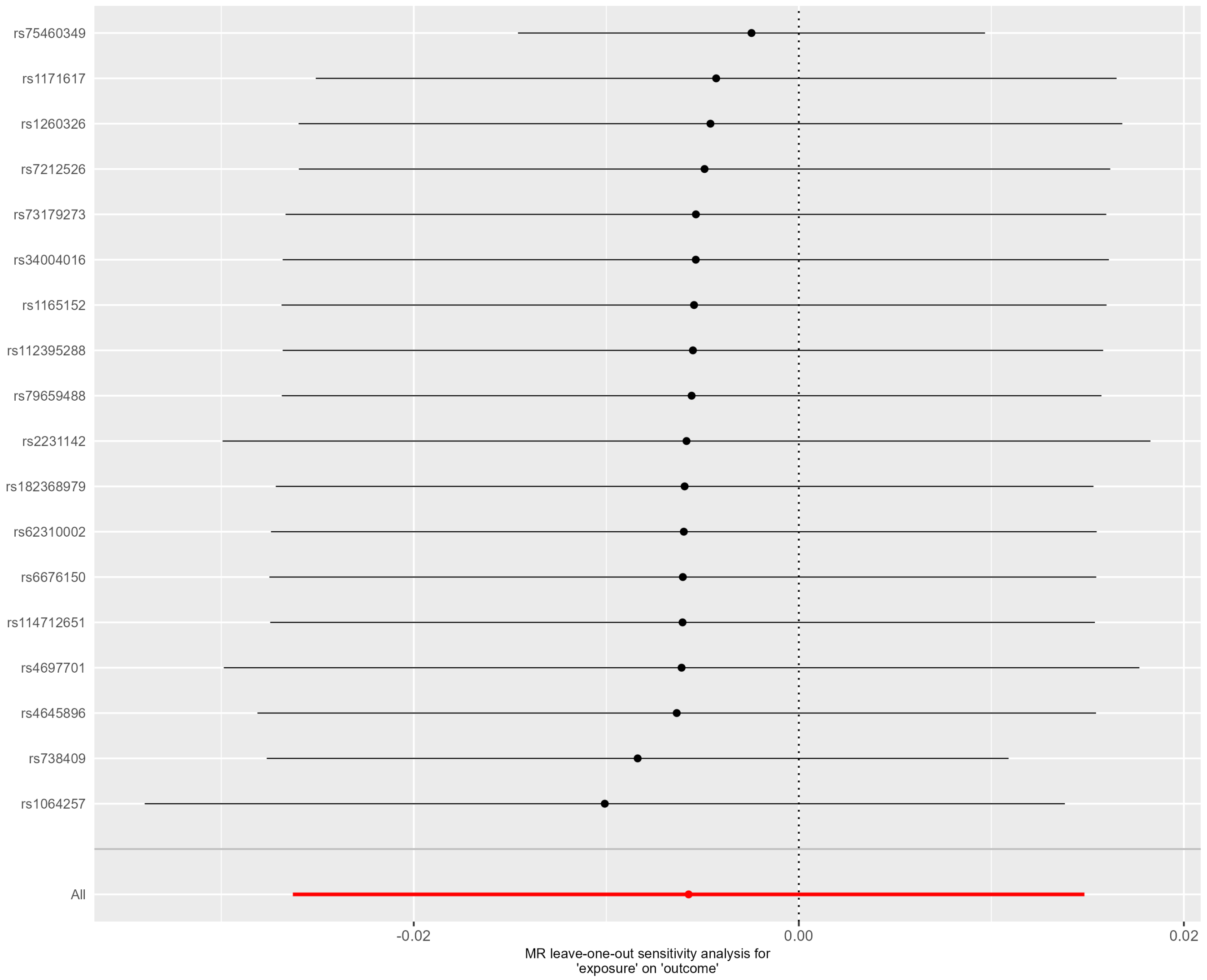
rs75460349
rs1171617
rs1260326
rs7212526
rs73179273
rs34004016
rs1165152
rs112395288
rs79659488
rs2231142
rs182368979
rs62310002
rs6676150
rs114712651
rs4697701
rs4645896
rs738409
rs1064257
All
-0.02
0.00
0.02
MR leave-one-out sensitivity analysis for
'exposure' on 'outcome'

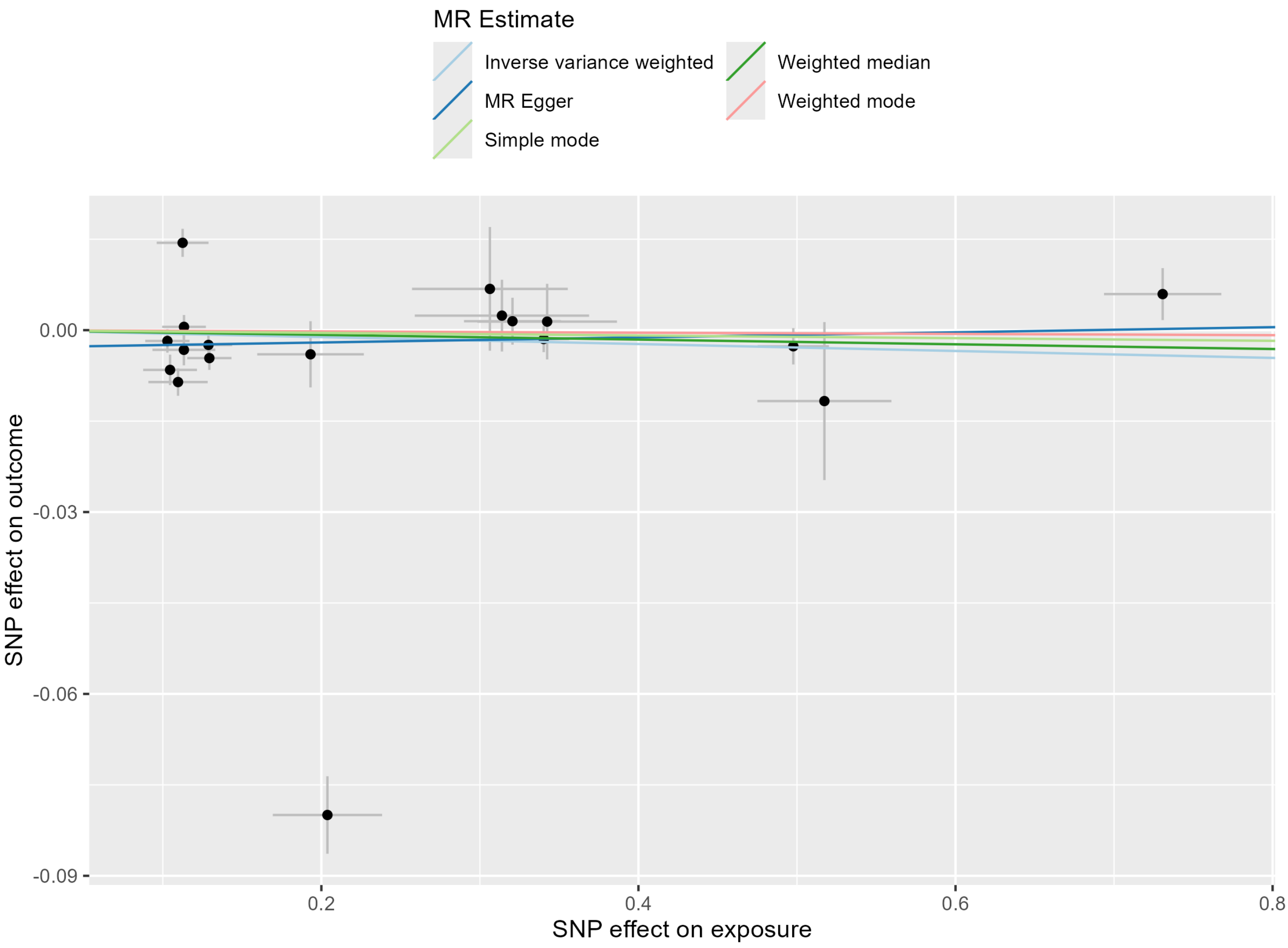
MR Estimate
Inverse variance weighted
MR Egger
Simple mode
Weighted median
Weighted mode
SNP effect on outcome
0.00
-0.03
-0.06
-0.09
0.2
0.4
0.6
0.8
SNP effect on exposure

GOUT_110

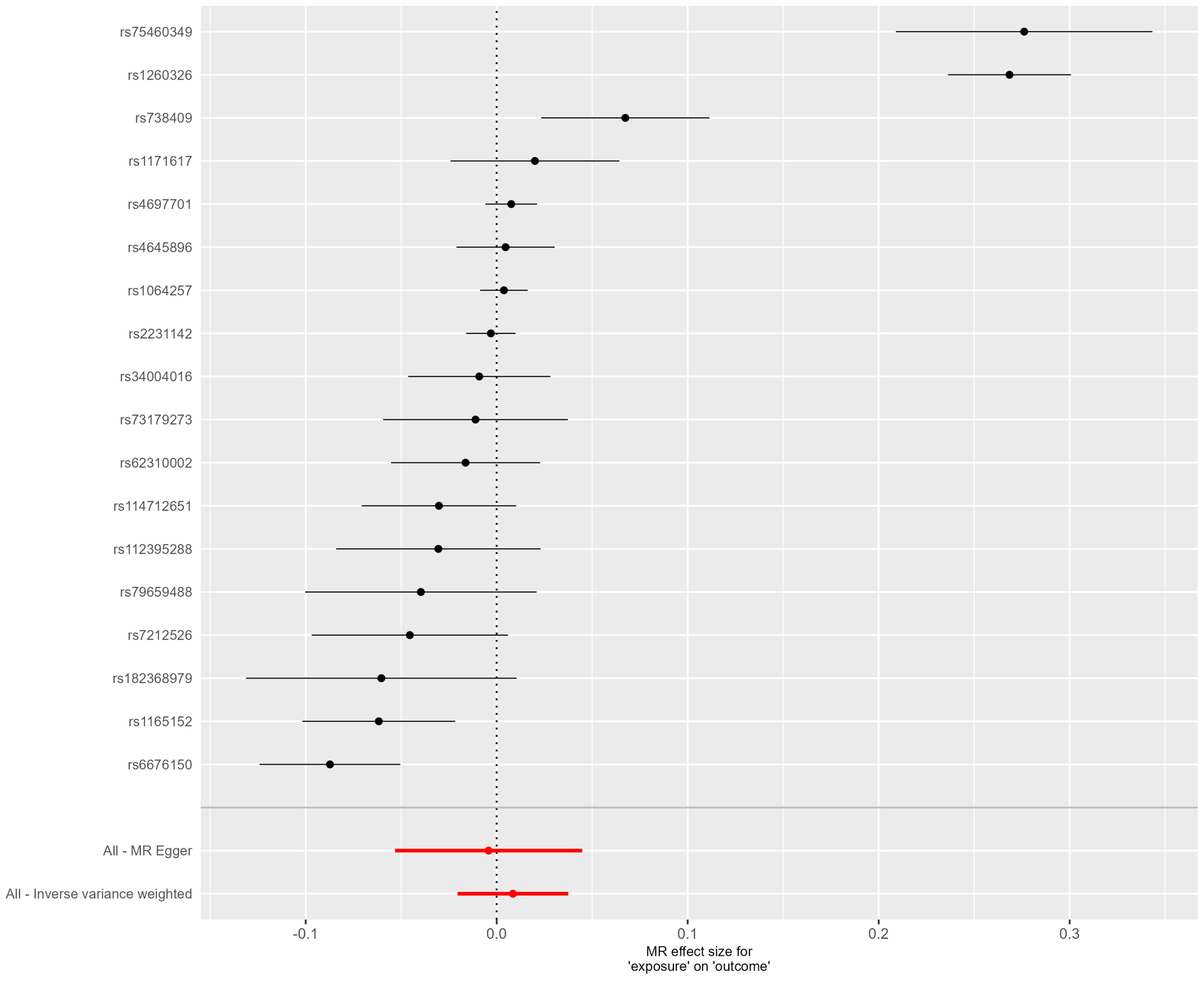

rs75460349
rs1260326
rs738409
rs1171617
rs4697701
rs4645896
rs1064257
rs2231142
rs34004016
rs73179273
rs62310002
rs114712651
rs112395288
rs79659488
rs7212526
rs182368979
rs1165152
rs6676150
All - MR Egger
All - Inverse variance weighted
-0.1
0.0
0.1
0.2
0.3
MR effect size for
'exposure' on 'outcome'

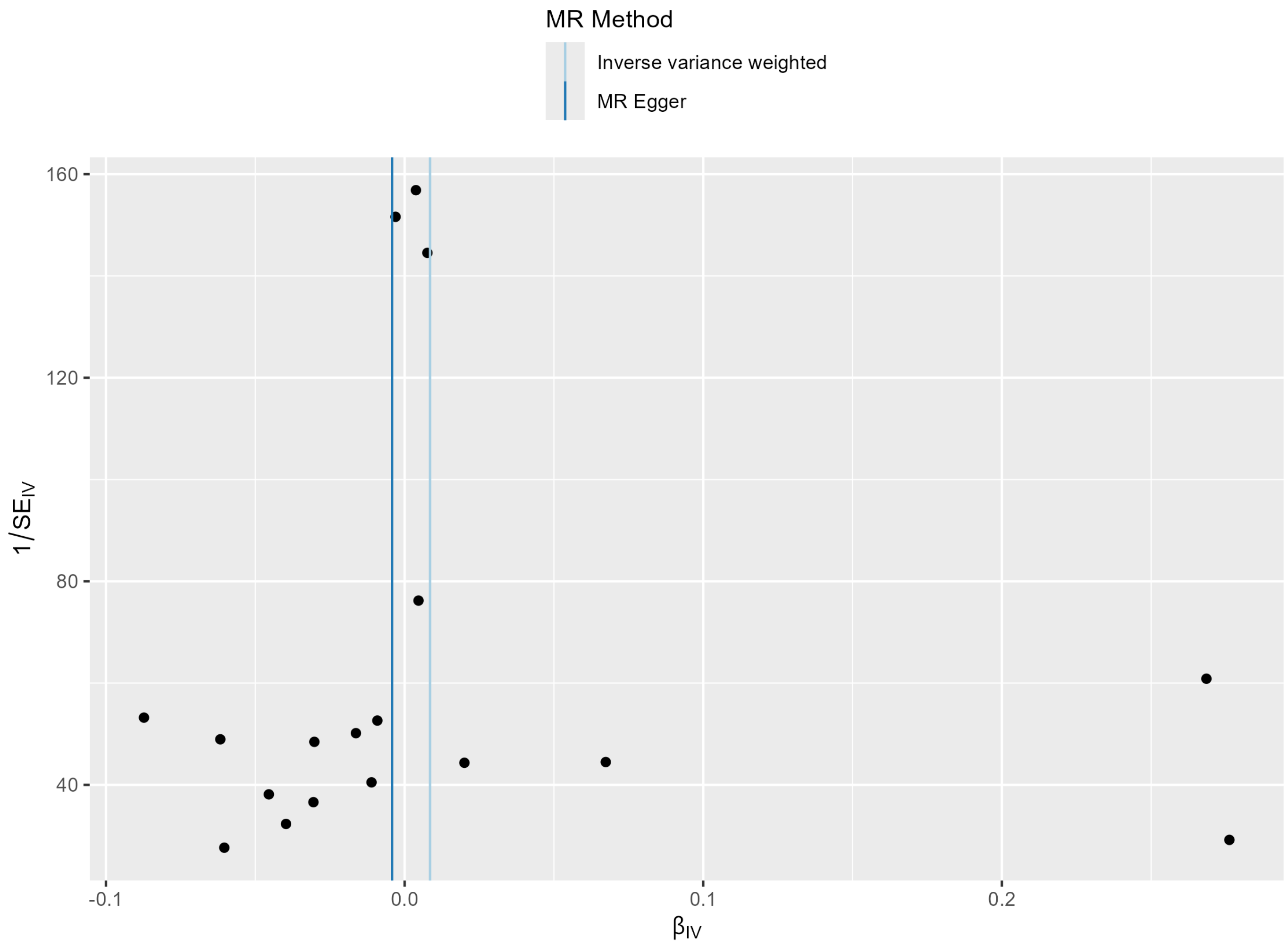
MR Method
Inverse variance weighted
MR Egger
160
120
80
40
1/SE_IV
-0.1
0.0
0.1
0.2
β_IV

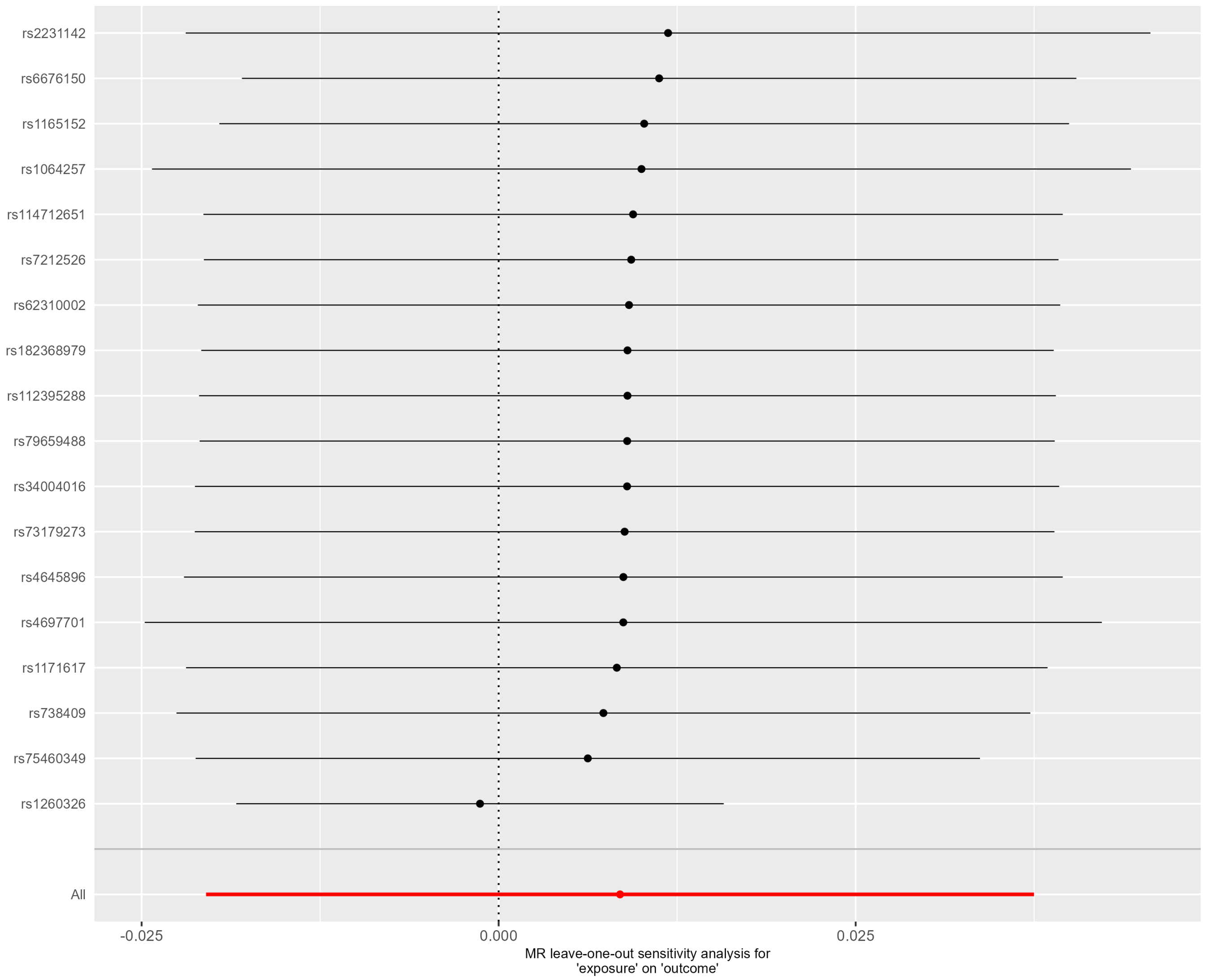
rs2231142
rs6676150
rs1165152
rs1064257
rs114712651
rs7212526
rs62310002
rs182368979
rs112395288
rs79659488
rs34004016
rs73179273
rs4645896
rs4697701
rs1171617
rs738409
rs75460349
rs1260326
All
-0.025
0.000
0.025
MR leave-one-out sensitivity analysis for
'exposure' on 'outcome'

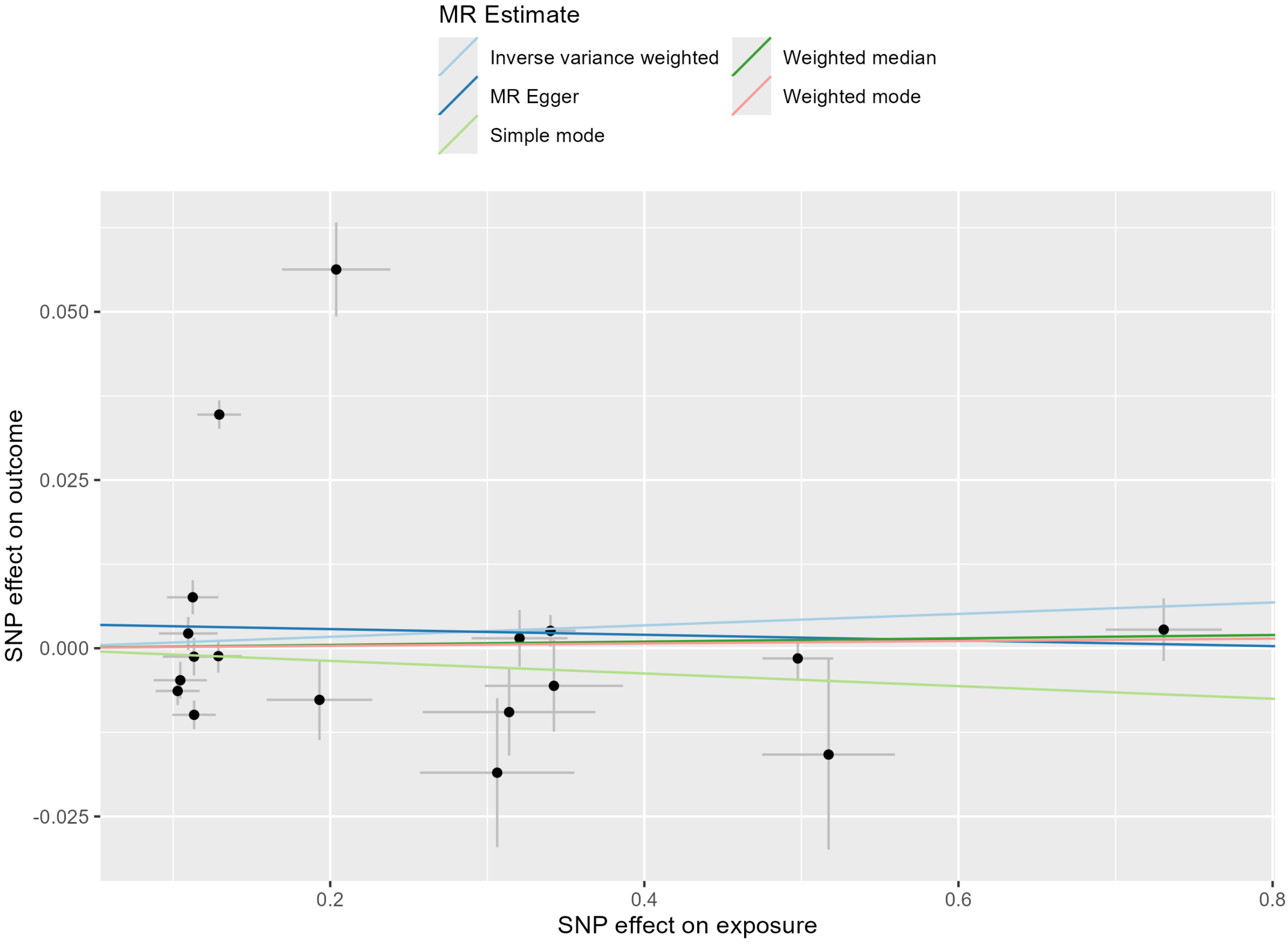

MR Estimate
Inverse variance weighted
MR Egger
Simple mode
Weighted median
Weighted mode
SNP effect on outcome
0.050
0.025
0.000
-0.025
0.2
0.4
0.6
0.8
SNP effect on exposure

GOUT_111

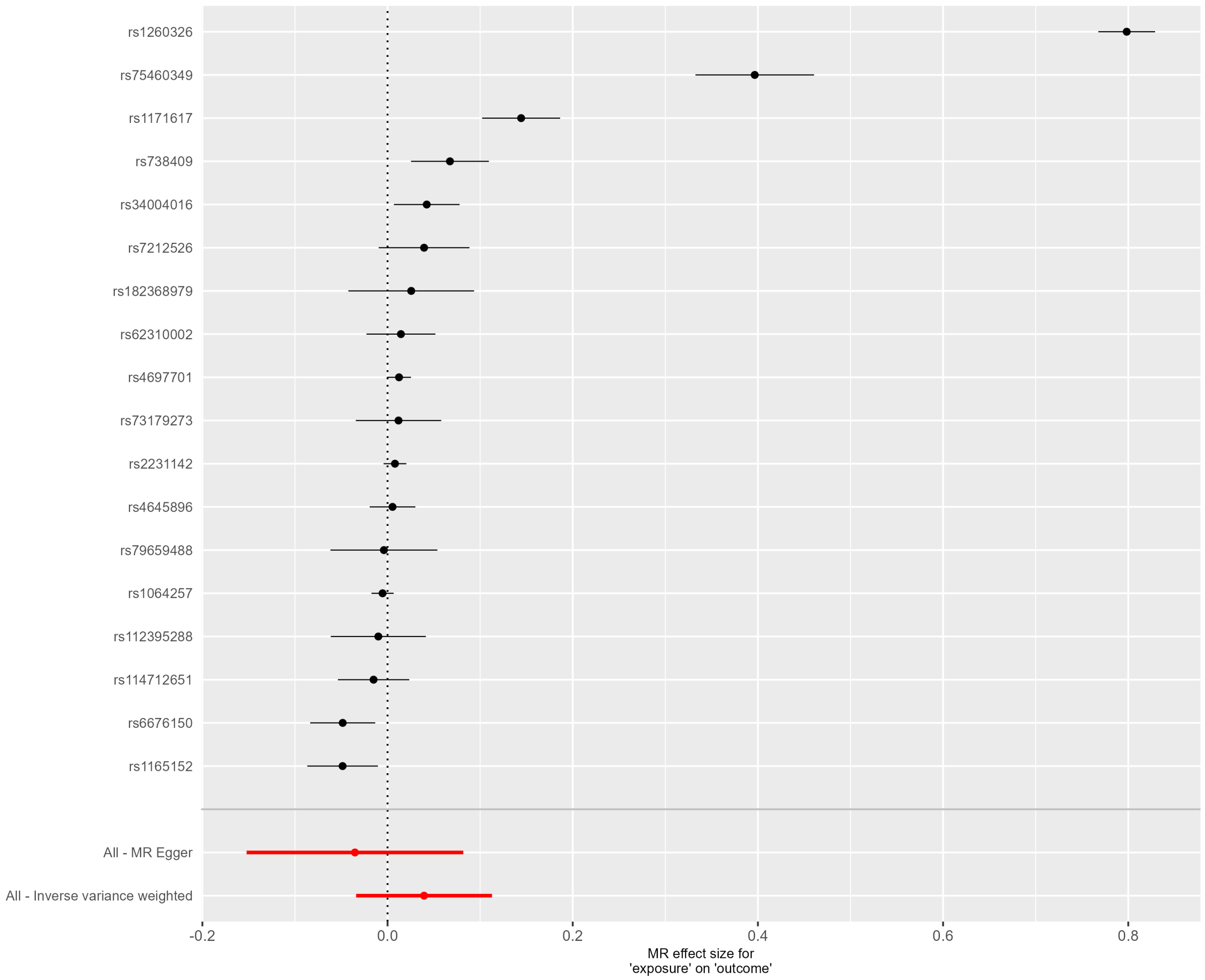
rs1260326
rs75460349
rs1171617
rs738409
rs34004016
rs7212526
rs182368979
rs62310002
rs4697701
rs73179273
rs2231142
rs4645896
rs79659488
rs1064257
rs112395288
rs114712651
rs6676150
rs1165152
All - MR Egger
All - Inverse variance weighted
-0.2
0.0
0.2
0.4
0.6
0.8
MR effect size for
'exposure' on 'outcome'

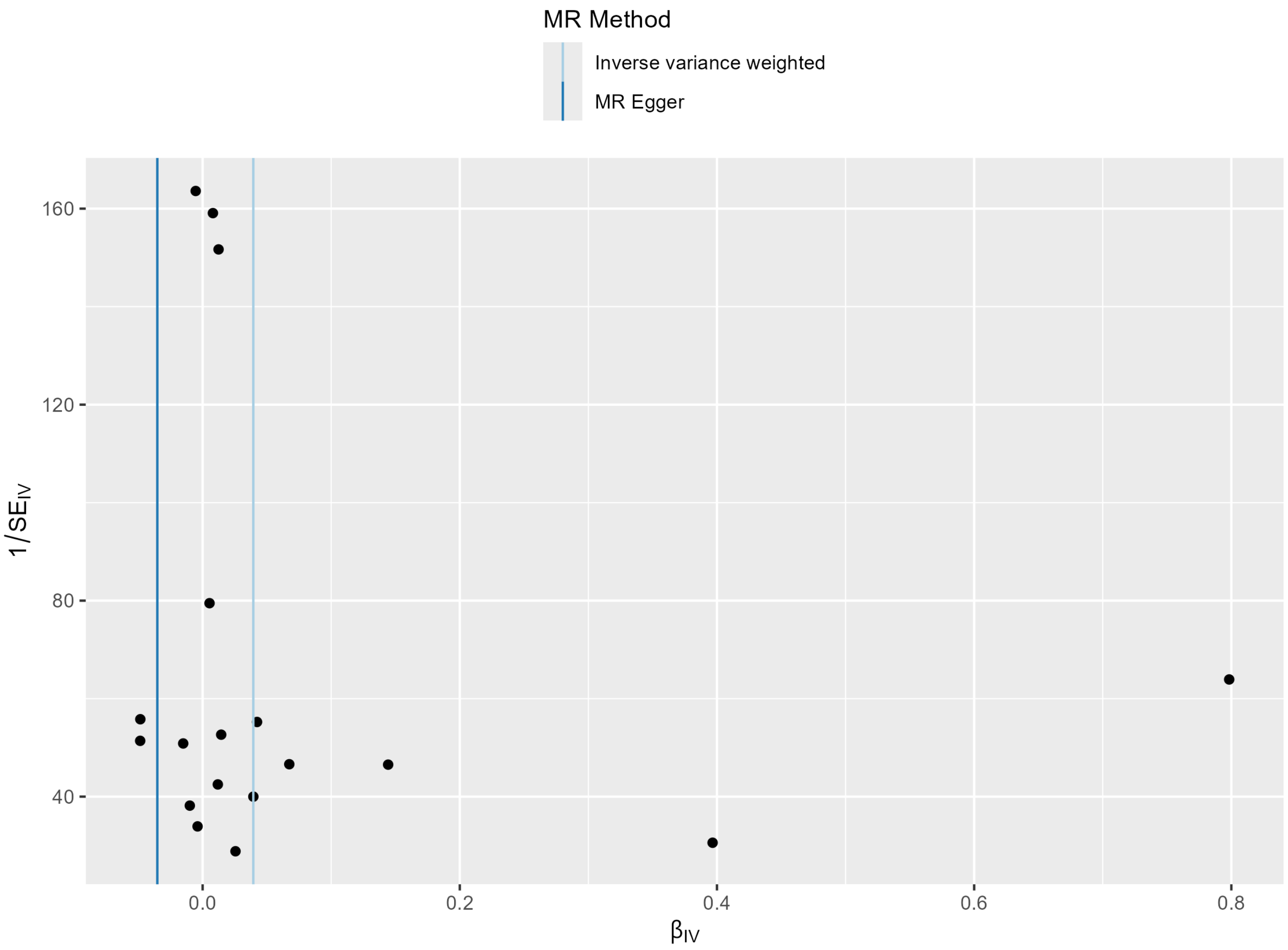

MR Method
Inverse variance weighted
MR Egger
1/SE_IV
160
120
80
40
0.0
0.2
0.4
0.6
0.8
β_IV

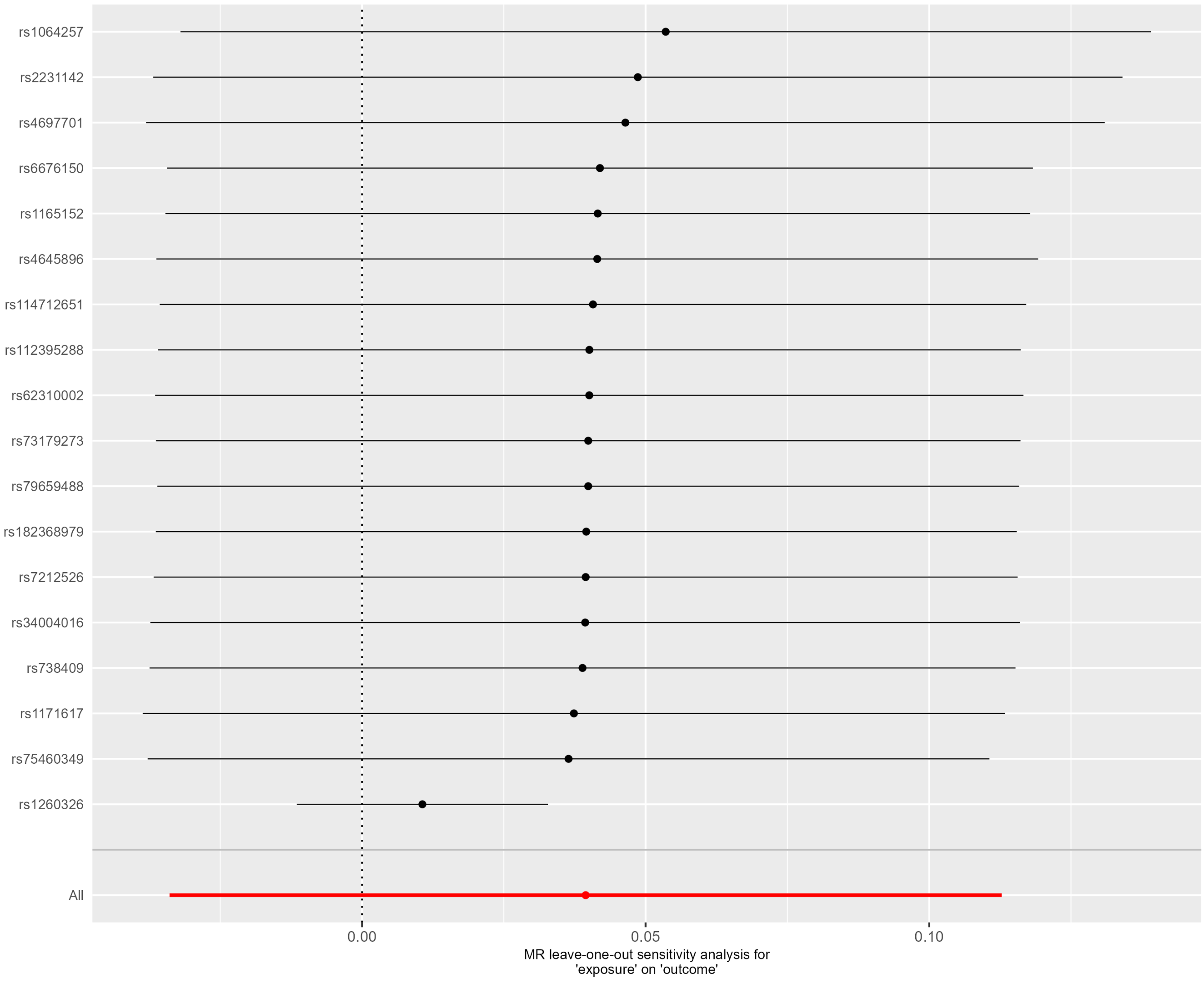

rs1064257
rs2231142
rs4697701
rs6676150
rs1165152
rs4645896
rs114712651
rs112395288
rs62310002
rs73179273
rs79659488
rs182368979
rs7212526
rs34004016
rs738409
rs1171617
rs75460349
rs1260326
All
0.00
0.05
0.10
MR leave-one-out sensitivity analysis for
'exposure' on 'outcome'

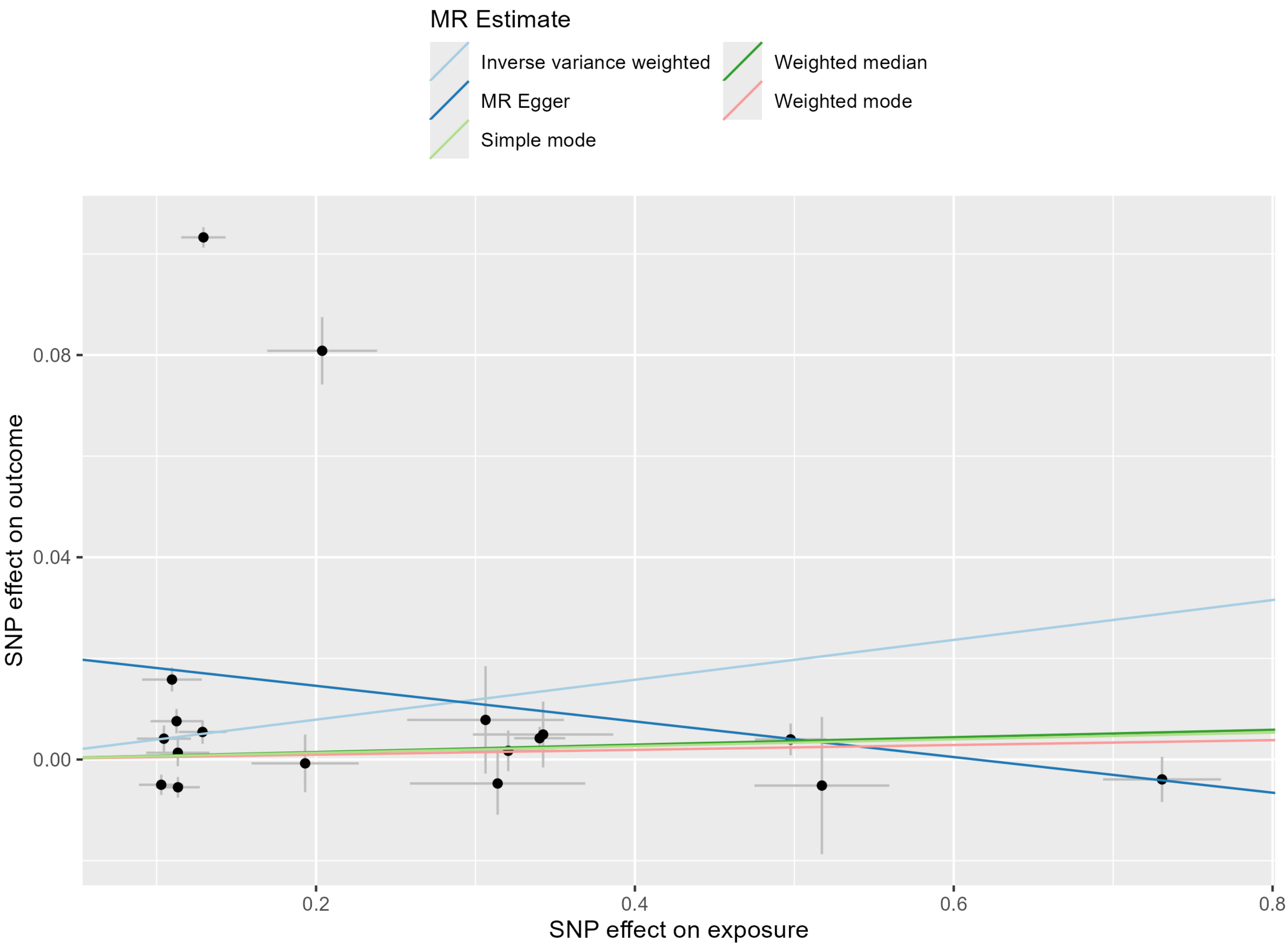
MR Estimate
Inverse variance weighted
MR Egger
Simple mode
Weighted median
Weighted mode
0.08
0.04
0.00
SNP effect on outcome
0.2
0.4
0.6
0.8
SNP effect on exposure

低密度蛋白_MR

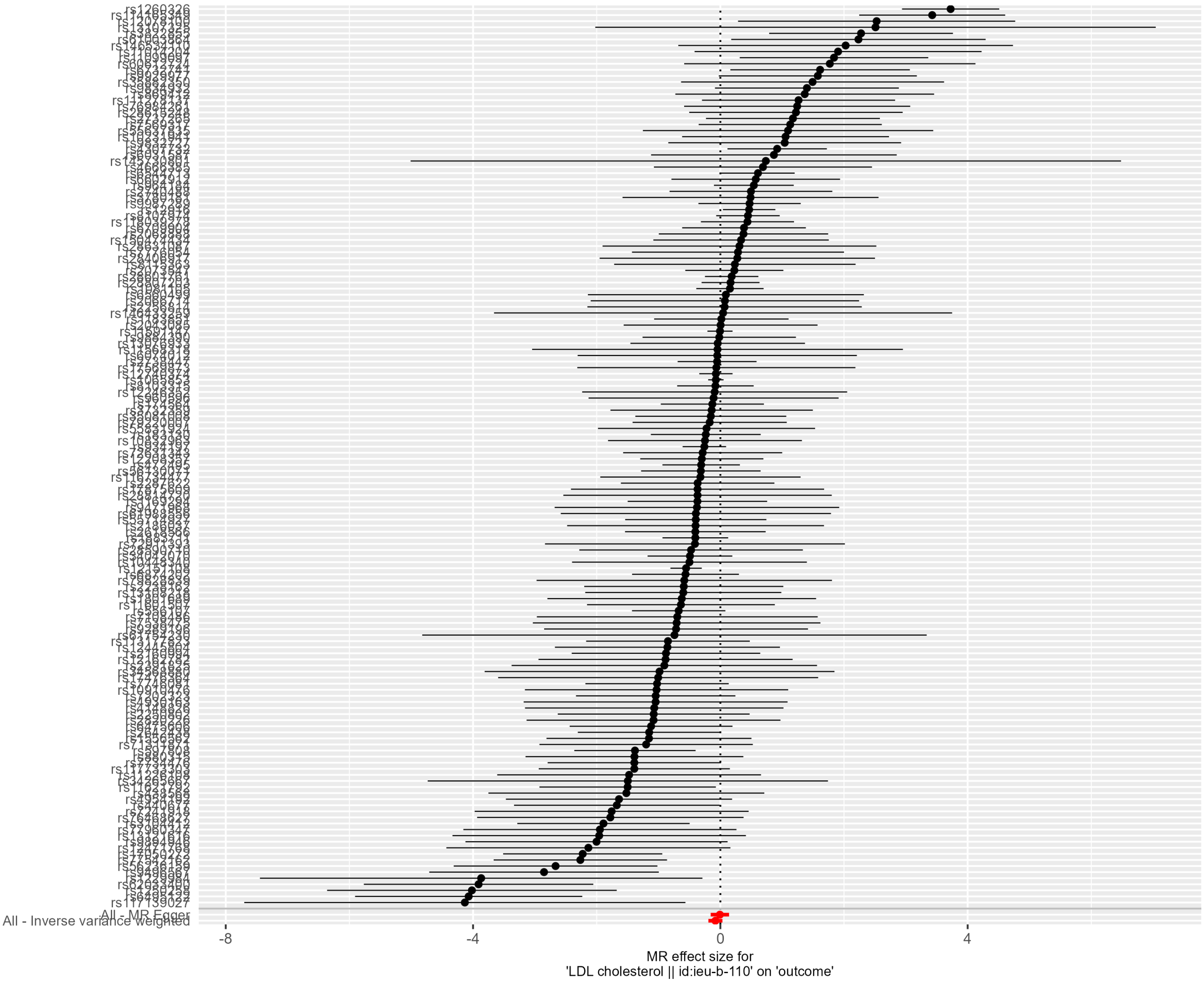

rs1260326
rs117139027
All - MR Egger
All - Inverse variance weighted
-8
-4
0
4
MR effect size for
'LDL cholesterol || id:ieu-b-110' on 'outcome'

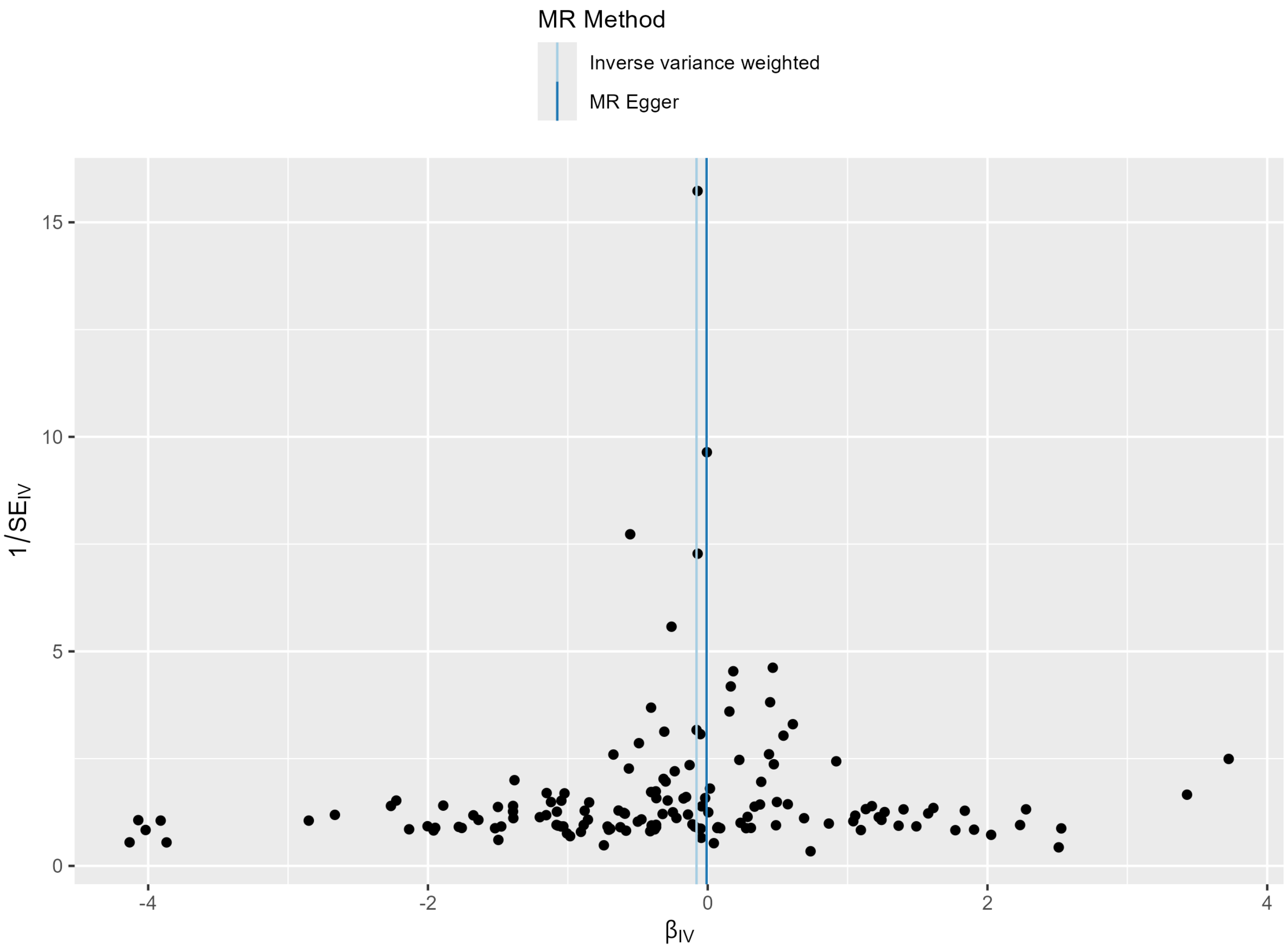

MR Method
Inverse variance weighted
MR Egger
1/SE_IV
β_IV
15
10
5
0
-4
-2
0
2
4

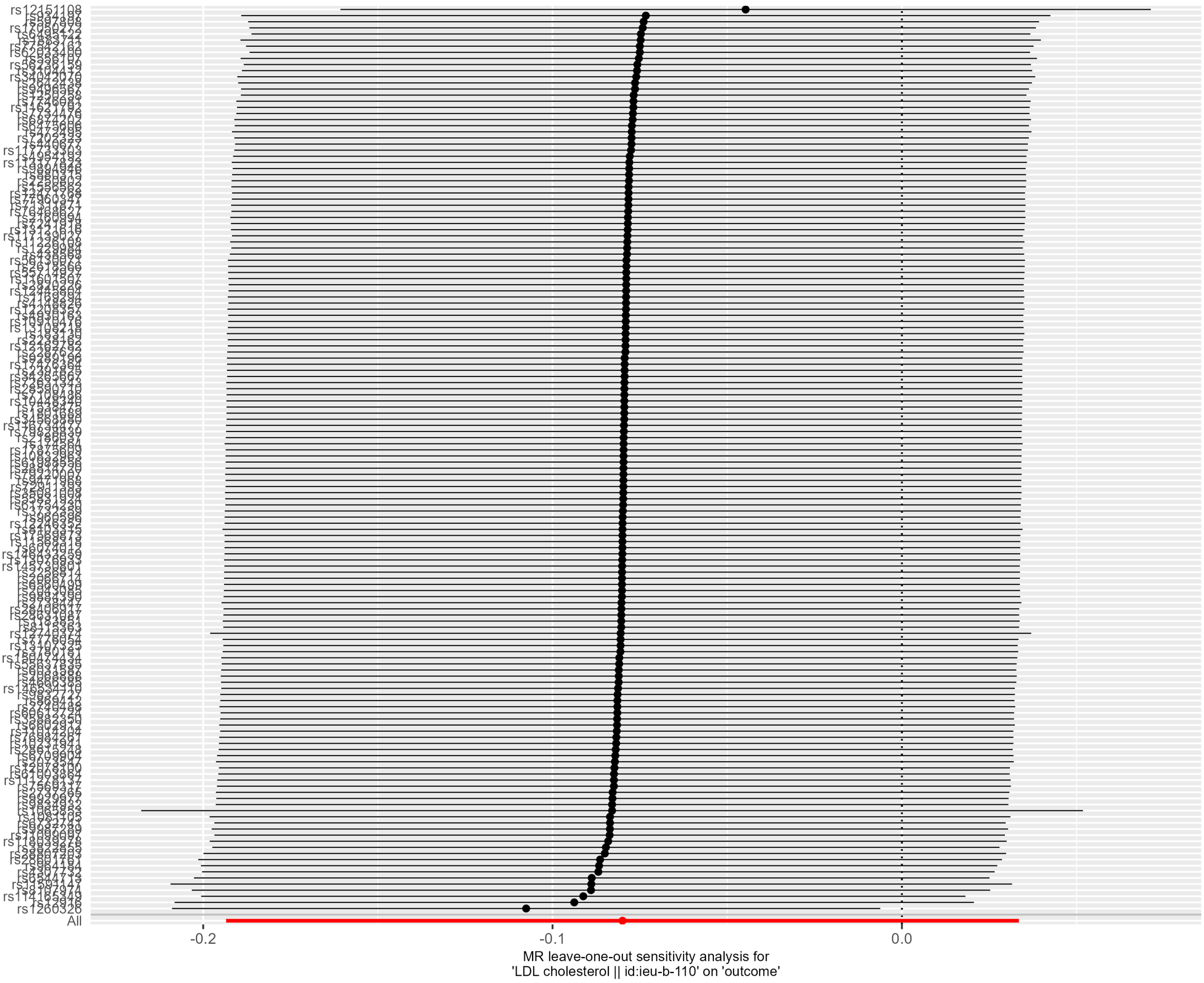
rs12151108
rs1260326
All
-0.2
-0.1
0.0
MR leave-one-out sensitivity analysis for
'LDL cholesterol || id:ieu-b-110' on 'outcome'

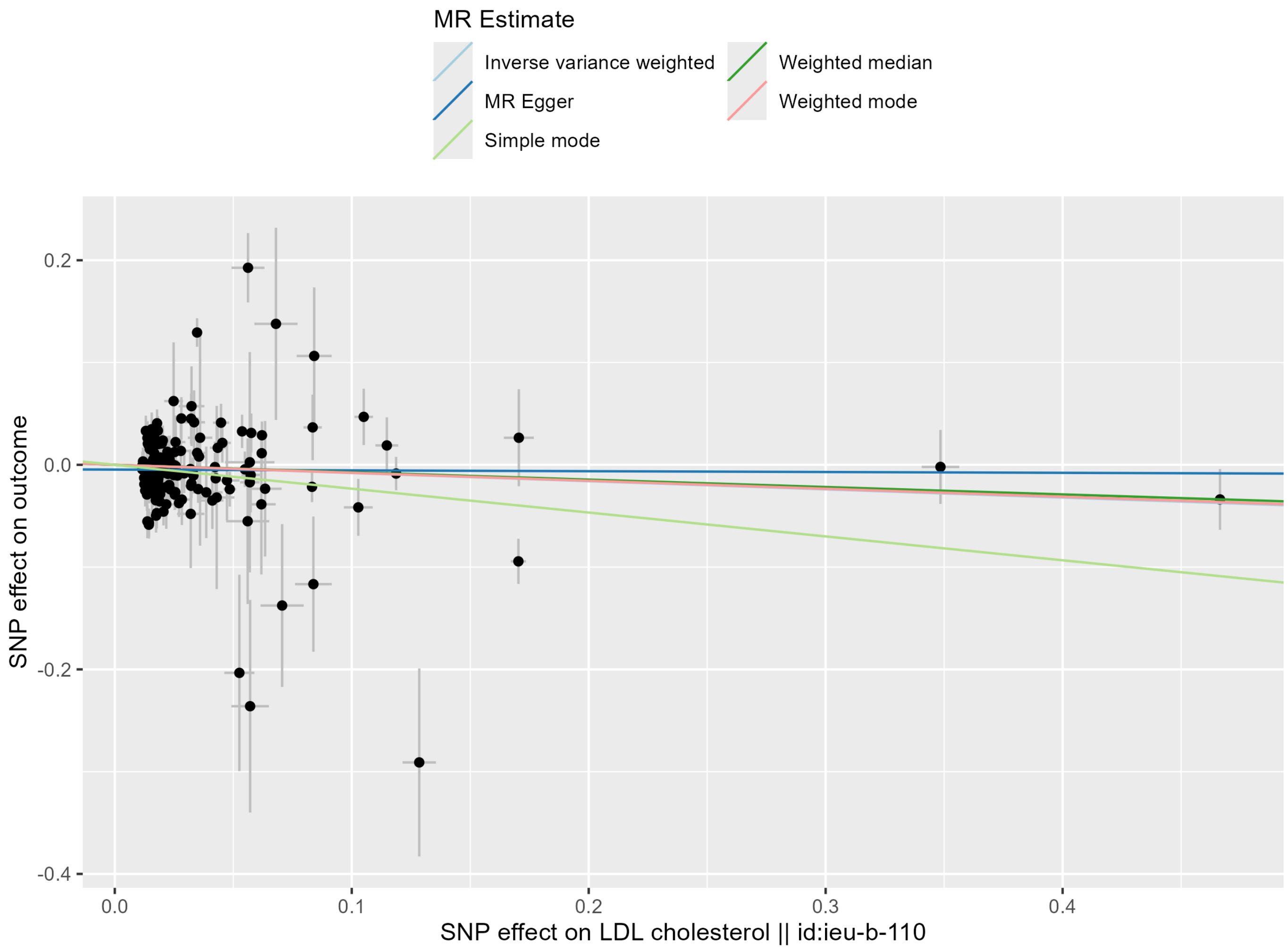
MR Estimate
Inverse variance weighted
MR Egger
Simple mode
Weighted median
Weighted mode
0.2
0.0
-0.2
-0.4
SNP effect on outcome
0.0
0.1
0.2
0.3
0.4
SNP effect on LDL cholesterol || id:ieu-b-110

甘油三酯_MR

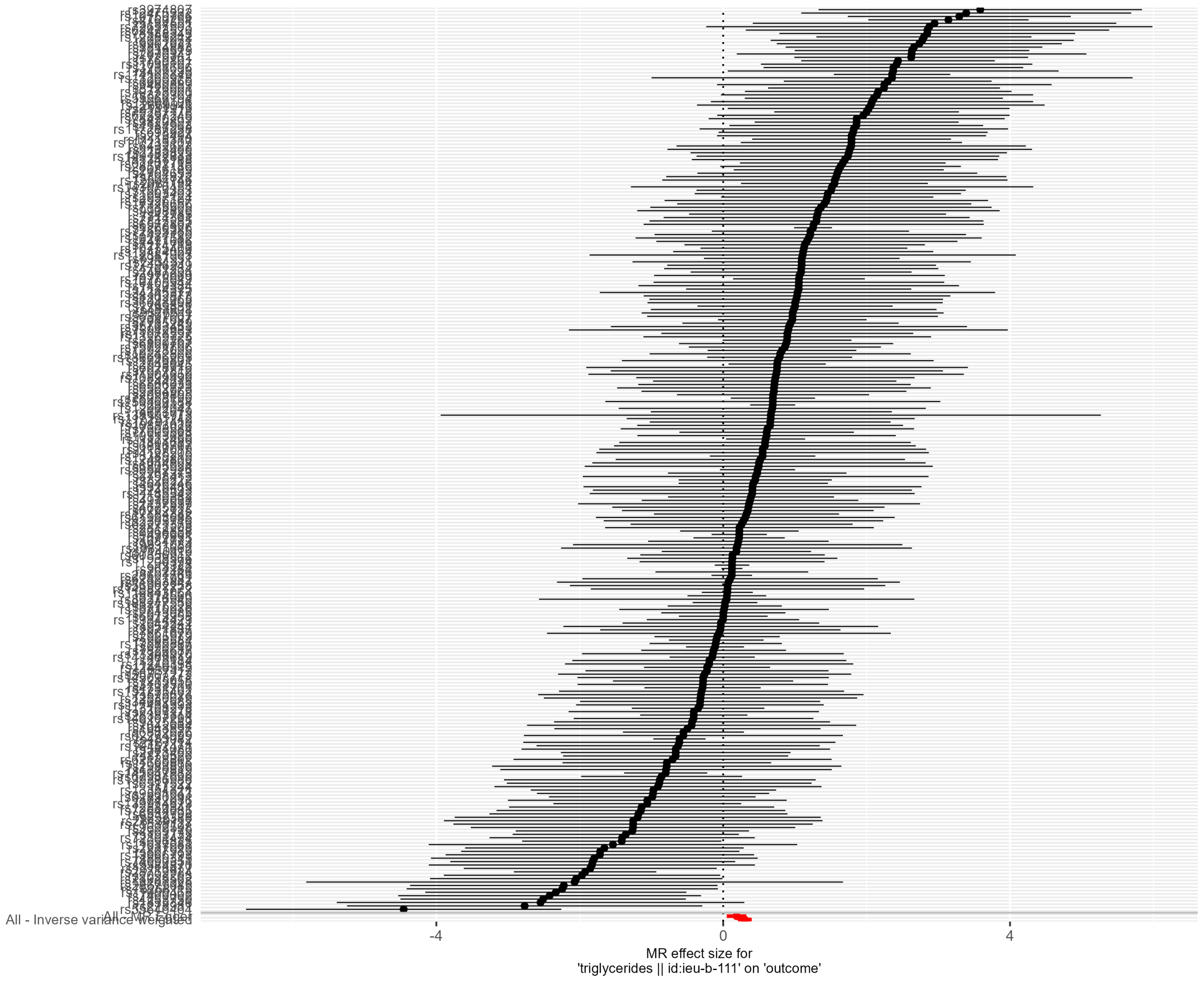

All - Inverse variance weighted
-4
0
4
MR effect size for
'triglycerides || id:ieu-b-111' on 'outcome'

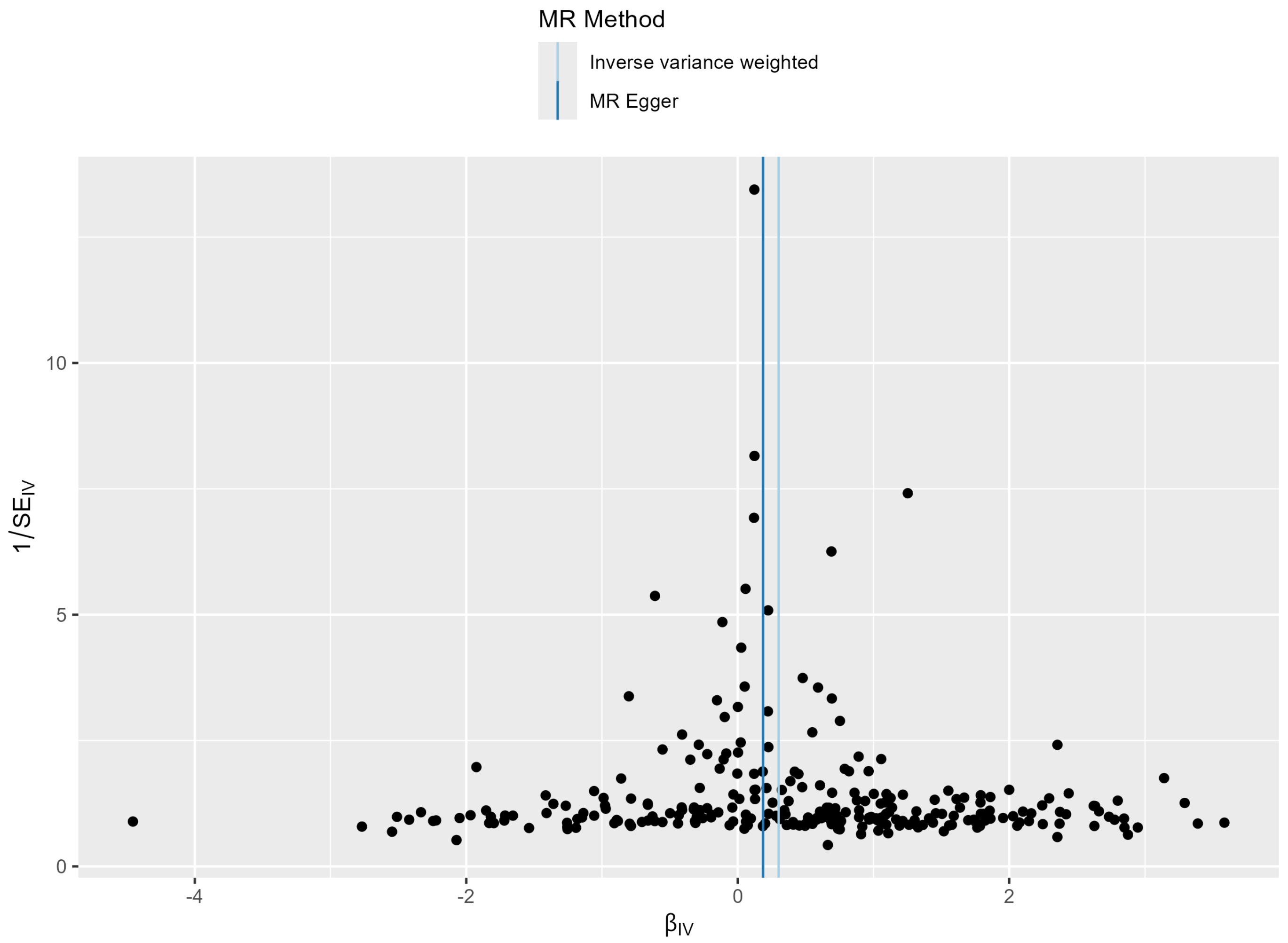

MR Method
Inverse variance weighted
MR Egger
1/SE_IV
β_IV
0
5
10
-4
-2
0
2

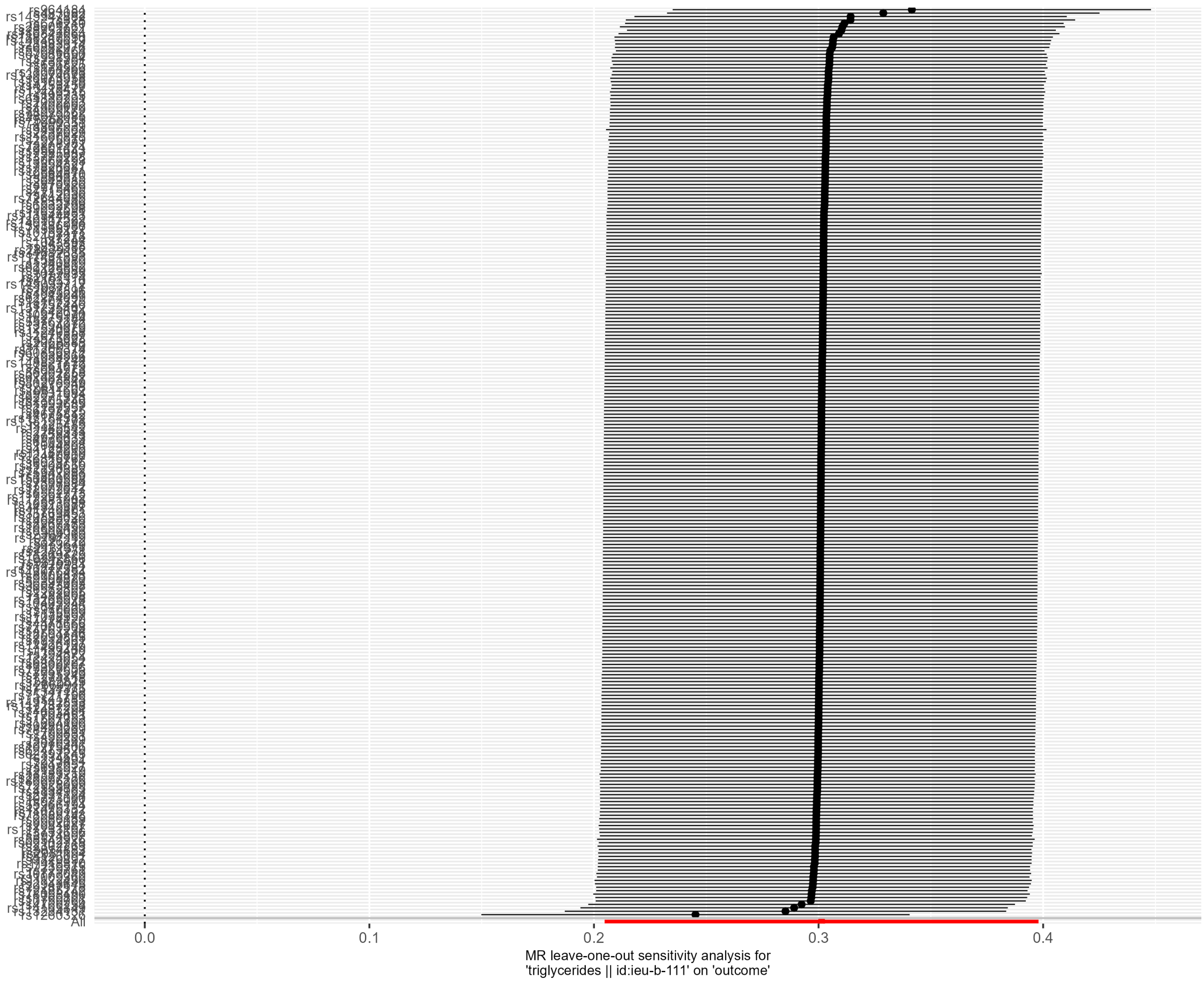
All
0.0
0.1
0.2
0.3
0.4
MR leave-one-out sensitivity analysis for
'triglycerides || id:ieu-b-111' on 'outcome'

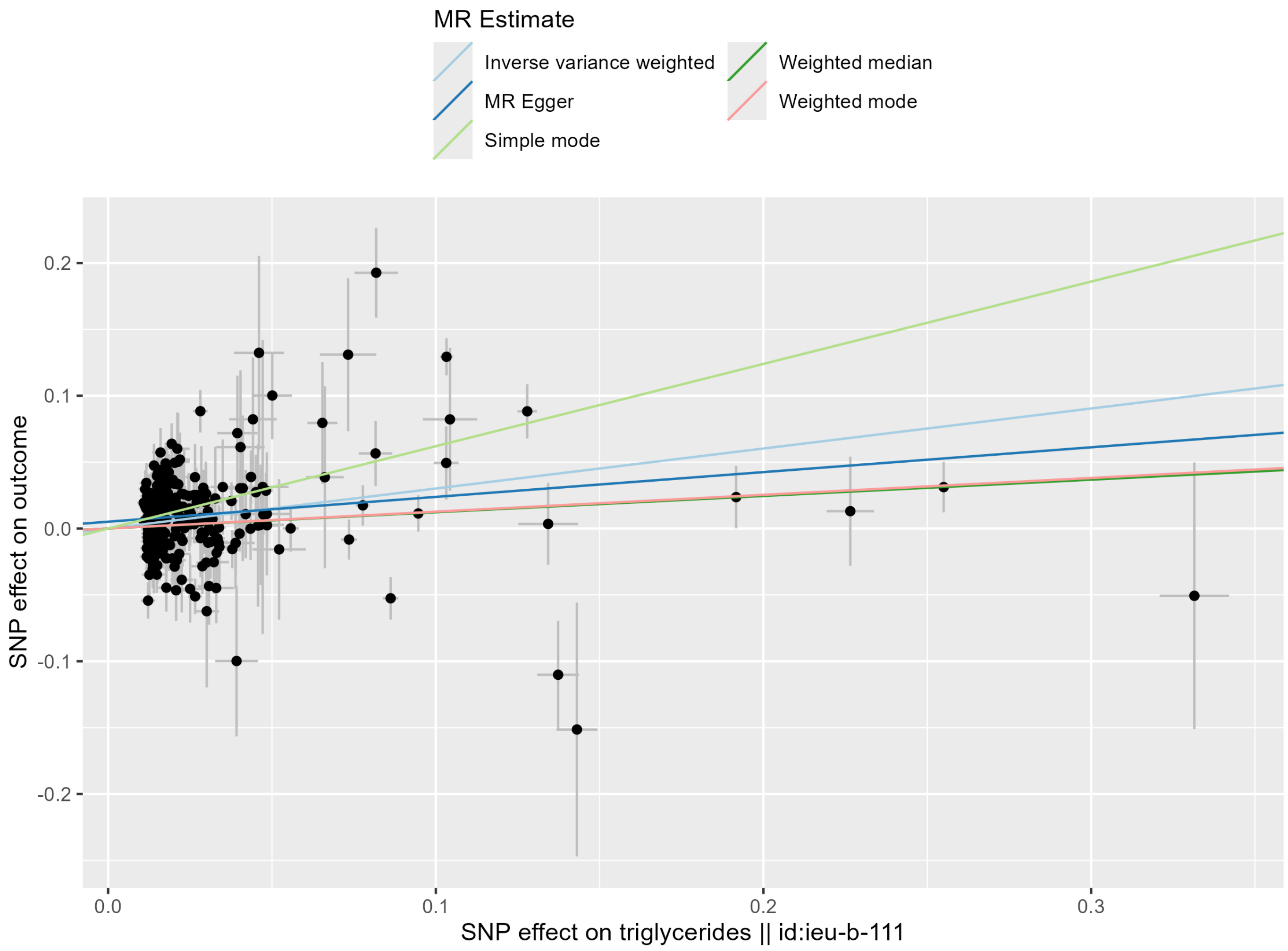

MR Estimate
Inverse variance weighted
MR Egger
Simple mode
Weighted median
Weighted mode
0.2
0.1
0.0
-0.1
-0.2
SNP effect on outcome
0.0
0.1
0.2
0.3
SNP effect on triglycerides || id:ieu-b-111

高密度蛋白_MR

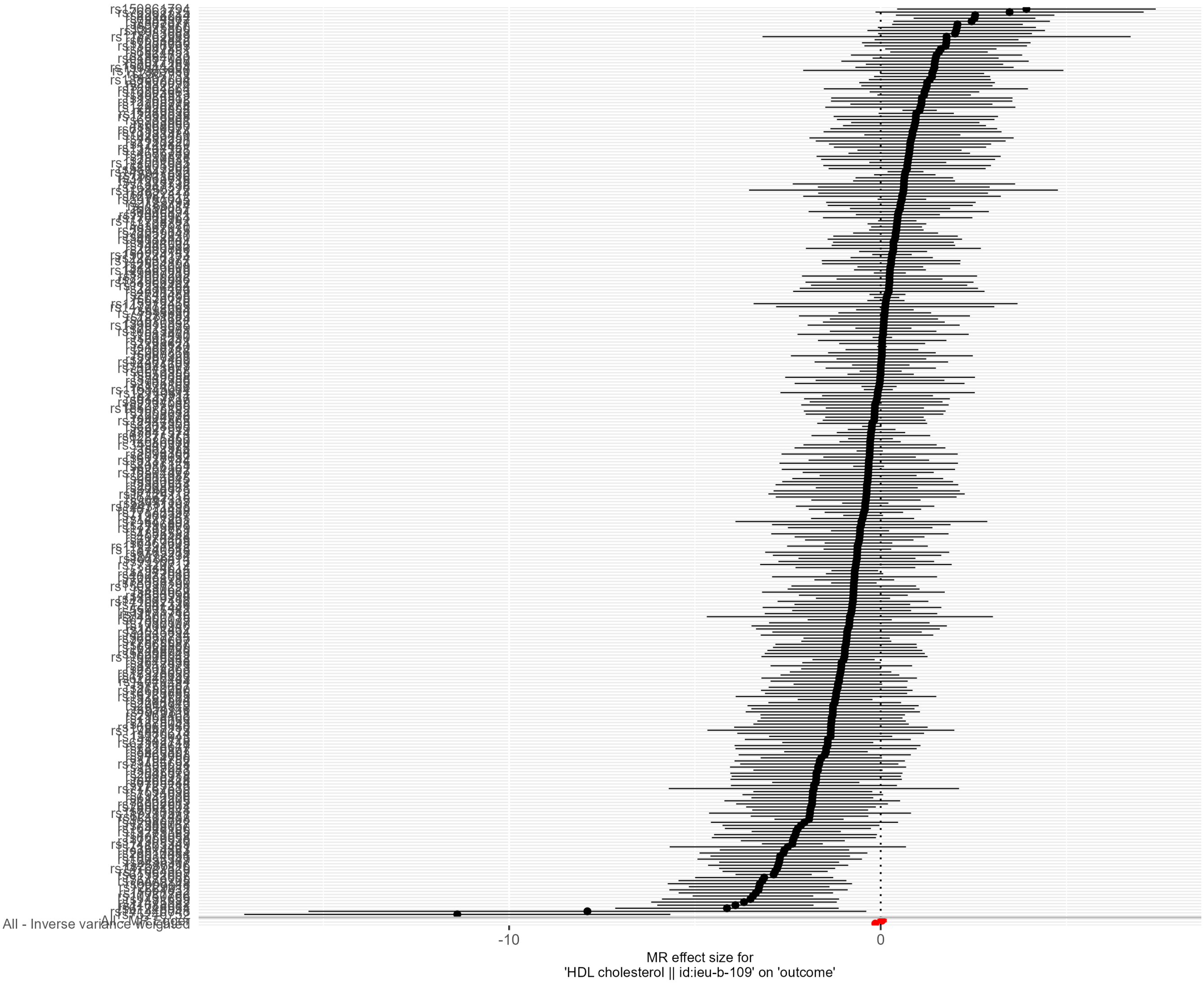
rs150861794
All - Inverse variance weighted
-10
0
MR effect size for
'HDL cholesterol || id:ieu-b-109' on 'outcome'

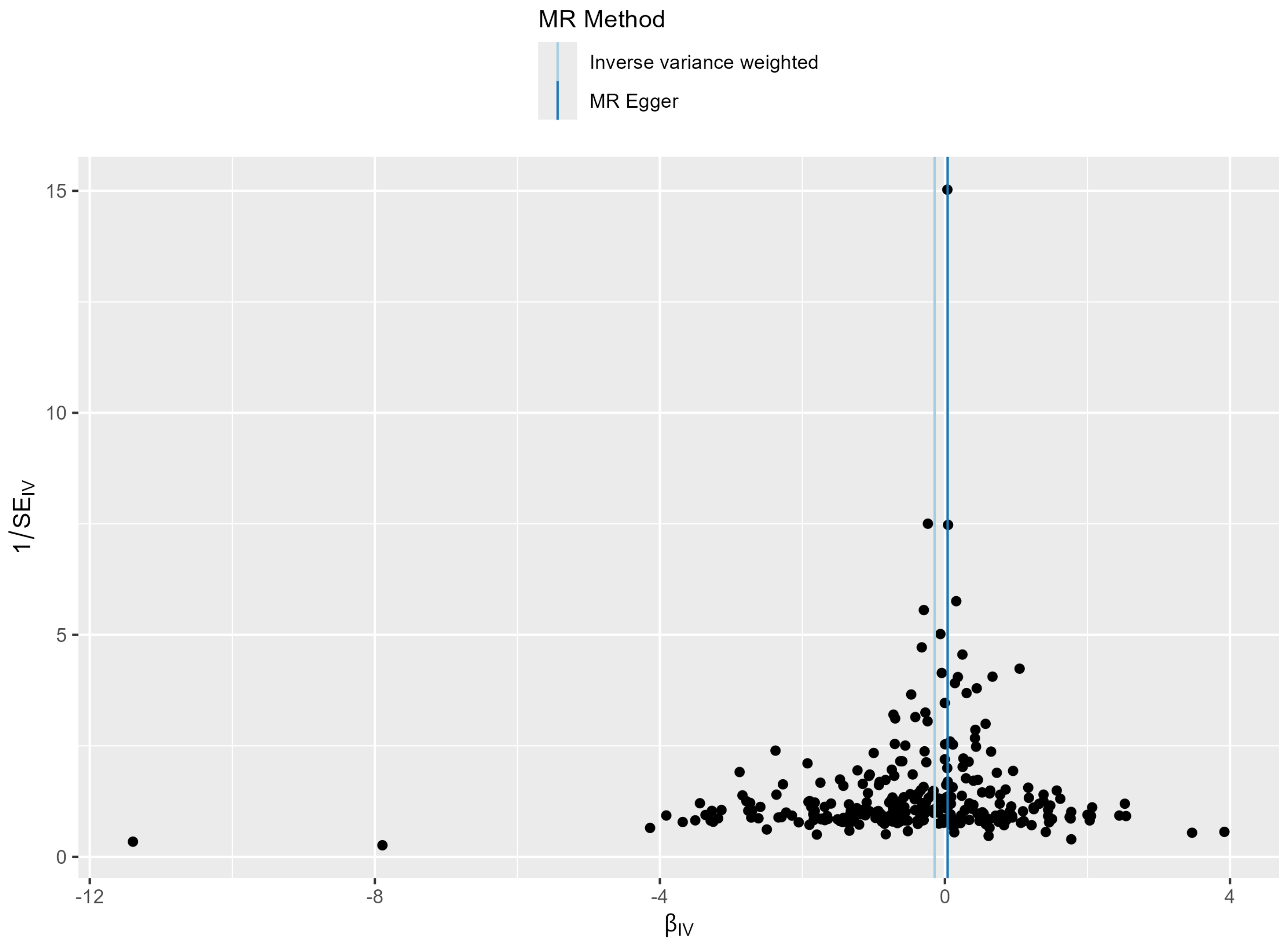
MR Method
Inverse variance weighted
MR Egger
15
10
5
0
$1/SE_{IV}$
-12
-8
-4
0
4
β_{IV}

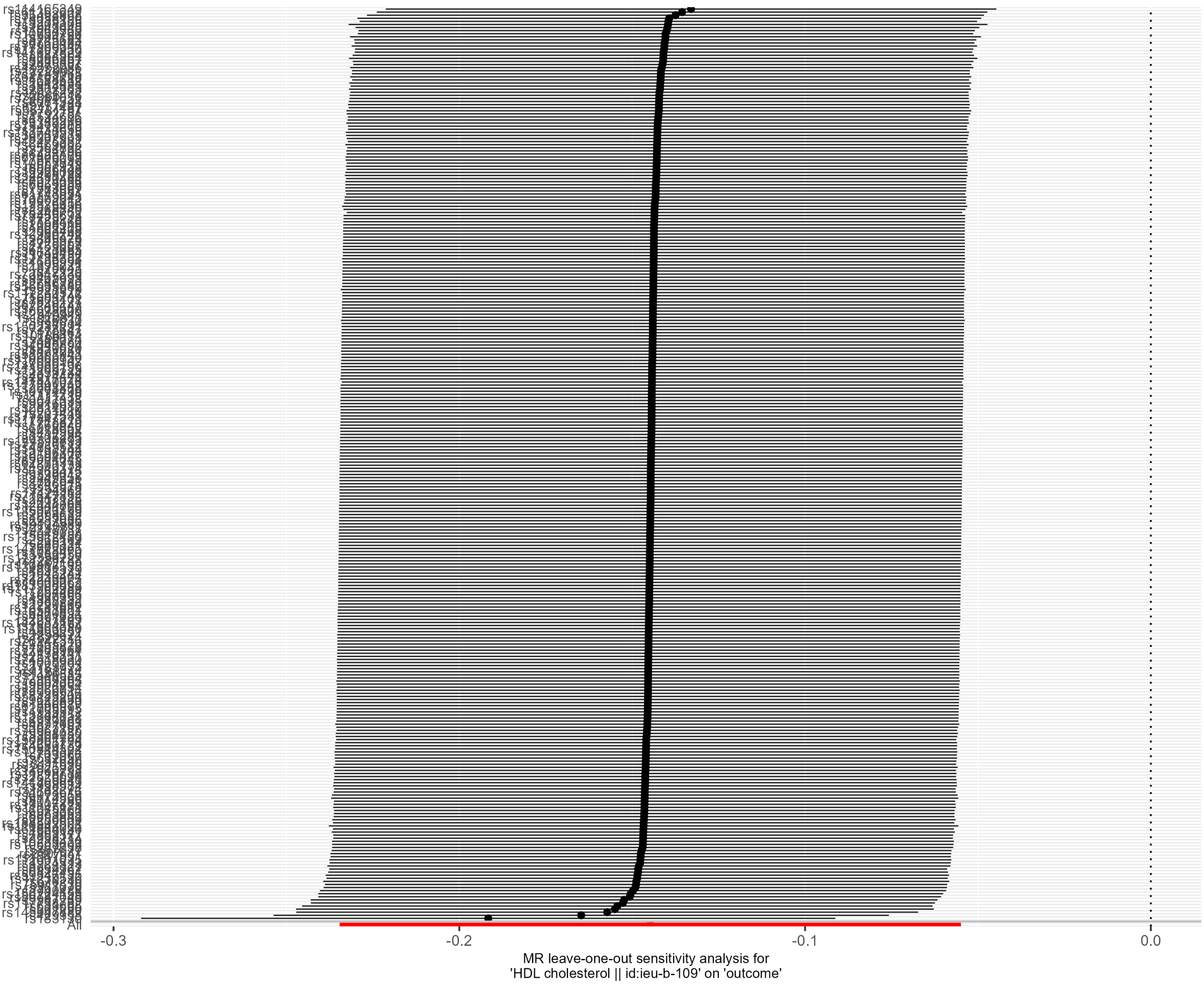

All
-0.3
-0.2
-0.1
0.0
MR leave-one-out sensitivity analysis for
'HDL cholesterol || id:ieu-b-109' on 'outcome'

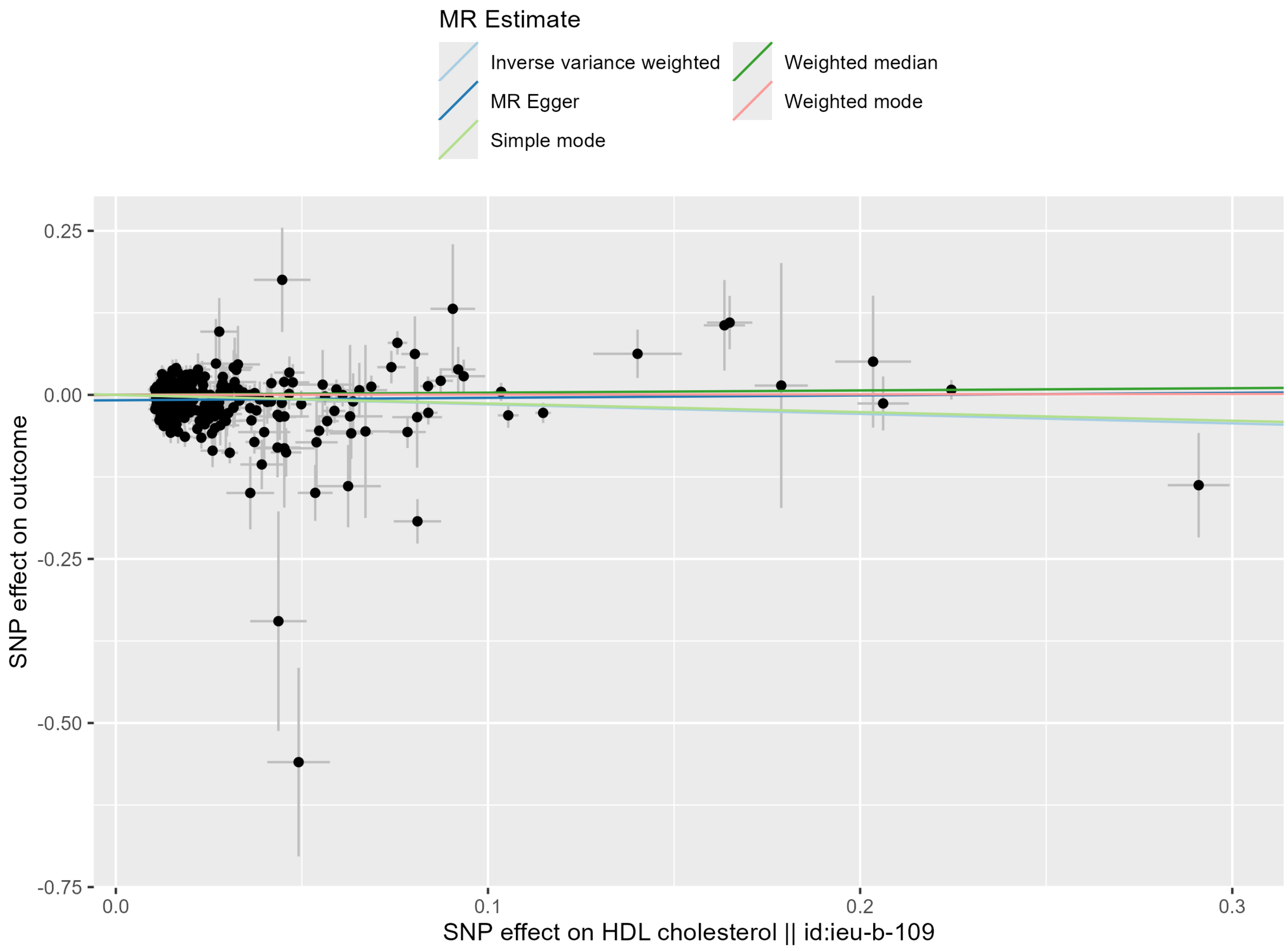
MR Estimate
Inverse variance weighted
MR Egger
Simple mode
Weighted median
Weighted mode
0.25
0.00
-0.25
-0.50
-0.75
0.0
0.1
0.2
0.3
SNP effect on outcome
SNP effect on HDL cholesterol || id:ieu-b-109
