# Supplementary material for: Decoding the lipid etiology of atherogenic index of plasma and gout: establishing the causal role of triglycerides through NHANES, Mendelian randomization, and network pharmacology
Source: Cardiovasc Diabetol Endocrinol Rep. 2026 Jul 13;12:40. doi: 10.1186/s40842-026-00309-0 (PMC13362044; doi:10.1186/s40842-026-00309-0)
Supplement: Supplementary file 5 — Supplementary Material 5 [file 40842_2026_309_MOESM5_ESM.pdf]

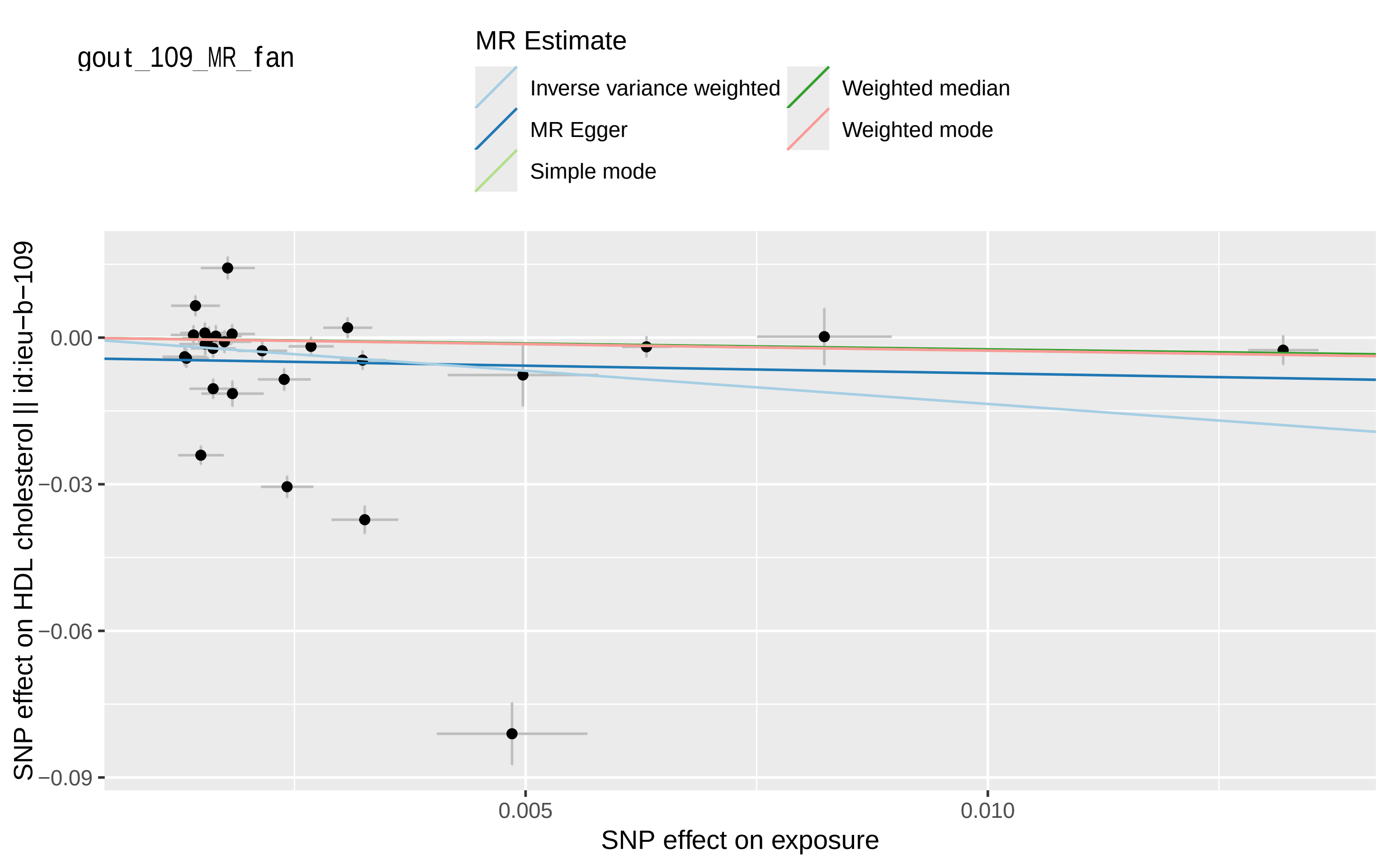
gout_109_MR_fan
MR Estimate
Inverse variance weighted
MR Egger
Simple mode
Weighted median
Weighted mode
SNP effect on HDL cholesterol || id:ieu-b-109
0.00
−0.03
−0.06
−0.09
0.005
0.010
SNP effect on exposure

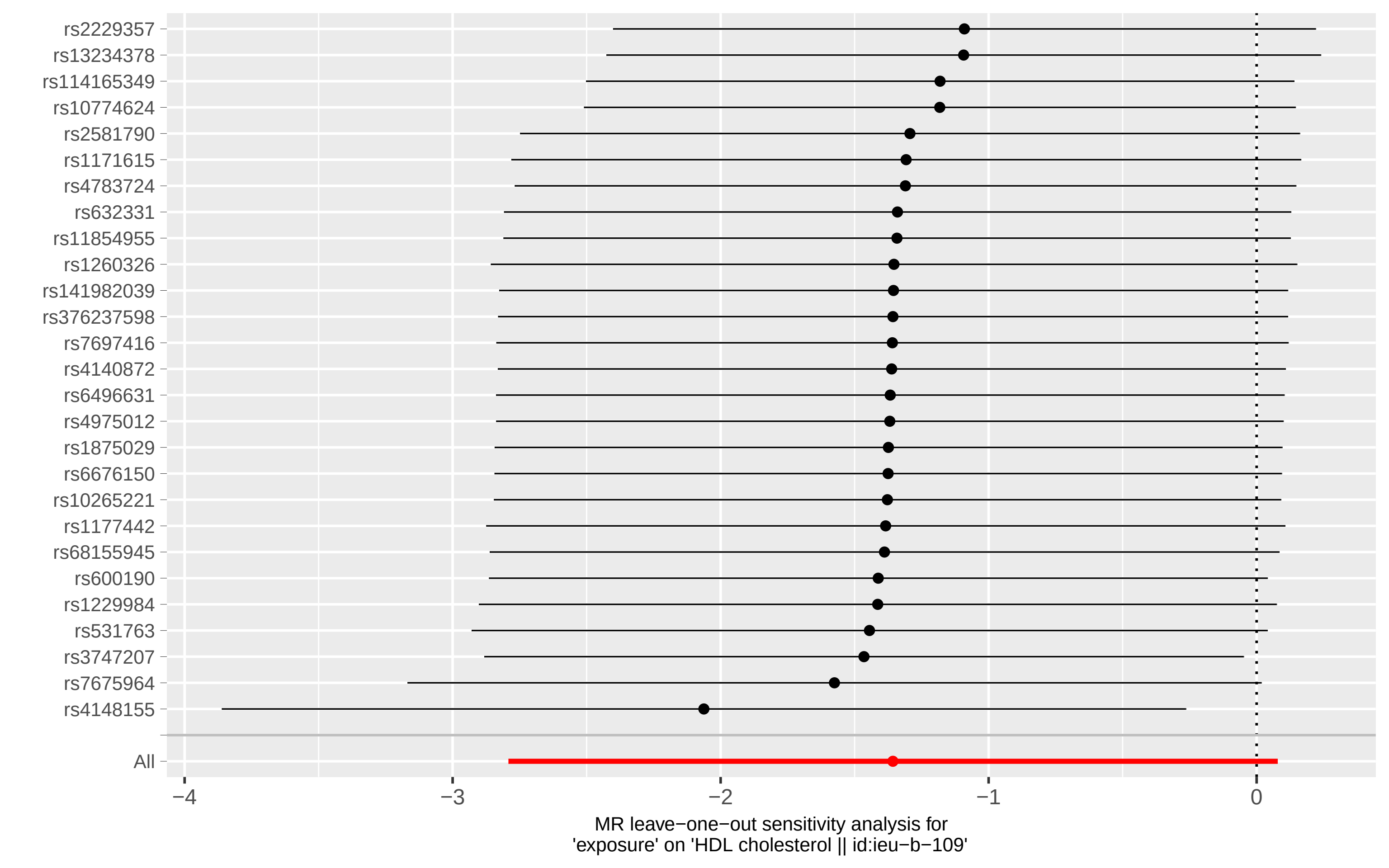

rs2229357
rs13234378
rs114165349
rs10774624
rs2581790
rs1171615
rs4783724
rs632331
rs11854955
rs1260326
rs141982039
rs376237598
rs7697416
rs4140872
rs6496631
rs4975012
rs1875029
rs6676150
rs10265221
rs1177442
rs68155945
rs600190
rs1229984
rs531763
rs3747207
rs7675964
rs4148155
All
−4
−3
−2
−1
0
MR leave−one−out sensitivity analysis for
'exposure' on 'HDL cholesterol || id:ieu−b−109'

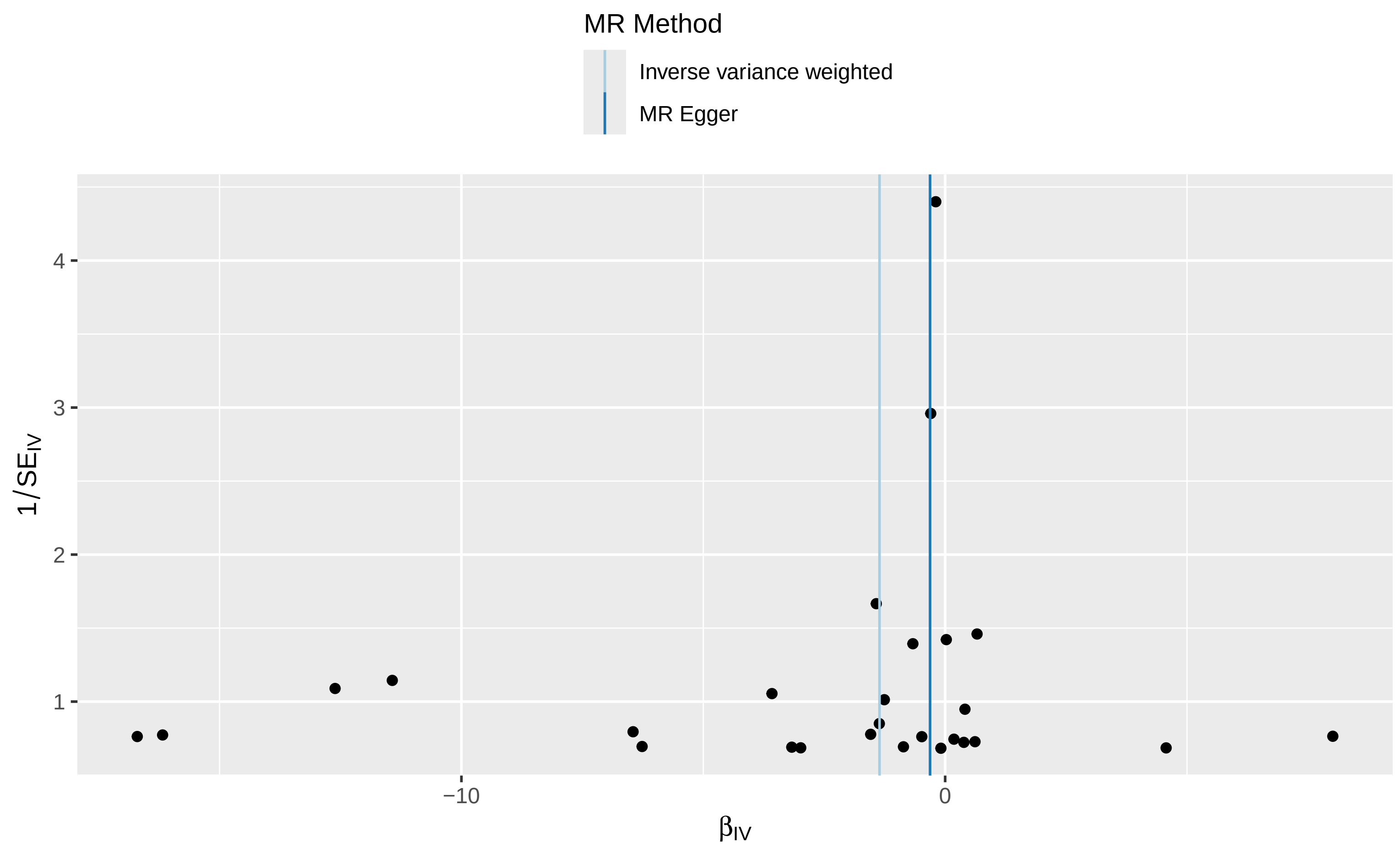
MR Method
Inverse variance weighted
MR Egger
4
3
2
1
$1/SE_{IV}$
−10
0
β_{IV}

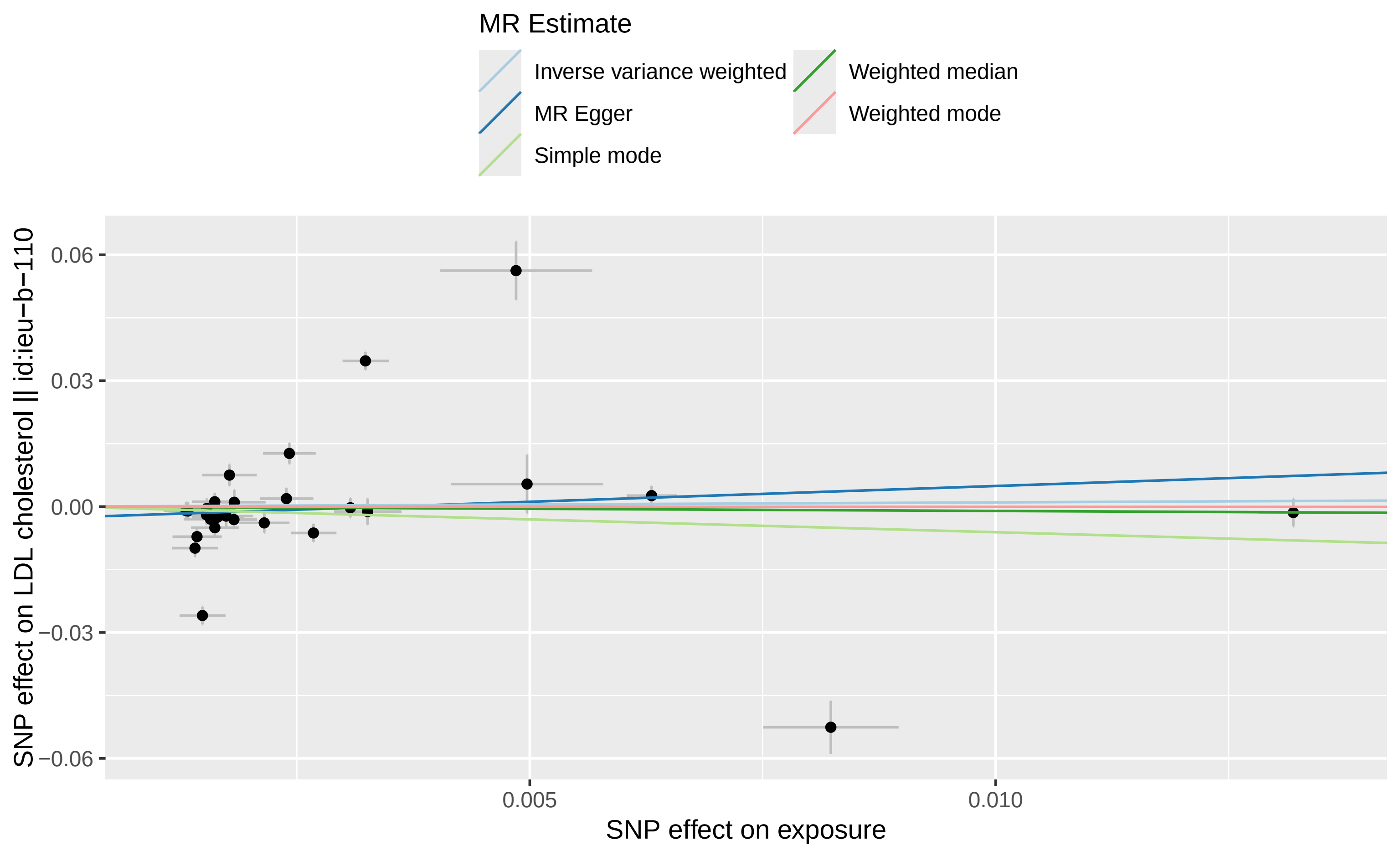
MR Estimate
Inverse variance weighted
MR Egger
Simple mode
Weighted median
Weighted mode
SNP effect on LDL cholesterol || id:ieu-b-110
0.06
0.03
0.00
−0.03
−0.06
0.005
0.010
SNP effect on exposure

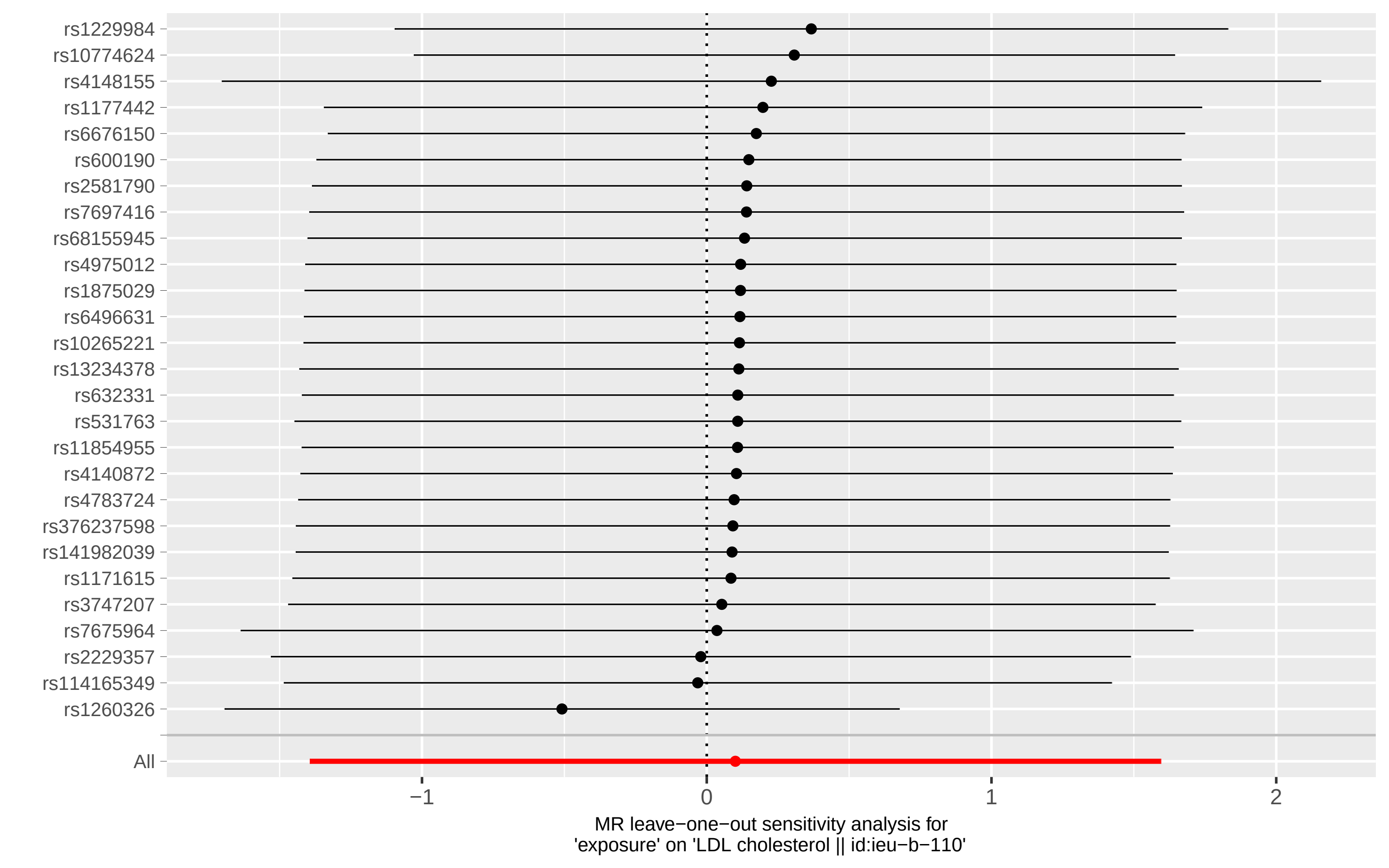
rs1229984
rs10774624
rs4148155
rs1177442
rs6676150
rs600190
rs2581790
rs7697416
rs68155945
rs4975012
rs1875029
rs6496631
rs10265221
rs13234378
rs632331
rs531763
rs11854955
rs4140872
rs4783724
rs376237598
rs141982039
rs1171615
rs3747207
rs7675964
rs2229357
rs114165349
rs1260326
All
−1
0
1
2
MR leave−one−out sensitivity analysis for
'exposure' on 'LDL cholesterol || id:ieu−b−110'

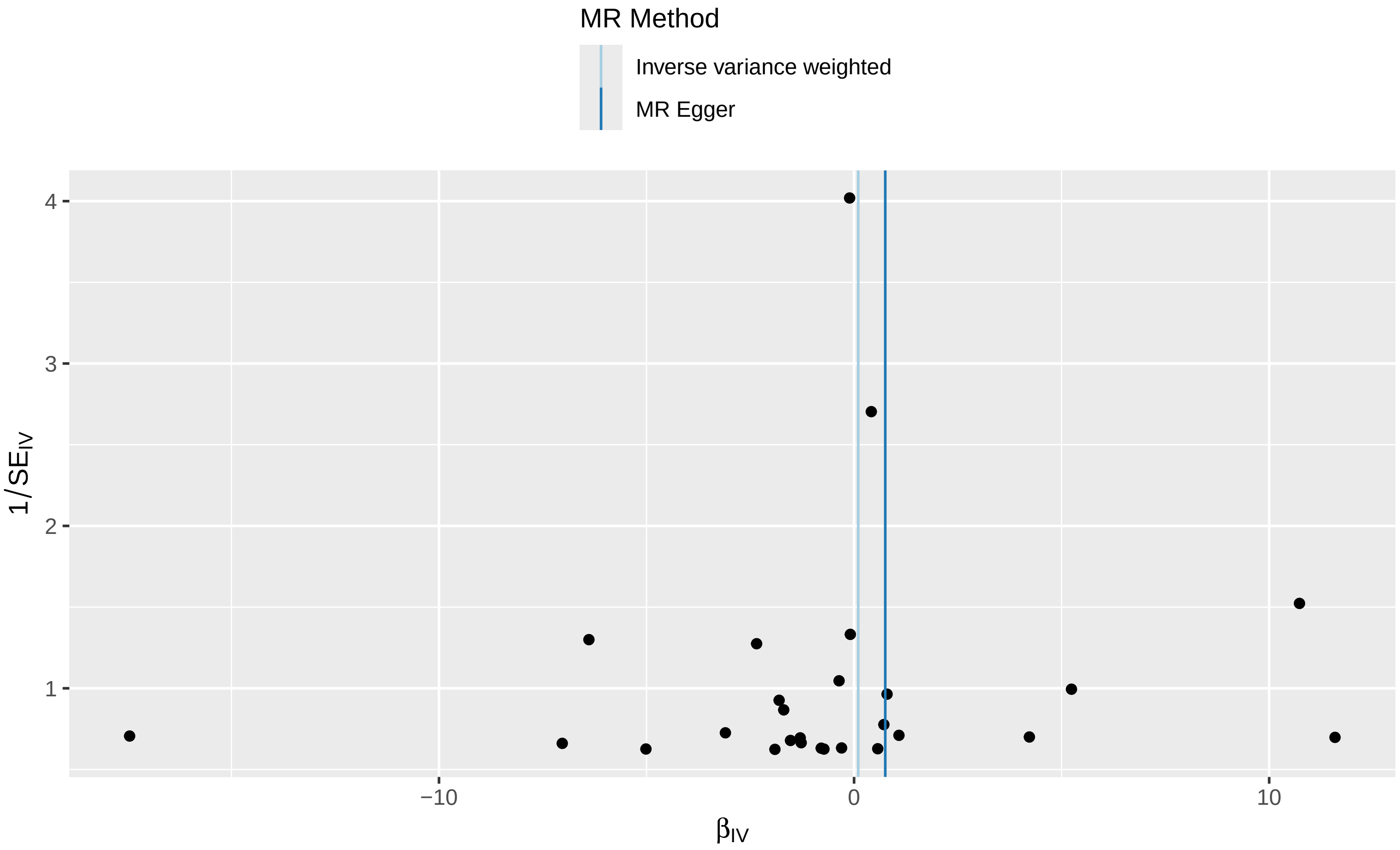

MR Method
Inverse variance weighted
MR Egger
4
3
2
1
1/SE_IV
−10
0
10
β_IV

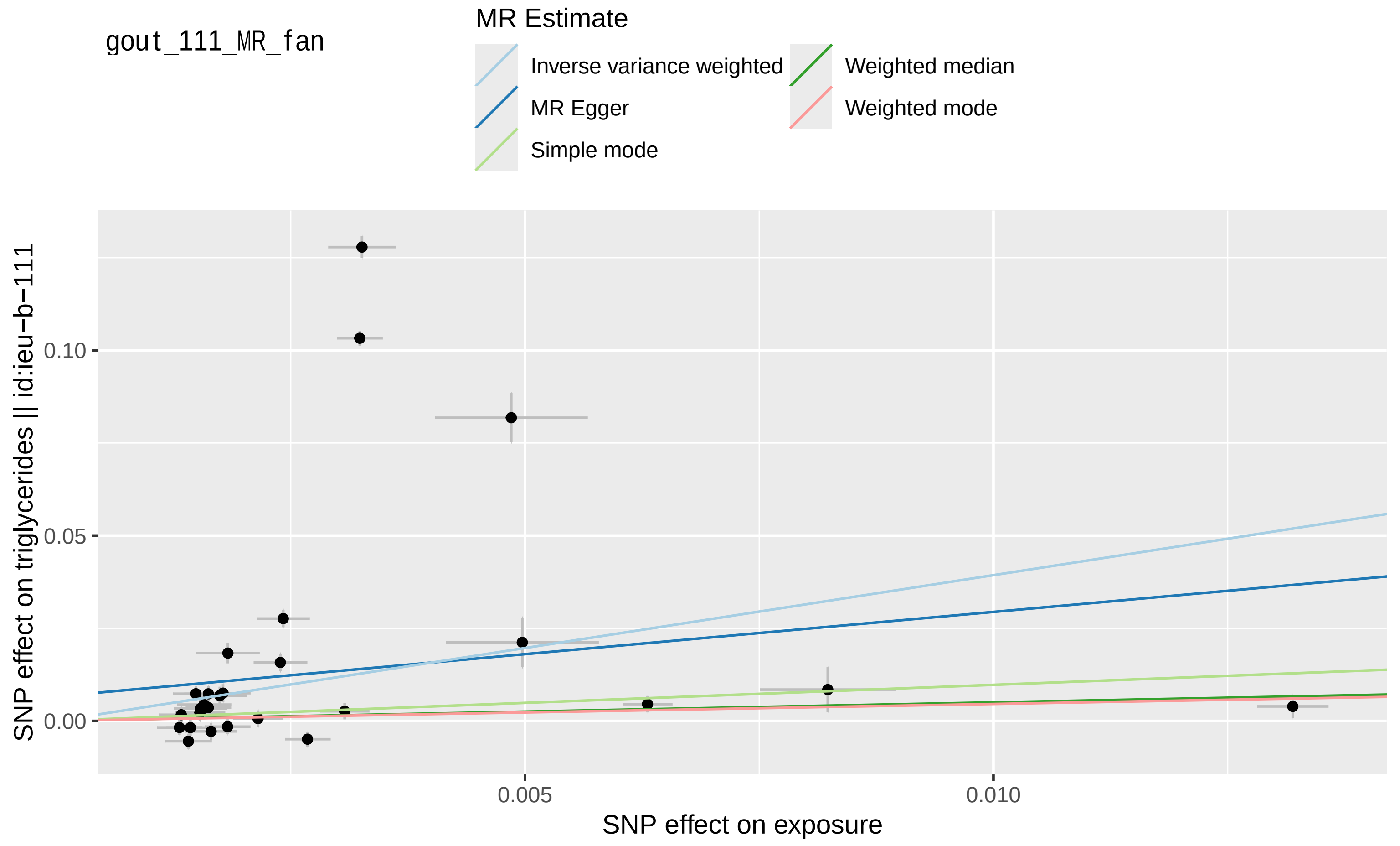
gout_111_MR_fan
MR Estimate
Inverse variance weighted
MR Egger
Simple mode
Weighted median
Weighted mode
SNP effect on triglycerides || id:ieu-b-111
0.10
0.05
0.00
0.005
0.010
SNP effect on exposure

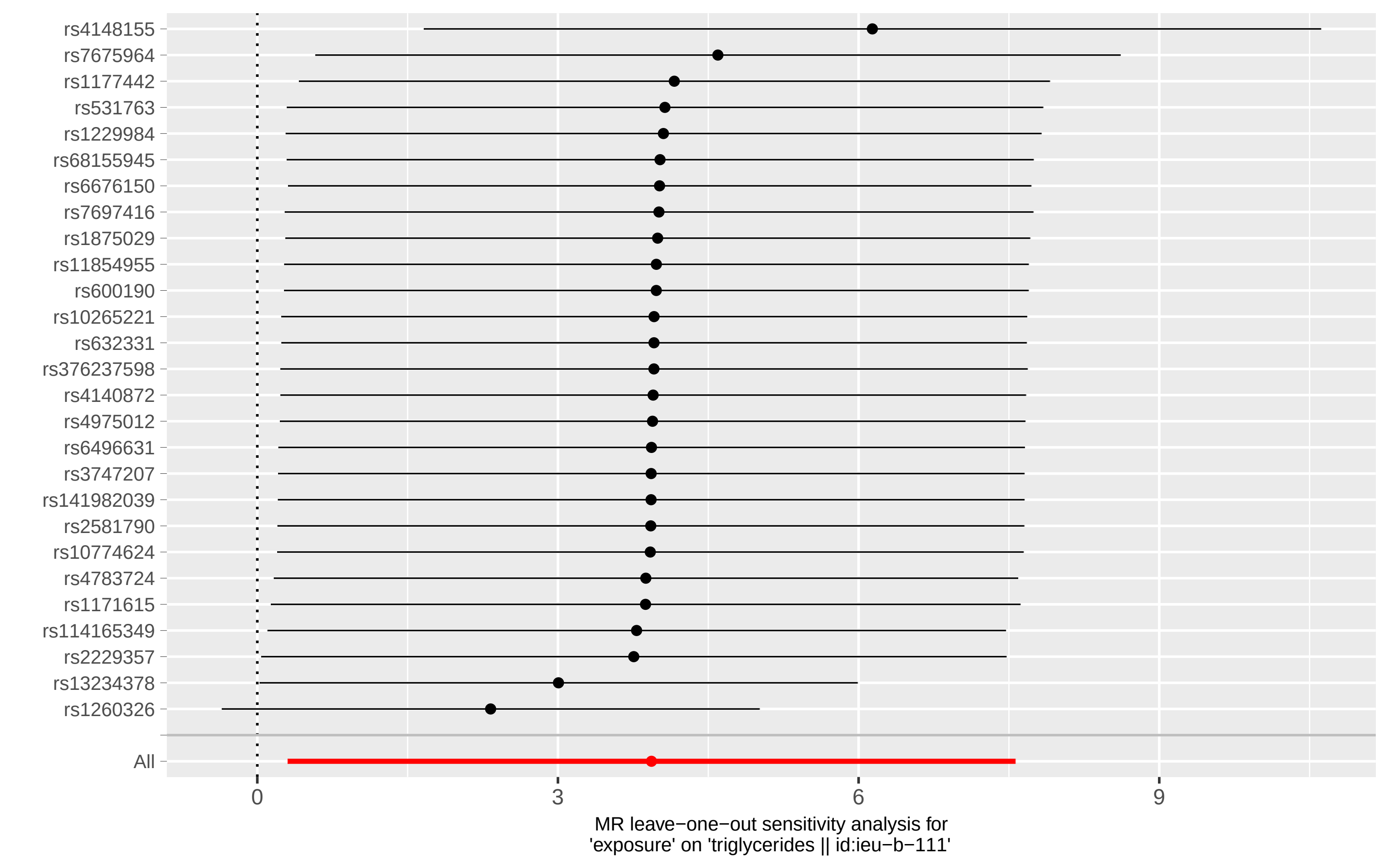
rs4148155
rs7675964
rs1177442
rs531763
rs1229984
rs68155945
rs6676150
rs7697416
rs1875029
rs11854955
rs600190
rs10265221
rs632331
rs376237598
rs4140872
rs4975012
rs6496631
rs3747207
rs141982039
rs2581790
rs10774624
rs4783724
rs1171615
rs114165349
rs2229357
rs13234378
rs1260326
All
0
3
6
9
MR leave−one−out sensitivity analysis for
'exposure' on 'triglycerides || id:ieu−b−111'

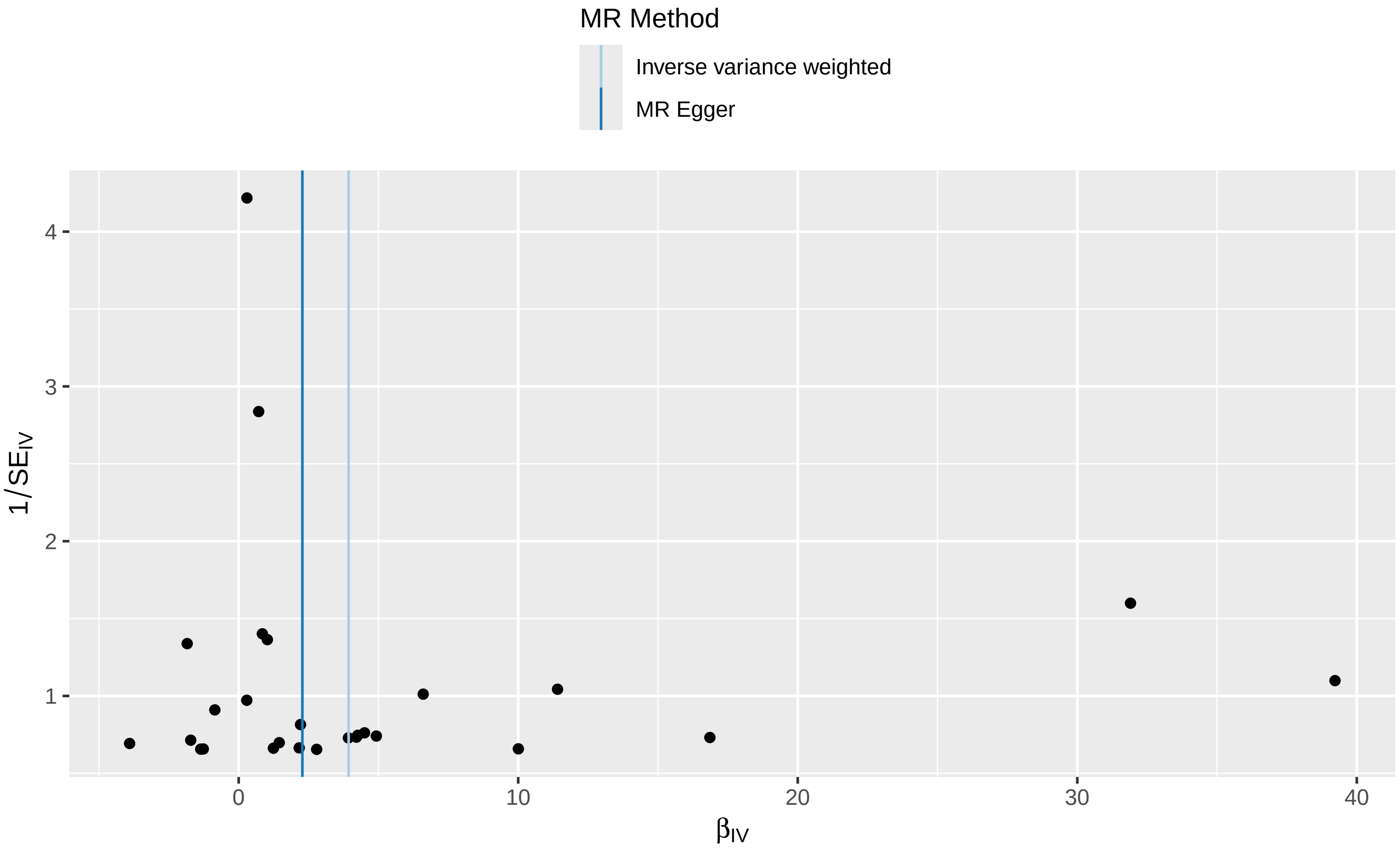

MR Method
Inverse variance weighted
MR Egger
4
3
2
1
$1/SE_{IV}$
0
10
20
30
40
β_{IV}

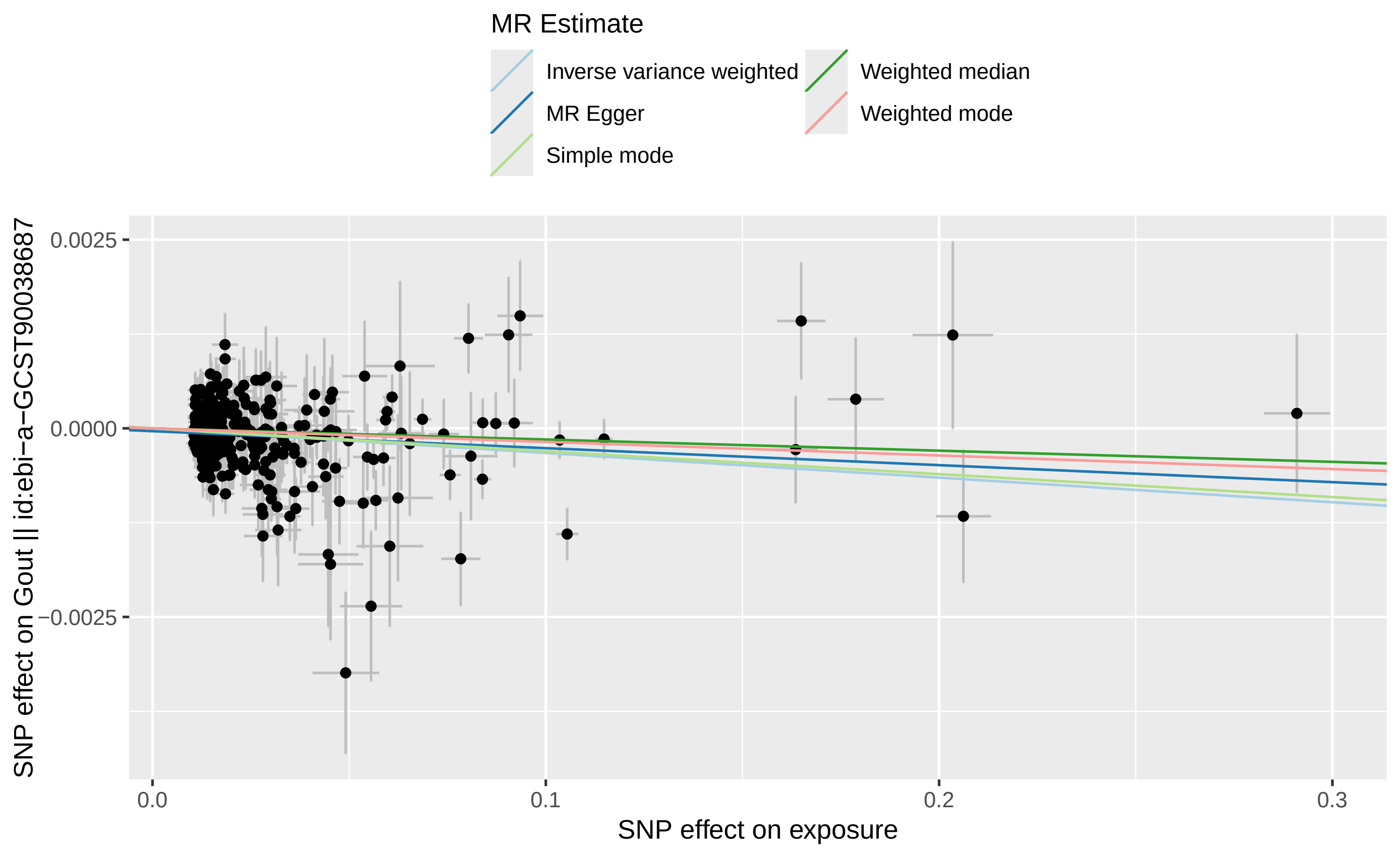

MR Estimate
Inverse variance weighted
Weighted median
MR Egger
Weighted mode
Simple mode
0.0025
0.0000
−0.0025
SNP effect on Gout || id:ebi−a−GCST90038687
0.0
0.1
0.2
0.3
SNP effect on exposure

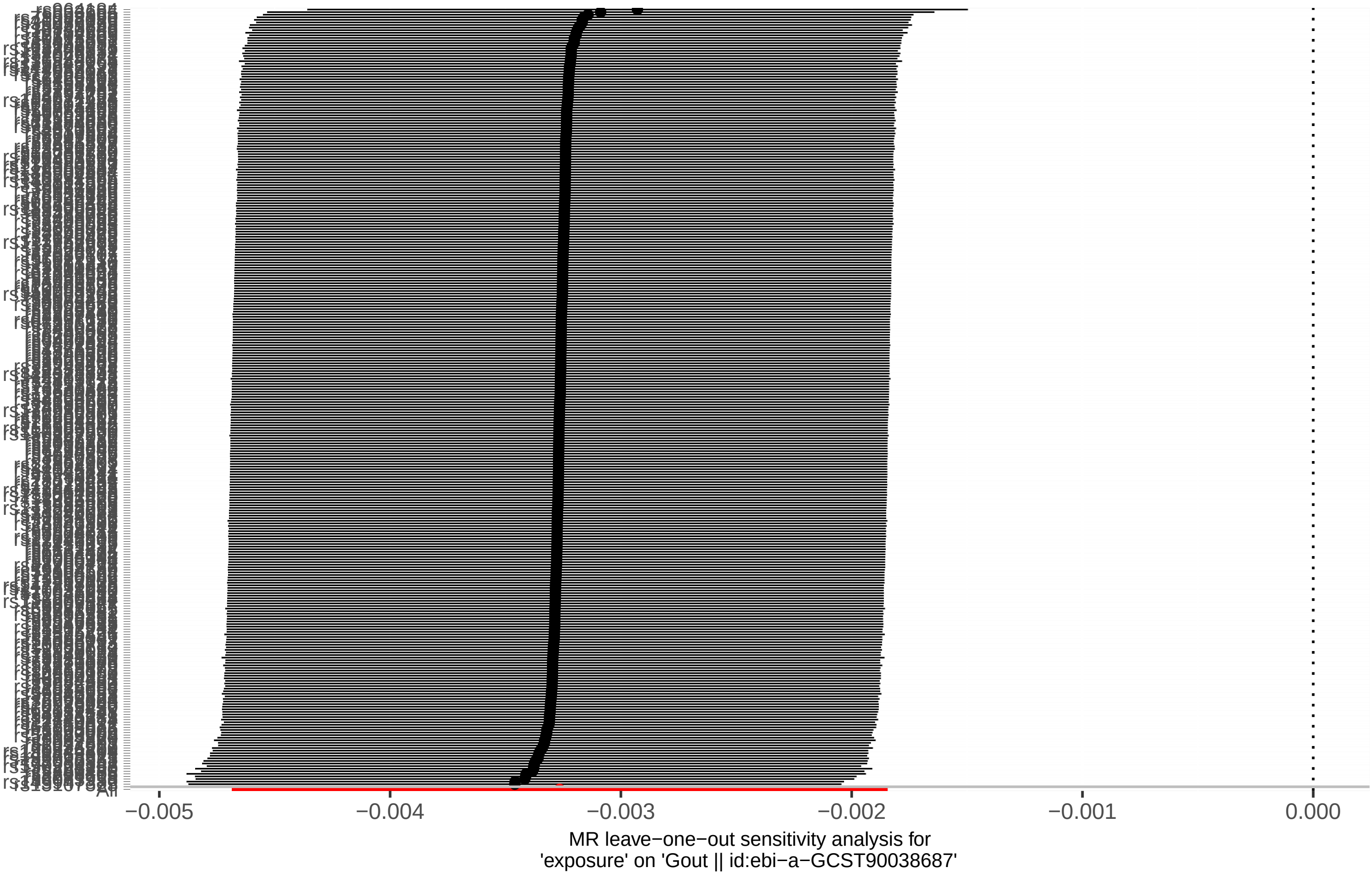
All
−0.005
−0.004
−0.003
−0.002
−0.001
0.000
MR leave−one−out sensitivity analysis for
'exposure' on 'Gout || id:ebi−a−GCST90038687'

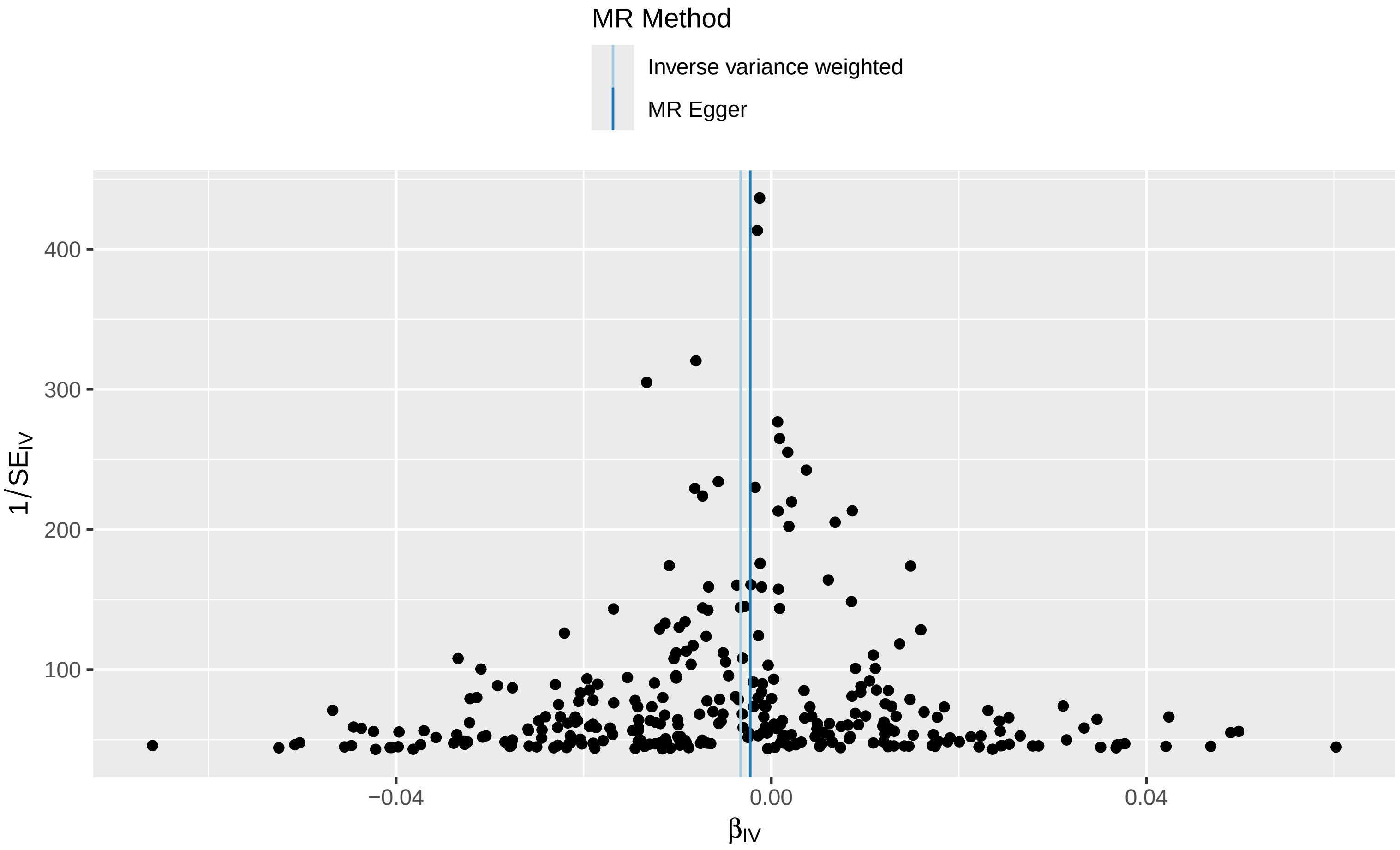

MR Method
Inverse variance weighted
MR Egger
400
300
200
100
1/SE$_{IV}$
−0.04
0.00
0.04
β_{IV}

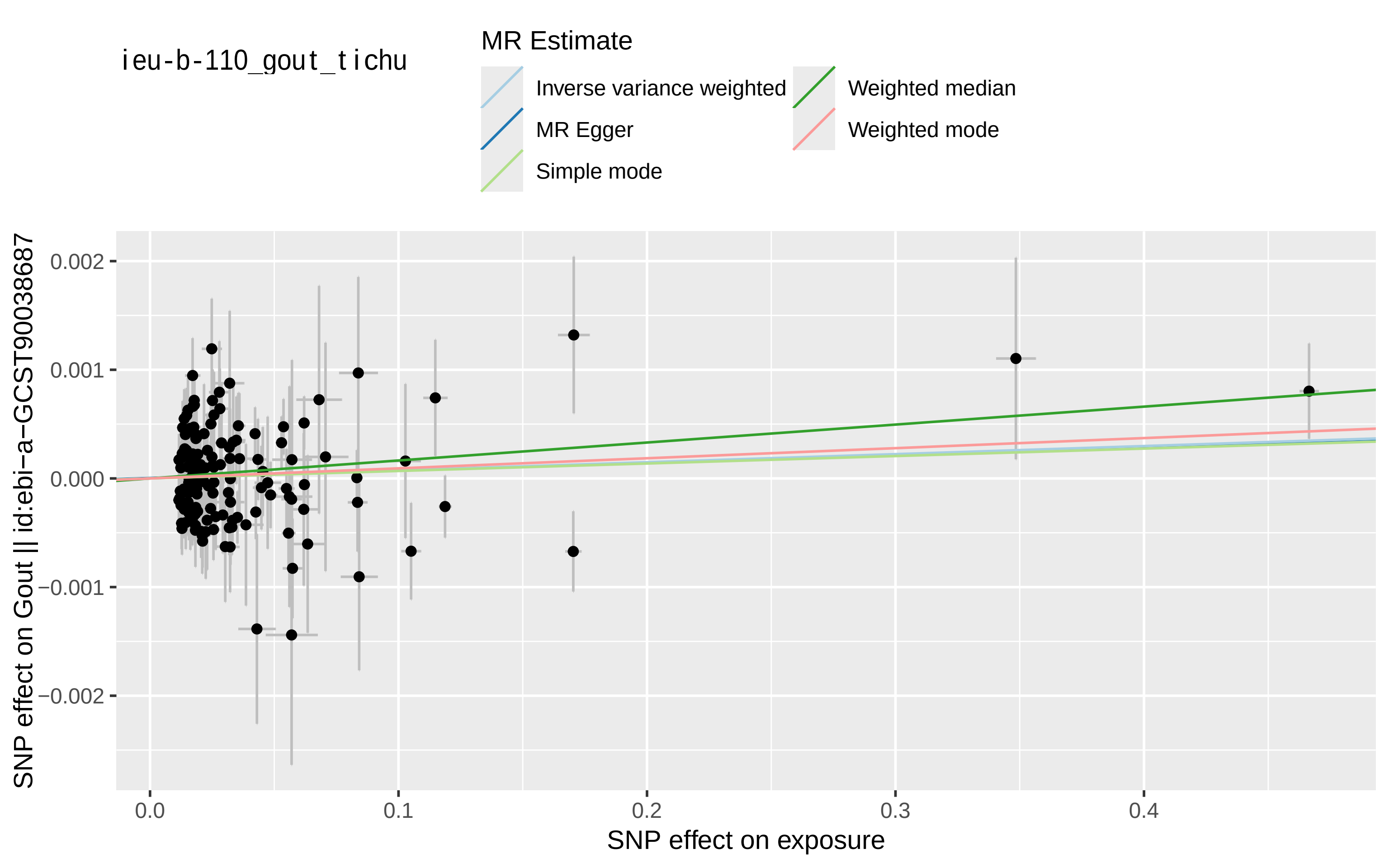

ieu-b-110_gout_tichu
MR Estimate
Inverse variance weighted
MR Egger
Simple mode
Weighted median
Weighted mode
SNP effect on Gout || id:ebi-a-GCST90038687
0.002
0.001
0.000
−0.001
−0.002
0.0
0.1
0.2
0.3
0.4
SNP effect on exposure

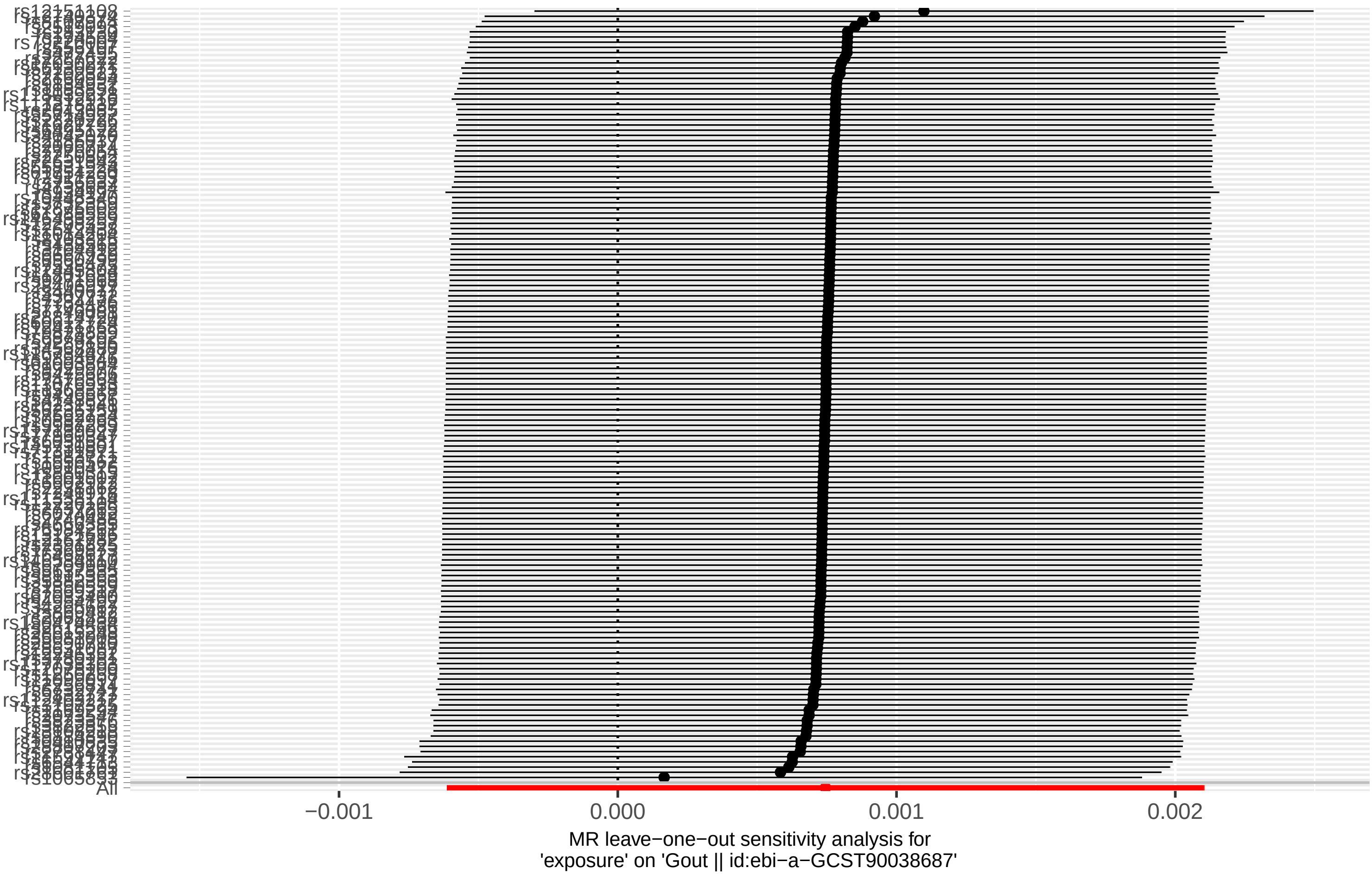

All
−0.001
0.000
0.001
0.002
MR leave−one−out sensitivity analysis for
'exposure' on 'Gout || id:ebi−a−GCST90038687'

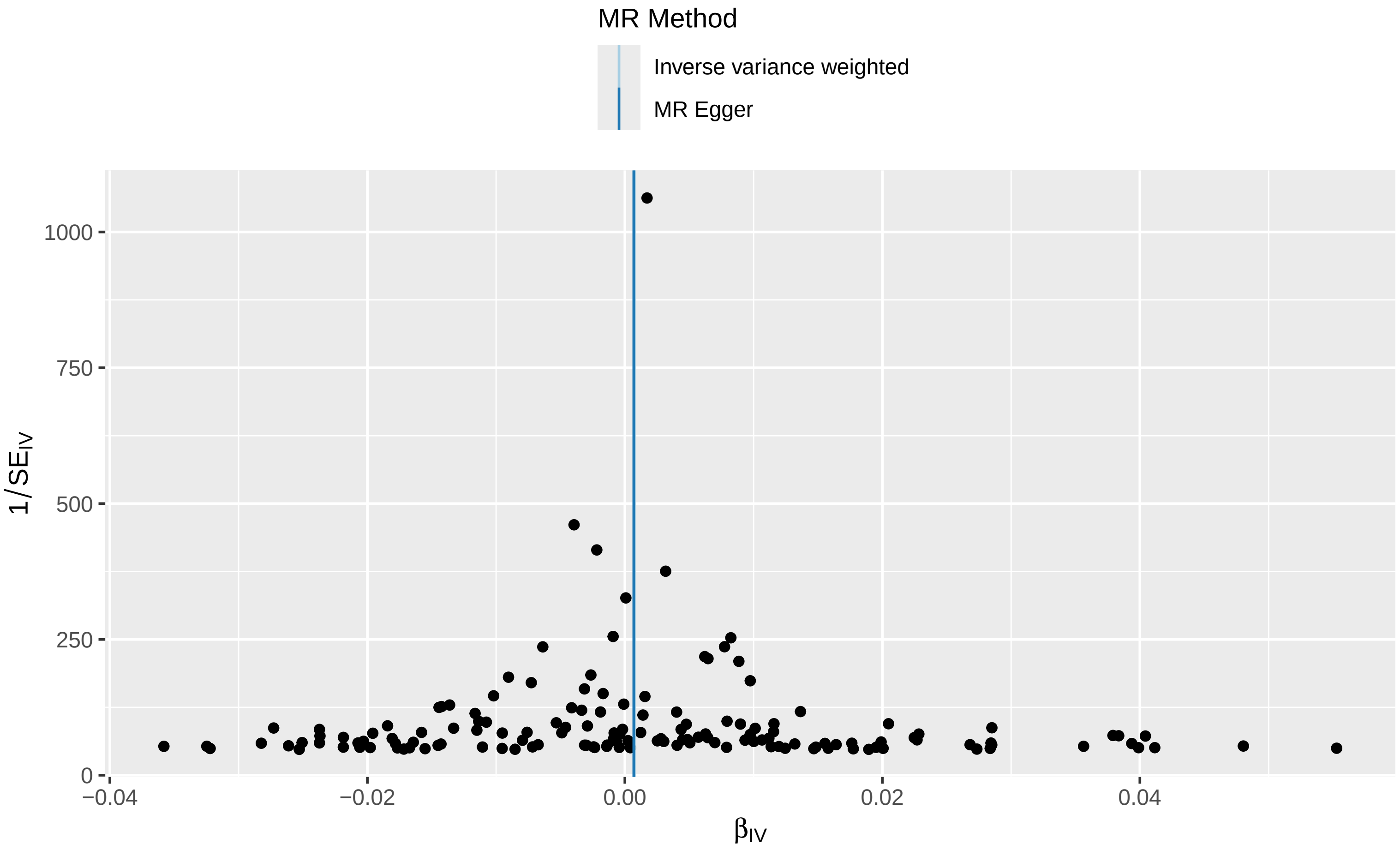
MR Method
Inverse variance weighted
MR Egger
1000
750
500
250
0
1/SE_IV
−0.04
−0.02
0.00
0.02
0.04
β_IV

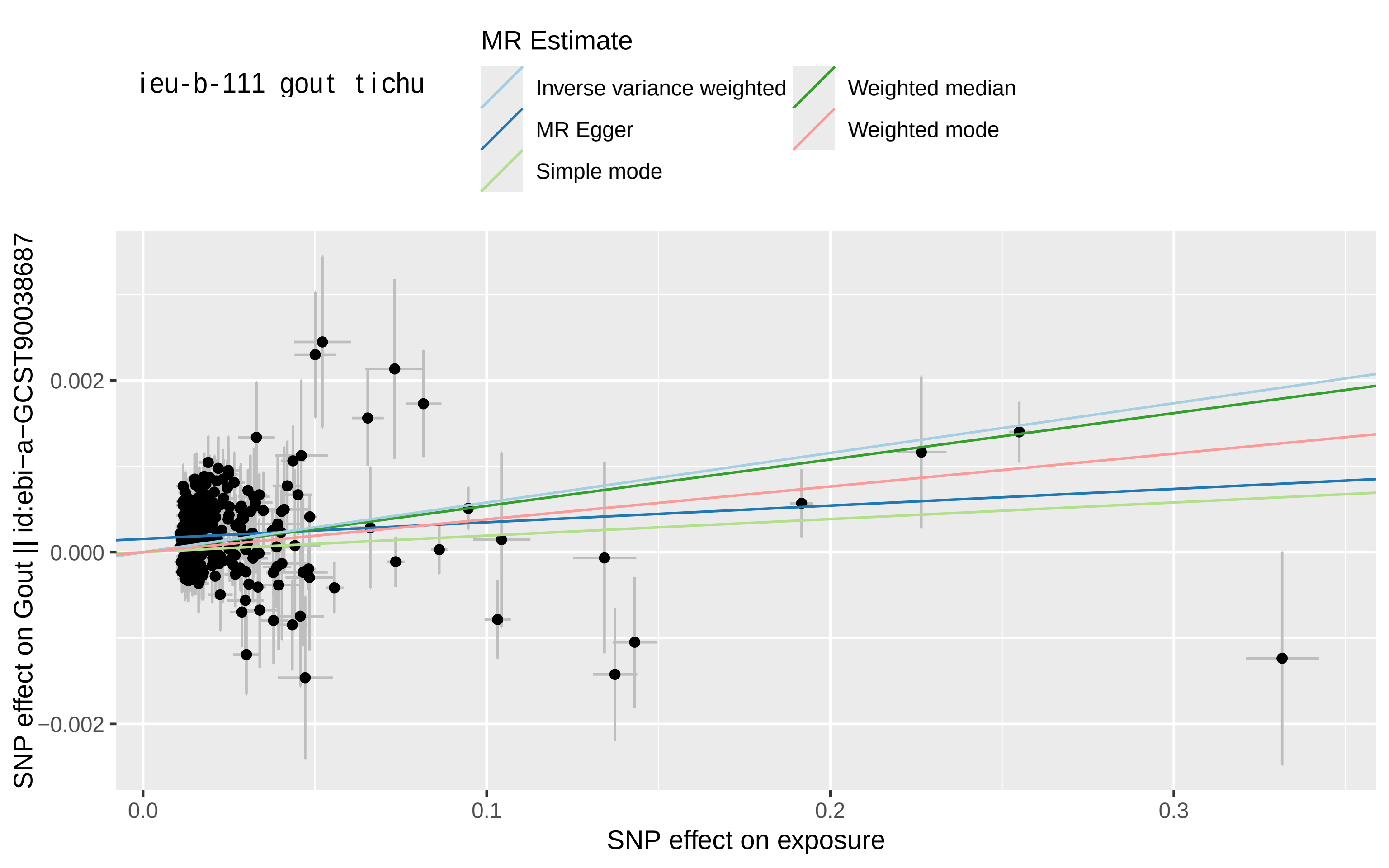

ieu-b-111_gout_tichu
MR Estimate
Inverse variance weighted
MR Egger
Simple mode
Weighted median
Weighted mode
SNP effect on Gout || id:ebi-a-GCST90038687
0.002
0.000
−0.002
0.0
0.1
0.2
0.3
SNP effect on exposure

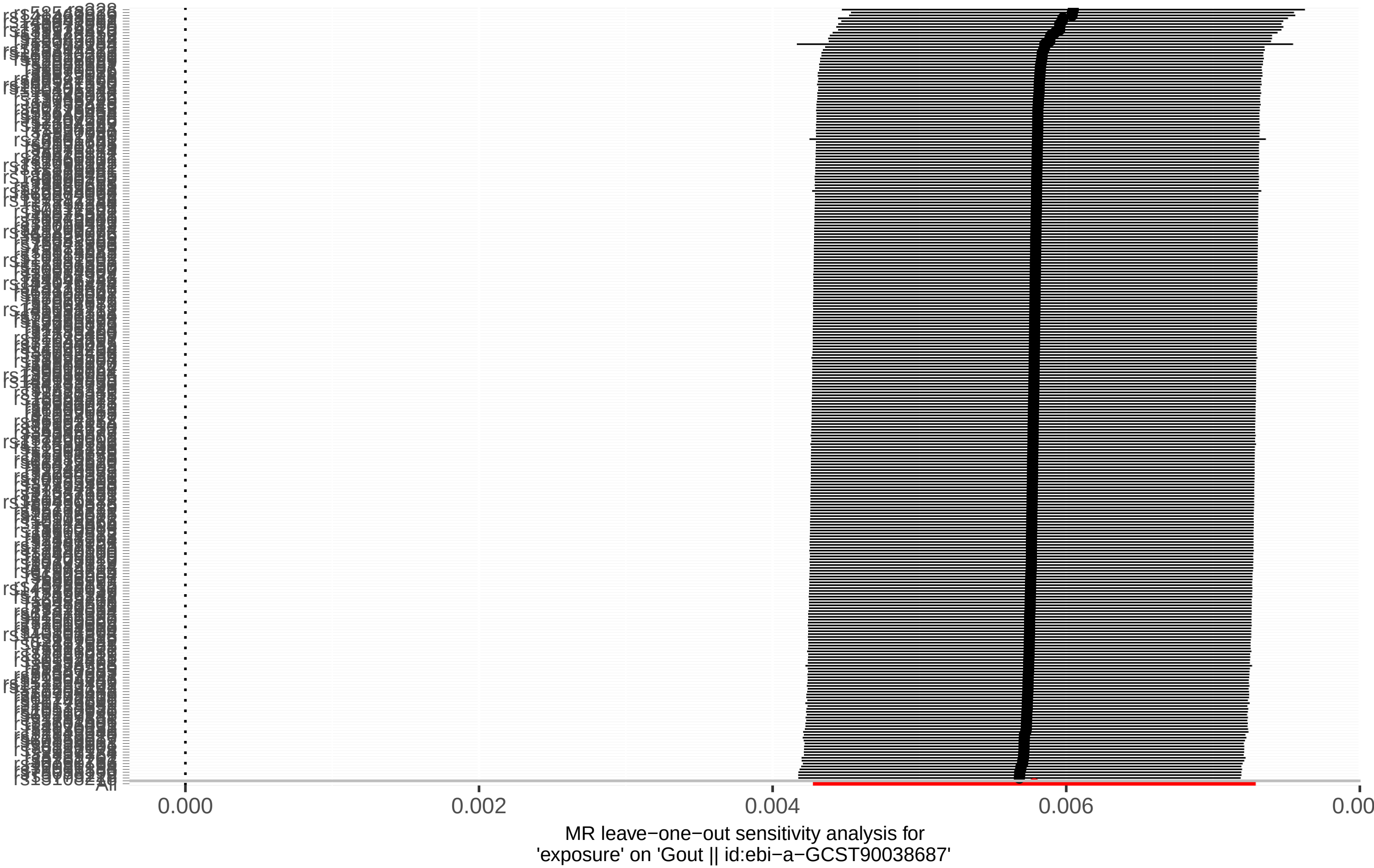

All
0.000
0.002
0.004
0.006
0.00
MR leave−one−out sensitivity analysis for
'exposure' on 'Gout || id:ebi−a−GCST90038687'

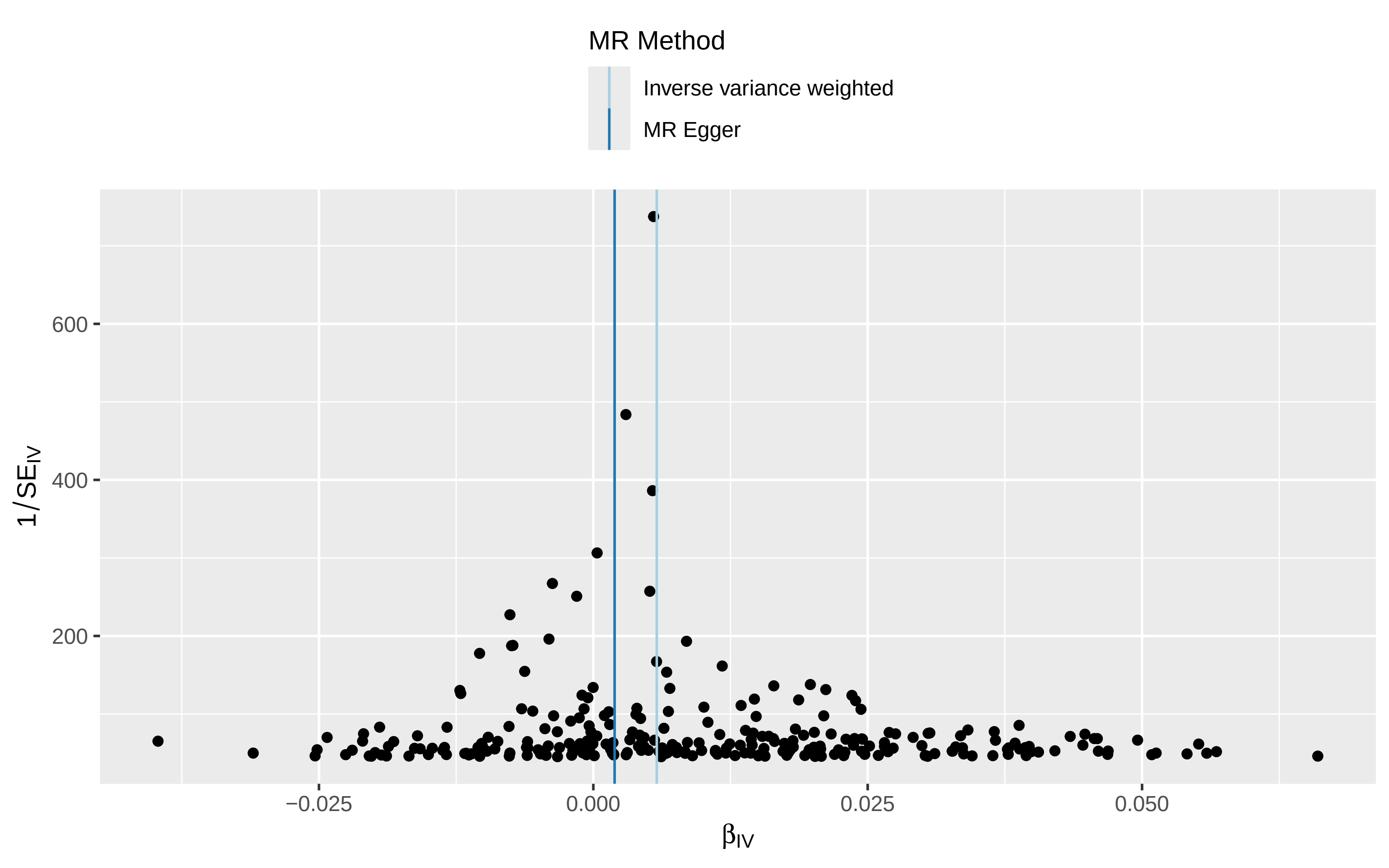
MR Method
Inverse variance weighted
MR Egger
1/SE_IV
600
400
200
−0.025
0.000
0.025
0.050
β_IV
