# Supplementary material for: Decoding the lipid etiology of atherogenic index of plasma and gout: establishing the causal role of triglycerides through NHANES, Mendelian randomization, and network pharmacology
Source: Cardiovasc Diabetol Endocrinol Rep. 2026 Jul 13;12:40. doi: 10.1186/s40842-026-00309-0 (PMC13362044; doi:10.1186/s40842-026-00309-0)
Supplement: Supplementary file 6 — Supplementary Material 6 [file 40842_2026_309_MOESM6_ESM.zip › 1-40842_2026_309_MOESM6_ESM.pdf]

UKB
109_GOUT

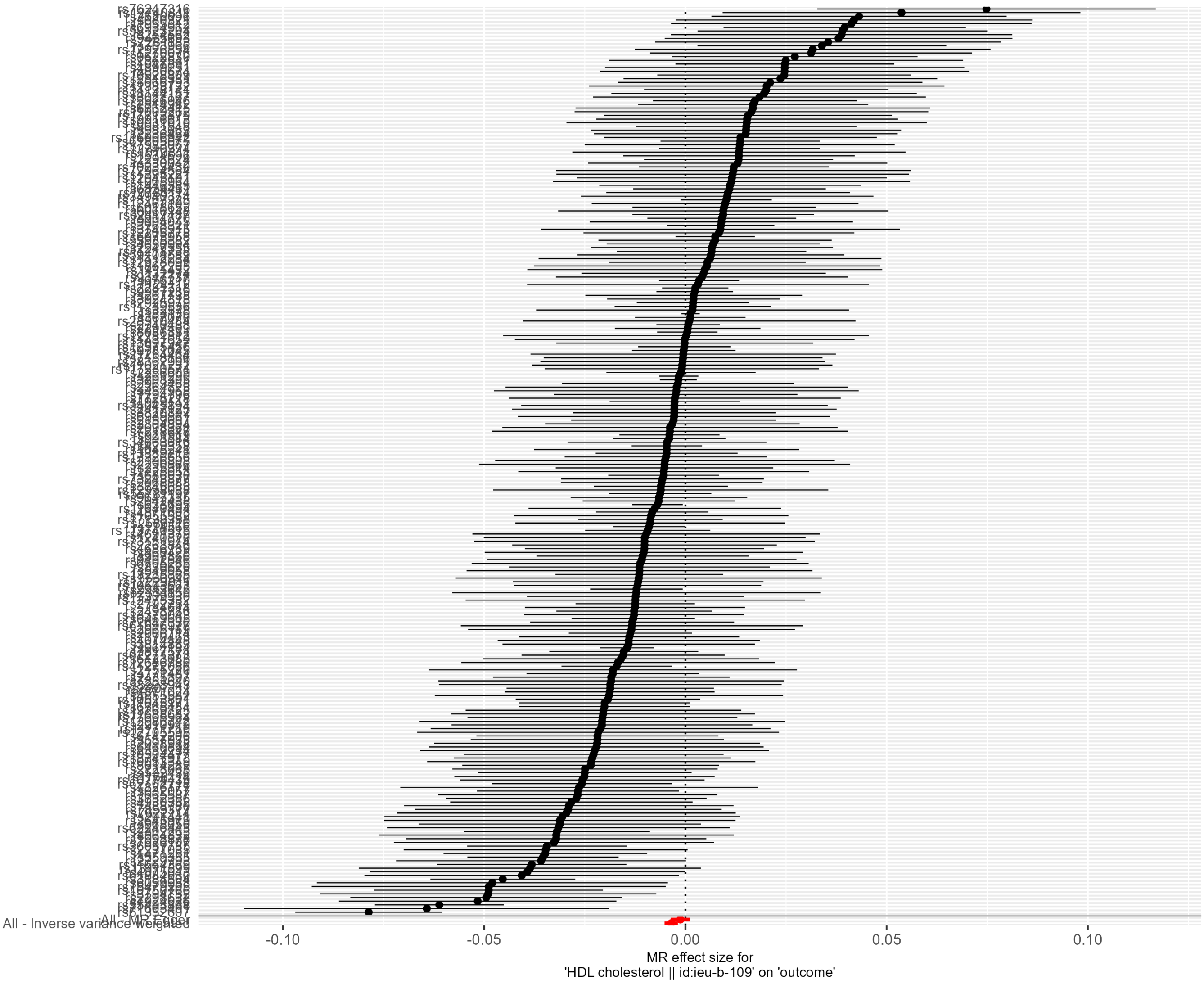
rs76247316
All - Inverse variance weighted
-0.10
-0.05
0.00
0.05
0.10
MR effect size for
'HDL cholesterol || id:ieu-b-109' on 'outcome'

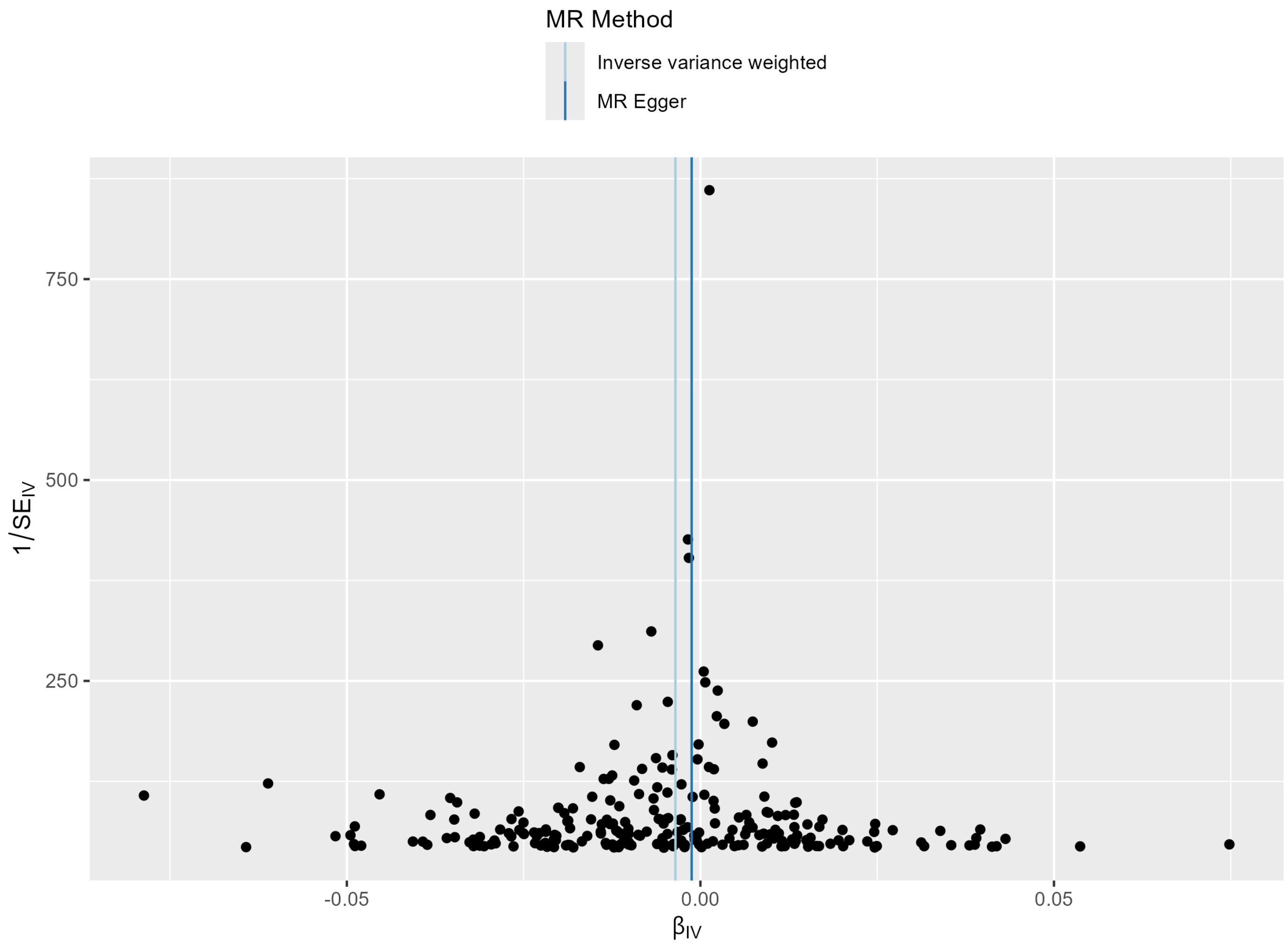
MR Method
Inverse variance weighted
MR Egger
750
500
250
1/SE_IV
-0.05
0.00
0.05
β_IV

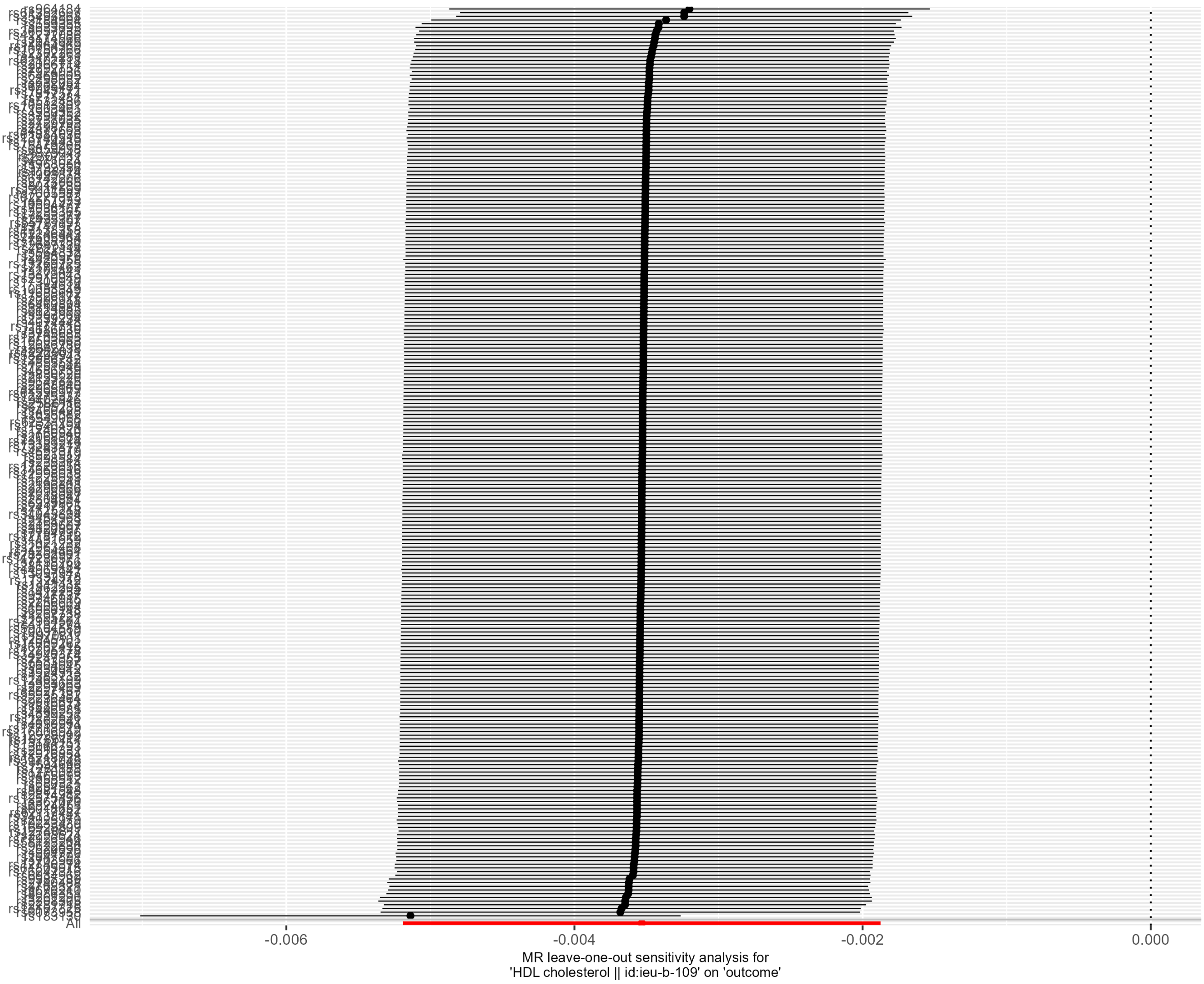
All
-0.006
-0.004
-0.002
0.000
MR leave-one-out sensitivity analysis for
'HDL cholesterol || id:ieu-b-109' on 'outcome'

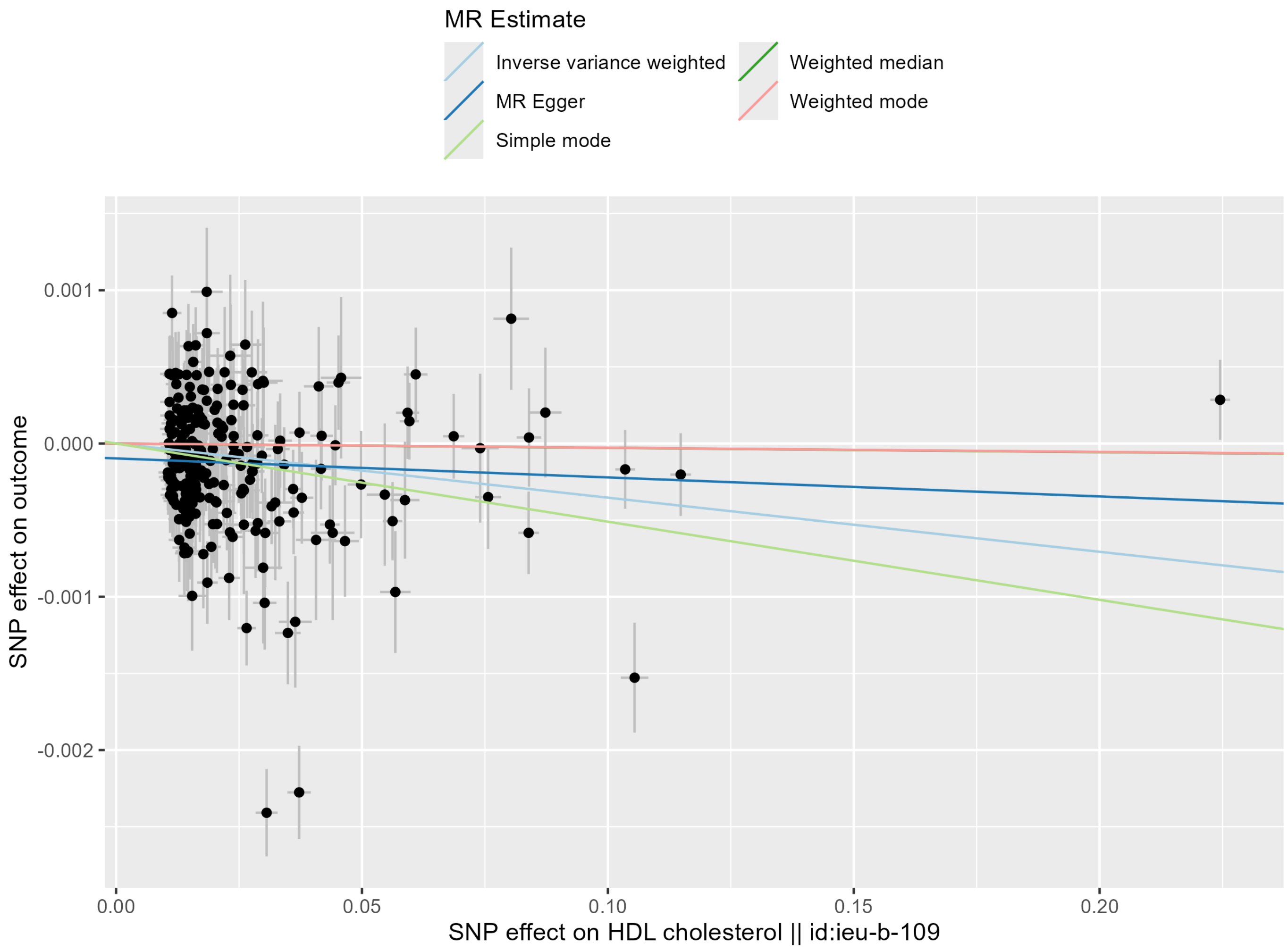
MR Estimate
Inverse variance weighted
MR Egger
Simple mode
Weighted median
Weighted mode
0.001
0.000
-0.001
-0.002
SNP effect on outcome
0.00
0.05
0.10
0.15
0.20
SNP effect on HDL cholesterol || id:ieu-b-109

110_gout

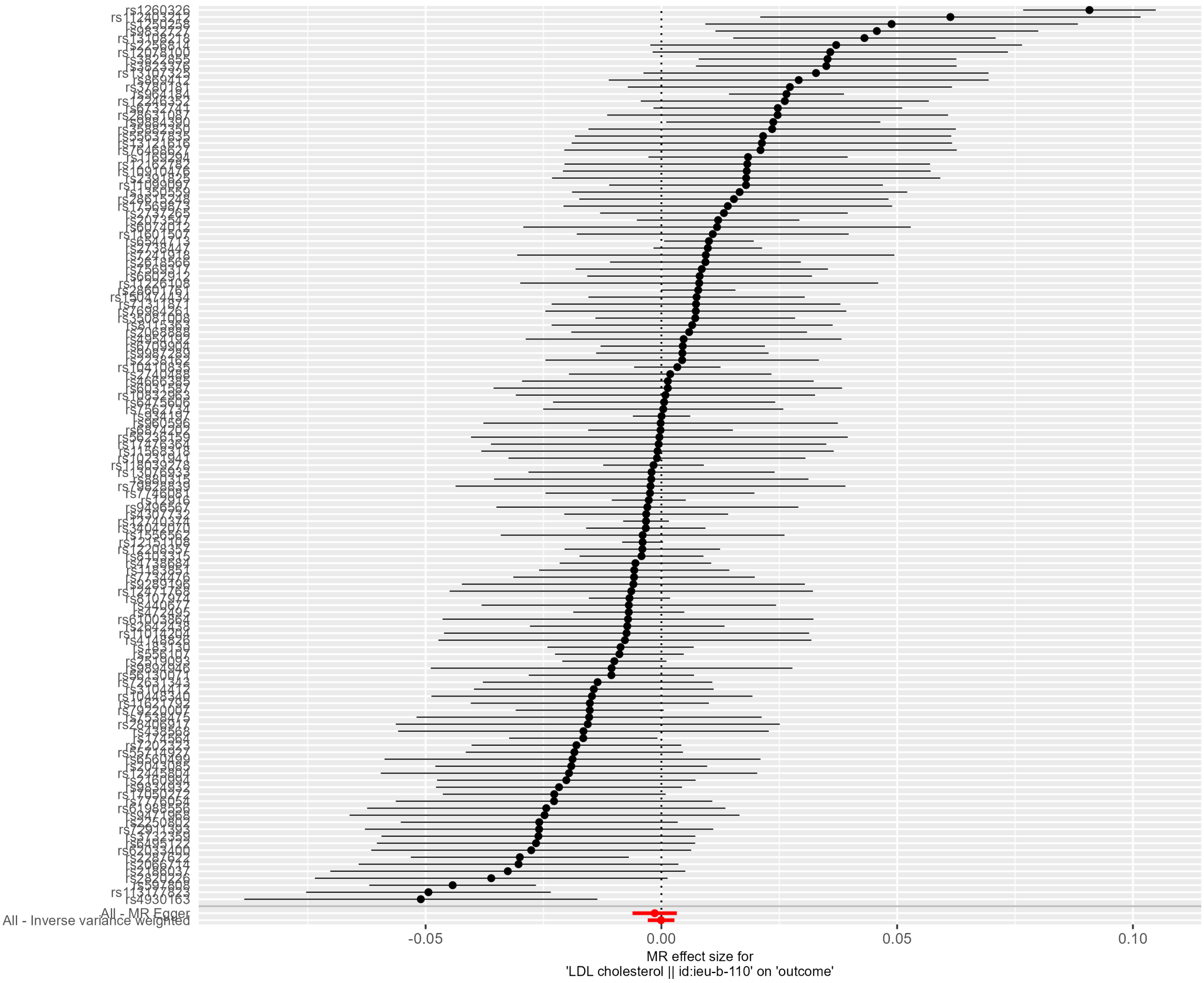
rs1260326
rs4930163
All - MR Egger
All - Inverse variance weighted
-0.05
0.00
0.05
0.10
MR effect size for
'LDL cholesterol || id:ieu-b-110' on 'outcome'

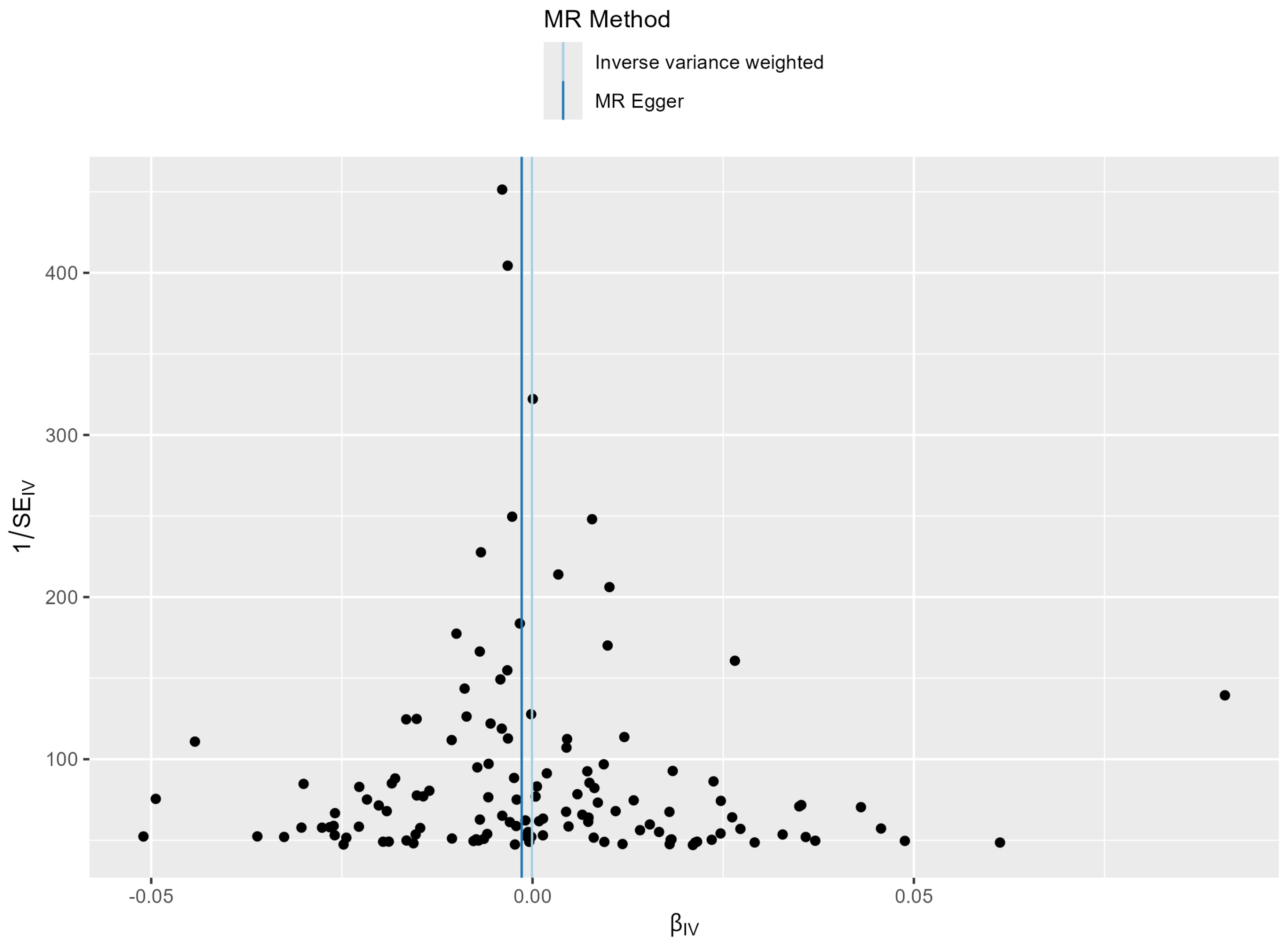

MR Method
Inverse variance weighted
MR Egger
400
300
200
100
1/SE_IV
-0.05
0.00
0.05
β_IV

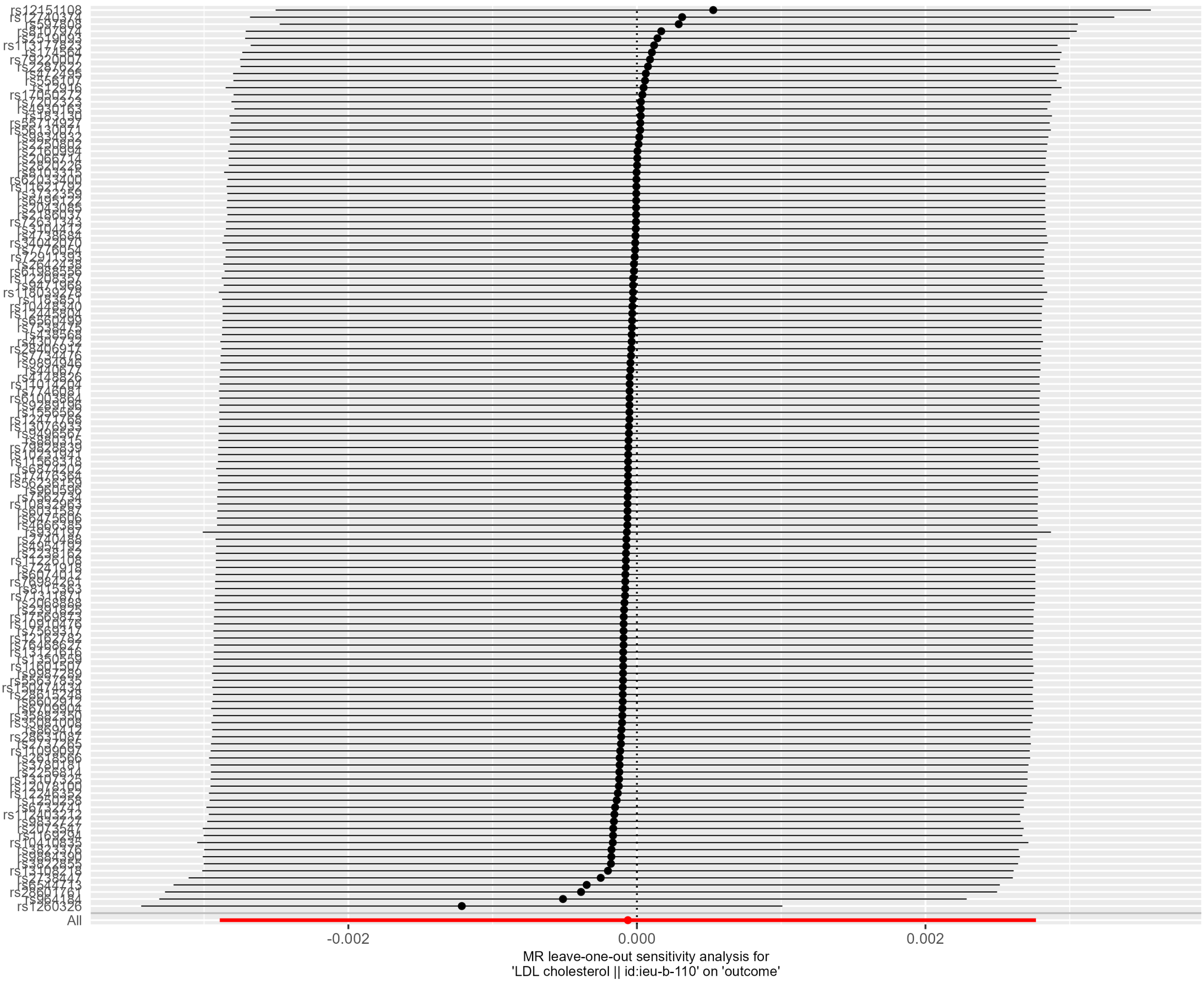
rs12151108
rs1260326
All
-0.002
0.000
0.002
MR leave-one-out sensitivity analysis for
'LDL cholesterol || id:ieu-b-110' on 'outcome'

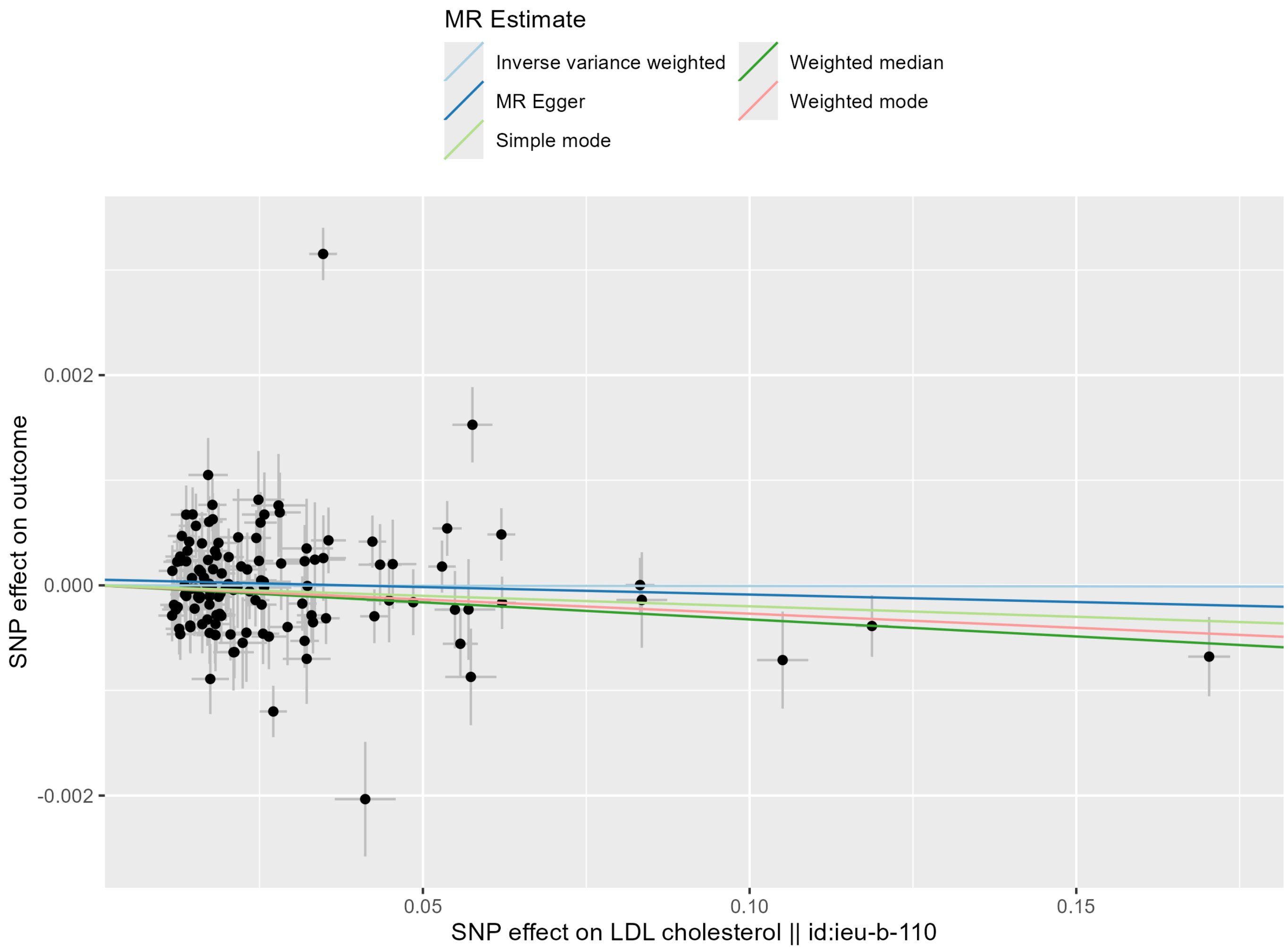
MR Estimate
Inverse variance weighted
MR Egger
Simple mode
Weighted median
Weighted mode
0.002
0.000
-0.002
SNP effect on outcome
0.05
0.10
0.15
SNP effect on LDL cholesterol || id:ieu-b-110

111_gout

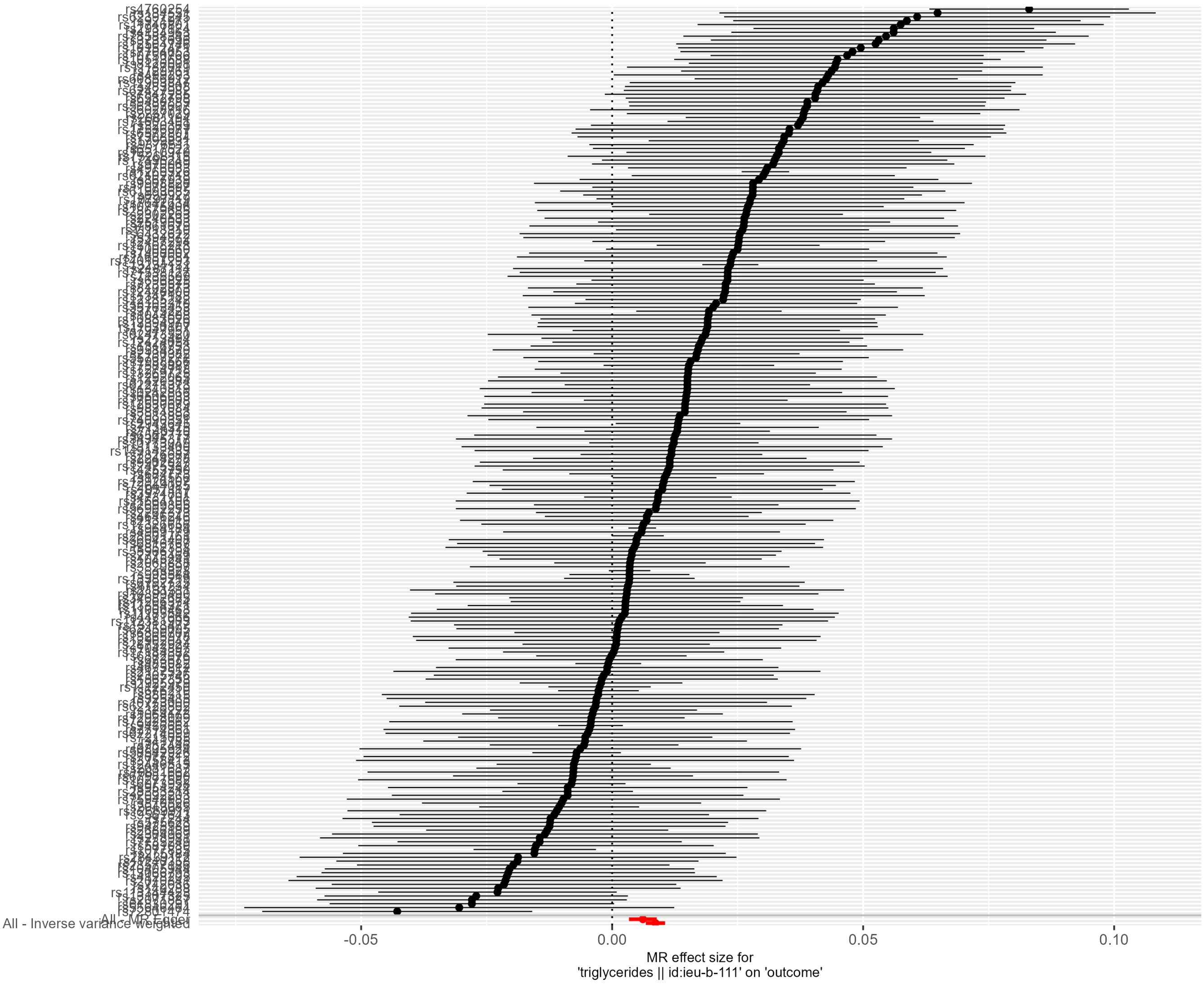

rs4760254
All - MR Egger
All - Inverse variance weighted
-0.05
0.00
0.05
0.10
MR effect size for
'triglycerides || id:ieu-b-111' on 'outcome'

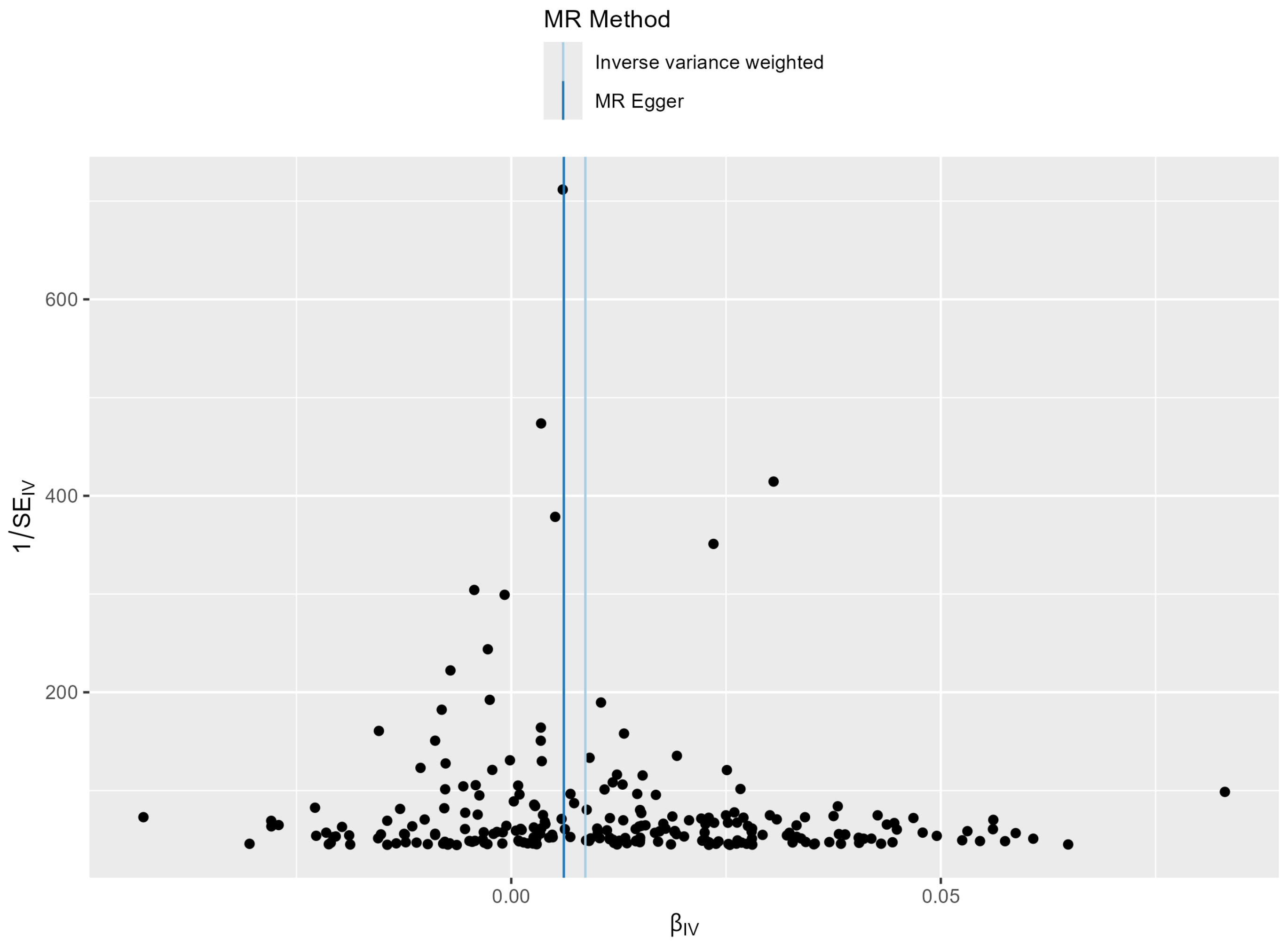
MR Method
Inverse variance weighted
MR Egger
600
400
200
1/SE_IV
0.00
0.05
β_IV

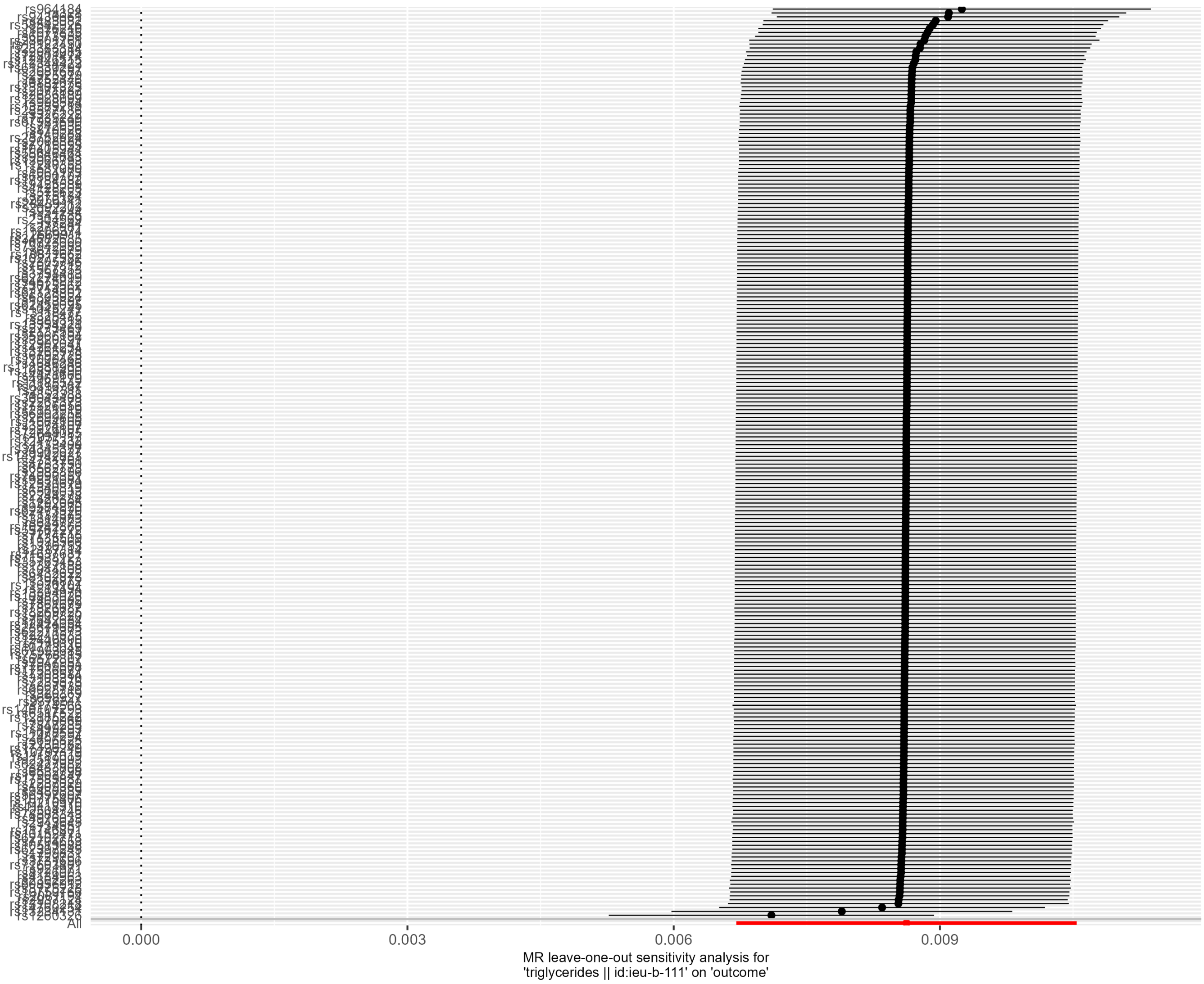
rs964184
All
0.000
0.003
0.006
0.009
MR leave-one-out sensitivity analysis for
'triglycerides || id:ieu-b-111' on 'outcome'

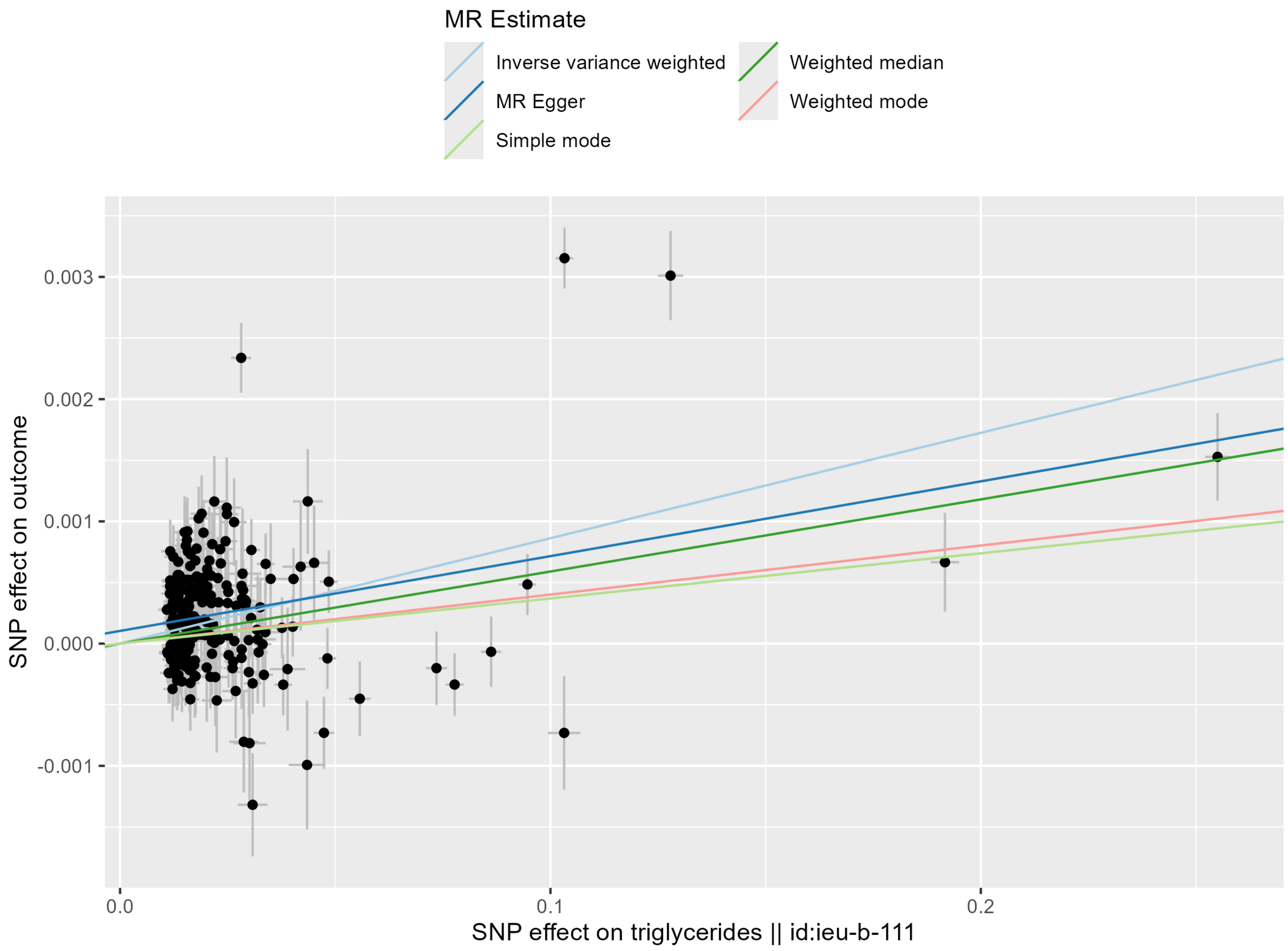
MR Estimate
Inverse variance weighted
MR Egger
Simple mode
Weighted median
Weighted mode
0.003
0.002
0.001
0.000
-0.001
SNP effect on outcome
0.0
0.1
0.2
SNP effect on triglycerides || id:ieu-b-111

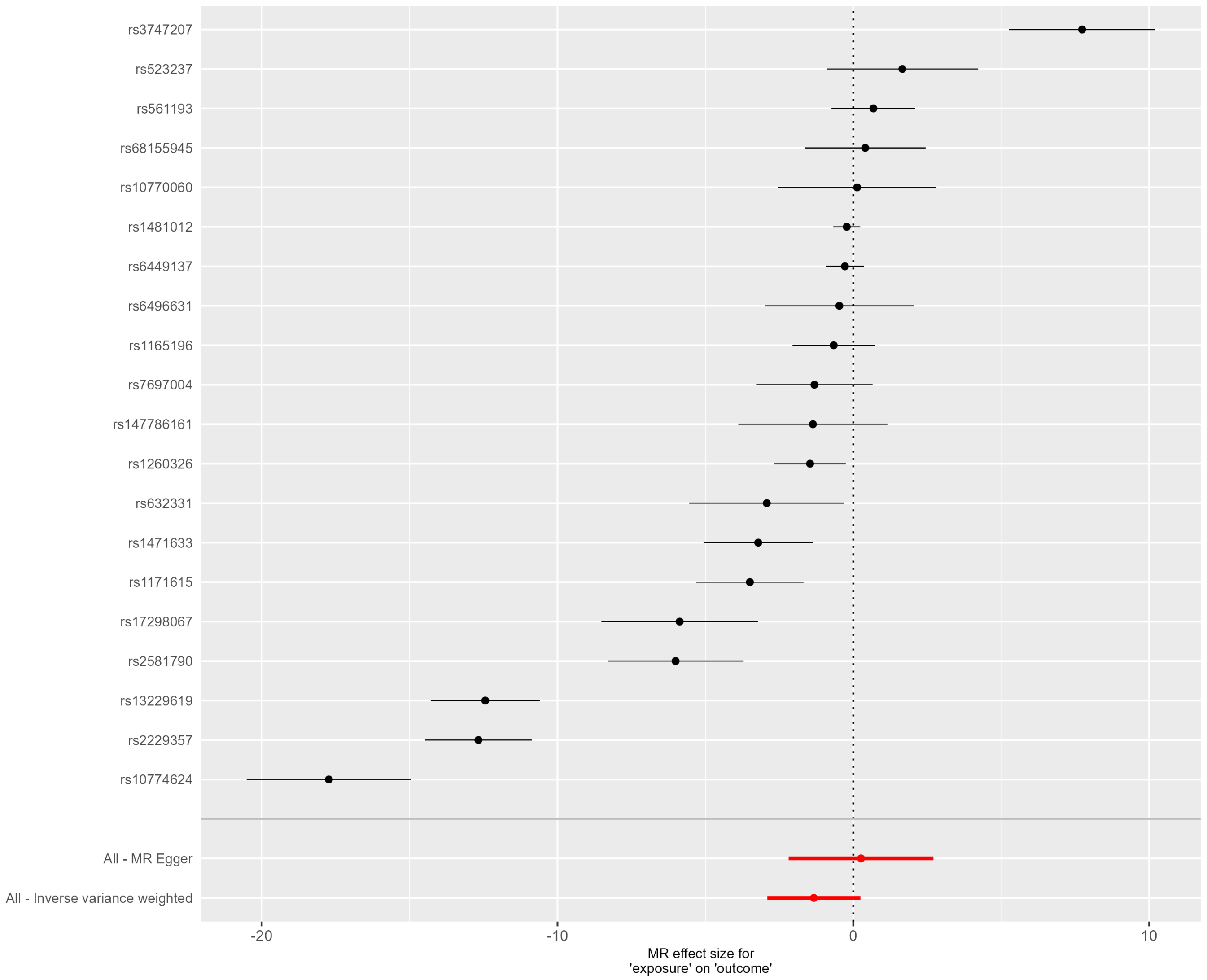
rs3747207
rs523237
rs561193
rs68155945
rs10770060
rs1481012
rs6449137
rs6496631
rs1165196
rs7697004
rs147786161
rs1260326
rs632331
rs1471633
rs1171615
rs17298067
rs2581790
rs13229619
rs2229357
rs10774624
All - MR Egger
All - Inverse variance weighted
-20
-10
0
10
MR effect size for
'exposure' on 'outcome'

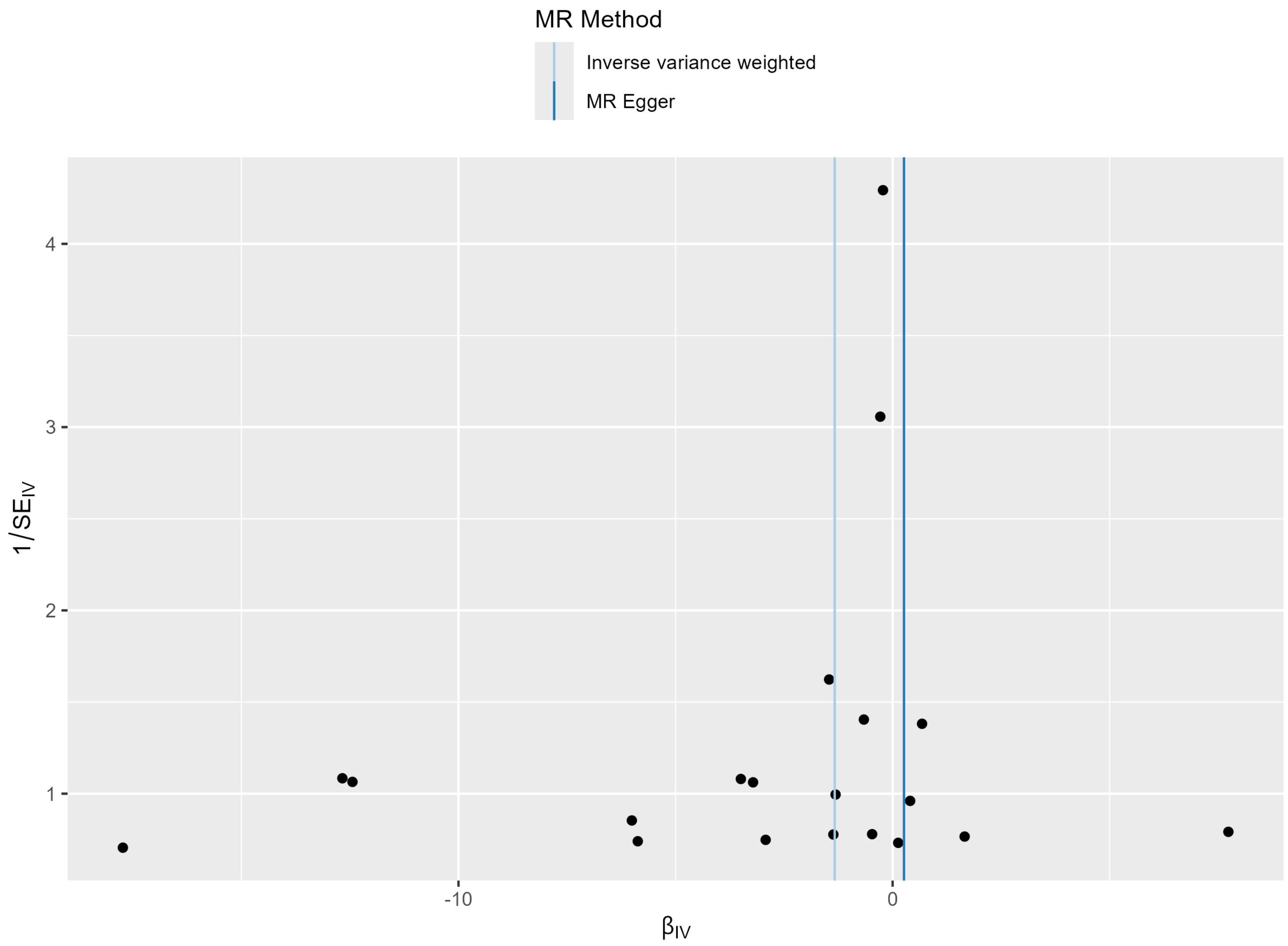

MR Method
Inverse variance weighted
MR Egger
4
3
2
1
1/SE_IV
-10
0
β_IV

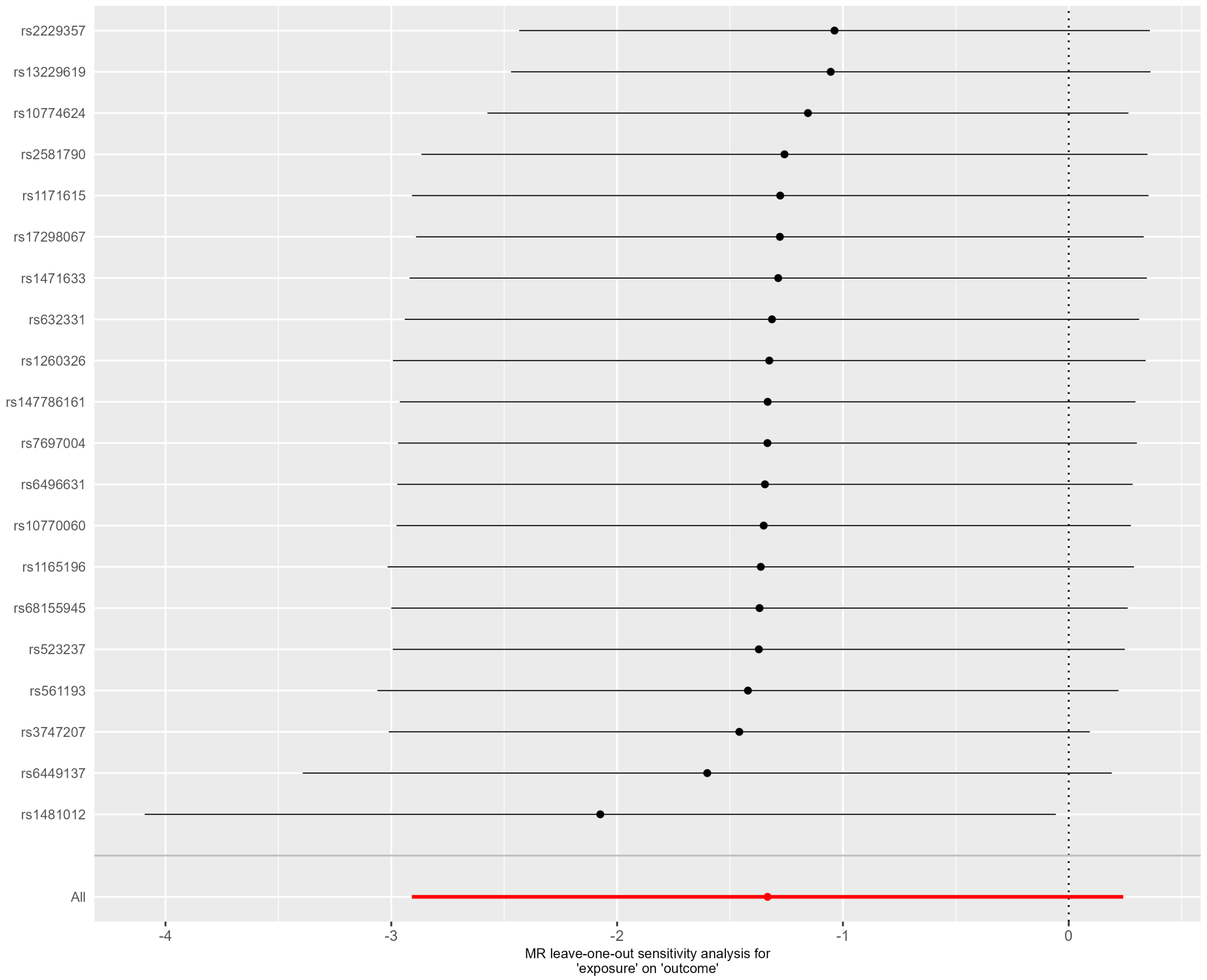

rs2229357
rs13229619
rs10774624
rs2581790
rs1171615
rs17298067
rs1471633
rs632331
rs1260326
rs147786161
rs7697004
rs6496631
rs10770060
rs1165196
rs68155945
rs523237
rs561193
rs3747207
rs6449137
rs1481012
All
-4
-3
-2
-1
0
MR leave-one-out sensitivity analysis for
'exposure' on 'outcome'

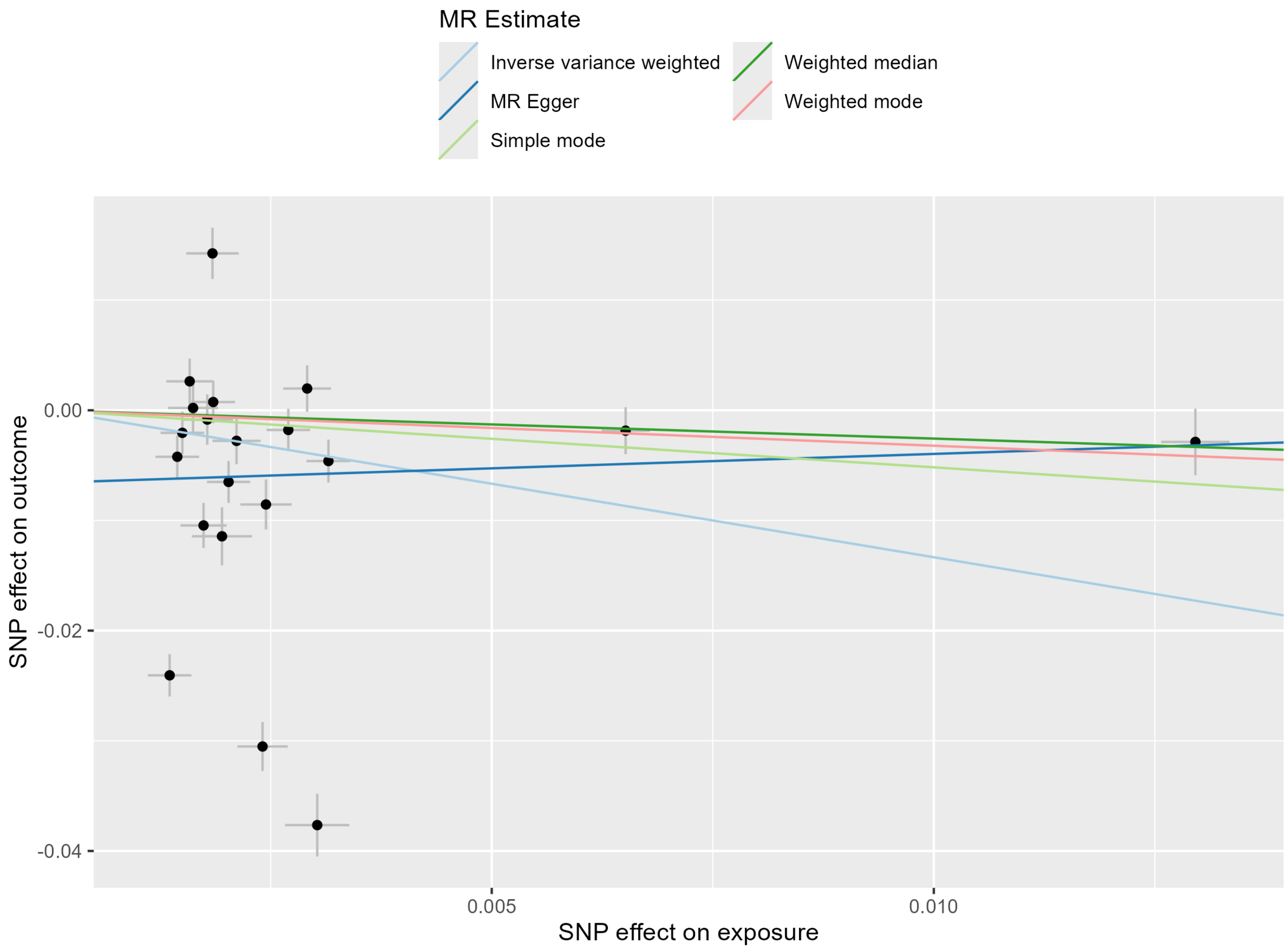

MR Estimate
Inverse variance weighted
MR Egger
Simple mode
Weighted median
Weighted mode
0.00
-0.02
-0.04
SNP effect on outcome
0.005
0.010
SNP effect on exposure

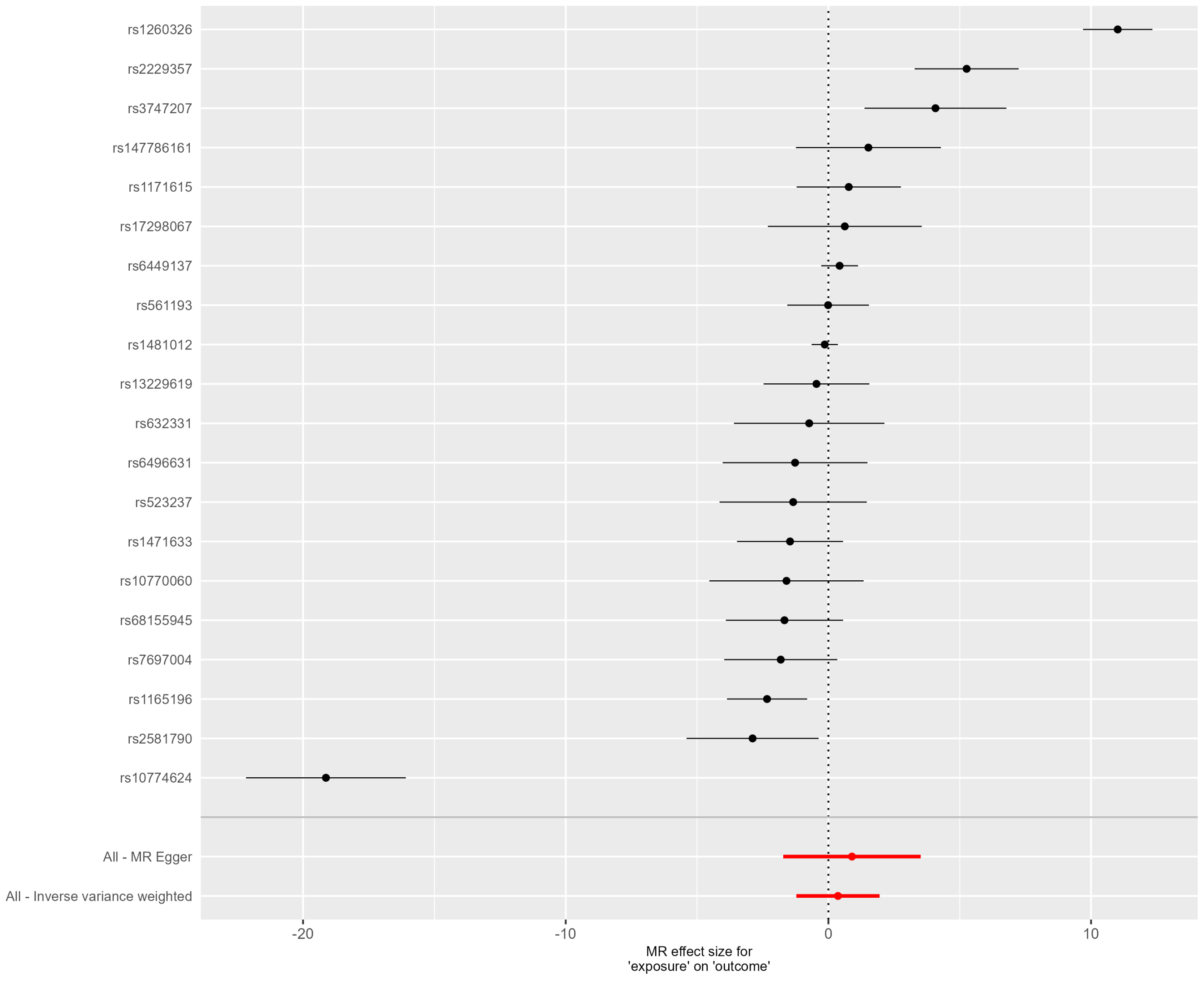
rs1260326
rs2229357
rs3747207
rs147786161
rs1171615
rs17298067
rs6449137
rs561193
rs1481012
rs13229619
rs632331
rs6496631
rs523237
rs1471633
rs10770060
rs68155945
rs7697004
rs1165196
rs2581790
rs10774624
All - MR Egger
All - Inverse variance weighted
-20
-10
0
10
MR effect size for
'exposure' on 'outcome'

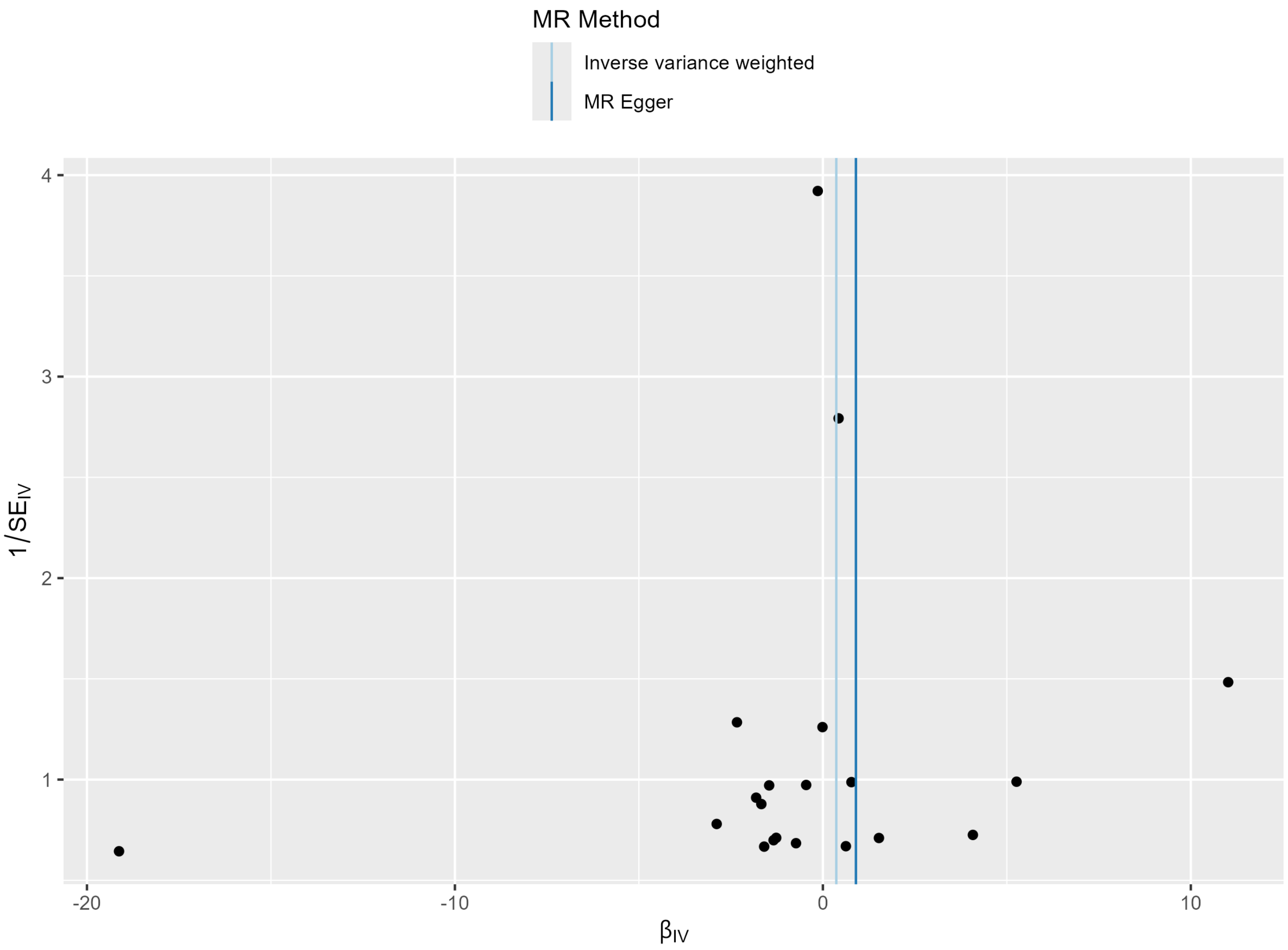

MR Method
Inverse variance weighted
MR Egger
4
3
2
1
1/SE$_{IV}$
-20
-10
0
10
β$_{IV}$

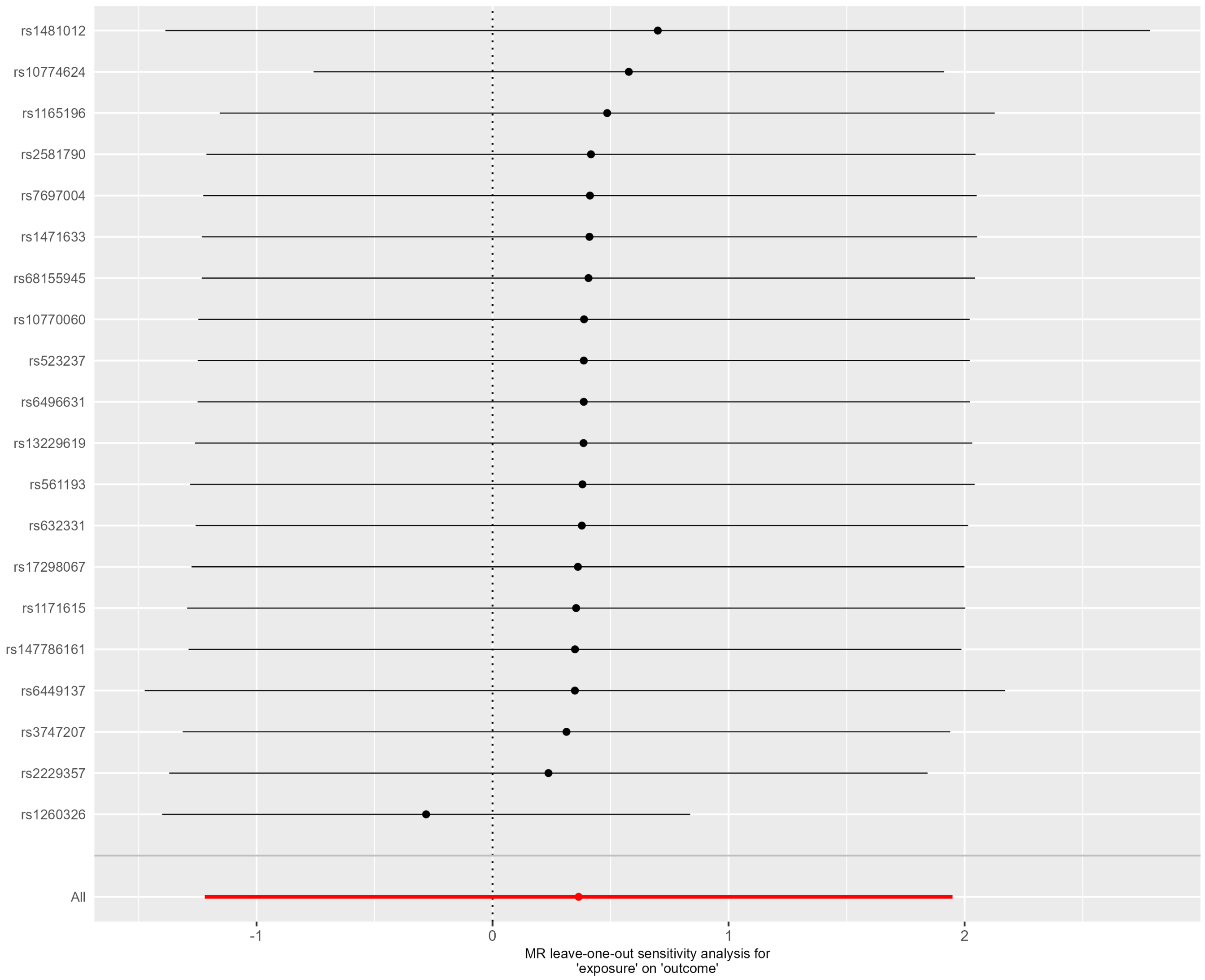
rs1481012
rs10774624
rs1165196
rs2581790
rs7697004
rs1471633
rs68155945
rs10770060
rs523237
rs6496631
rs13229619
rs561193
rs632331
rs17298067
rs1171615
rs147786161
rs6449137
rs3747207
rs2229357
rs1260326
All
-1
0
1
2
MR leave-one-out sensitivity analysis for
'exposure' on 'outcome'

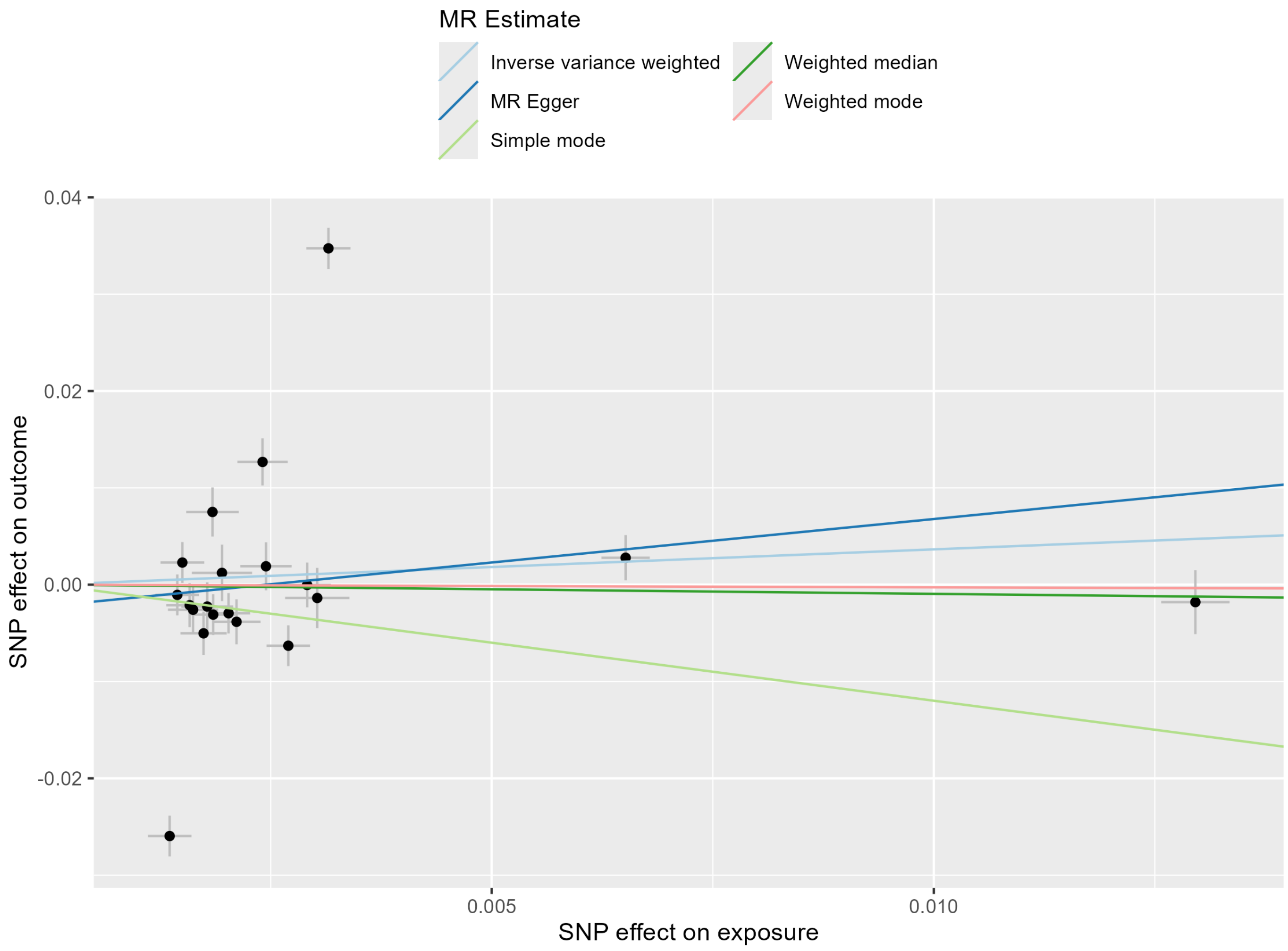
MR Estimate
Inverse variance weighted
MR Egger
Simple mode
Weighted median
Weighted mode
0.04
0.02
0.00
-0.02
SNP effect on outcome
0.005
0.010
SNP effect on exposure

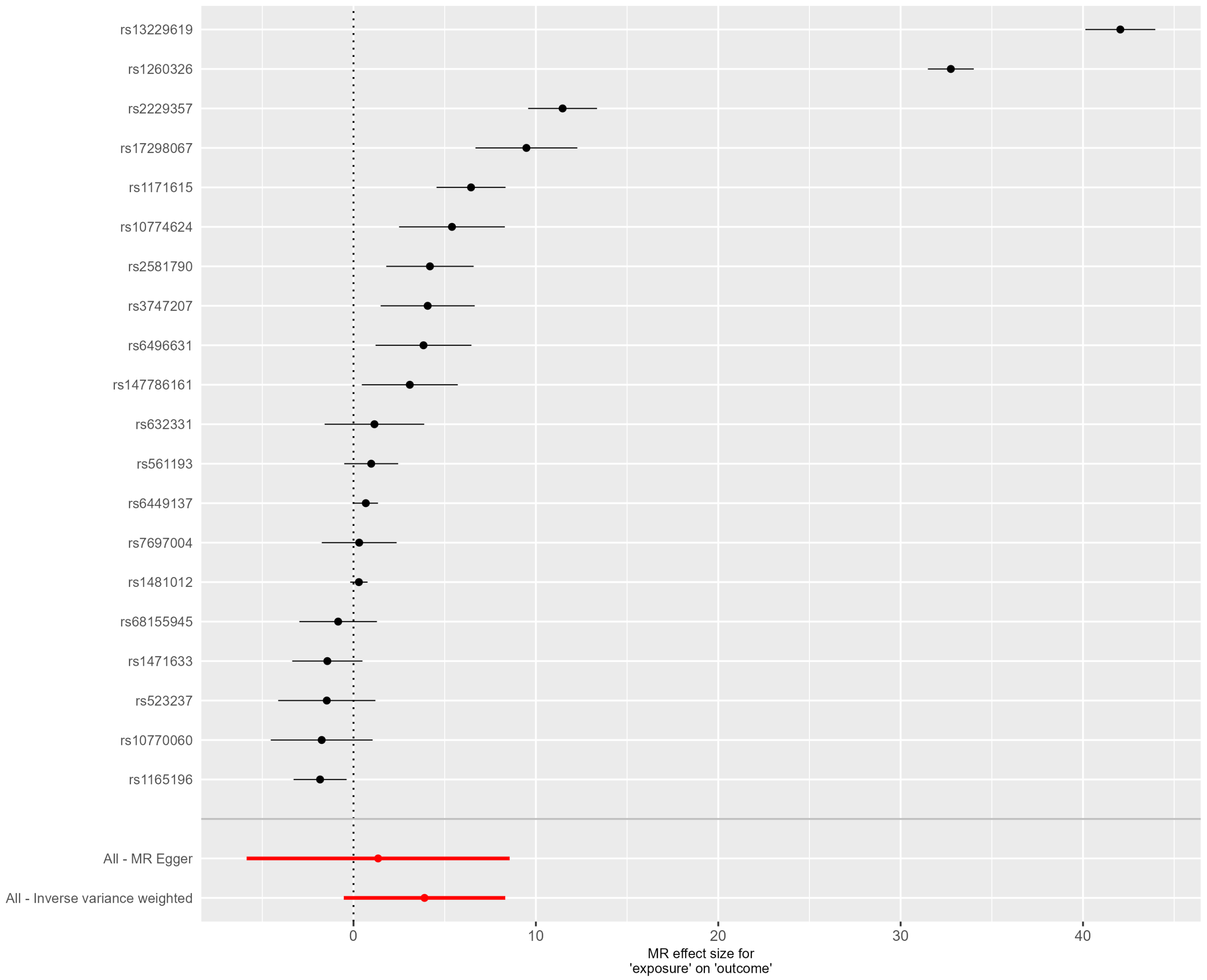
rs13229619
rs1260326
rs2229357
rs17298067
rs1171615
rs10774624
rs2581790
rs3747207
rs6496631
rs147786161
rs632331
rs561193
rs6449137
rs7697004
rs1481012
rs68155945
rs1471633
rs523237
rs10770060
rs1165196
All - MR Egger
All - Inverse variance weighted
0
10
20
30
40
MR effect size for
'exposure' on 'outcome'

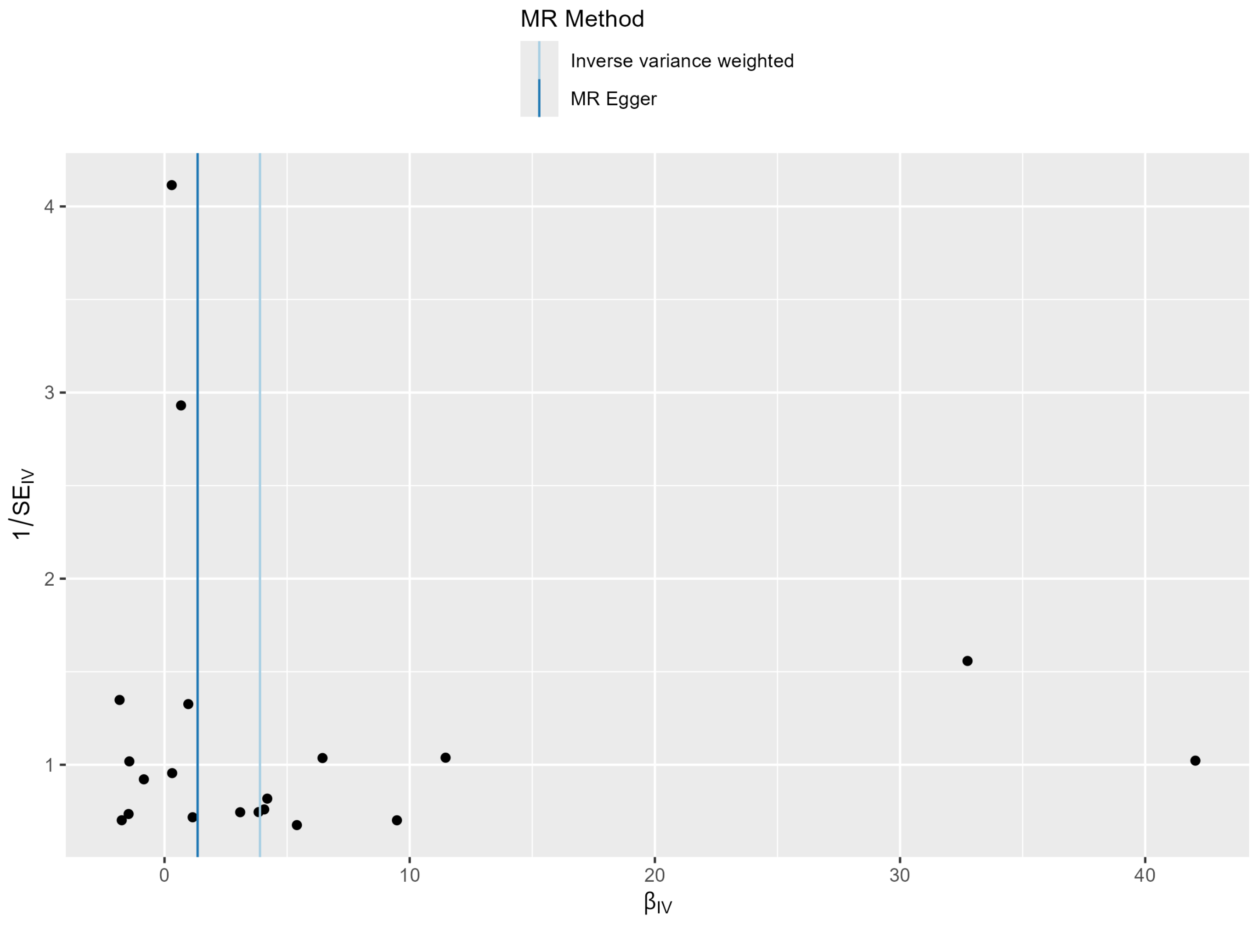
MR Method
Inverse variance weighted
MR Egger
1/SE_IV
β_IV
0
10
20
30
40
1
2
3
4

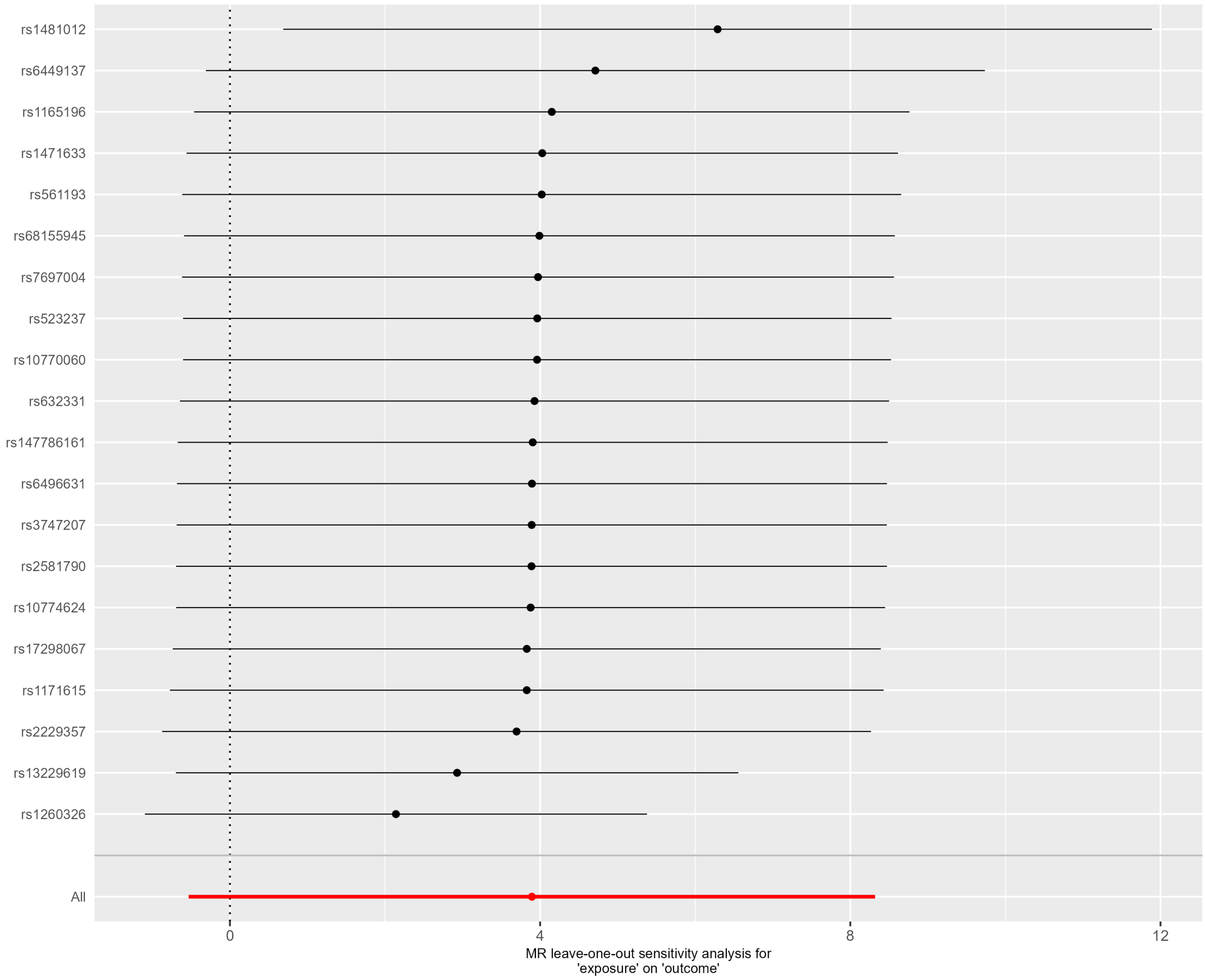
rs1481012
rs6449137
rs1165196
rs1471633
rs561193
rs68155945
rs7697004
rs523237
rs10770060
rs632331
rs147786161
rs6496631
rs3747207
rs2581790
rs10774624
rs17298067
rs1171615
rs2229357
rs13229619
rs1260326
All
0
4
8
12
MR leave-one-out sensitivity analysis for
'exposure' on 'outcome'

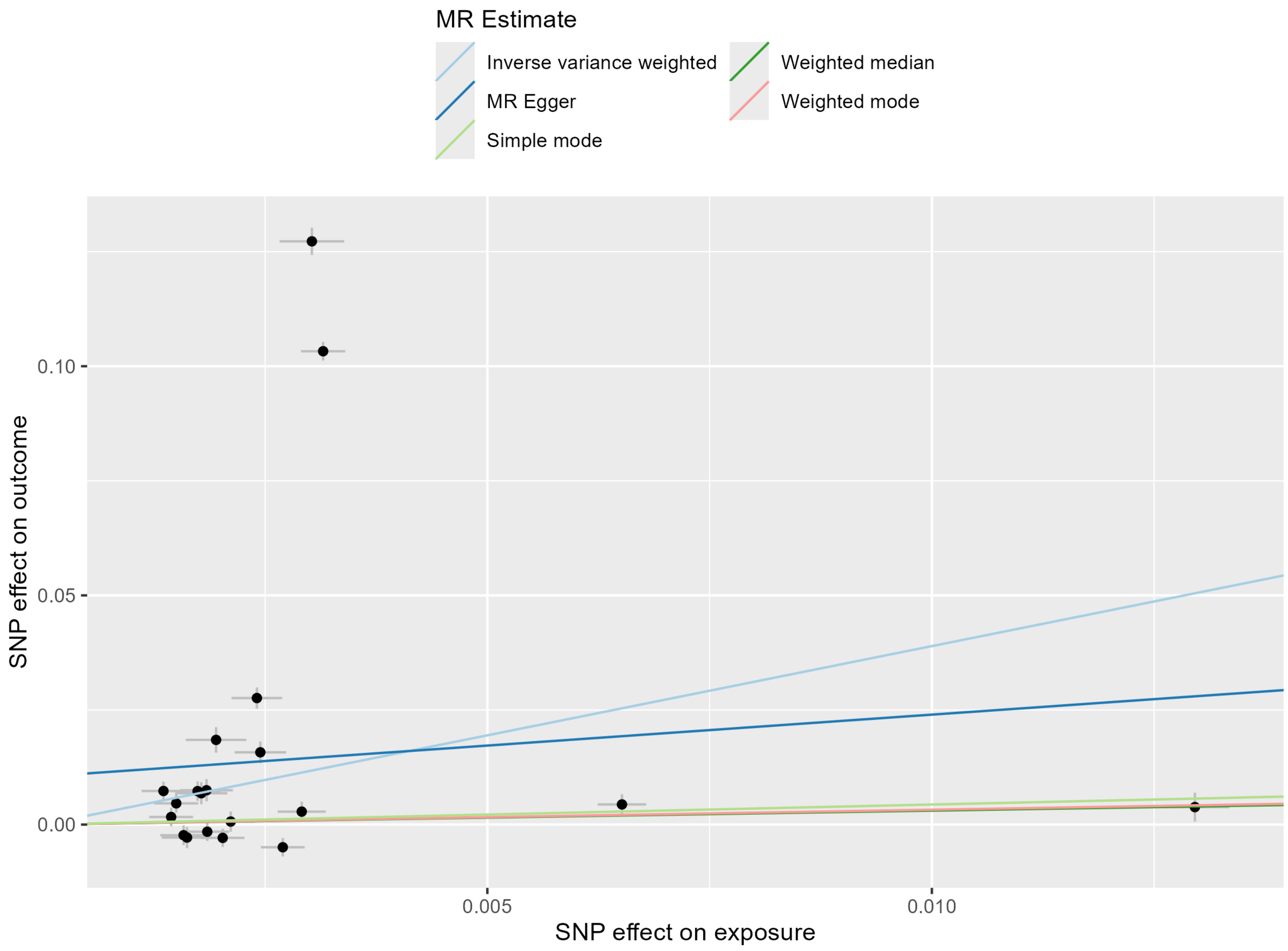
MR Estimate
Inverse variance weighted
MR Egger
Simple mode
Weighted median
Weighted mode
0.10
0.05
0.00
SNP effect on outcome
0.005
0.010
SNP effect on exposure
