# Supplementary material for: Decoding the lipid etiology of atherogenic index of plasma and gout: establishing the causal role of triglycerides through NHANES, Mendelian randomization, and network pharmacology
Source: Cardiovasc Diabetol Endocrinol Rep. 2026 Jul 13;12:40. doi: 10.1186/s40842-026-00309-0 (PMC13362044; doi:10.1186/s40842-026-00309-0)
Supplement: Supplementary file 7 — Supplementary Material 7 [file 40842_2026_309_MOESM7_ESM.pdf]

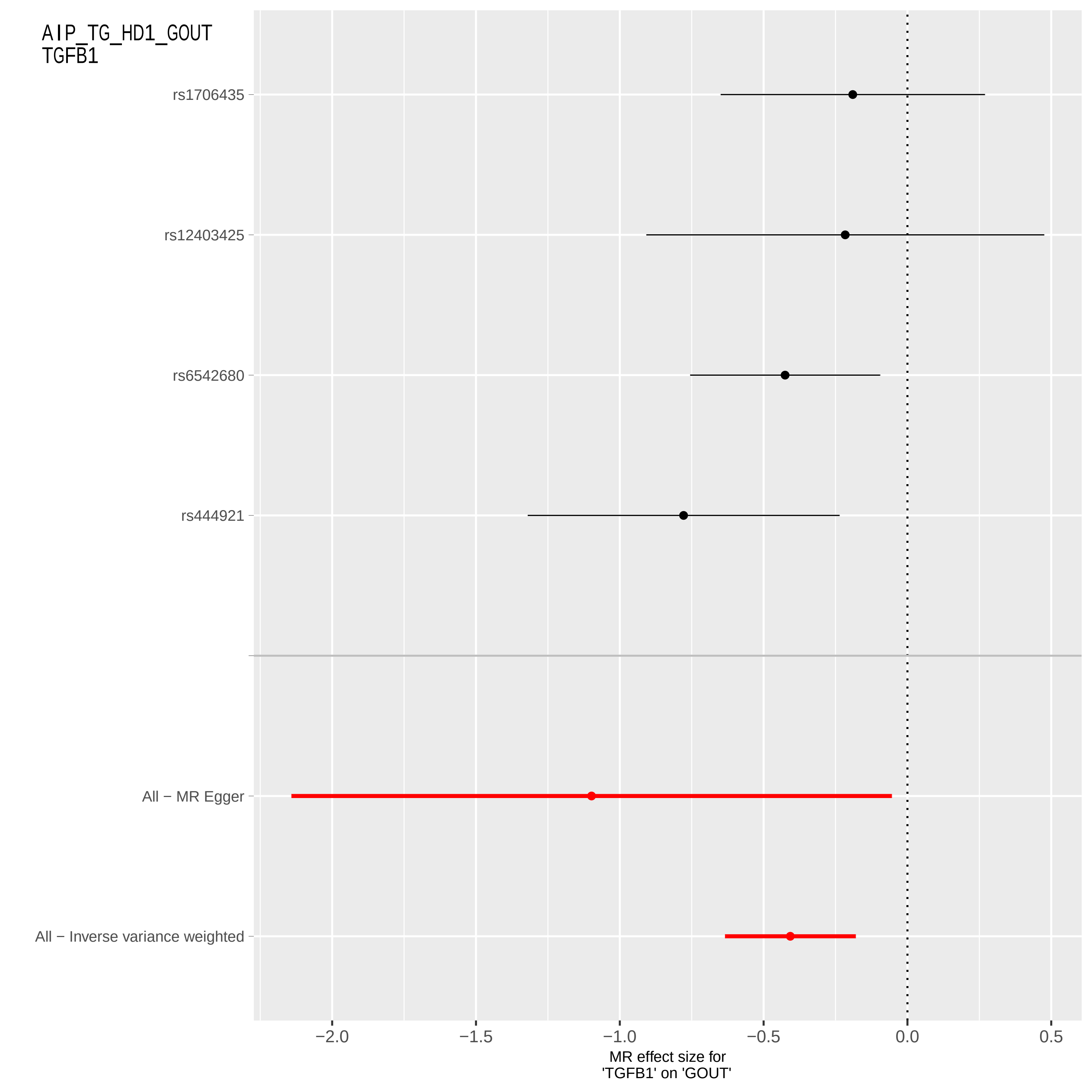
AIP_TG_HD1_GOUT
TGFB1
rs1706435
rs12403425
rs6542680
rs444921
All − MR Egger
All − Inverse variance weighted
−2.0
−1.5
−1.0
−0.5
0.0
0.5
MR effect size for
'TGFB1' on 'GOUT'

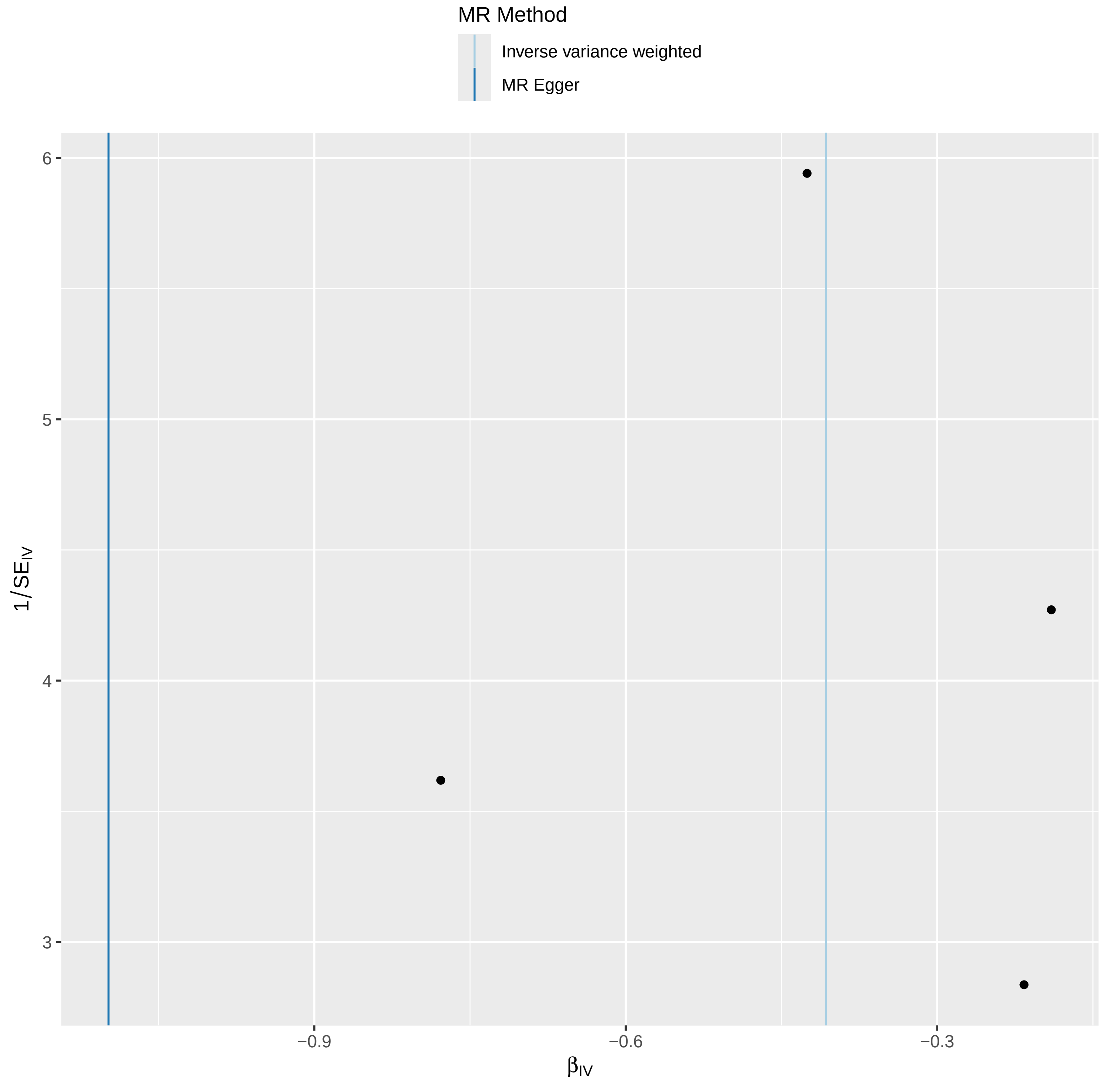
MR Method
Inverse variance weighted
MR Egger
6
5
4
3
1/SE$_{IV}$
−0.9
−0.6
−0.3
β$_{IV}$

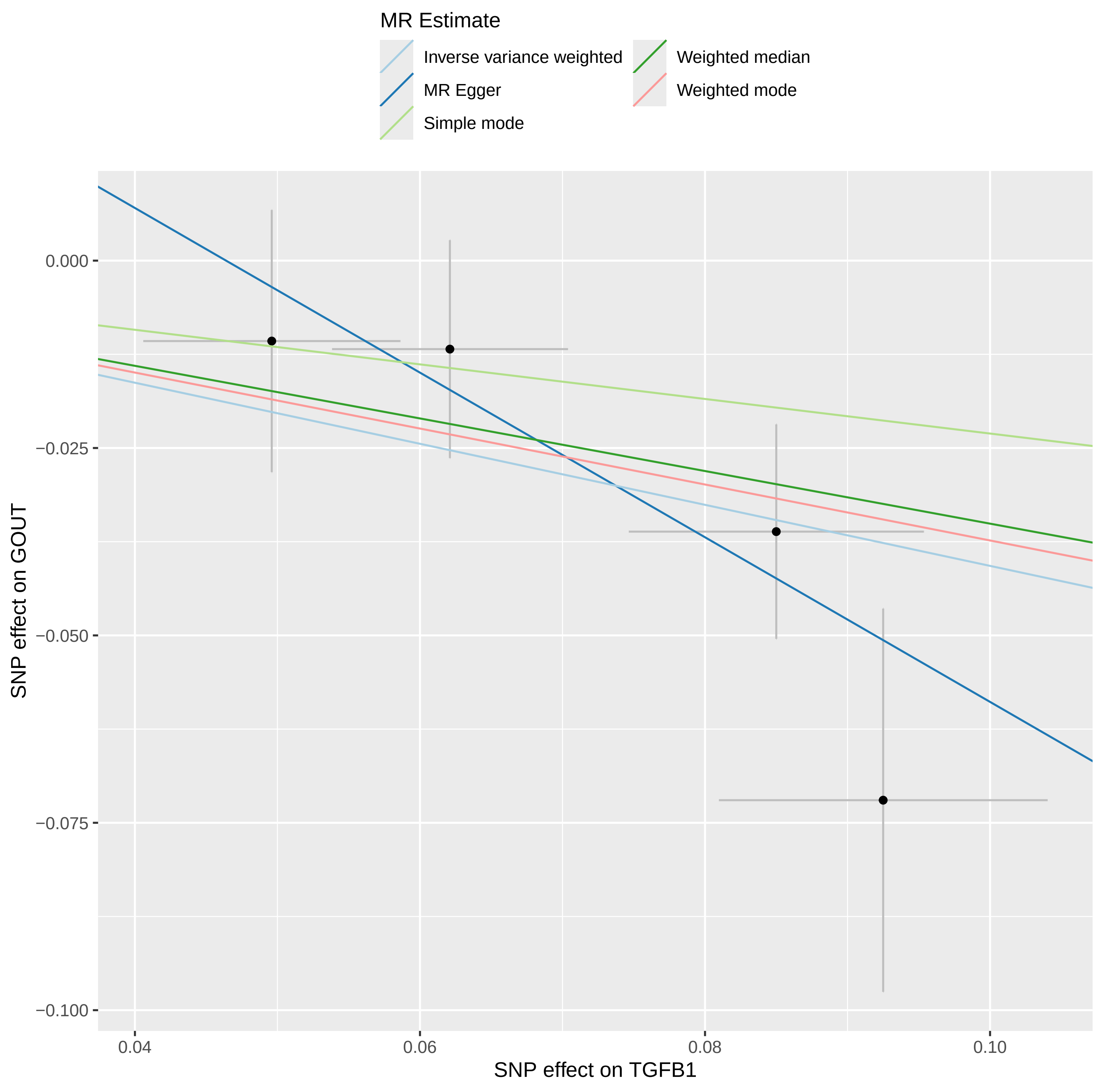

MR Estimate
Inverse variance weighted
MR Egger
Simple mode
Weighted median
Weighted mode
0.000
−0.025
−0.050
−0.075
−0.100
0.04
0.06
0.08
0.10
SNP effect on GOUT
SNP effect on TGFB1

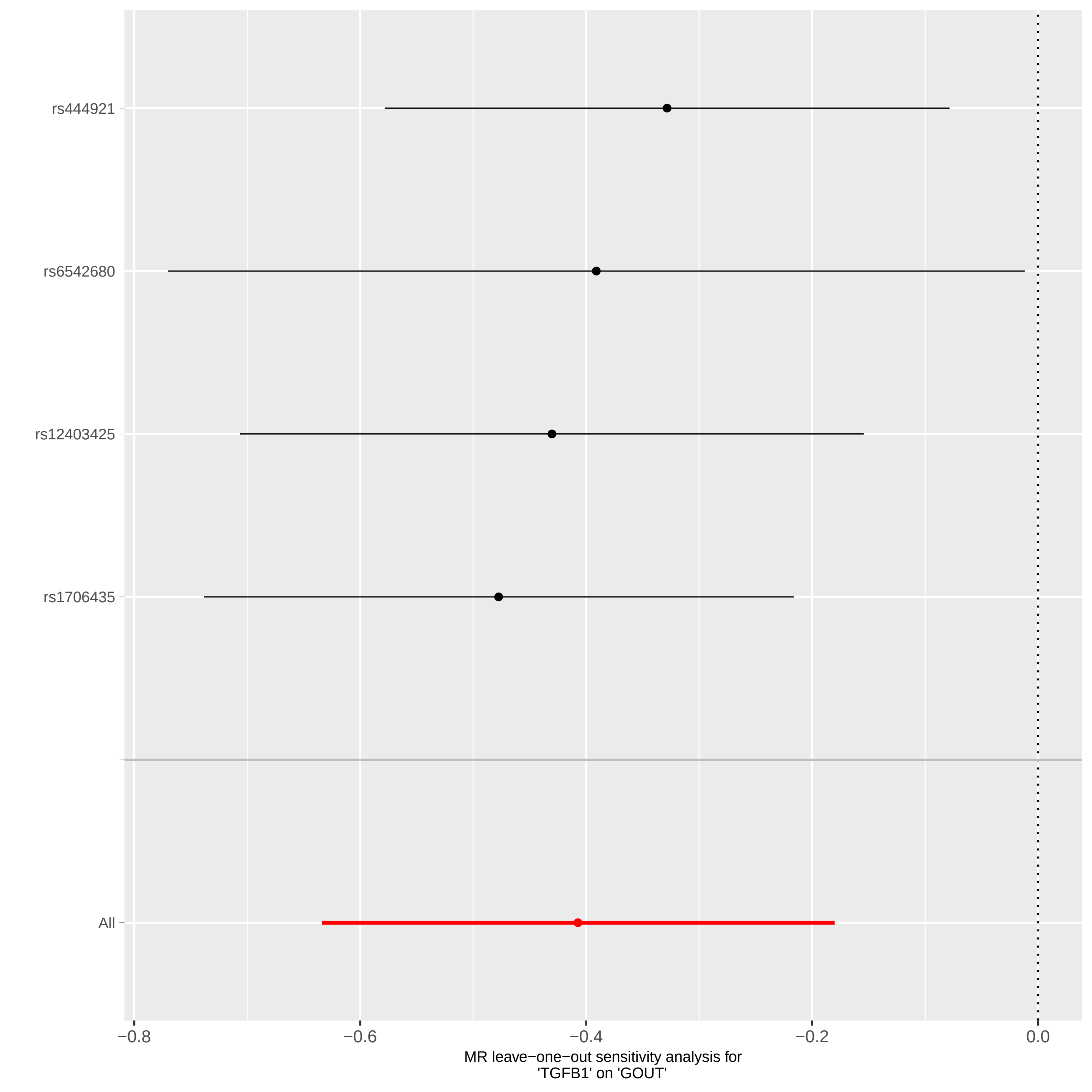

rs444921
rs6542680
rs12403425
rs1706435
All
−0.8
−0.6
−0.4
−0.2
0.0
MR leave−one−out sensitivity analysis for
'TGFB1' on 'GOUT'

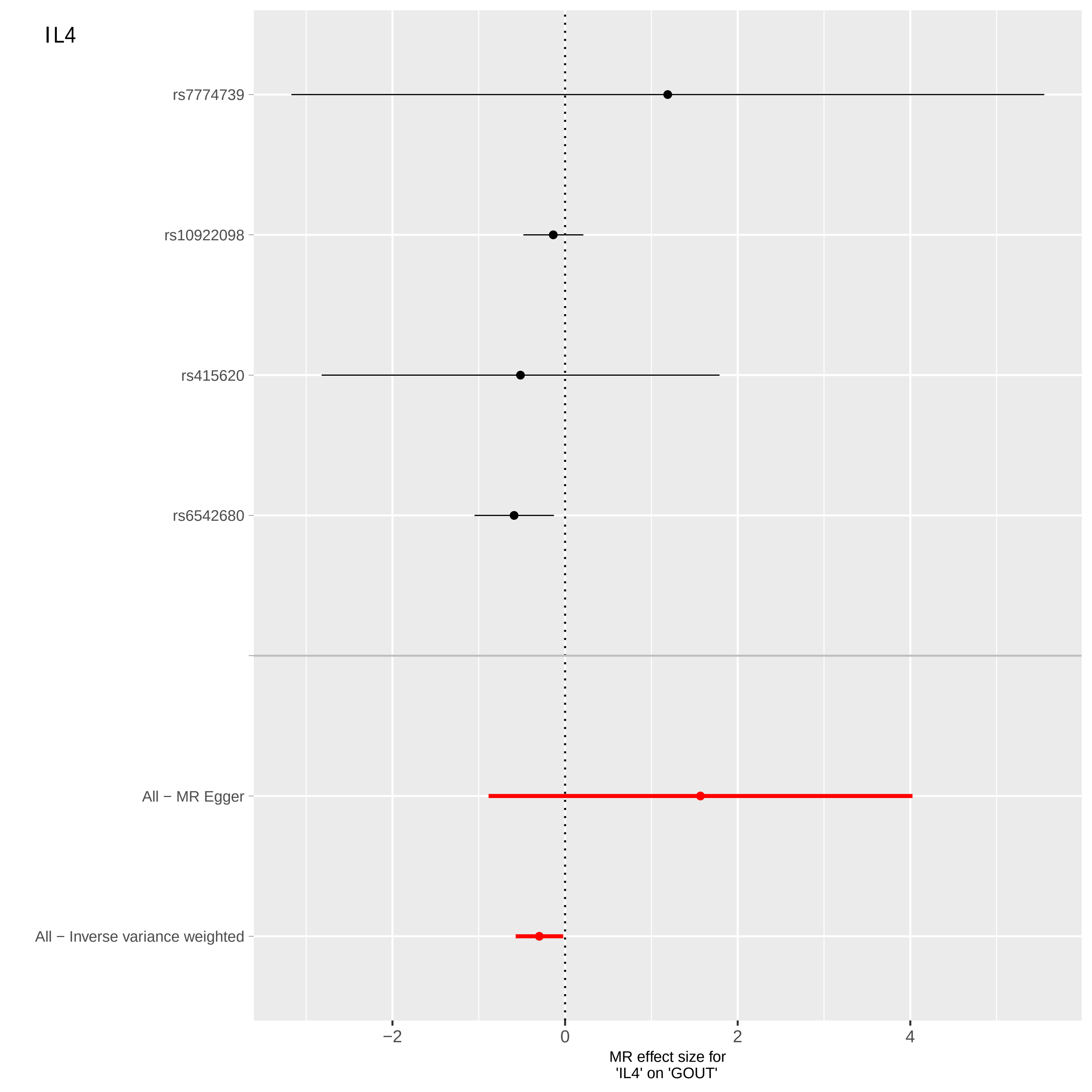

IL4
rs7774739
rs10922098
rs415620
rs6542680
All − MR Egger
All − Inverse variance weighted
−2
0
2
4
MR effect size for
'IL4' on 'GOUT'

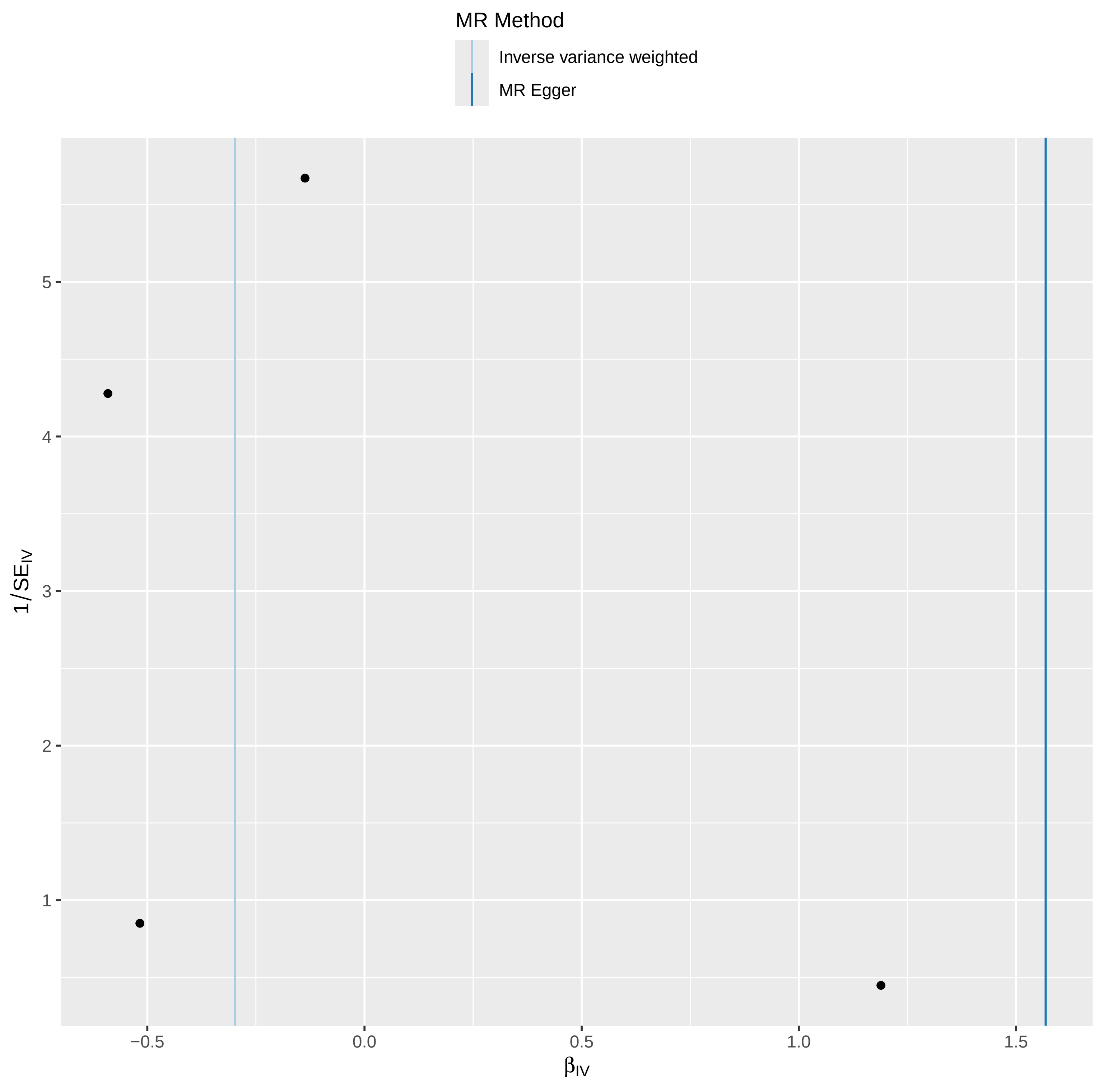
MR Method
Inverse variance weighted
MR Egger
1/SE_IV
β_IV
−0.5
0.0
0.5
1.0
1.5
1
2
3
4
5

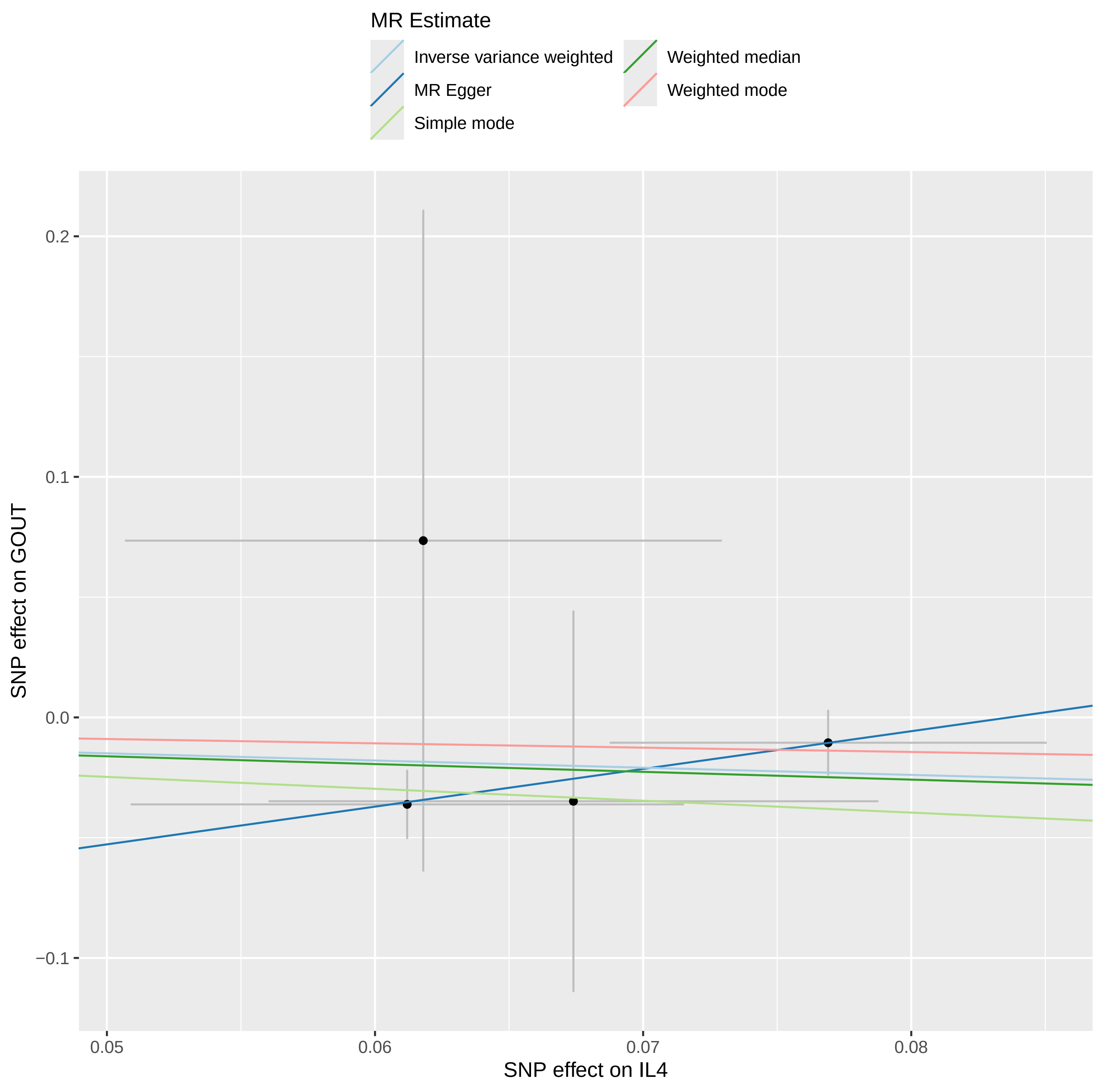

MR Estimate
Inverse variance weighted
MR Egger
Simple mode
Weighted median
Weighted mode
0.2
0.1
0.0
−0.1
0.05
0.06
0.07
0.08
SNP effect on GOUT
SNP effect on IL4

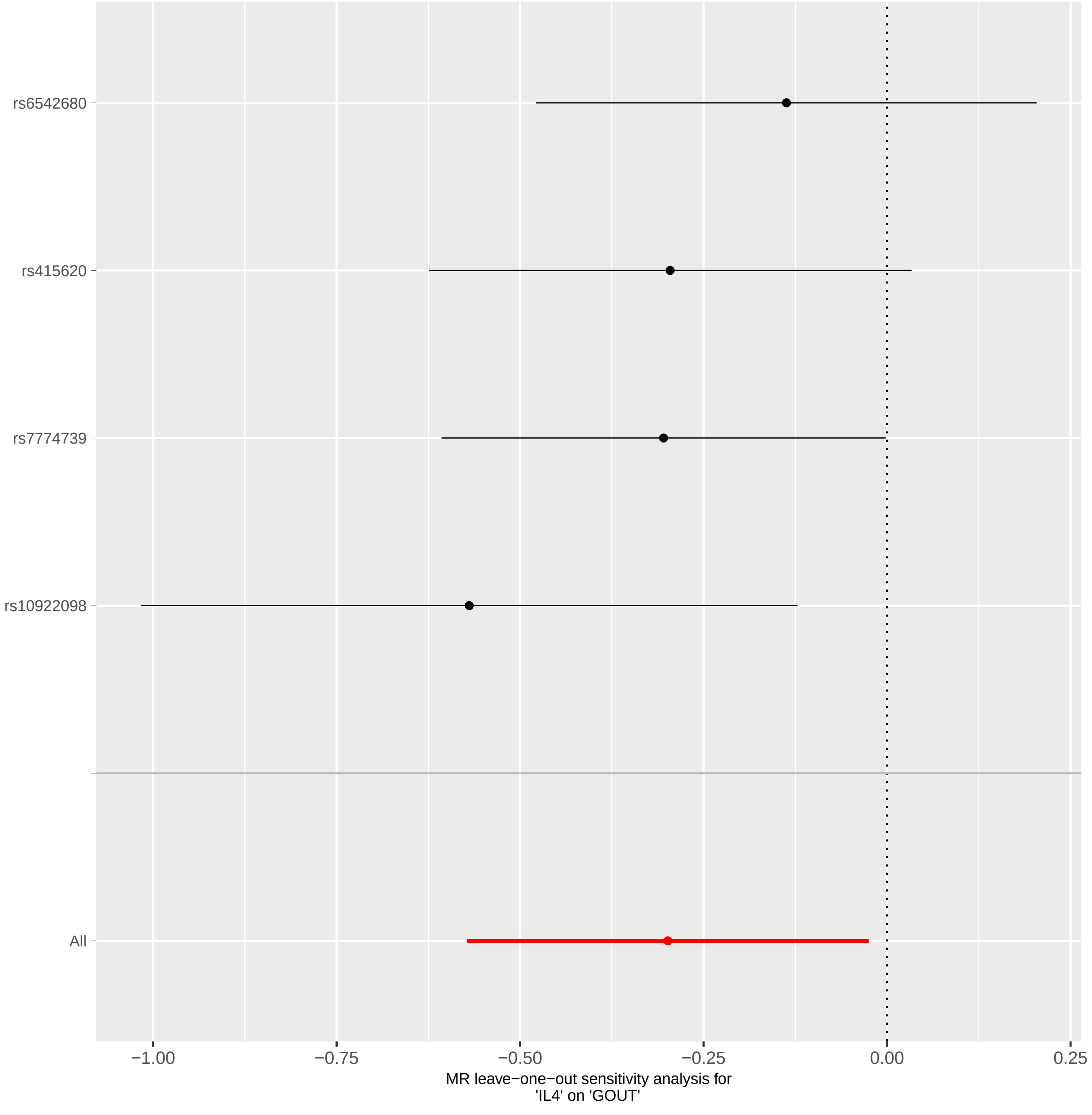

rs6542680
rs415620
rs7774739
rs10922098
All
−1.00
−0.75
−0.50
−0.25
0.00
0.25
MR leave−one−out sensitivity analysis for
'IL4' on 'GOUT'

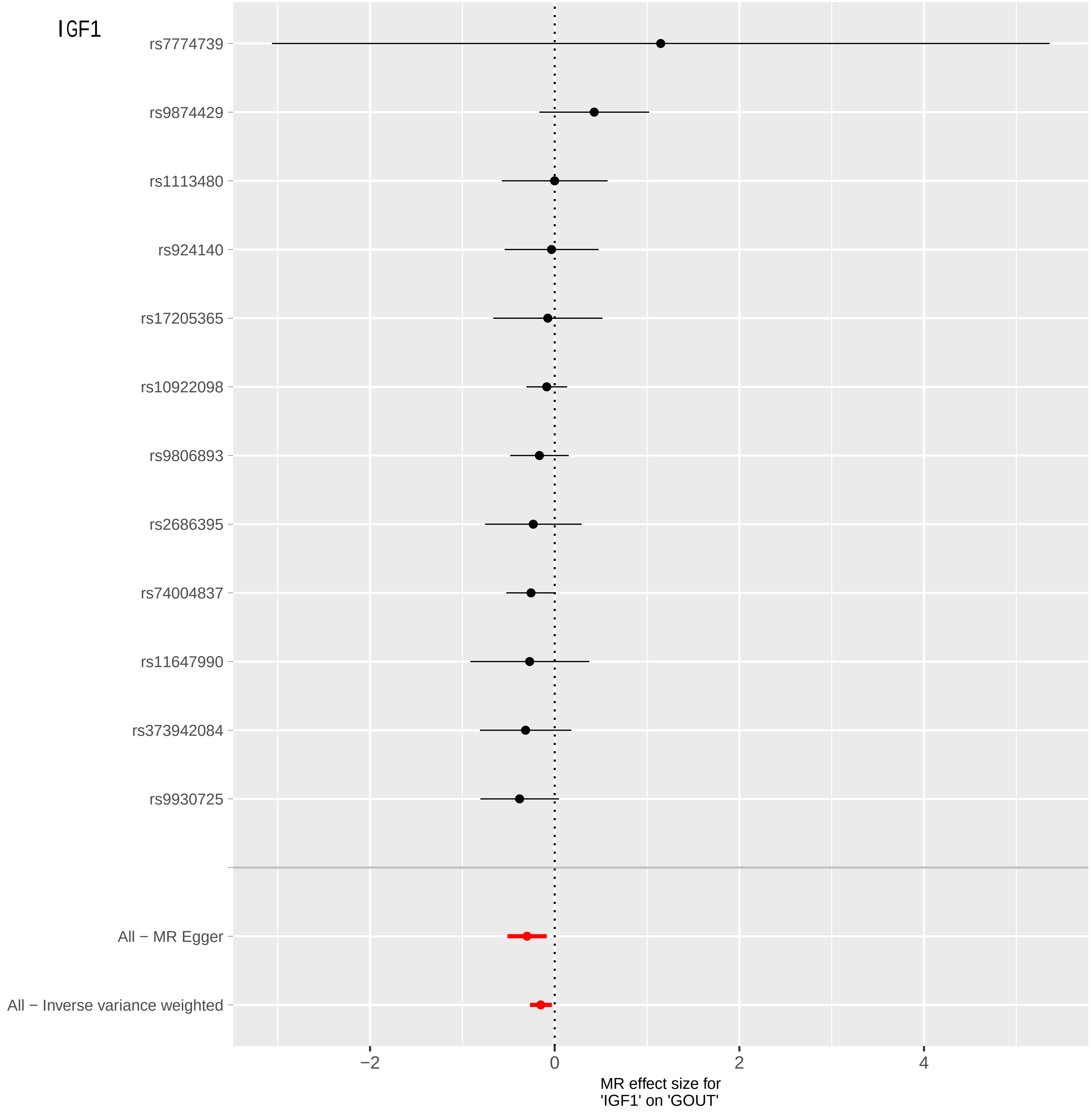
IGF1
rs7774739
rs9874429
rs1113480
rs924140
rs17205365
rs10922098
rs9806893
rs2686395
rs74004837
rs11647990
rs373942084
rs9930725
All − MR Egger
All − Inverse variance weighted
−2
0
2
4
MR effect size for
'IGF1' on 'GOUT'

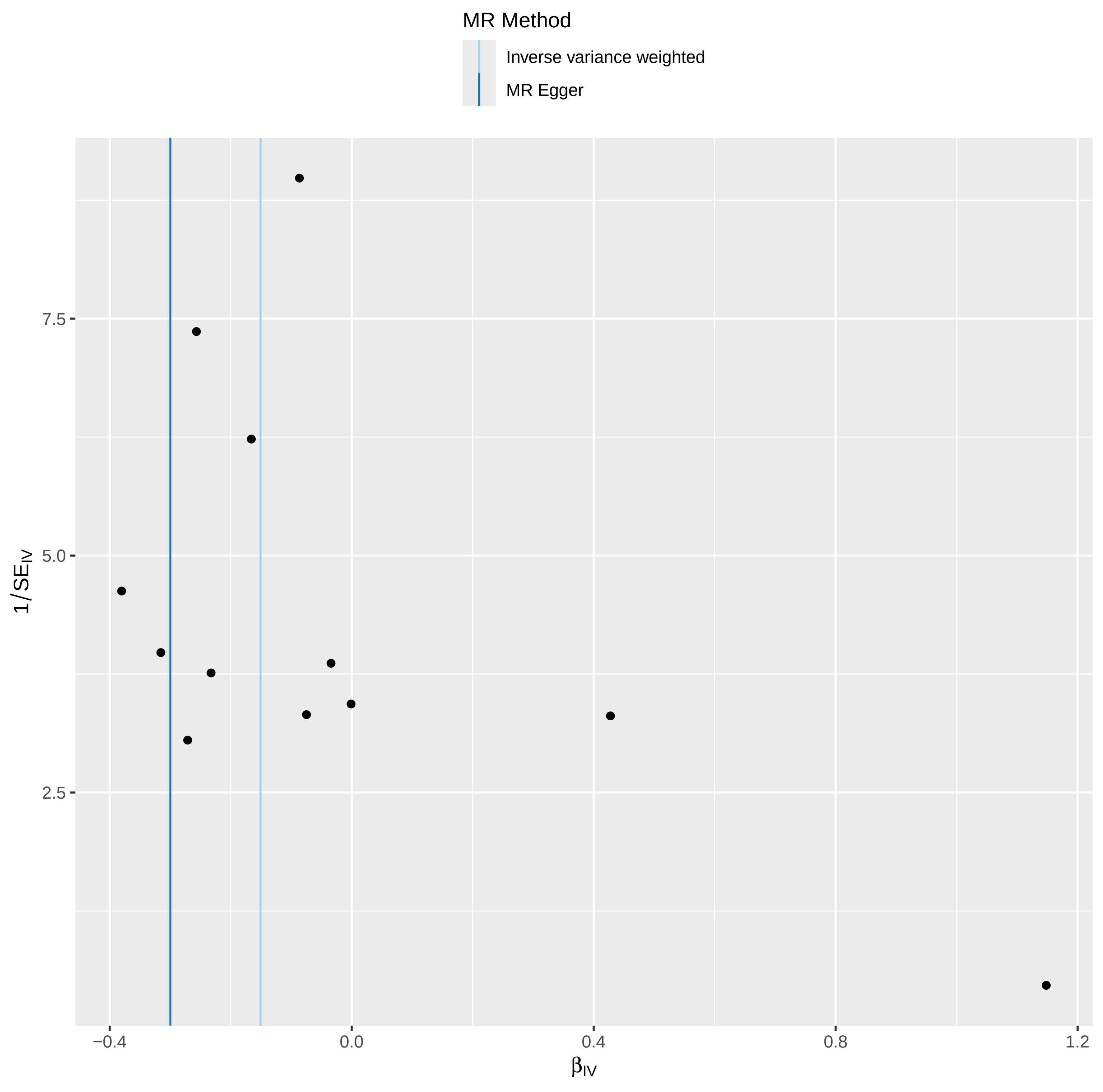
MR Method
Inverse variance weighted
MR Egger
7.5
5.0
2.5
−0.4
0.0
0.4
0.8
1.2
β_{IV}
$1/SE_{IV}$

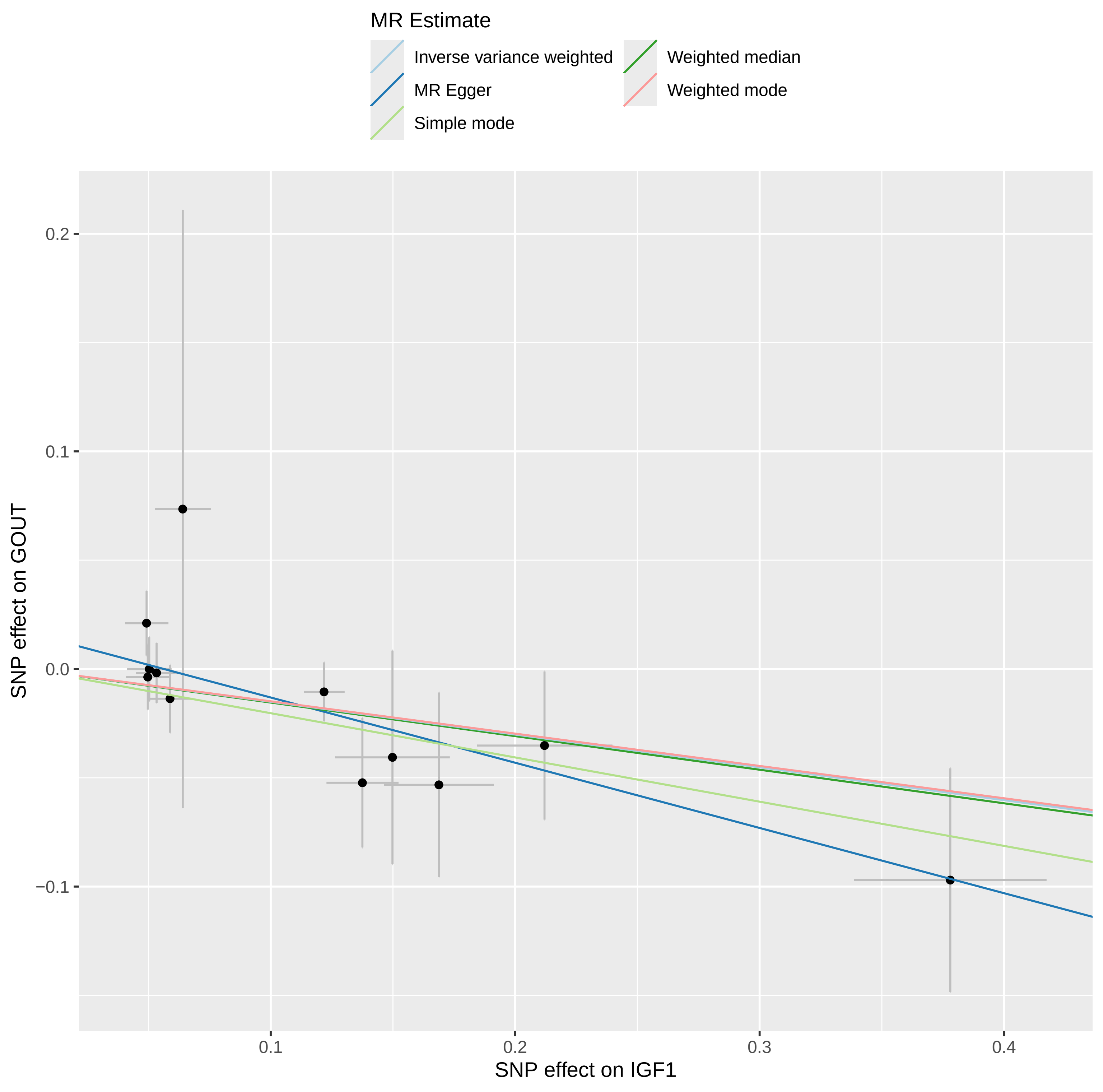
MR Estimate
Inverse variance weighted
MR Egger
Simple mode
Weighted median
Weighted mode
0.2
0.1
0.0
−0.1
SNP effect on GOUT
0.1
0.2
0.3
0.4
SNP effect on IGF1

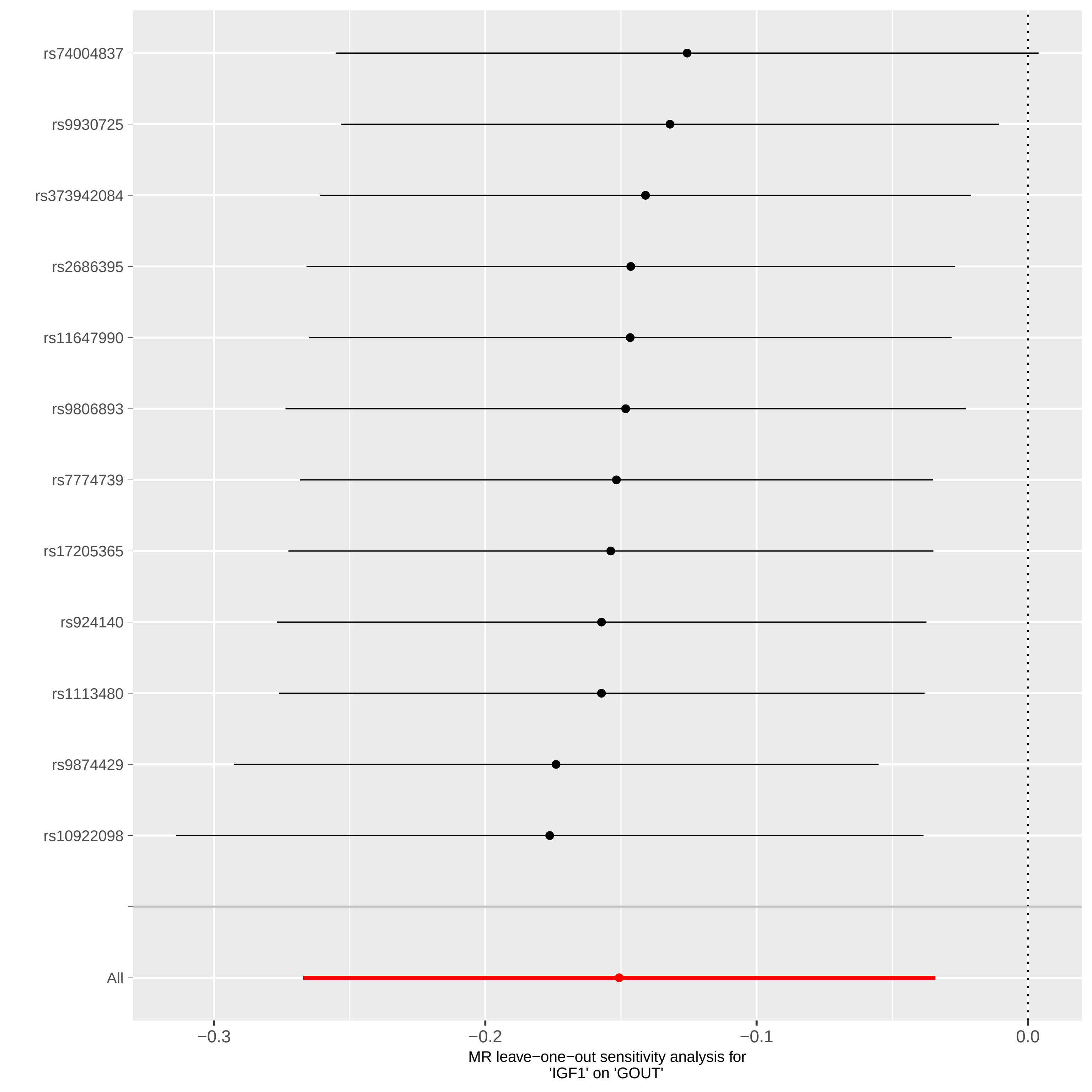
rs74004837
rs9930725
rs373942084
rs2686395
rs11647990
rs9806893
rs7774739
rs17205365
rs924140
rs1113480
rs9874429
rs10922098
All
−0.3
−0.2
−0.1
0.0
MR leave−one−out sensitivity analysis for
'IGF1' on 'GOUT'

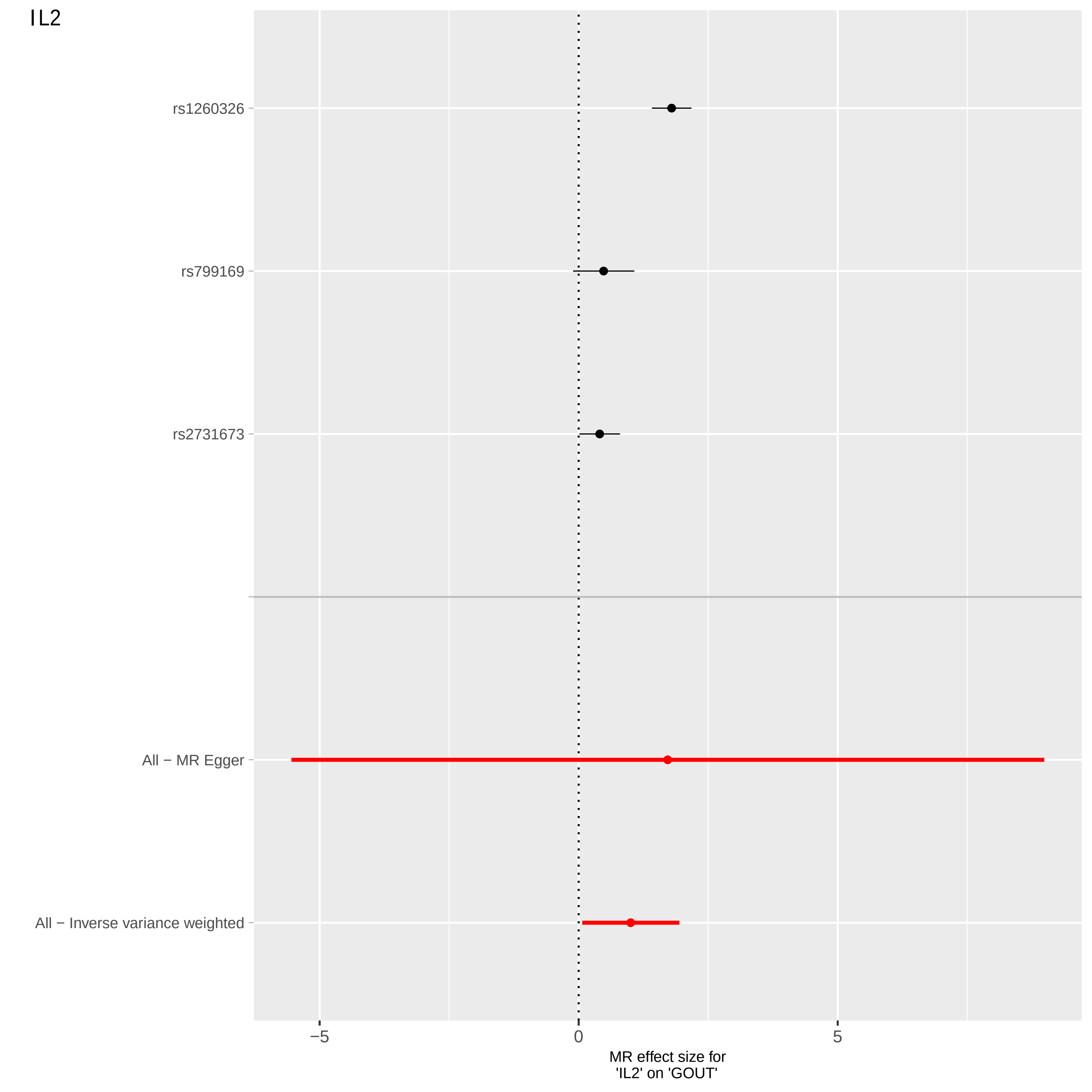
IL2
rs1260326
rs799169
rs2731673
All − MR Egger
All − Inverse variance weighted
−5
0
5
MR effect size for
'IL2' on 'GOUT'

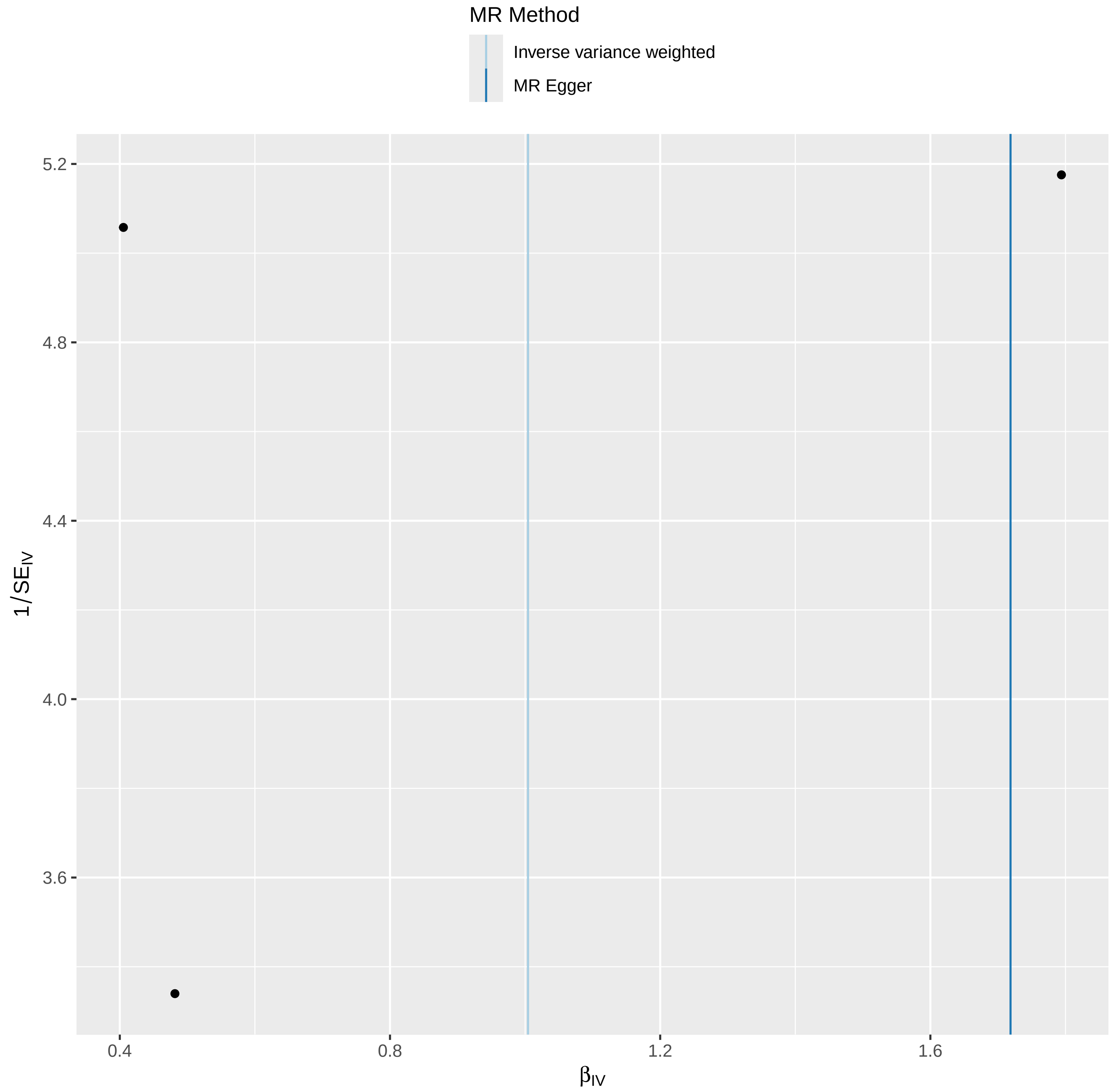
MR Method
Inverse variance weighted
MR Egger
5.2
4.8
4.4
4.0
3.6
$1/SE_{IV}$
0.4
0.8
1.2
1.6
β_{IV}

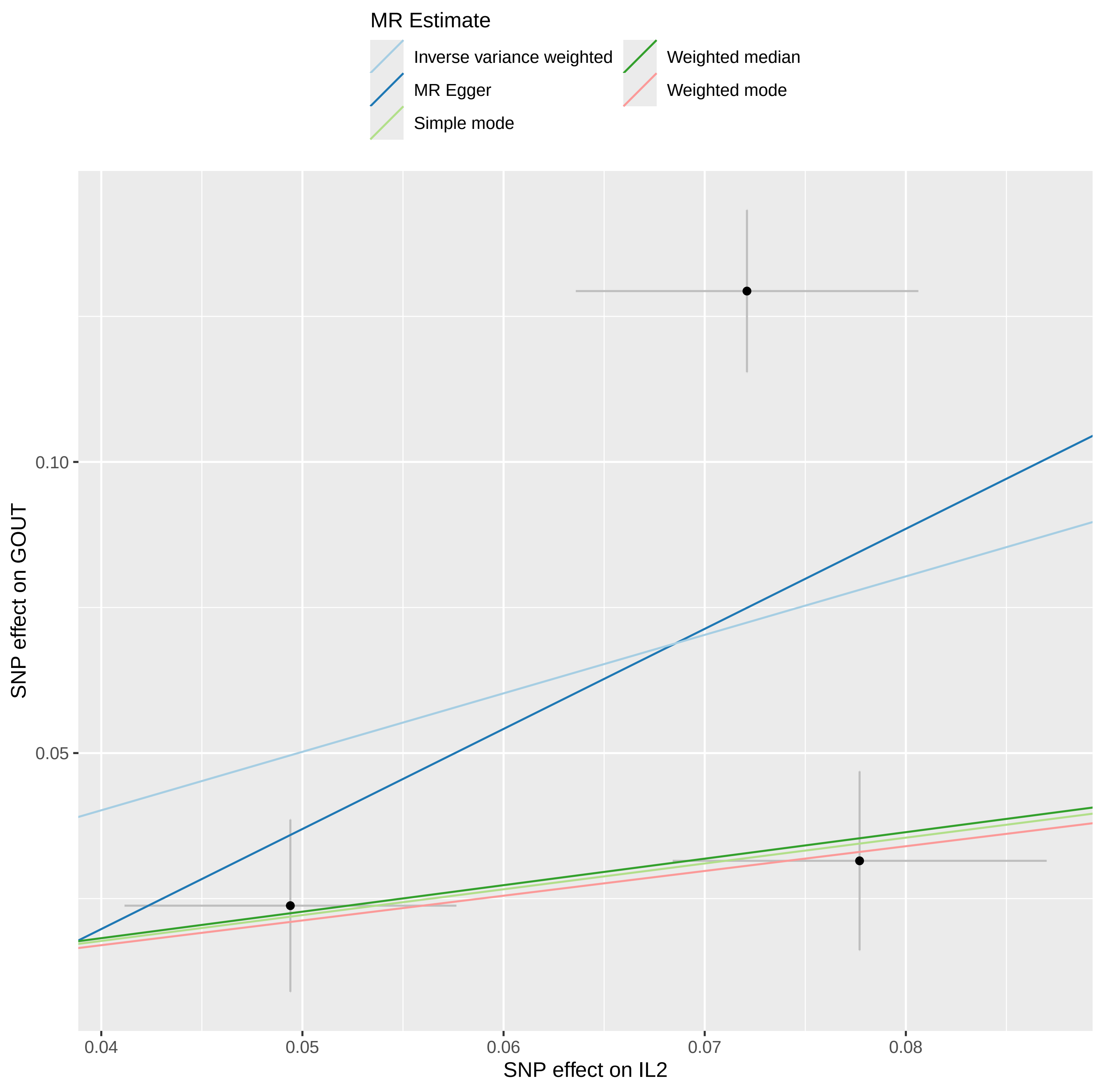
MR Estimate
Inverse variance weighted
MR Egger
Simple mode
Weighted median
Weighted mode
0.10
0.05
SNP effect on GOUT
0.04
0.05
0.06
0.07
0.08
SNP effect on IL2

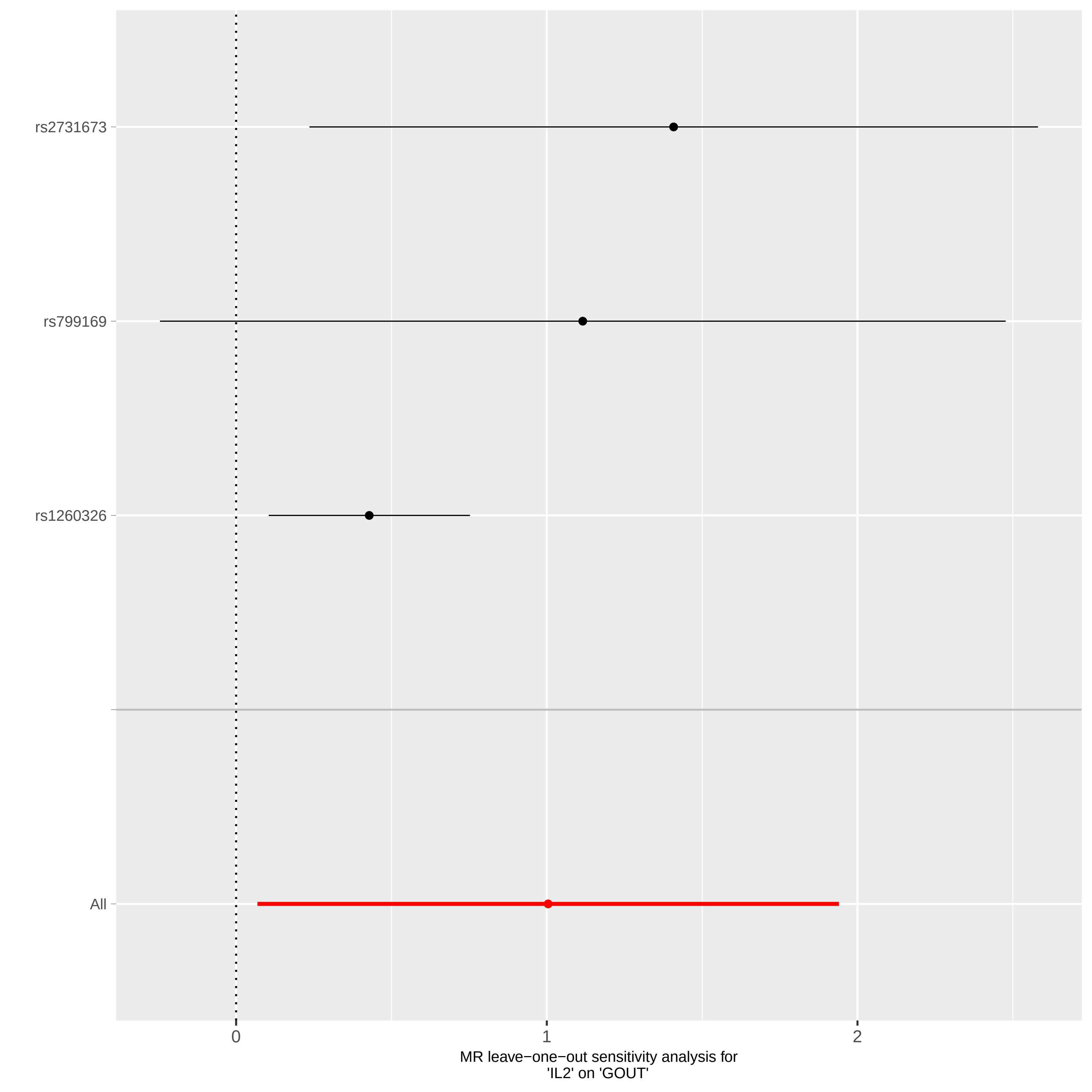
rs2731673
rs799169
rs1260326
All
0
1
2
MR leave−one−out sensitivity analysis for
'IL2' on 'GOUT'

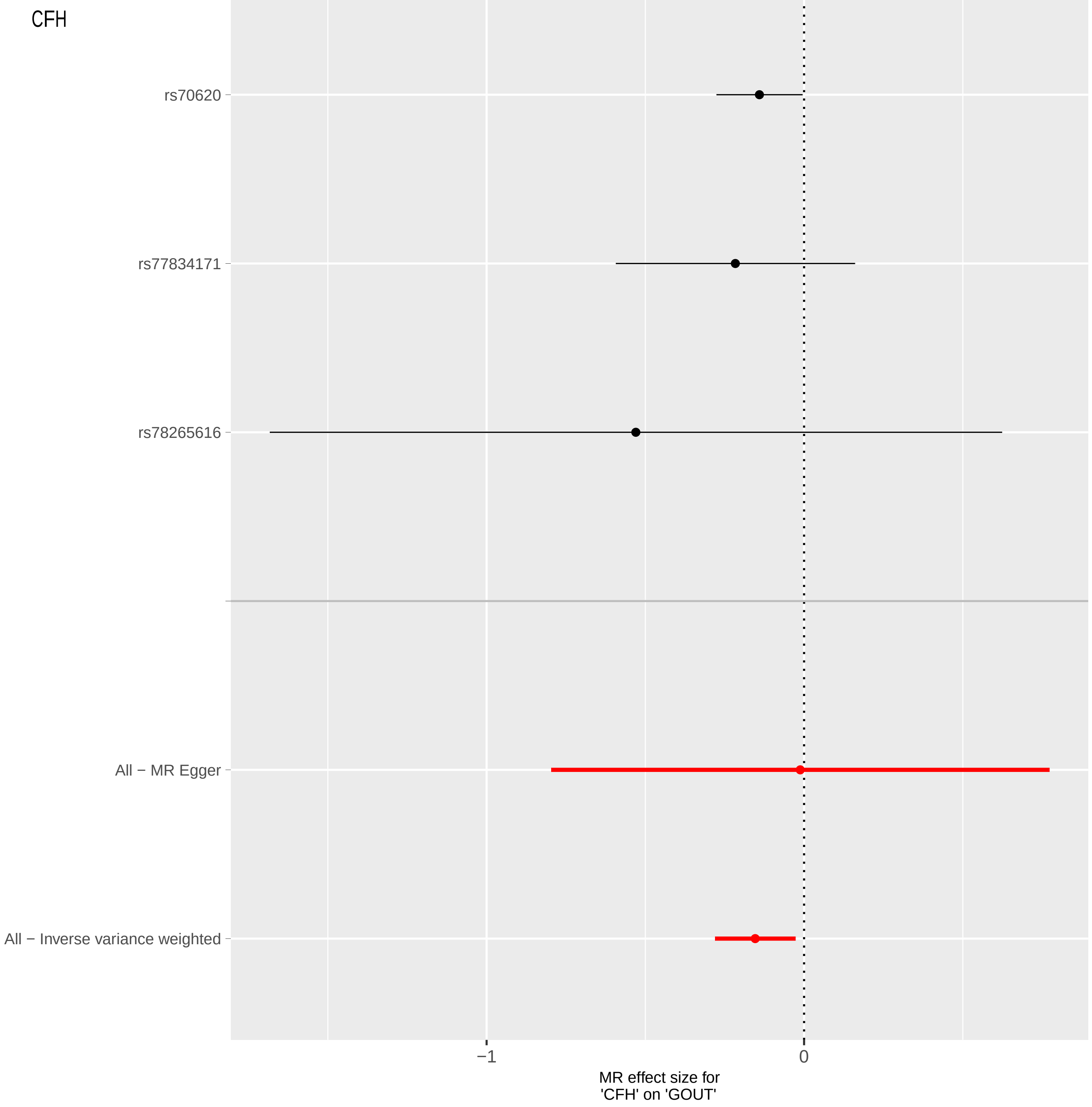
CFH
rs70620
rs77834171
rs78265616
All − MR Egger
All − Inverse variance weighted
−1
0
MR effect size for
'CFH' on 'GOUT'

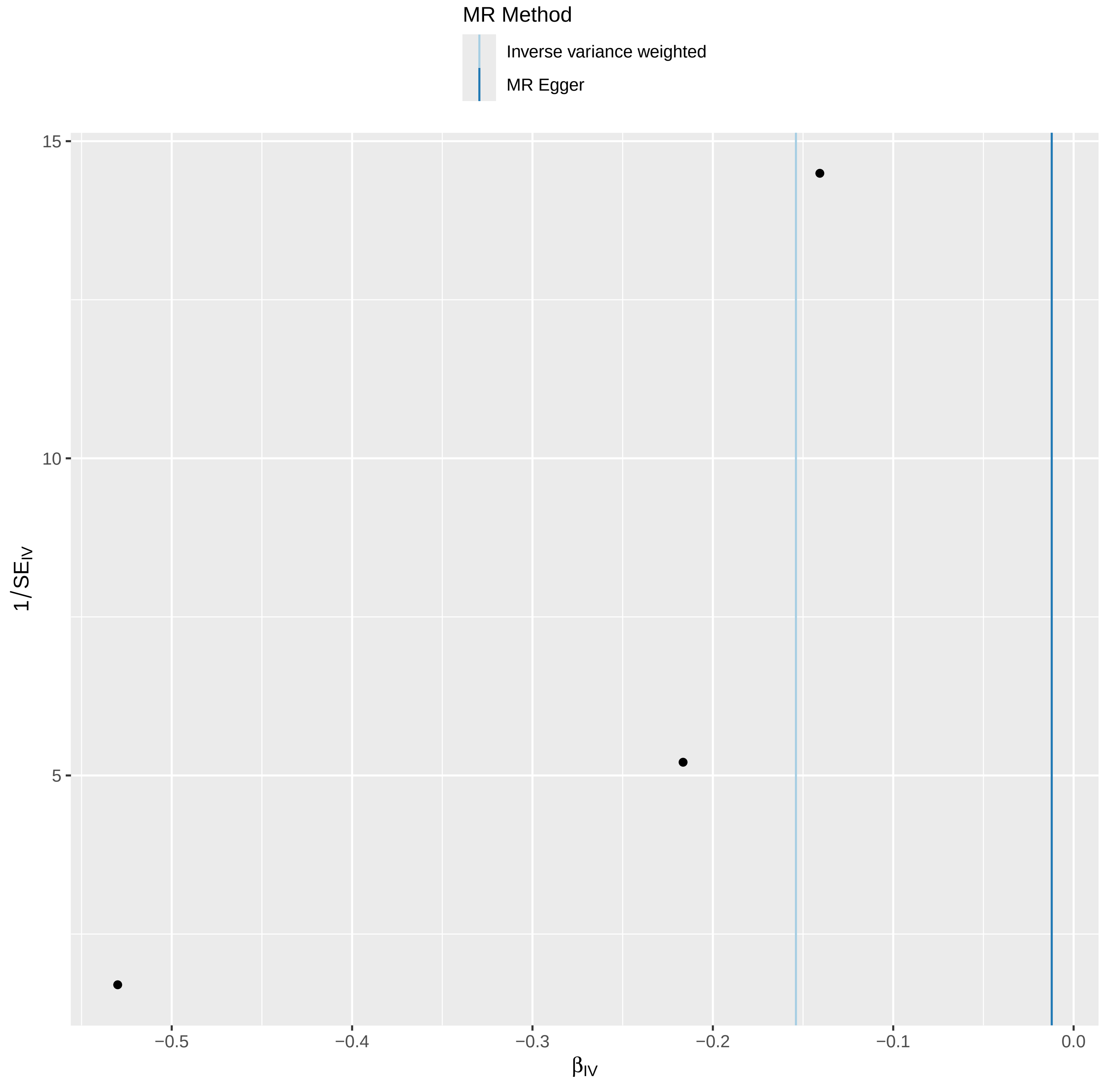

MR Method
Inverse variance weighted
MR Egger
15
10
5
1/SE_IV
−0.5
−0.4
−0.3
−0.2
−0.1
0.0
β_IV

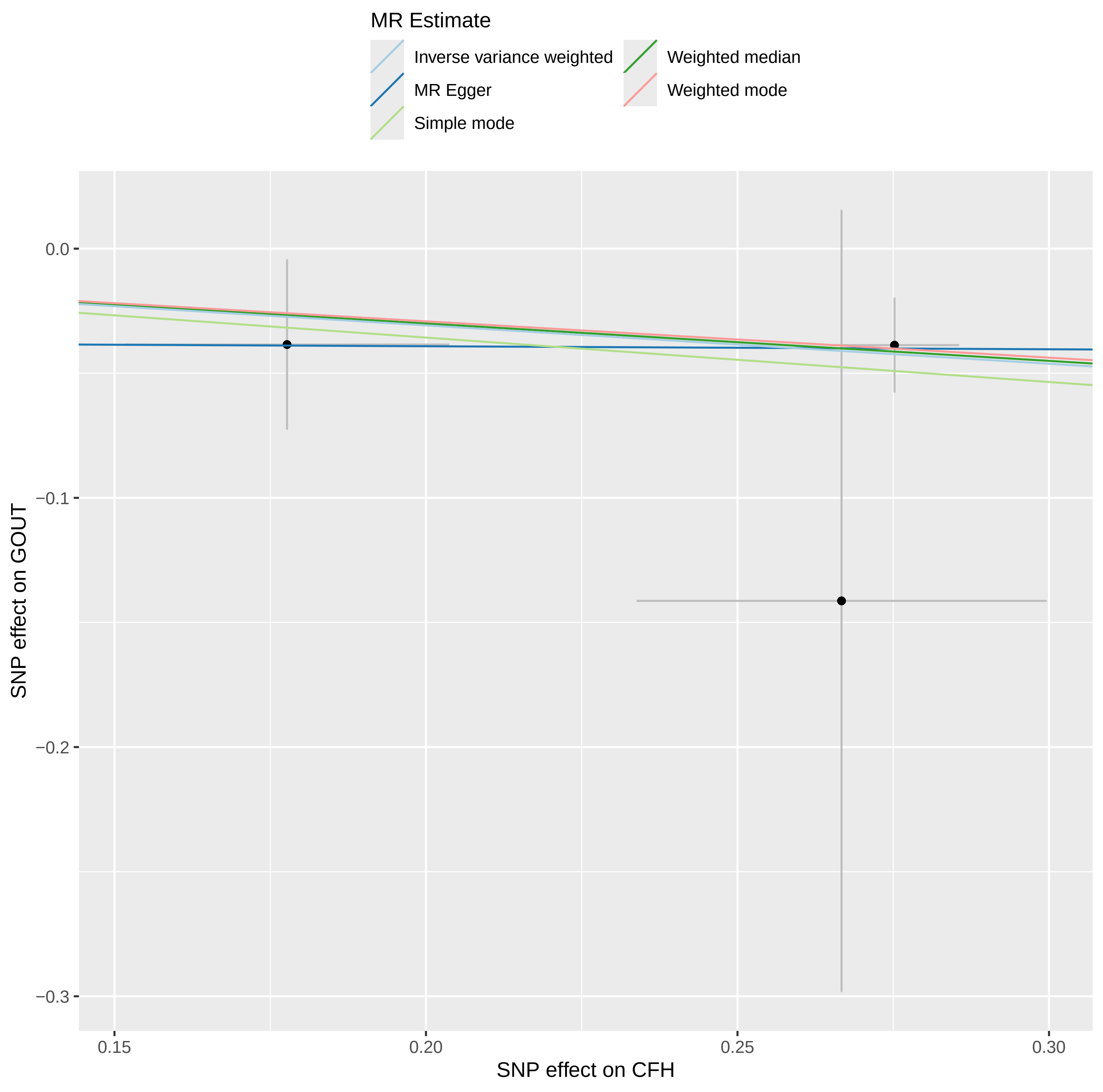
MR Estimate
Inverse variance weighted
MR Egger
Simple mode
Weighted median
Weighted mode
0.0
−0.1
−0.2
−0.3
0.15
0.20
0.25
0.30
SNP effect on GOUT
SNP effect on CFH

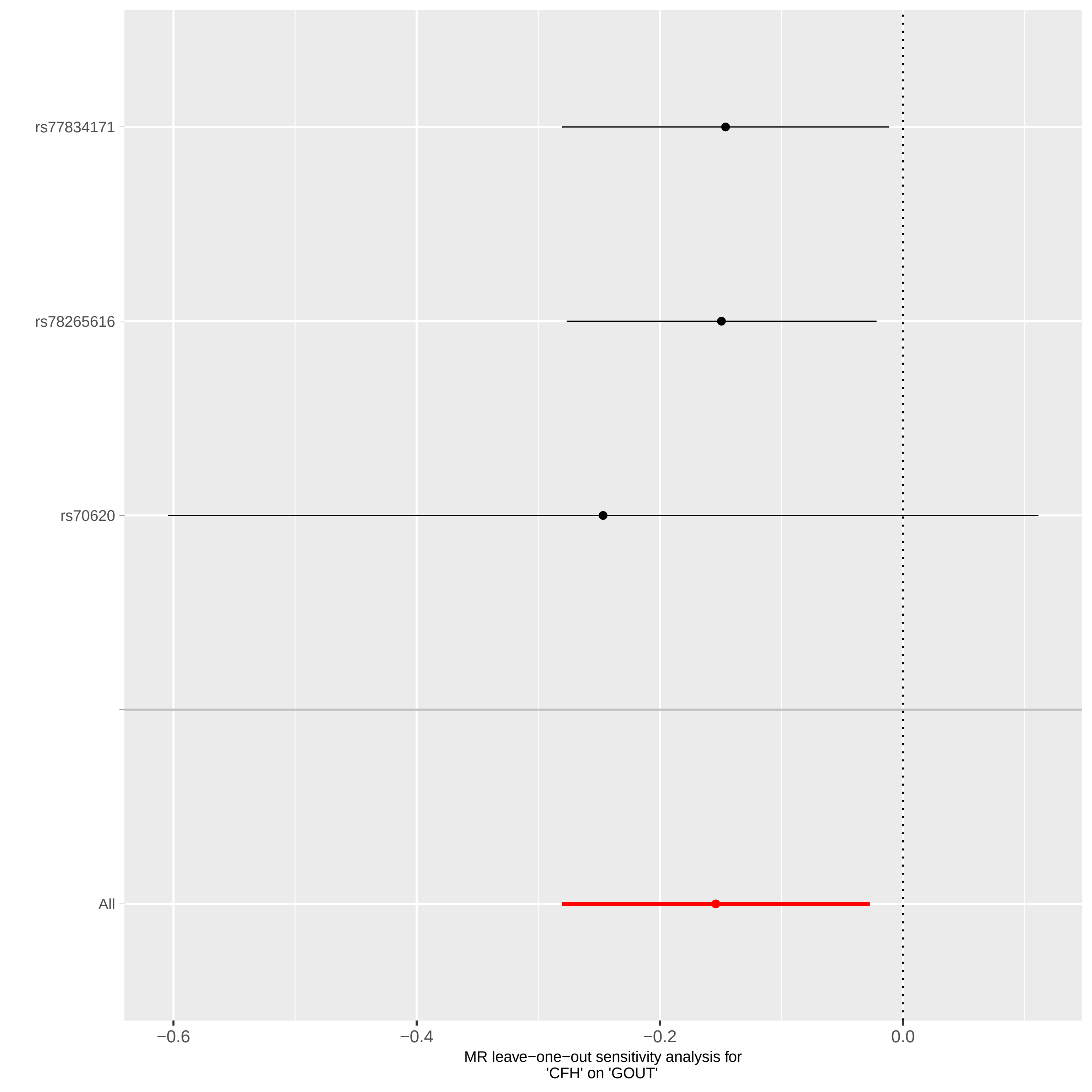
rs77834171
rs78265616
rs70620
All
−0.6
−0.4
−0.2
0.0
MR leave−one−out sensitivity analysis for
'CFH' on 'GOUT'

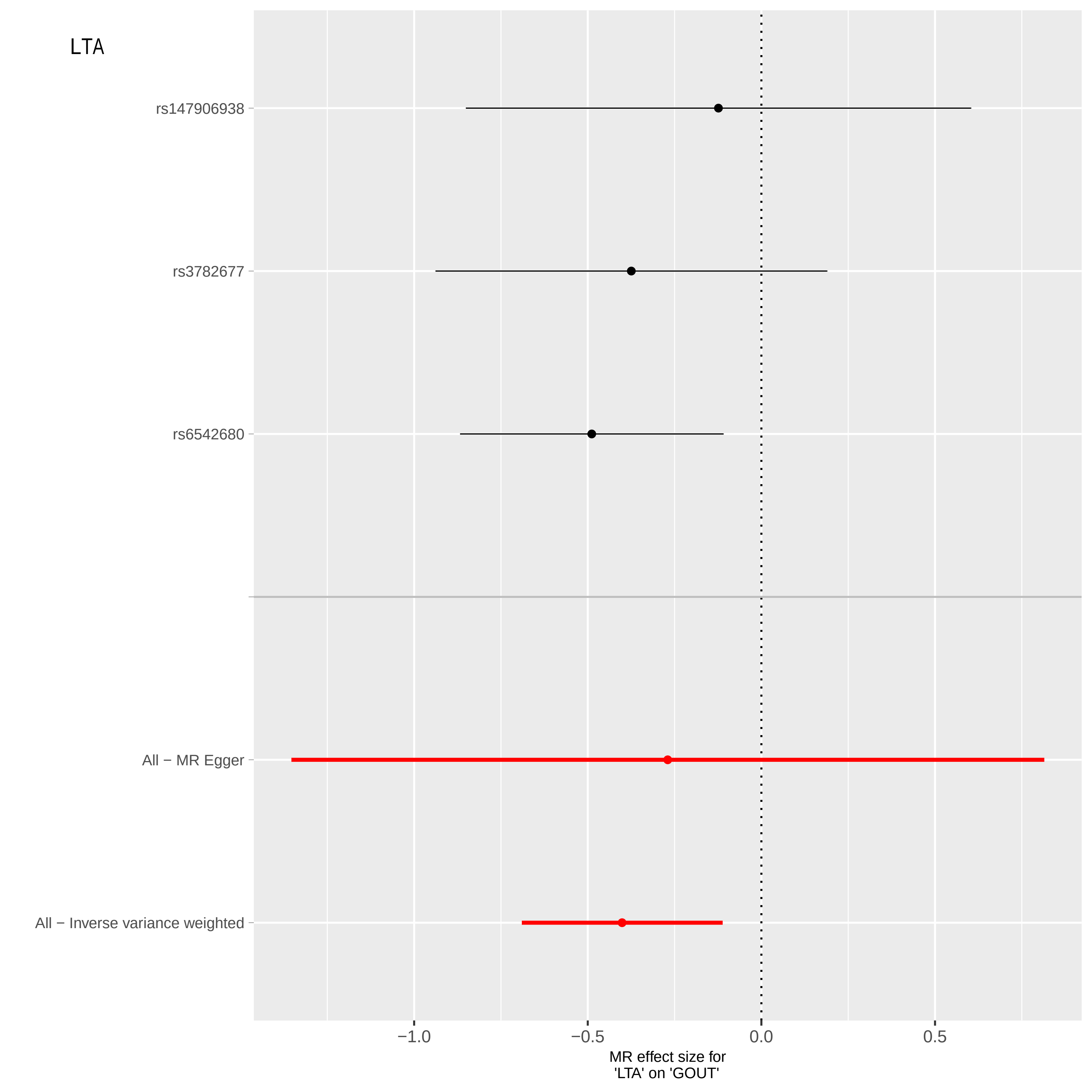
LTA
rs147906938
rs3782677
rs6542680
All − MR Egger
All − Inverse variance weighted
−1.0
−0.5
0.0
0.5
MR effect size for
'LTA' on 'GOUT'

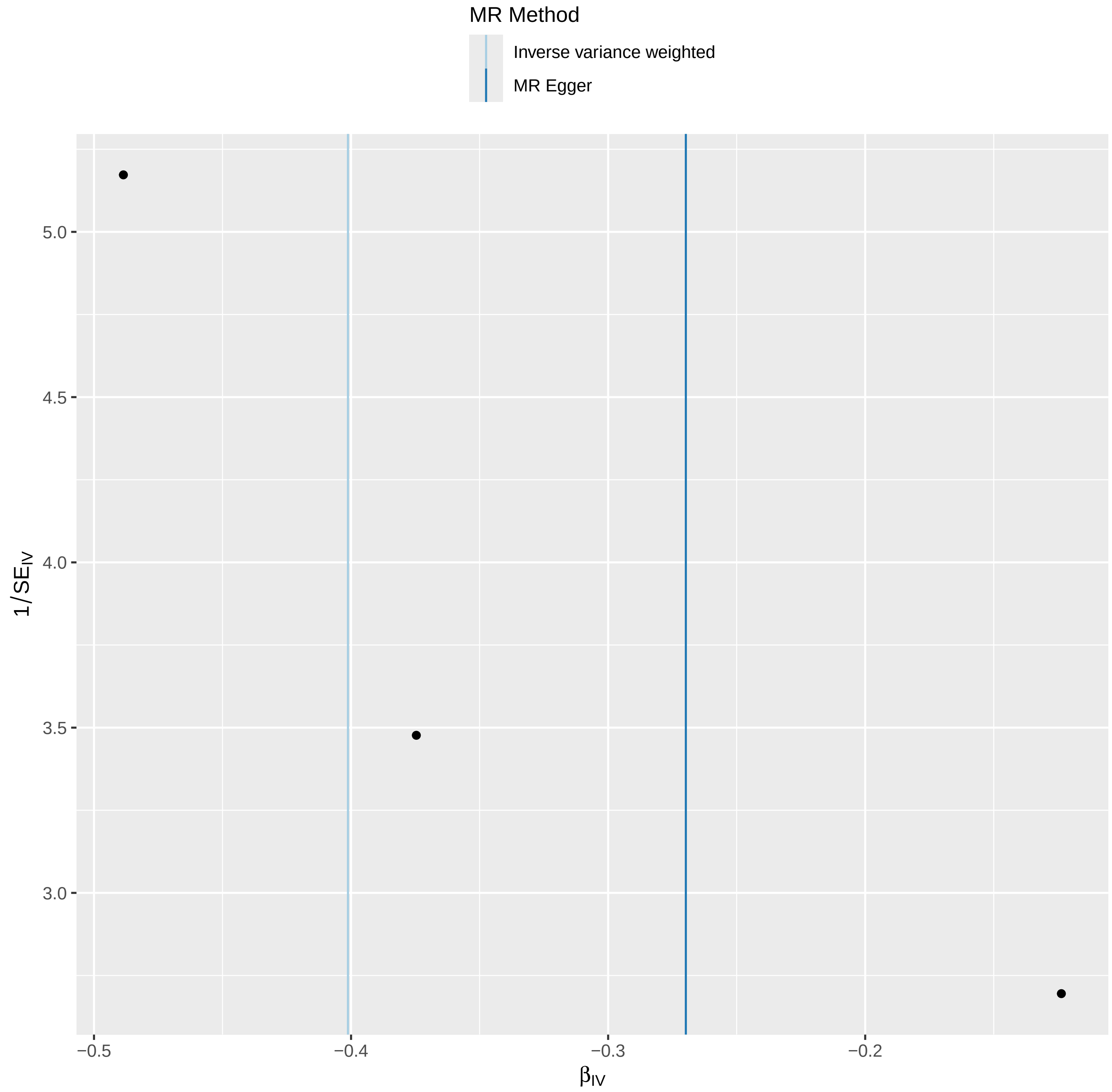

MR Method
Inverse variance weighted
MR Egger
5.0
4.5
4.0
3.5
3.0
1/SE$_{IV}$
−0.5
−0.4
−0.3
−0.2
β$_{IV}$

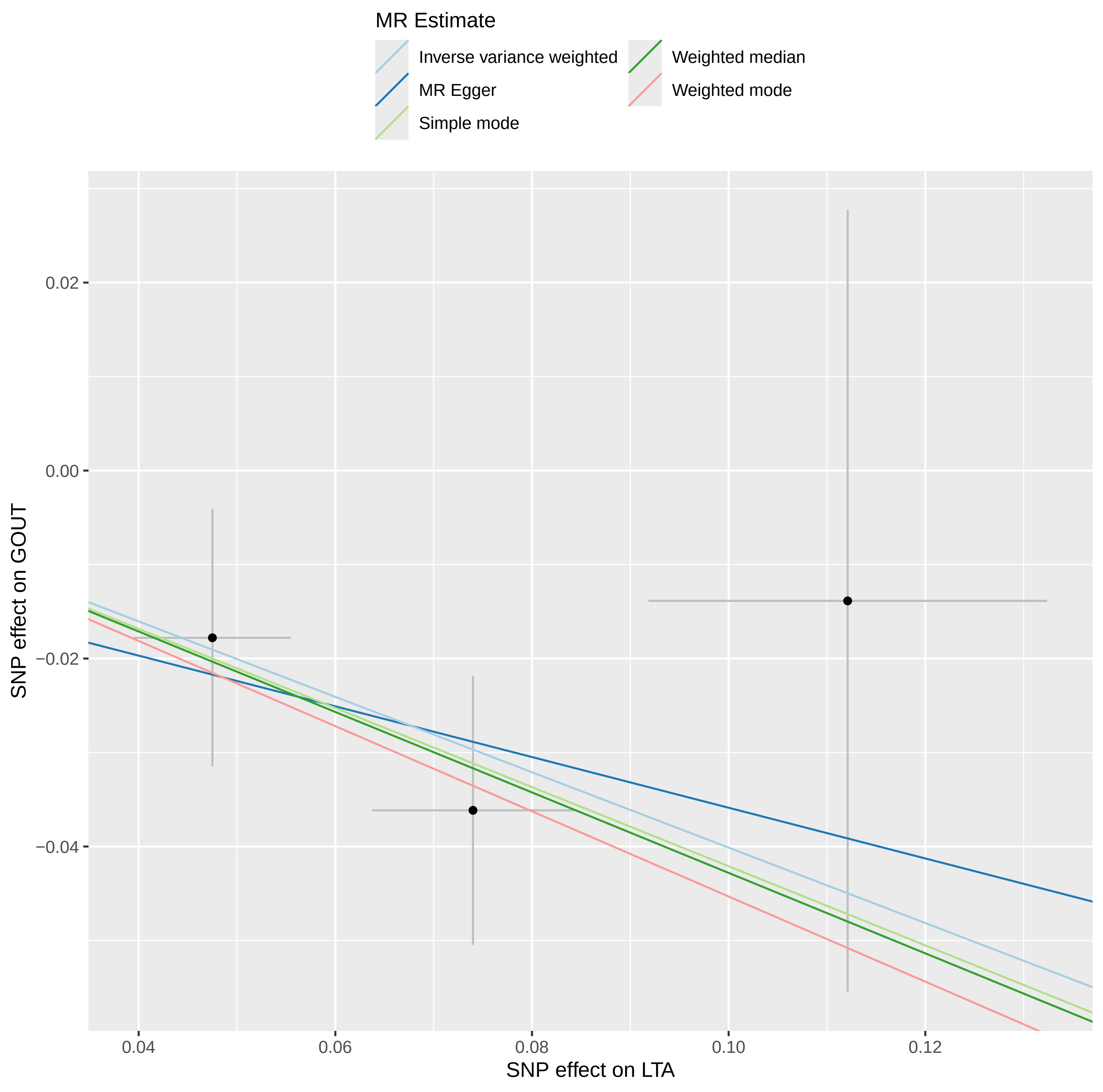
MR Estimate
Inverse variance weighted
MR Egger
Simple mode
Weighted median
Weighted mode
0.02
0.00
−0.02
−0.04
SNP effect on GOUT
0.04
0.06
0.08
0.10
0.12
SNP effect on LTA

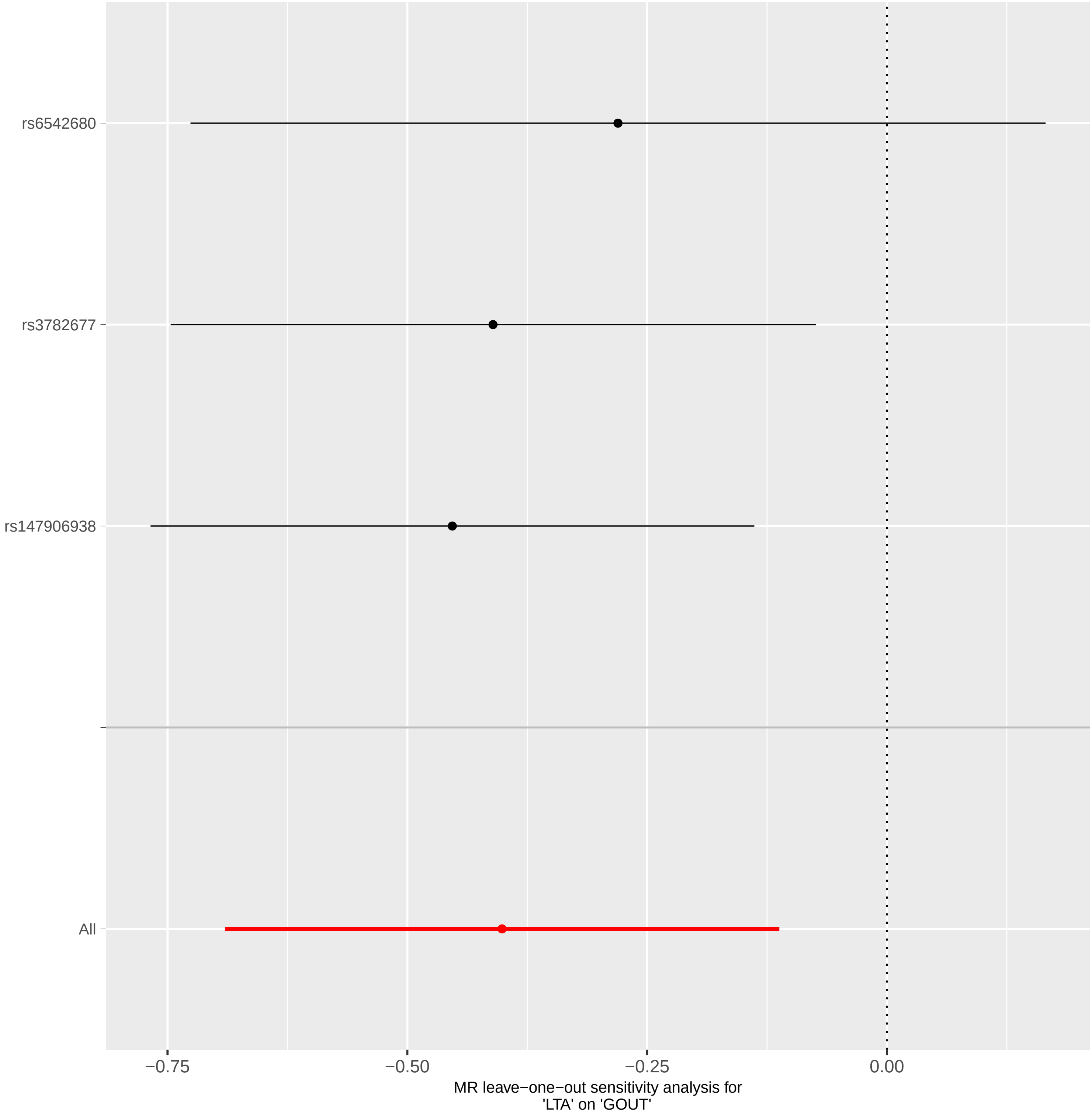
rs6542680
rs3782677
rs147906938
All
−0.75
−0.50
−0.25
0.00
MR leave−one−out sensitivity analysis for
'LTA' on 'GOUT'

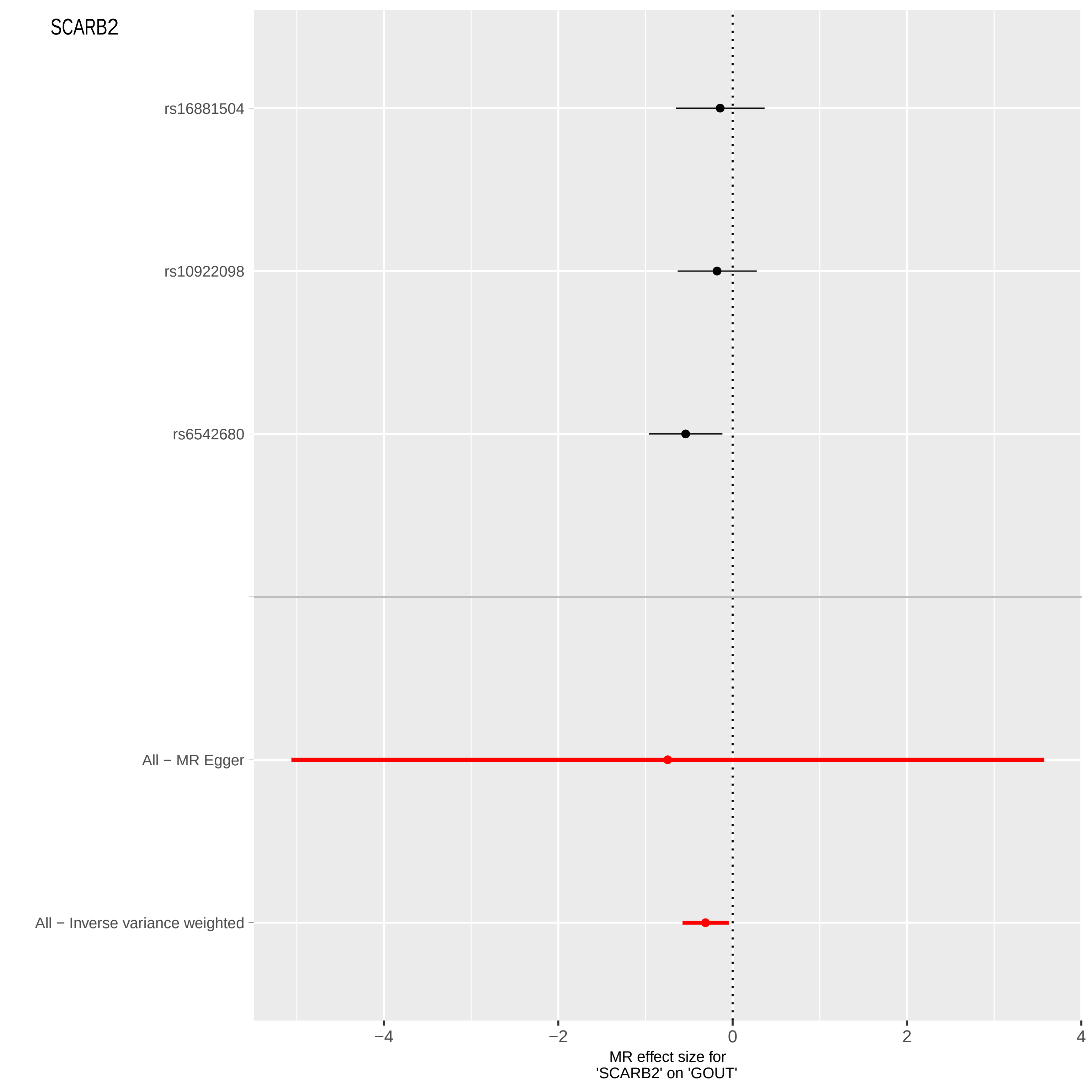

SCARB2
rs16881504
rs10922098
rs6542680
All − MR Egger
All − Inverse variance weighted
−4
−2
0
2
4
MR effect size for
'SCARB2' on 'GOUT'

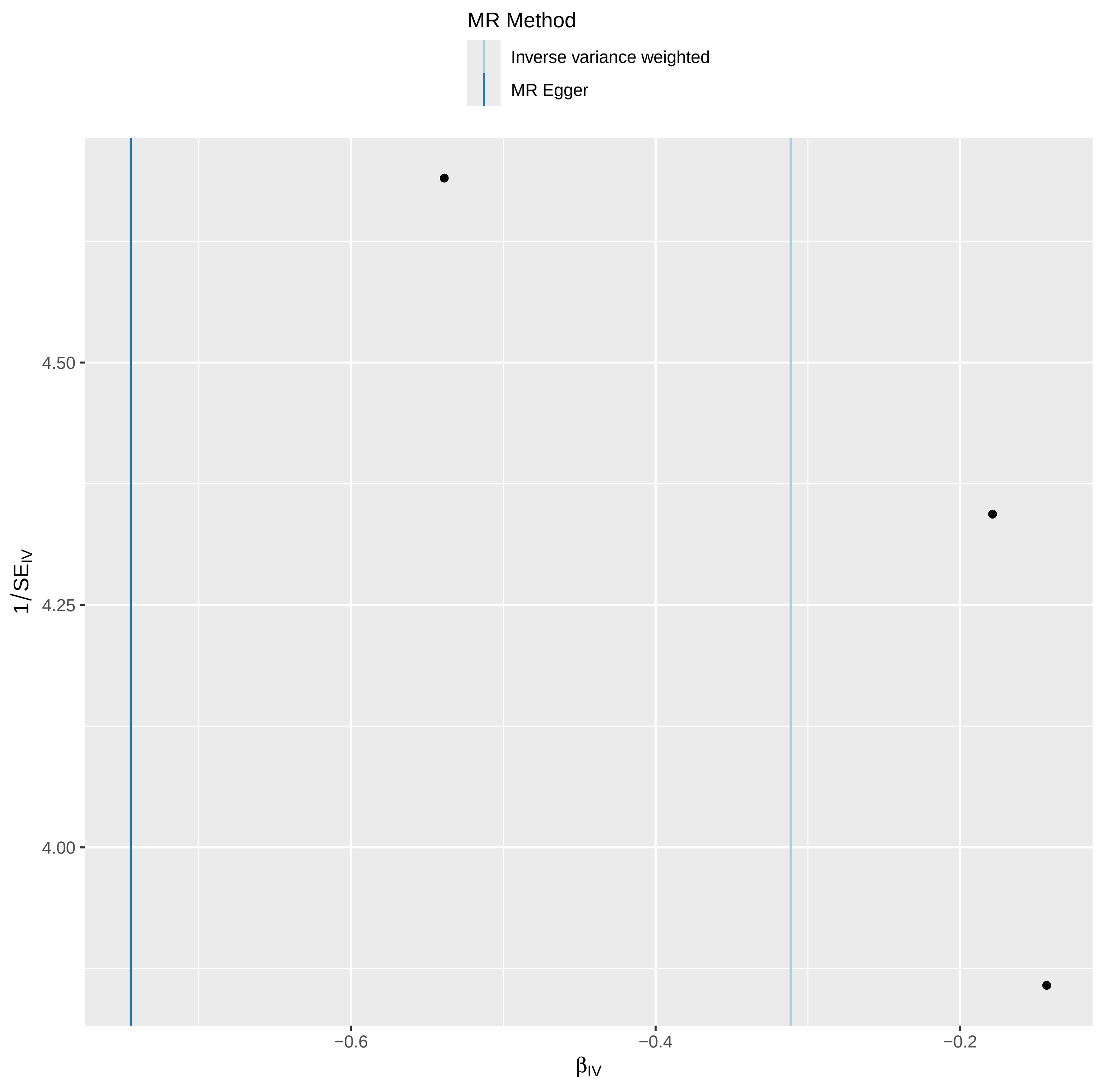
MR Method
Inverse variance weighted
MR Egger
4.50
4.25
4.00
−0.6
−0.4
−0.2
β_{IV}
$1/SE_{IV}$

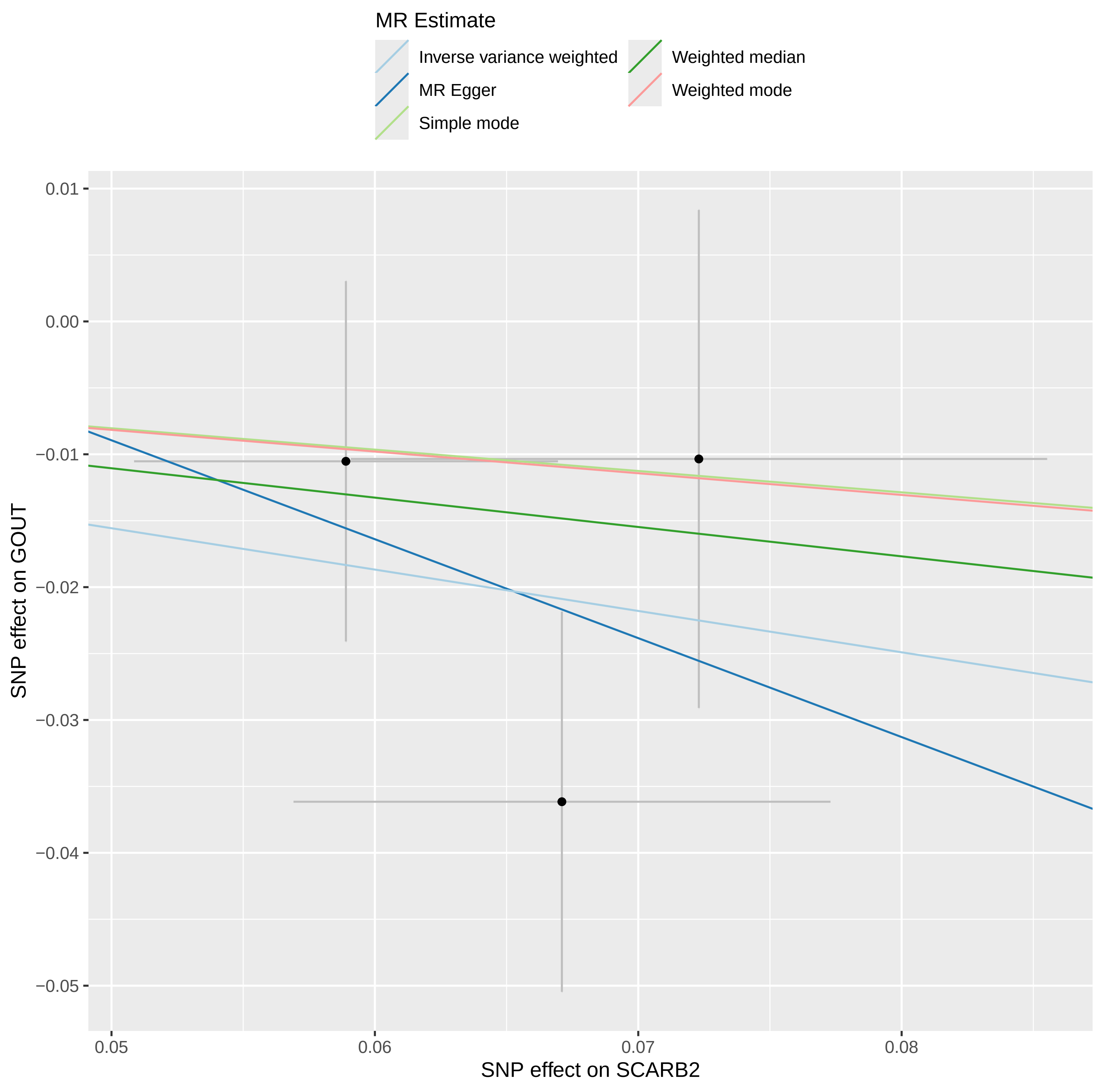

MR Estimate
Inverse variance weighted
MR Egger
Simple mode
Weighted median
Weighted mode
0.01
0.00
−0.01
−0.02
−0.03
−0.04
−0.05
SNP effect on GOUT
0.05
0.06
0.07
0.08
SNP effect on SCARB2

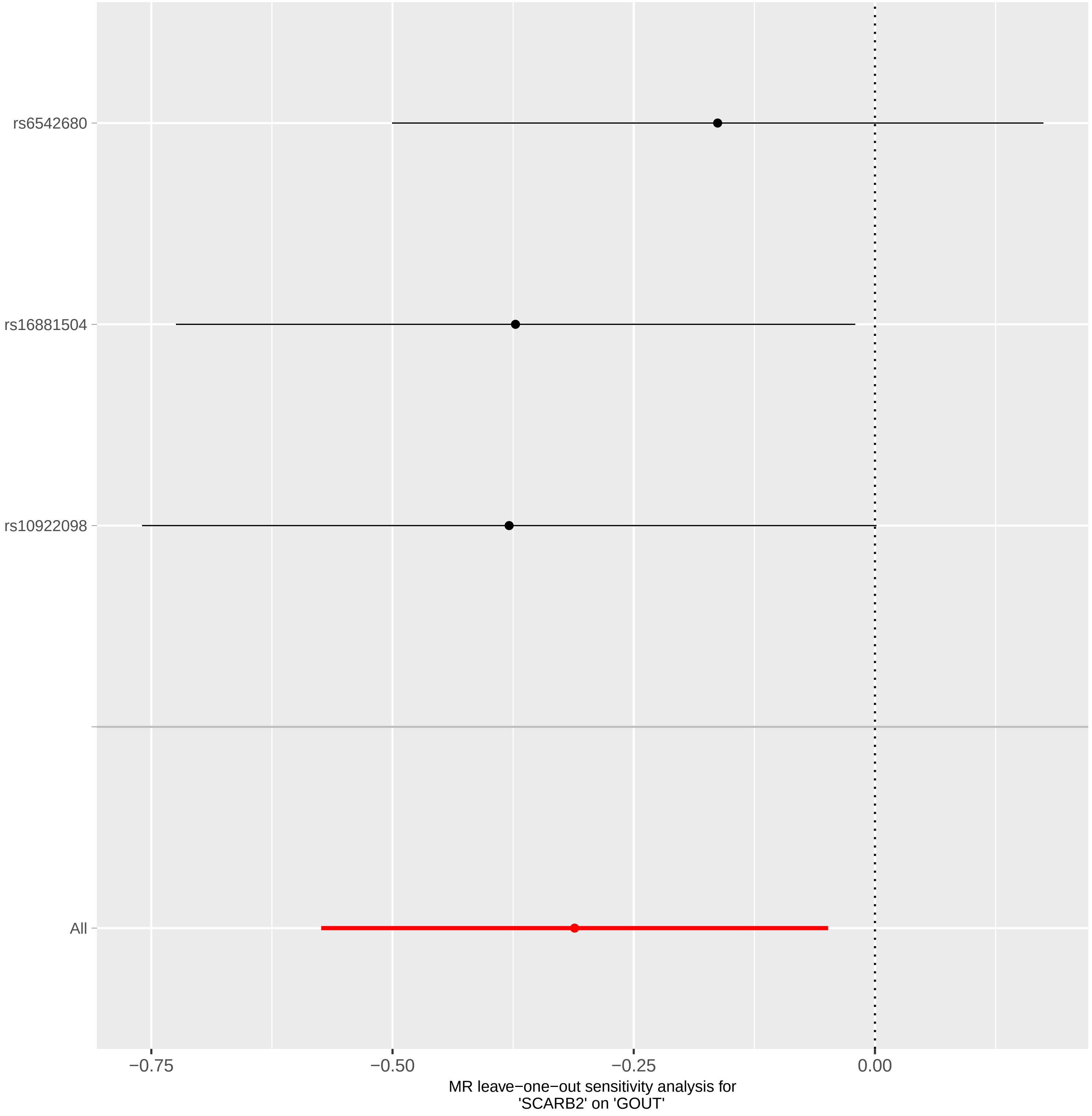
rs6542680
rs16881504
rs10922098
All
−0.75
−0.50
−0.25
0.00
MR leave−one−out sensitivity analysis for
'SCARB2' on 'GOUT'

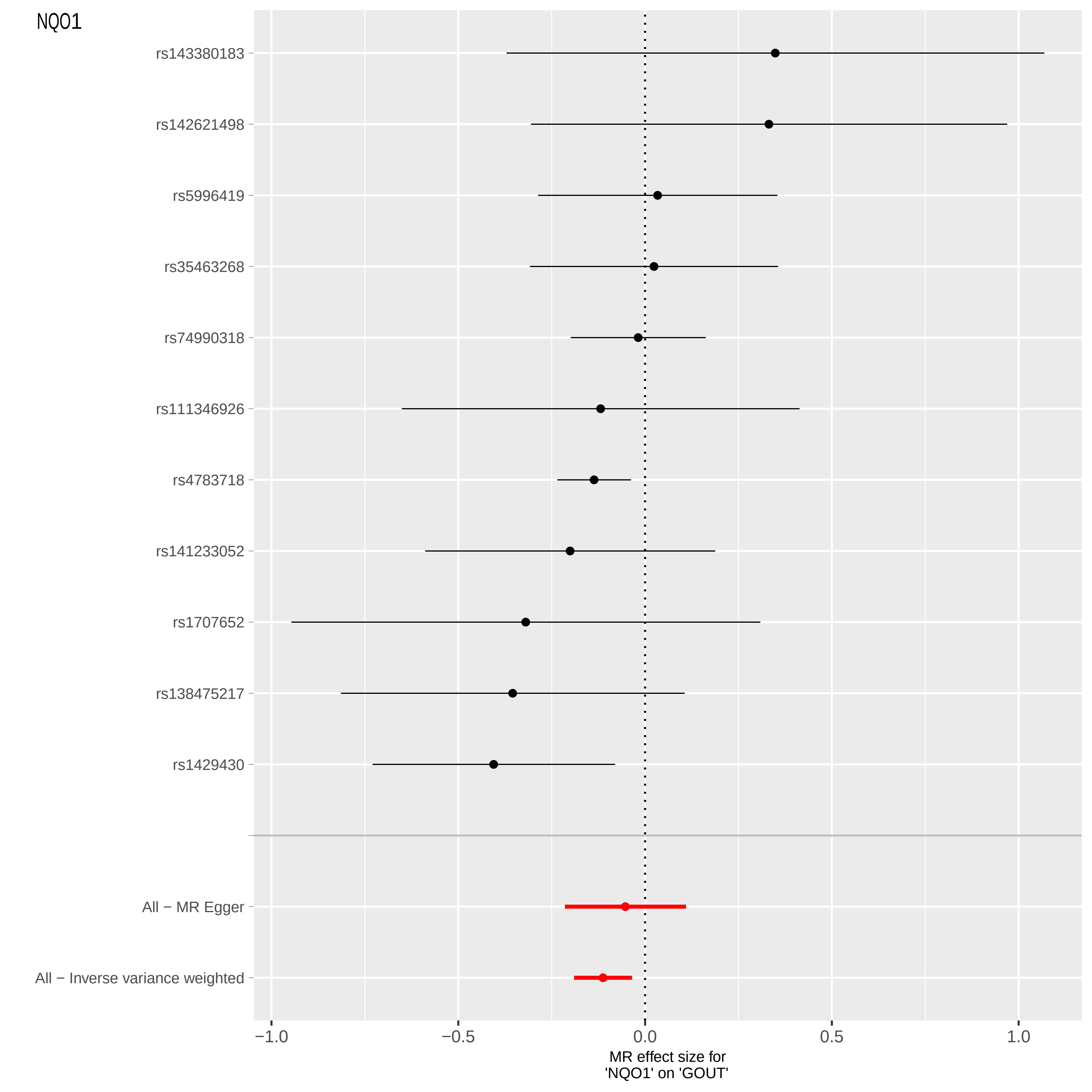
NQO1
rs143380183
rs142621498
rs5996419
rs35463268
rs74990318
rs111346926
rs4783718
rs141233052
rs1707652
rs138475217
rs1429430
All − MR Egger
All − Inverse variance weighted
−1.0
−0.5
0.0
0.5
1.0
MR effect size for
'NQO1' on 'GOUT'

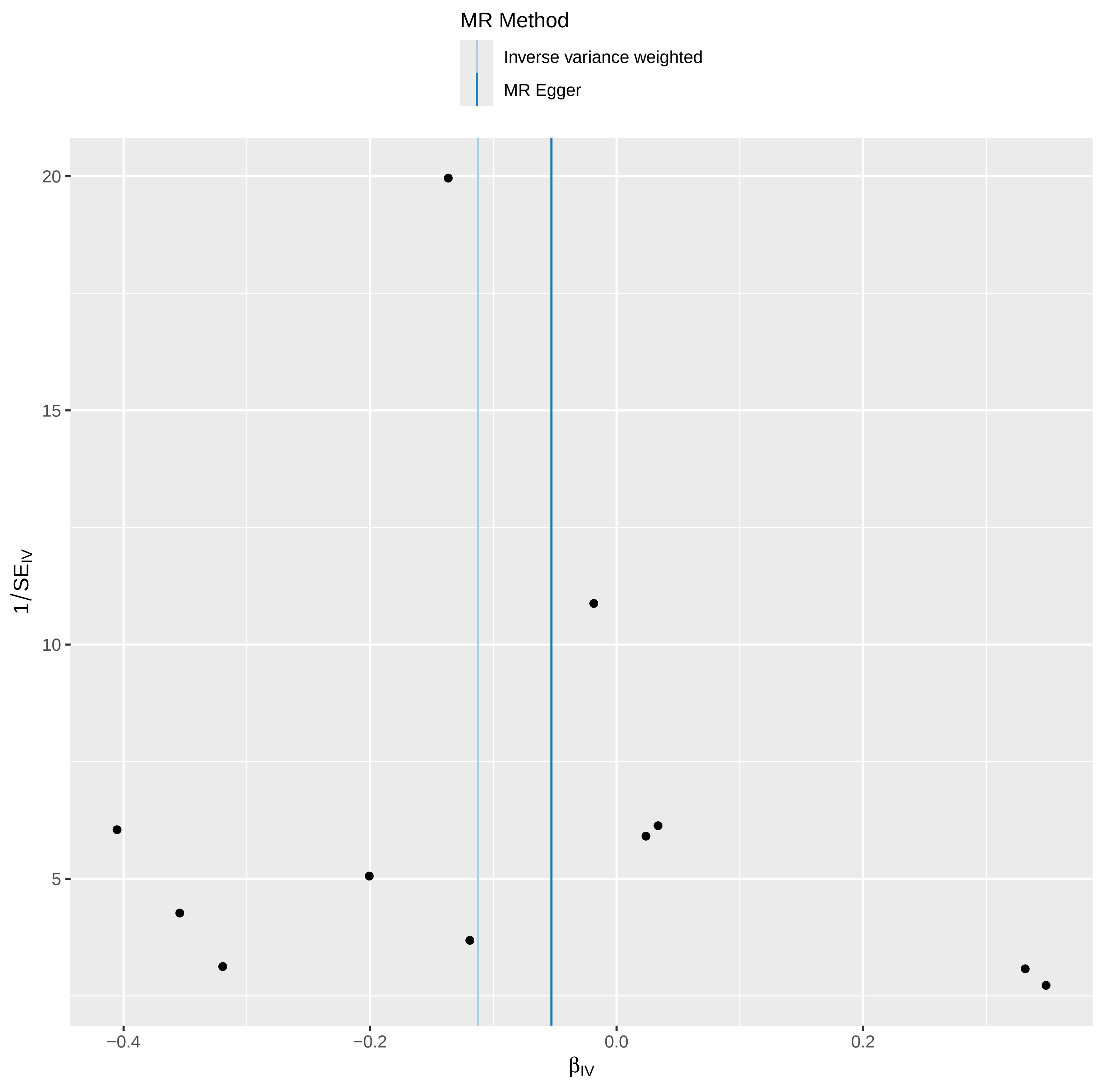
MR Method
Inverse variance weighted
MR Egger
20
15
10
5
1/SE_{IV}
−0.4
−0.2
0.0
0.2
β_{IV}

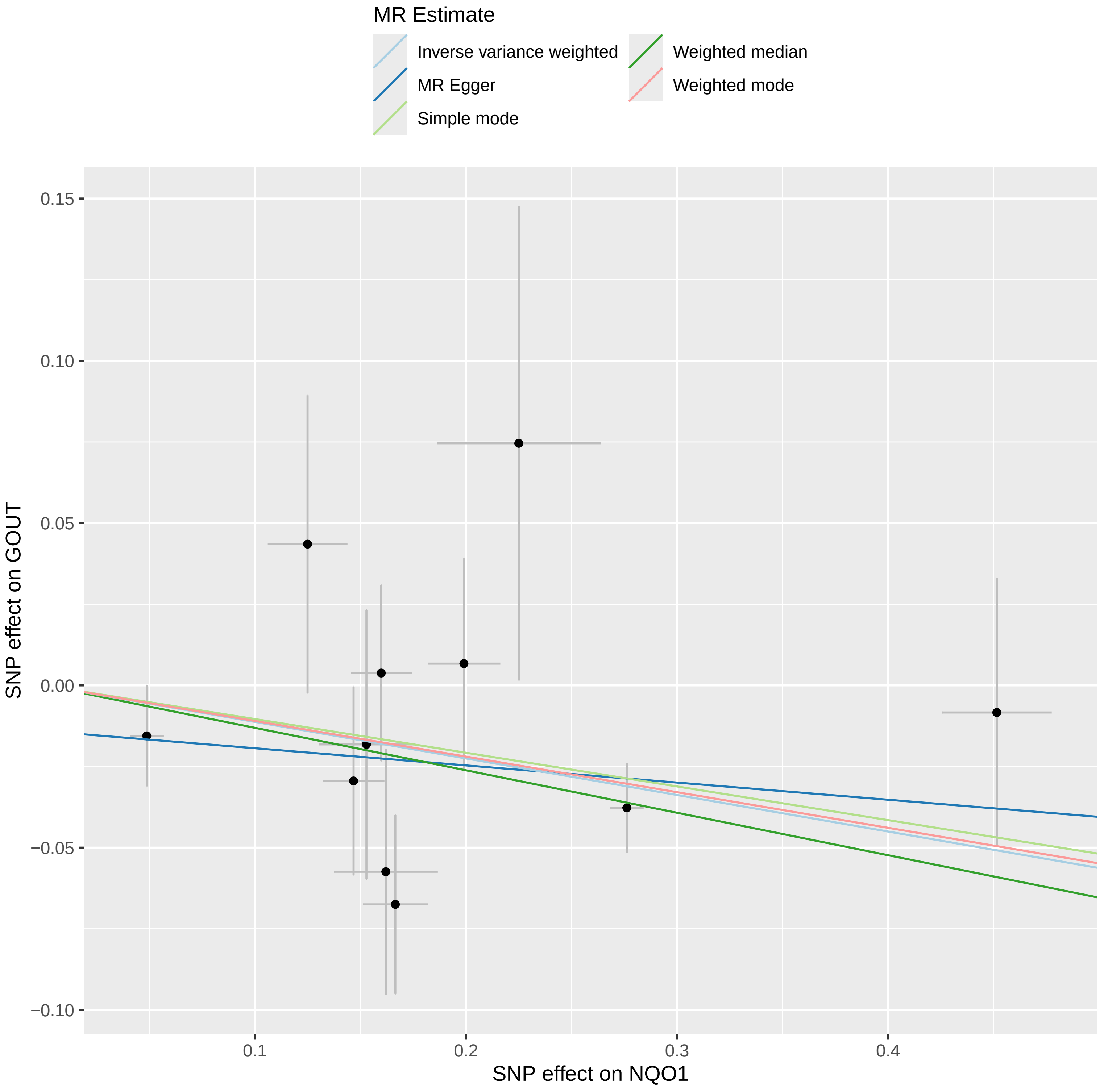
MR Estimate
Inverse variance weighted
MR Egger
Simple mode
Weighted median
Weighted mode
0.15
0.10
0.05
0.00
−0.05
−0.10
0.1
0.2
0.3
0.4
SNP effect on GOUT
SNP effect on NQO1

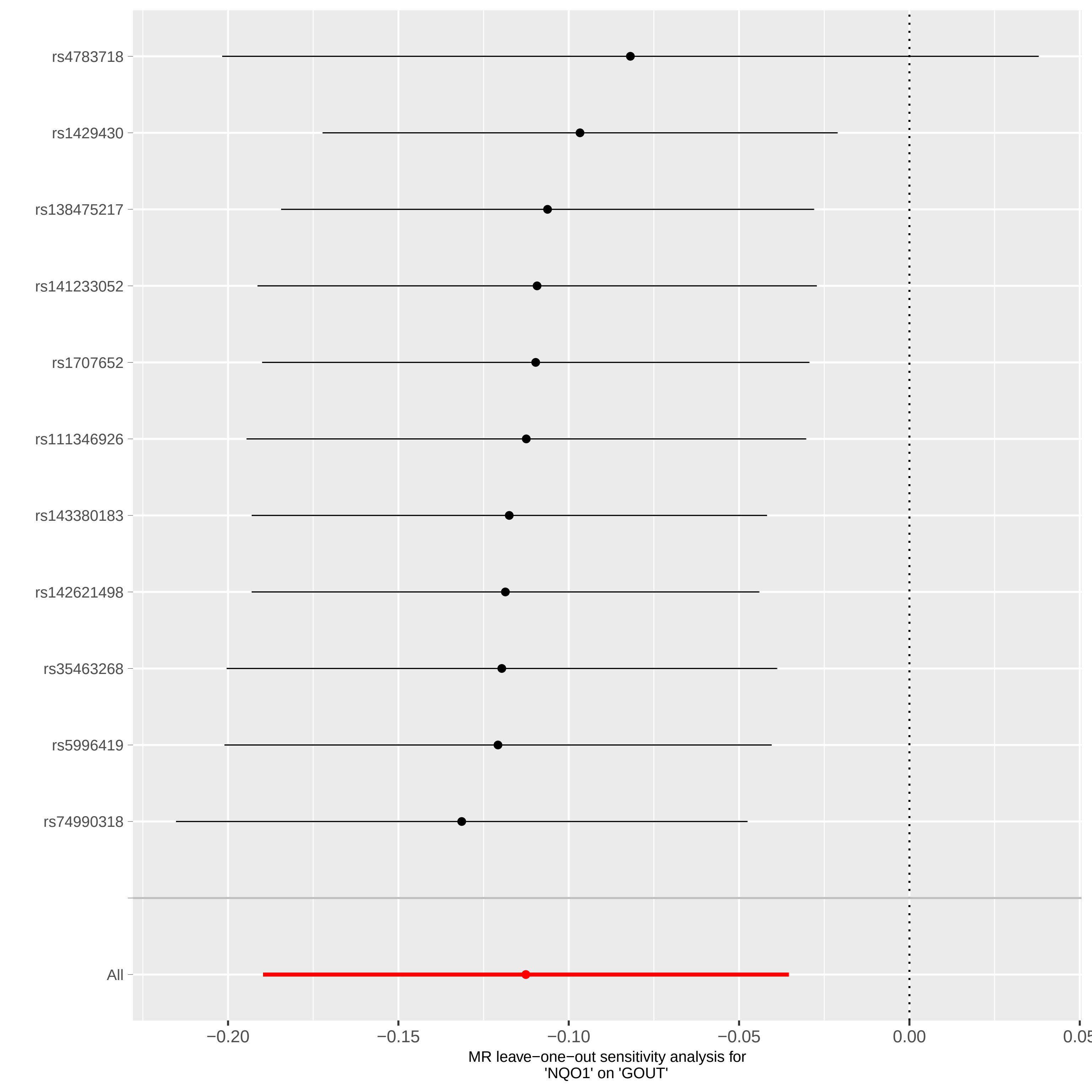
rs4783718
rs1429430
rs138475217
rs141233052
rs1707652
rs111346926
rs143380183
rs142621498
rs35463268
rs5996419
rs74990318
All
−0.20
−0.15
−0.10
−0.05
0.00
0.05
MR leave−one−out sensitivity analysis for
'NQO1' on 'GOUT'

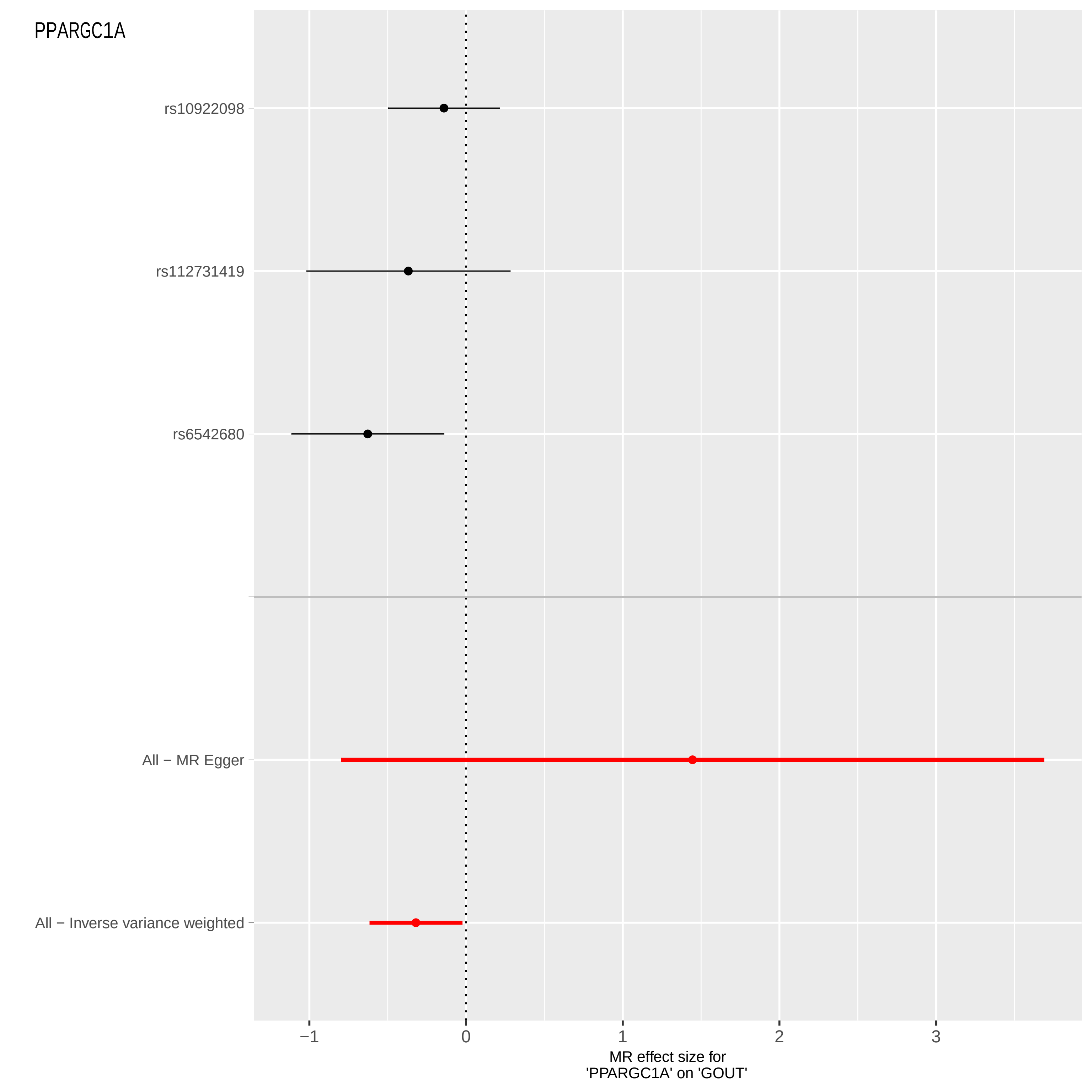
PPARGC1A
rs10922098
rs112731419
rs6542680
All − MR Egger
All − Inverse variance weighted
−1
0
1
2
3
MR effect size for
'PPARGC1A' on 'GOUT'

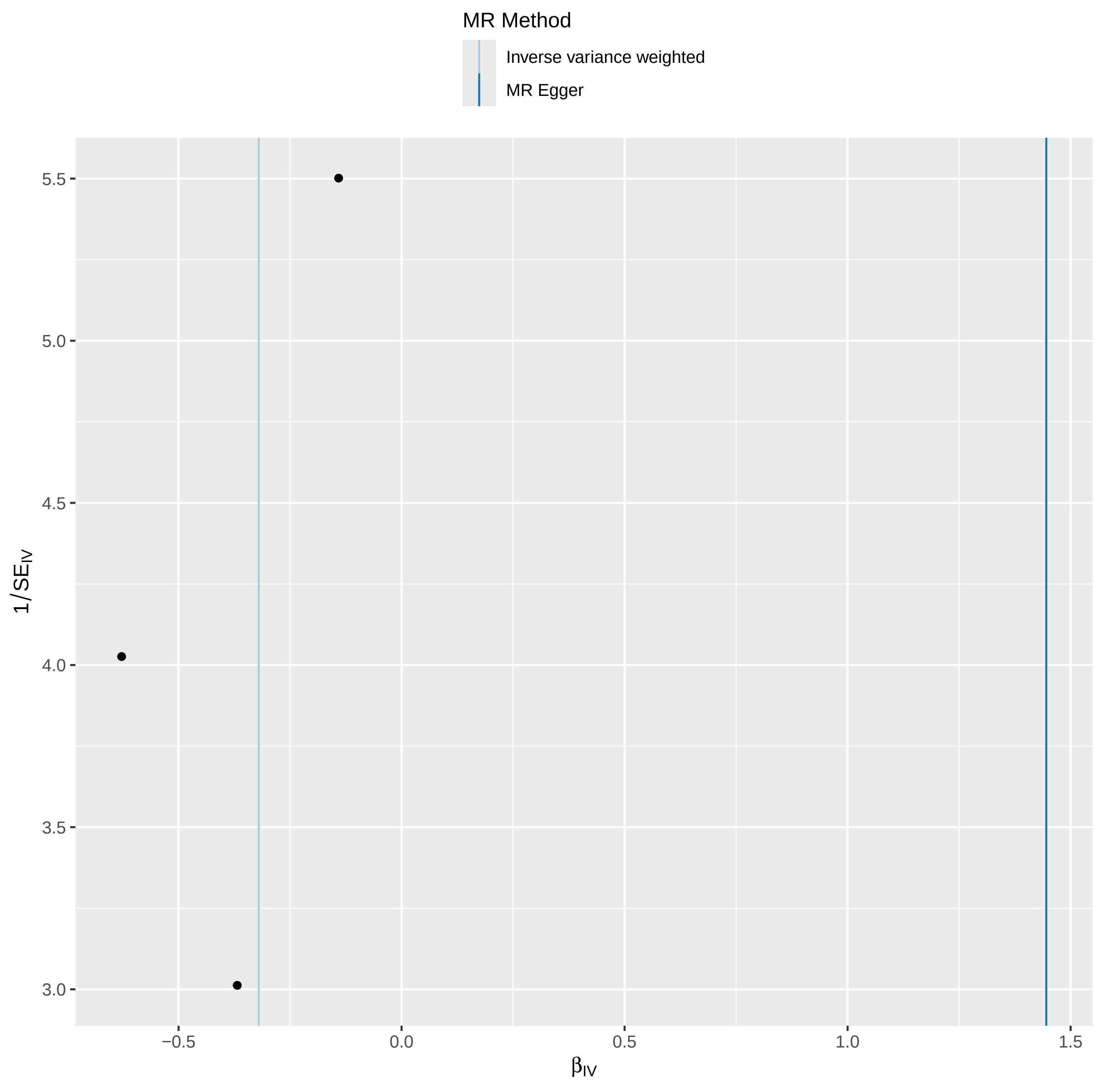

MR Method
Inverse variance weighted
MR Egger
5.5
5.0
4.5
4.0
3.5
3.0
1/SE_IV
−0.5
0.0
0.5
1.0
1.5
β_IV

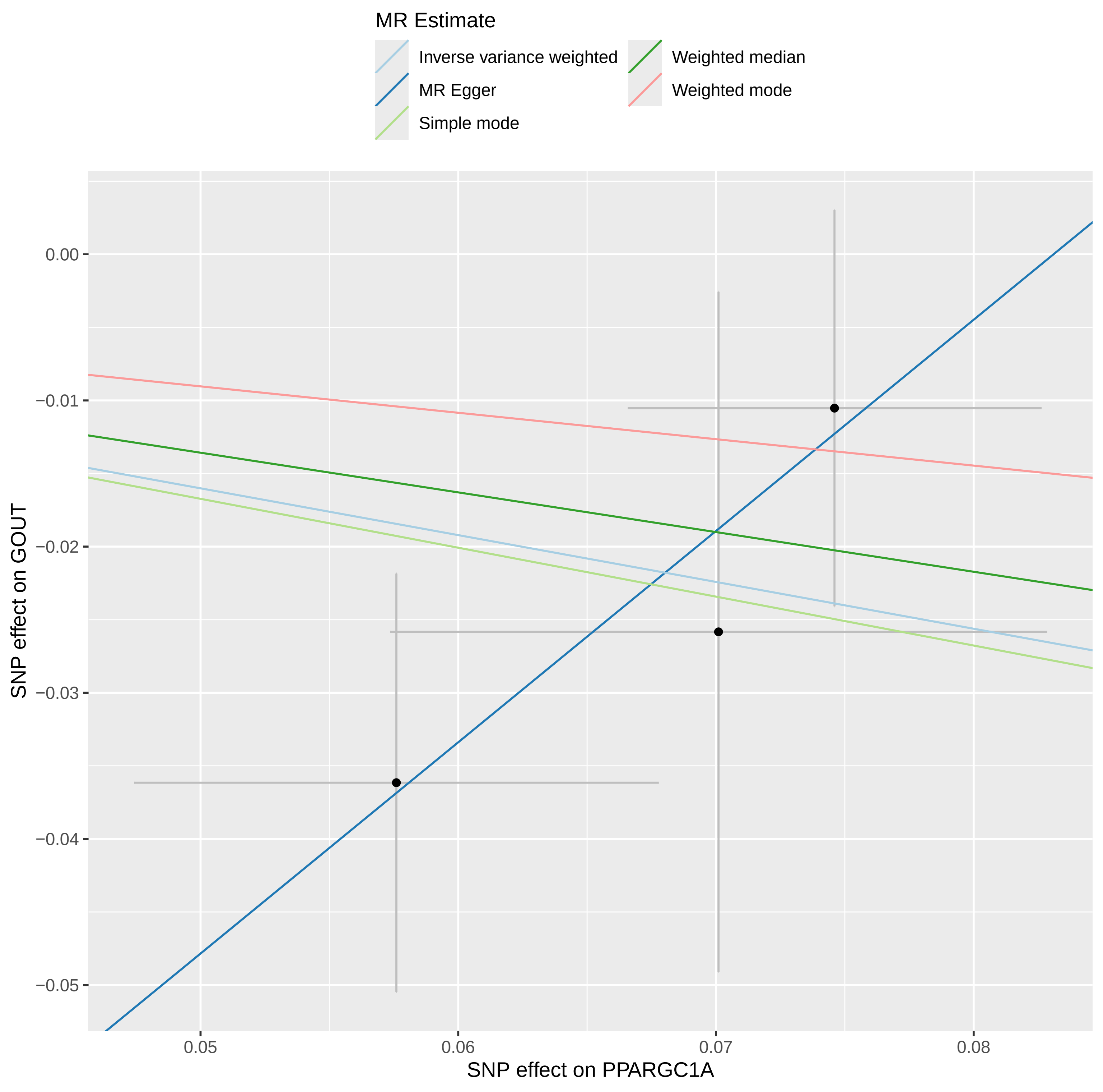
MR Estimate
Inverse variance weighted
MR Egger
Simple mode
Weighted median
Weighted mode
0.00
−0.01
−0.02
−0.03
−0.04
−0.05
SNP effect on GOUT
0.05
0.06
0.07
0.08
SNP effect on PPARGC1A

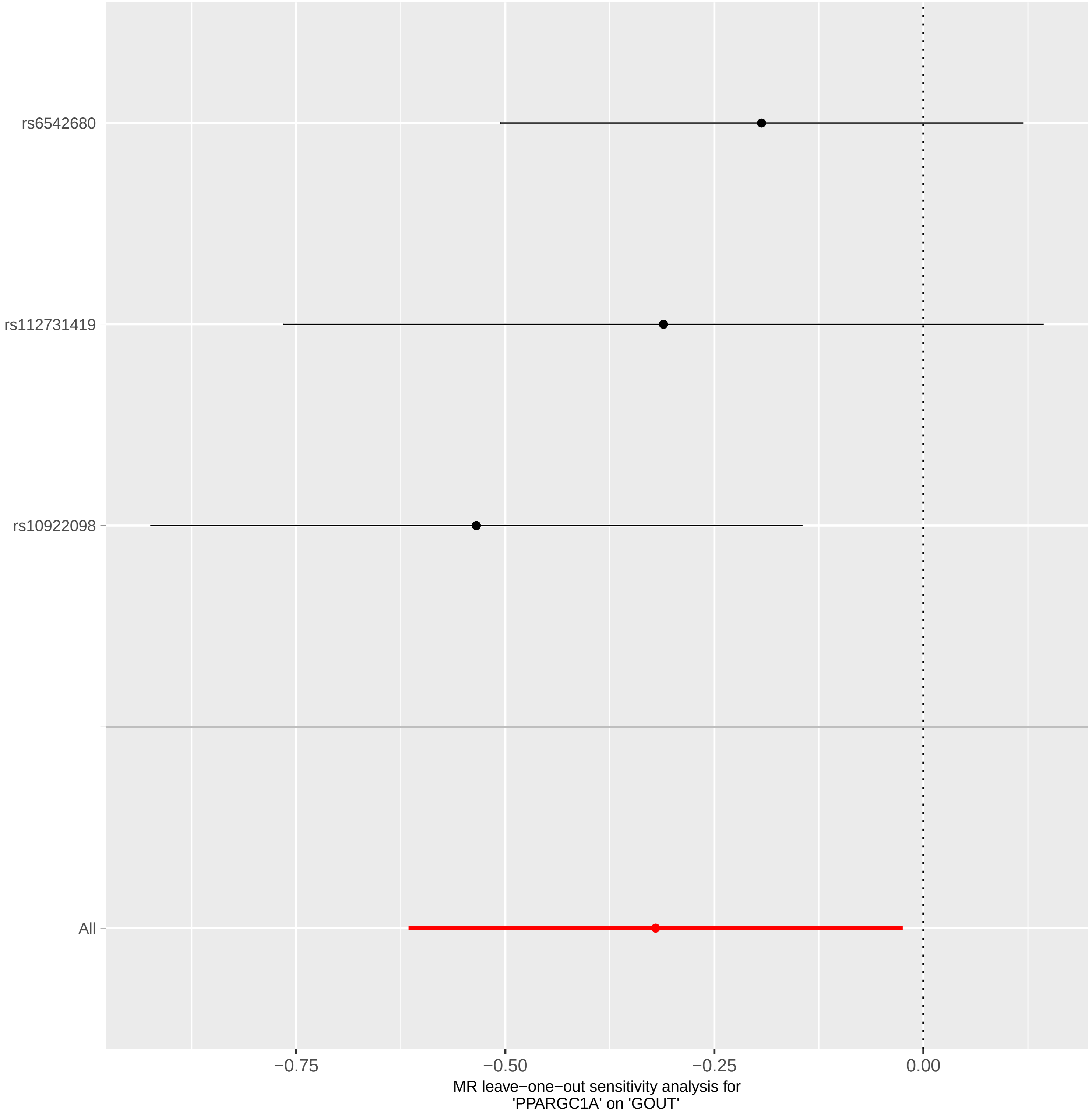

rs6542680
rs112731419
rs10922098
All
−0.75
−0.50
−0.25
0.00
MR leave−one−out sensitivity analysis for
'PPARGC1A' on 'GOUT'

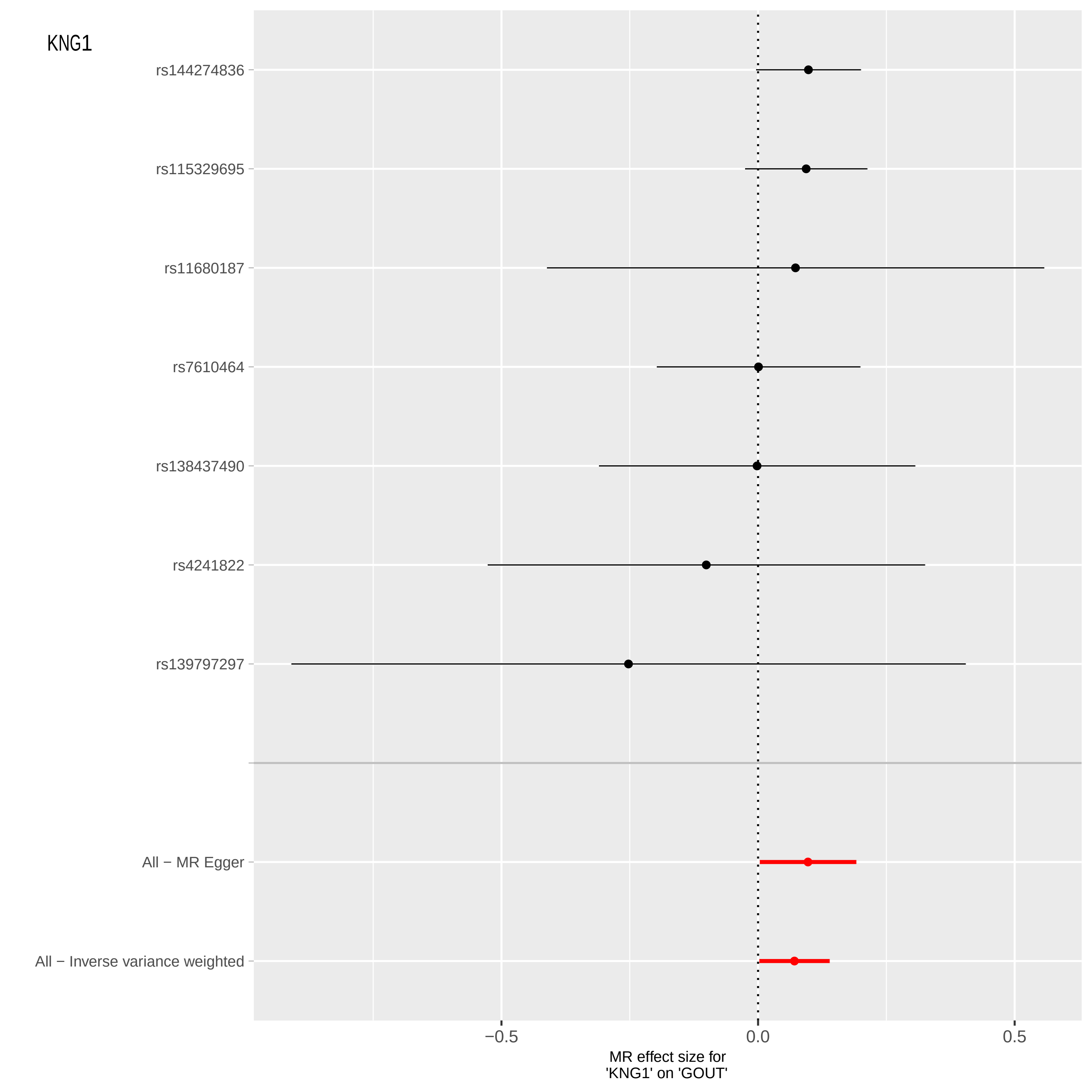

KNG1
rs144274836
rs115329695
rs11680187
rs7610464
rs138437490
rs4241822
rs139797297
All − MR Egger
All − Inverse variance weighted
−0.5
0.0
0.5
MR effect size for
'KNG1' on 'GOUT'

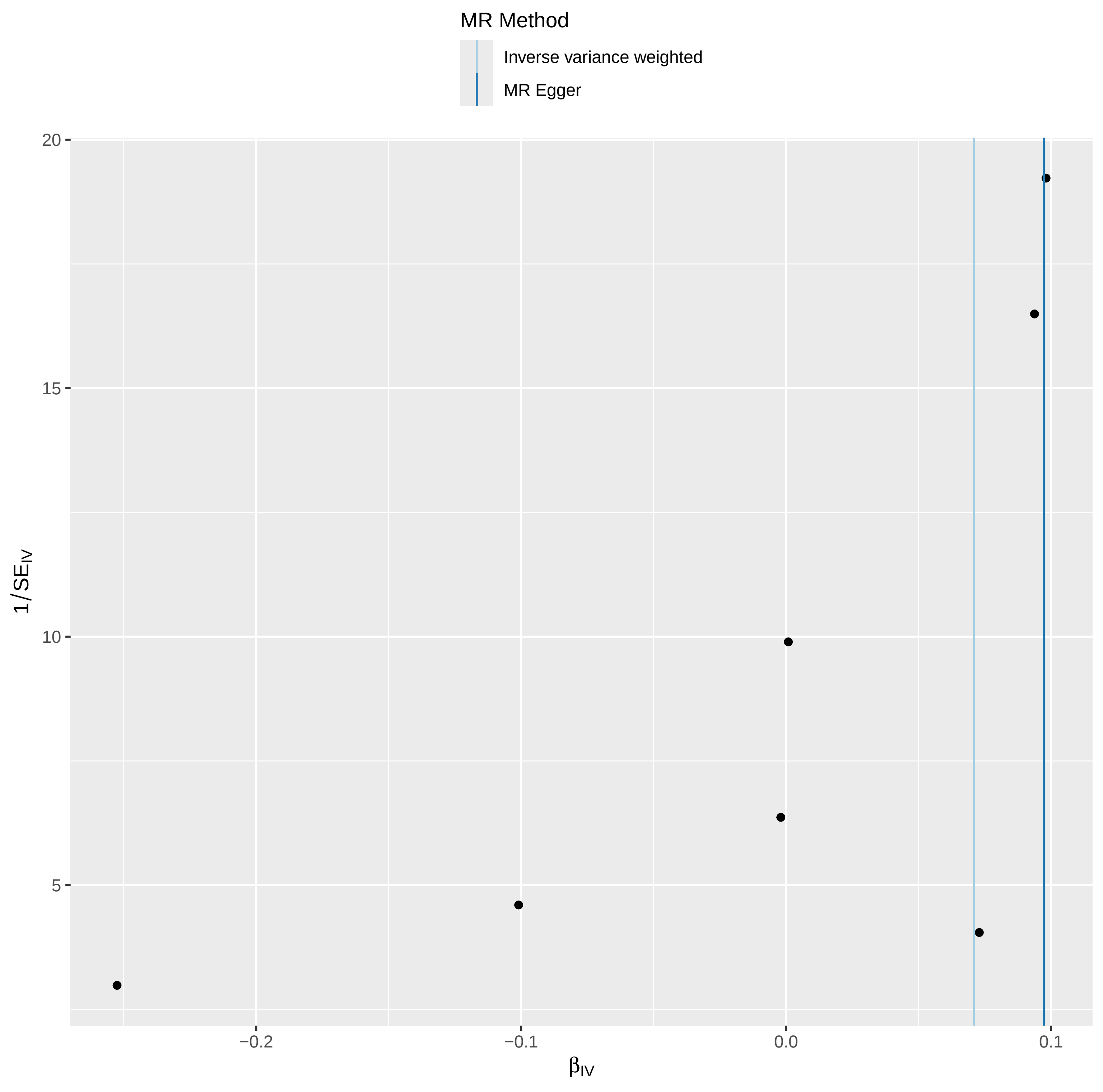

MR Method
Inverse variance weighted
MR Egger
20
15
10
5
1/SE_IV
−0.2
−0.1
0.0
0.1
β_IV

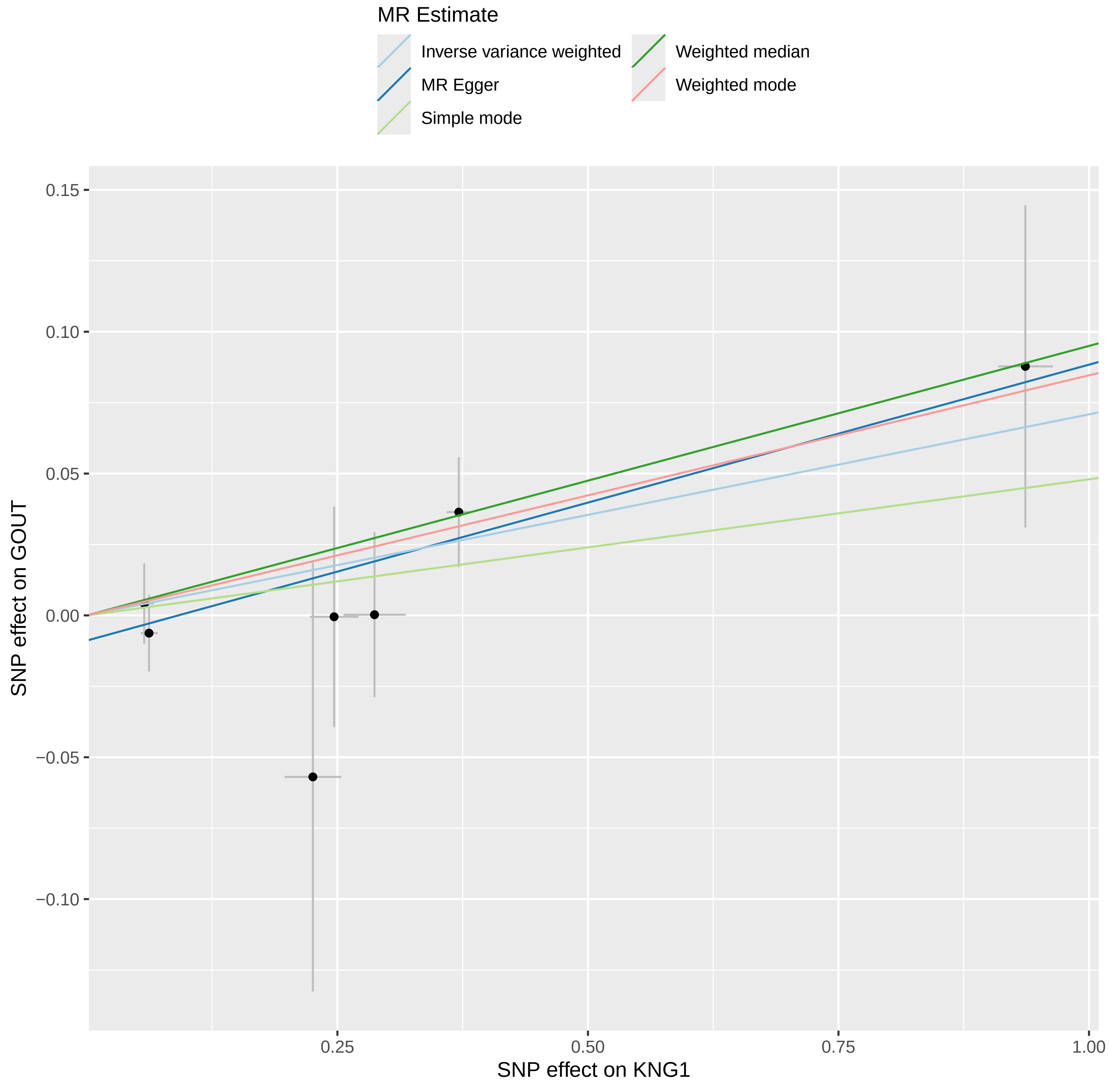

MR Estimate
Inverse variance weighted
MR Egger
Simple mode
Weighted median
Weighted mode
0.15
0.10
0.05
0.00
−0.05
−0.10
0.25
0.50
0.75
1.00
SNP effect on GOUT
SNP effect on KNG1

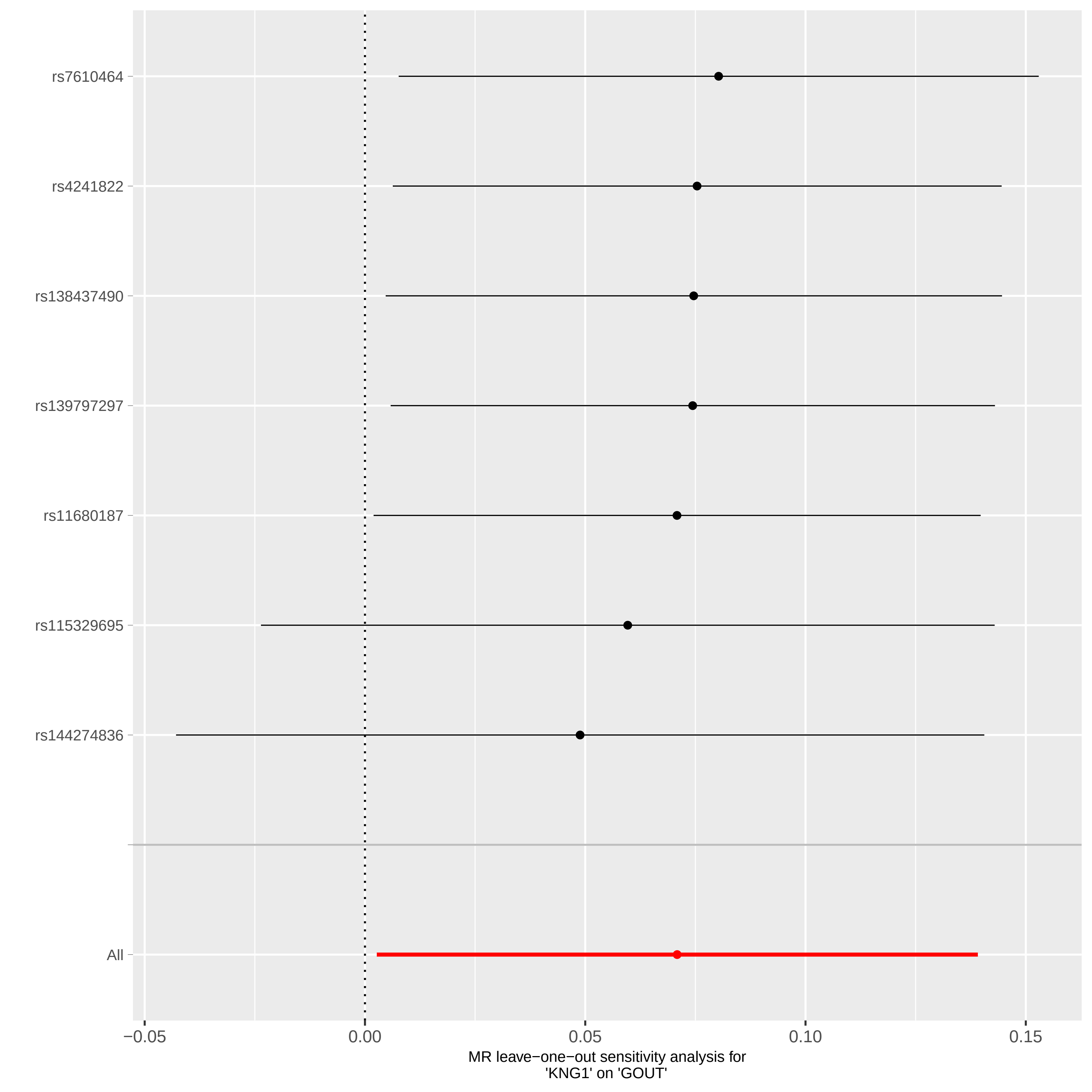

rs7610464
rs4241822
rs138437490
rs139797297
rs11680187
rs115329695
rs144274836
All
−0.05
0.00
0.05
0.10
0.15
MR leave−one−out sensitivity analysis for
'KNG1' on 'GOUT'

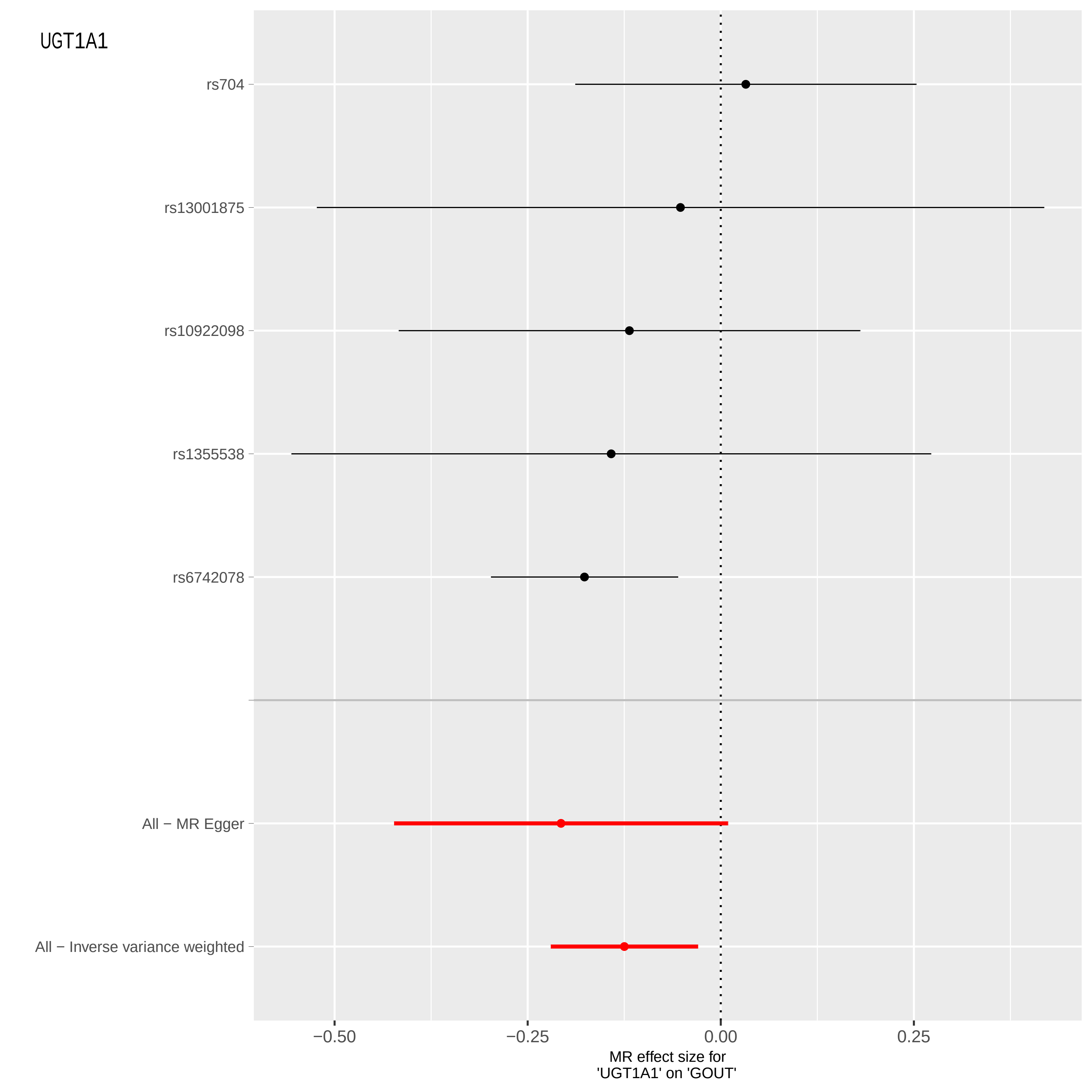

UGT1A1
rs704
rs13001875
rs10922098
rs1355538
rs6742078
All − MR Egger
All − Inverse variance weighted
−0.50
−0.25
0.00
0.25
MR effect size for
'UGT1A1' on 'GOUT'

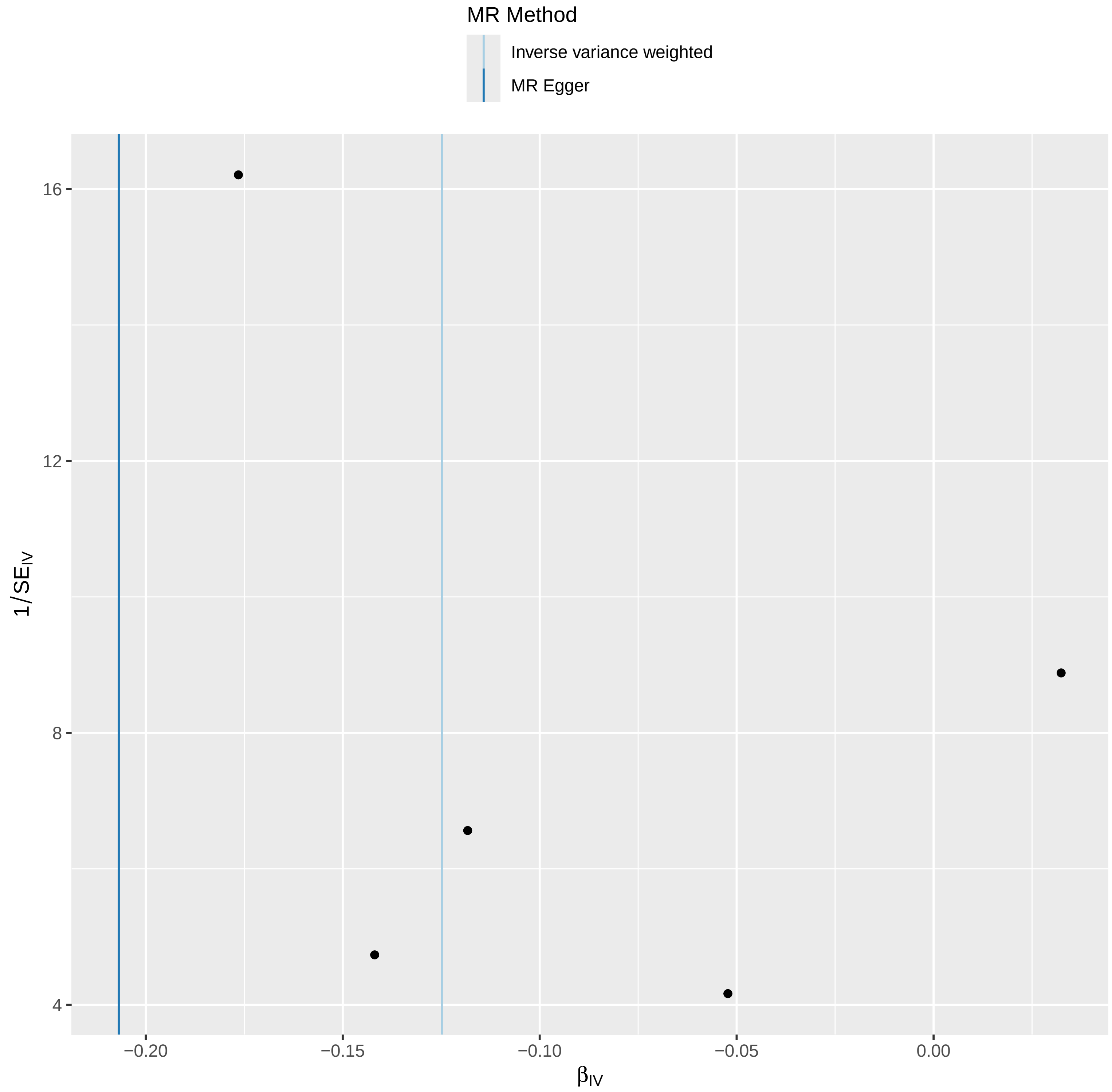
MR Method
Inverse variance weighted
MR Egger
16
12
8
4
1/SE_IV
−0.20
−0.15
−0.10
−0.05
0.00
β_IV

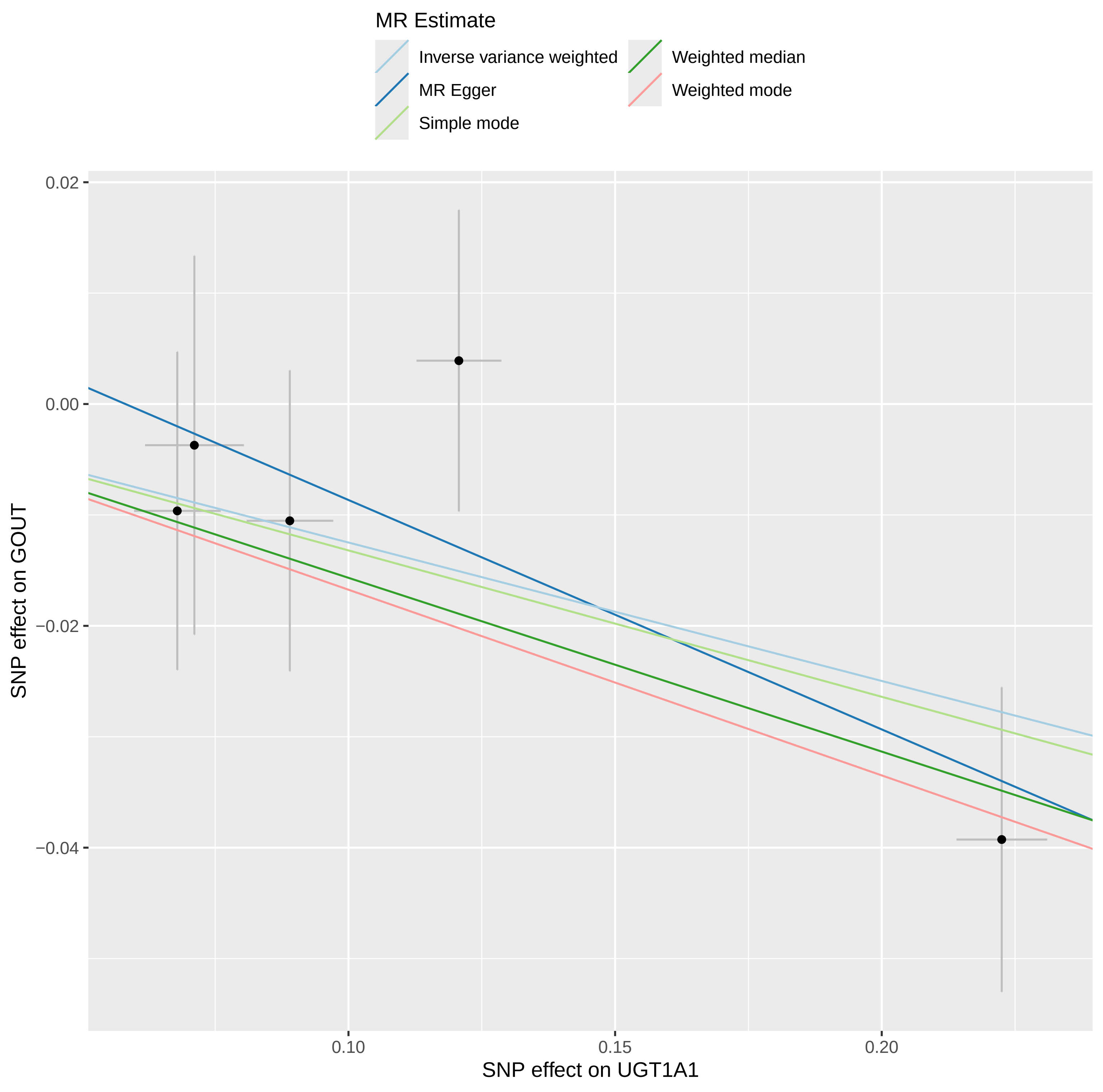
MR Estimate
Inverse variance weighted
MR Egger
Simple mode
Weighted median
Weighted mode
0.02
0.00
−0.02
−0.04
SNP effect on GOUT
0.10
0.15
0.20
SNP effect on UGT1A1

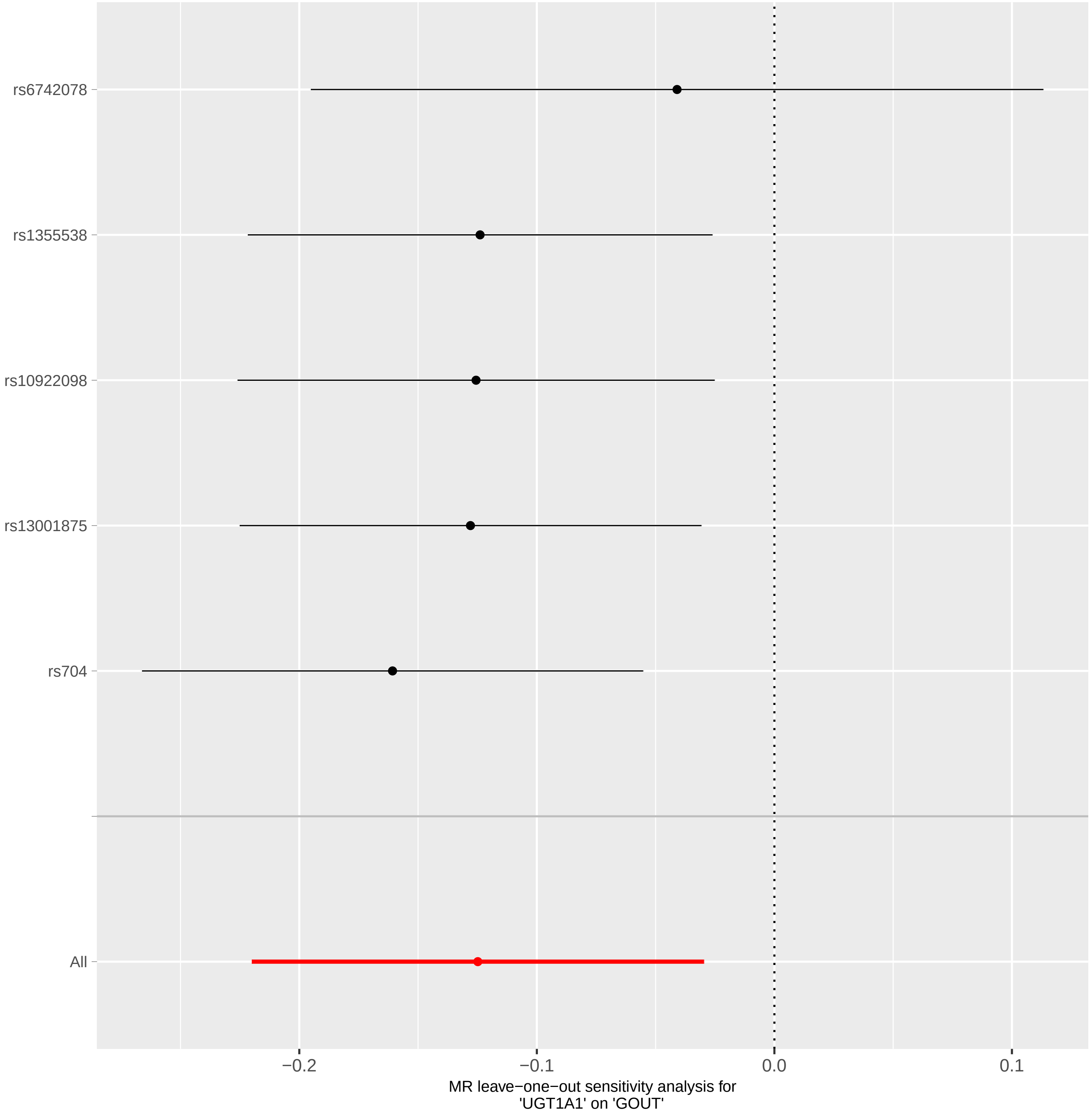

rs6742078
rs1355538
rs10922098
rs13001875
rs704
All
−0.2
−0.1
0.0
0.1
MR leave−one−out sensitivity analysis for
'UGT1A1' on 'GOUT'

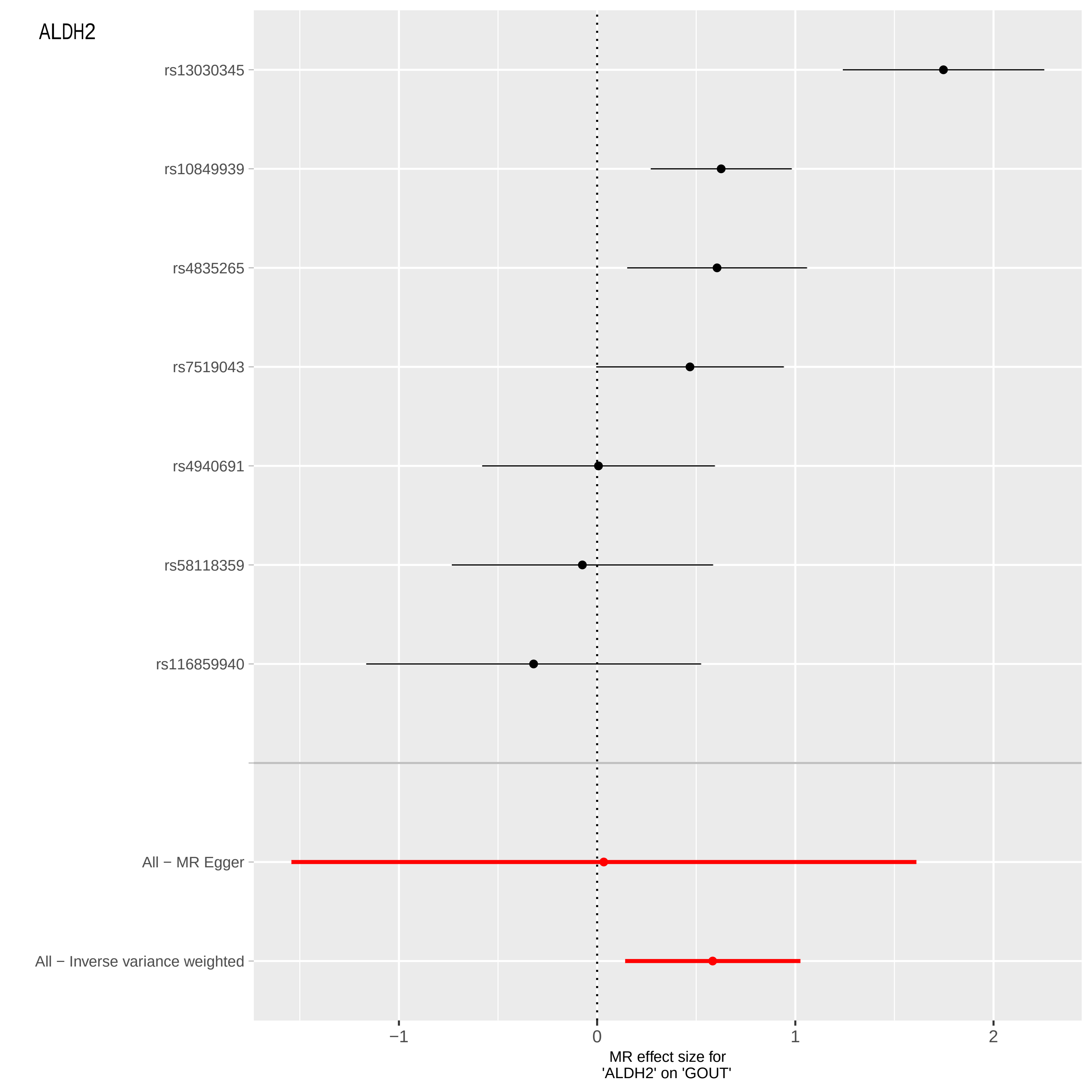
ALDH2
rs13030345
rs10849939
rs4835265
rs7519043
rs4940691
rs58118359
rs116859940
All − MR Egger
All − Inverse variance weighted
−1
0
1
2
MR effect size for
'ALDH2' on 'GOUT'

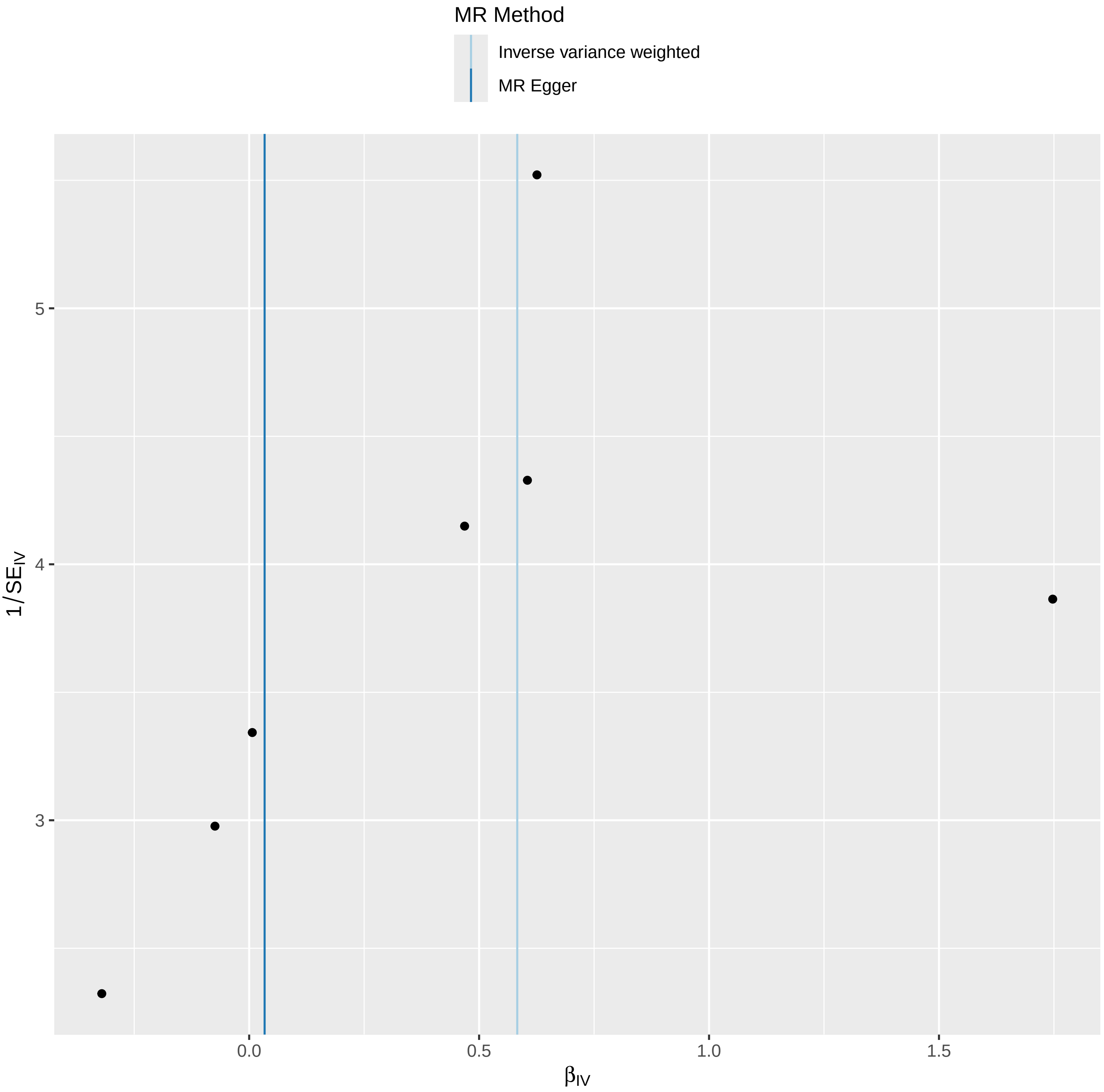
MR Method
Inverse variance weighted
MR Egger
$1/SE_{IV}$
5
4
3
0.0
0.5
1.0
1.5
β_{IV}

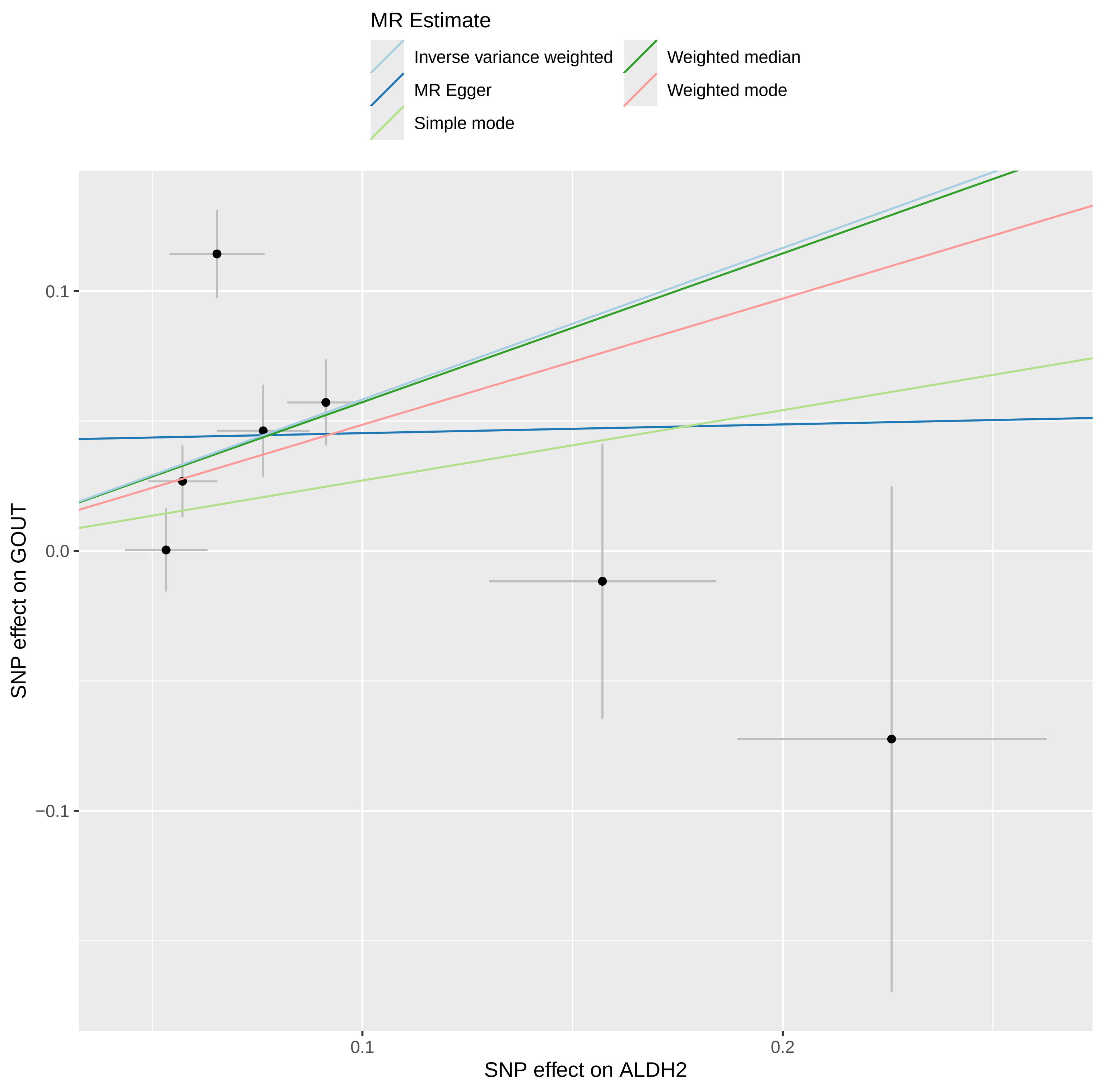

MR Estimate
Inverse variance weighted
MR Egger
Simple mode
Weighted median
Weighted mode
0.1
0.0
−0.1
SNP effect on GOUT
0.1
0.2
SNP effect on ALDH2

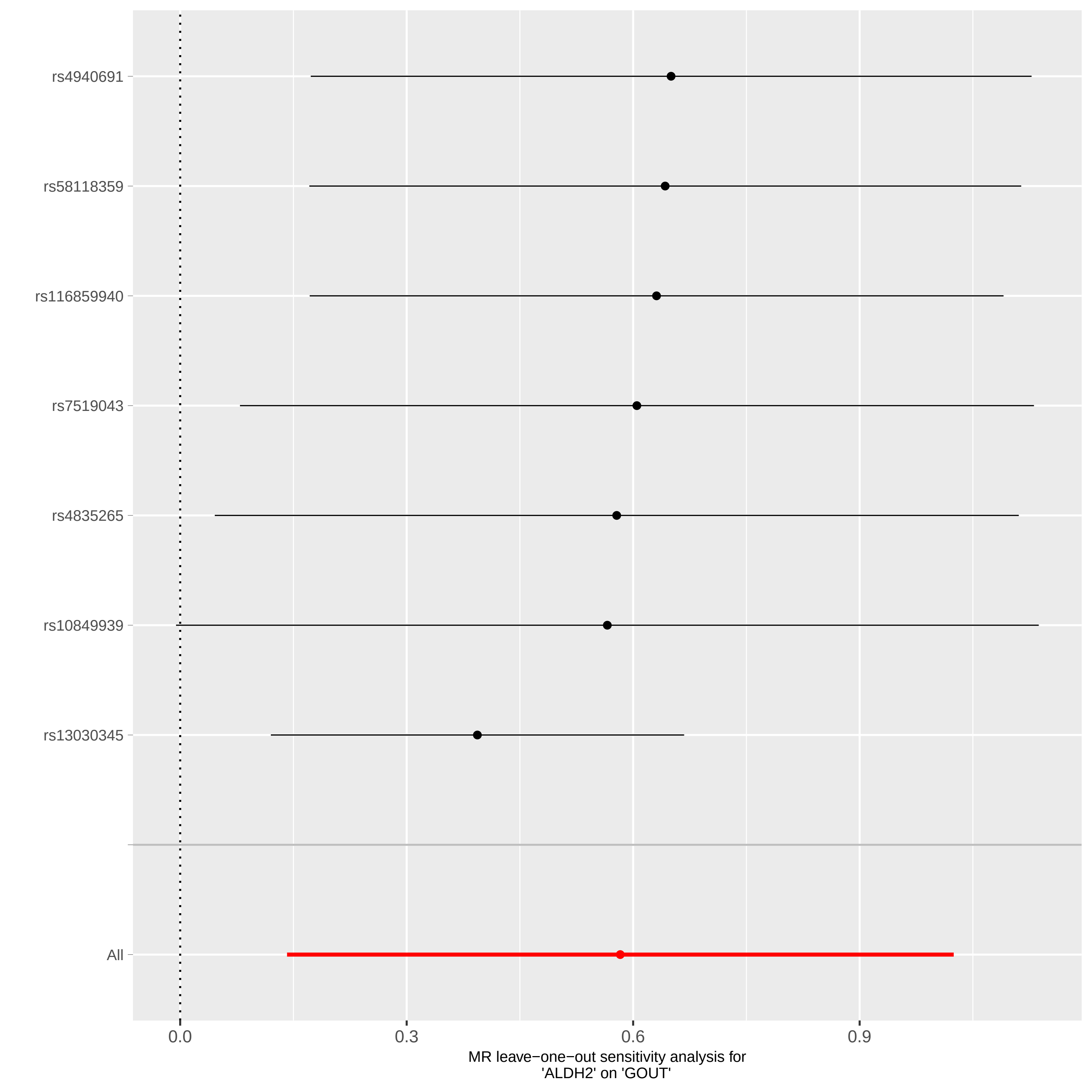
rs4940691
rs58118359
rs116859940
rs7519043
rs4835265
rs10849939
rs13030345
All
0.0
0.3
0.6
0.9
MR leave−one−out sensitivity analysis for
'ALDH2' on 'GOUT'

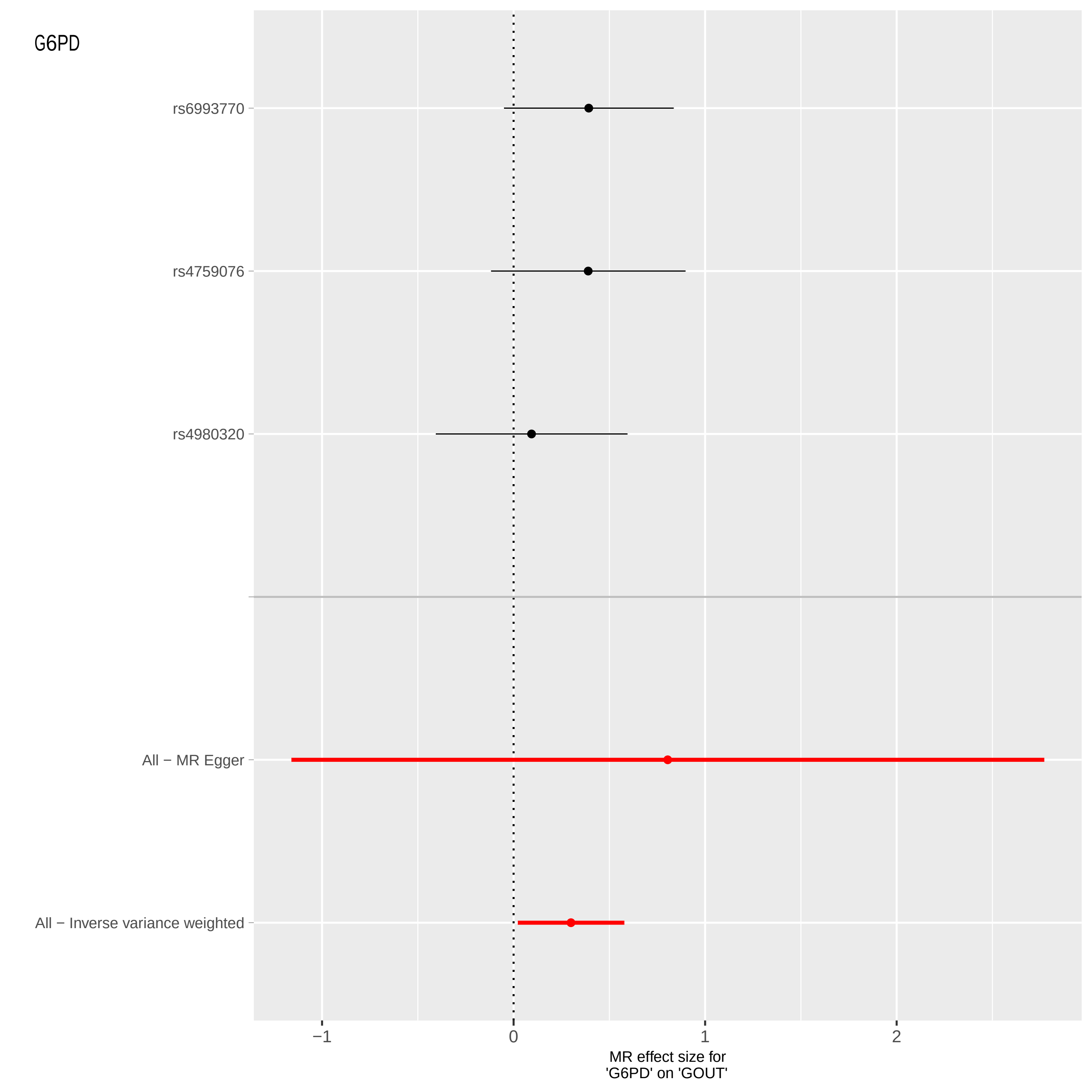

G6PD
rs6993770
rs4759076
rs4980320
All − MR Egger
All − Inverse variance weighted
−1
0
1
2
MR effect size for
'G6PD' on 'GOUT'

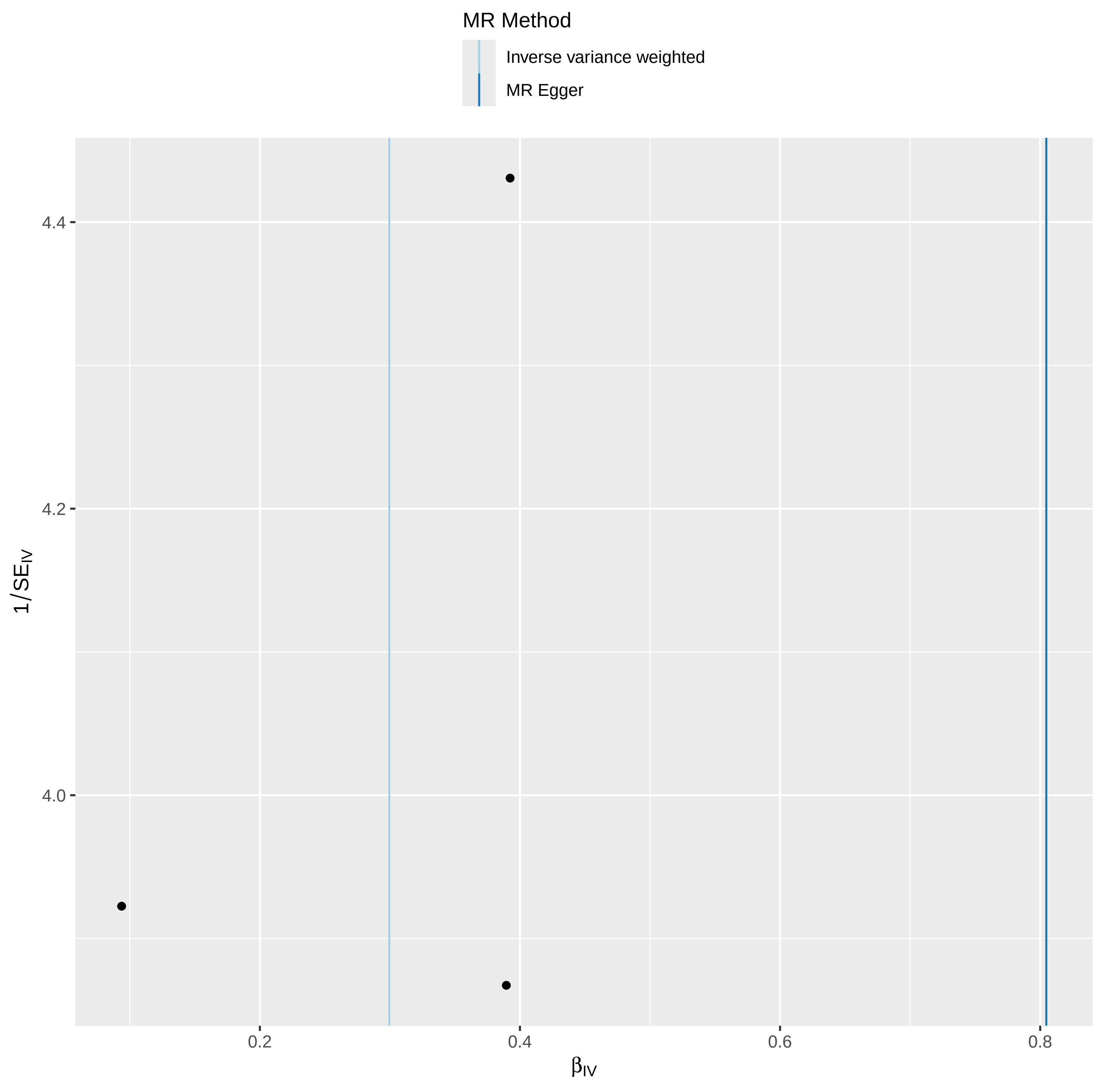
MR Method
Inverse variance weighted
MR Egger
4.4
4.2
4.0
1/SE_IV
0.2
0.4
0.6
0.8
β_IV

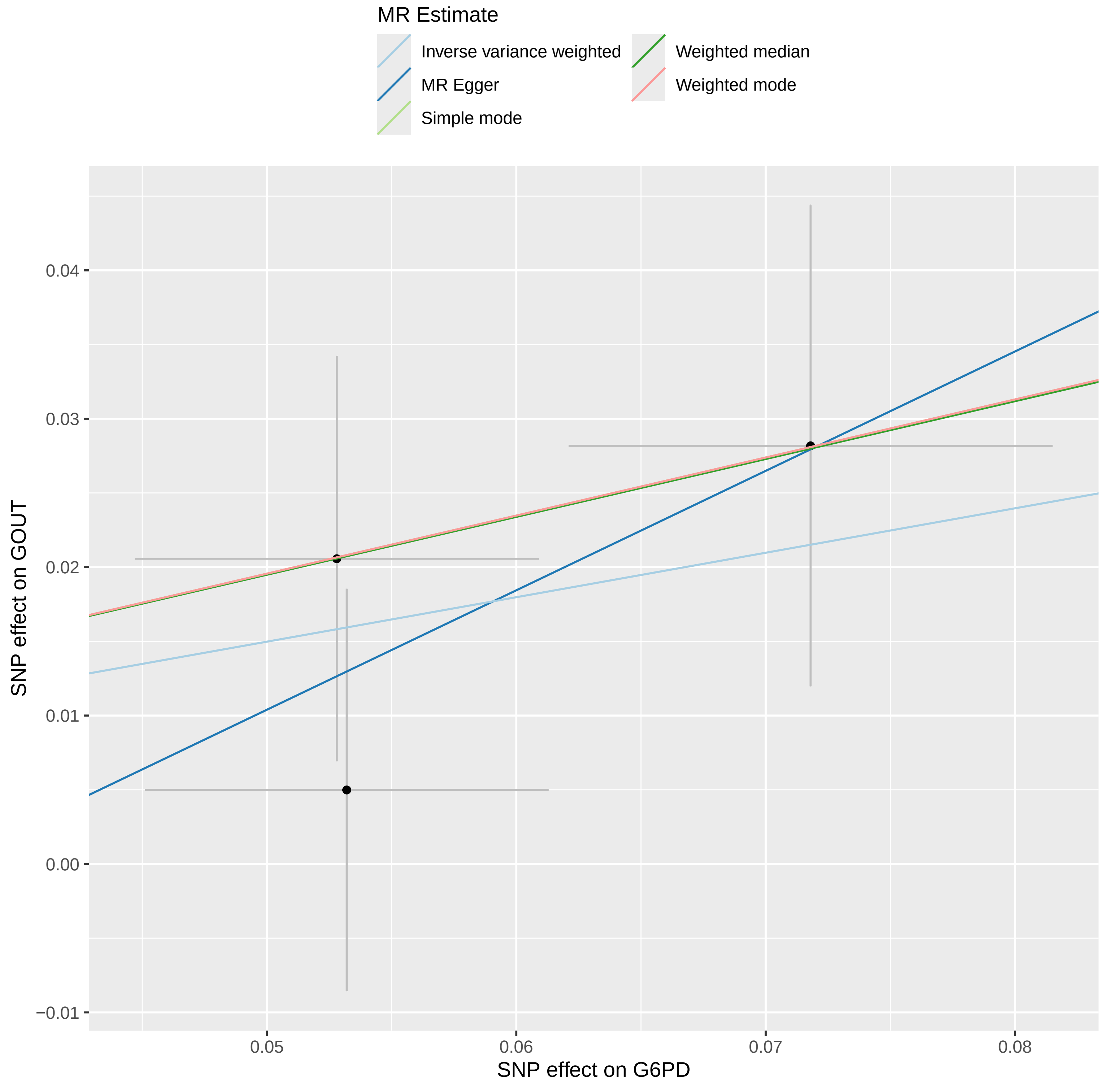
MR Estimate
Inverse variance weighted
MR Egger
Simple mode
Weighted median
Weighted mode
0.04
0.03
0.02
0.01
0.00
−0.01
SNP effect on GOUT
0.05
0.06
0.07
0.08
SNP effect on G6PD

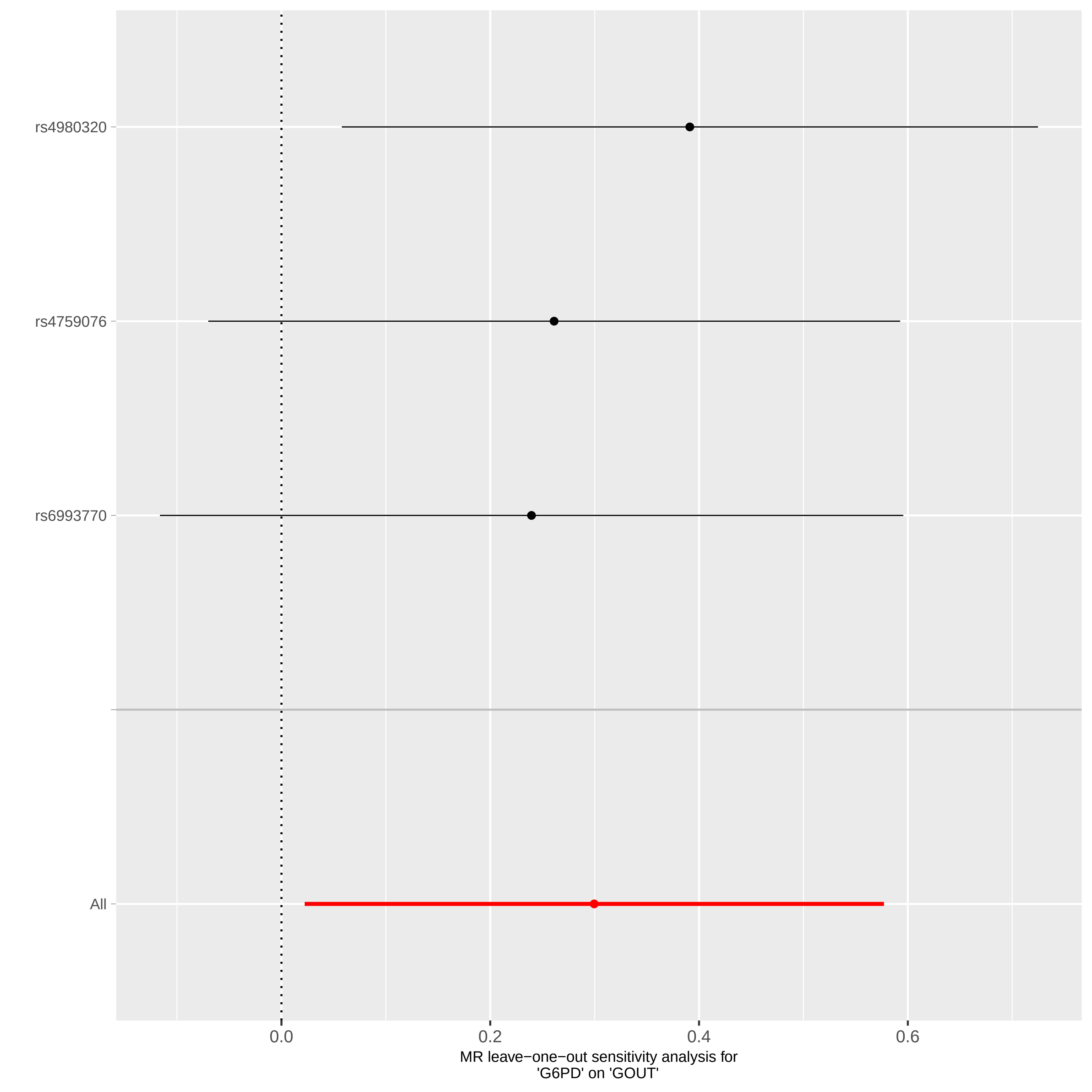

rs4980320
rs4759076
rs6993770
All
0.0
0.2
0.4
0.6
MR leave−one−out sensitivity analysis for
'G6PD' on 'GOUT'

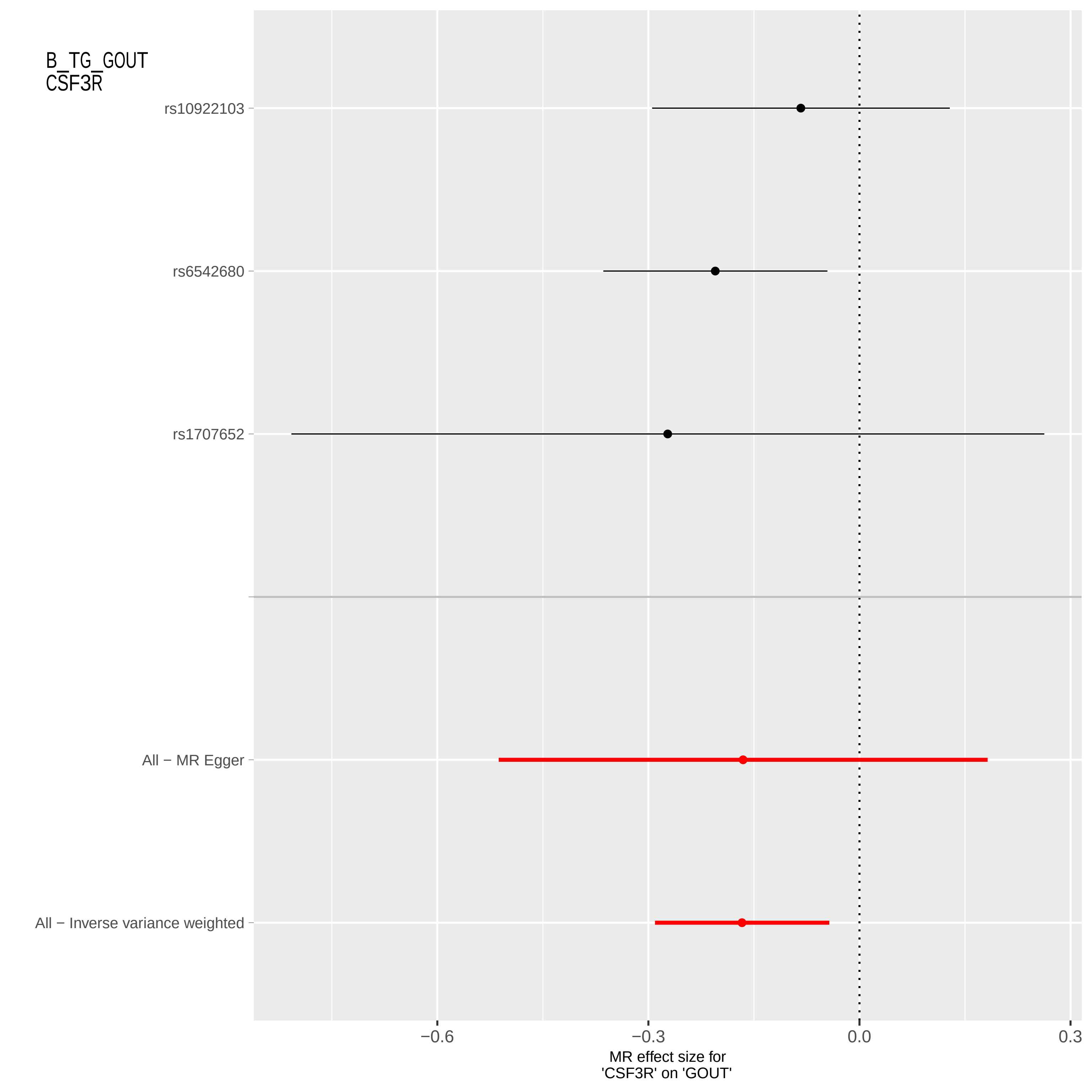

B_TG_GOUT
CSF3R
rs10922103
rs6542680
rs1707652
All − MR Egger
All − Inverse variance weighted
−0.6
−0.3
0.0
0.3
MR effect size for
'CSF3R' on 'GOUT'

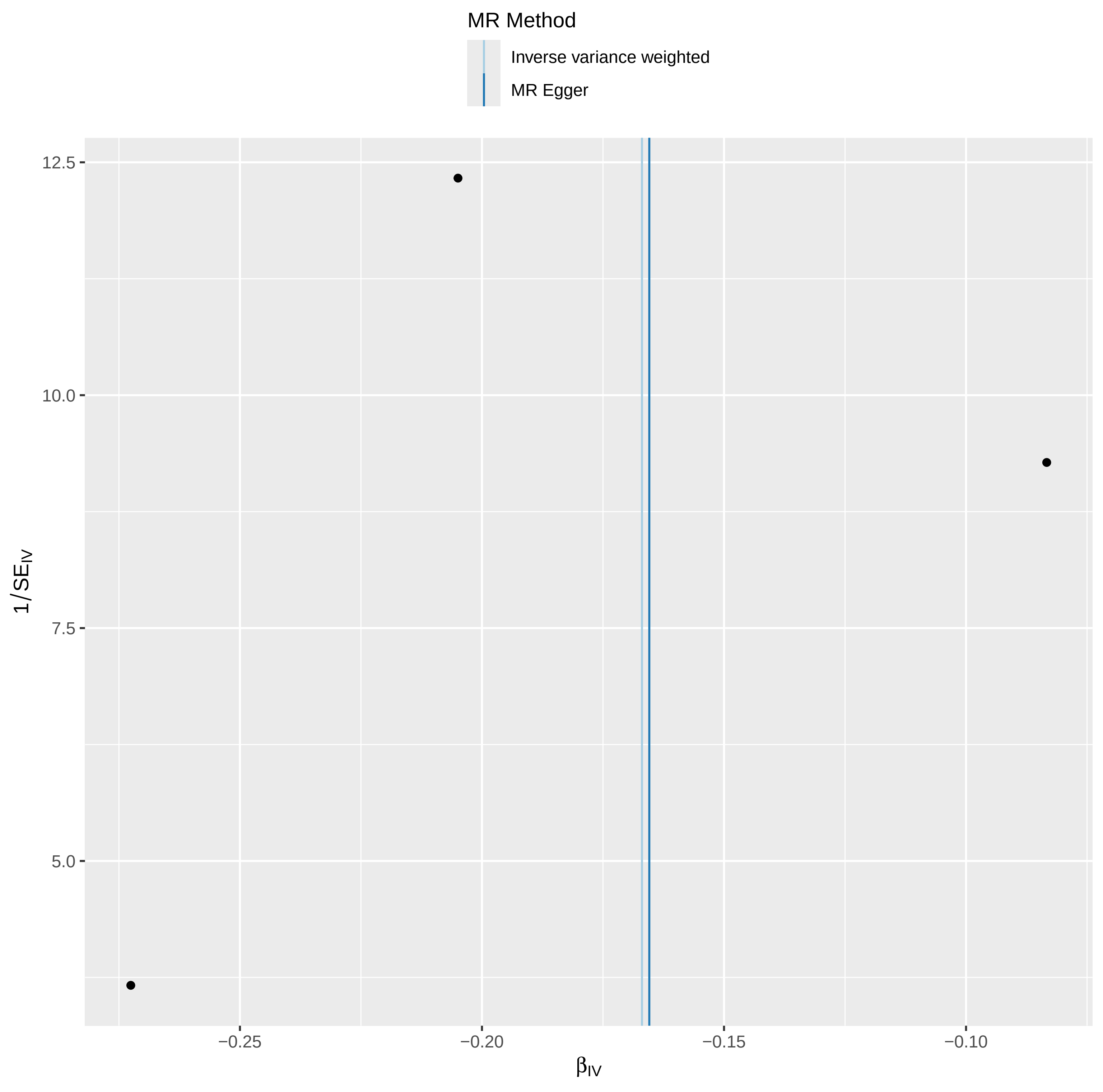
MR Method
Inverse variance weighted
MR Egger
12.5
10.0
7.5
5.0
1/SE_{IV}
−0.25
−0.20
−0.15
−0.10
β_{IV}

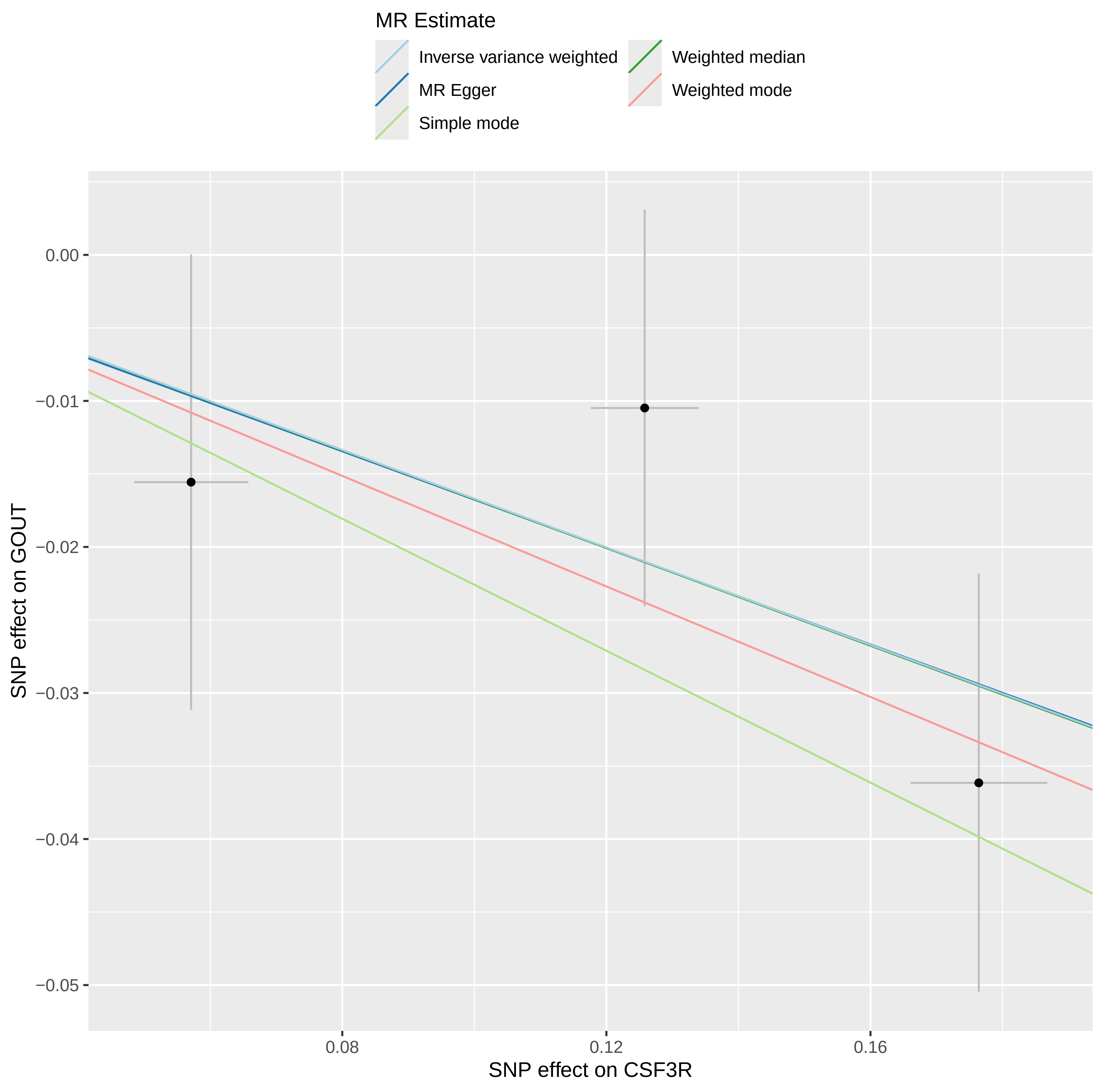
MR Estimate
Inverse variance weighted
MR Egger
Simple mode
Weighted median
Weighted mode
0.00
−0.01
−0.02
−0.03
−0.04
−0.05
SNP effect on GOUT
0.08
0.12
0.16
SNP effect on CSF3R

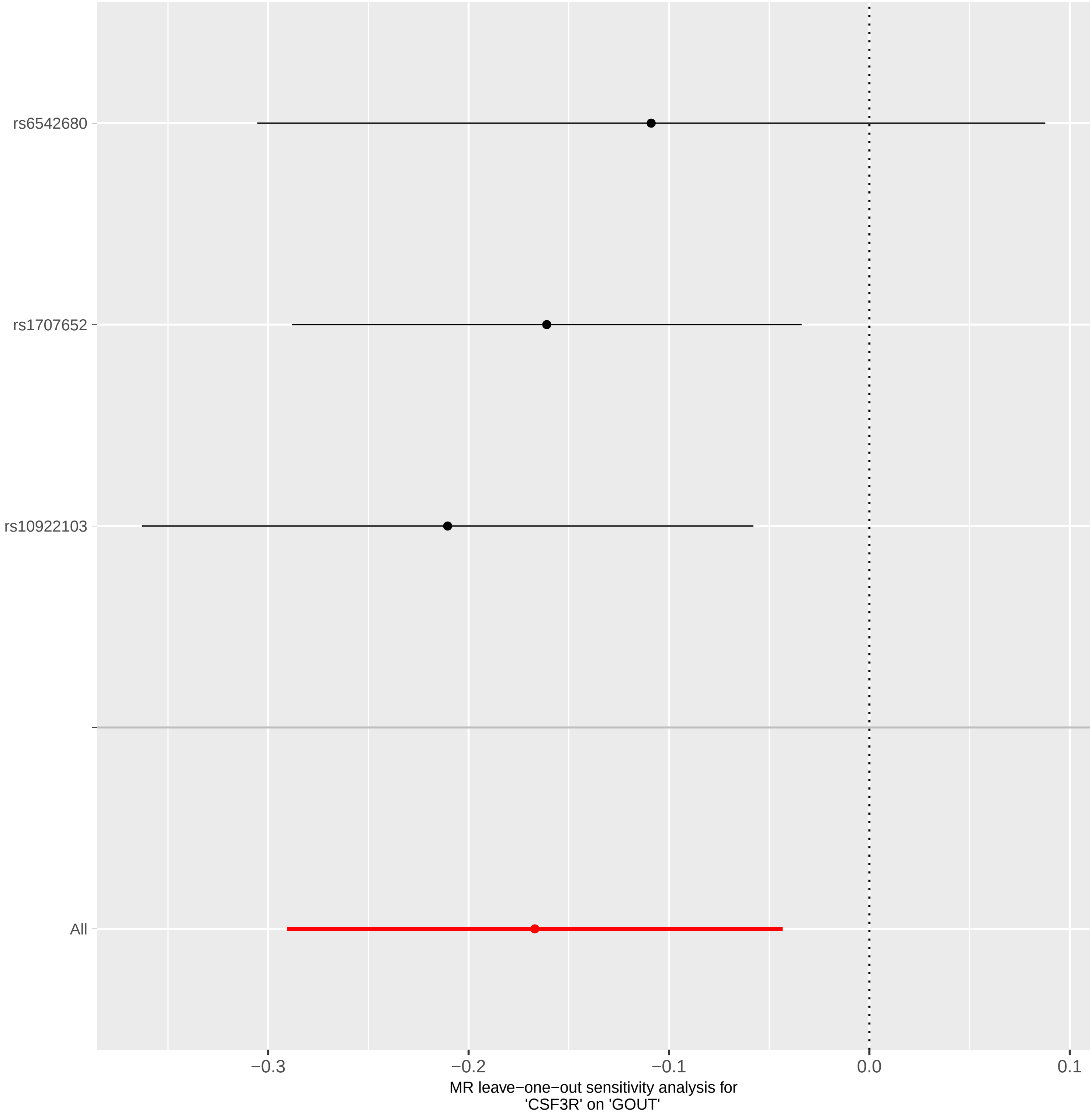

rs6542680
rs1707652
rs10922103
All
−0.3
−0.2
−0.1
0.0
0.1
MR leave−one−out sensitivity analysis for
'CSF3R' on 'GOUT'

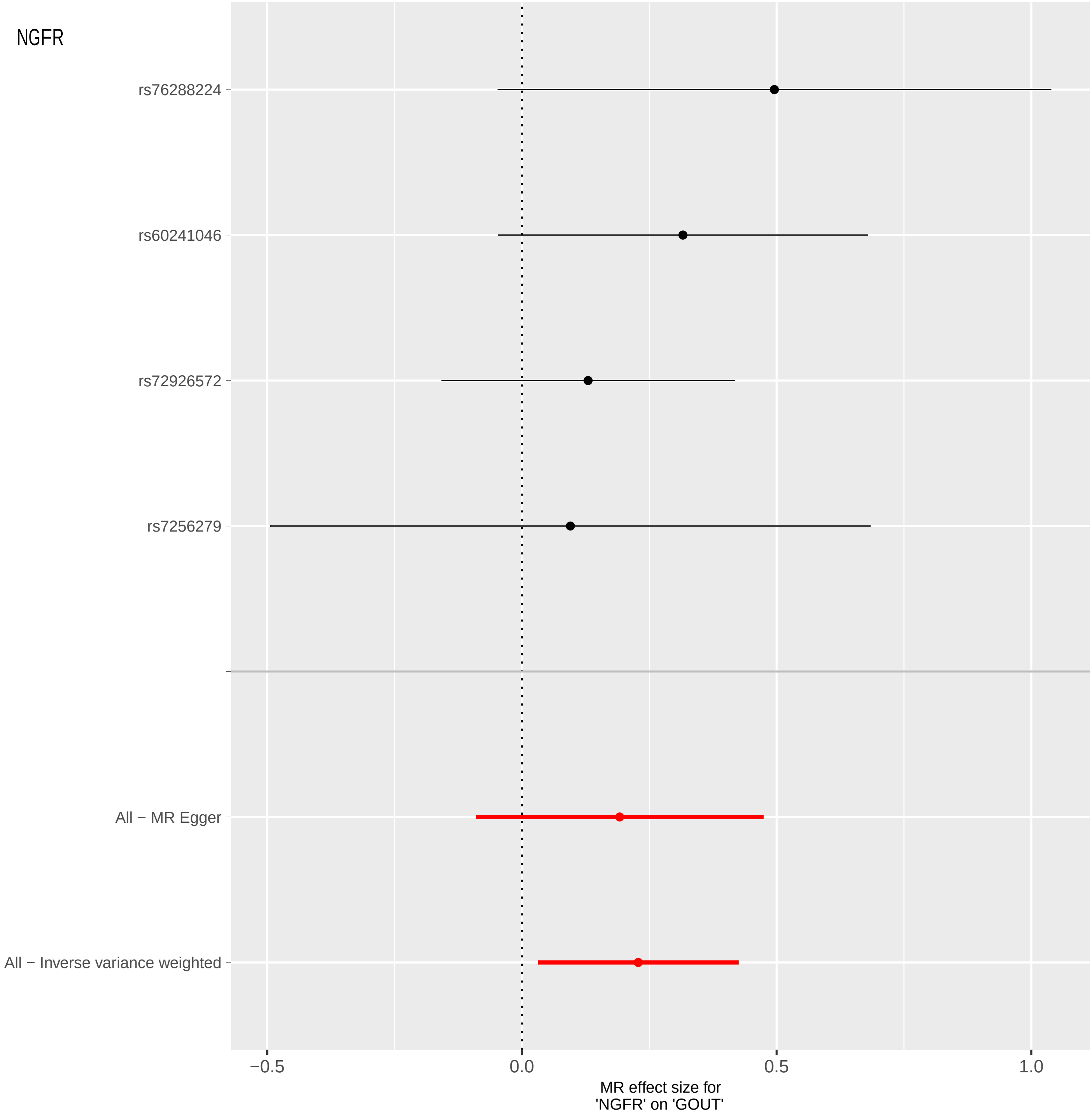
NGFR
rs76288224
rs60241046
rs72926572
rs7256279
All − MR Egger
All − Inverse variance weighted
−0.5
0.0
0.5
1.0
MR effect size for
'NGFR' on 'GOUT'

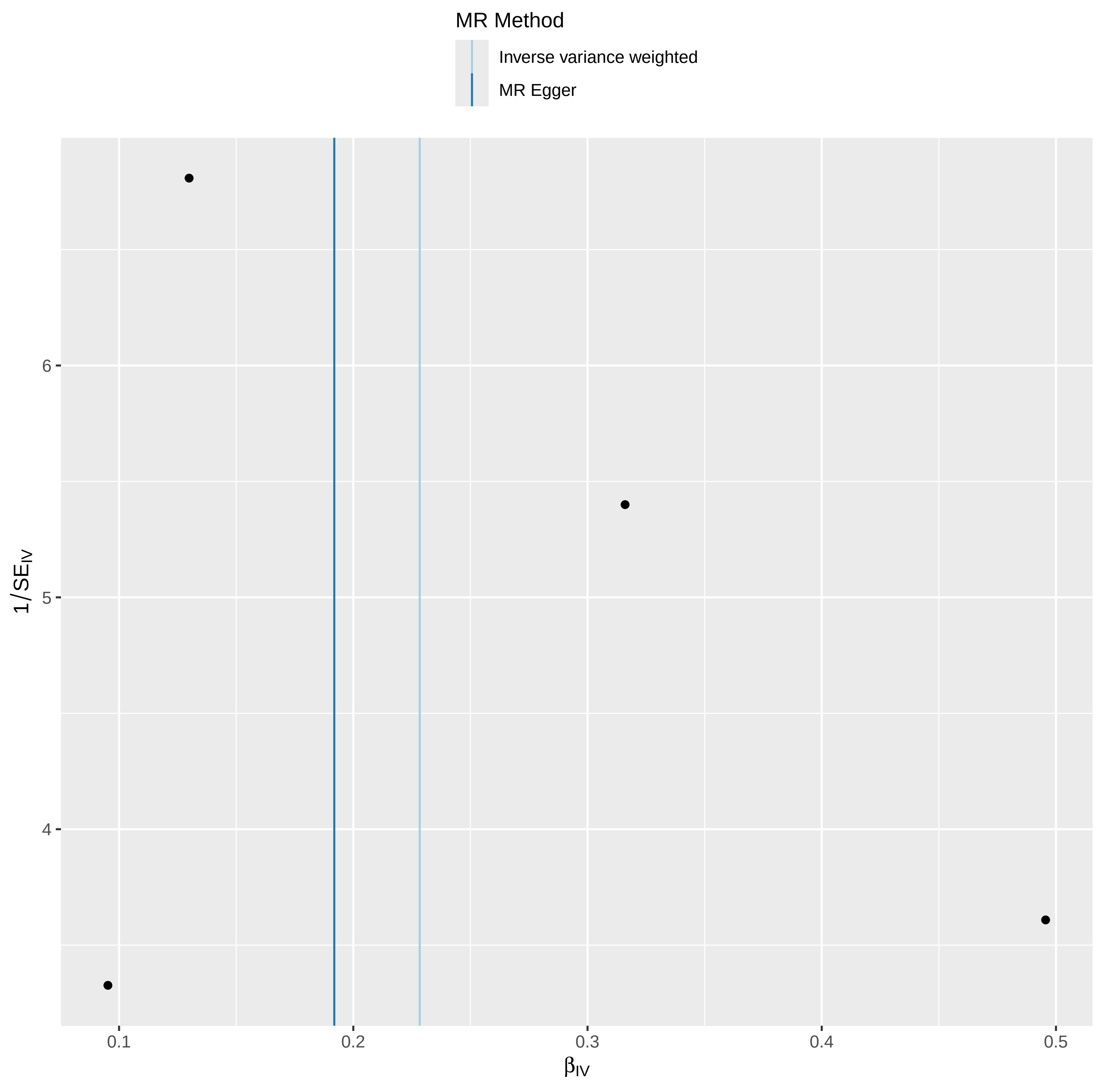
MR Method
Inverse variance weighted
MR Egger
6
5
4
1/SE$_{IV}$
0.1
0.2
0.3
0.4
0.5
β_{IV}

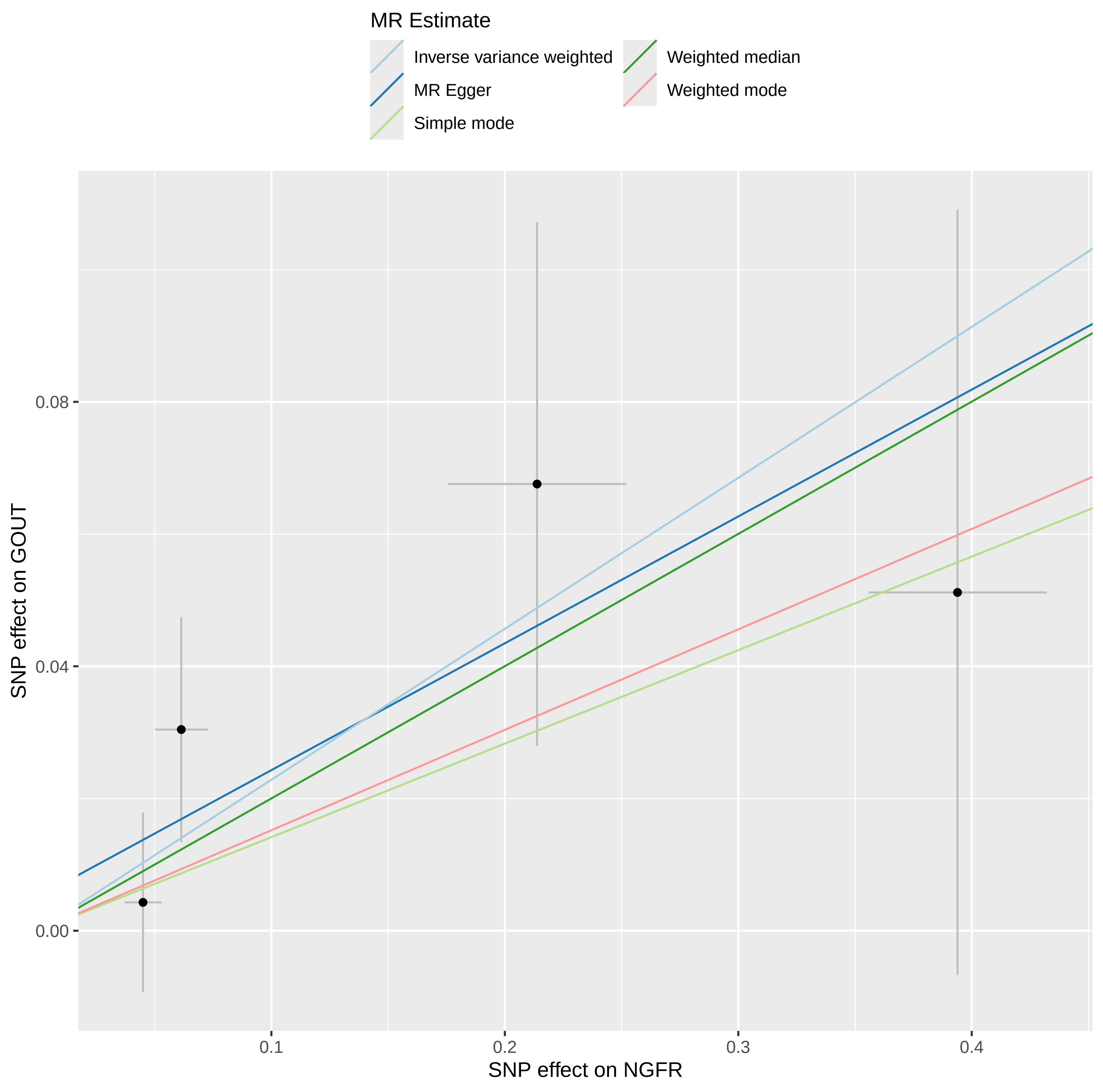
MR Estimate
Inverse variance weighted
MR Egger
Simple mode
Weighted median
Weighted mode
0.08
0.04
0.00
0.1
0.2
0.3
0.4
SNP effect on GOUT
SNP effect on NGFR

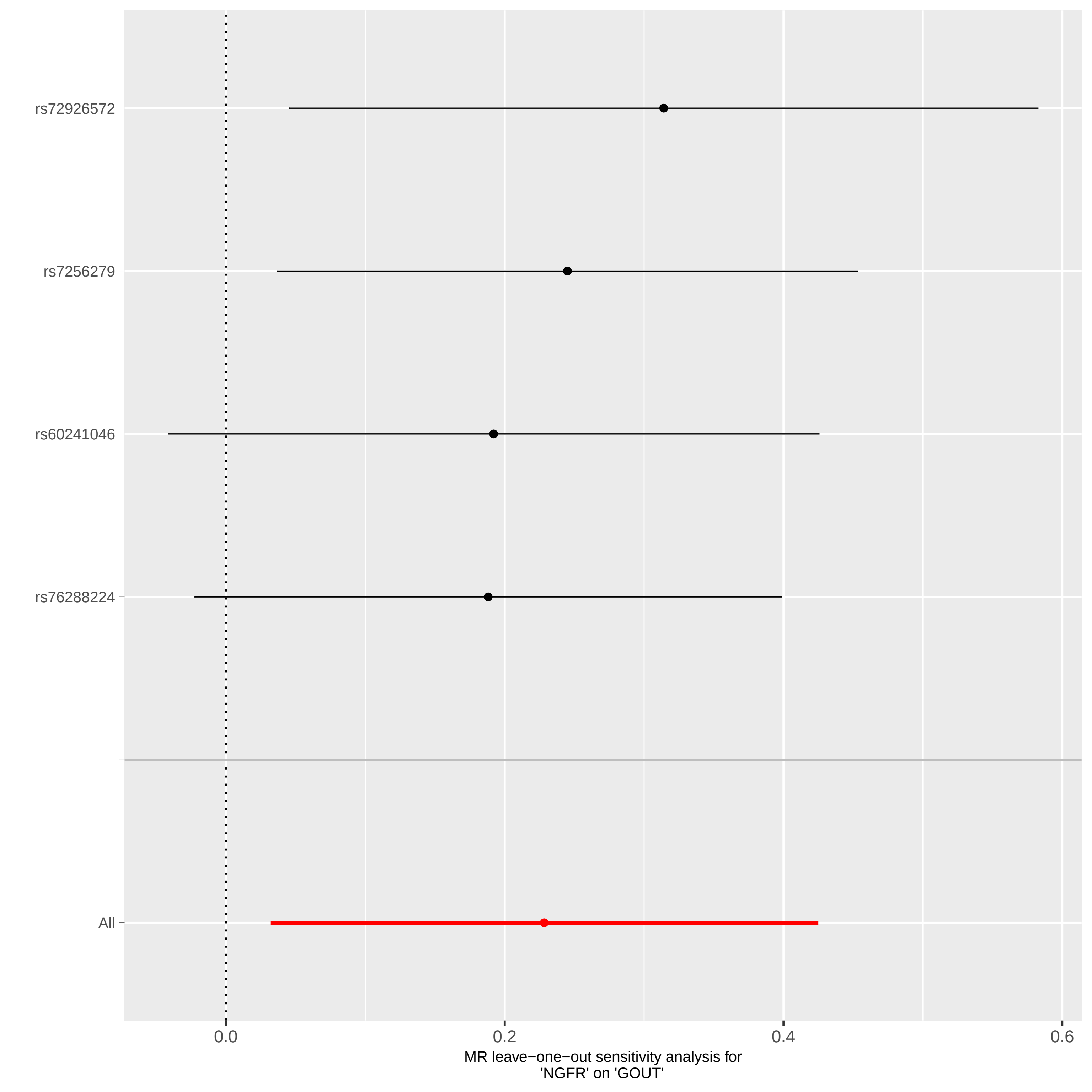

rs72926572
rs7256279
rs60241046
rs76288224
All
0.0
0.2
0.4
0.6
MR leave−one−out sensitivity analysis for
'NGFR' on 'GOUT'

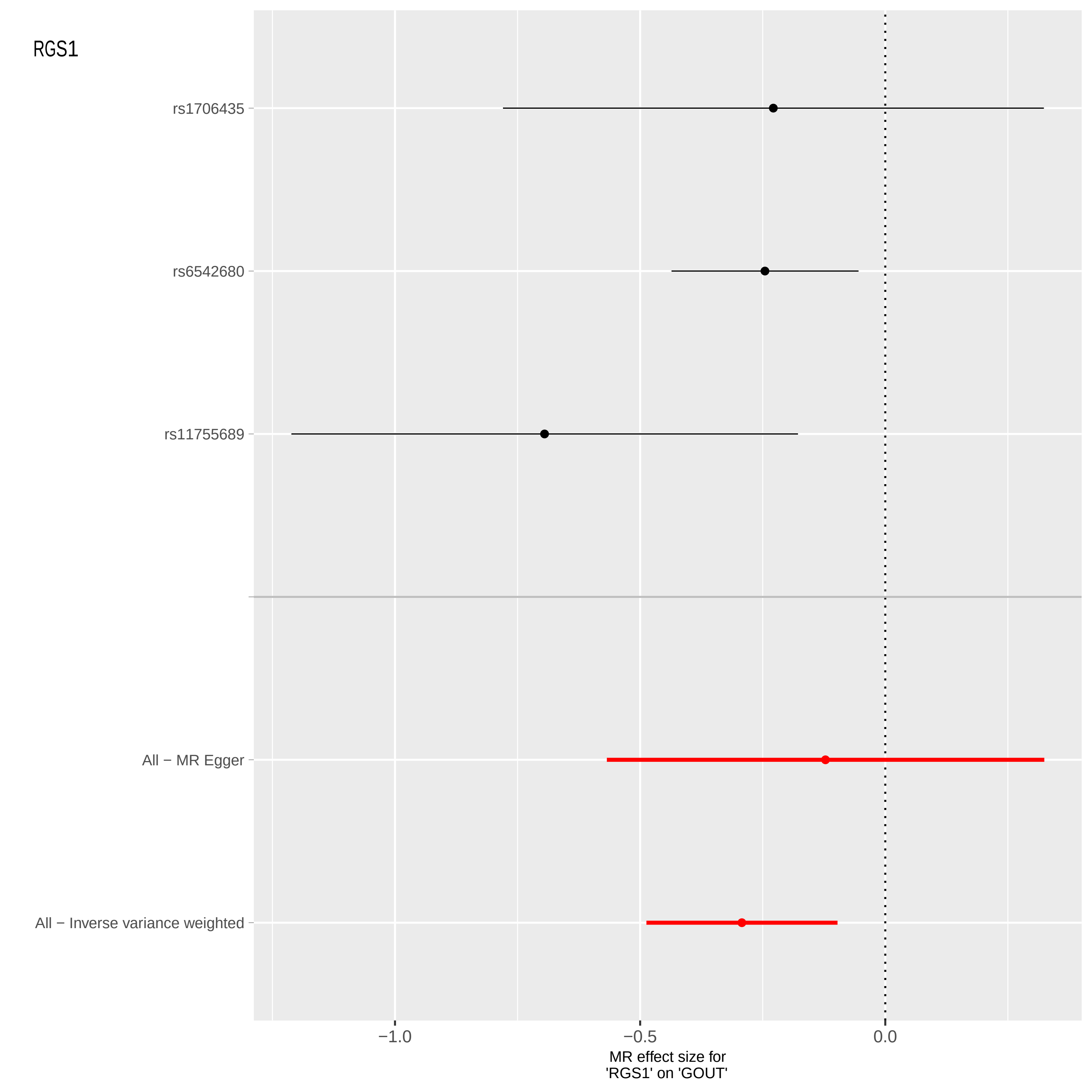
RGS1
rs1706435
rs6542680
rs11755689
All − MR Egger
All − Inverse variance weighted
−1.0
−0.5
0.0
MR effect size for
'RGS1' on 'GOUT'

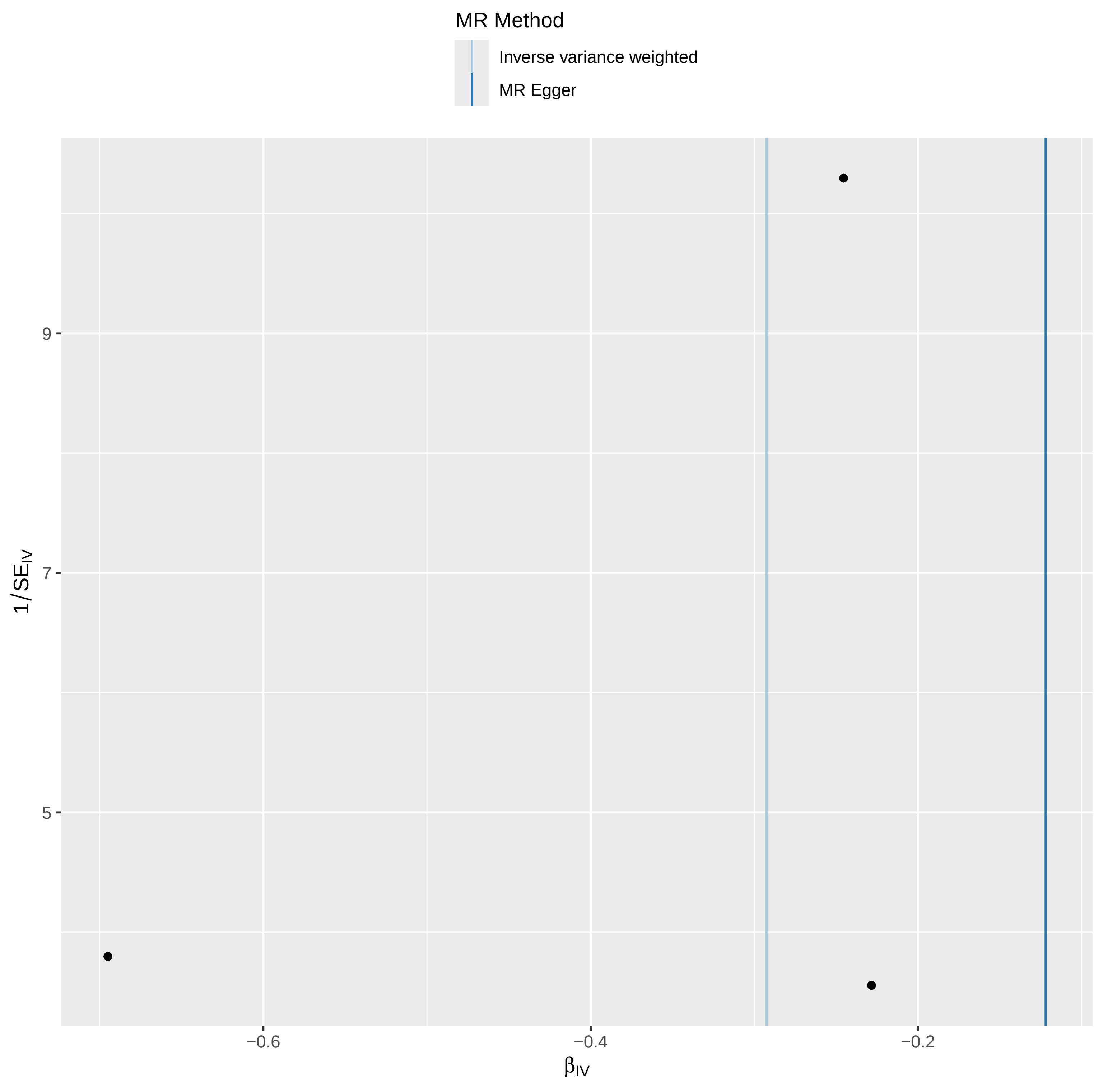

MR Method
Inverse variance weighted
MR Egger
9
7
5
1/SE_IV
−0.6
−0.4
−0.2
β_IV

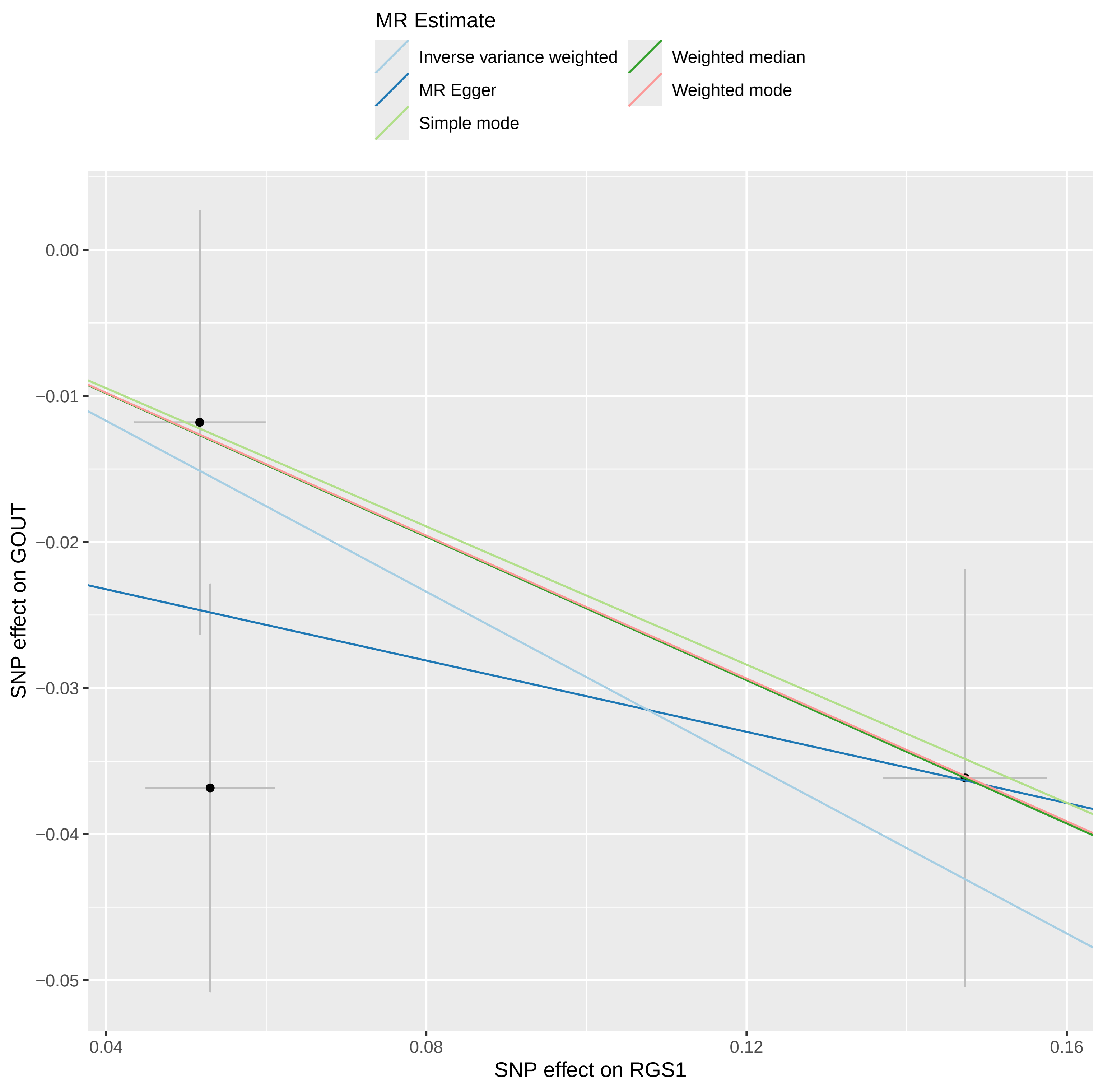

MR Estimate
Inverse variance weighted
MR Egger
Simple mode
Weighted median
Weighted mode
SNP effect on GOUT
0.00
−0.01
−0.02
−0.03
−0.04
−0.05
0.04
0.08
0.12
0.16
SNP effect on RGS1

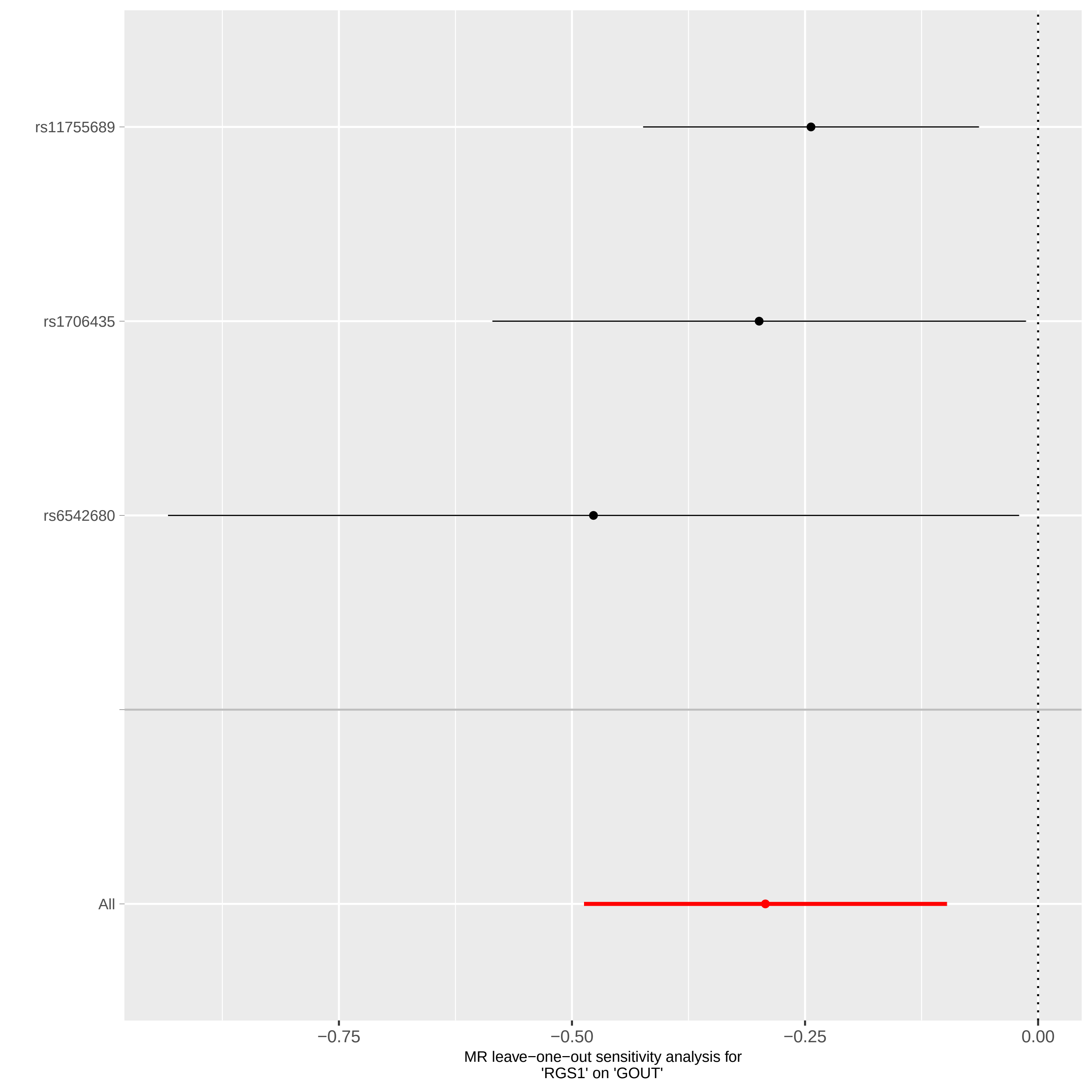
rs11755689
rs1706435
rs6542680
All
−0.75
−0.50
−0.25
0.00
MR leave−one−out sensitivity analysis for
'RGS1' on 'GOUT'

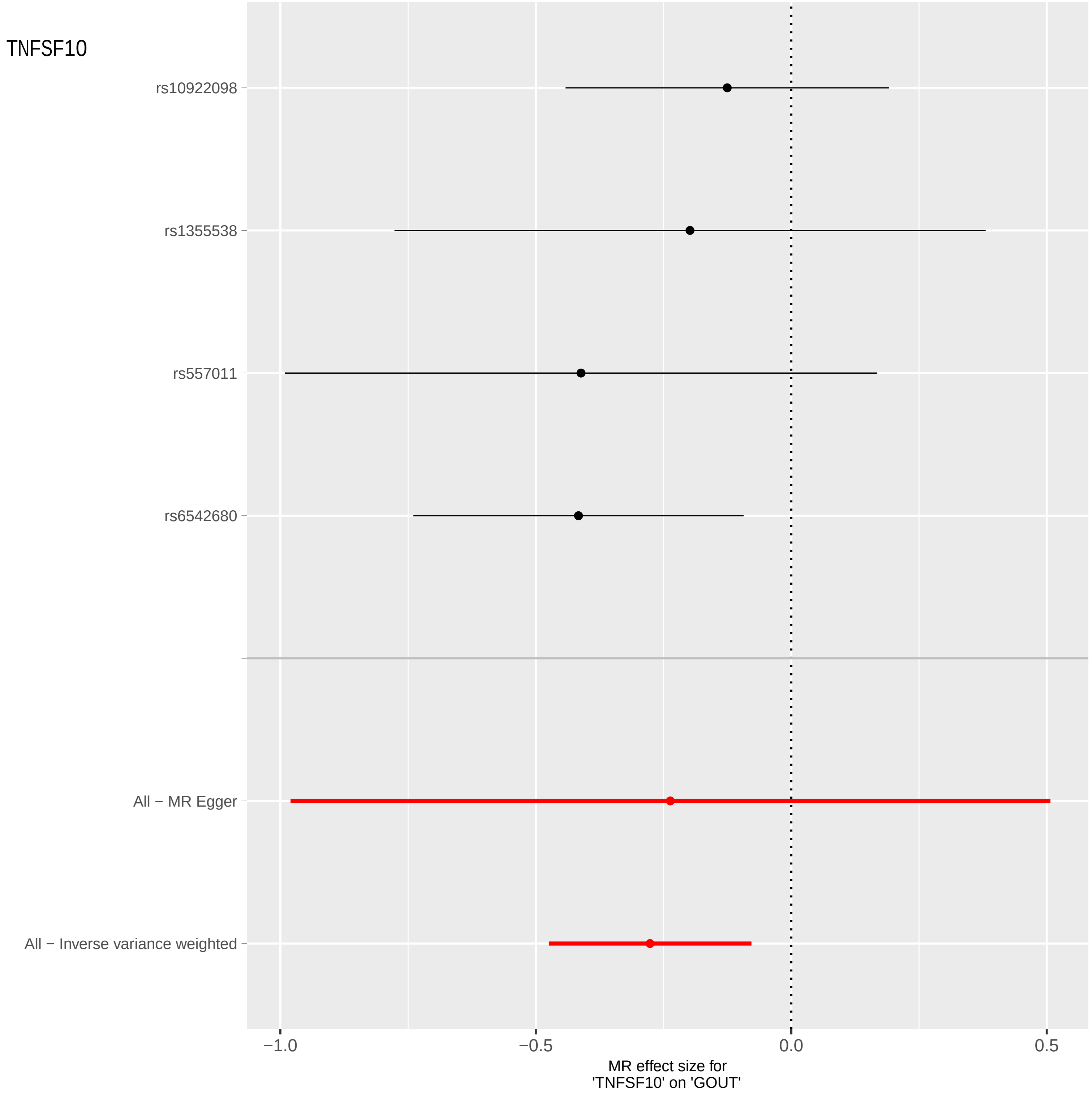
TNFSF10
rs10922098
rs1355538
rs557011
rs6542680
All − MR Egger
All − Inverse variance weighted
−1.0
−0.5
0.0
0.5
MR effect size for
'TNFSF10' on 'GOUT'

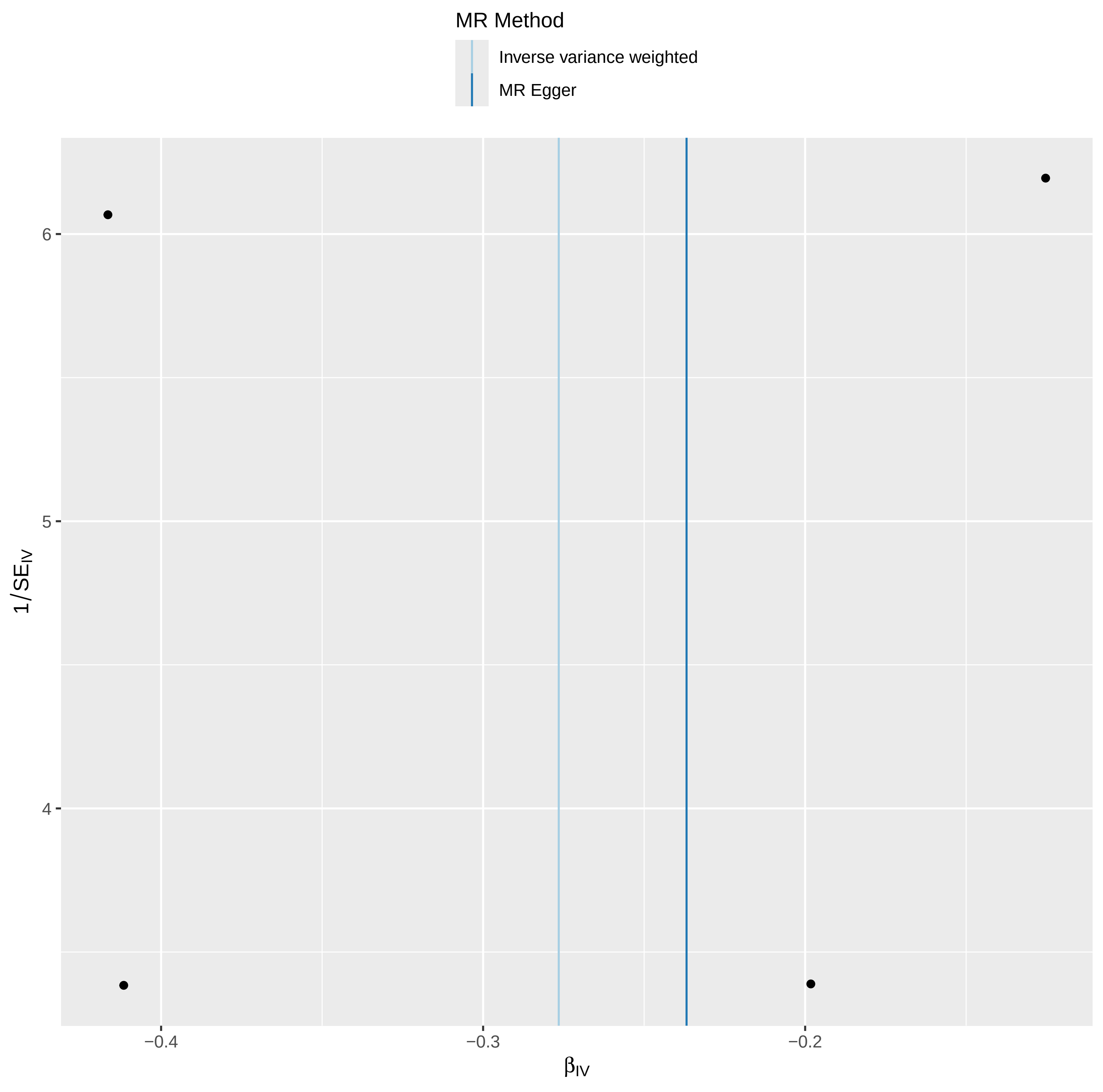
MR Method
Inverse variance weighted
MR Egger
6
5
4
1/SE_IV
−0.4
−0.3
−0.2
β_IV

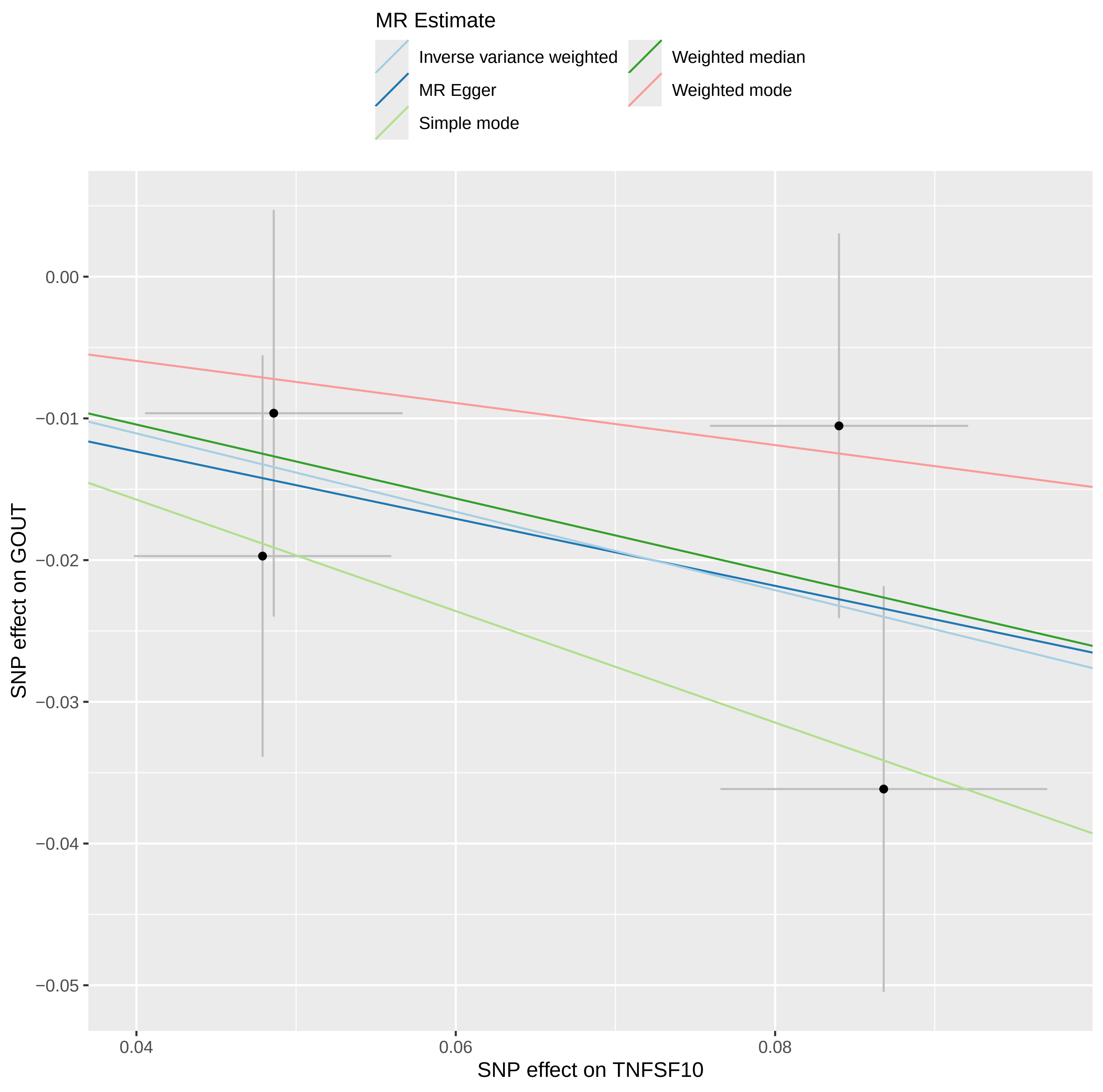

MR Estimate
Inverse variance weighted
MR Egger
Simple mode
Weighted median
Weighted mode
0.00
−0.01
−0.02
−0.03
−0.04
−0.05
SNP effect on GOUT
0.04
0.06
0.08
SNP effect on TNFSF10

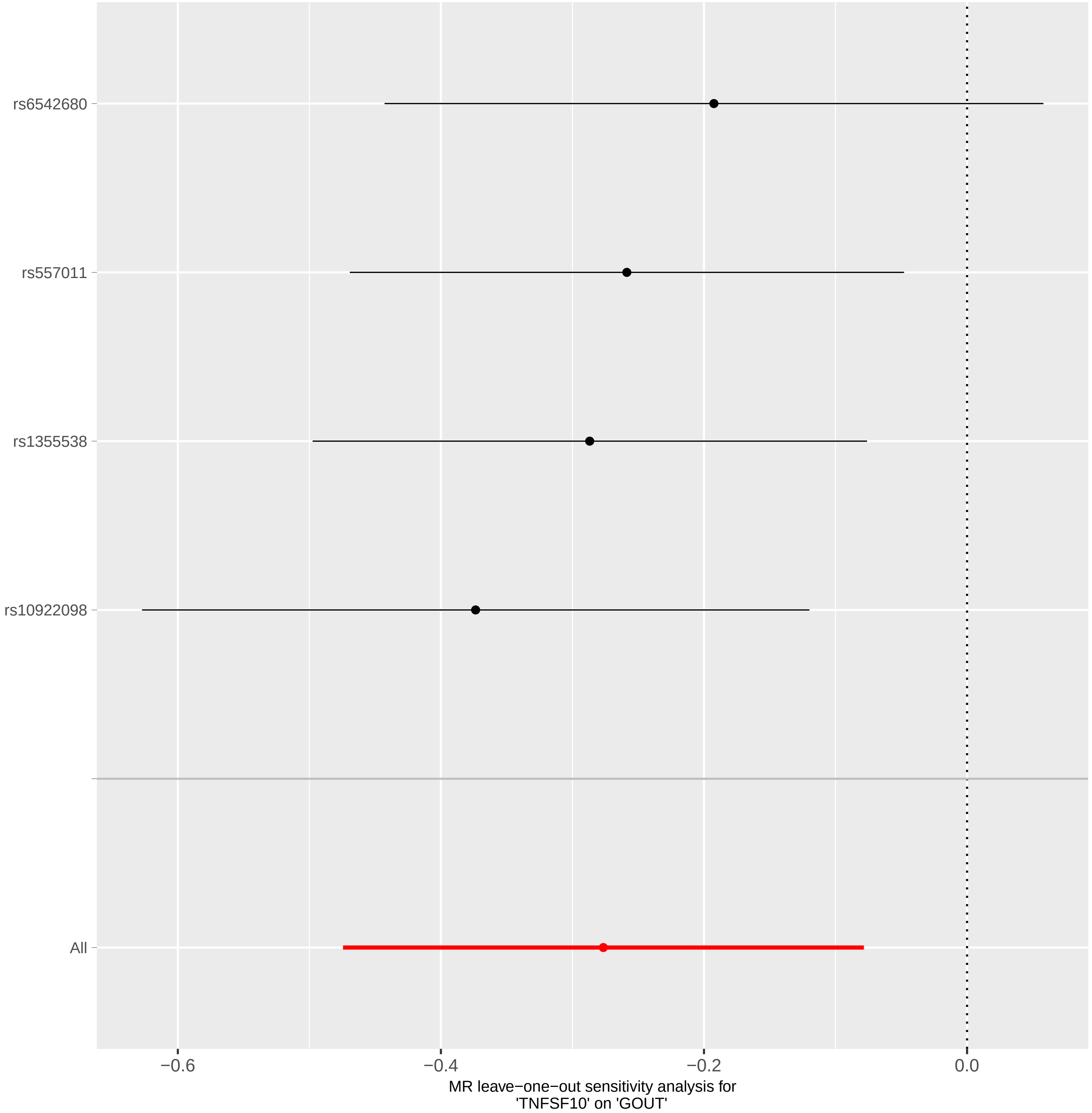

rs6542680
rs557011
rs1355538
rs10922098
All
−0.6
−0.4
−0.2
0.0
MR leave−one−out sensitivity analysis for
'TNFSF10' on 'GOUT'

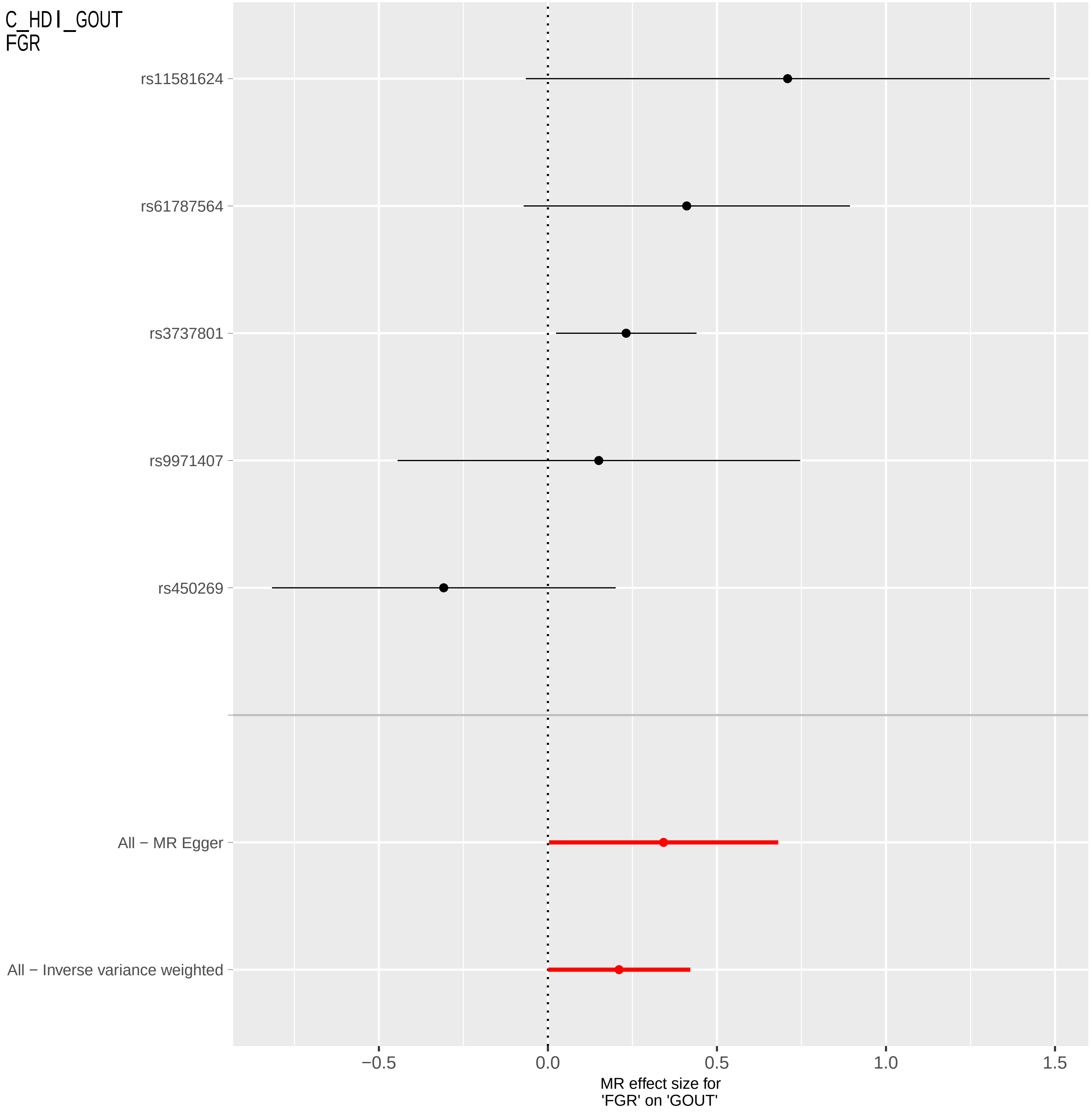
C_HDI_GOUT
FGR
rs11581624
rs61787564
rs3737801
rs9971407
rs450269
All − MR Egger
All − Inverse variance weighted
−0.5
0.0
0.5
1.0
1.5
MR effect size for
'FGR' on 'GOUT'

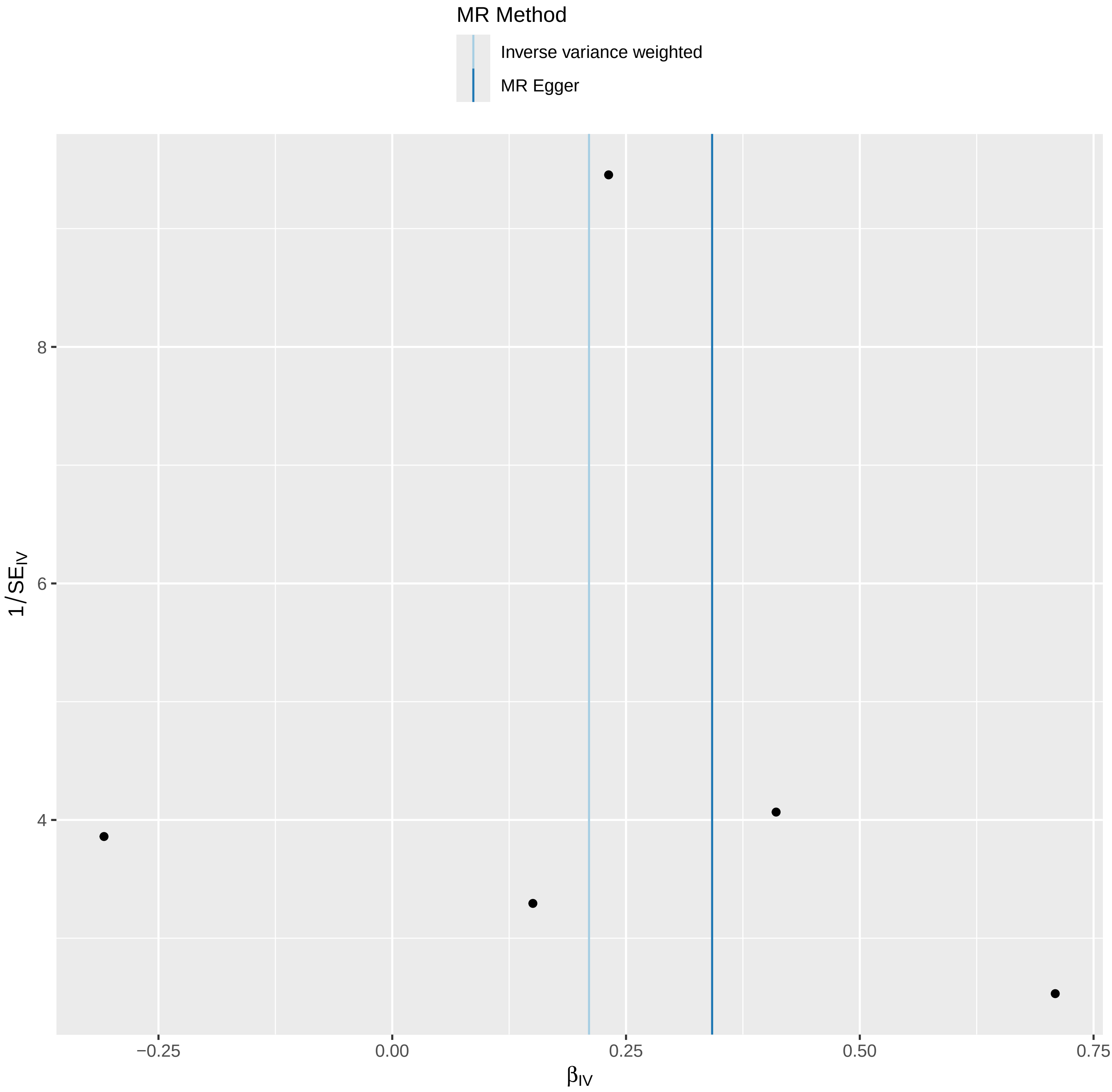
MR Method
Inverse variance weighted
MR Egger
8
6
4
1/SE_IV
−0.25
0.00
0.25
0.50
0.75
β_IV

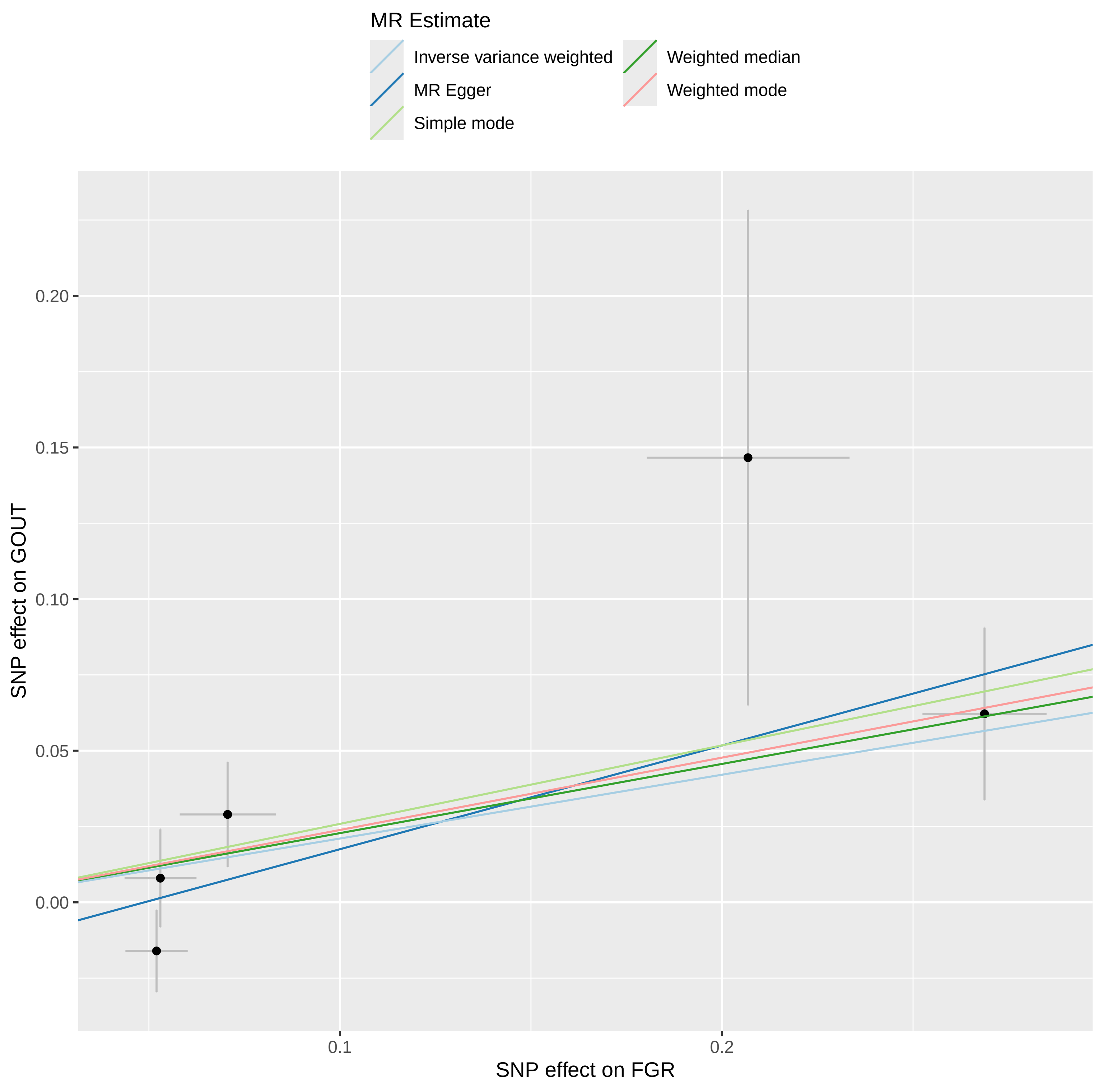
MR Estimate
Inverse variance weighted
MR Egger
Simple mode
Weighted median
Weighted mode
SNP effect on GOUT
0.20
0.15
0.10
0.05
0.00
0.1
0.2
SNP effect on FGR

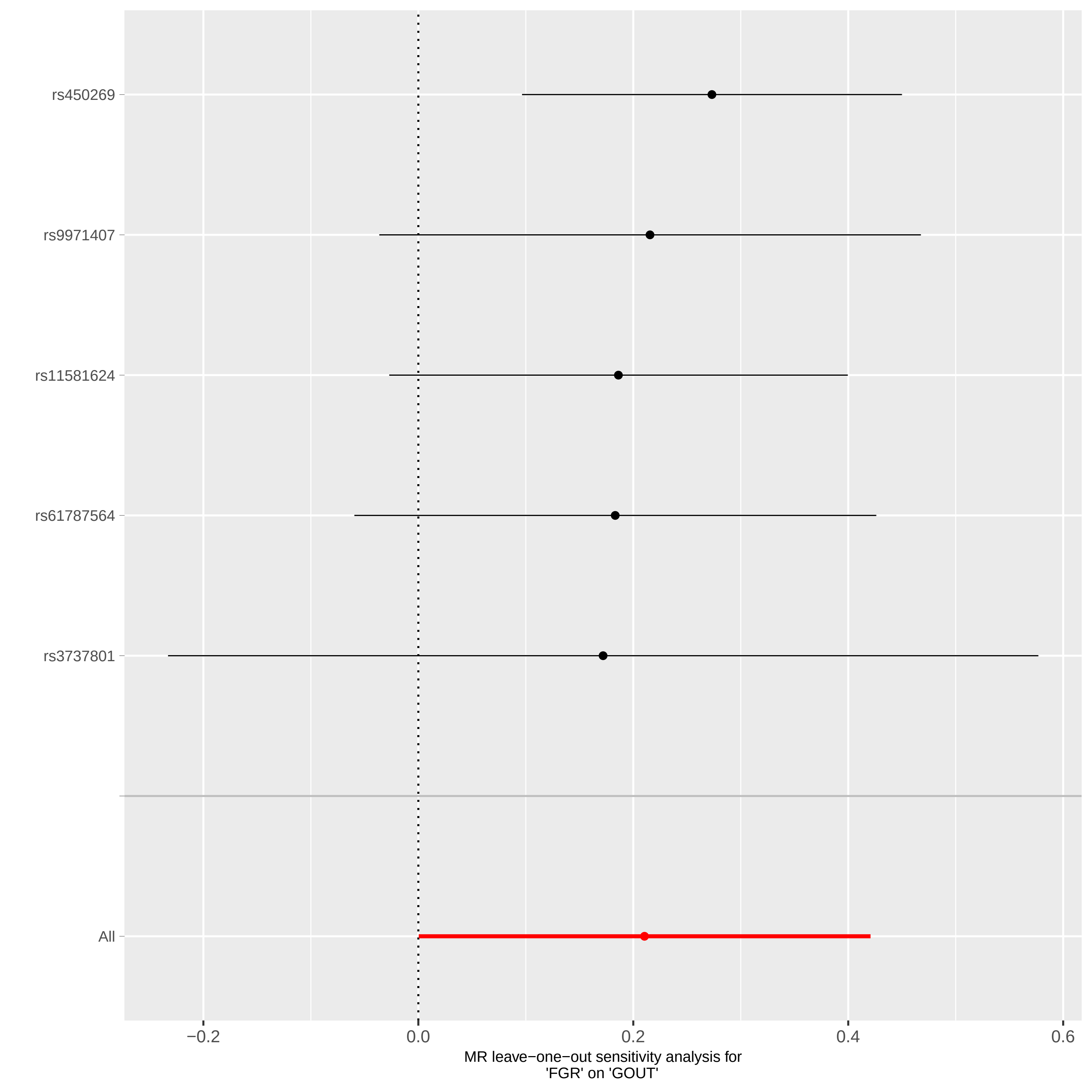
rs450269
rs9971407
rs11581624
rs61787564
rs3737801
All
−0.2
0.0
0.2
0.4
0.6
MR leave−one−out sensitivity analysis for
'FGR' on 'GOUT'

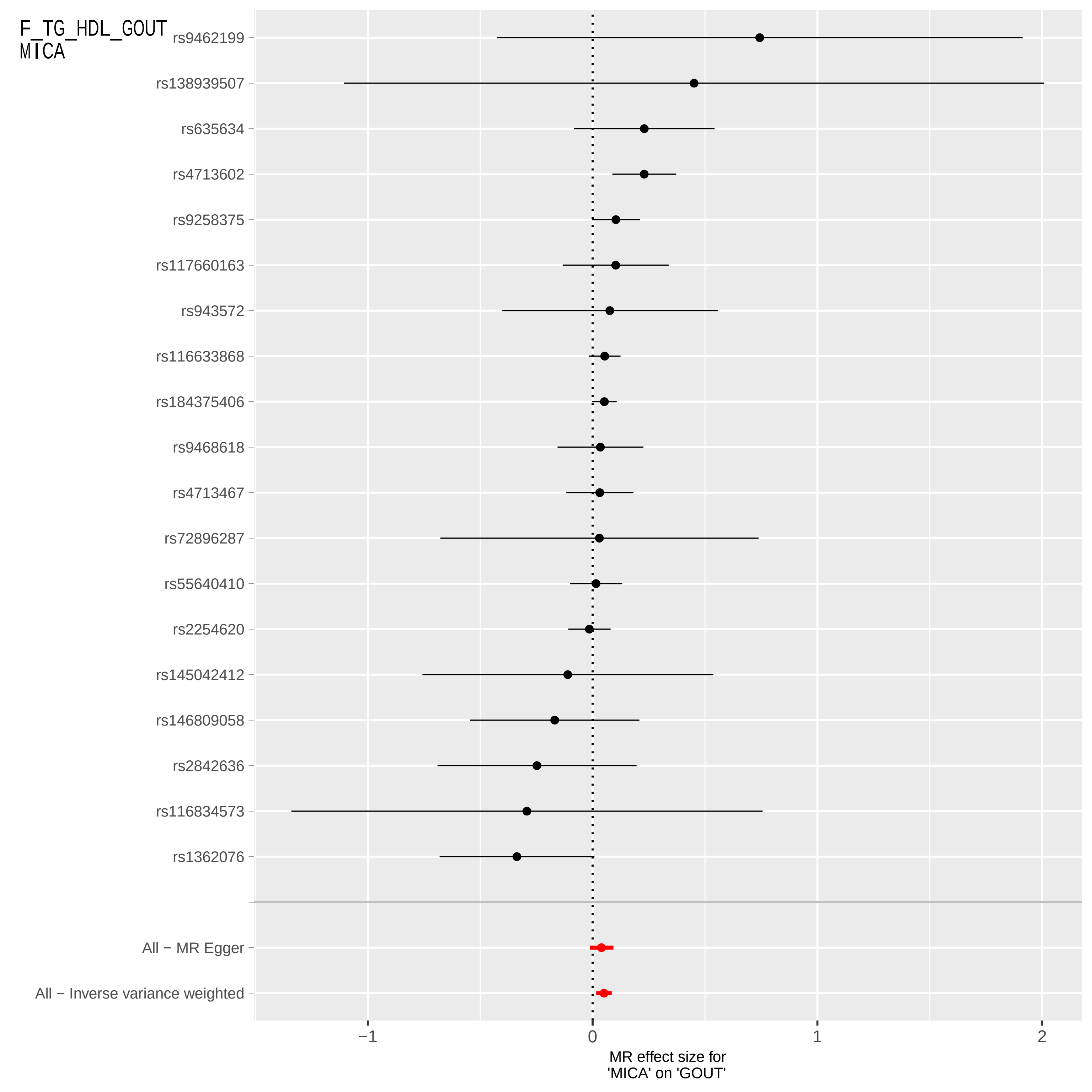
F_TG_HDL_GOUT
MICA
rs9462199
rs138939507
rs635634
rs4713602
rs9258375
rs117660163
rs943572
rs116633868
rs184375406
rs9468618
rs4713467
rs72896287
rs55640410
rs2254620
rs145042412
rs146809058
rs2842636
rs116834573
rs1362076
All − MR Egger
All − Inverse variance weighted
−1
0
1
2
MR effect size for
'MICA' on 'GOUT'

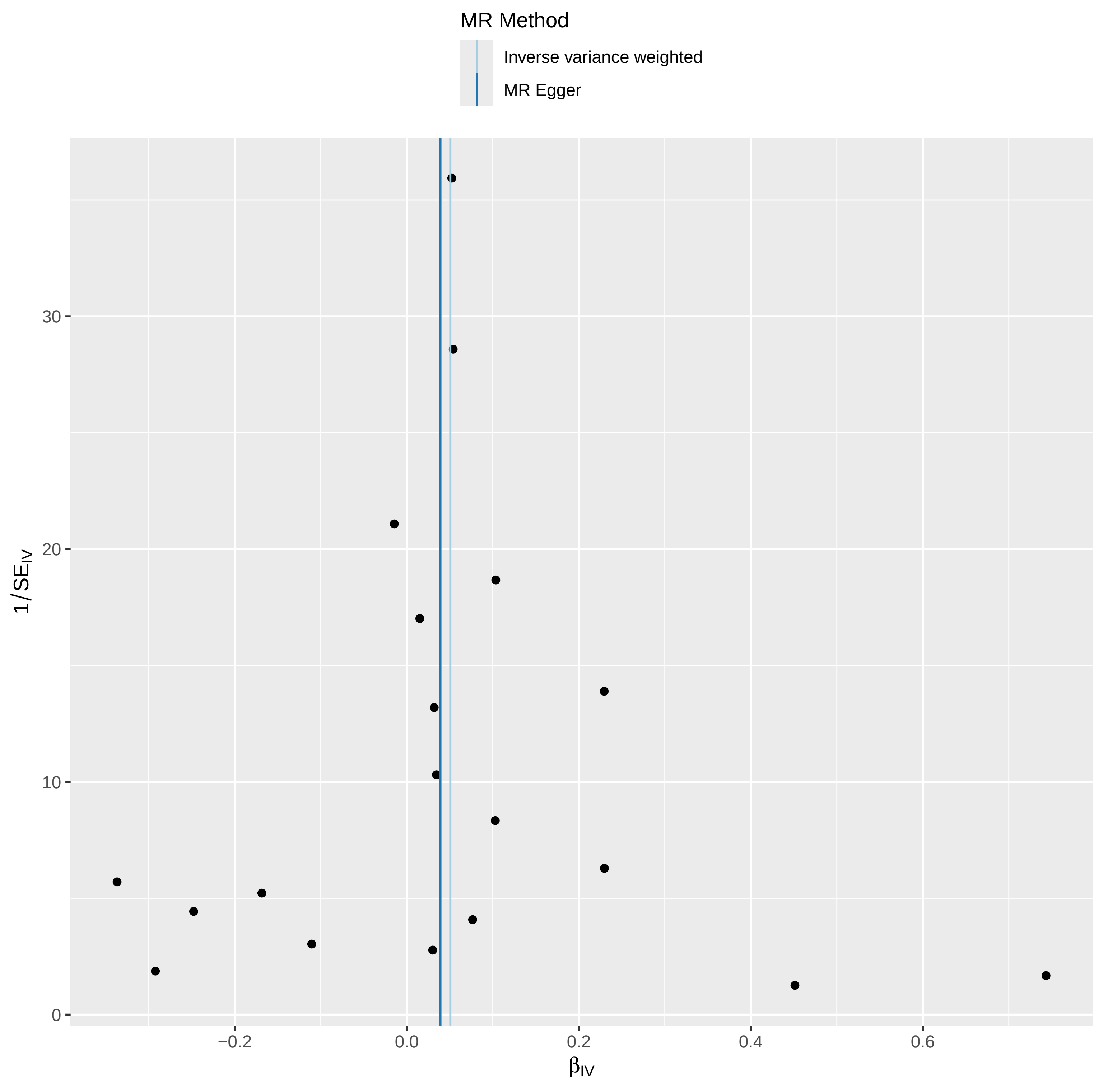

MR Method
Inverse variance weighted
MR Egger
30
20
10
0
1/SE$_{IV}$
−0.2
0.0
0.2
0.4
0.6
β$_{IV}$

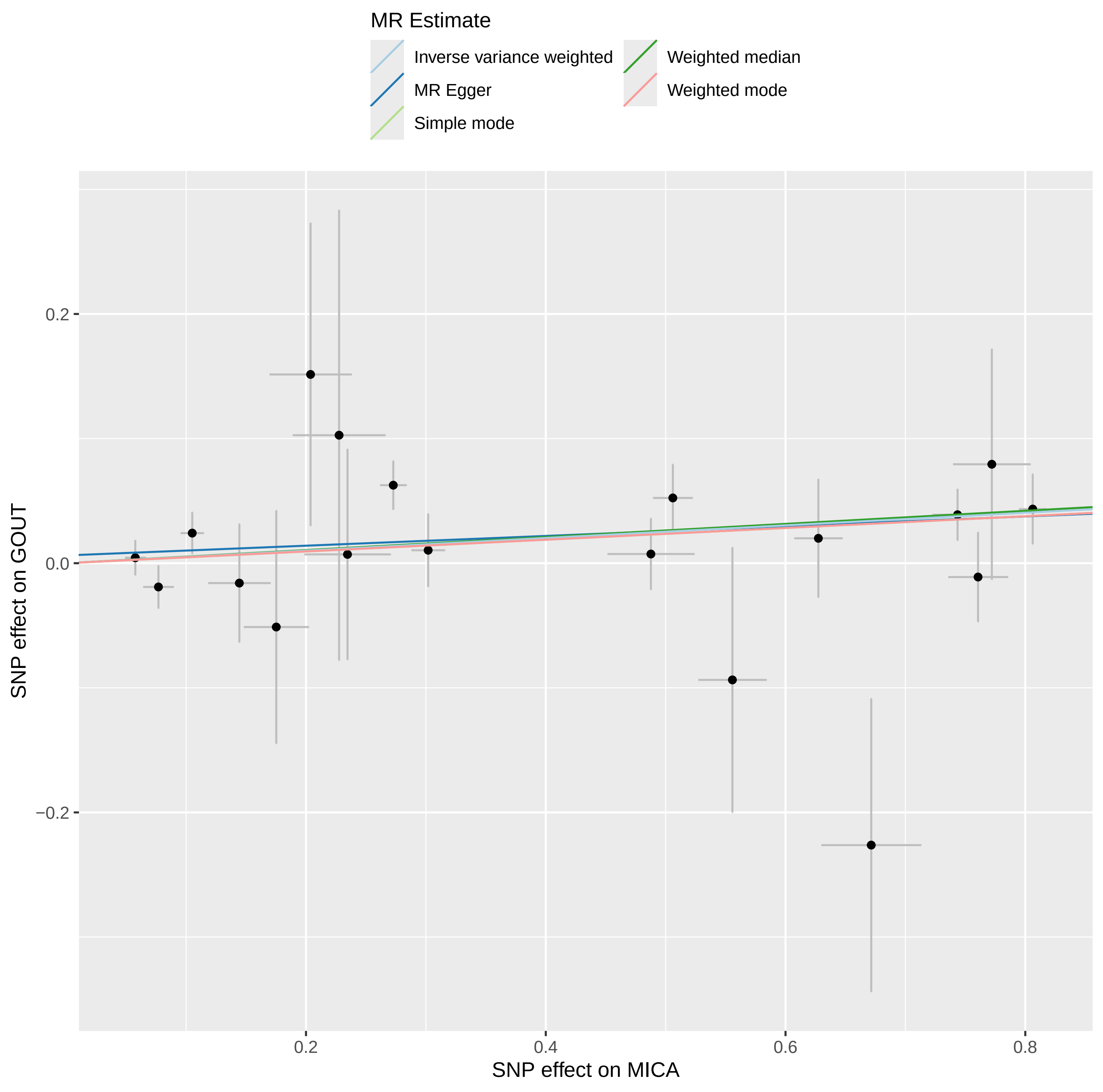
MR Estimate
Inverse variance weighted
MR Egger
Simple mode
Weighted median
Weighted mode
0.2
0.0
−0.2
SNP effect on GOUT
0.2
0.4
0.6
0.8
SNP effect on MICA

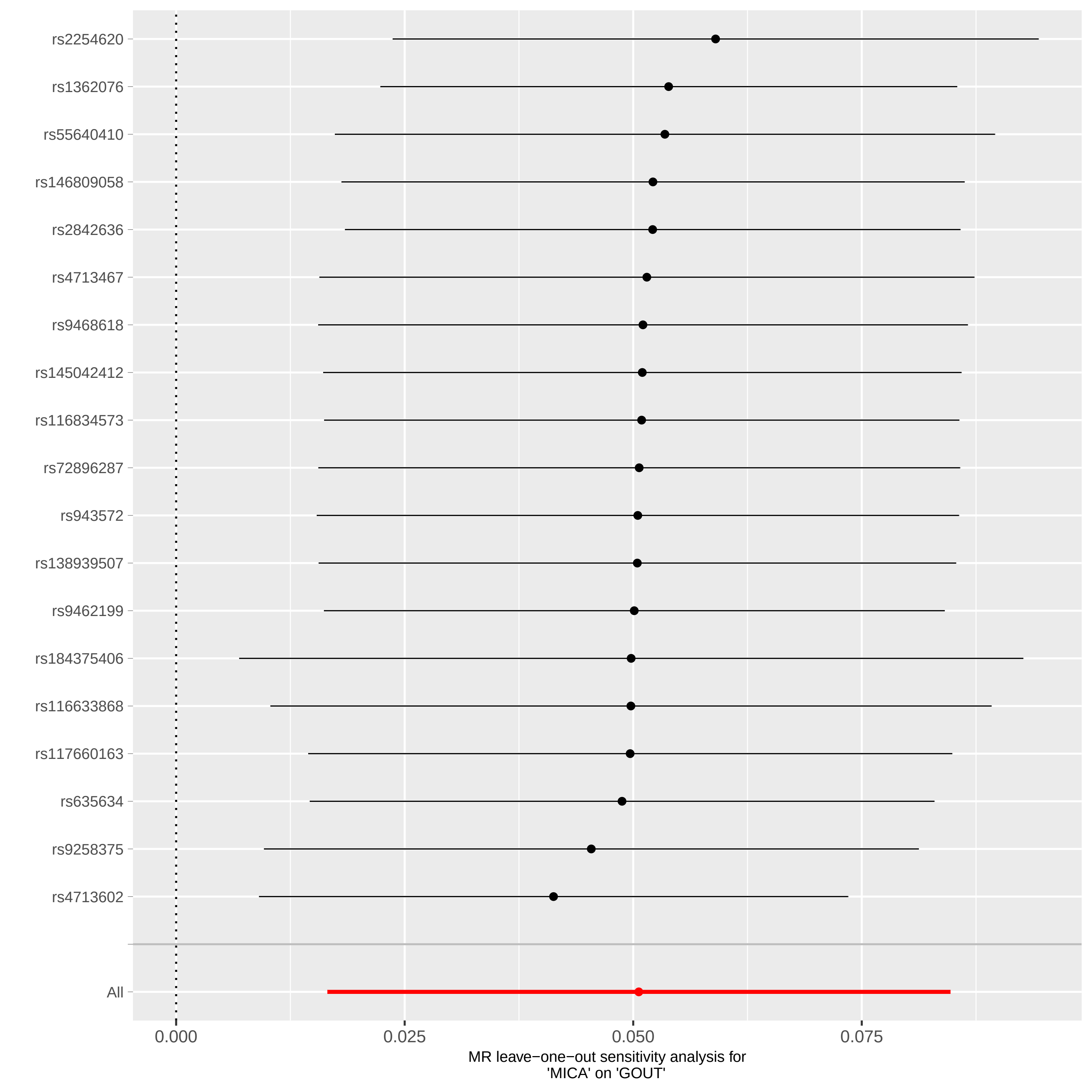
rs2254620
rs1362076
rs55640410
rs146809058
rs2842636
rs4713467
rs9468618
rs145042412
rs116834573
rs72896287
rs943572
rs138939507
rs9462199
rs184375406
rs116633868
rs117660163
rs635634
rs9258375
rs4713602
All
0.000
0.025
0.050
0.075
MR leave−one−out sensitivity analysis for
'MICA' on 'GOUT'

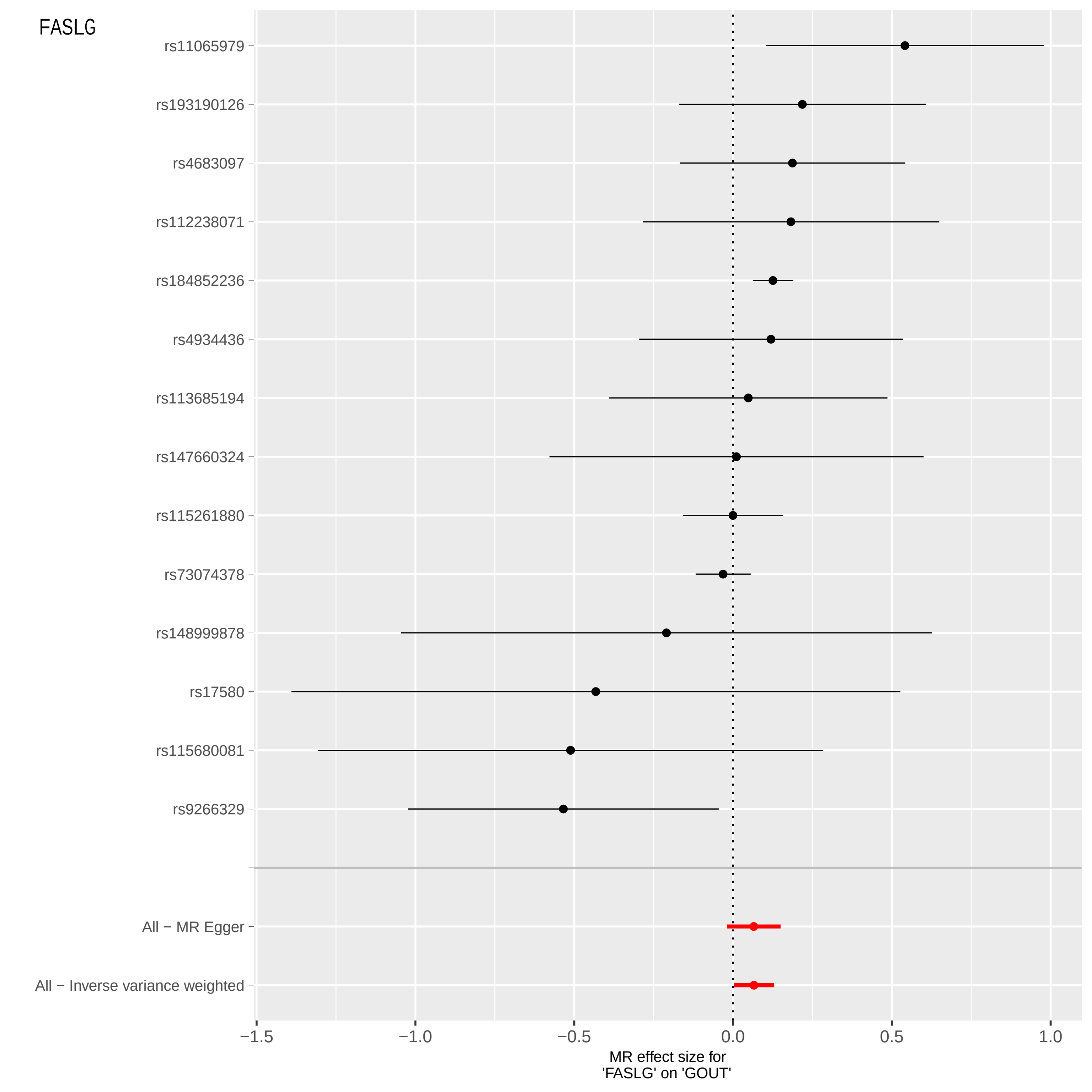
FASLG
rs11065979
rs193190126
rs4683097
rs112238071
rs184852236
rs4934436
rs113685194
rs147660324
rs115261880
rs73074378
rs148999878
rs17580
rs115680081
rs9266329
All − MR Egger
All − Inverse variance weighted
−1.5
−1.0
−0.5
0.0
0.5
1.0
MR effect size for
'FASLG' on 'GOUT'

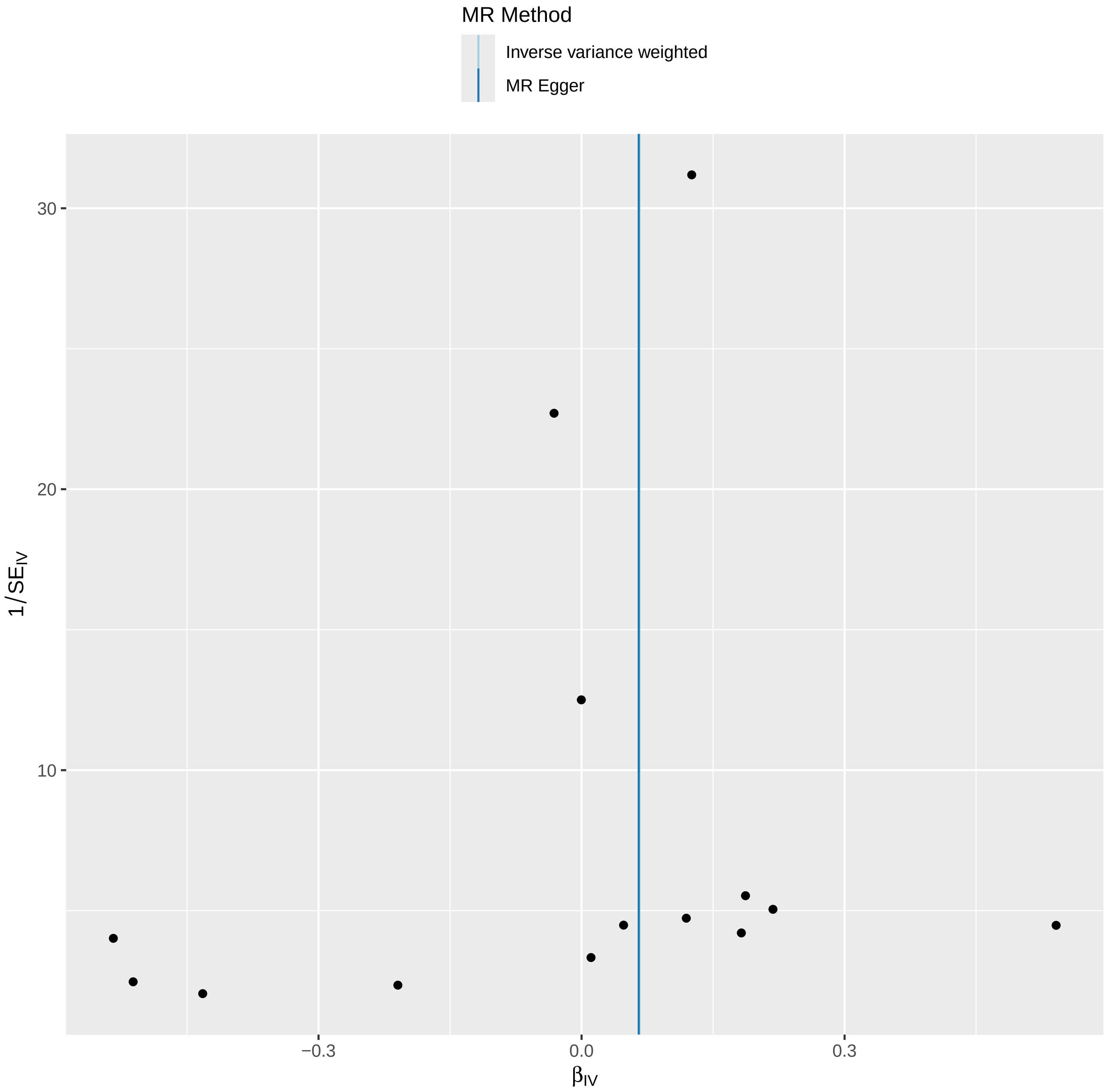

MR Method
Inverse variance weighted
MR Egger
30
20
10
1/SE$_{IV}$
−0.3
0.0
0.3
β$_{IV}$

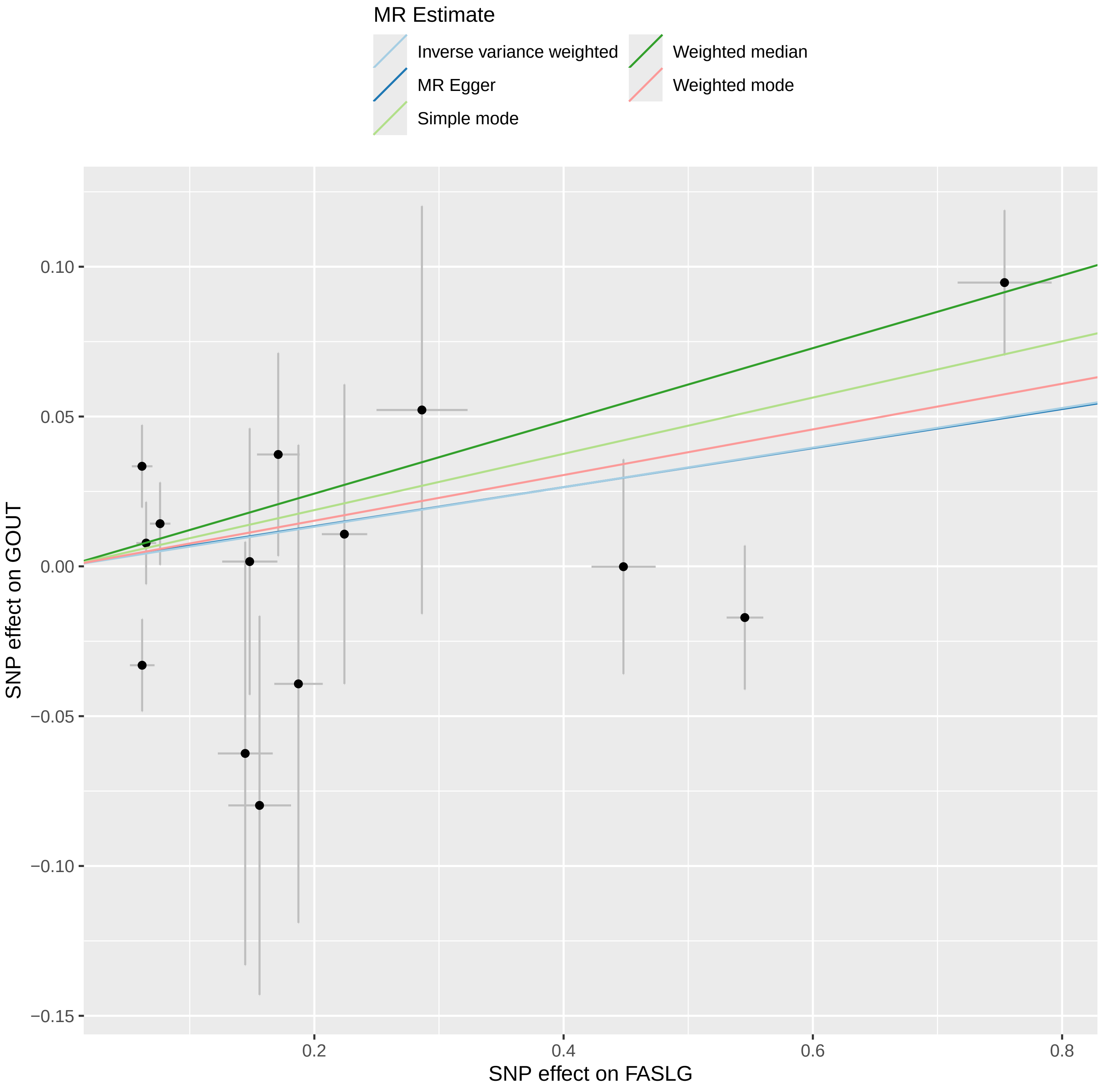
MR Estimate
Inverse variance weighted
MR Egger
Simple mode
Weighted median
Weighted mode
0.10
0.05
0.00
−0.05
−0.10
−0.15
SNP effect on GOUT
0.2
0.4
0.6
0.8
SNP effect on FASLG

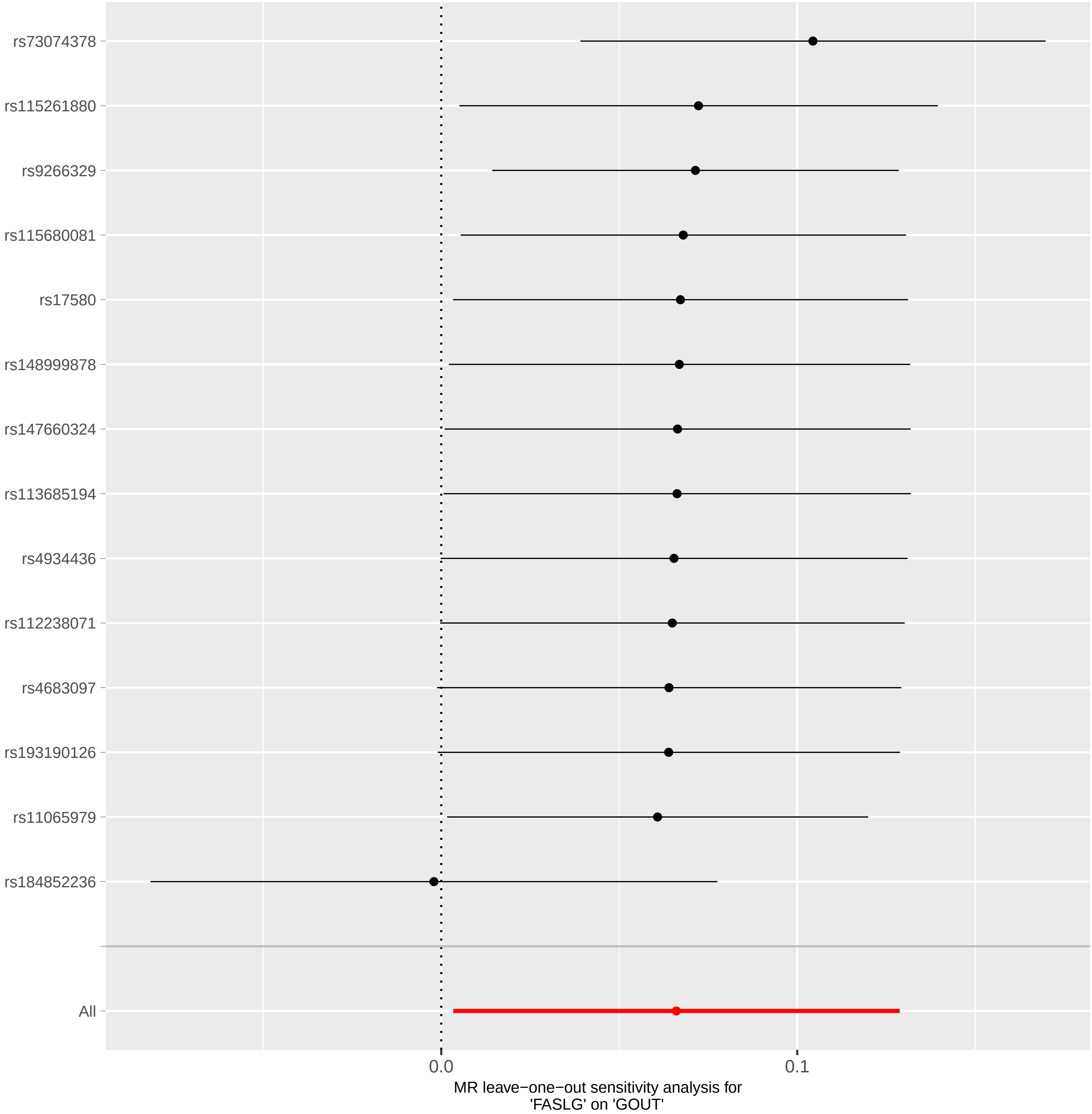
rs73074378
rs115261880
rs9266329
rs115680081
rs17580
rs148999878
rs147660324
rs113685194
rs4934436
rs112238071
rs4683097
rs193190126
rs11065979
rs184852236
All
0.0
0.1
MR leave−one−out sensitivity analysis for
'FASLG' on 'GOUT'

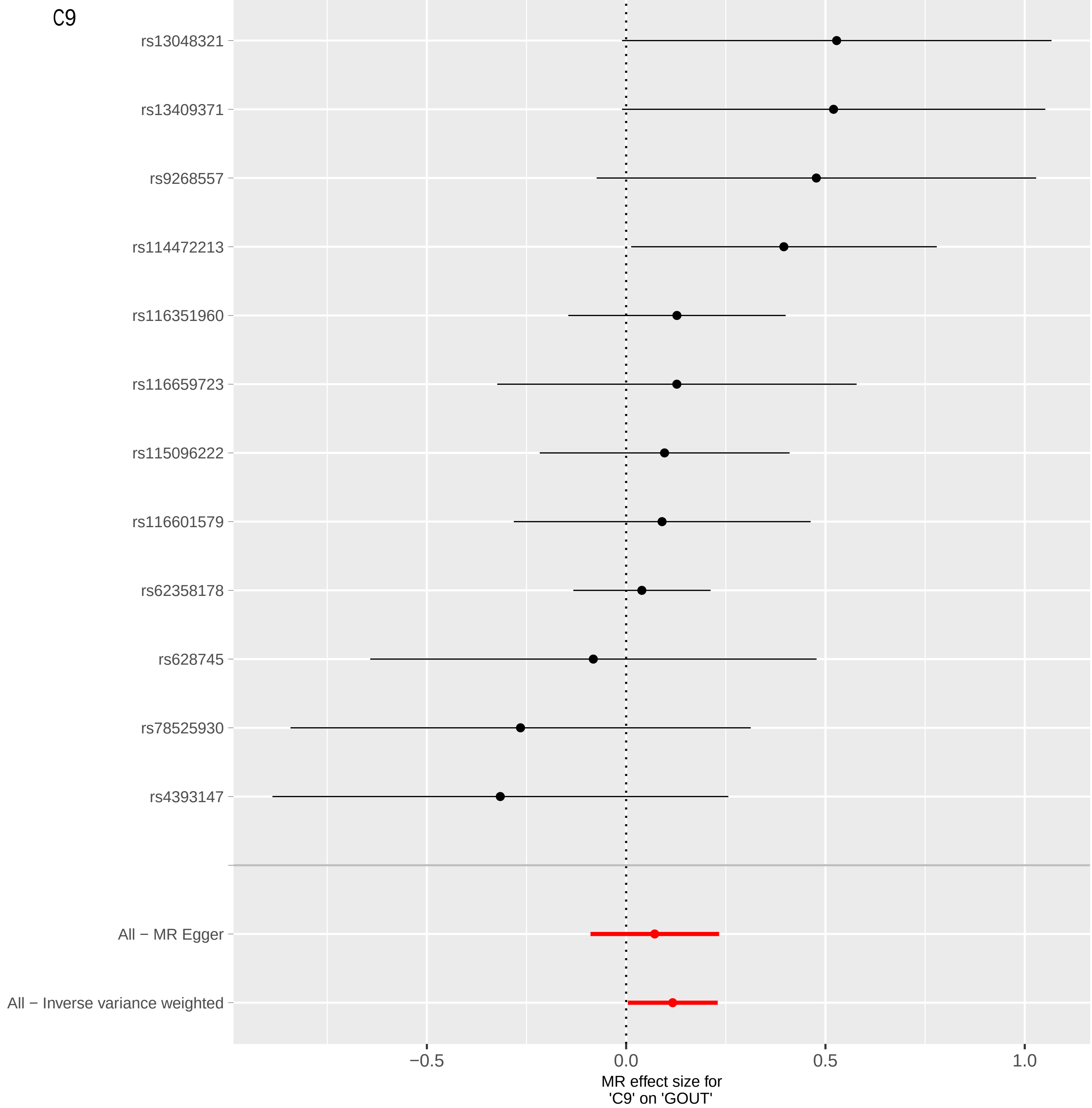
C9
rs13048321
rs13409371
rs9268557
rs114472213
rs116351960
rs116659723
rs115096222
rs116601579
rs62358178
rs628745
rs78525930
rs4393147
All − MR Egger
All − Inverse variance weighted
−0.5
0.0
0.5
1.0
MR effect size for
'C9' on 'GOUT'

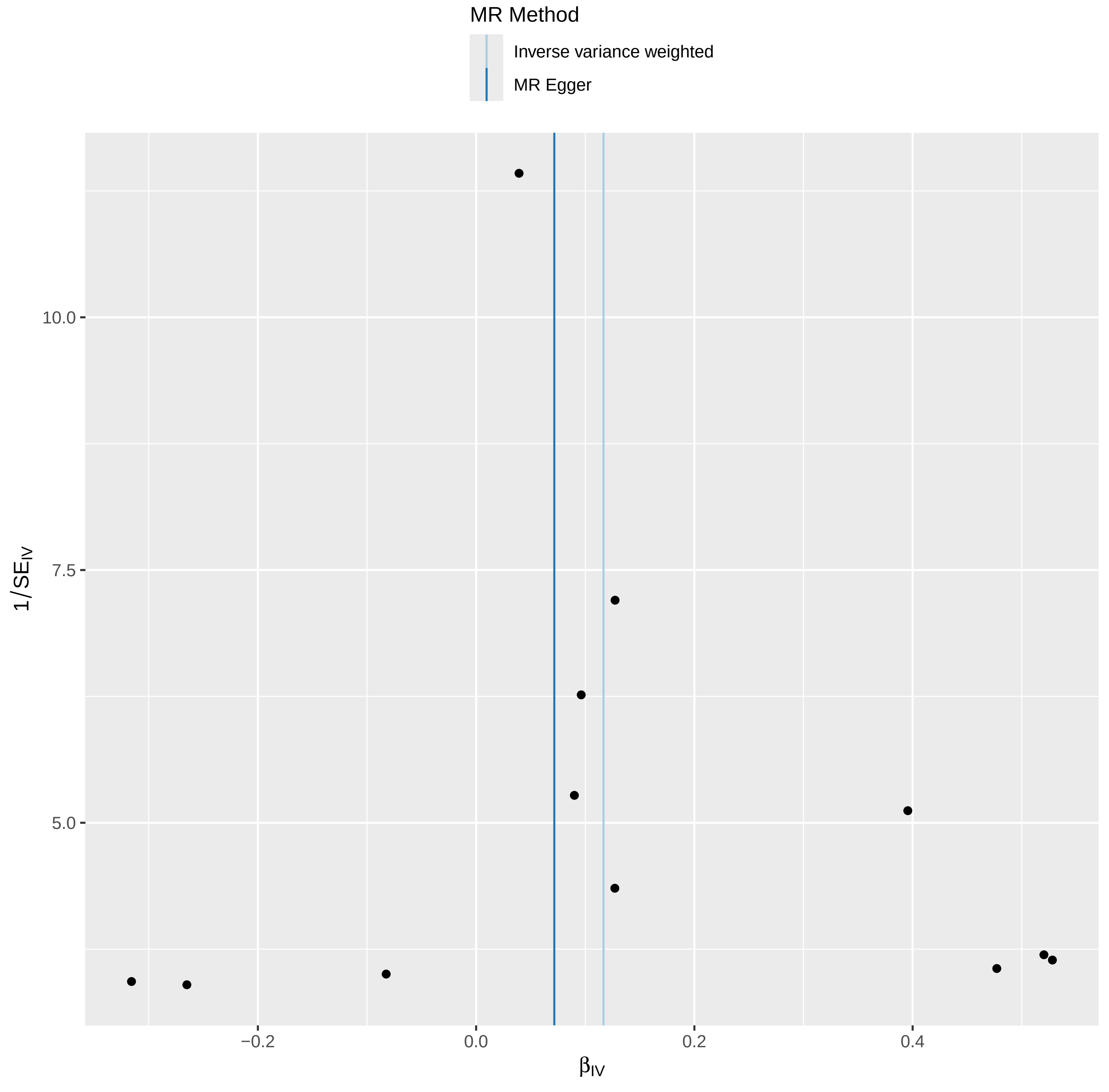
MR Method
Inverse variance weighted
MR Egger
10.0
7.5
5.0
$1/SE_{IV}$
−0.2
0.0
0.2
0.4
β_{IV}

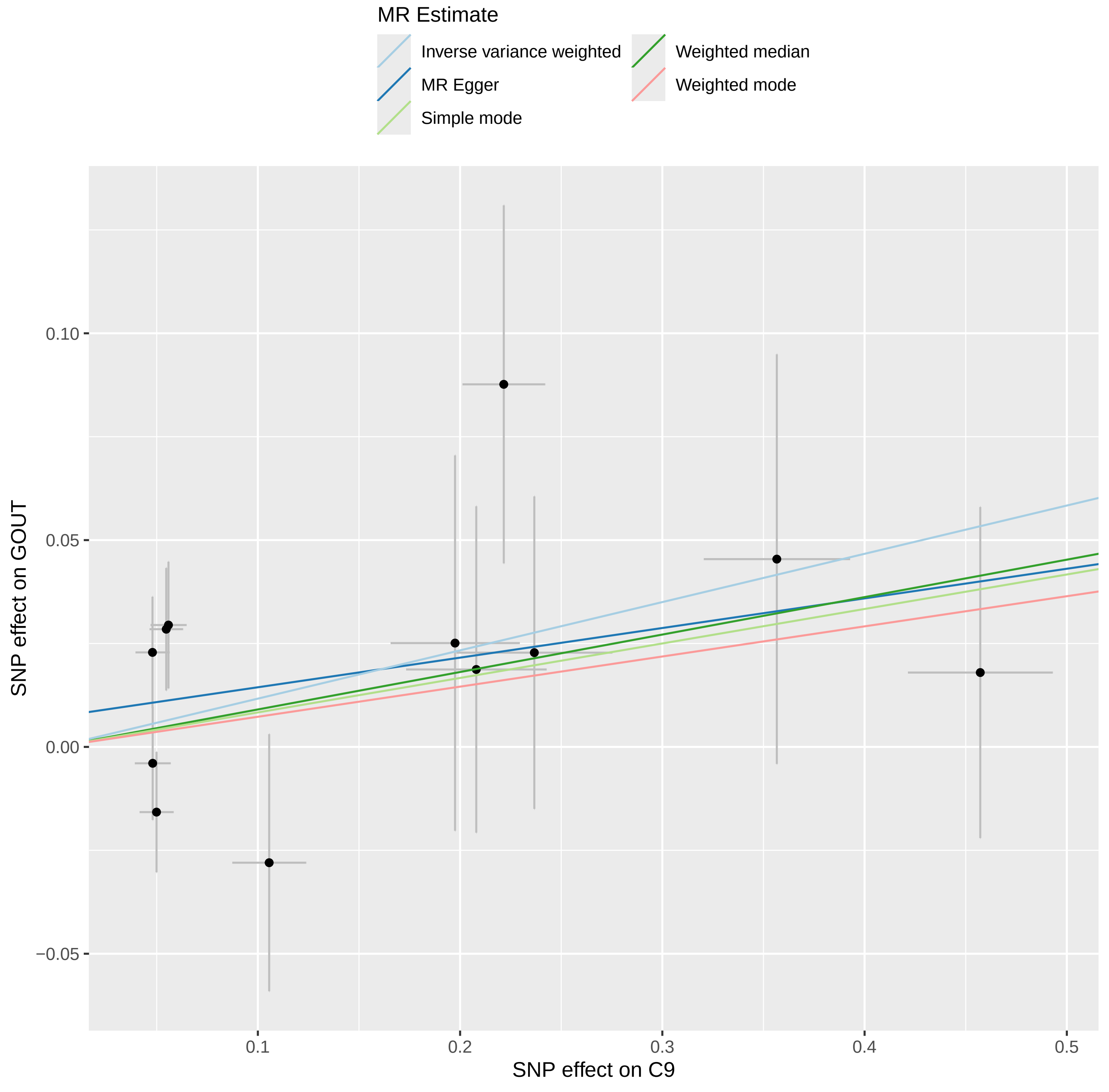
MR Estimate
Inverse variance weighted
MR Egger
Simple mode
Weighted median
Weighted mode
SNP effect on GOUT
0.10
0.05
0.00
−0.05
0.1
0.2
0.3
0.4
0.5
SNP effect on C9

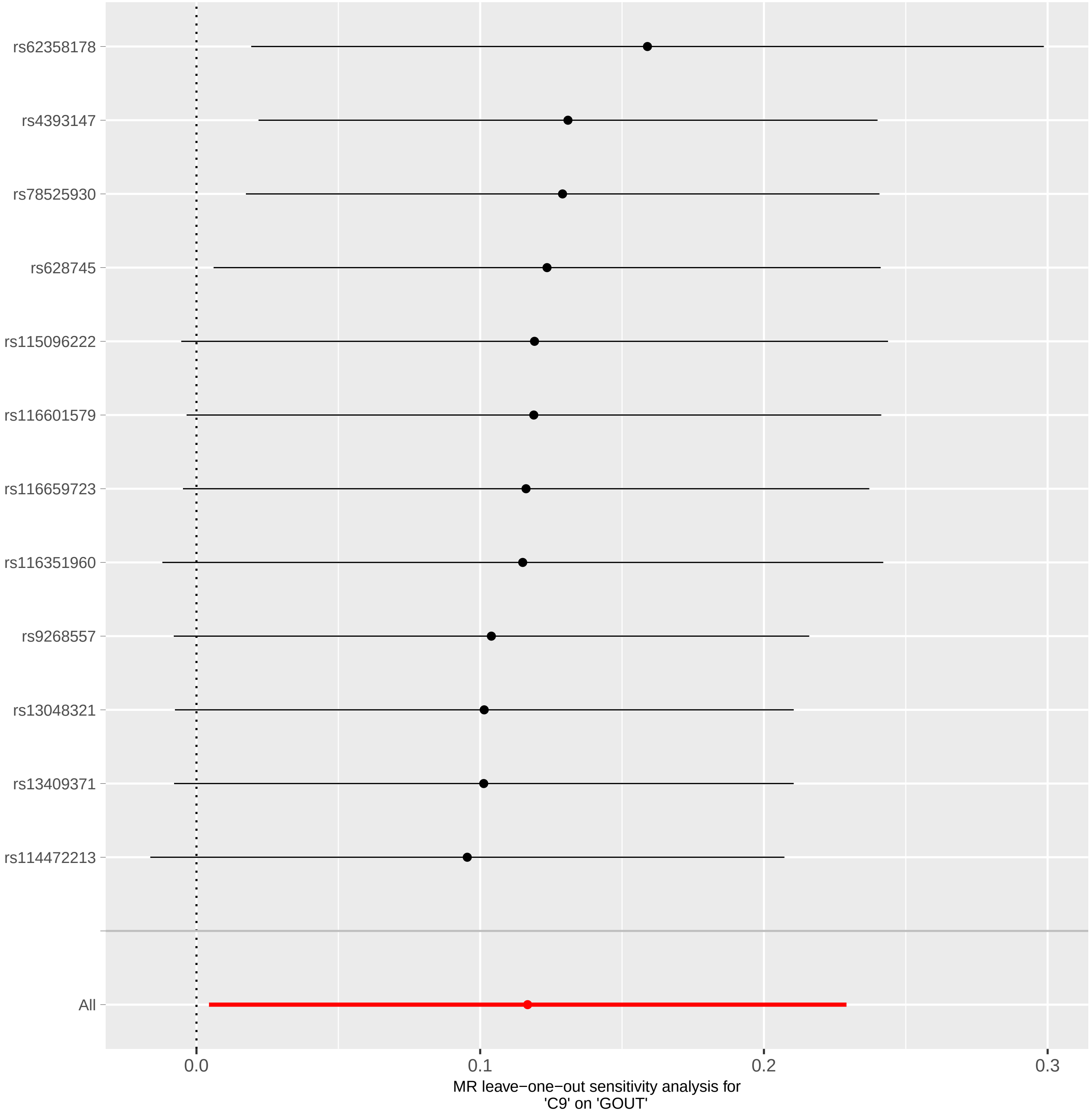
rs62358178
rs4393147
rs78525930
rs628745
rs115096222
rs116601579
rs116659723
rs116351960
rs9268557
rs13048321
rs13409371
rs114472213
All
0.0
0.1
0.2
0.3
MR leave−one−out sensitivity analysis for
'C9' on 'GOUT'

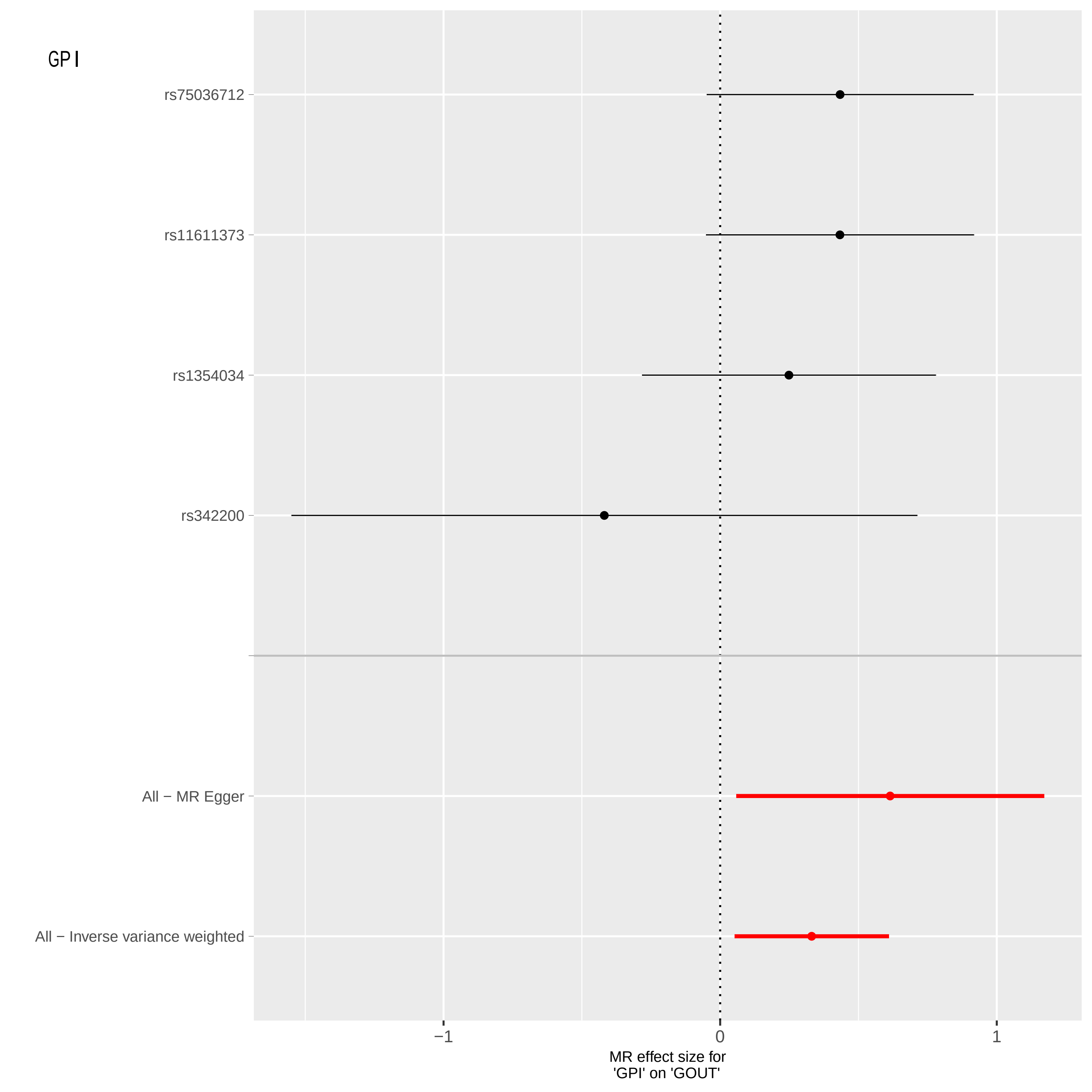

GPI
rs75036712
rs11611373
rs1354034
rs342200
All − MR Egger
All − Inverse variance weighted
−1
0
1
MR effect size for
'GPI' on 'GOUT'

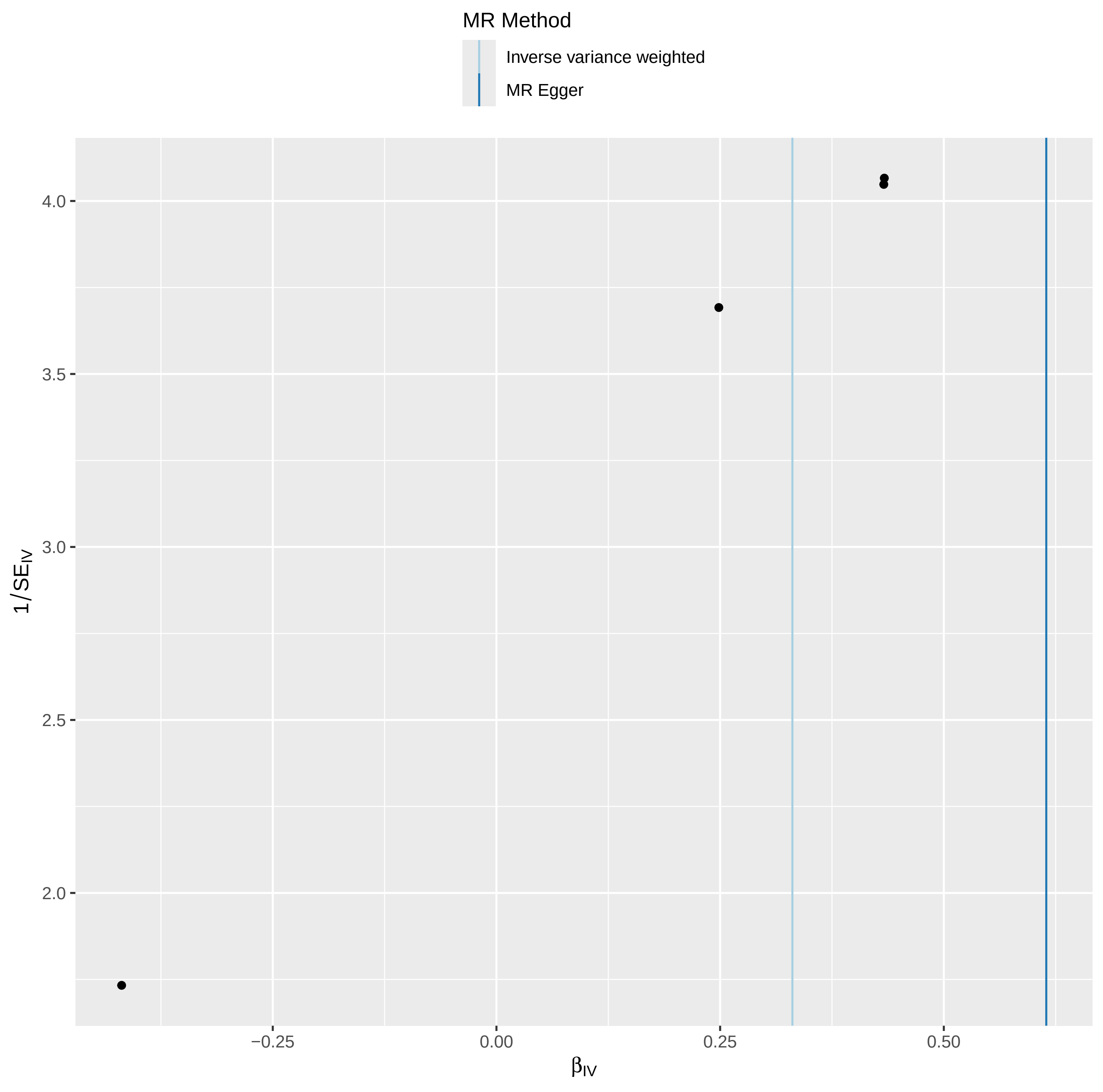
MR Method
Inverse variance weighted
MR Egger
4.0
3.5
3.0
2.5
2.0
1/SE$_{IV}$
−0.25
0.00
0.25
0.50
β$_{IV}$

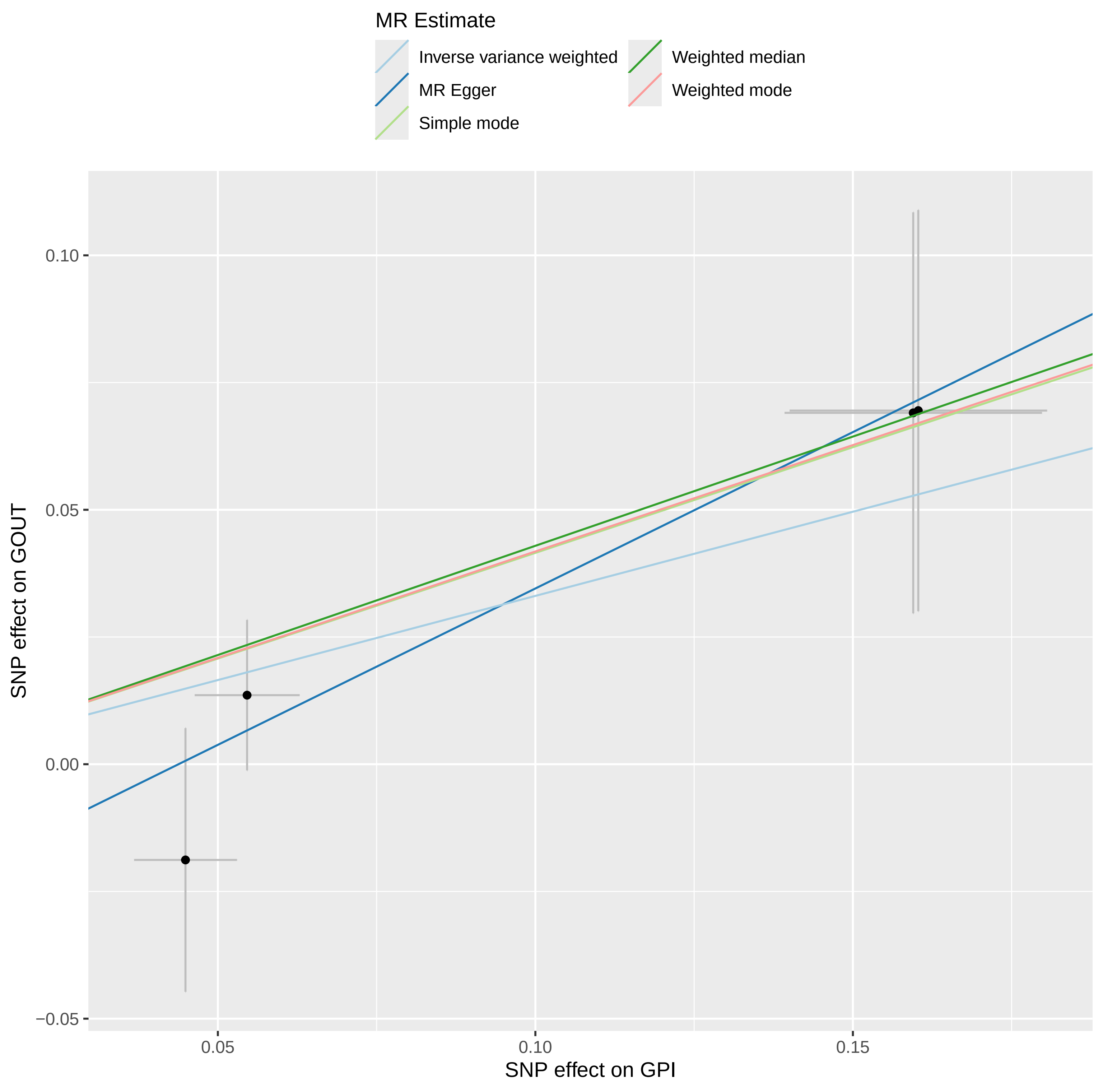
MR Estimate
Inverse variance weighted
MR Egger
Simple mode
Weighted median
Weighted mode
SNP effect on GOUT
0.10
0.05
0.00
−0.05
SNP effect on GPI
0.05
0.10
0.15

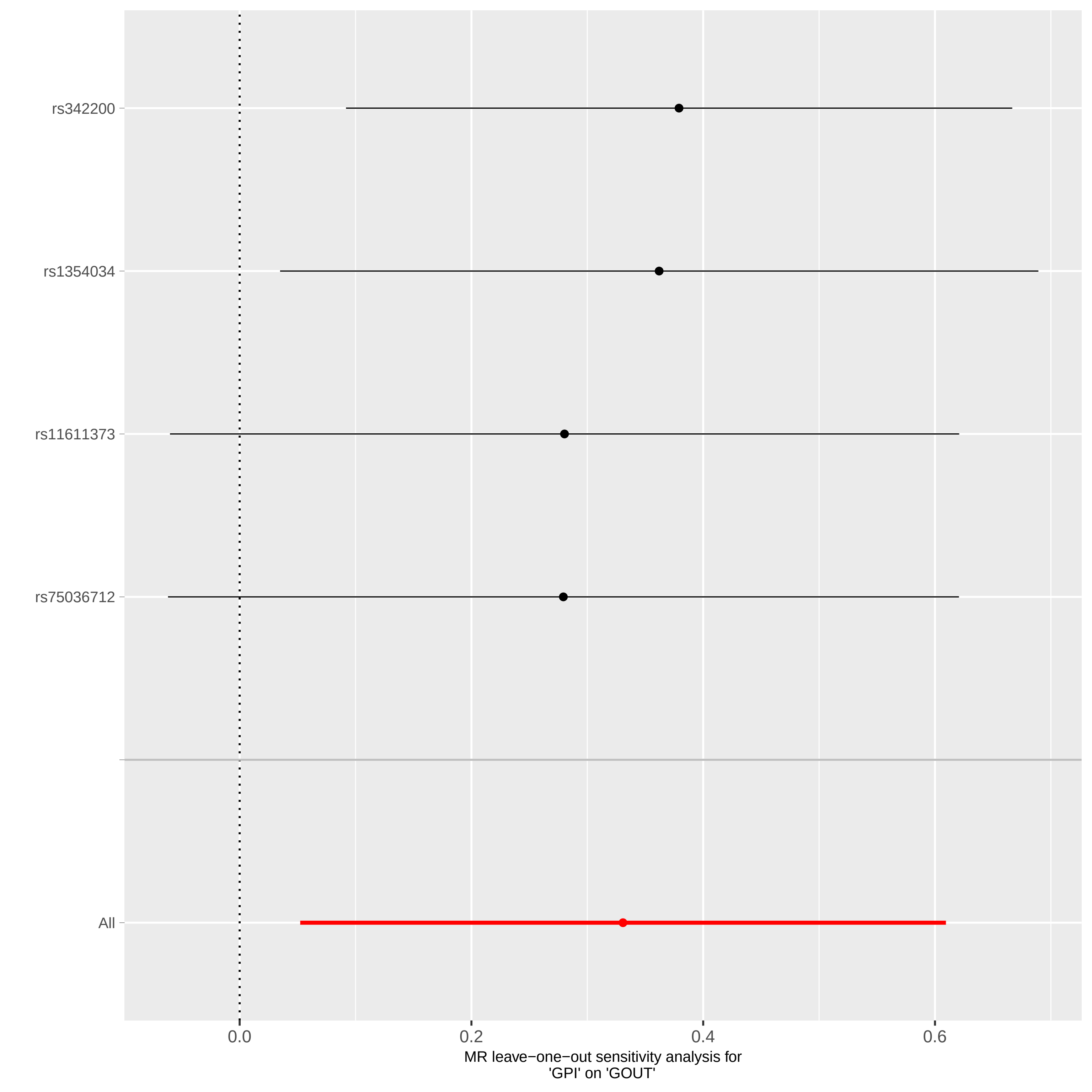

rs342200
rs1354034
rs11611373
rs75036712
All
0.0
0.2
0.4
0.6
MR leave−one−out sensitivity analysis for
'GPI' on 'GOUT'

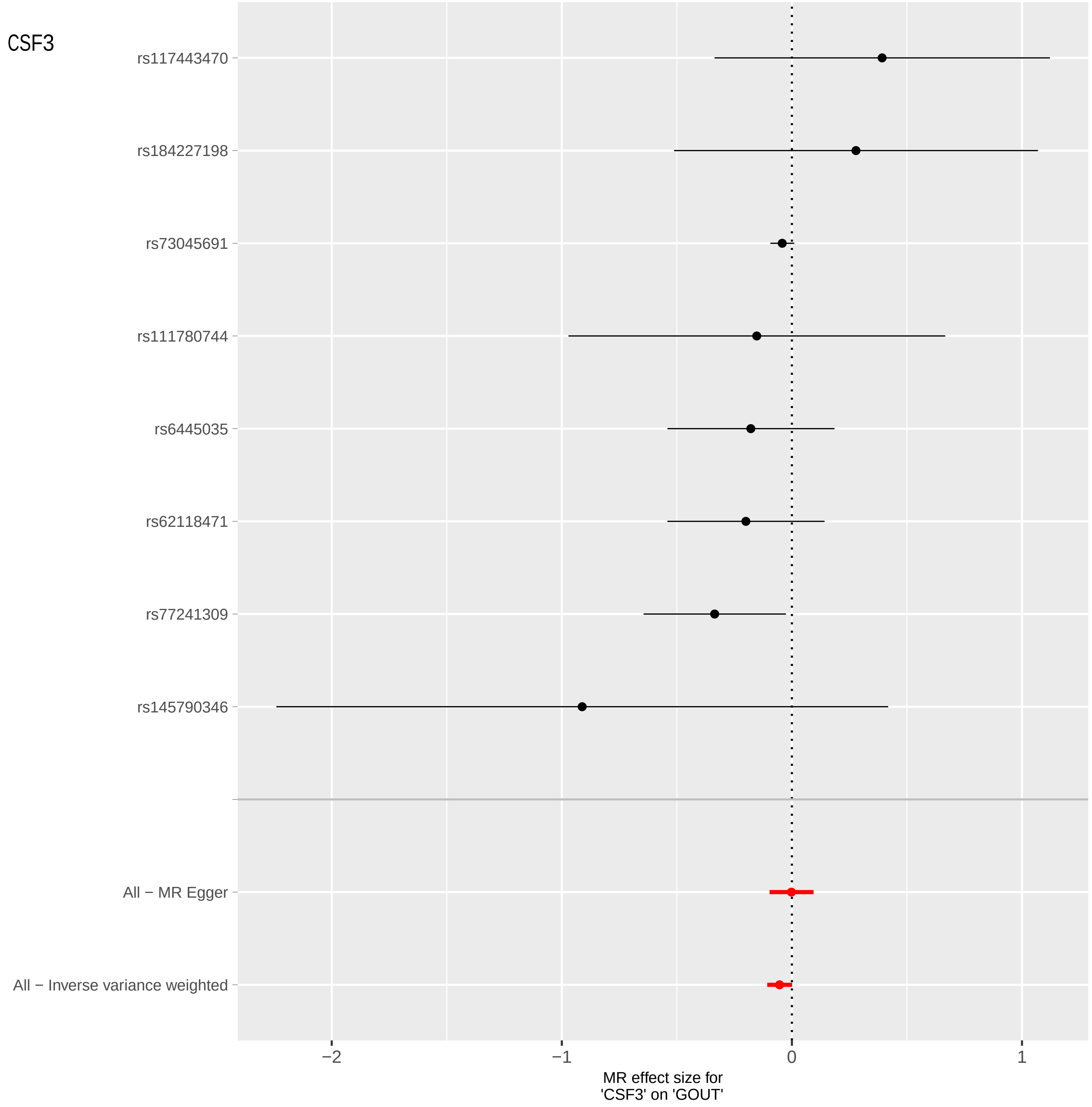

CSF3
rs117443470
rs184227198
rs73045691
rs111780744
rs6445035
rs62118471
rs77241309
rs145790346
All − MR Egger
All − Inverse variance weighted
−2
−1
0
1
MR effect size for
'CSF3' on 'GOUT'

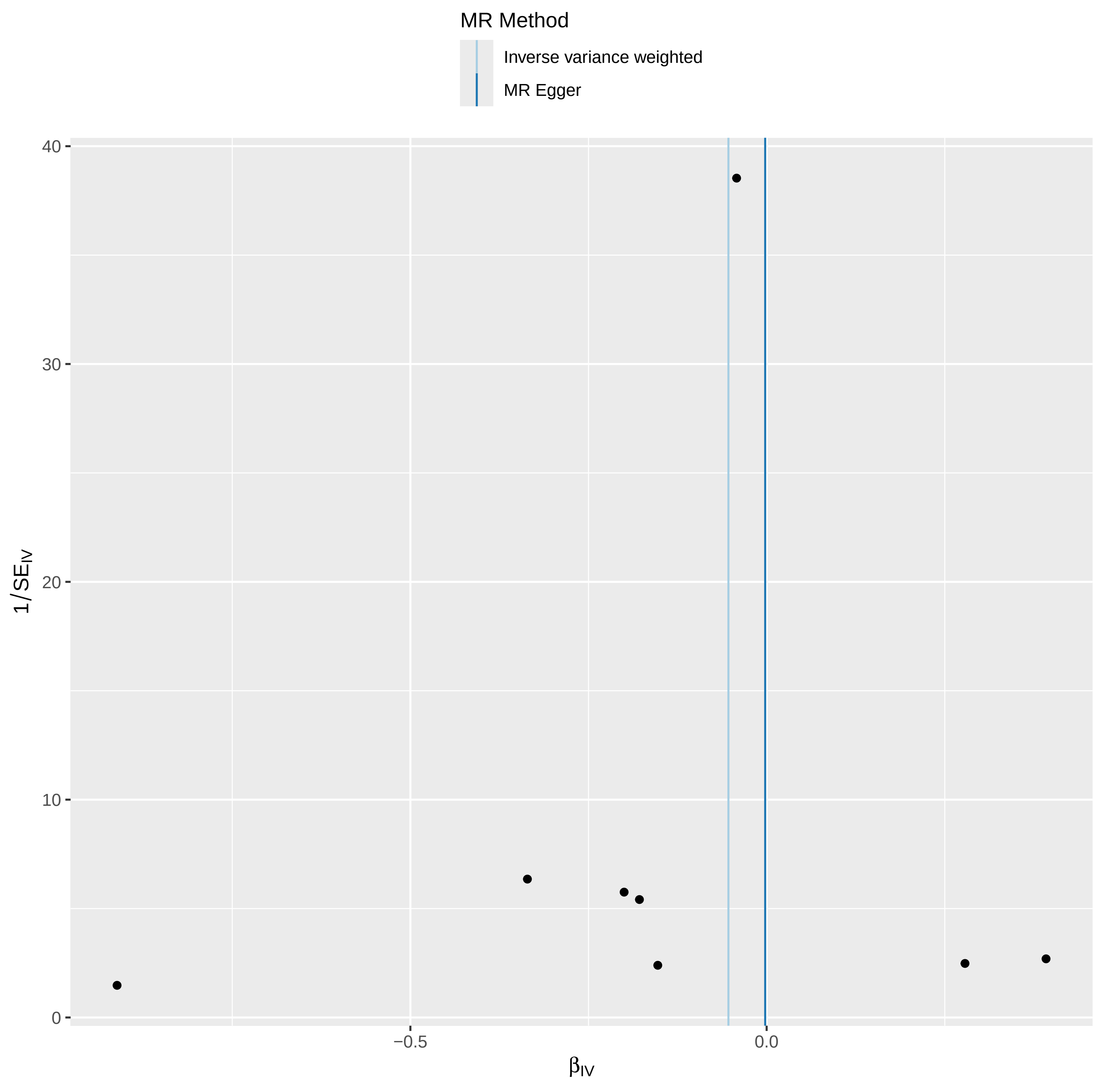

MR Method
Inverse variance weighted
MR Egger
40
30
20
10
0
1/SE$_{IV}$
−0.5
0.0
β$_{IV}$

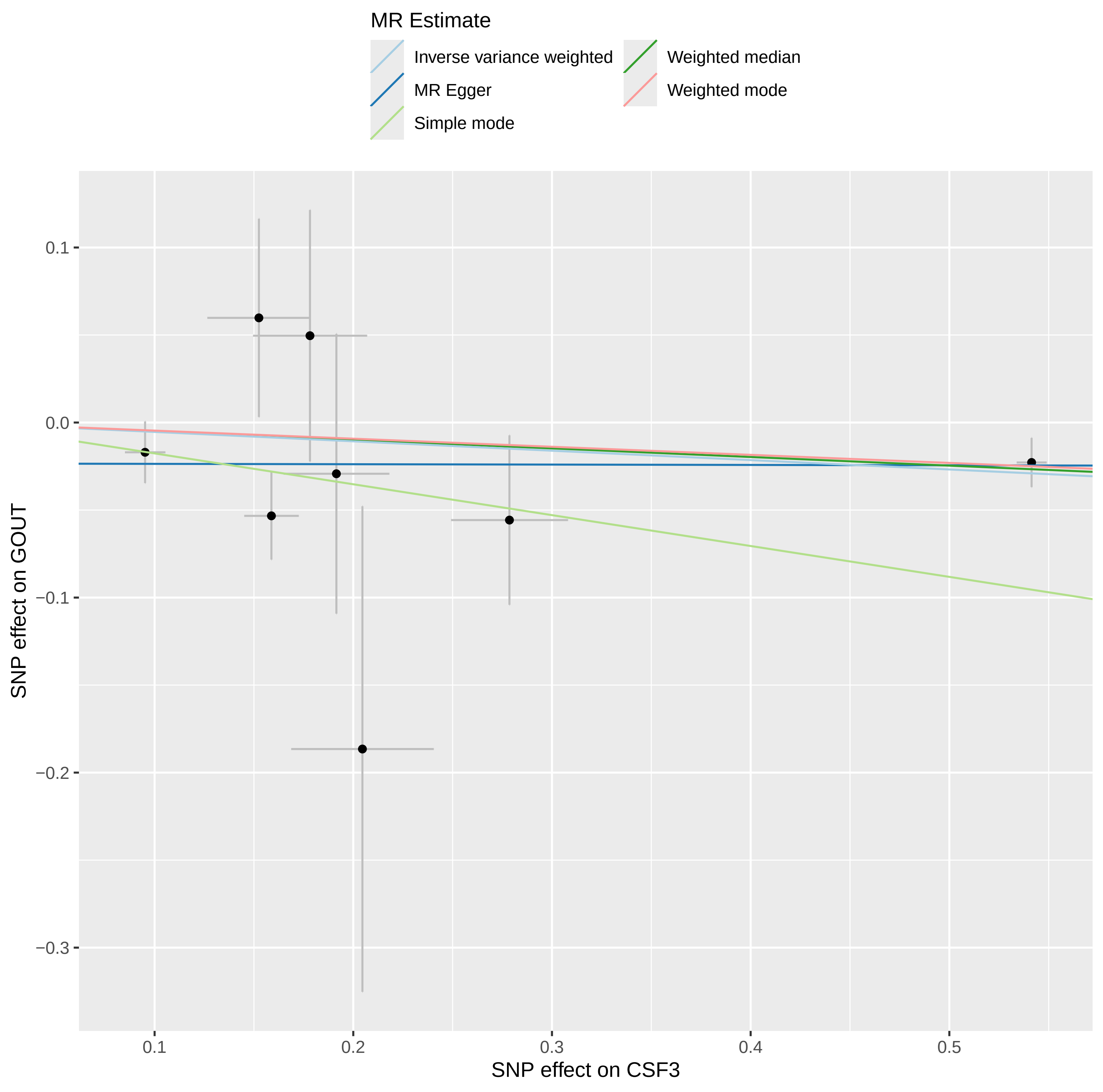
MR Estimate
Inverse variance weighted
MR Egger
Simple mode
Weighted median
Weighted mode
0.1
0.0
−0.1
−0.2
−0.3
SNP effect on GOUT
0.1
0.2
0.3
0.4
0.5
SNP effect on CSF3

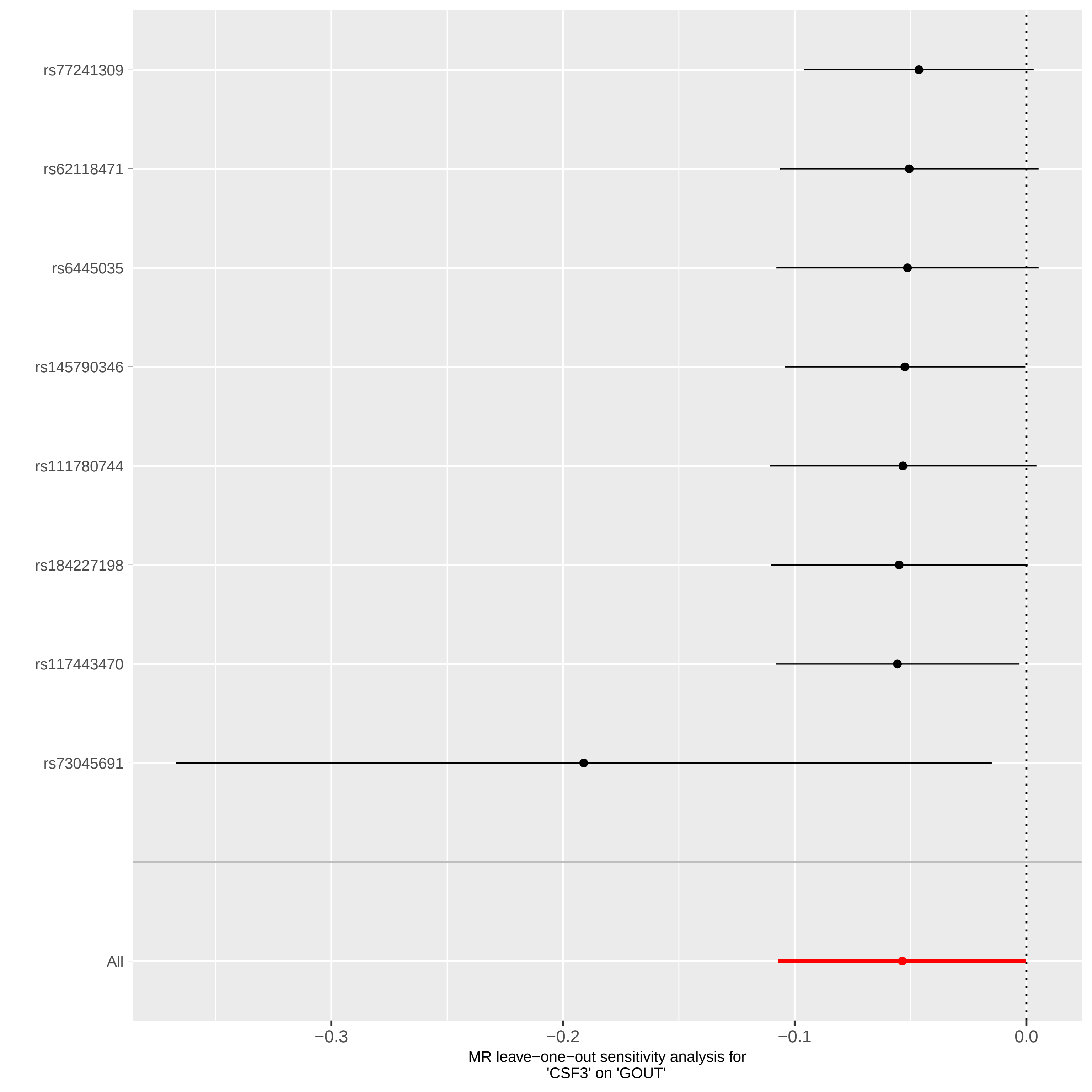
rs77241309
rs62118471
rs6445035
rs145790346
rs111780744
rs184227198
rs117443470
rs73045691
All
−0.3
−0.2
−0.1
0.0
MR leave−one−out sensitivity analysis for
'CSF3' on 'GOUT'

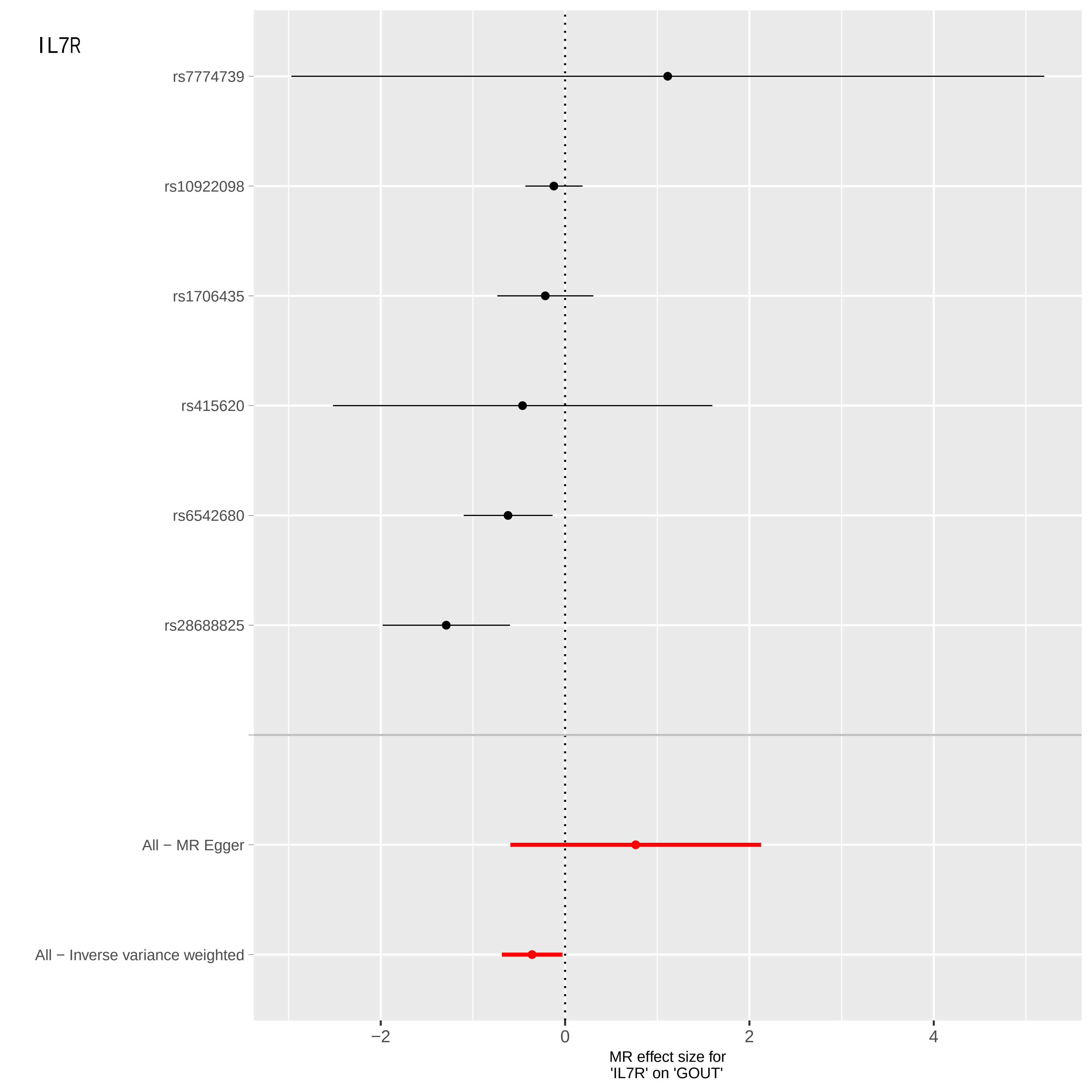

IL7R
rs7774739
rs10922098
rs1706435
rs415620
rs6542680
rs28688825
All − MR Egger
All − Inverse variance weighted
−2
0
2
4
MR effect size for
'IL7R' on 'GOUT'

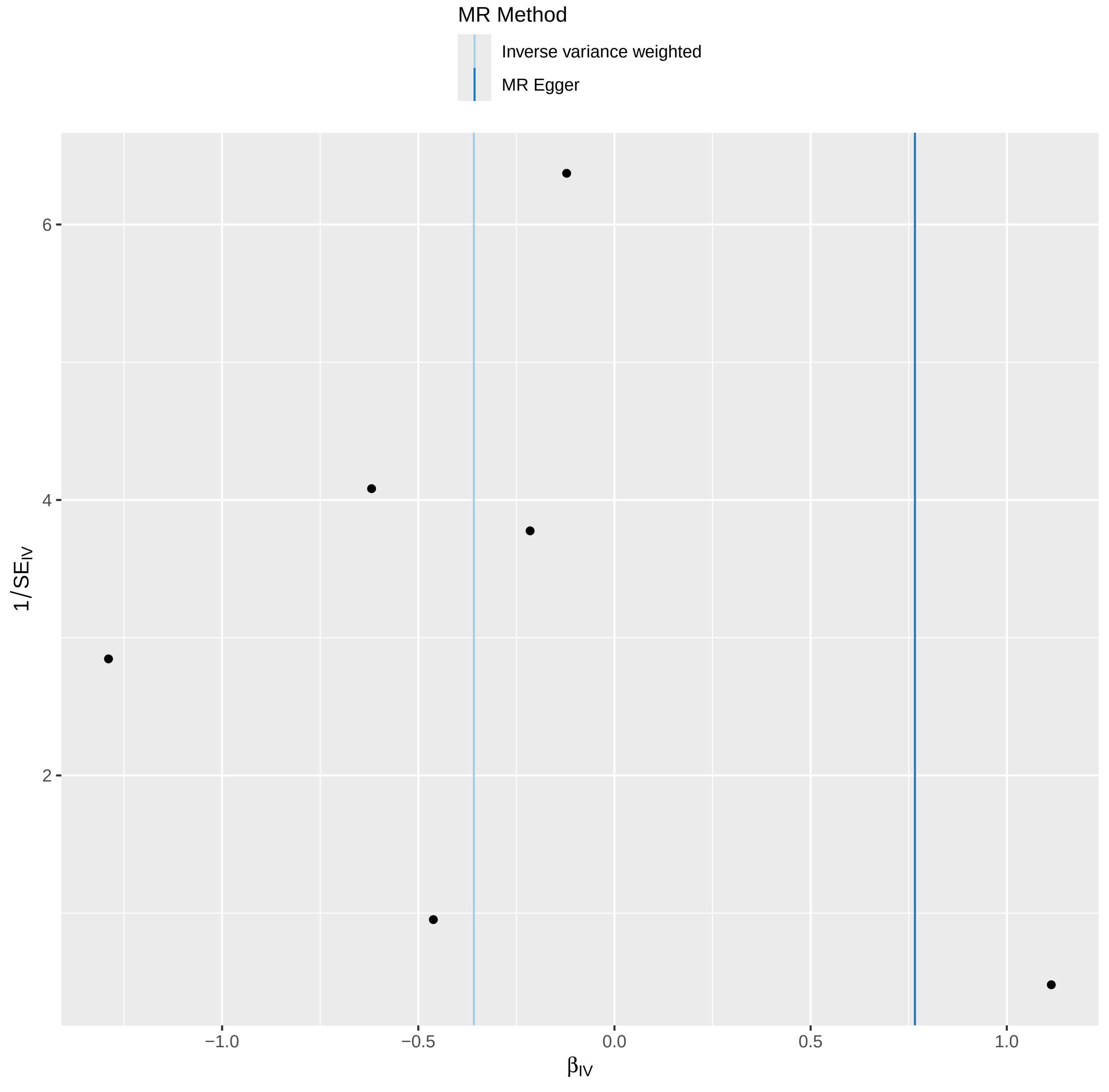

MR Method
Inverse variance weighted
MR Egger
6
4
2
$1/SE_{IV}$
−1.0
−0.5
0.0
0.5
1.0
β_{IV}

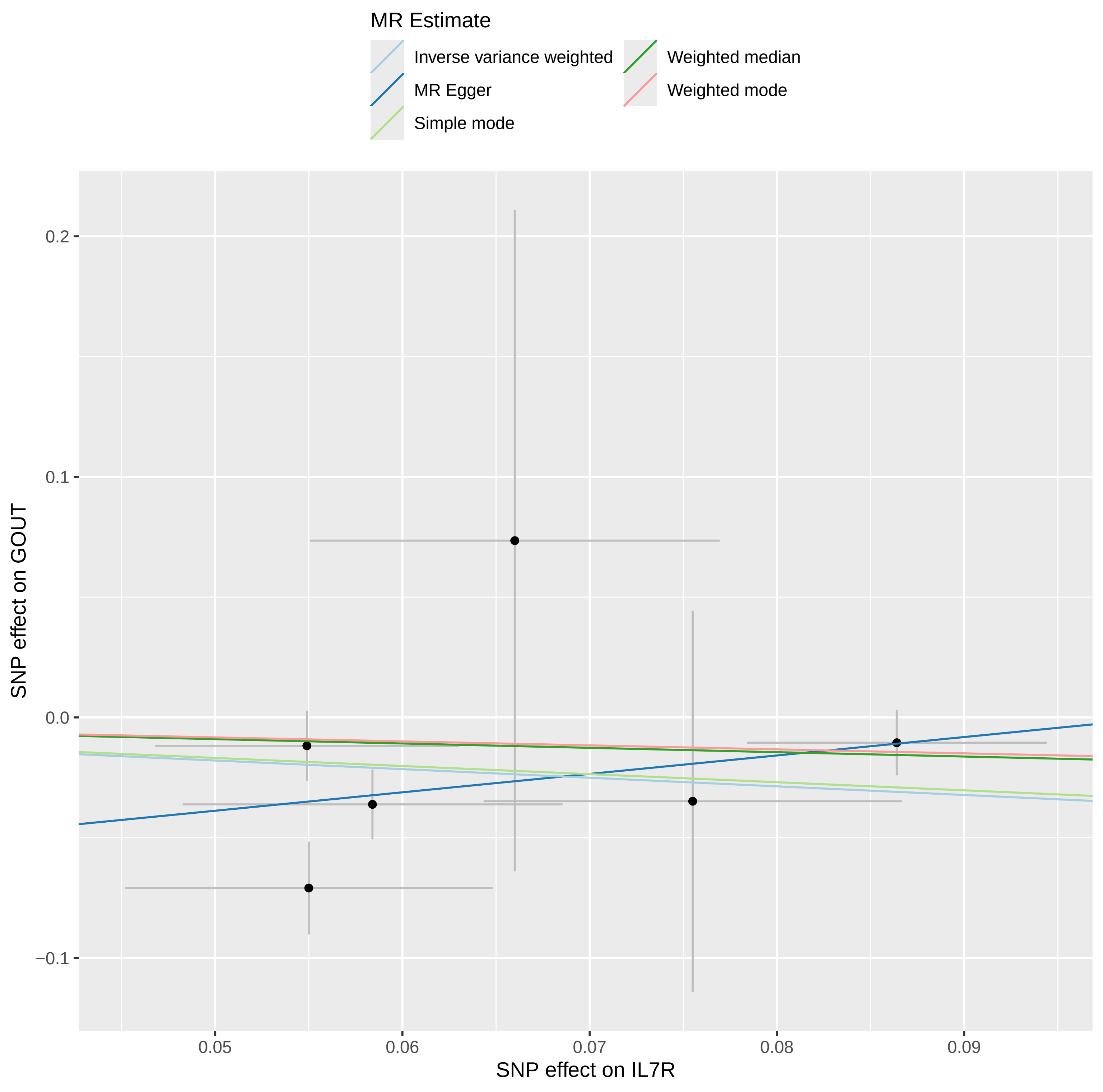

MR Estimate
Inverse variance weighted
MR Egger
Simple mode
Weighted median
Weighted mode
0.2
0.1
0.0
−0.1
SNP effect on GOUT
0.05
0.06
0.07
0.08
0.09
SNP effect on IL7R

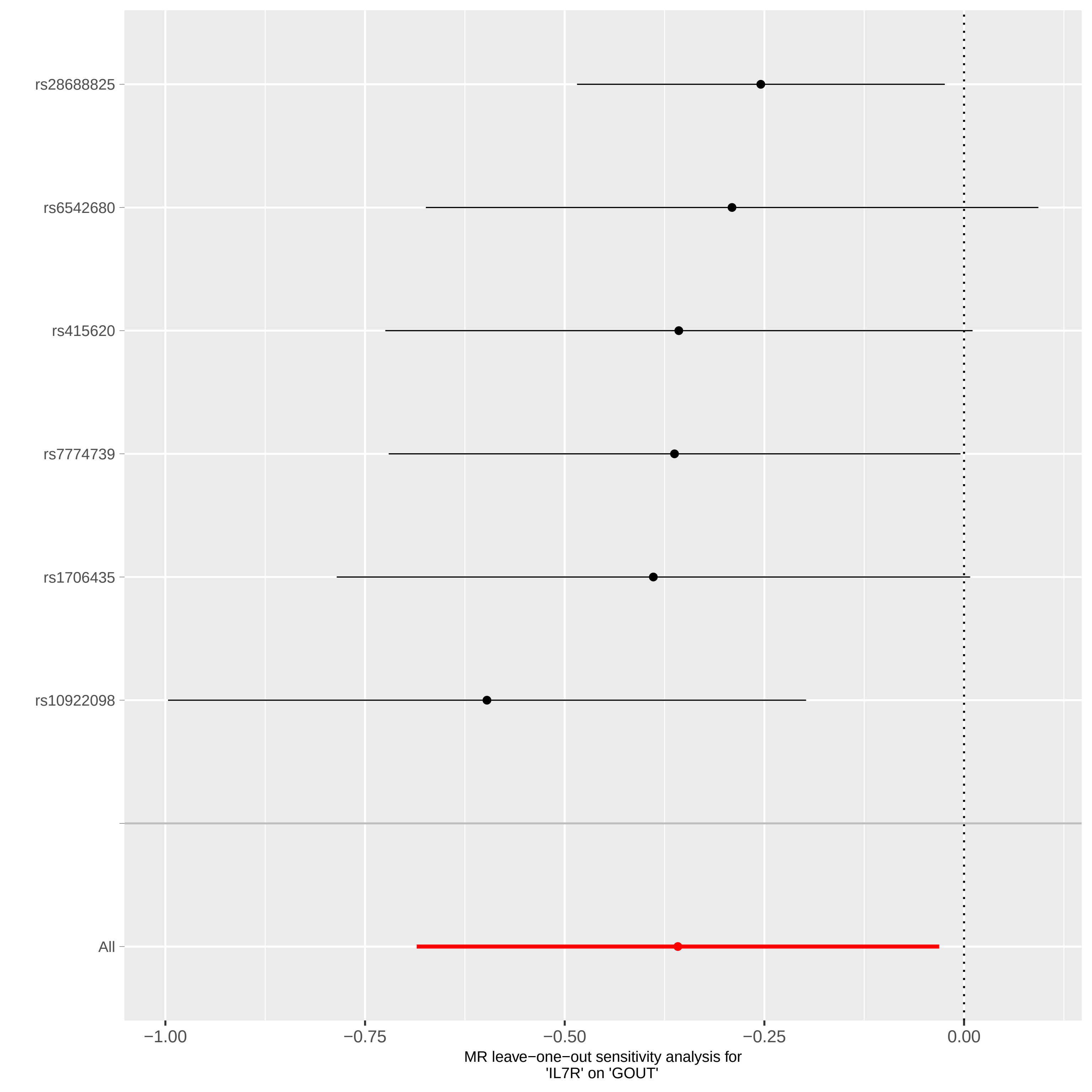
rs28688825
rs6542680
rs415620
rs7774739
rs1706435
rs10922098
All
−1.00
−0.75
−0.50
−0.25
0.00
MR leave−one−out sensitivity analysis for
'IL7R' on 'GOUT'

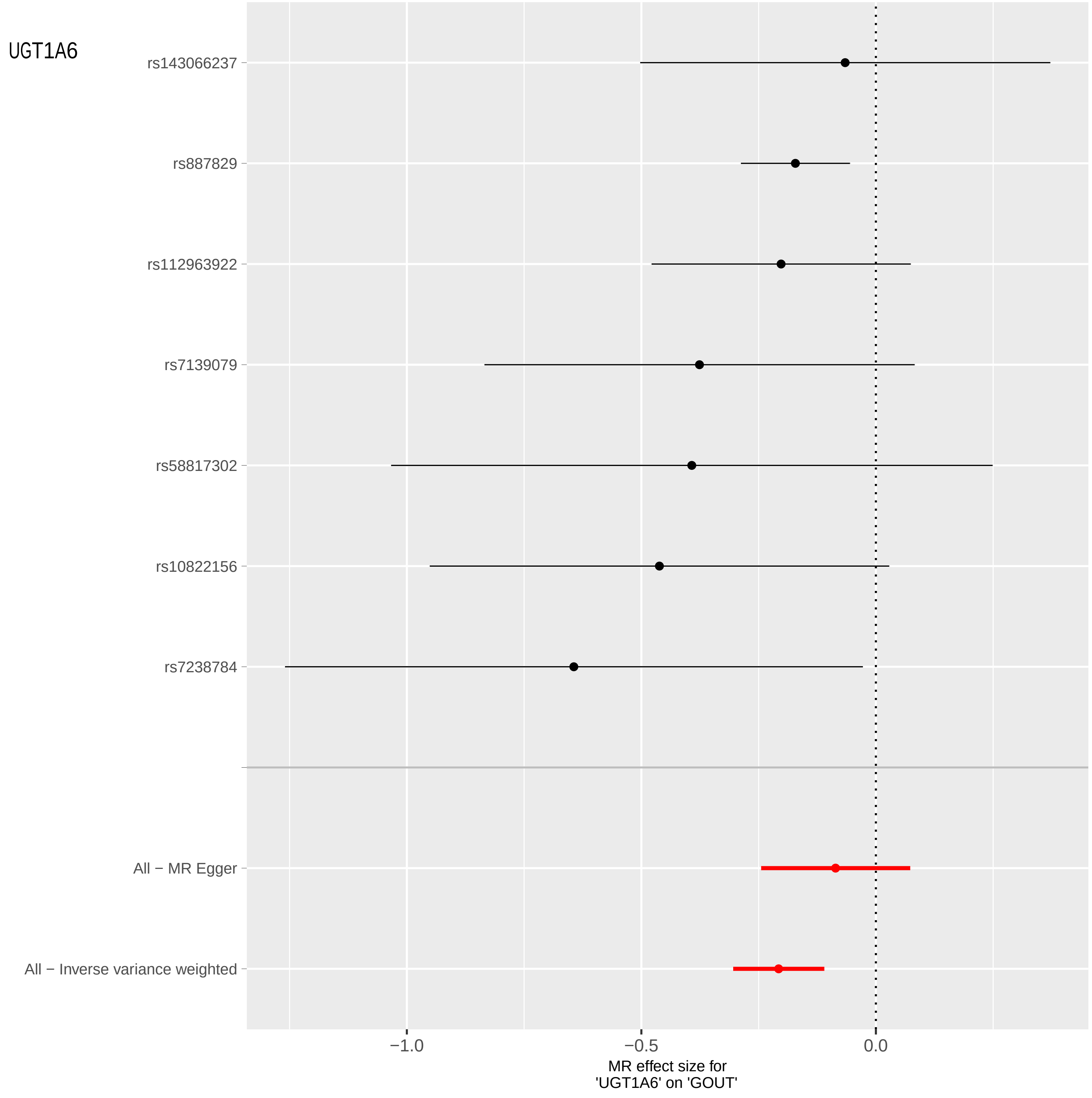

UGT1A6
rs143066237
rs887829
rs112963922
rs7139079
rs58817302
rs10822156
rs7238784
All − MR Egger
All − Inverse variance weighted
−1.0
−0.5
0.0
MR effect size for
'UGT1A6' on 'GOUT'

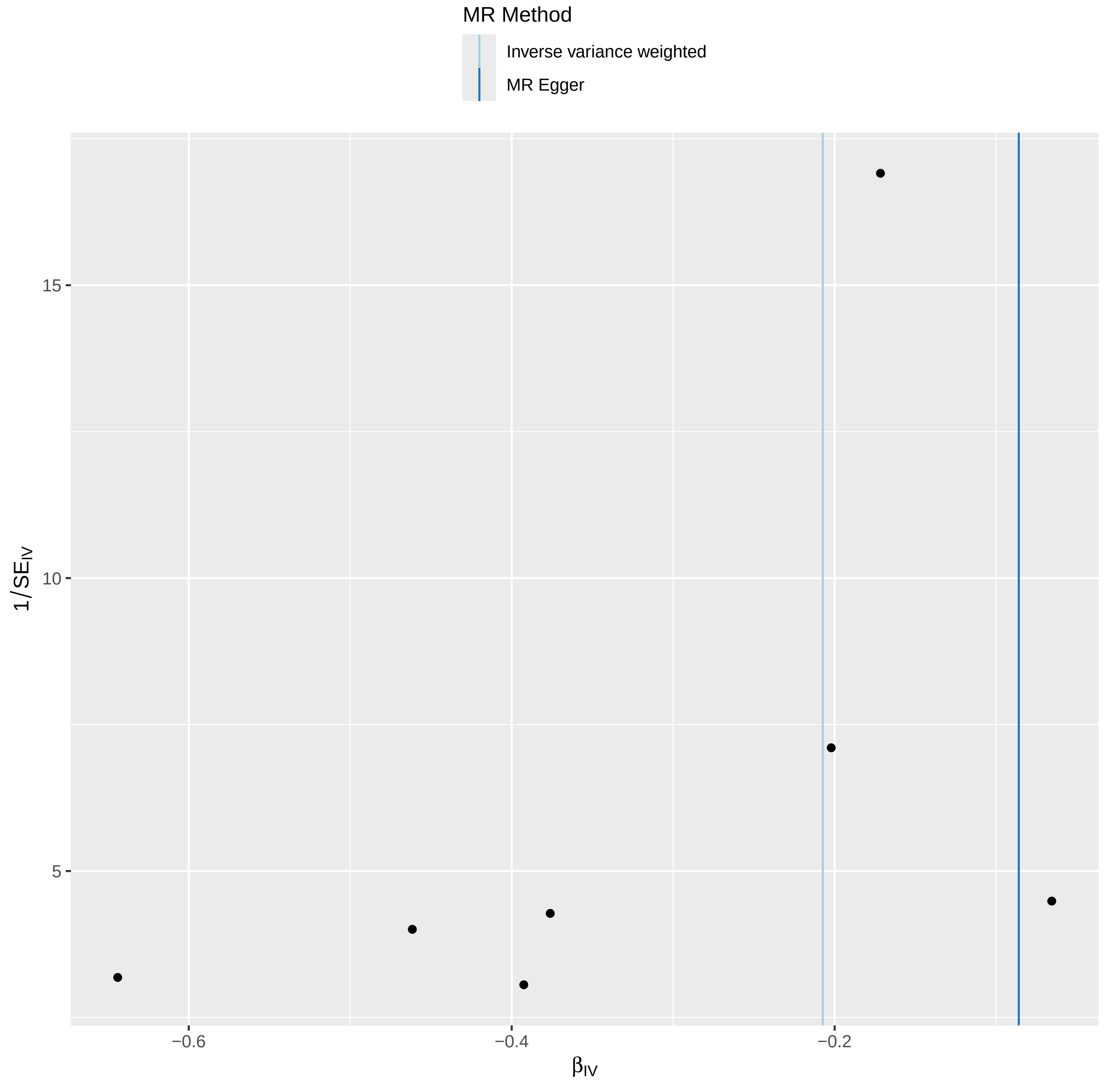

MR Method
Inverse variance weighted
MR Egger
15
10
5
1/SE$_{IV}$
−0.6
−0.4
−0.2
β$_{IV}$

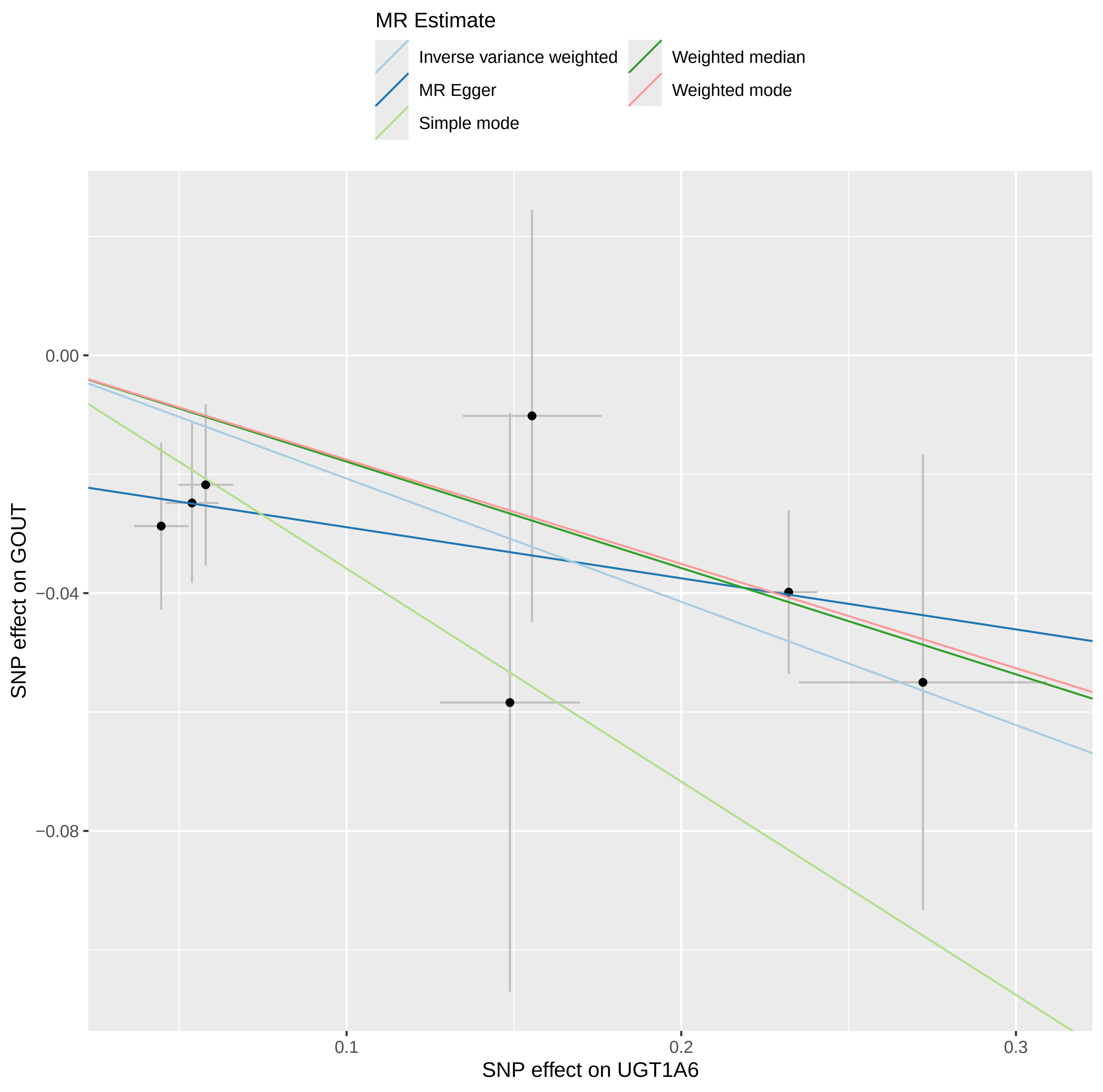
MR Estimate
Inverse variance weighted
MR Egger
Simple mode
Weighted median
Weighted mode
0.00
−0.04
−0.08
0.1
0.2
0.3
SNP effect on GOUT
SNP effect on UGT1A6

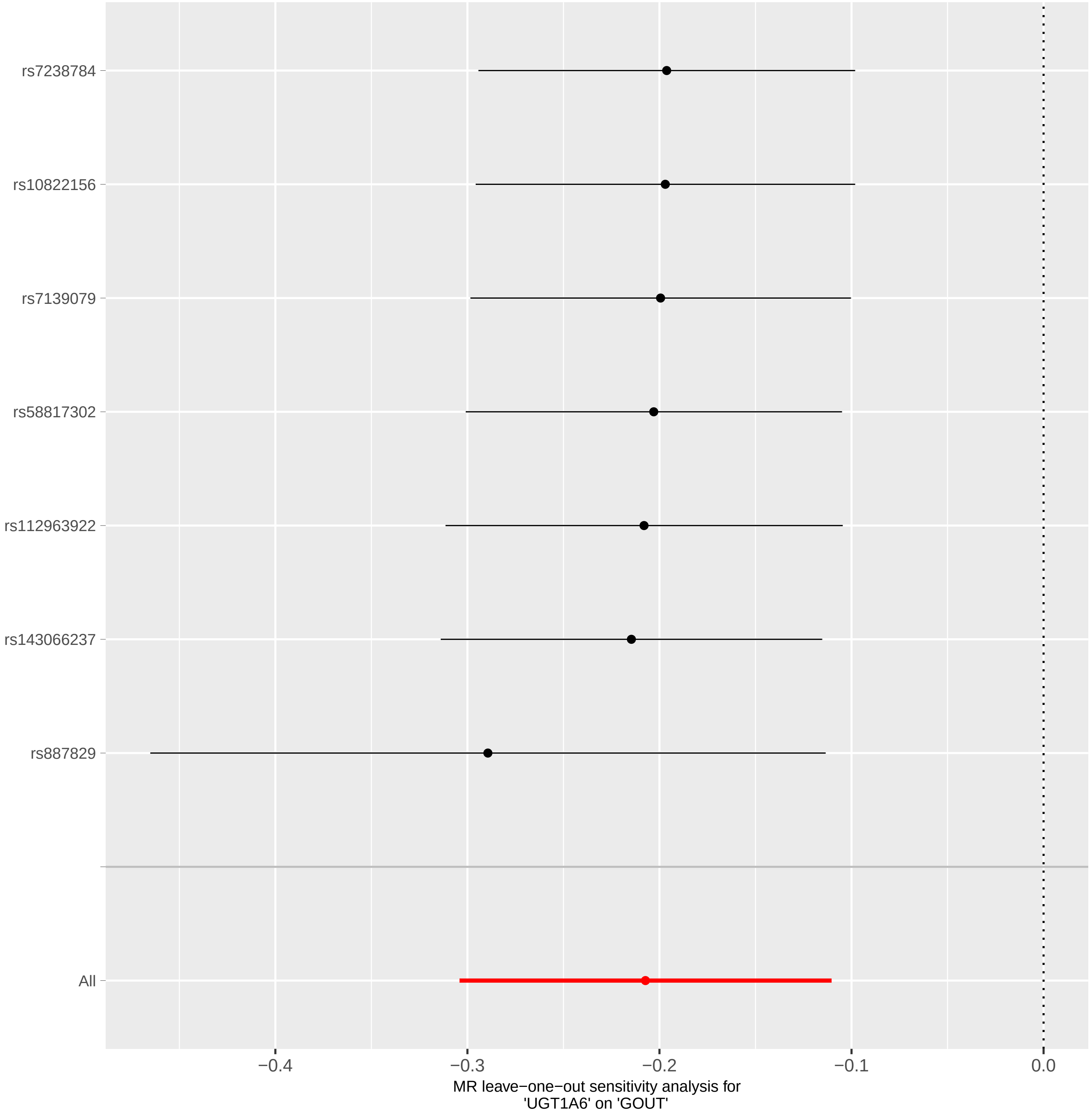

rs7238784
rs10822156
rs7139079
rs58817302
rs112963922
rs143066237
rs887829
All
−0.4
−0.3
−0.2
−0.1
0.0
MR leave−one−out sensitivity analysis for
'UGT1A6' on 'GOUT'

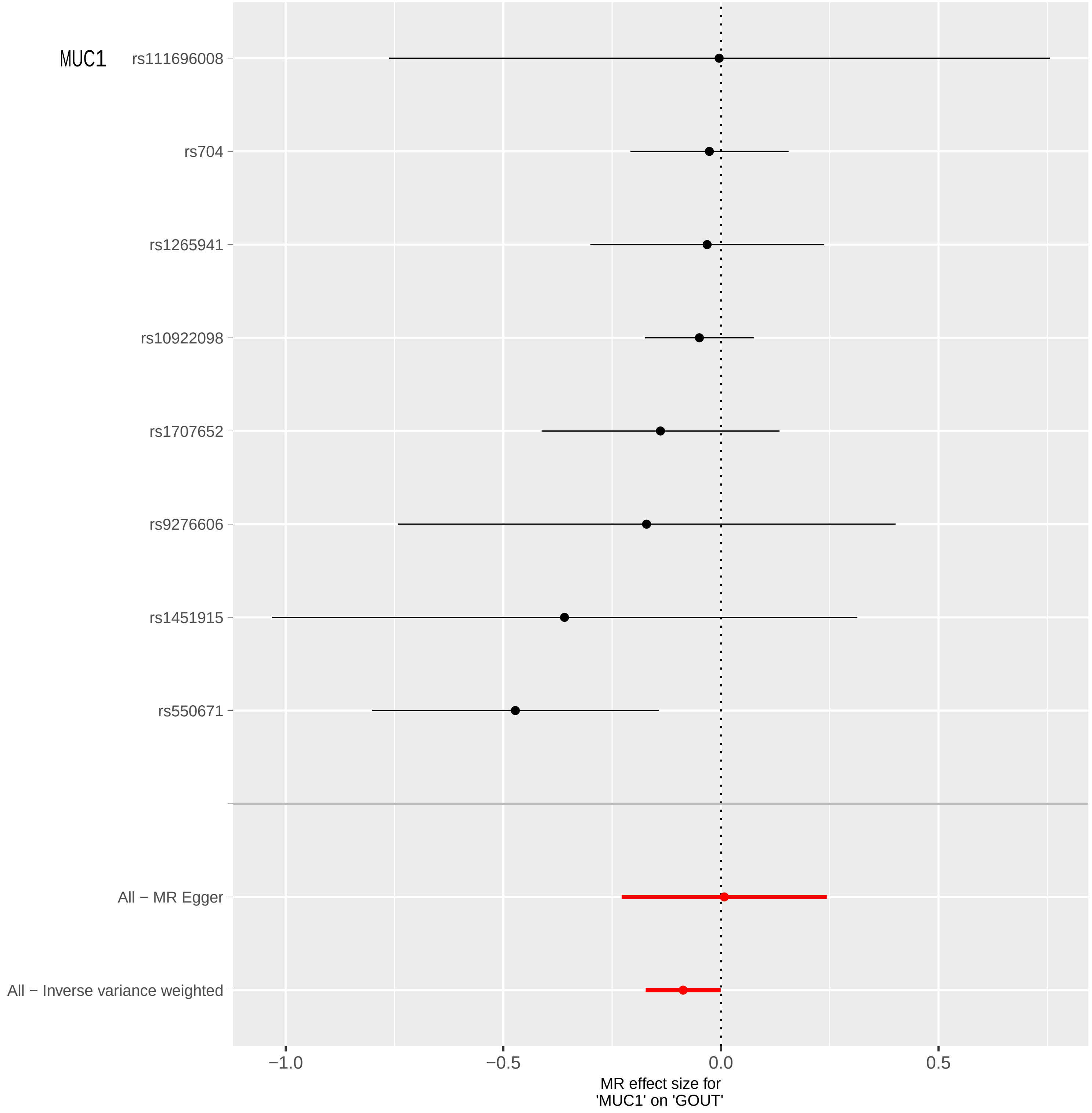

MUC1
rs111696008
rs704
rs1265941
rs10922098
rs1707652
rs9276606
rs1451915
rs550671
All − MR Egger
All − Inverse variance weighted
−1.0
−0.5
0.0
0.5
MR effect size for
'MUC1' on 'GOUT'

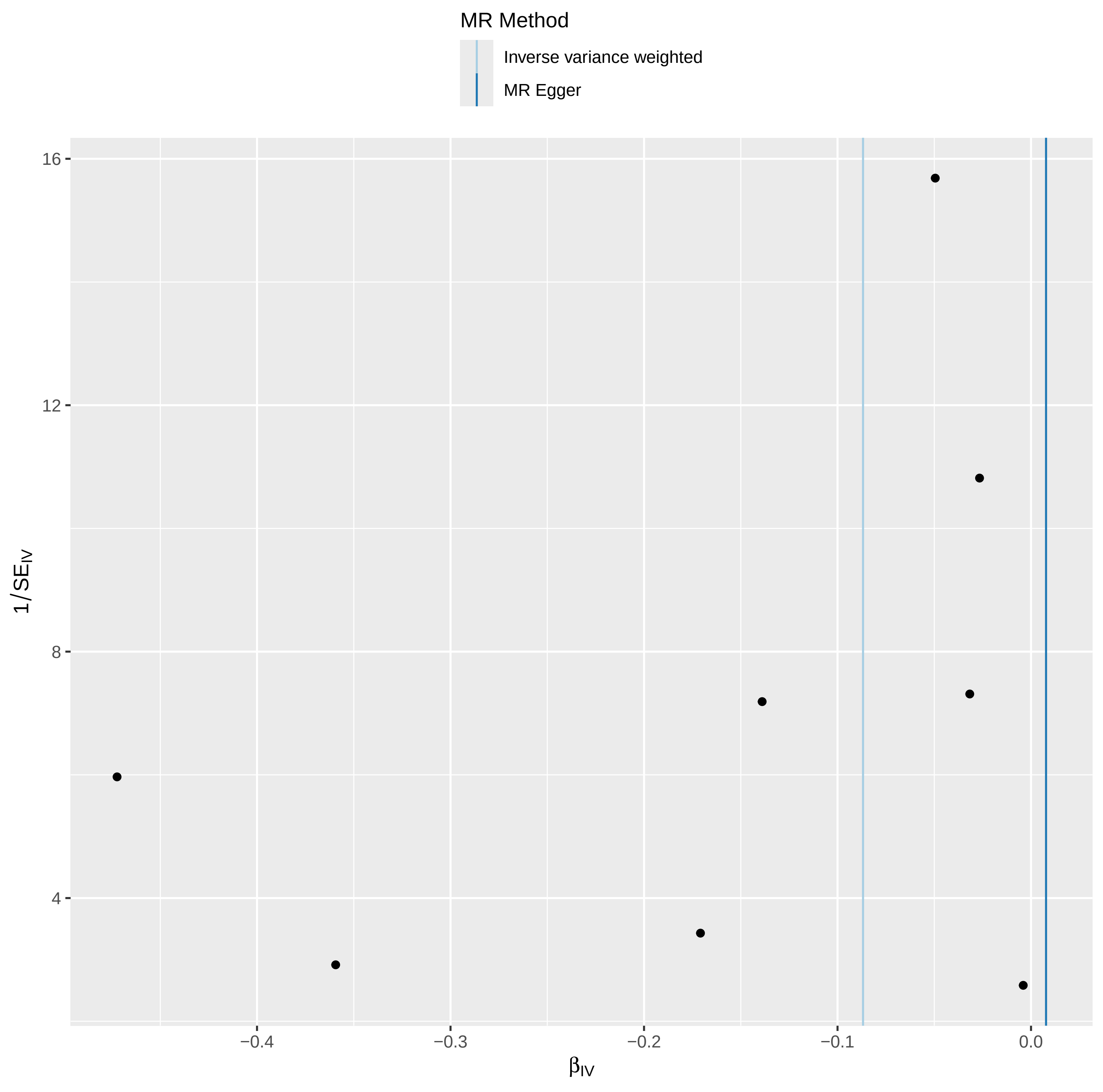

MR Method
Inverse variance weighted
MR Egger
16
12
8
4
1/SE_IV
−0.4
−0.3
−0.2
−0.1
0.0
β_IV

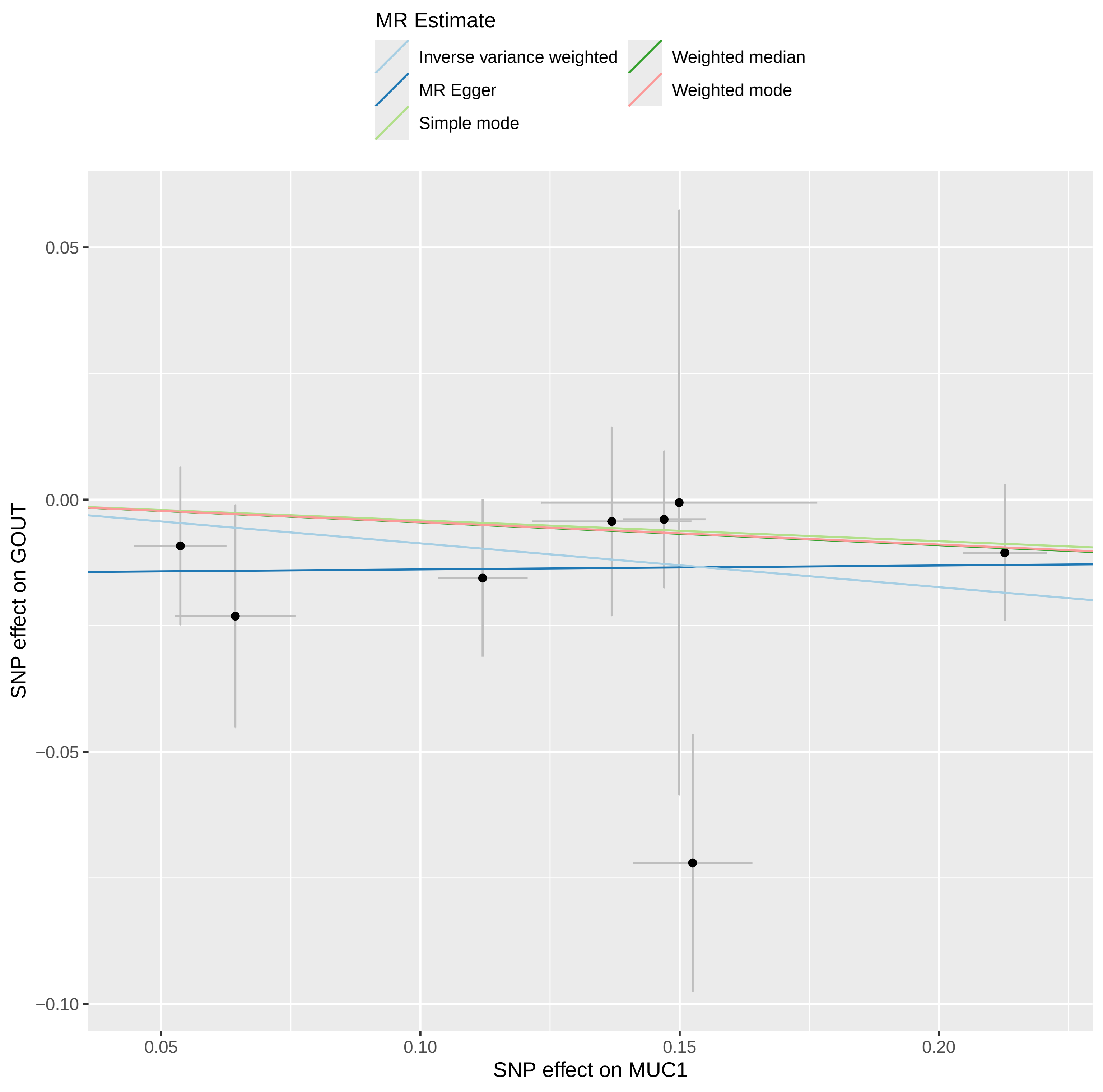
MR Estimate
Inverse variance weighted
MR Egger
Simple mode
Weighted median
Weighted mode
0.05
0.00
−0.05
−0.10
SNP effect on GOUT
0.05
0.10
0.15
0.20
SNP effect on MUC1

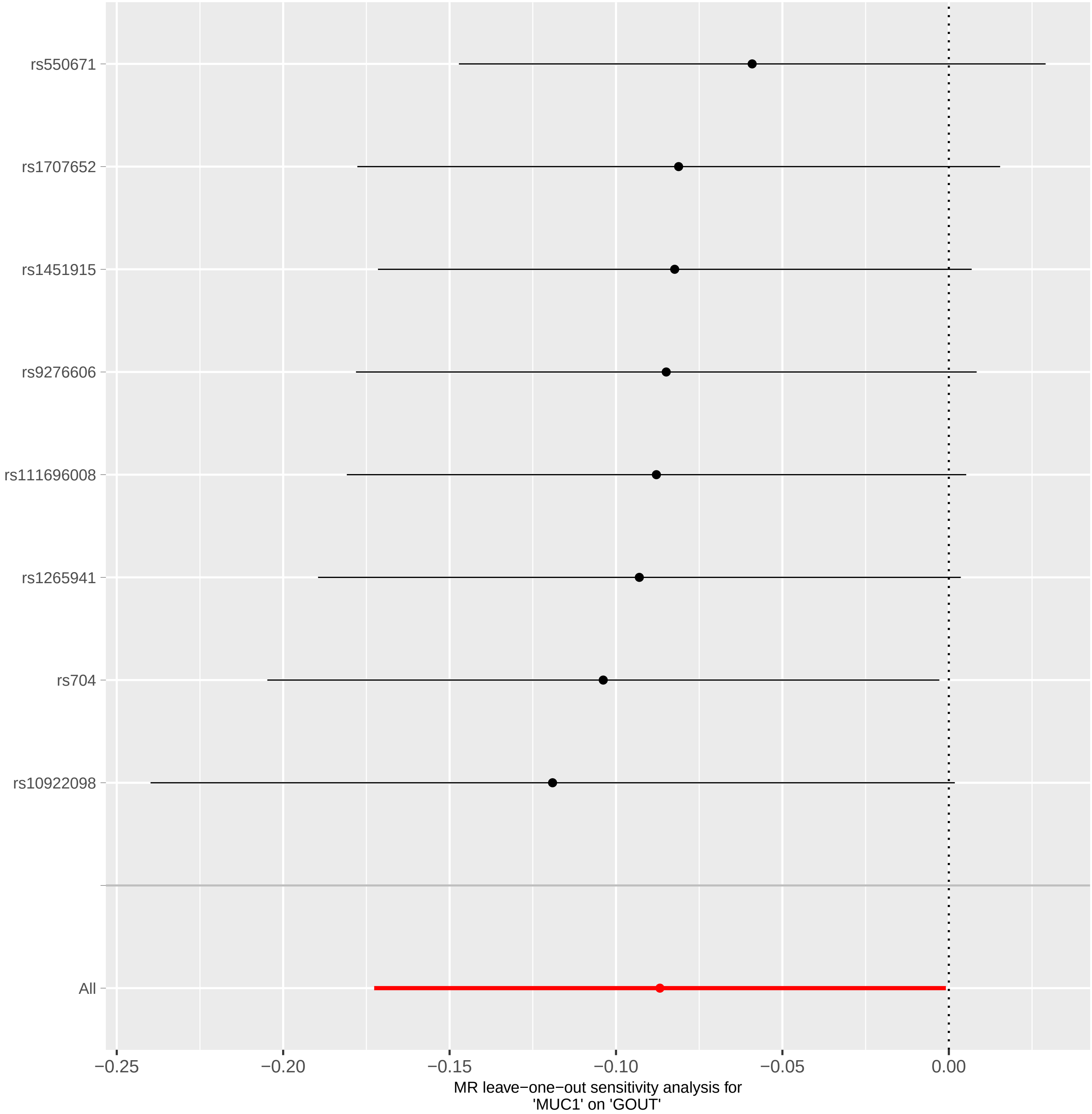
rs550671
rs1707652
rs1451915
rs9276606
rs111696008
rs1265941
rs704
rs10922098
All
−0.25
−0.20
−0.15
−0.10
−0.05
0.00
MR leave−one−out sensitivity analysis for
'MUC1' on 'GOUT'

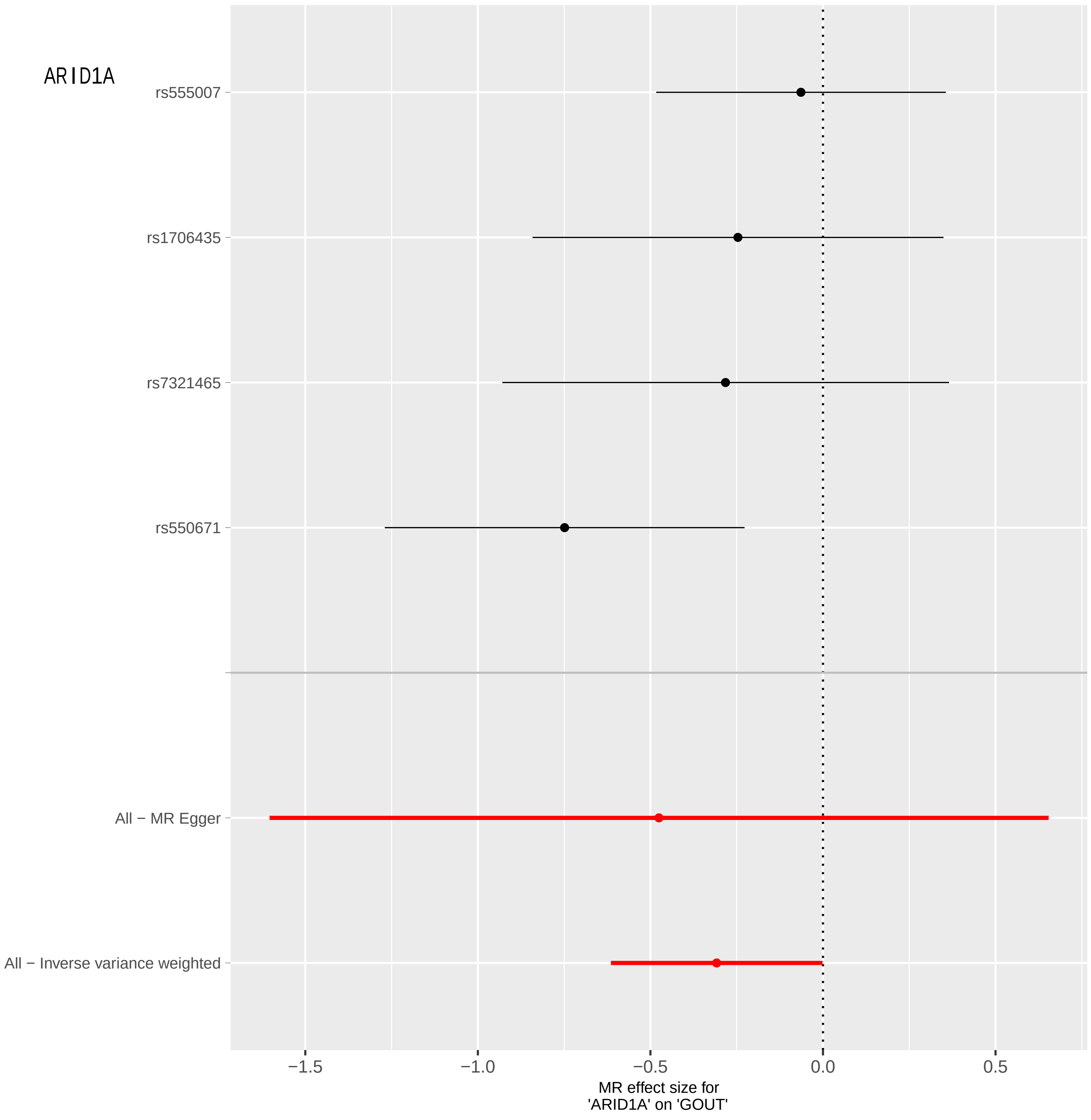
ARID1A
rs555007
rs1706435
rs7321465
rs550671
All − MR Egger
All − Inverse variance weighted
−1.5
−1.0
−0.5
0.0
0.5
MR effect size for
'ARID1A' on 'GOUT'

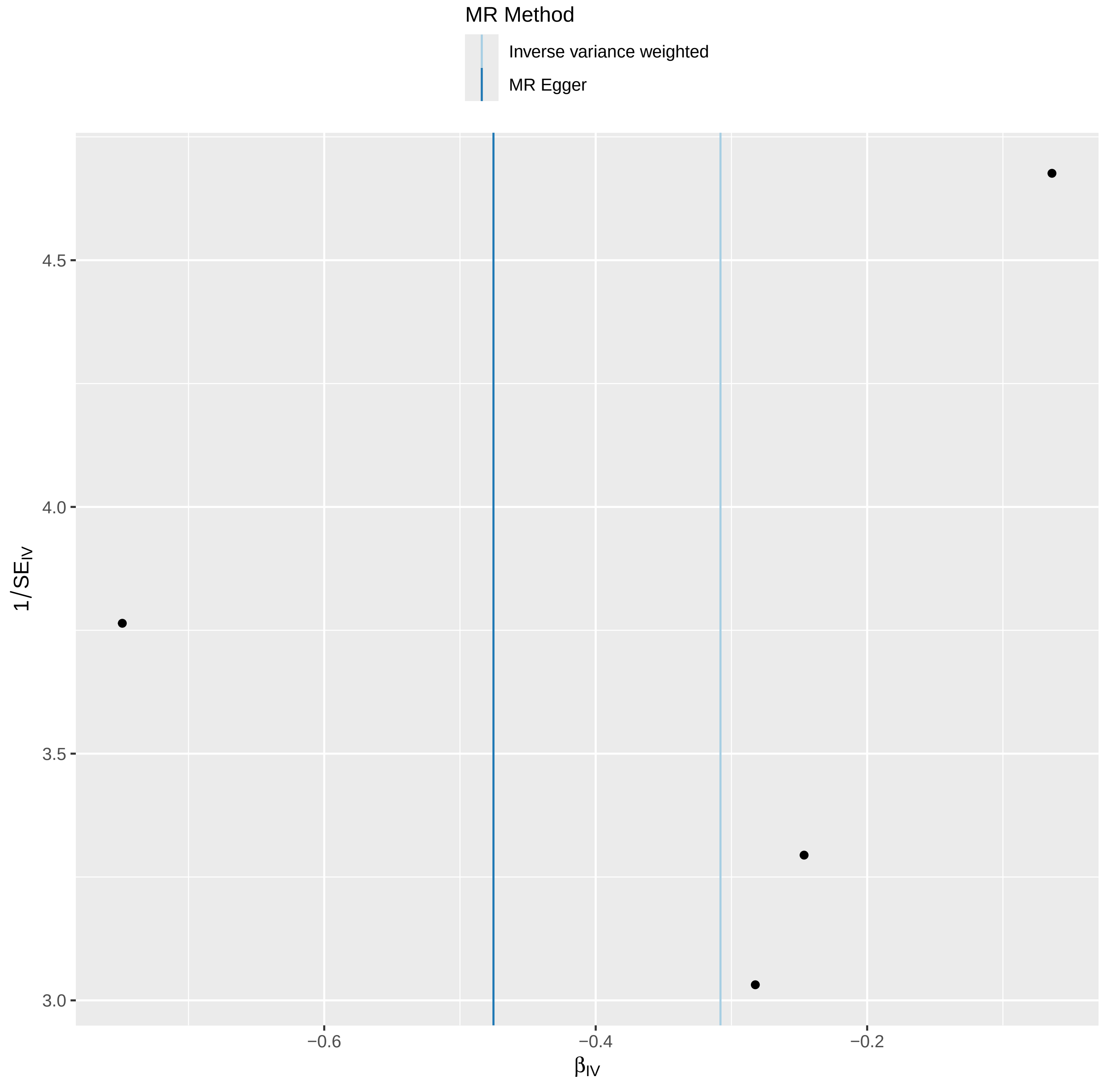
MR Method
Inverse variance weighted
MR Egger
4.5
4.0
3.5
3.0
$1/SE_{IV}$
−0.6
−0.4
−0.2
β_{IV}

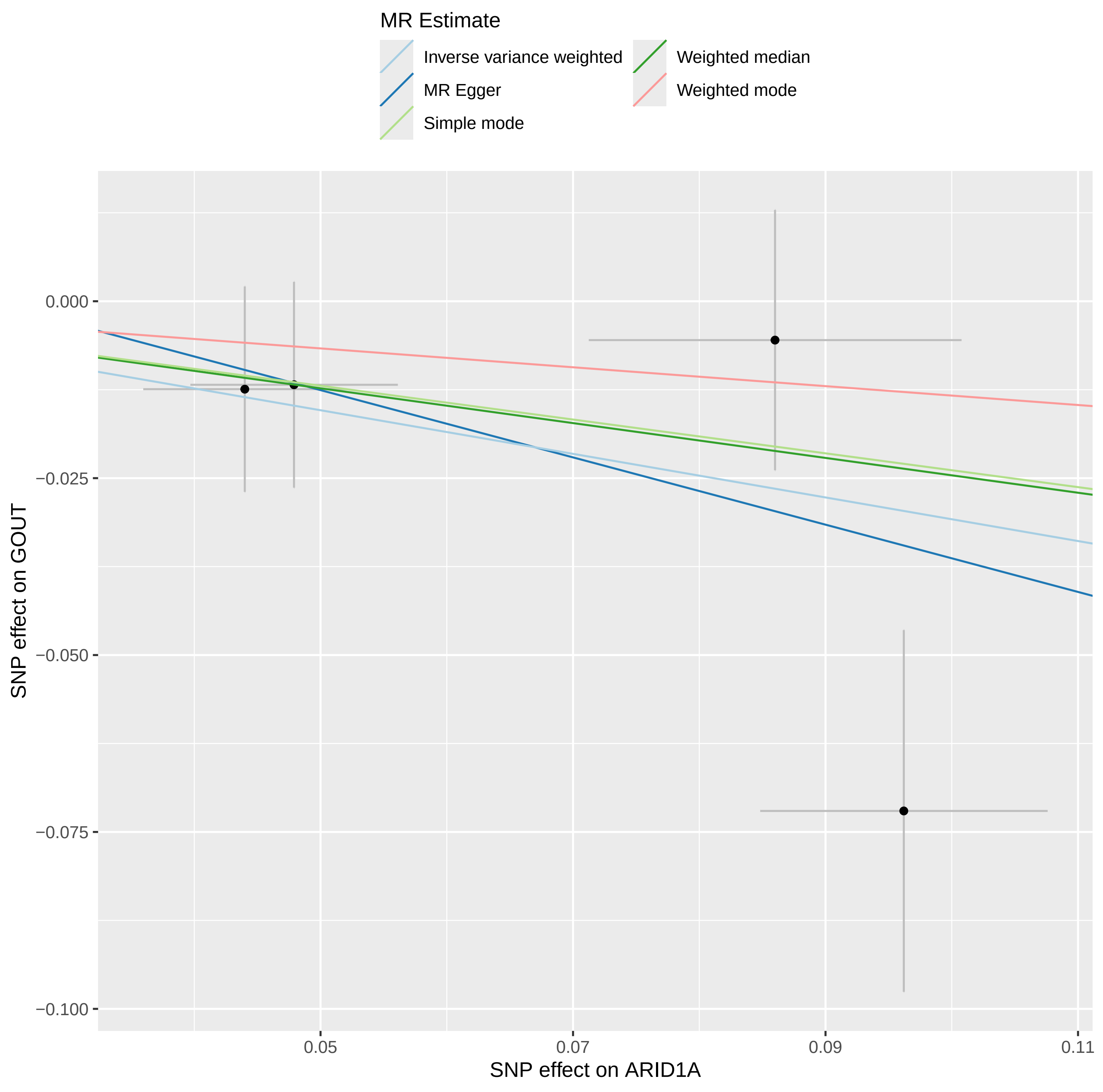

MR Estimate
Inverse variance weighted
MR Egger
Simple mode
Weighted median
Weighted mode
SNP effect on GOUT
0.000
−0.025
−0.050
−0.075
−0.100
SNP effect on ARID1A
0.05
0.07
0.09
0.11

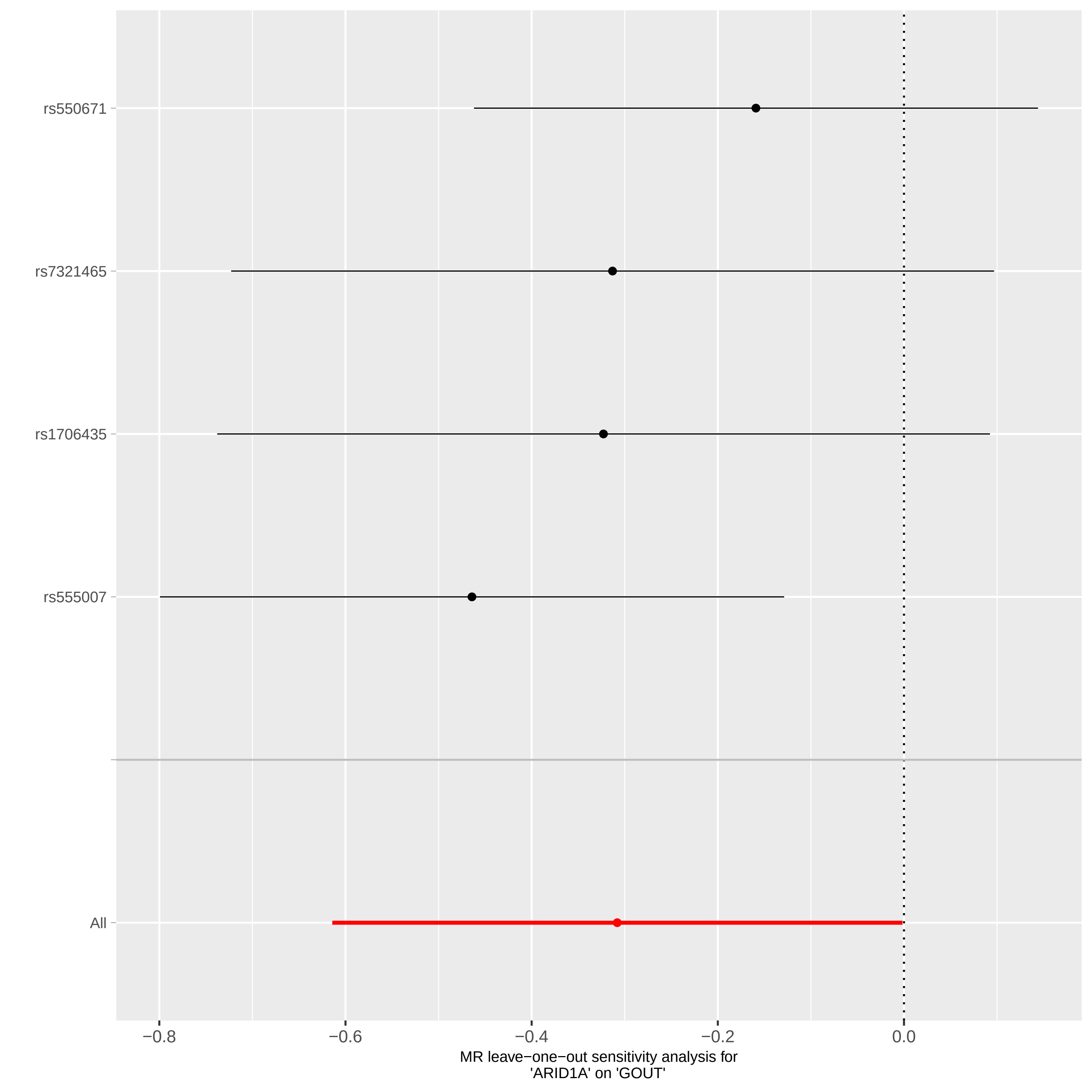
rs550671
rs7321465
rs1706435
rs555007
All
−0.8
−0.6
−0.4
−0.2
0.0
MR leave−one−out sensitivity analysis for
'ARID1A' on 'GOUT'

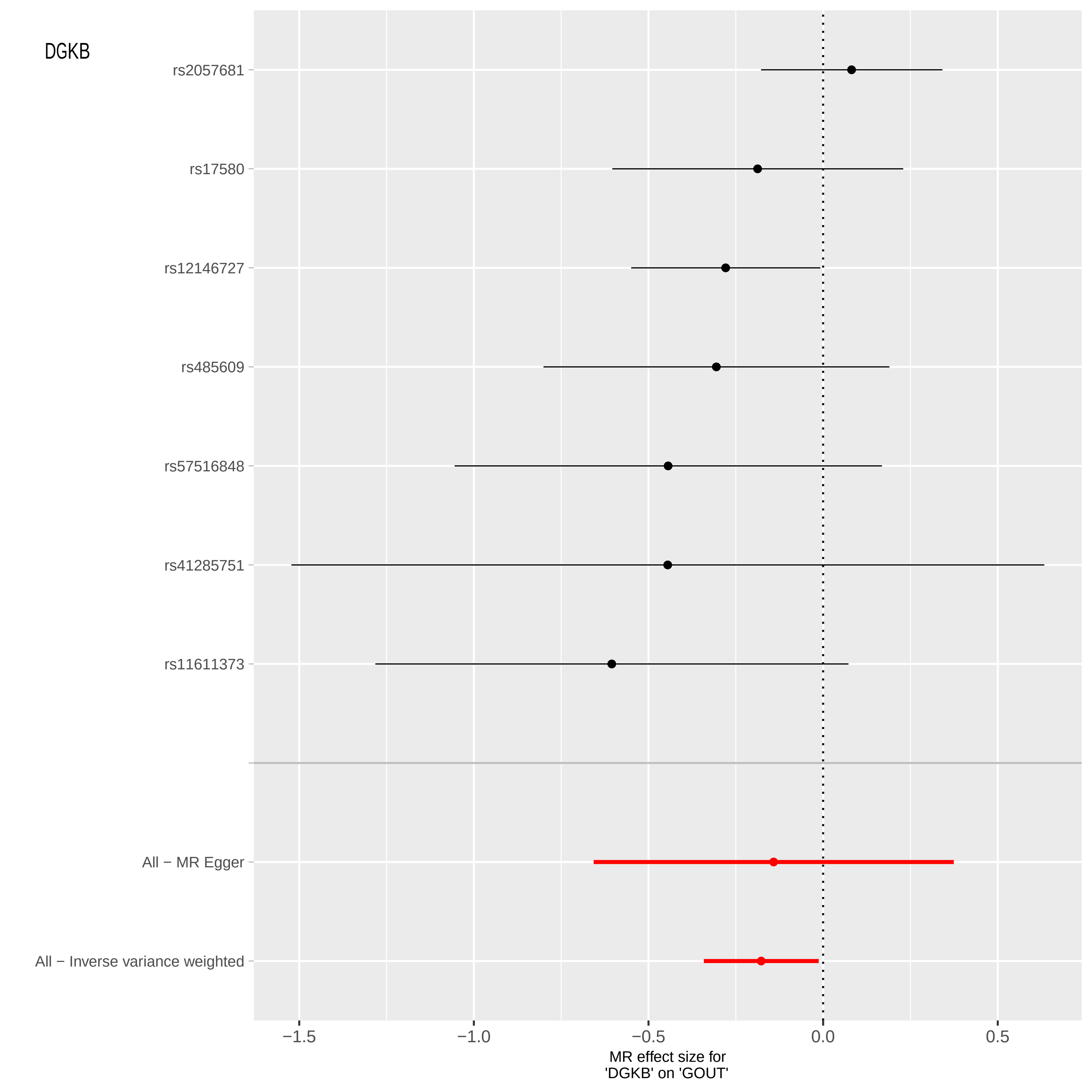
DGKB
rs2057681
rs17580
rs12146727
rs485609
rs57516848
rs41285751
rs11611373
All − MR Egger
All − Inverse variance weighted
−1.5
−1.0
−0.5
0.0
0.5
MR effect size for
'DGKB' on 'GOUT'

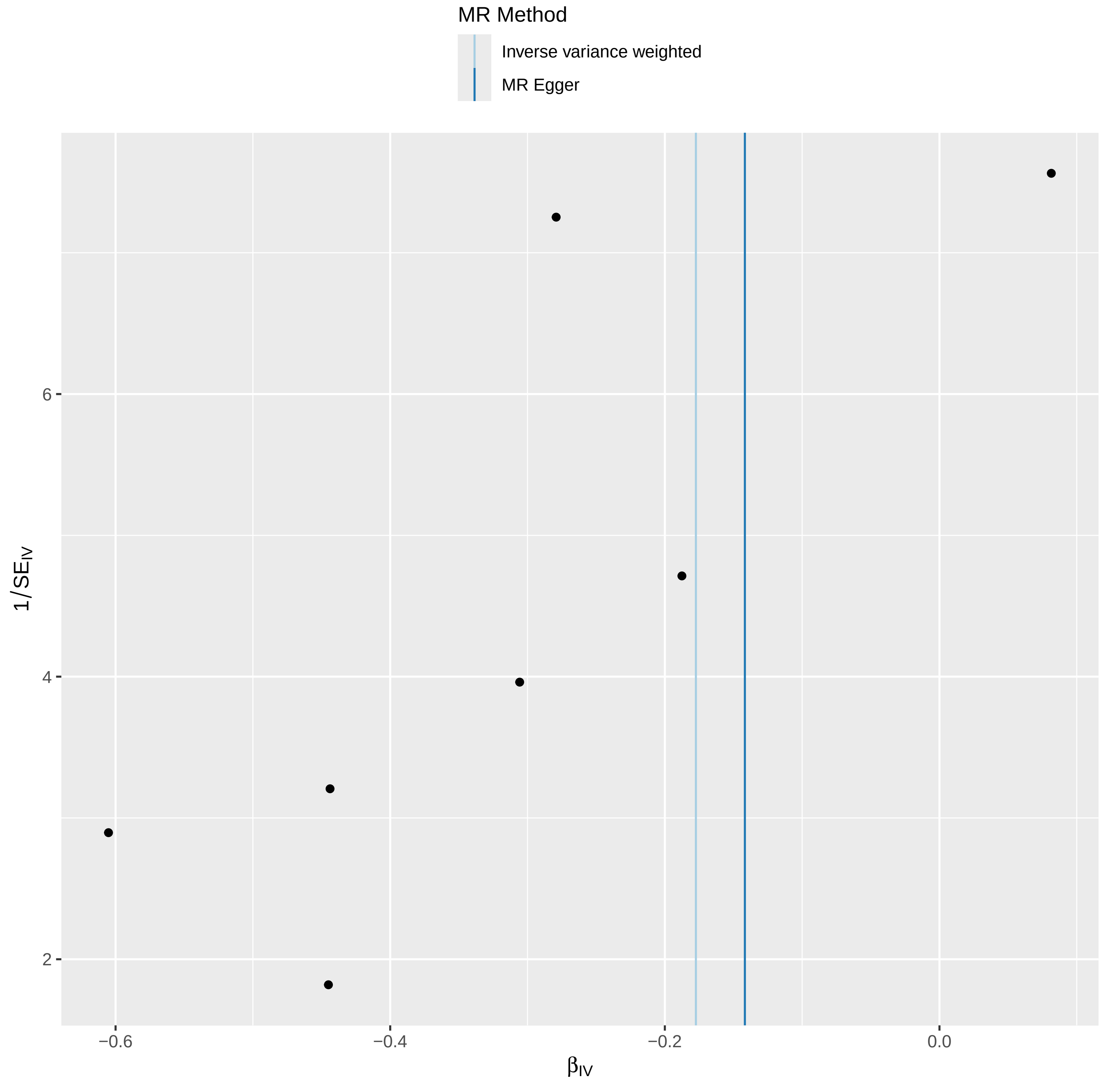

MR Method
Inverse variance weighted
MR Egger
6
4
2
$1/SE_{IV}$
−0.6
−0.4
−0.2
0.0
β_{IV}

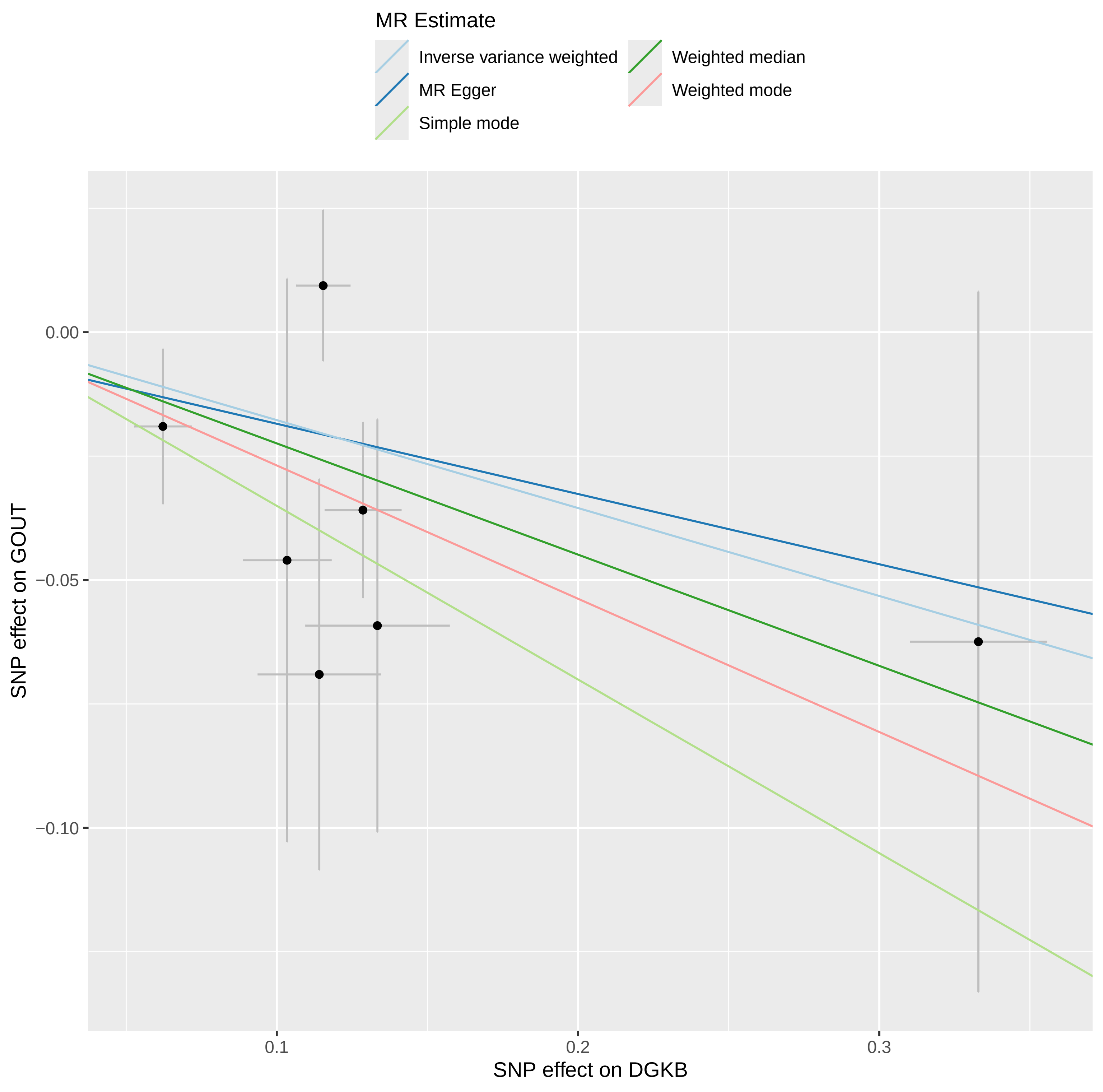

MR Estimate
Inverse variance weighted
MR Egger
Simple mode
Weighted median
Weighted mode
0.00
−0.05
−0.10
SNP effect on GOUT
0.1
0.2
0.3
SNP effect on DGKB

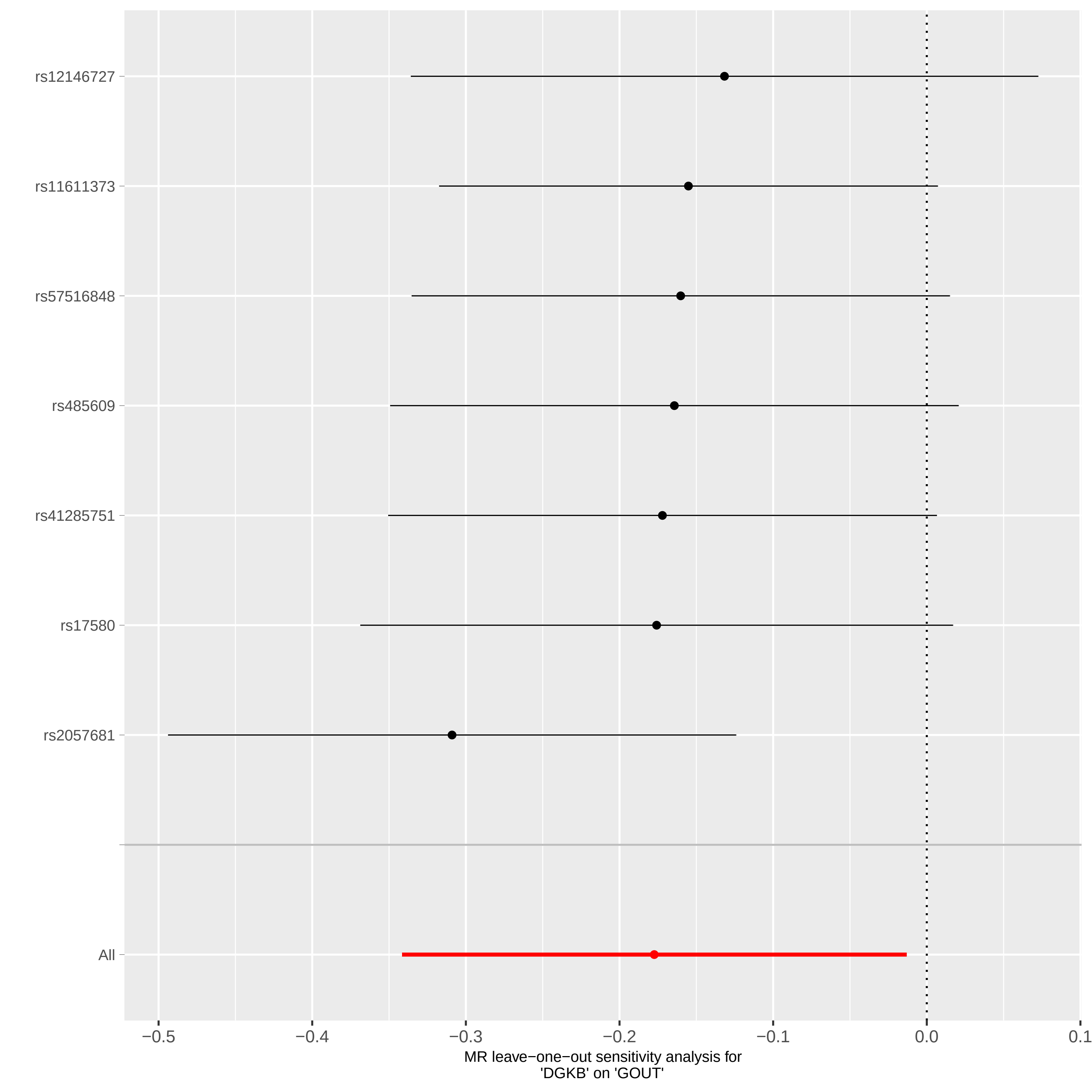
rs12146727
rs11611373
rs57516848
rs485609
rs41285751
rs17580
rs2057681
All
−0.5
−0.4
−0.3
−0.2
−0.1
0.0
0.1
MR leave−one−out sensitivity analysis for
'DGKB' on 'GOUT'

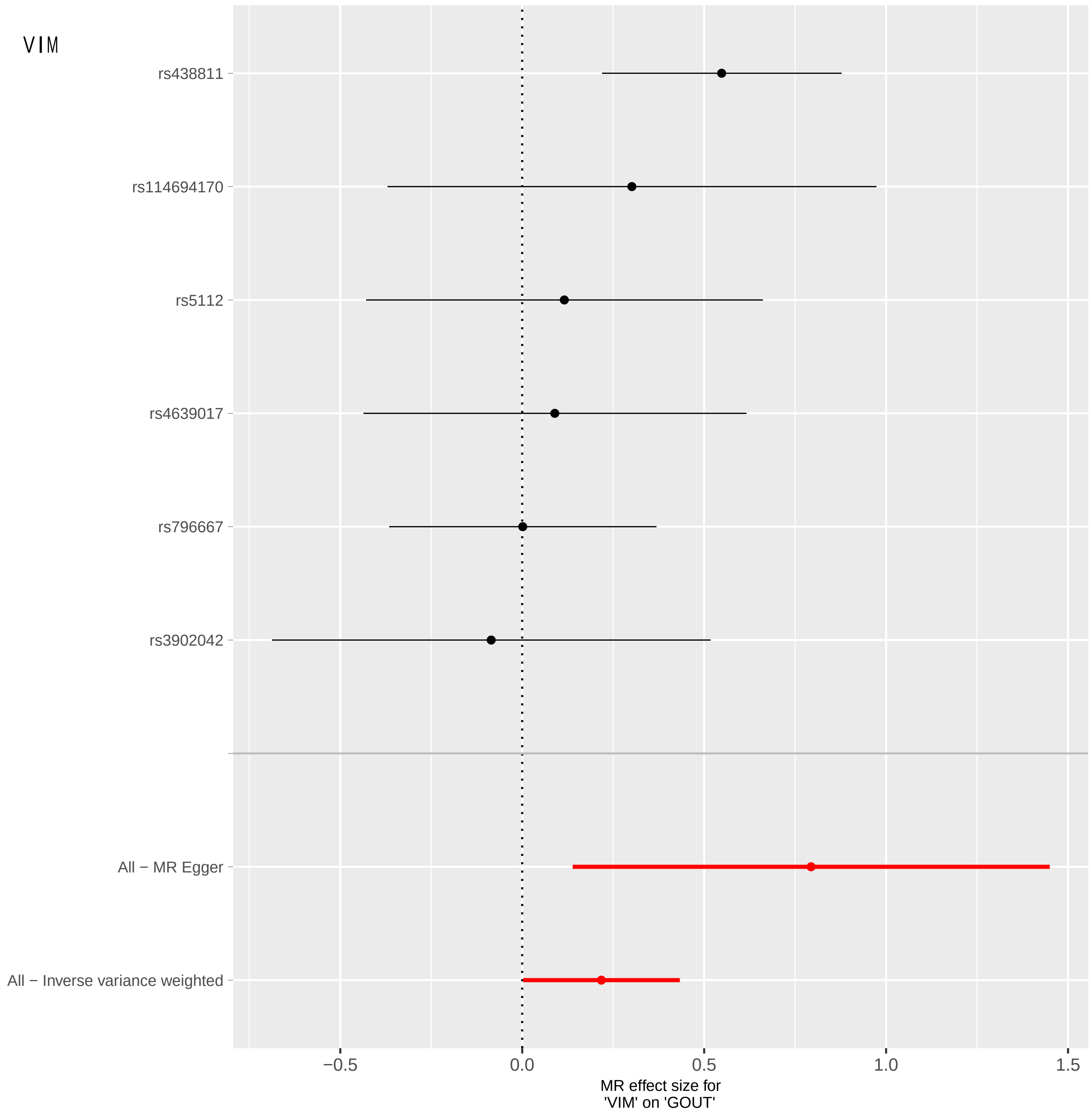
VIM
rs438811
rs114694170
rs5112
rs4639017
rs796667
rs3902042
All − MR Egger
All − Inverse variance weighted
−0.5
0.0
0.5
1.0
1.5
MR effect size for
'VIM' on 'GOUT'

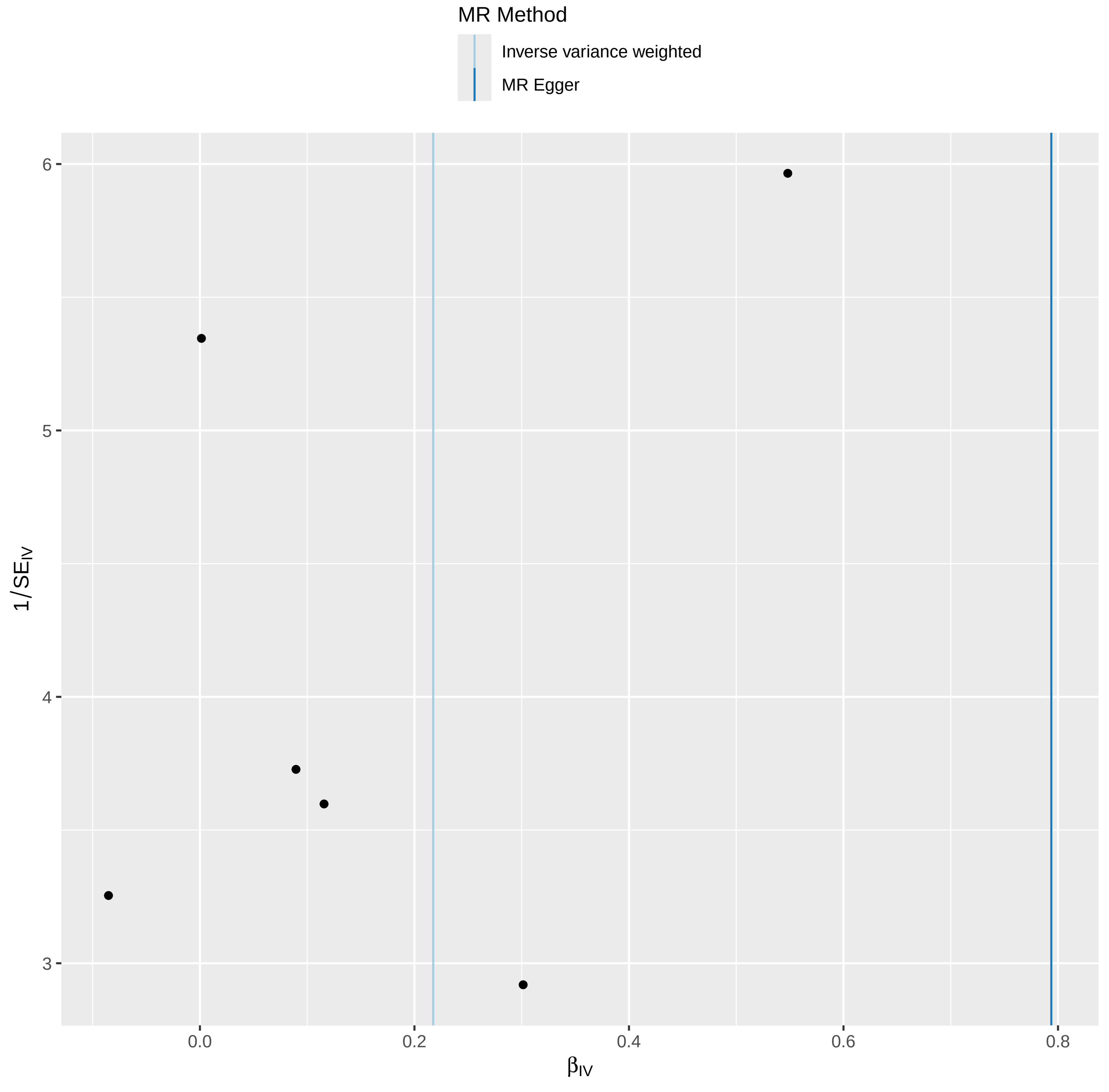

MR Method
Inverse variance weighted
MR Egger
6
5
4
3
1/SE_IV
0.0
0.2
0.4
0.6
0.8
β_IV

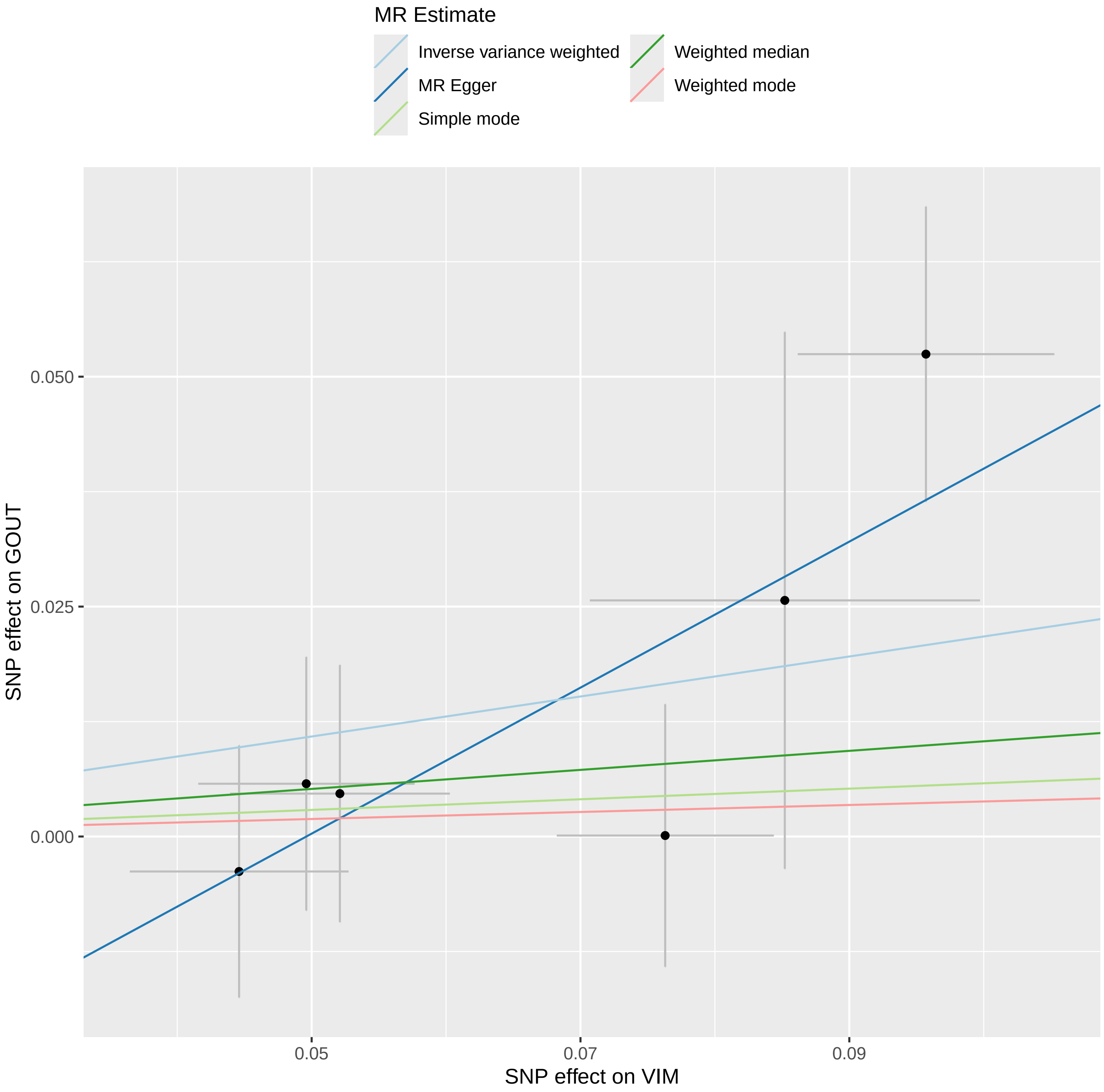
MR Estimate
Inverse variance weighted
MR Egger
Simple mode
Weighted median
Weighted mode
0.050
0.025
0.000
SNP effect on GOUT
0.05
0.07
0.09
SNP effect on VIM

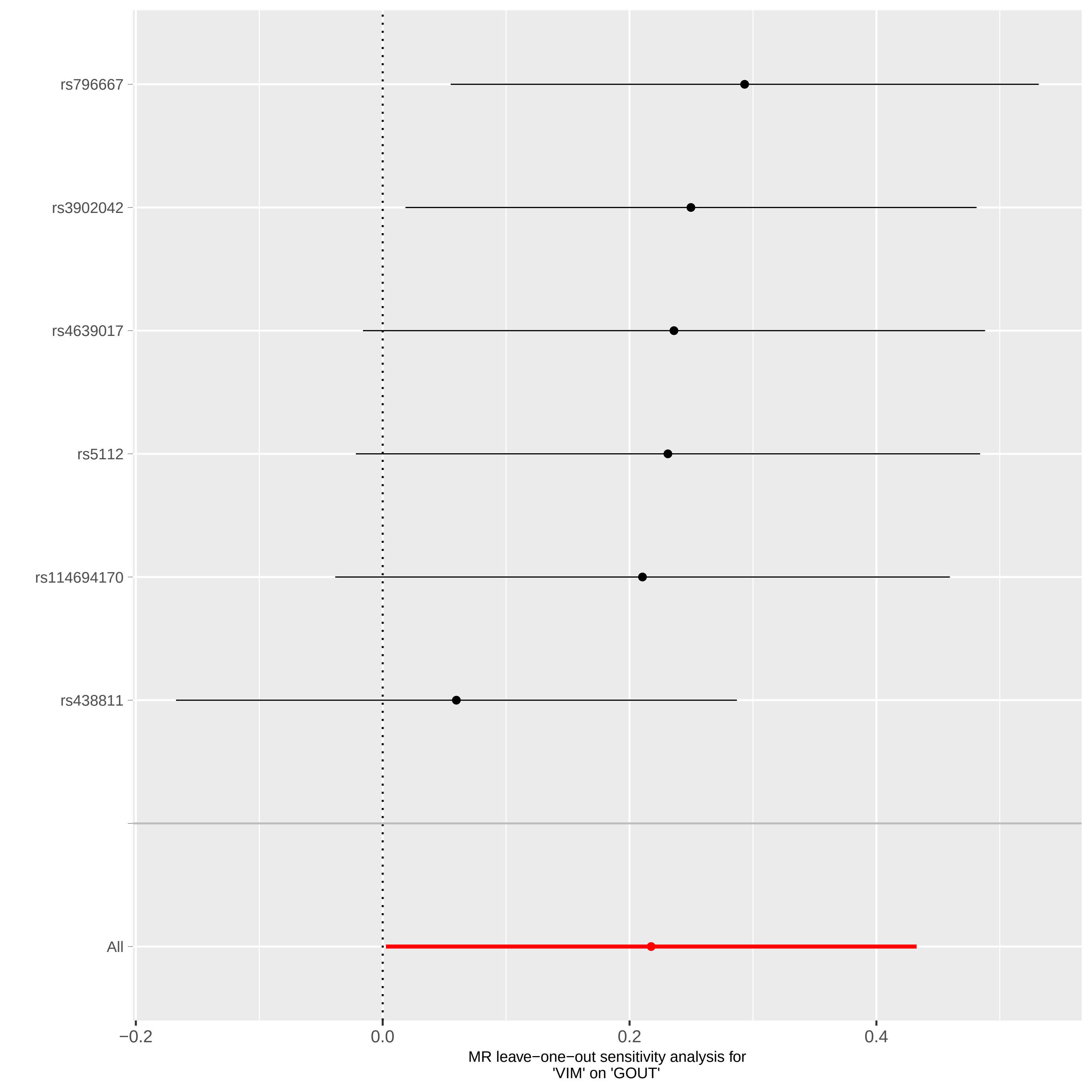

rs796667
rs3902042
rs4639017
rs5112
rs114694170
rs438811
All
−0.2
0.0
0.2
0.4
MR leave−one−out sensitivity analysis for
'VIM' on 'GOUT'
