# Supplementary material for: Decoding the lipid etiology of atherogenic index of plasma and gout: establishing the causal role of triglycerides through NHANES, Mendelian randomization, and network pharmacology
Source: Cardiovasc Diabetol Endocrinol Rep. 2026 Jul 13;12:40. doi: 10.1186/s40842-026-00309-0 (PMC13362044; doi:10.1186/s40842-026-00309-0)
Supplement: Supplementary file 8 — Supplementary Material 8 [file 40842_2026_309_MOESM8_ESM.pdf]

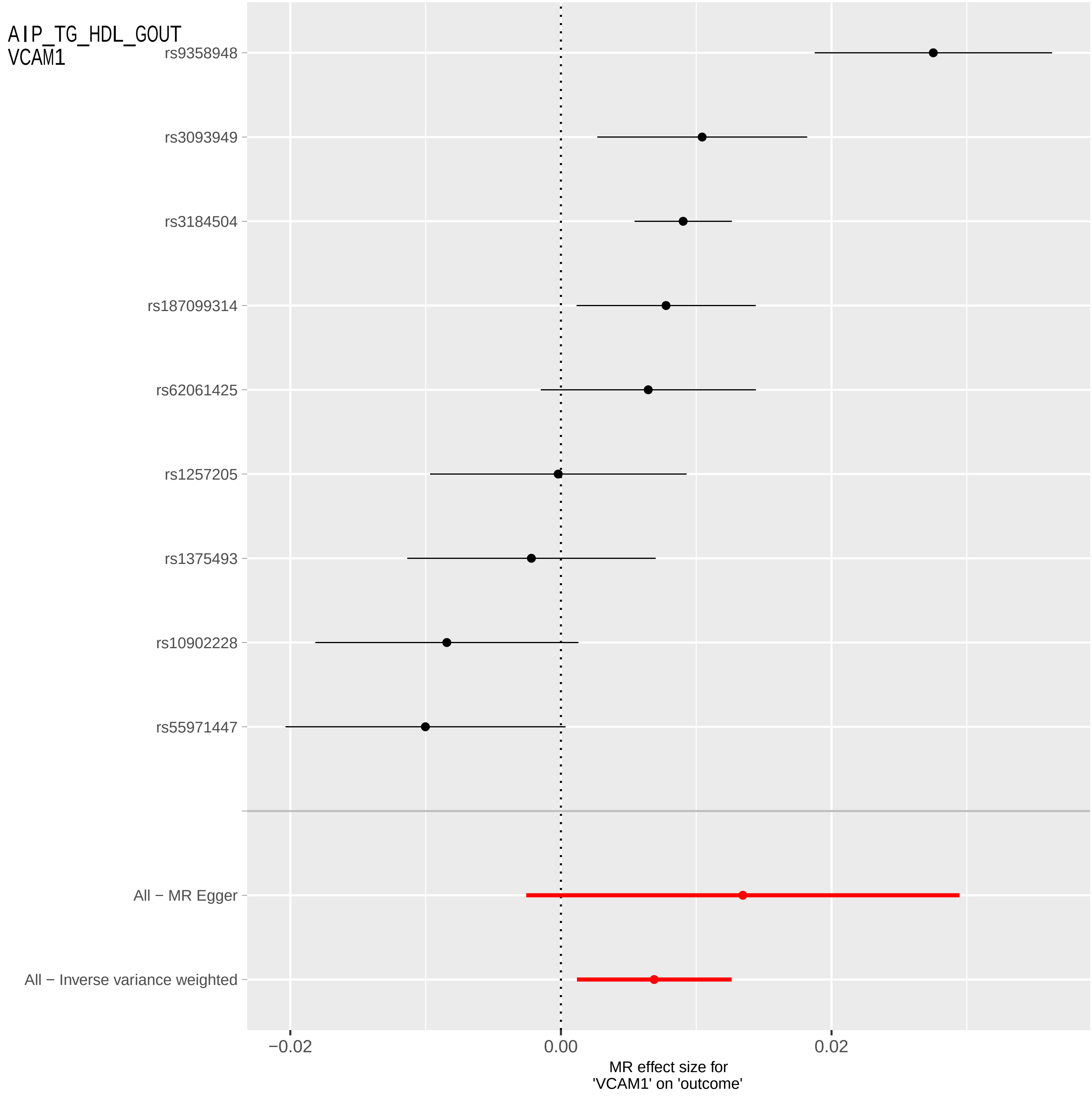
AIP_TG_HDL_GOUT
VCAM1
rs9358948
rs3093949
rs3184504
rs187099314
rs62061425
rs1257205
rs1375493
rs10902228
rs55971447
All − MR Egger
All − Inverse variance weighted
−0.02
0.00
0.02
MR effect size for
'VCAM1' on 'outcome'

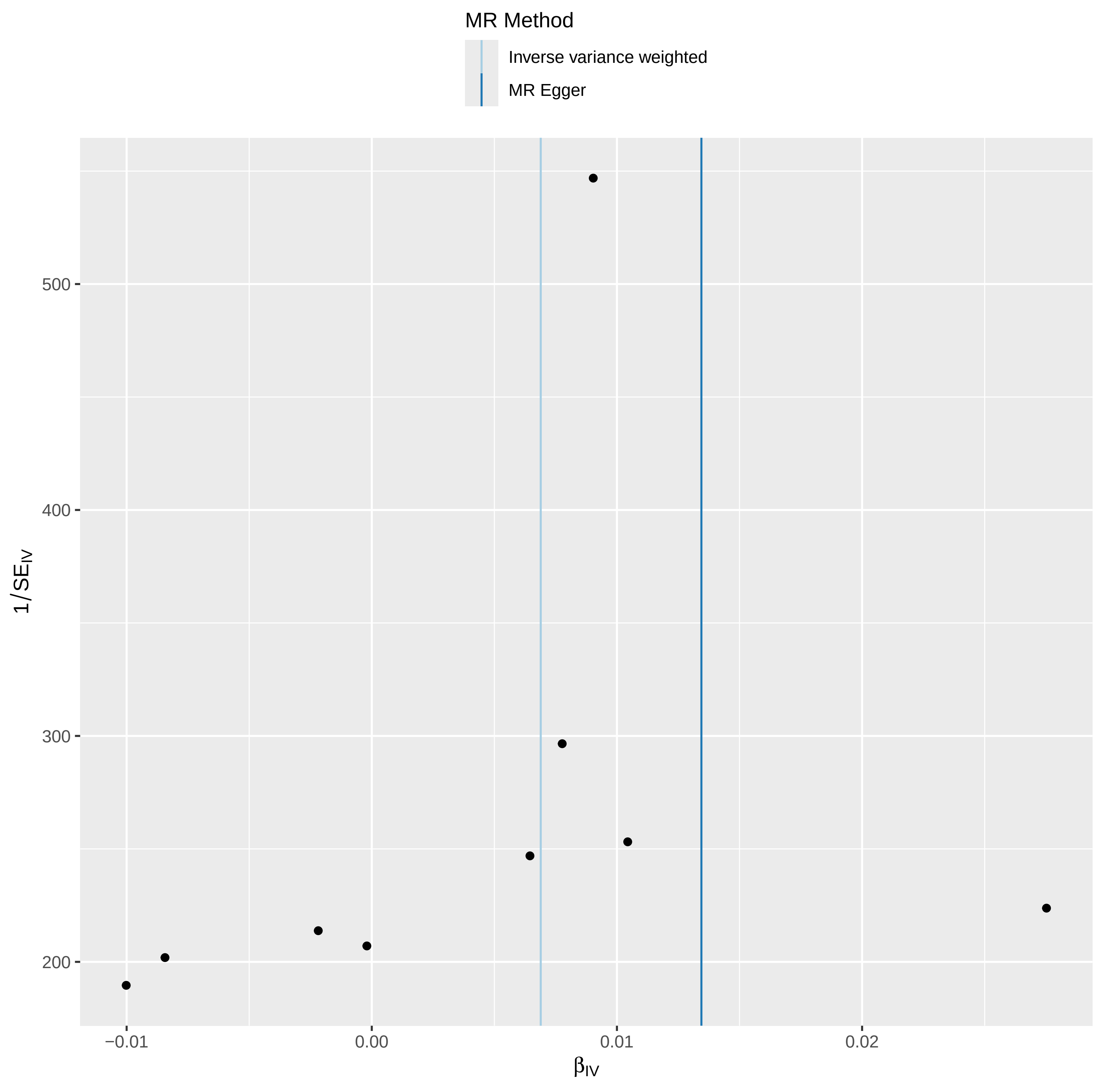

MR Method
Inverse variance weighted
MR Egger
500
400
300
200
$1/SE_{IV}$
−0.01
0.00
0.01
0.02
β_{IV}

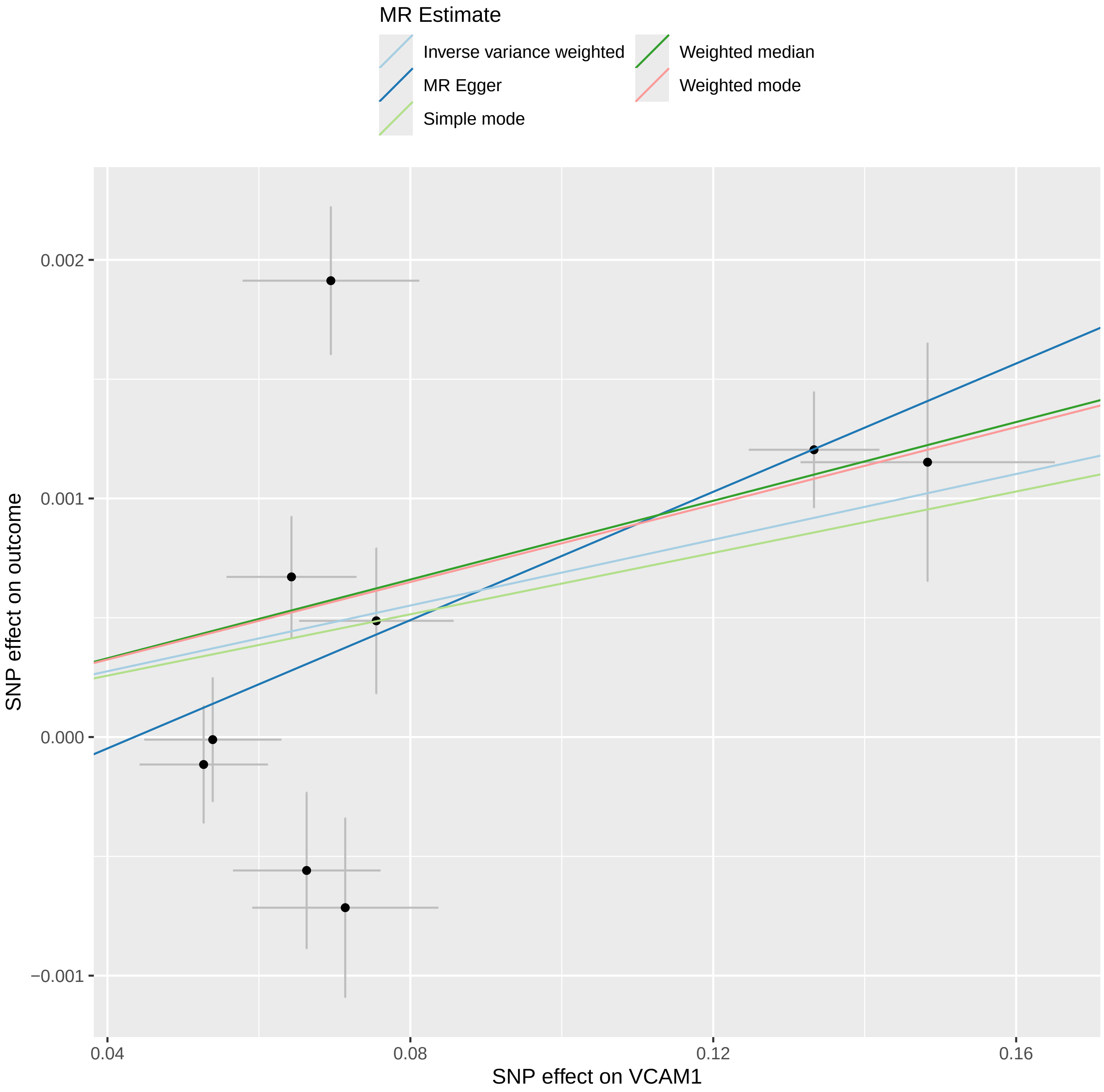

MR Estimate
Inverse variance weighted
MR Egger
Simple mode
Weighted median
Weighted mode
0.002
0.001
0.000
−0.001
SNP effect on outcome
0.04
0.08
0.12
0.16
SNP effect on VCAM1

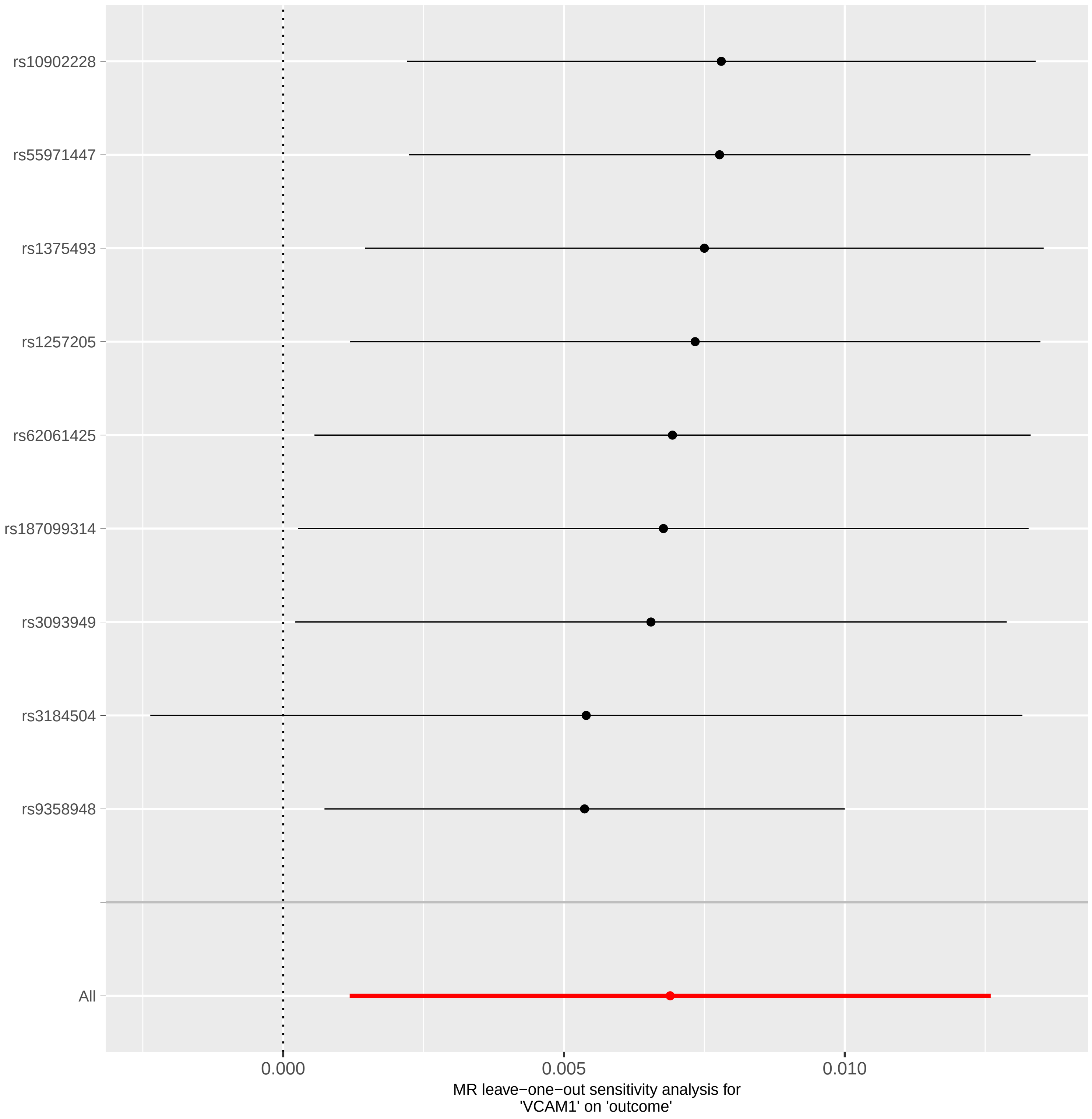
rs10902228
rs55971447
rs1375493
rs1257205
rs62061425
rs187099314
rs3093949
rs3184504
rs9358948
All
0.000
0.005
0.010
MR leave−one−out sensitivity analysis for
'VCAM1' on 'outcome'

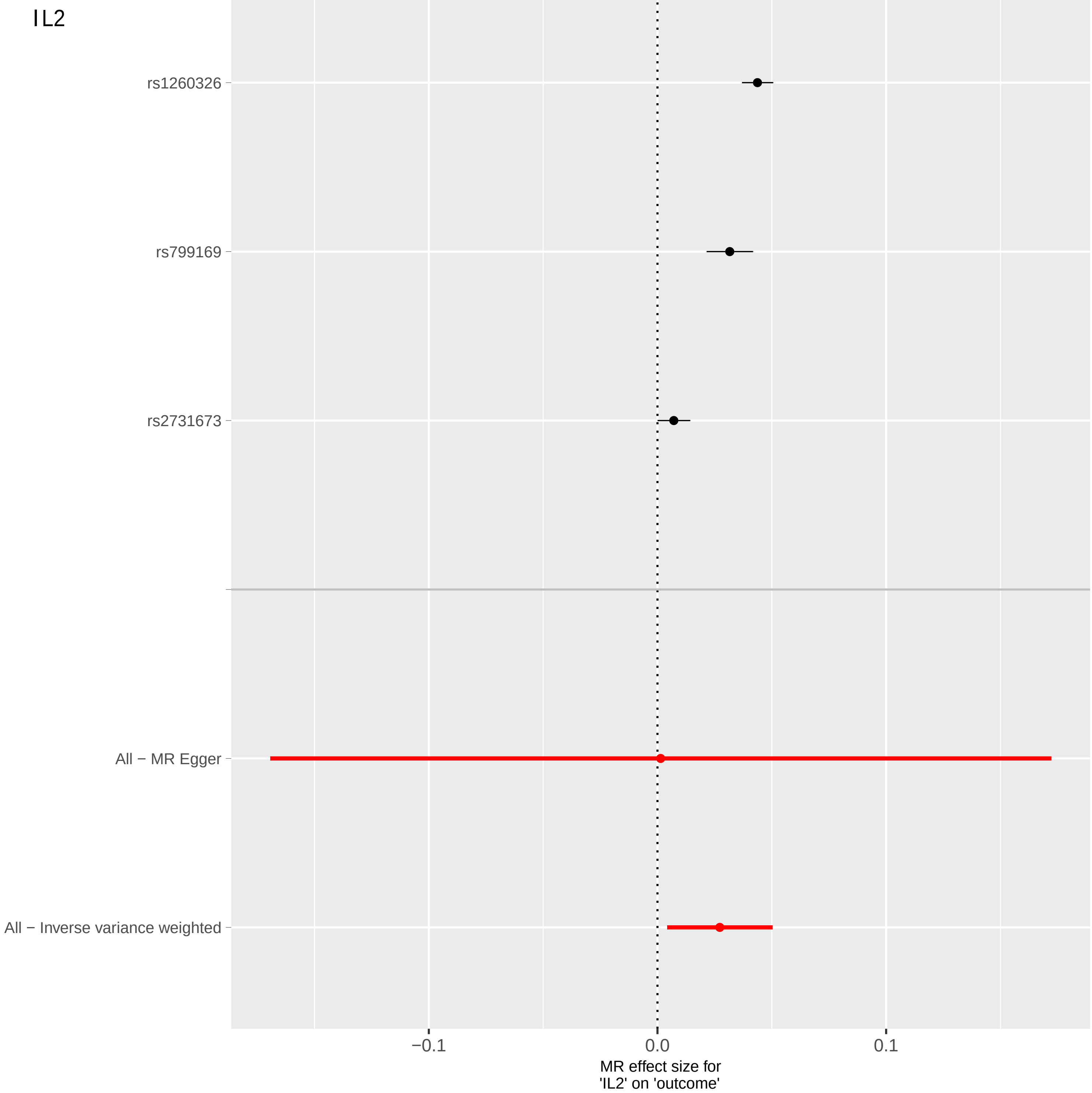

IL2
rs1260326
rs799169
rs2731673
All − MR Egger
All − Inverse variance weighted
−0.1
0.0
0.1
MR effect size for
'IL2' on 'outcome'

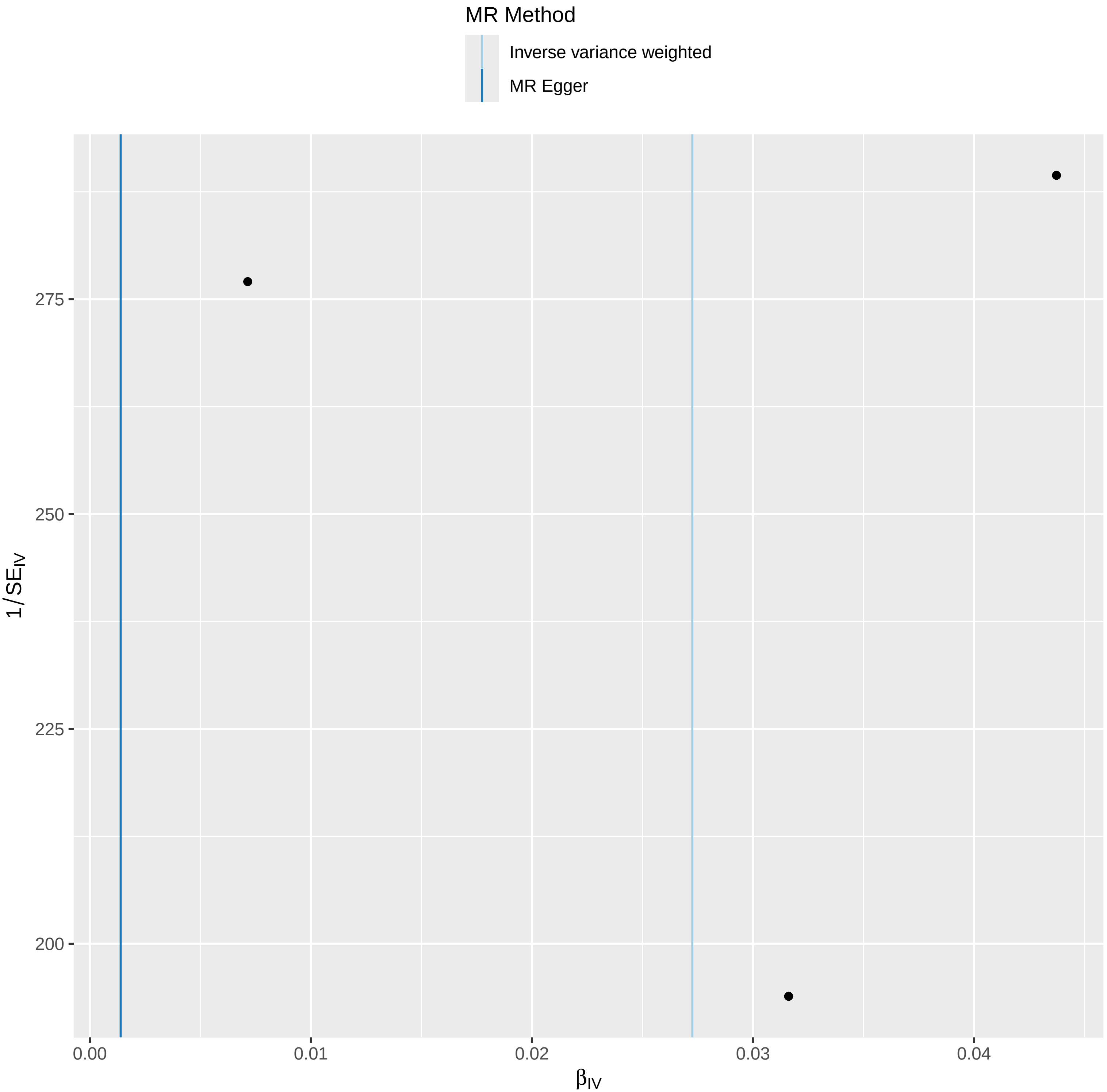
MR Method
Inverse variance weighted
MR Egger
275
250
225
200
1/SE_IV
0.00
0.01
0.02
0.03
0.04
β_IV

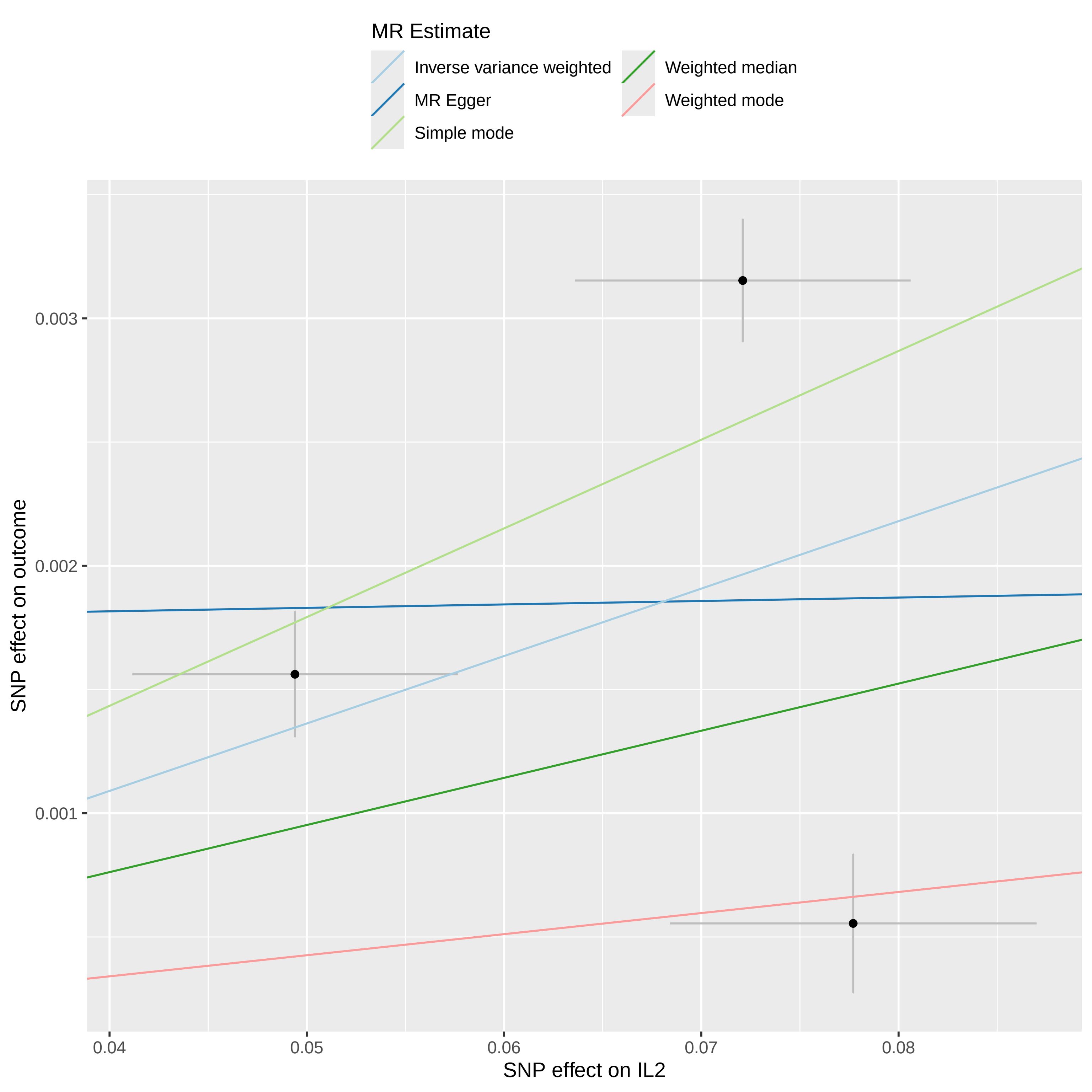
MR Estimate
Inverse variance weighted
MR Egger
Simple mode
Weighted median
Weighted mode
0.003
0.002
0.001
SNP effect on outcome
0.04
0.05
0.06
0.07
0.08
SNP effect on IL2

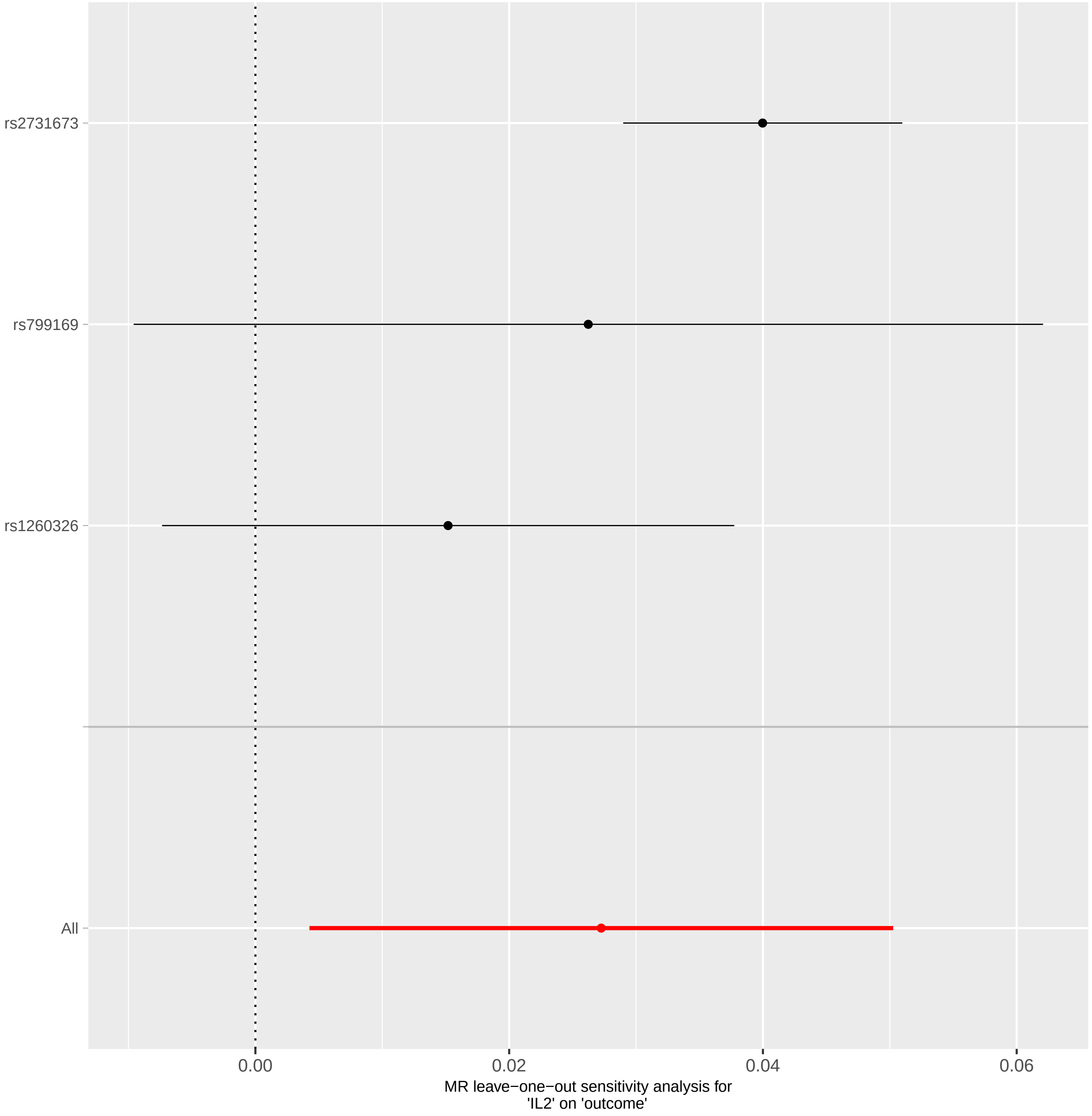

rs2731673
rs799169
rs1260326
All
0.00
0.02
0.04
0.06
MR leave−one−out sensitivity analysis for
'IL2' on 'outcome'

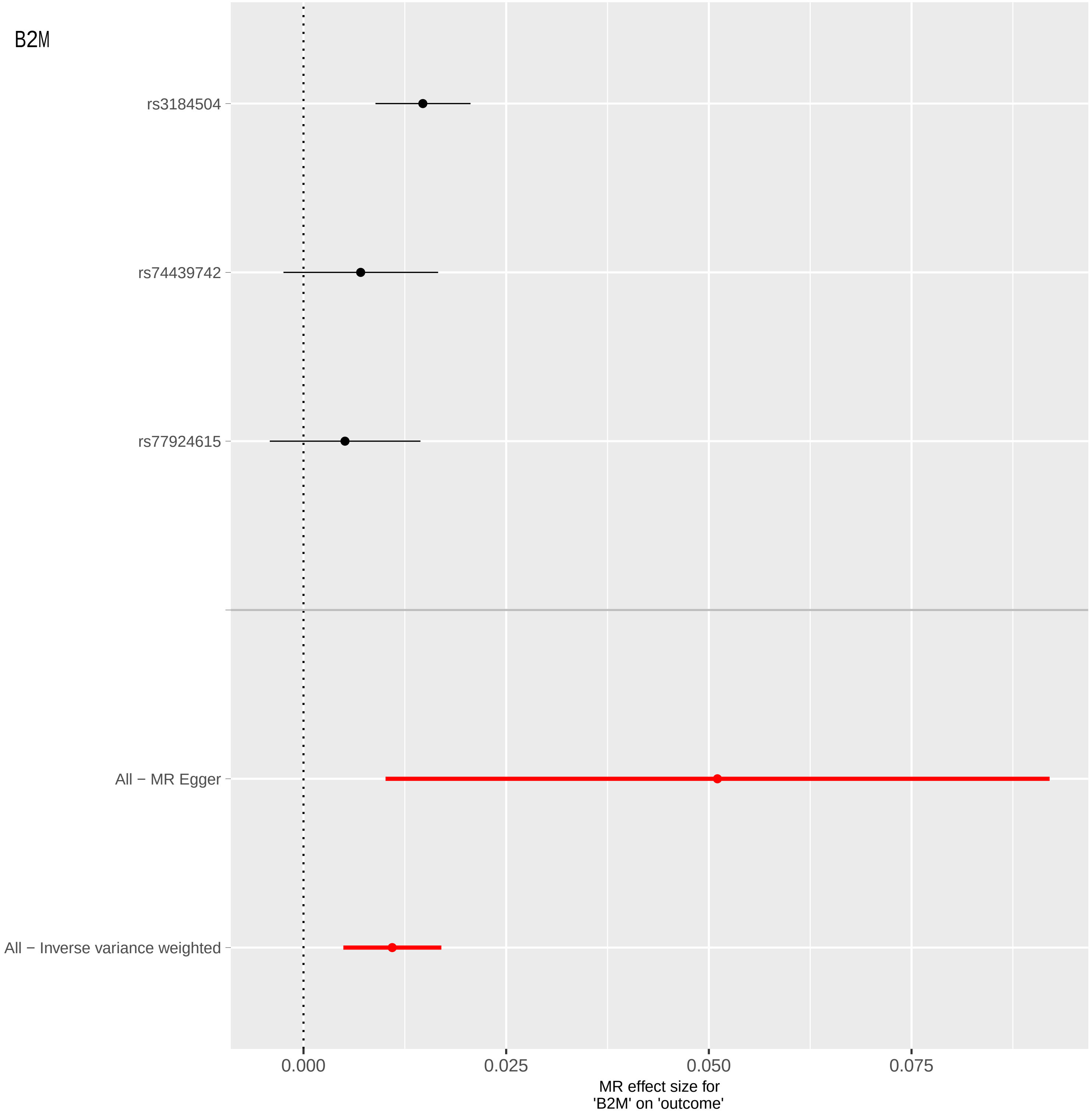
B2M
rs3184504
rs74439742
rs77924615
All − MR Egger
All − Inverse variance weighted
0.000
0.025
0.050
0.075
MR effect size for
'B2M' on 'outcome'

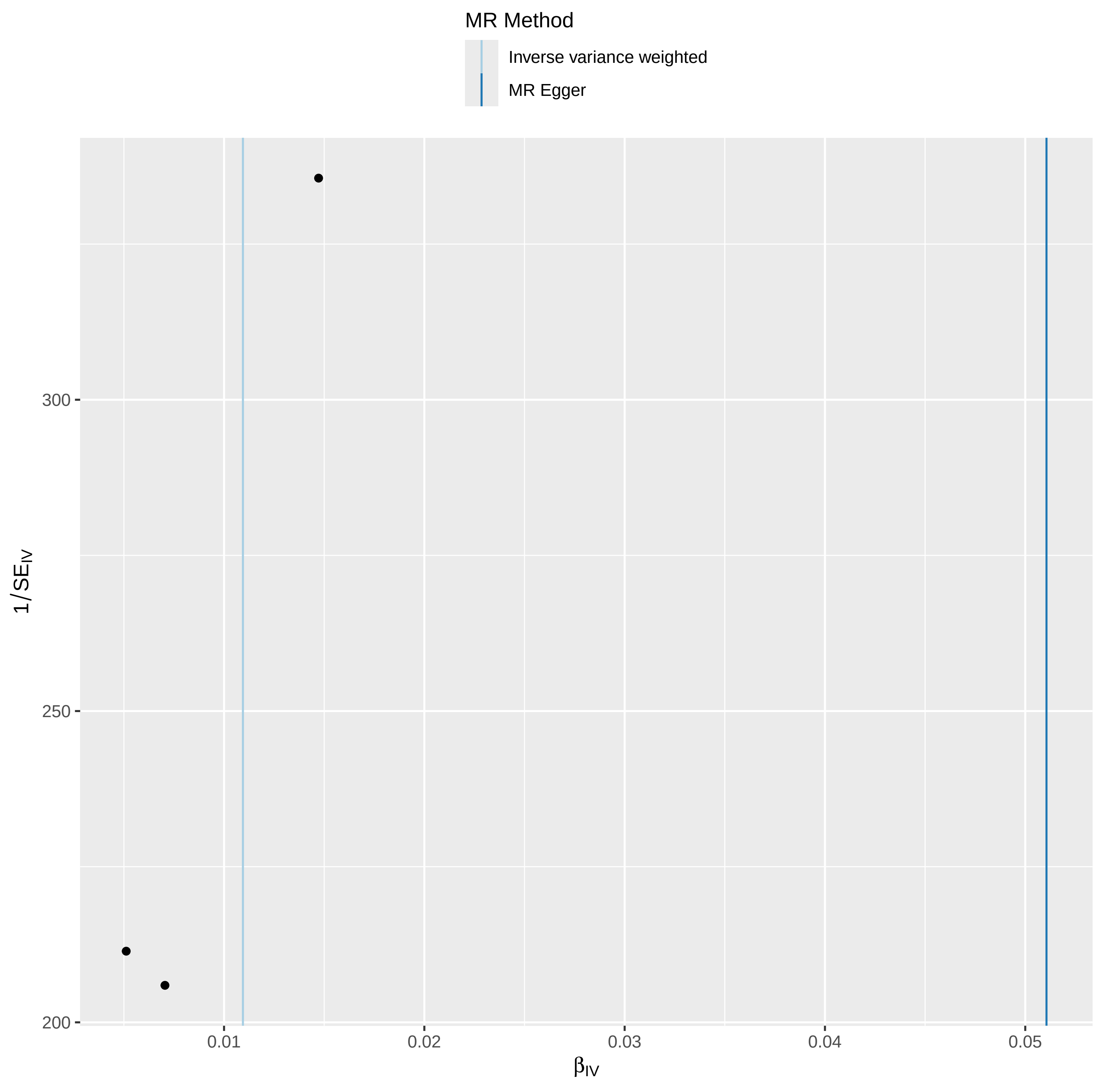

MR Method
Inverse variance weighted
MR Egger
300
250
200
1/SE_IV
0.01
0.02
0.03
0.04
0.05
β_IV

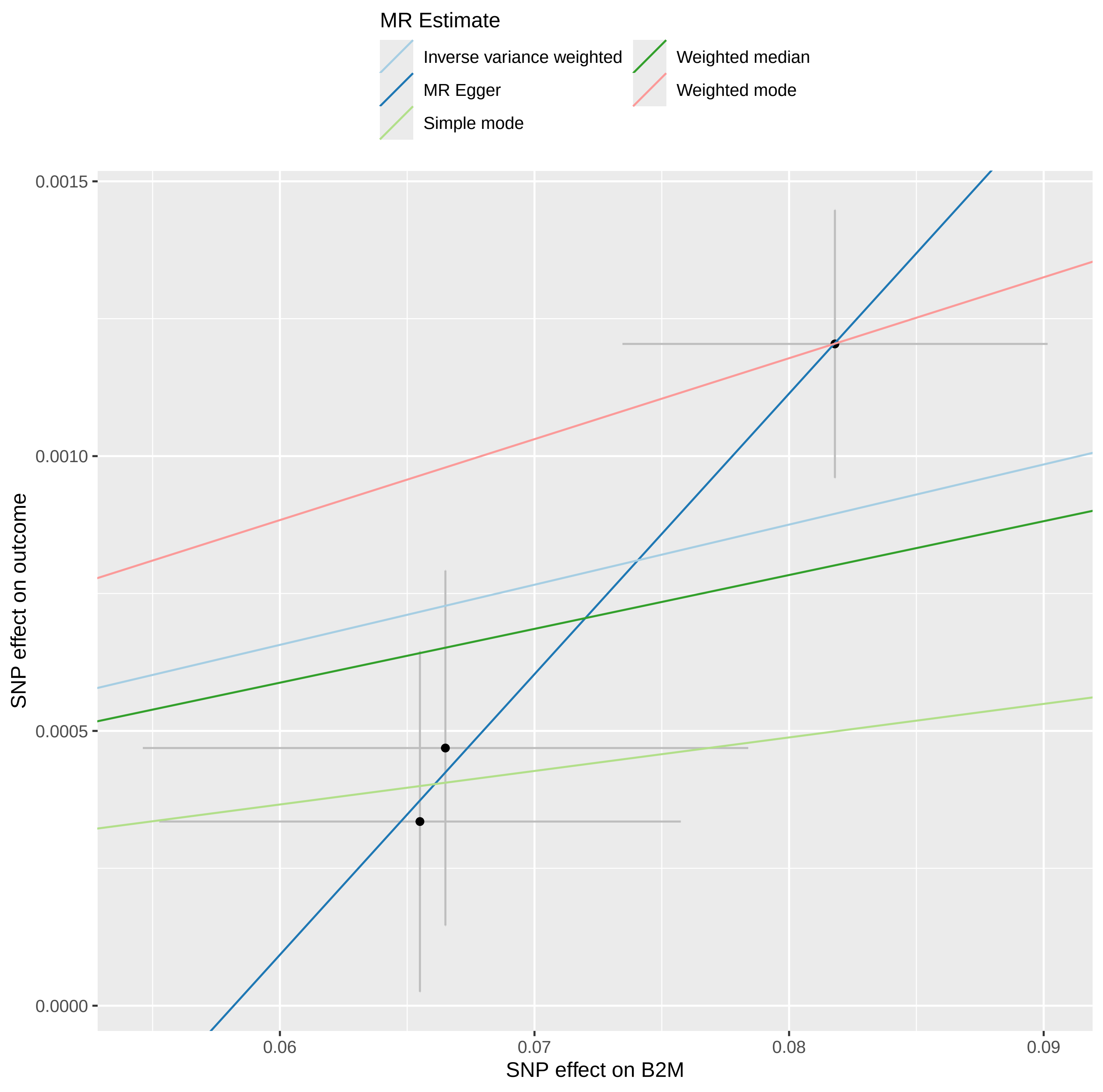
MR Estimate
Inverse variance weighted
MR Egger
Simple mode
Weighted median
Weighted mode
0.0015
0.0010
0.0005
0.0000
SNP effect on outcome
0.06
0.07
0.08
0.09
SNP effect on B2M

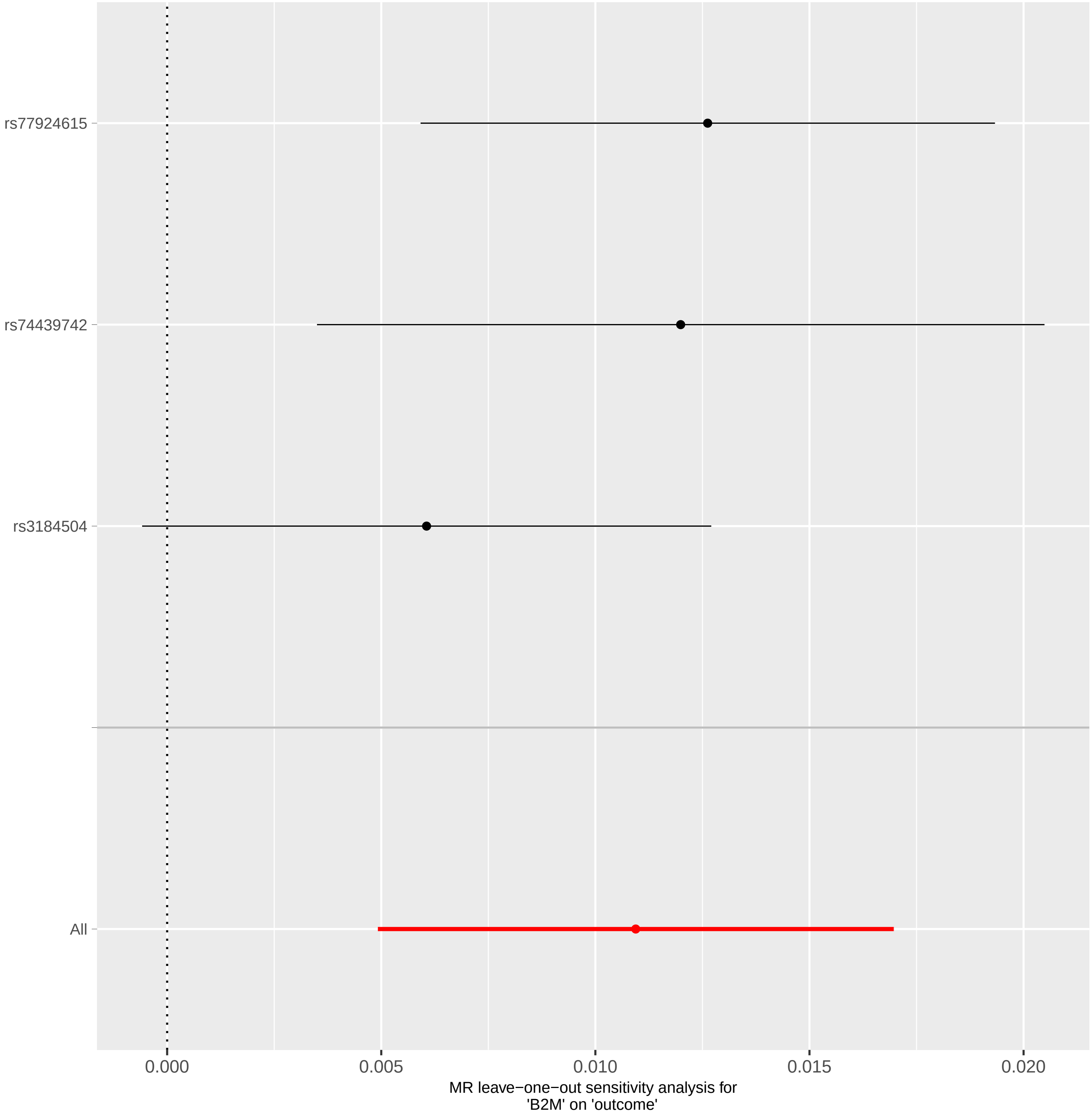

rs77924615
rs74439742
rs3184504
All
0.000
0.005
0.010
0.015
0.020
MR leave−one−out sensitivity analysis for
'B2M' on 'outcome'

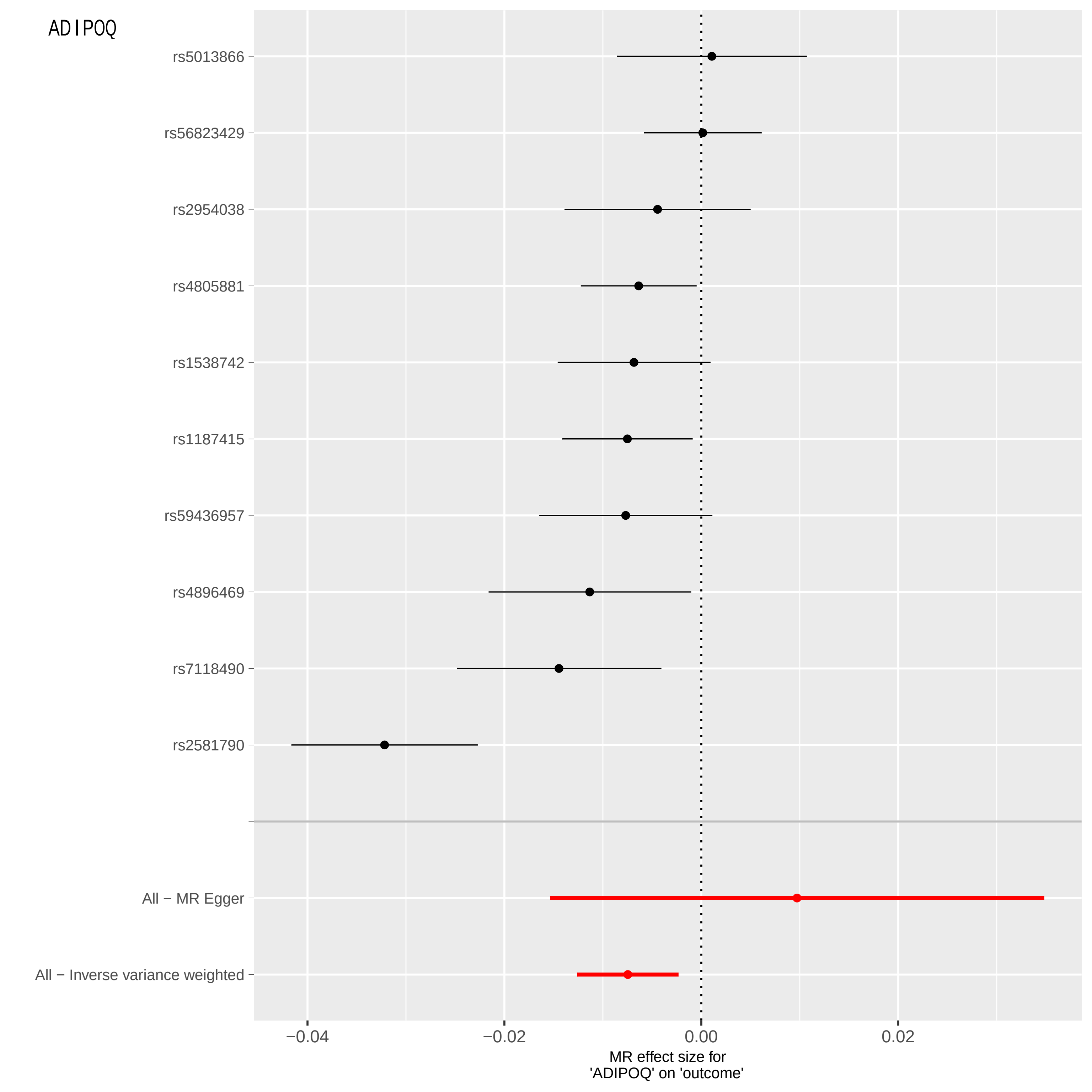

ADIPOQ
rs5013866
rs56823429
rs2954038
rs4805881
rs1538742
rs1187415
rs59436957
rs4896469
rs7118490
rs2581790
All − MR Egger
All − Inverse variance weighted
−0.04
−0.02
0.00
0.02
MR effect size for
'ADIPOQ' on 'outcome'

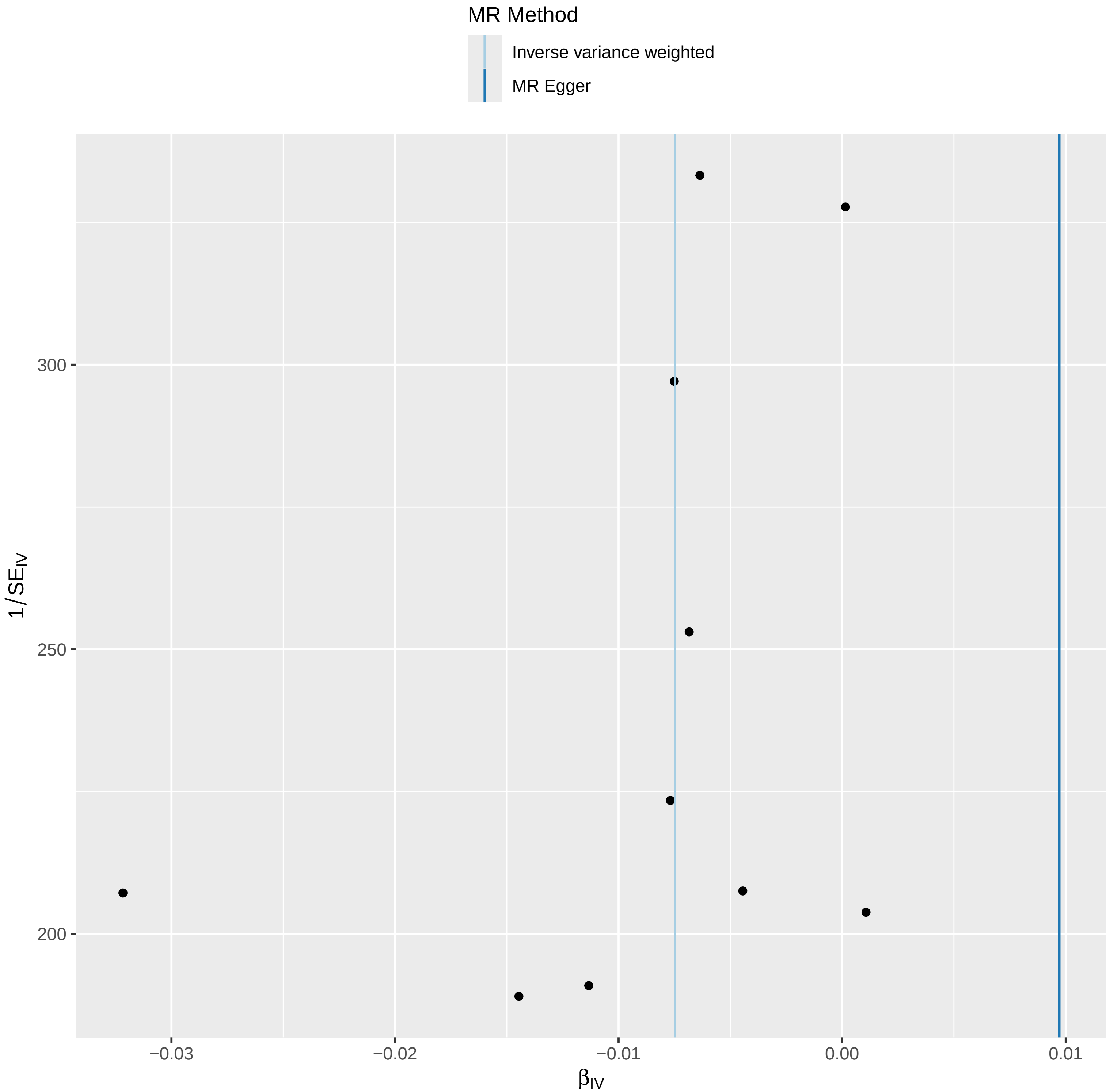

MR Method
Inverse variance weighted
MR Egger
300
250
200
1/SE_IV
−0.03
−0.02
−0.01
0.00
0.01
β_IV

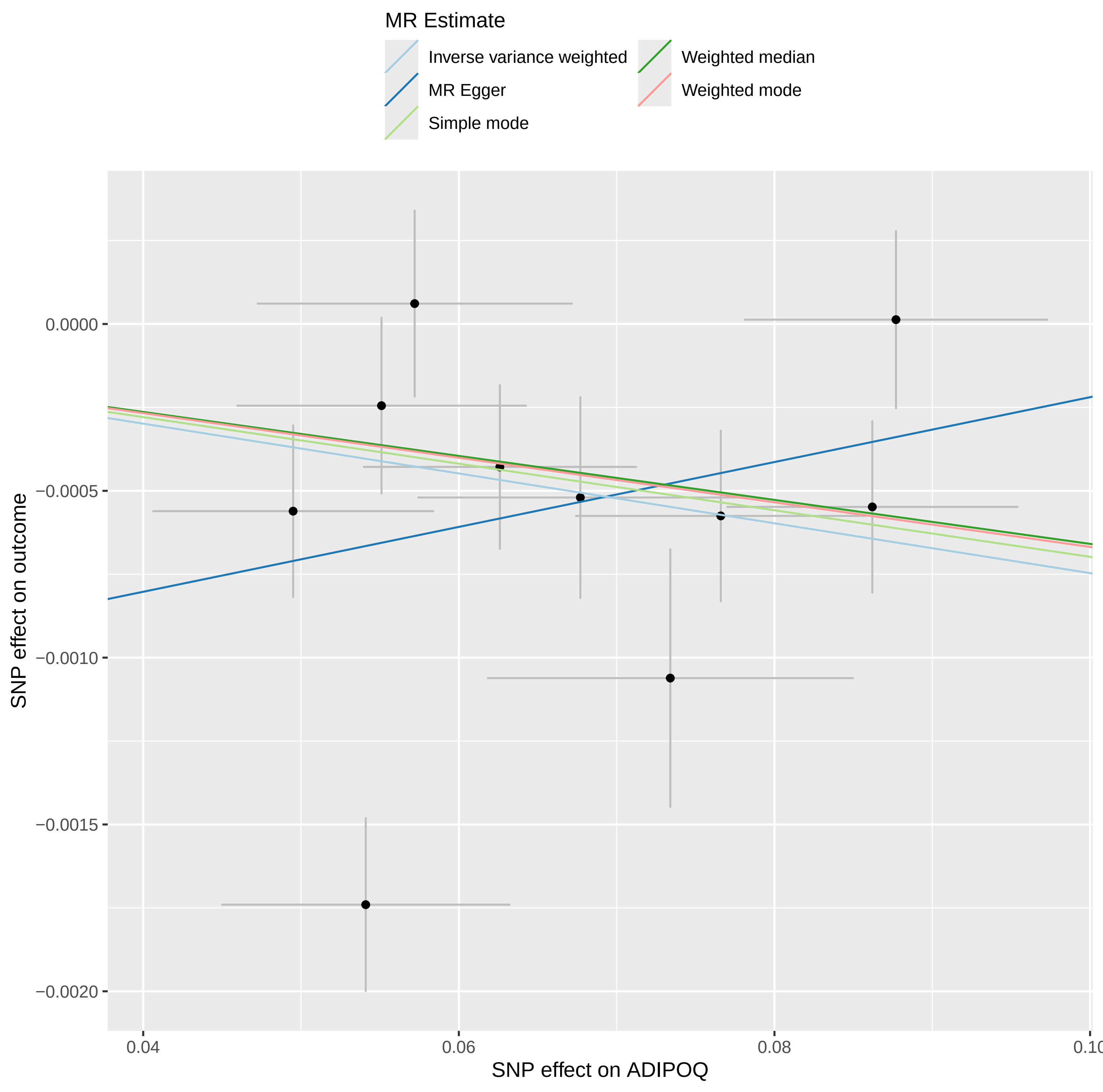

MR Estimate
Inverse variance weighted
MR Egger
Simple mode
Weighted median
Weighted mode
SNP effect on outcome
0.0000
−0.0005
−0.0010
−0.0015
−0.0020
0.04
0.06
0.08
0.10
SNP effect on ADIPOQ

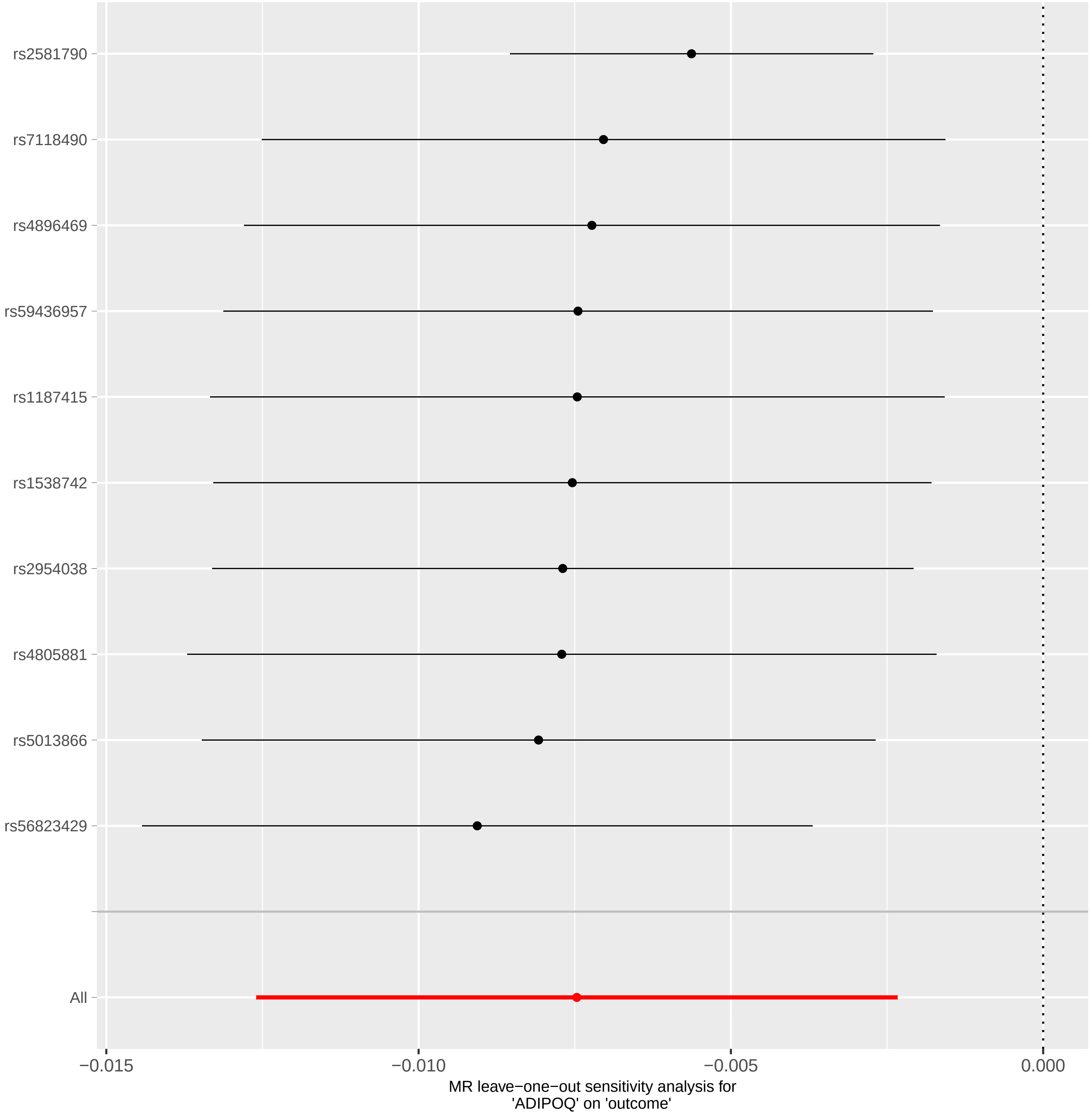

rs2581790
rs7118490
rs4896469
rs59436957
rs1187415
rs1538742
rs2954038
rs4805881
rs5013866
rs56823429
All
−0.015
−0.010
−0.005
0.000
MR leave−one−out sensitivity analysis for
'ADIPOQ' on 'outcome'

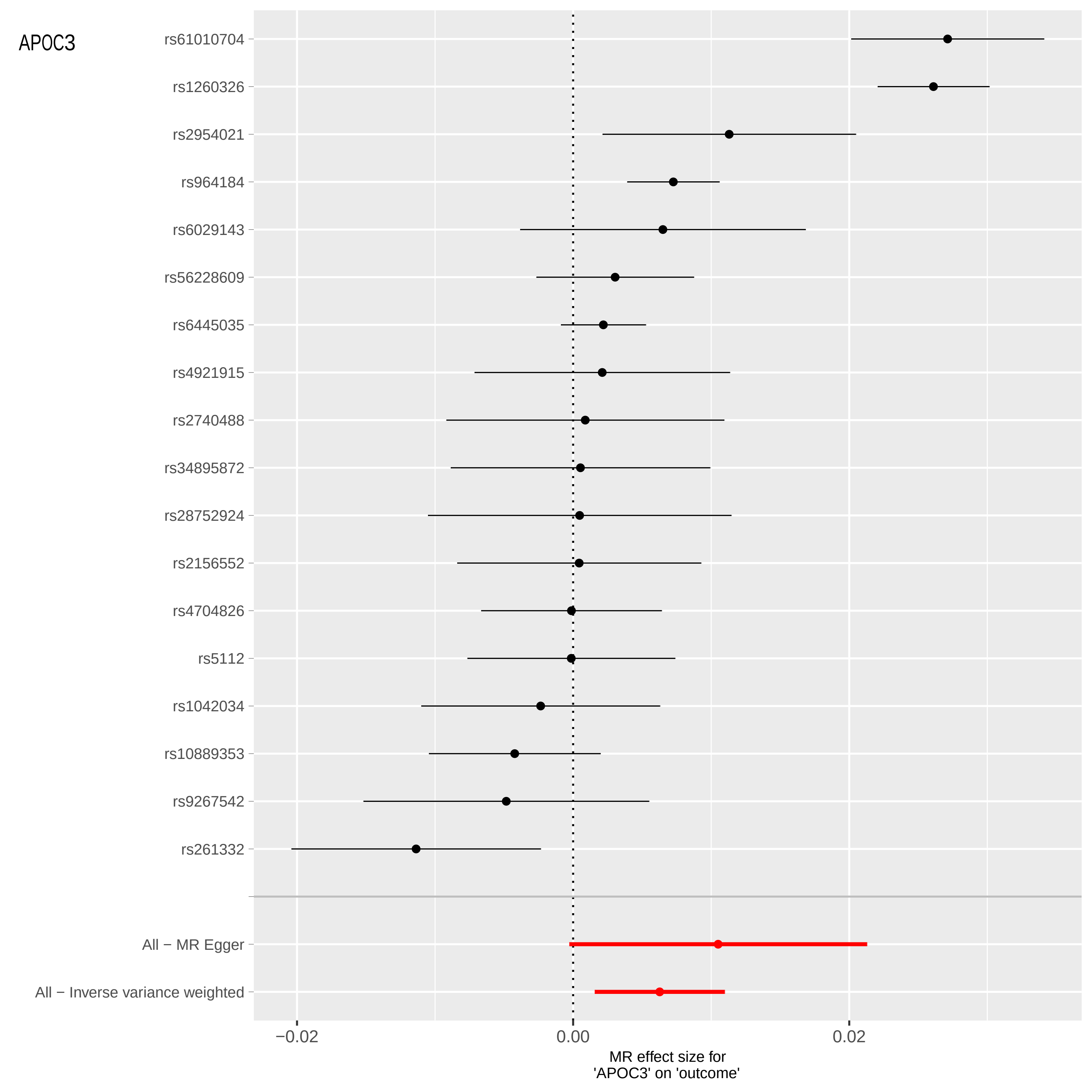
APOC3
rs61010704
rs1260326
rs2954021
rs964184
rs6029143
rs56228609
rs6445035
rs4921915
rs2740488
rs34895872
rs28752924
rs2156552
rs4704826
rs5112
rs1042034
rs10889353
rs9267542
rs261332
All − MR Egger
All − Inverse variance weighted
−0.02
0.00
0.02
MR effect size for
'APOC3' on 'outcome'

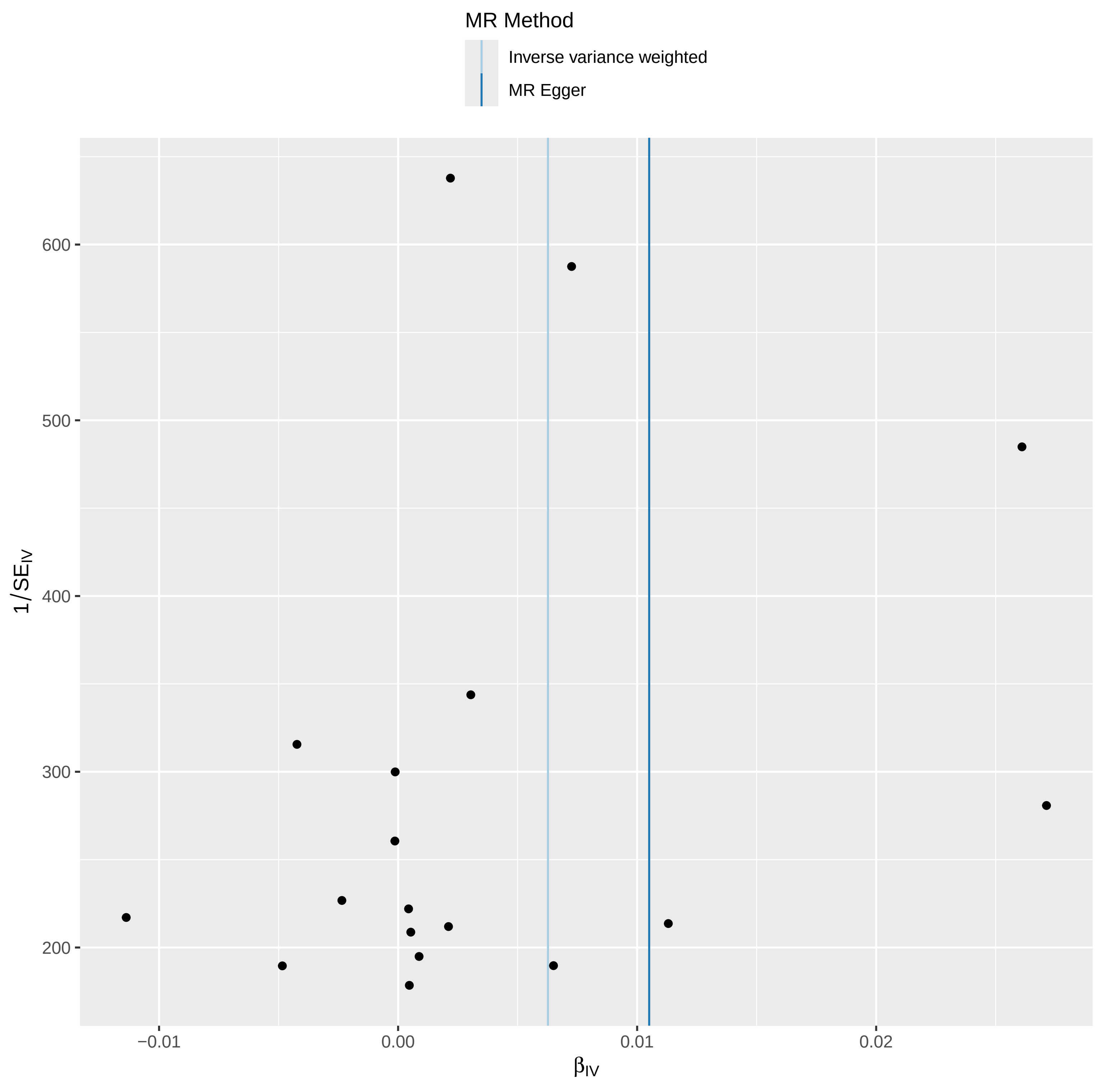

MR Method
Inverse variance weighted
MR Egger
600
500
400
300
200
1/SE_IV
−0.01
0.00
0.01
0.02
β_IV

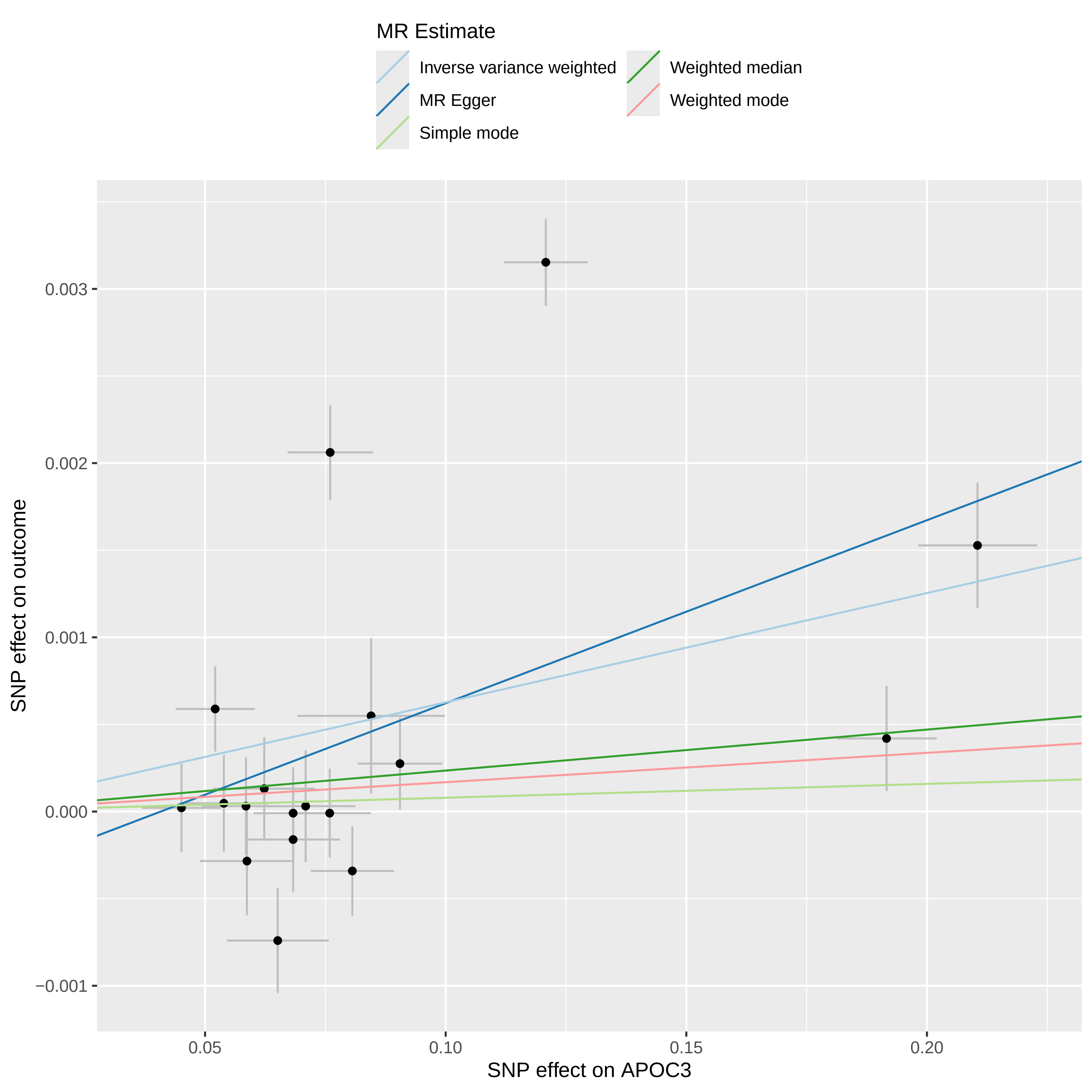

MR Estimate
Inverse variance weighted
MR Egger
Simple mode
Weighted median
Weighted mode
SNP effect on outcome
0.003
0.002
0.001
0.000
−0.001
0.05
0.10
0.15
0.20
SNP effect on APOC3

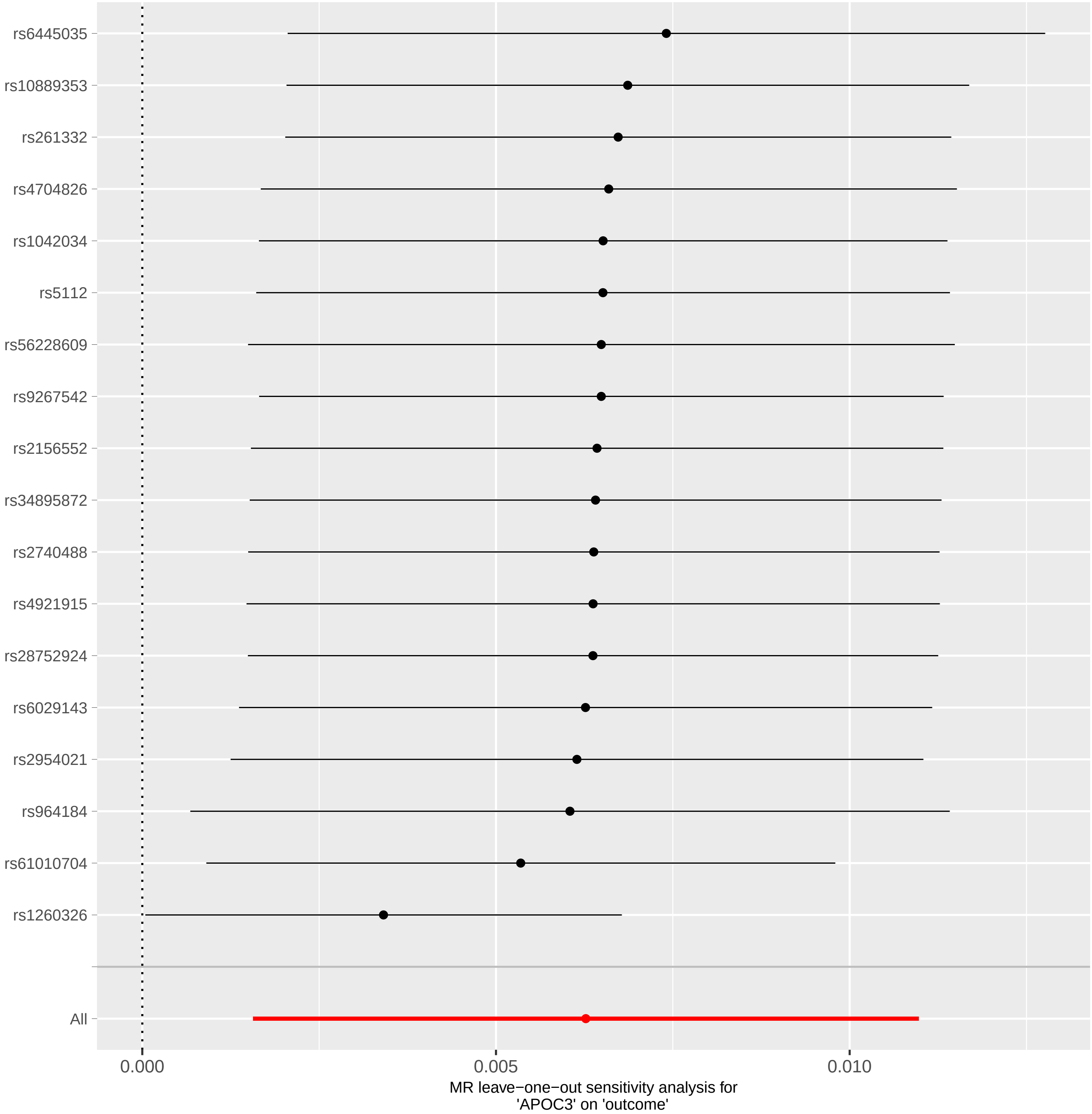

rs6445035
rs10889353
rs261332
rs4704826
rs1042034
rs5112
rs56228609
rs9267542
rs2156552
rs34895872
rs2740488
rs4921915
rs28752924
rs6029143
rs2954021
rs964184
rs61010704
rs1260326
All
0.000
0.005
0.010
MR leave−one−out sensitivity analysis for
'APOC3' on 'outcome'

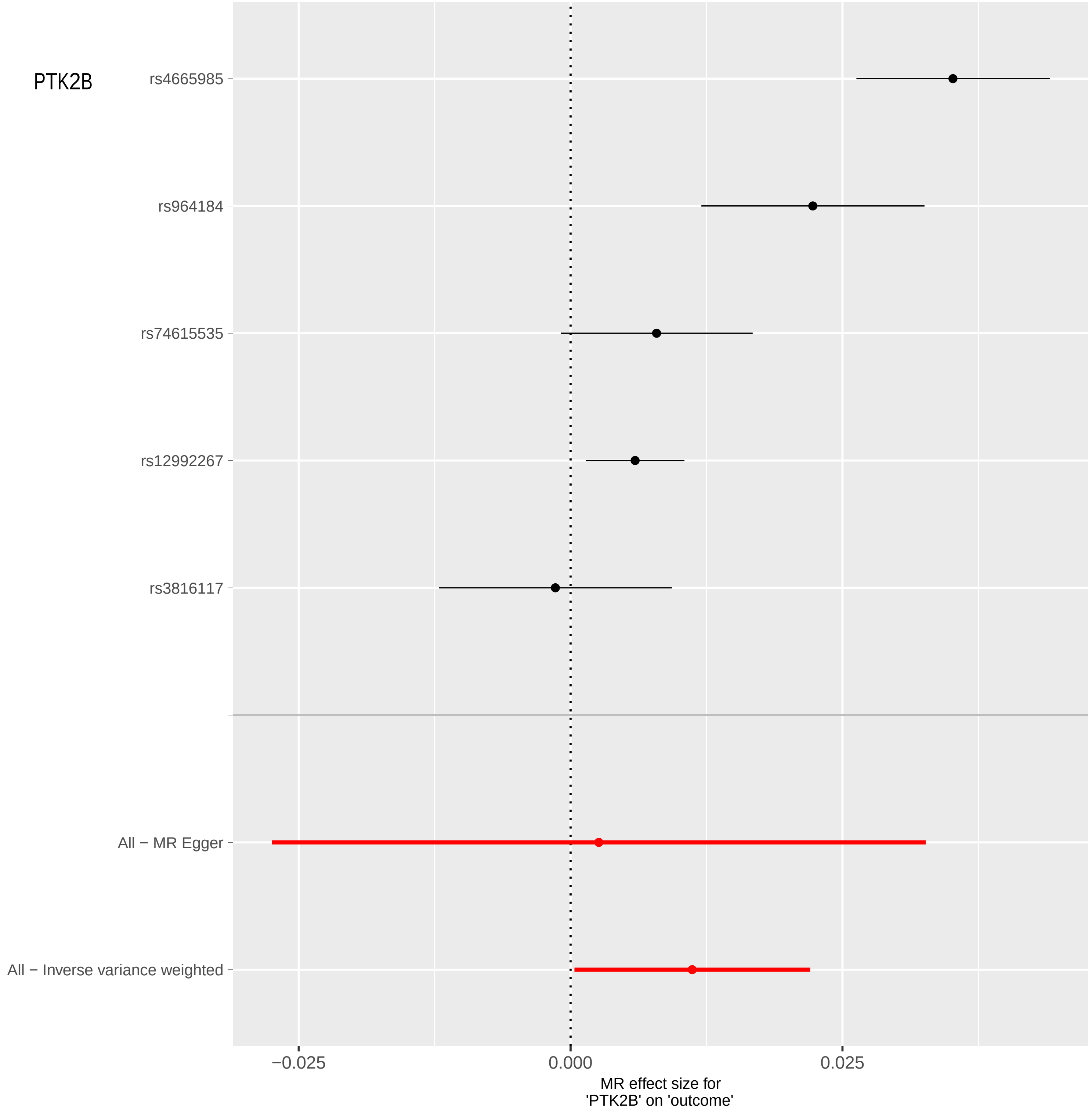
PTK2B
rs4665985
rs964184
rs74615535
rs12992267
rs3816117
All − MR Egger
All − Inverse variance weighted
−0.025
0.000
0.025
MR effect size for
'PTK2B' on 'outcome'

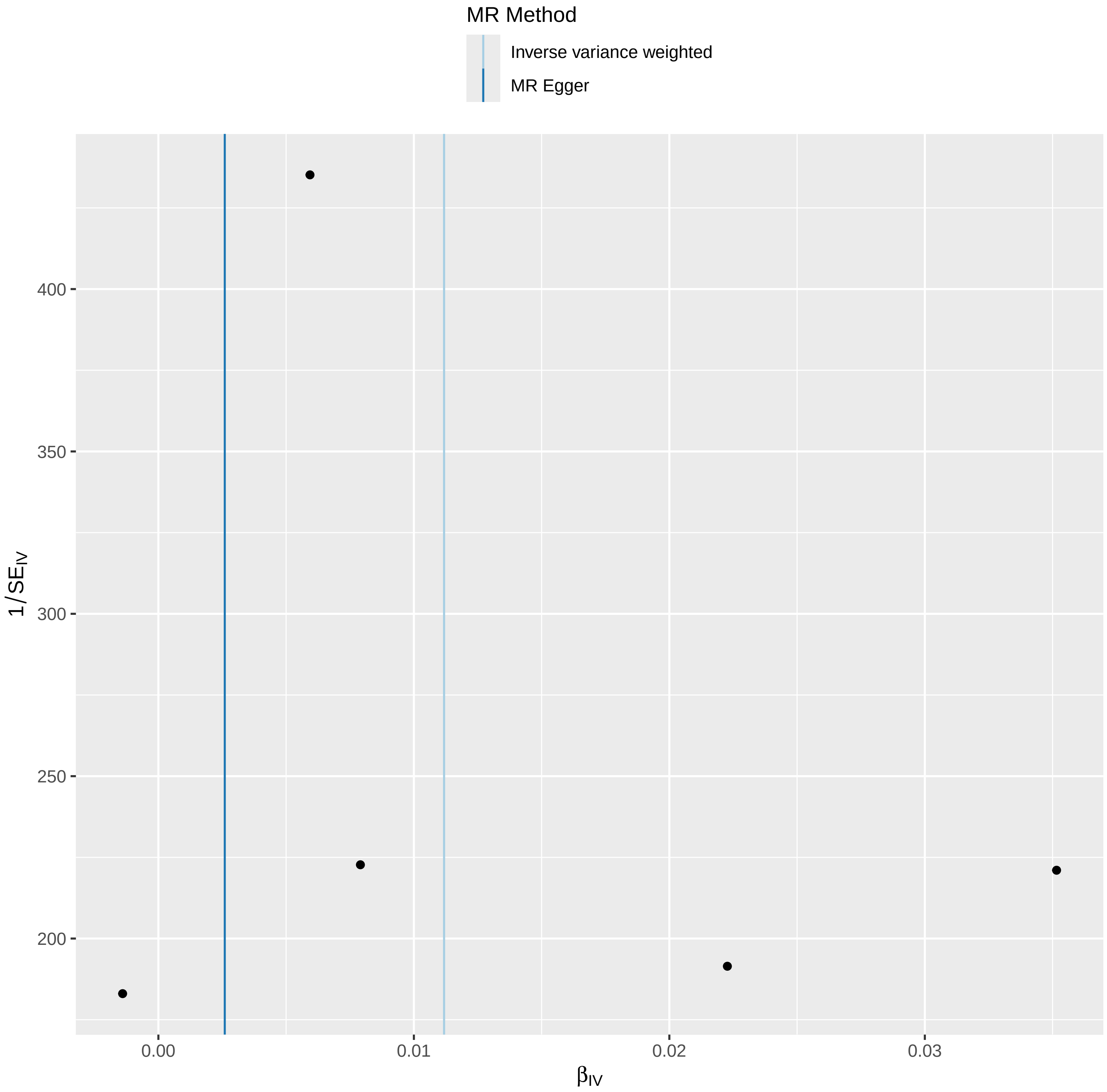
MR Method
Inverse variance weighted
MR Egger
400
350
300
250
200
0.00
0.01
0.02
0.03
1/SE_IV
β_IV

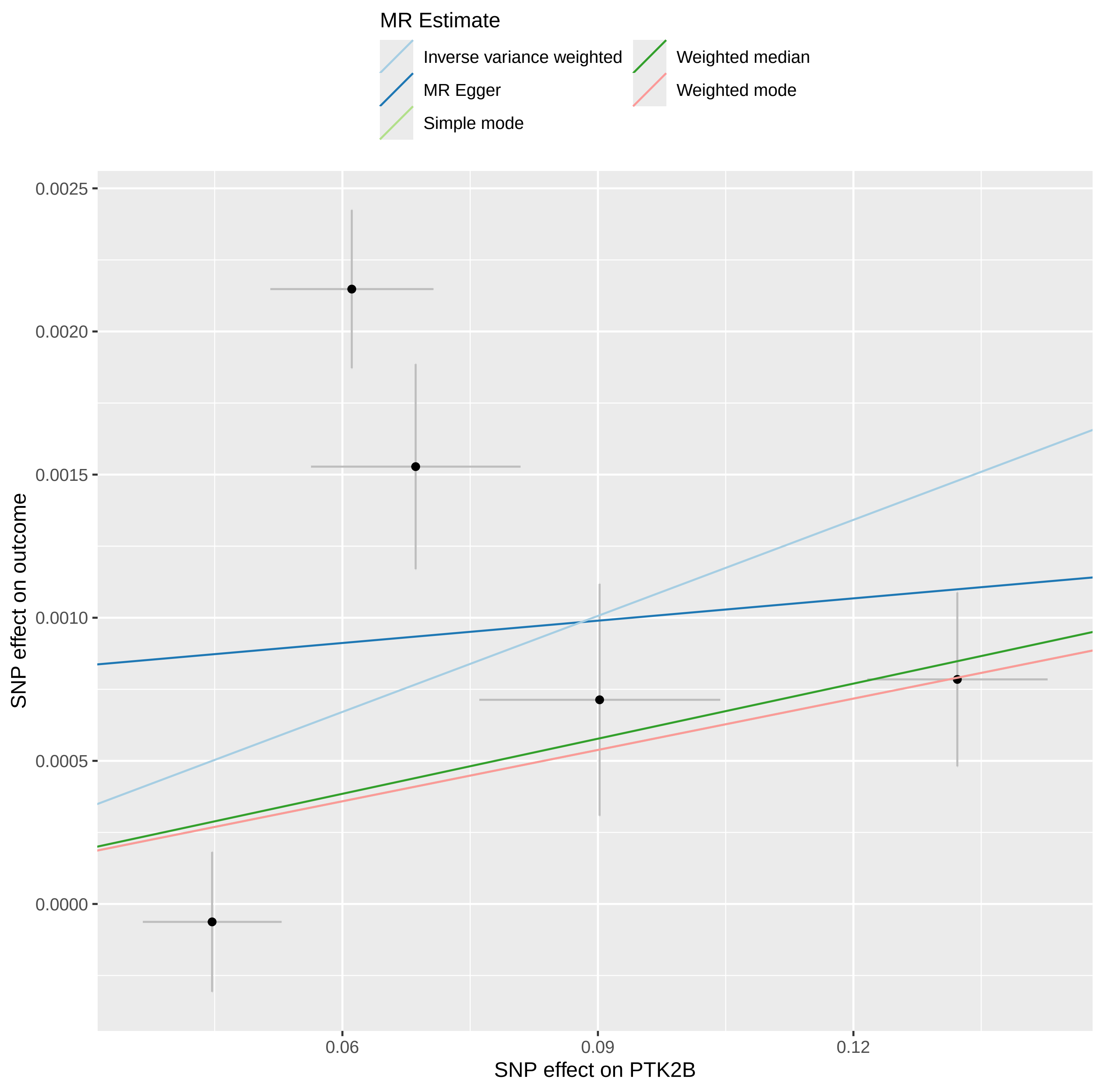
MR Estimate
Inverse variance weighted
MR Egger
Simple mode
Weighted median
Weighted mode
0.0025
0.0020
0.0015
0.0010
0.0005
0.0000
SNP effect on outcome
0.06
0.09
0.12
SNP effect on PTK2B

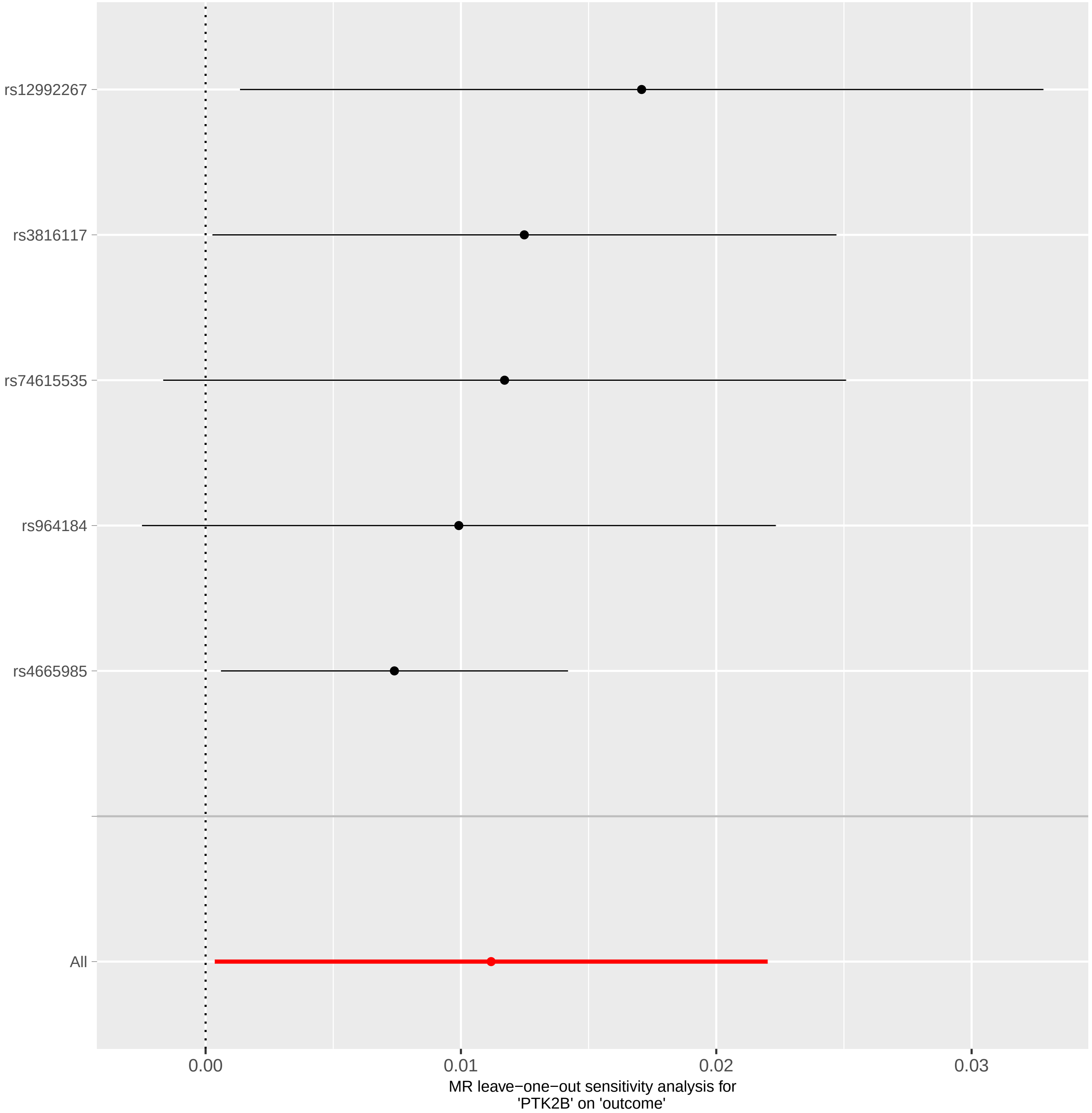
rs12992267
rs3816117
rs74615535
rs964184
rs4665985
All
0.00
0.01
0.02
0.03
MR leave−one−out sensitivity analysis for
'PTK2B' on 'outcome'

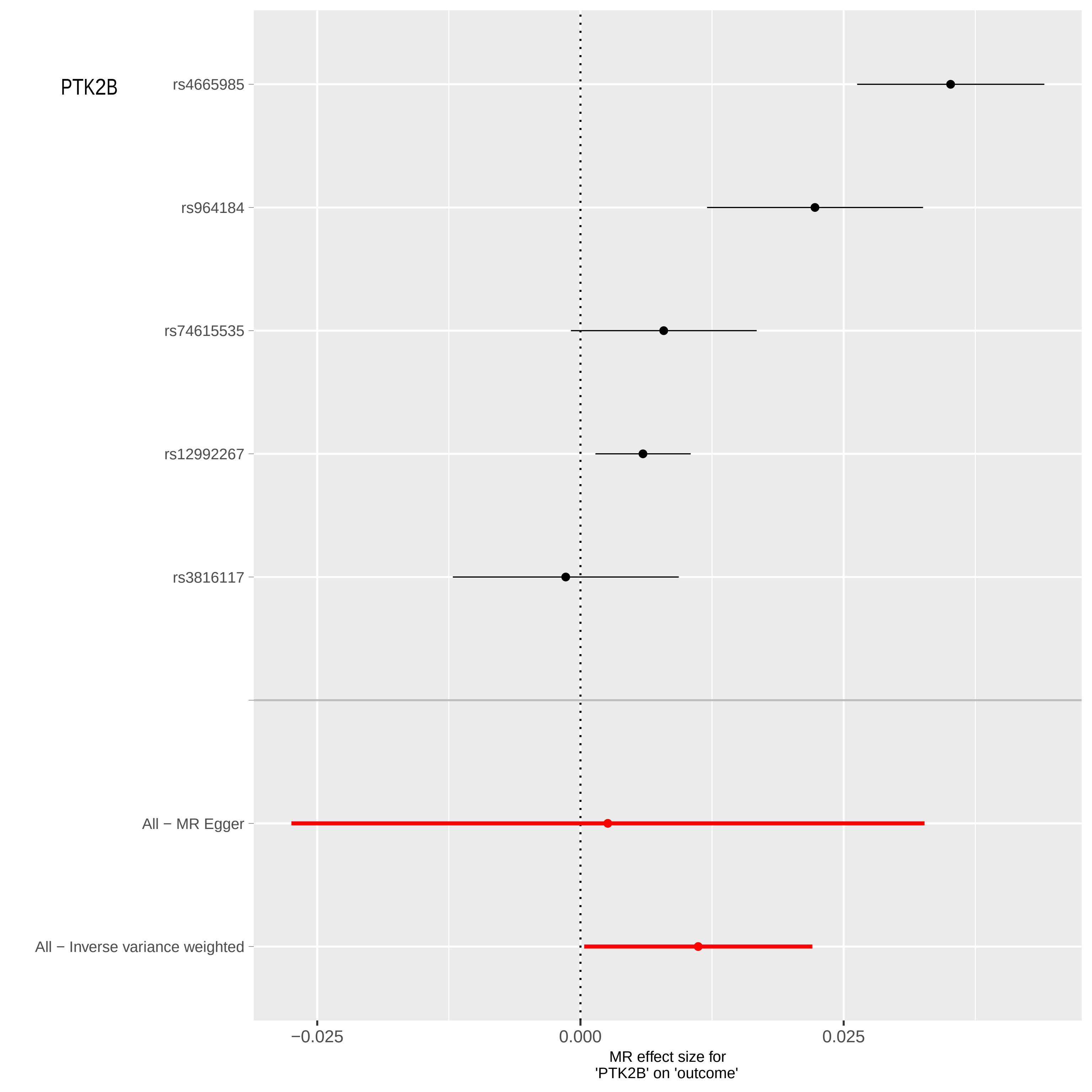
PTK2B
rs4665985
rs964184
rs74615535
rs12992267
rs3816117
All − MR Egger
All − Inverse variance weighted
−0.025
0.000
0.025
MR effect size for
'PTK2B' on 'outcome'

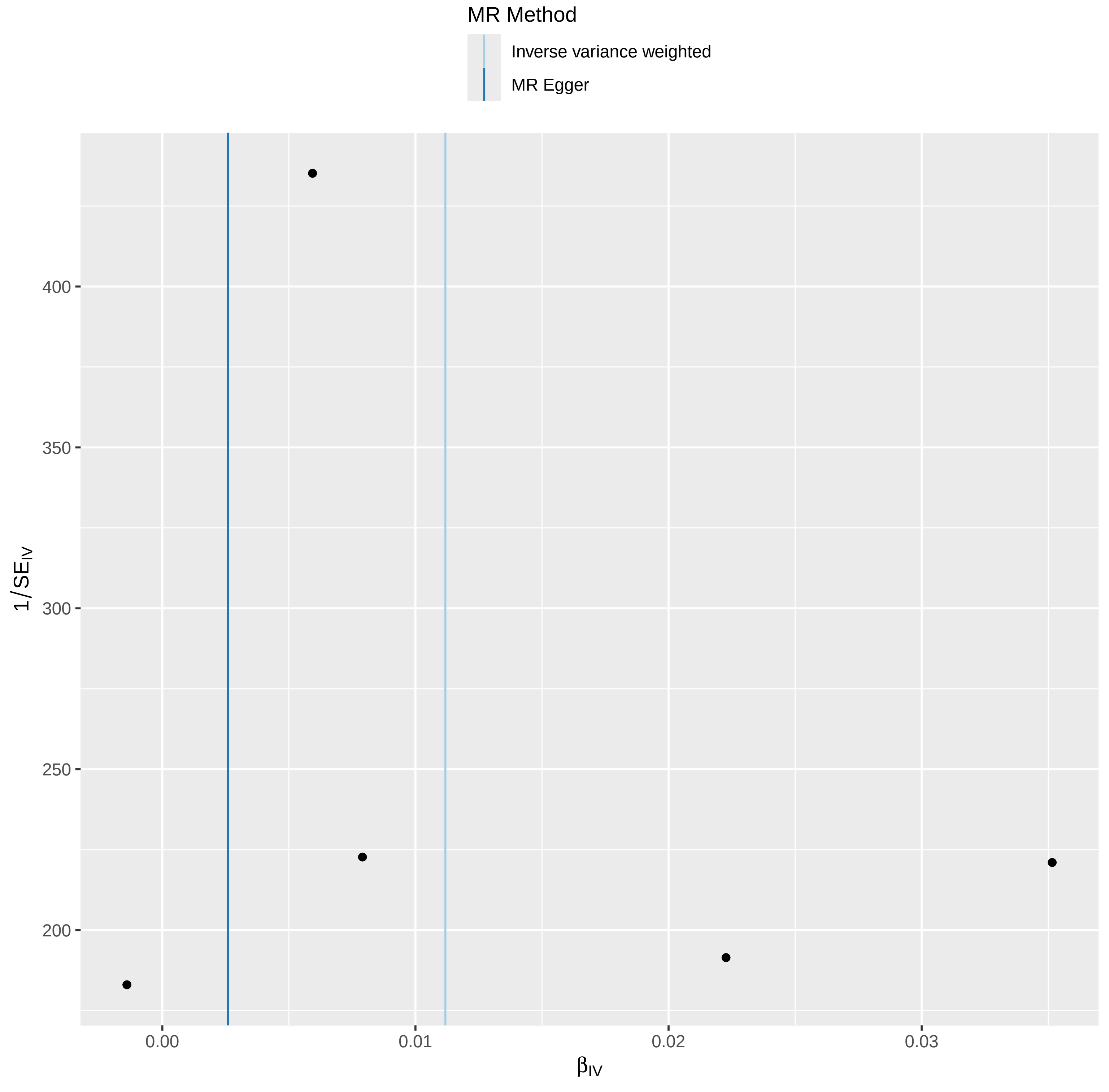

MR Method
Inverse variance weighted
MR Egger
400
350
300
250
200
0.00
0.01
0.02
0.03
$1/SE_{IV}$
β_{IV}

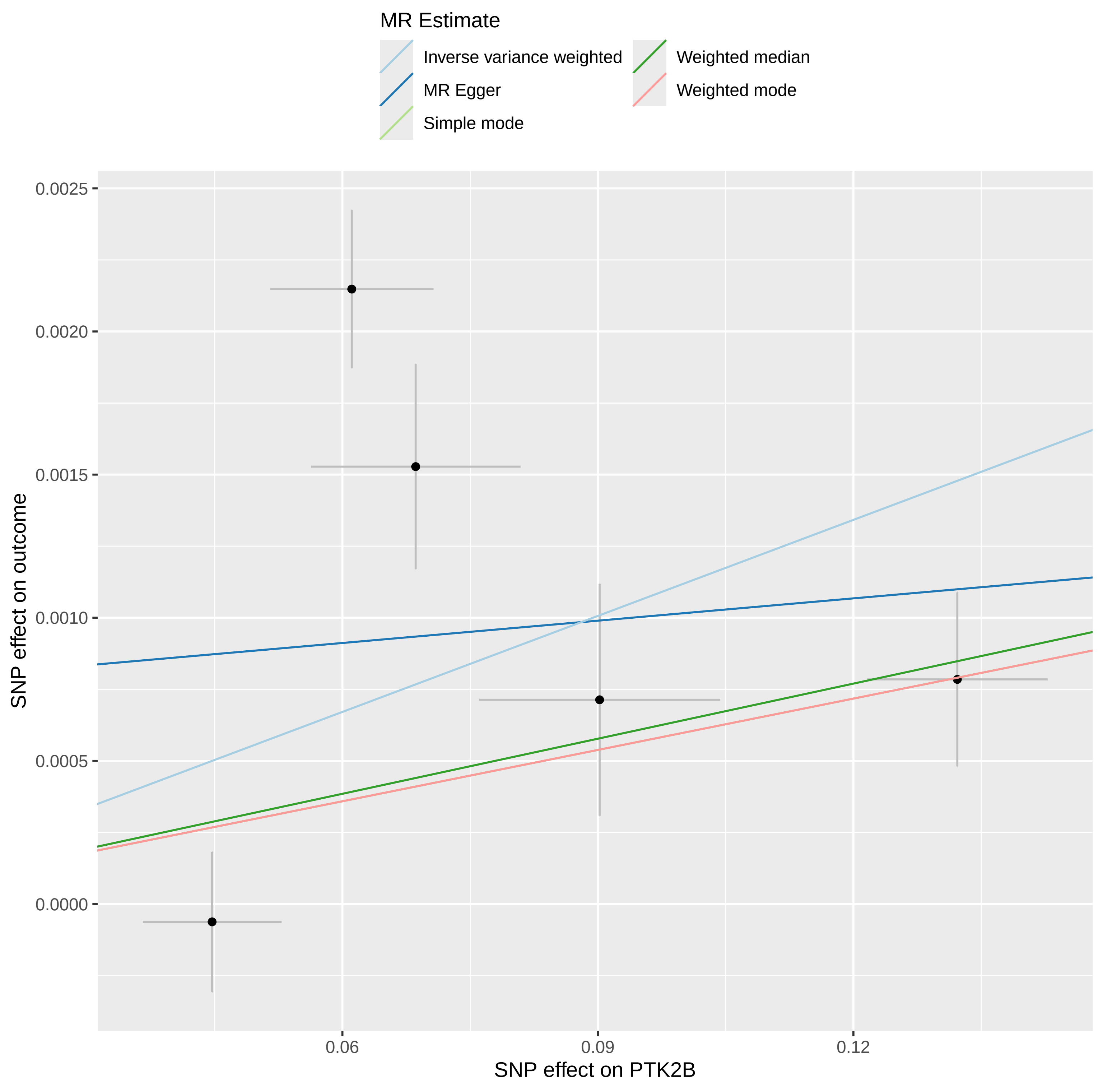
MR Estimate
Inverse variance weighted
MR Egger
Simple mode
Weighted median
Weighted mode
0.0025
0.0020
0.0015
0.0010
0.0005
0.0000
SNP effect on outcome
0.06
0.09
0.12
SNP effect on PTK2B

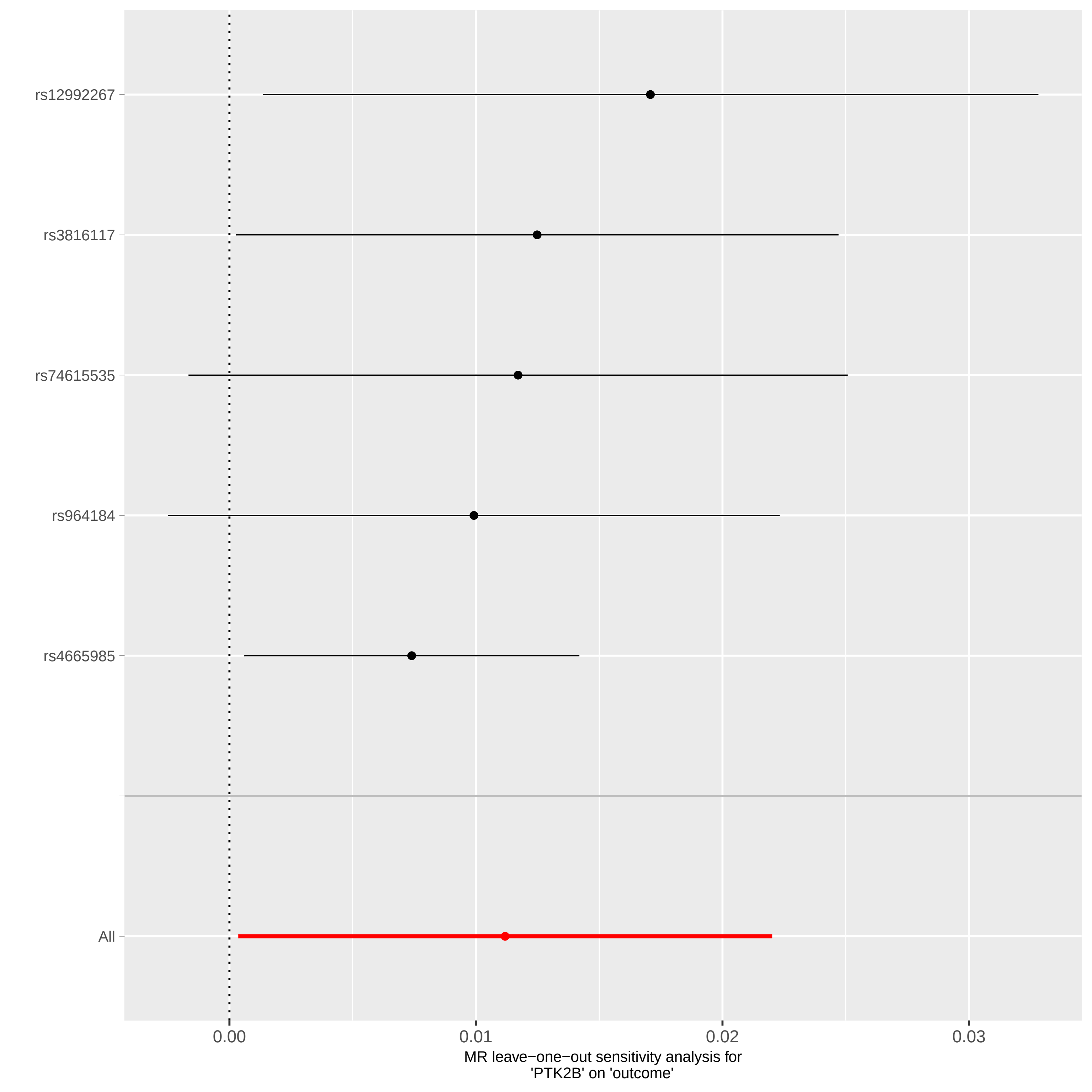

rs12992267
rs3816117
rs74615535
rs964184
rs4665985
All
0.00
0.01
0.02
0.03
MR leave−one−out sensitivity analysis for
'PTK2B' on 'outcome'

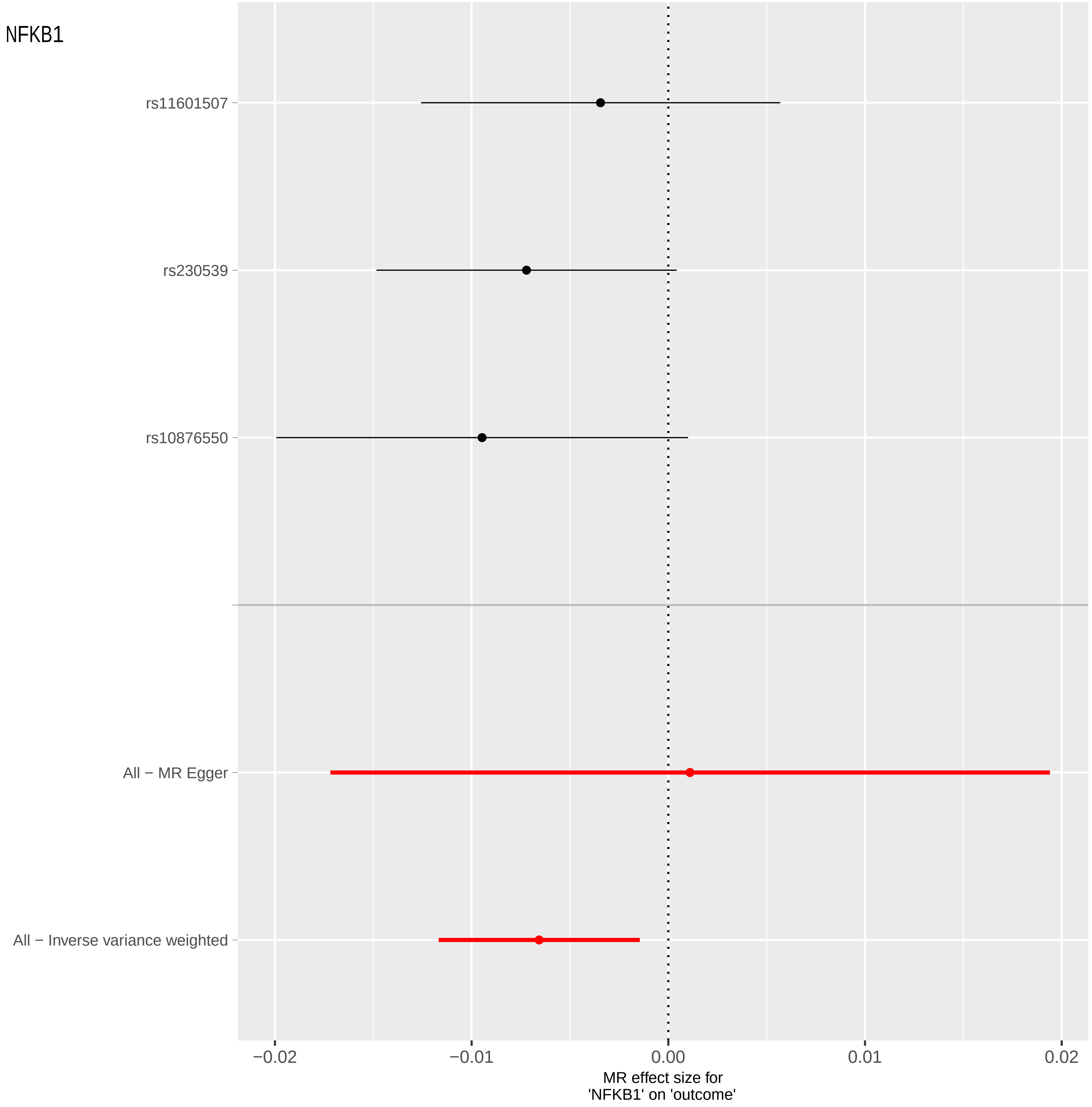

NFKB1
rs11601507
rs230539
rs10876550
All − MR Egger
All − Inverse variance weighted
−0.02
−0.01
0.00
0.01
0.02
MR effect size for
'NFKB1' on 'outcome'

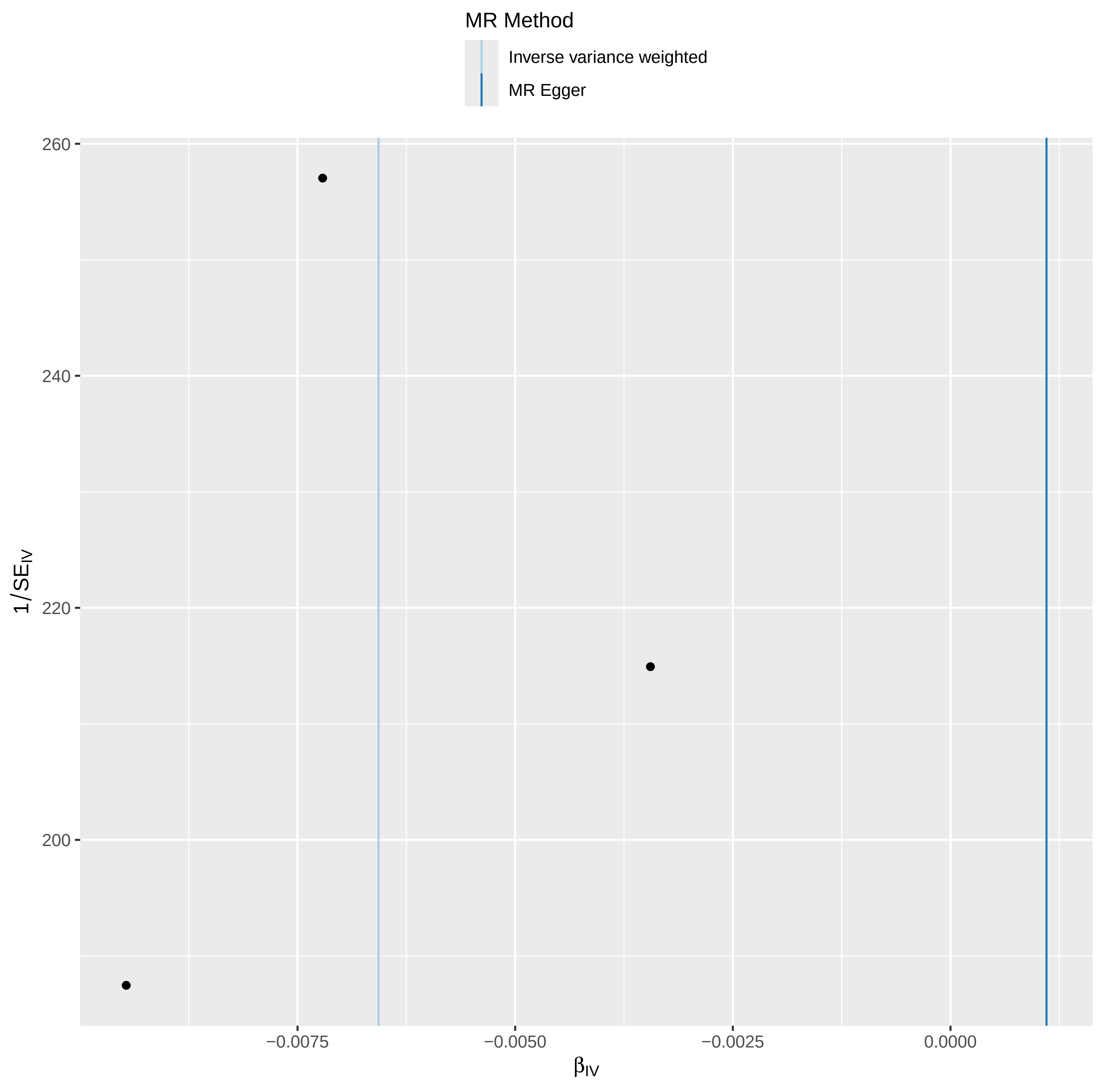

MR Method
Inverse variance weighted
MR Egger
260
240
220
200
1/SE$_{IV}$
−0.0075
−0.0050
−0.0025
0.0000
β_{IV}

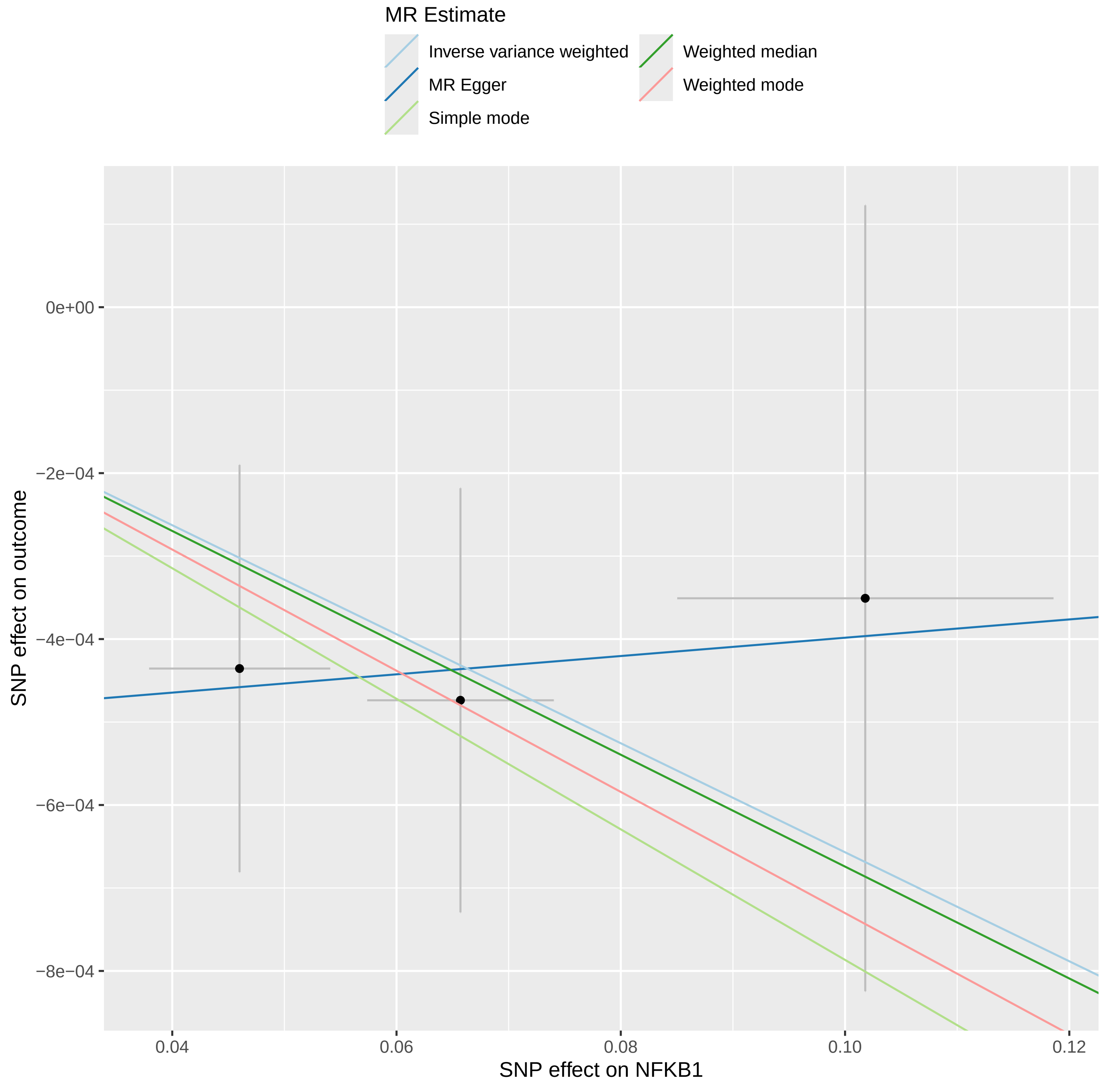

MR Estimate
Inverse variance weighted
MR Egger
Simple mode
Weighted median
Weighted mode
0e+00
−2e−04
−4e−04
−6e−04
−8e−04
SNP effect on outcome
0.04
0.06
0.08
0.10
0.12
SNP effect on NFKB1

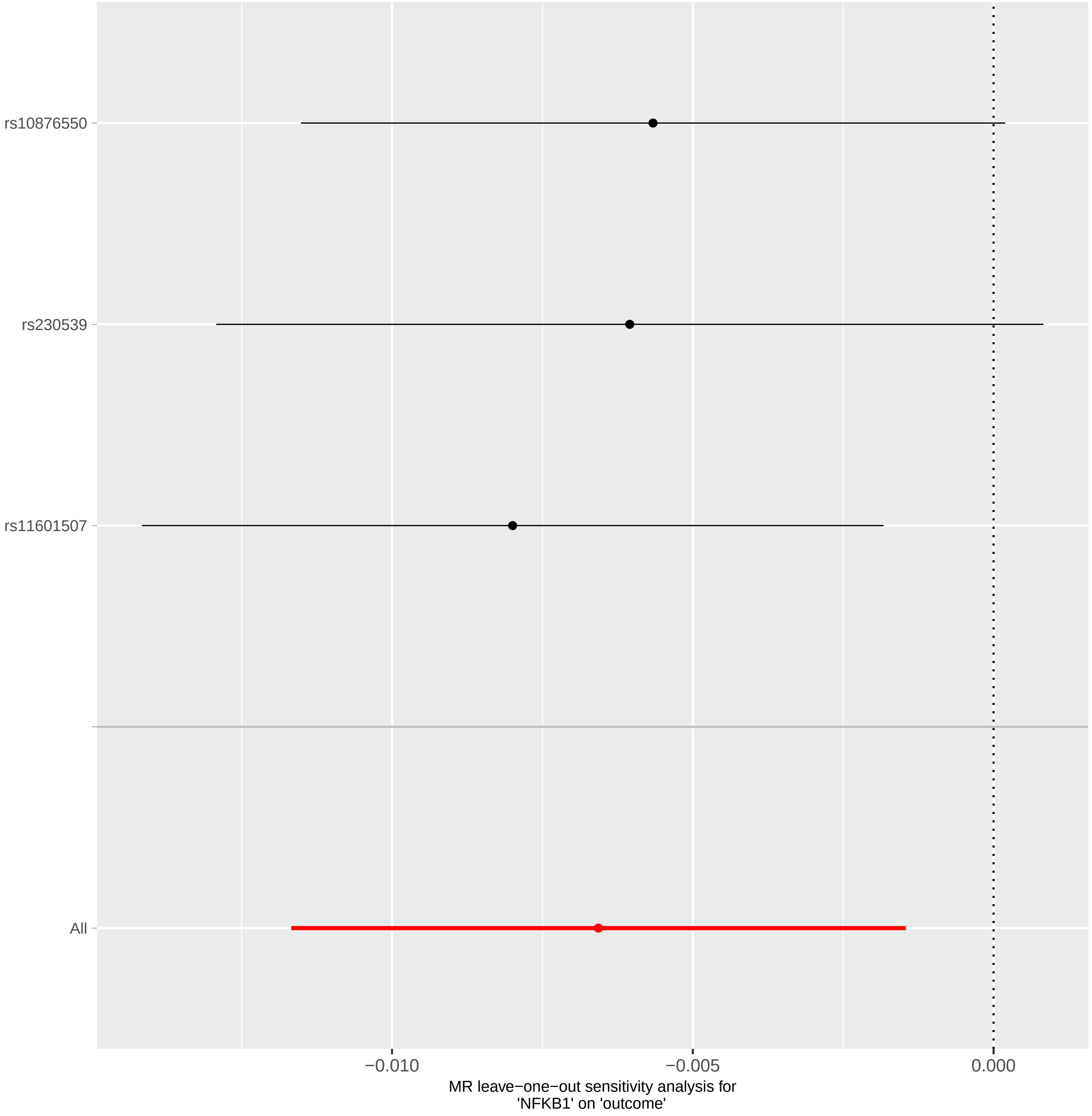

rs10876550
rs230539
rs11601507
All
−0.010
−0.005
0.000
MR leave−one−out sensitivity analysis for
'NFKB1' on 'outcome'

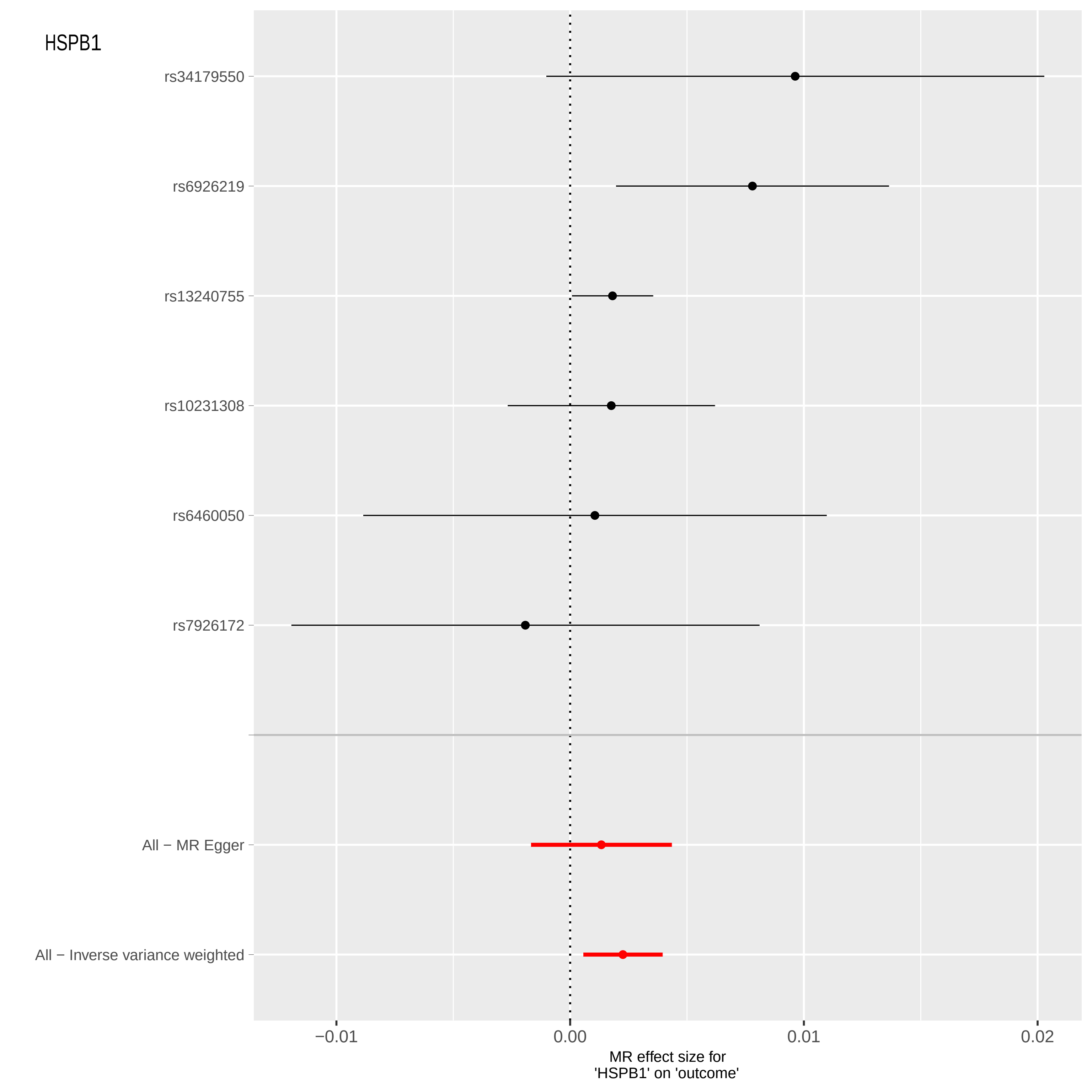
HSPB1
rs34179550
rs6926219
rs13240755
rs10231308
rs6460050
rs7926172
All − MR Egger
All − Inverse variance weighted
−0.01
0.00
0.01
0.02
MR effect size for
'HSPB1' on 'outcome'

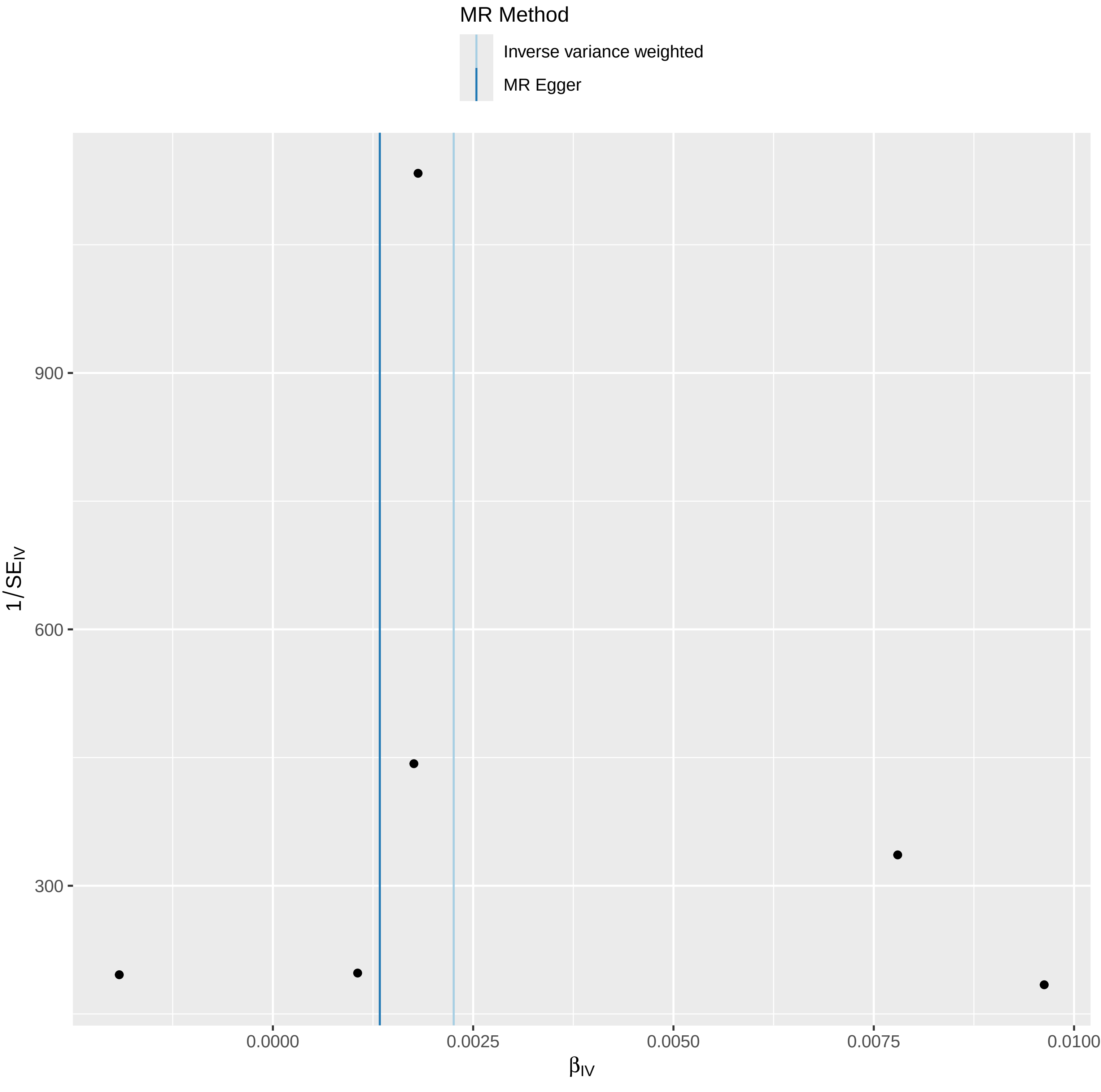

MR Method
Inverse variance weighted
MR Egger
900
600
300
1/SE_IV
0.0000
0.0025
0.0050
0.0075
0.0100
β_IV

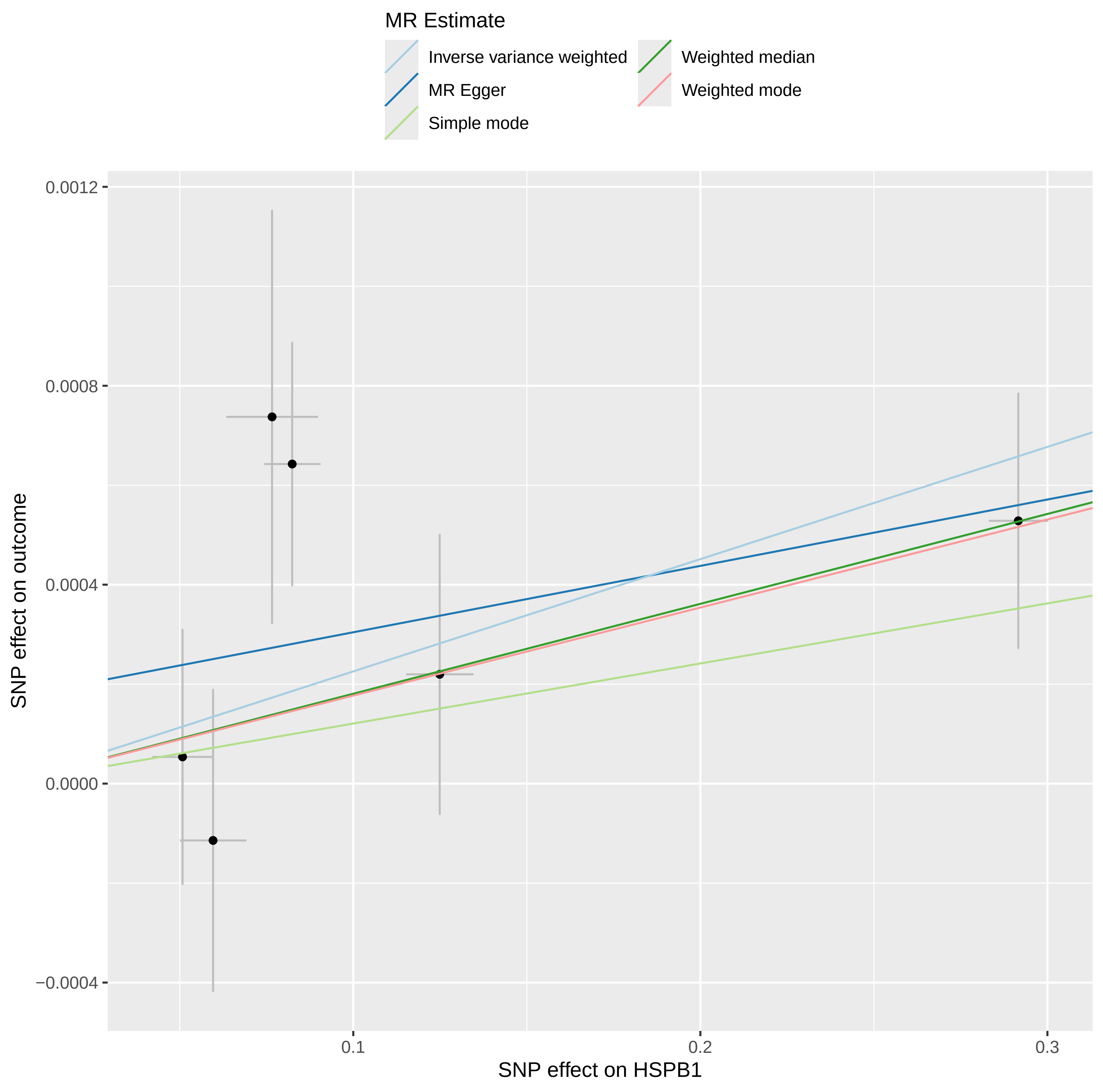

MR Estimate
Inverse variance weighted
MR Egger
Simple mode
Weighted median
Weighted mode
0.0012
0.0008
0.0004
0.0000
−0.0004
SNP effect on outcome
0.1
0.2
0.3
SNP effect on HSPB1

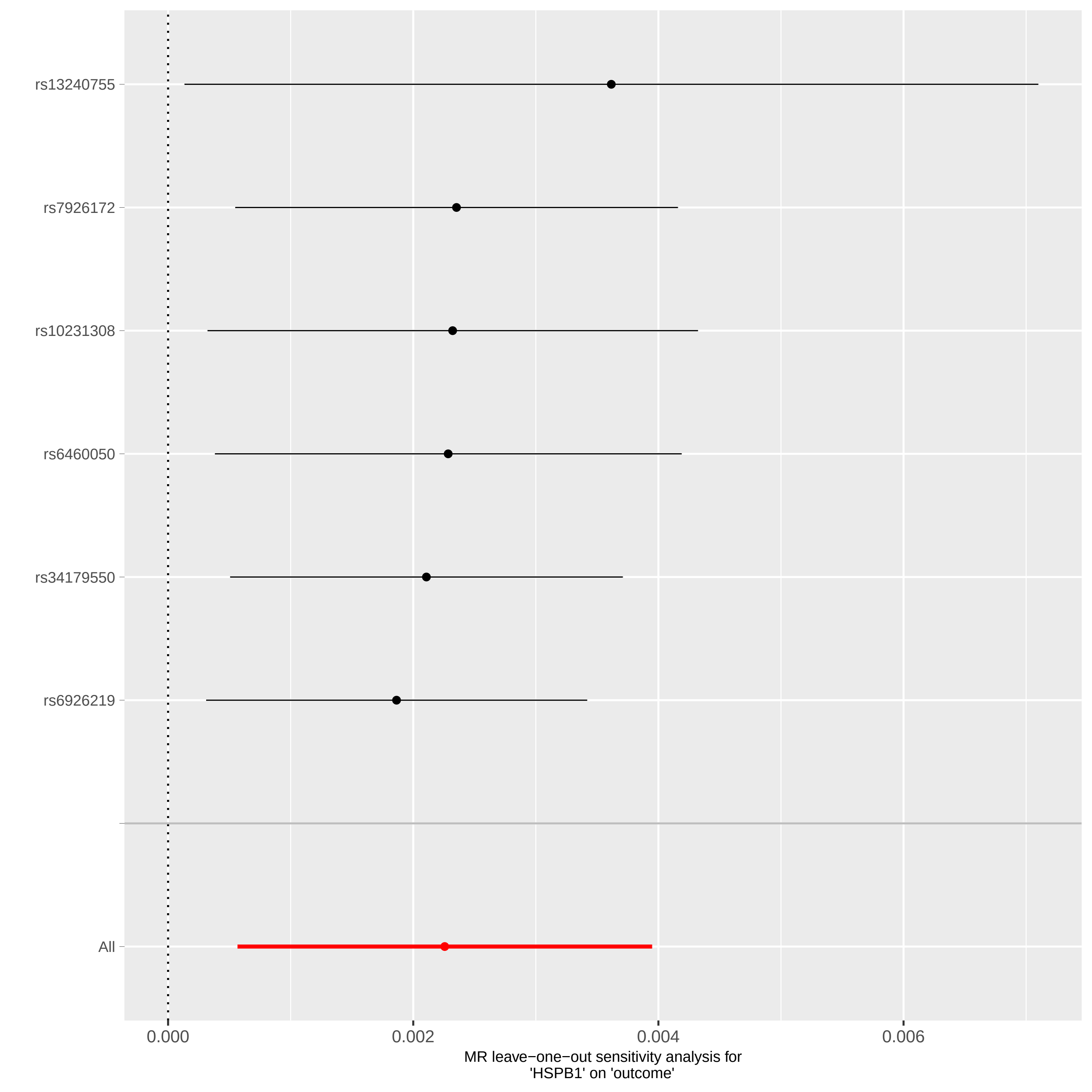
rs13240755
rs7926172
rs10231308
rs6460050
rs34179550
rs6926219
All
0.000
0.002
0.004
0.006
MR leave−one−out sensitivity analysis for
'HSPB1' on 'outcome'

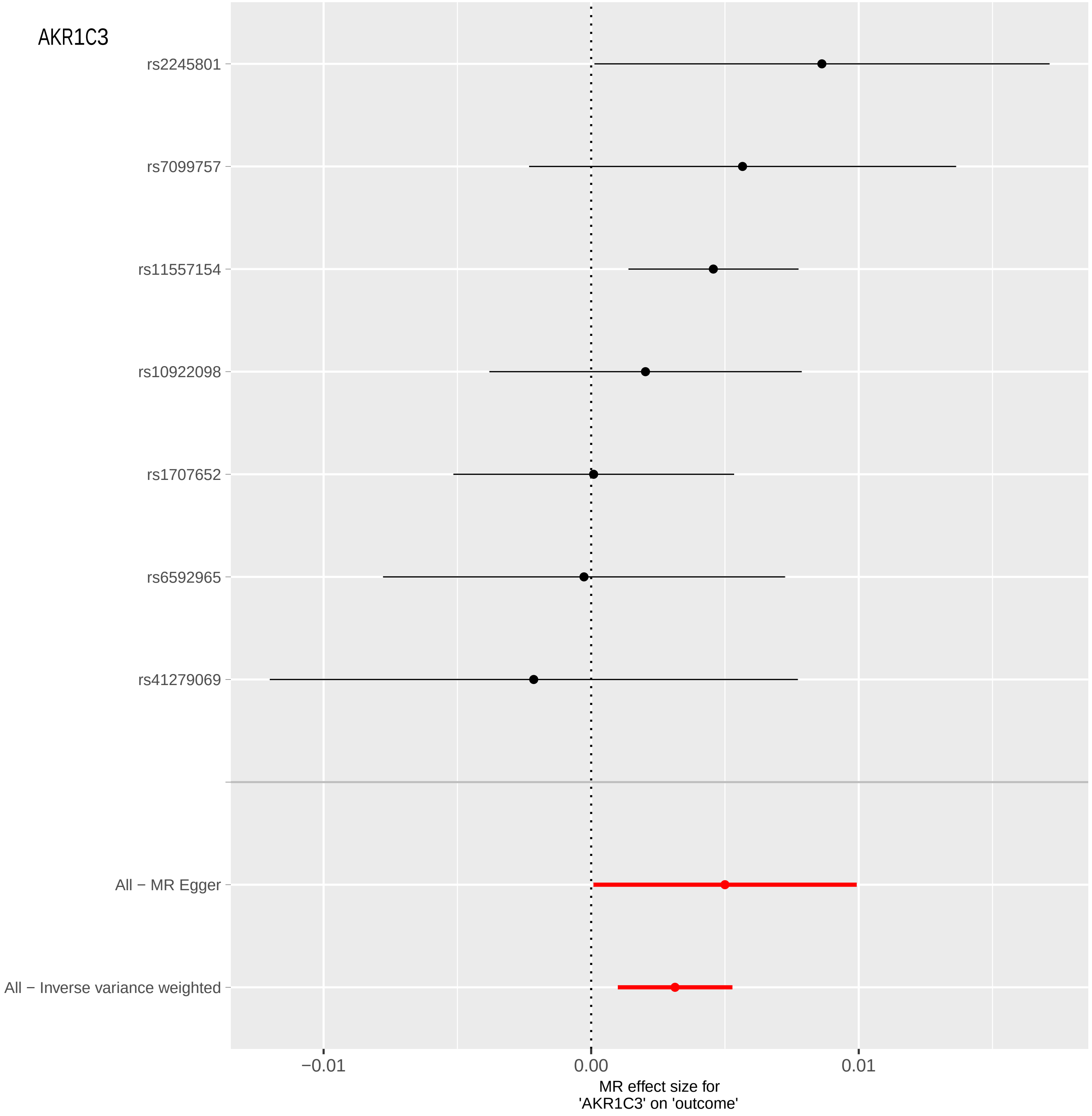
AKR1C3
rs2245801
rs7099757
rs11557154
rs10922098
rs1707652
rs6592965
rs41279069
All − MR Egger
All − Inverse variance weighted
−0.01
0.00
0.01
MR effect size for
'AKR1C3' on 'outcome'

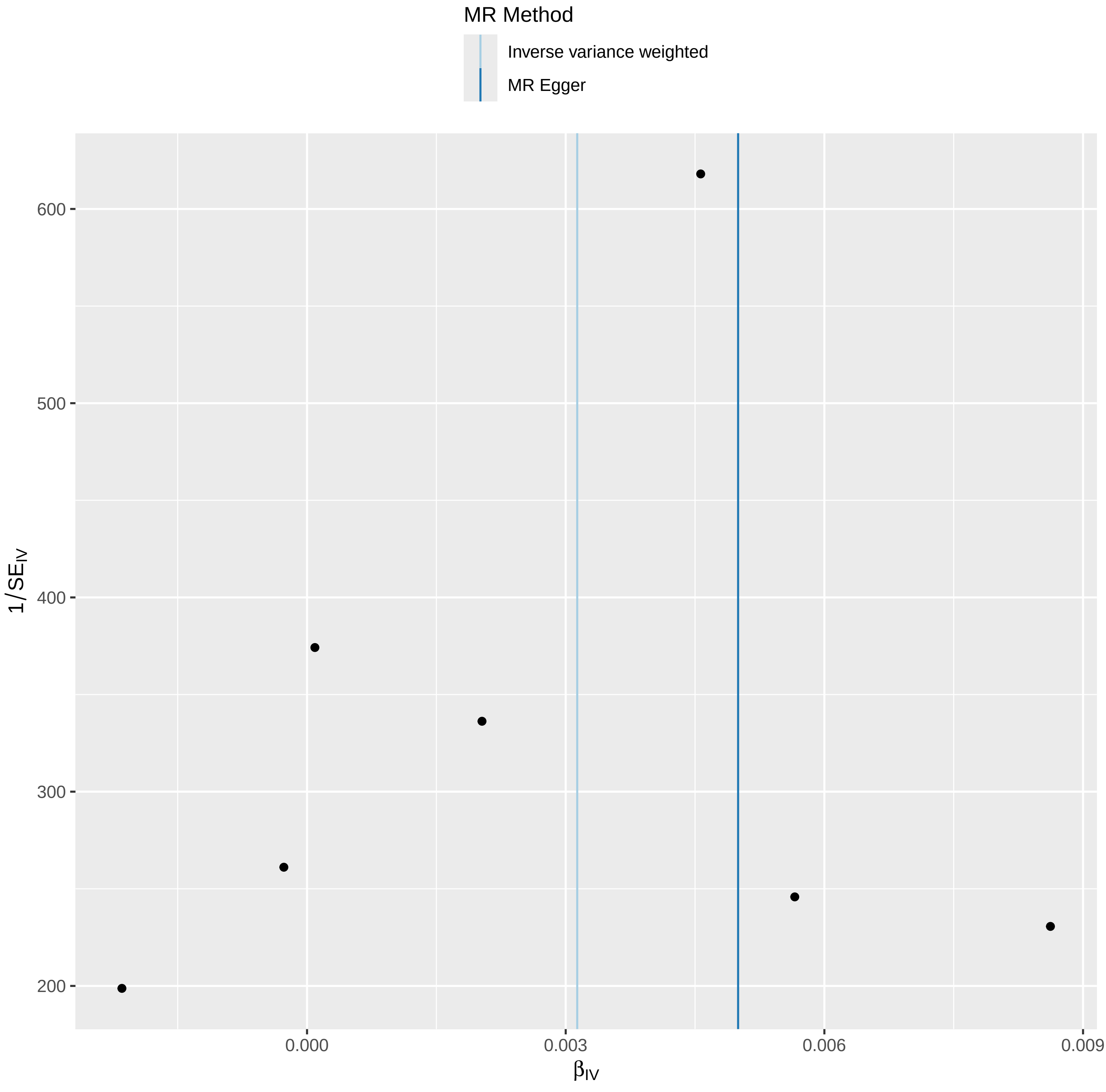

MR Method
Inverse variance weighted
MR Egger
600
500
400
300
200
1/SE_IV
0.000
0.003
0.006
0.009
β_IV

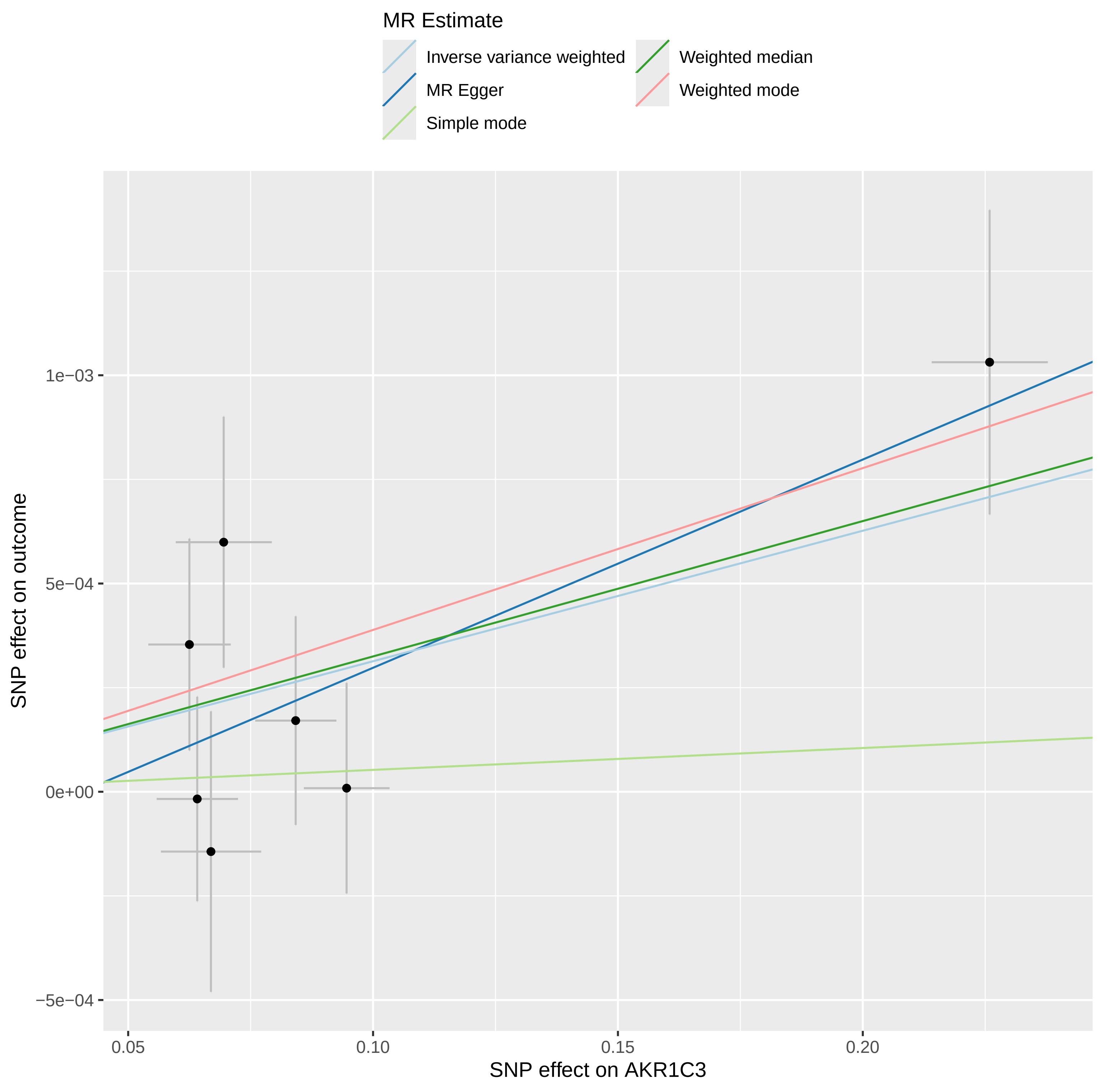

MR Estimate
Inverse variance weighted
MR Egger
Simple mode
Weighted median
Weighted mode
SNP effect on outcome
1e−03
5e−04
0e+00
−5e−04
0.05
0.10
0.15
0.20
SNP effect on AKR1C3

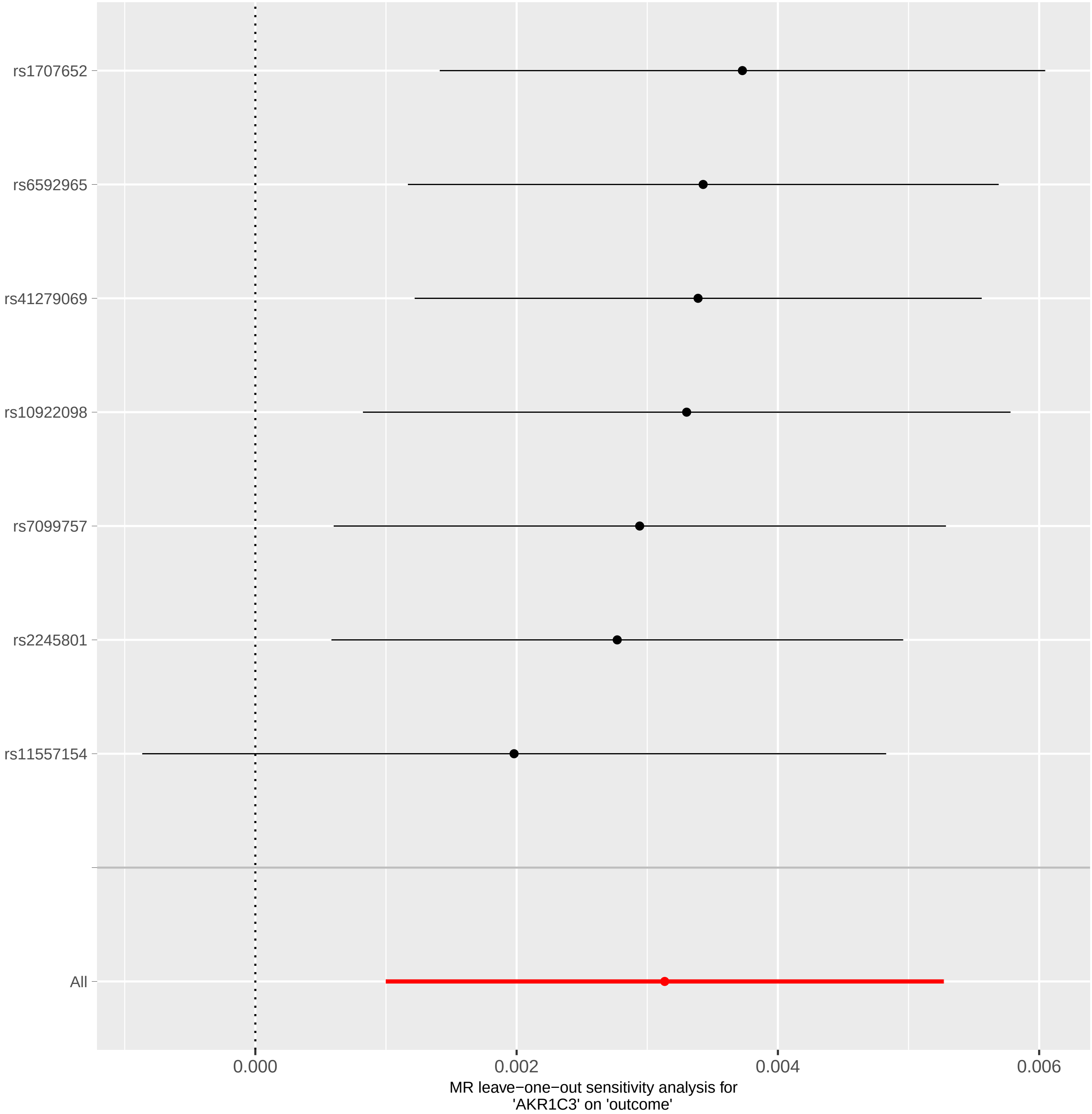

rs1707652
rs6592965
rs41279069
rs10922098
rs7099757
rs2245801
rs11557154
All
0.000
0.002
0.004
0.006
MR leave−one−out sensitivity analysis for
'AKR1C3' on 'outcome'

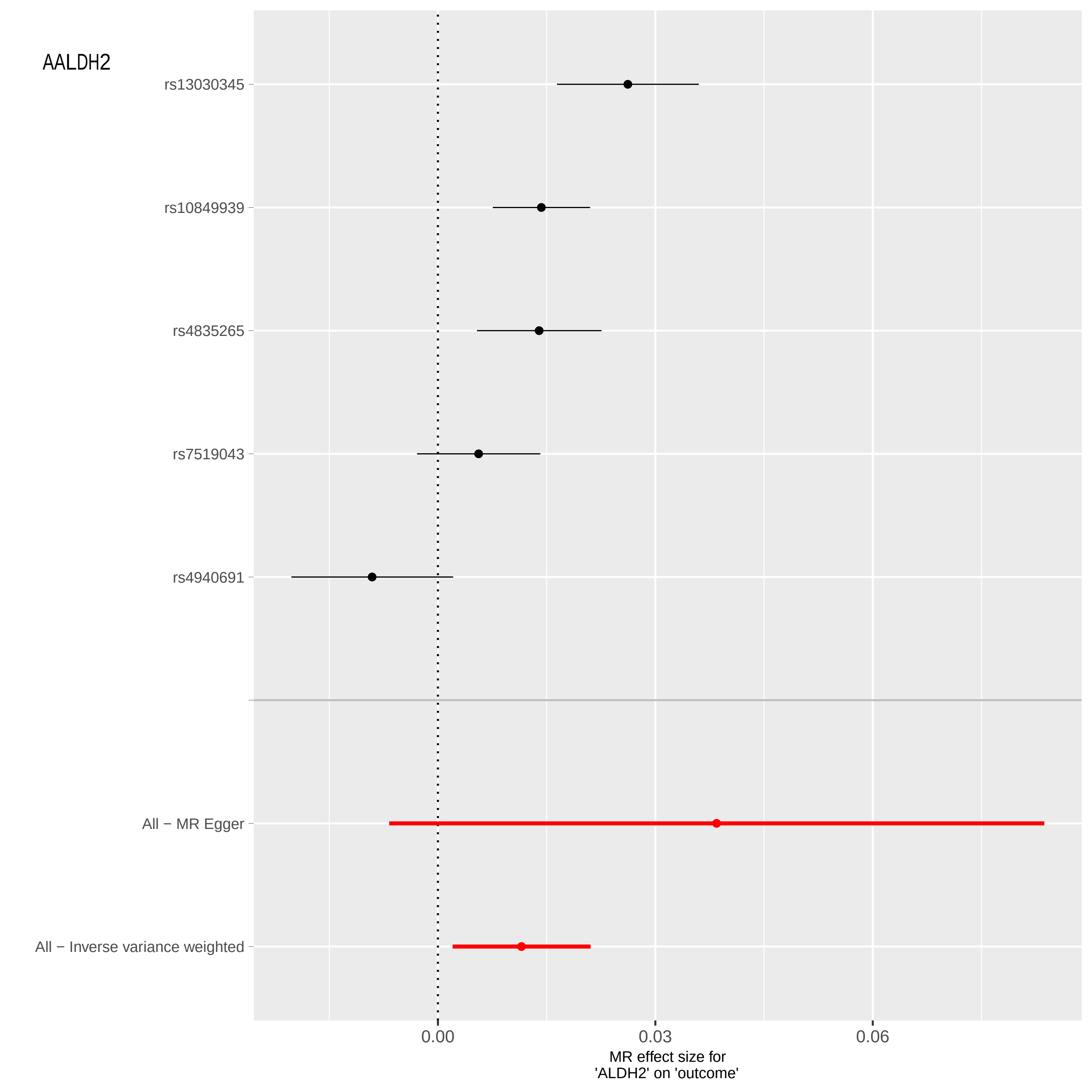
AALDH2
rs13030345
rs10849939
rs4835265
rs7519043
rs4940691
All − MR Egger
All − Inverse variance weighted
0.00
0.03
0.06
MR effect size for
'ALDH2' on 'outcome'

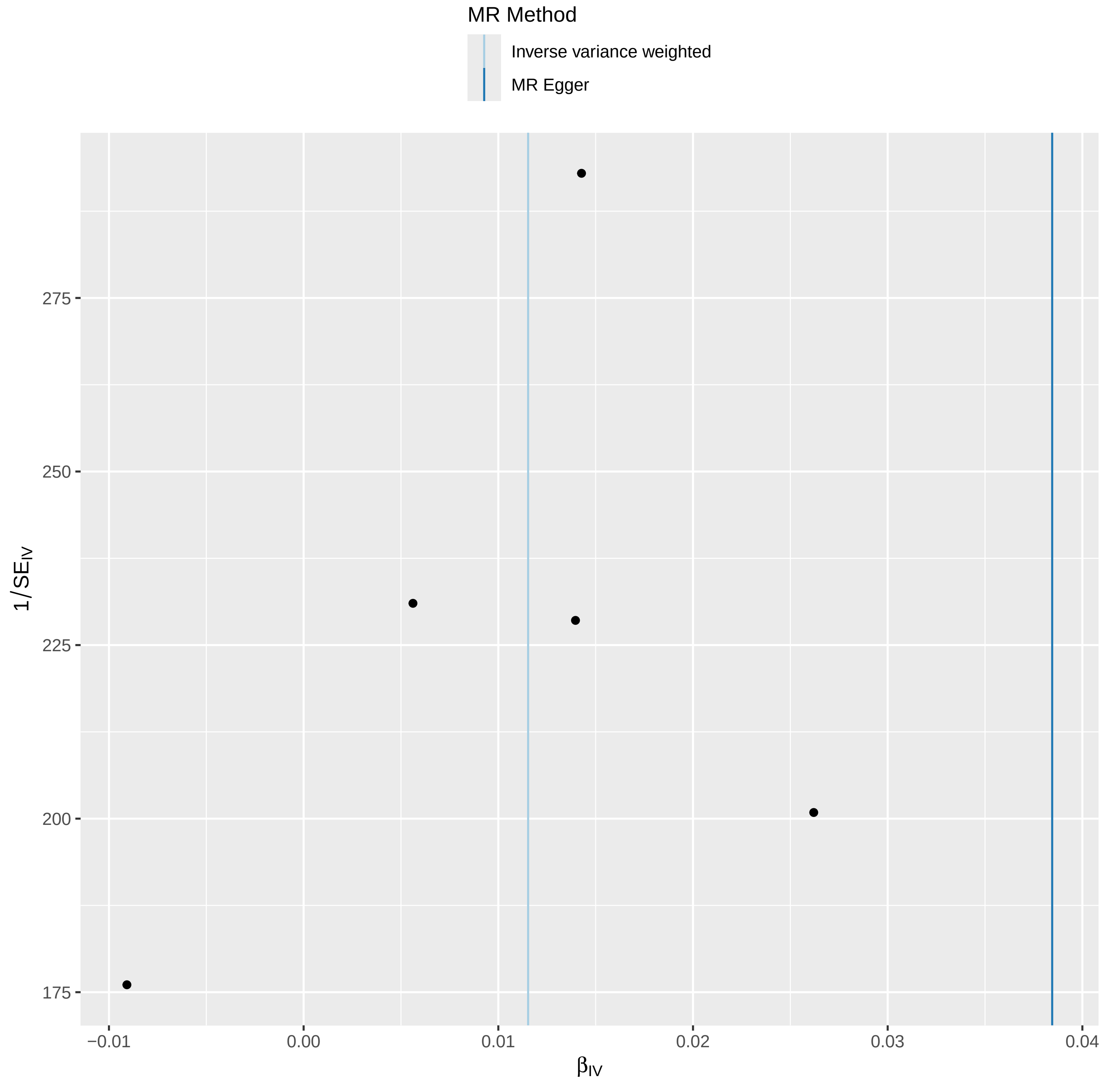
MR Method
Inverse variance weighted
MR Egger
1/SE_IV
275
250
225
200
175
−0.01
0.00
0.01
0.02
0.03
0.04
β_IV

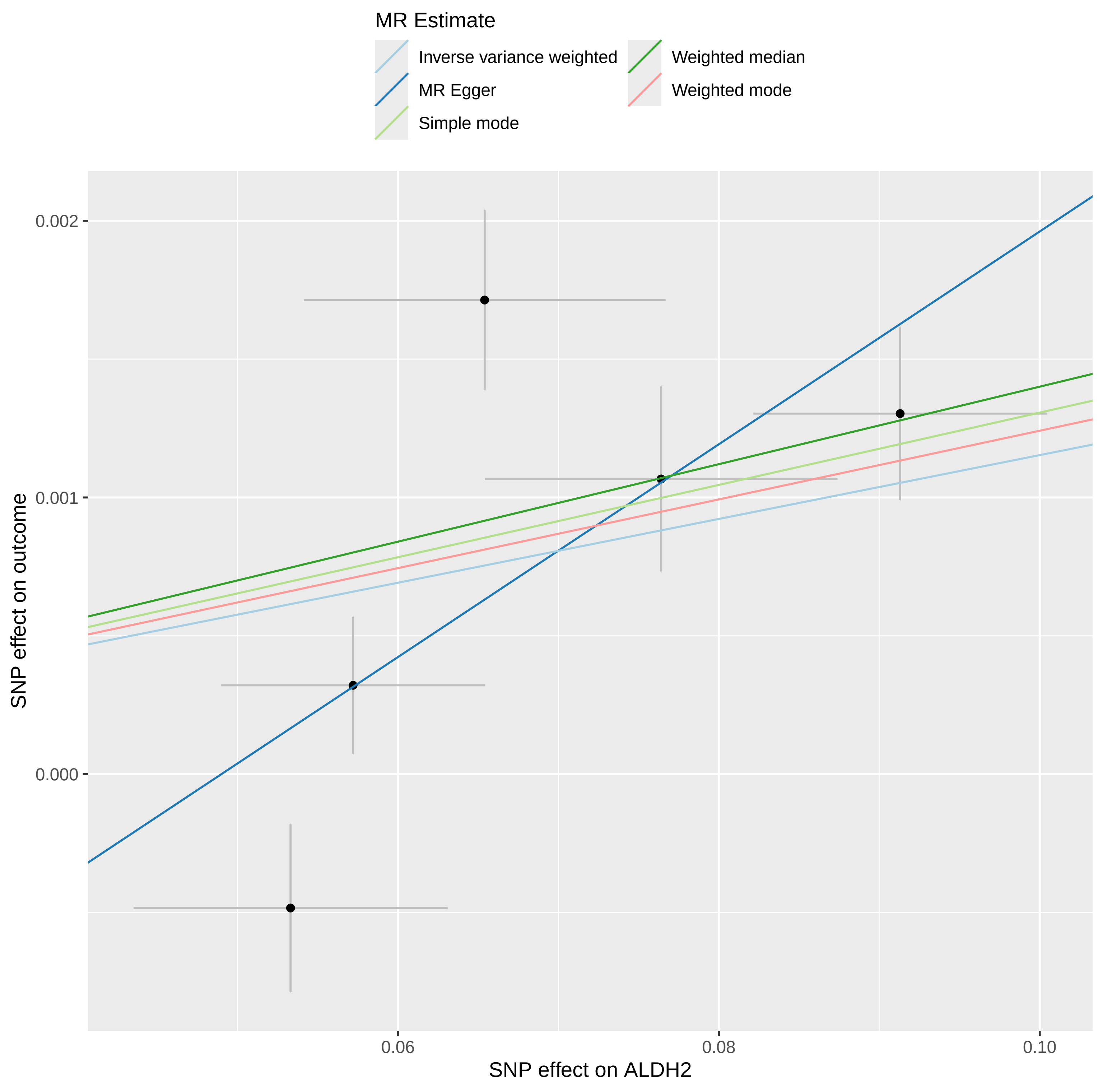
MR Estimate
Inverse variance weighted
MR Egger
Simple mode
Weighted median
Weighted mode
0.002
0.001
0.000
SNP effect on outcome
0.06
0.08
0.10
SNP effect on ALDH2

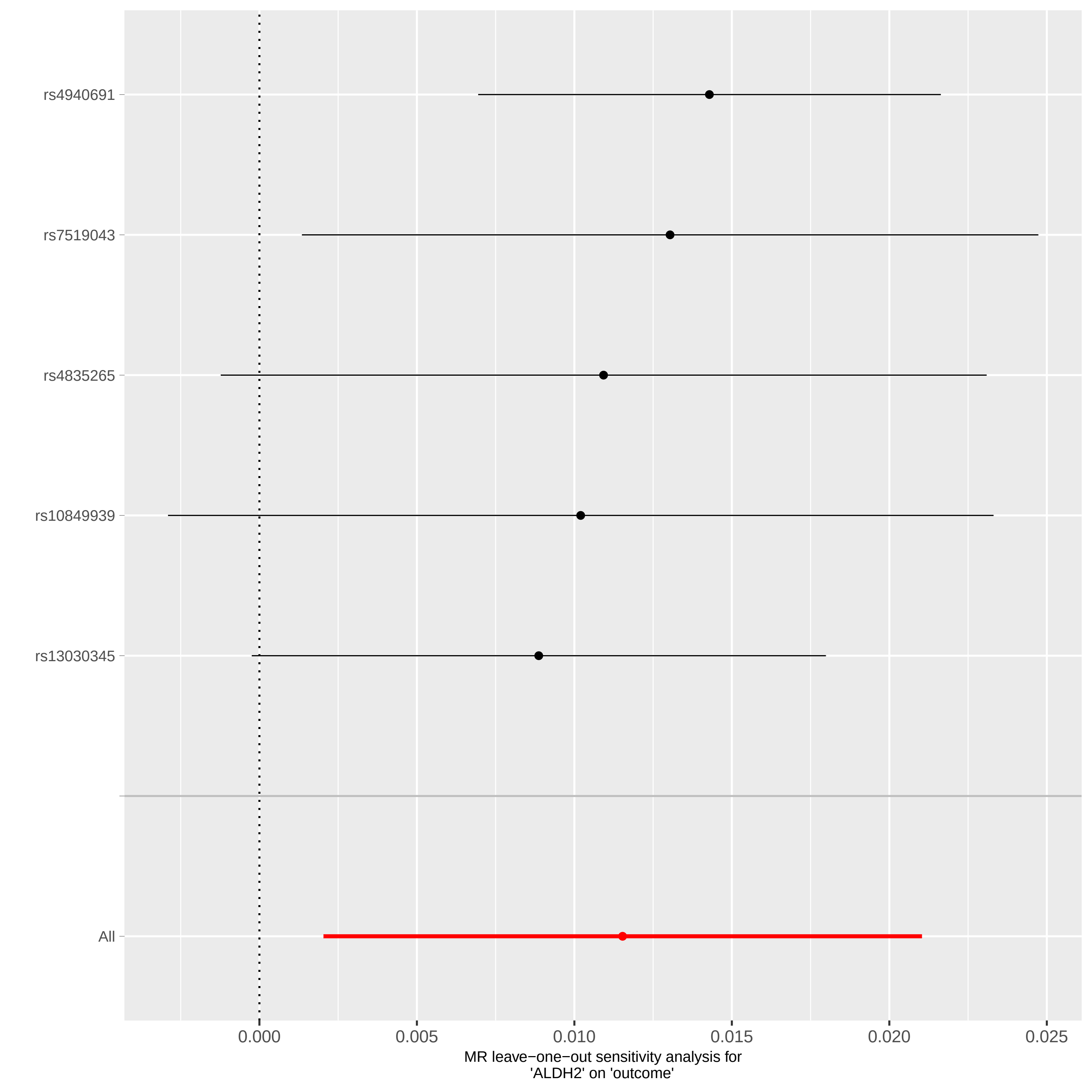

rs4940691
rs7519043
rs4835265
rs10849939
rs13030345
All
0.000
0.005
0.010
0.015
0.020
0.025
MR leave−one−out sensitivity analysis for
'ALDH2' on 'outcome'

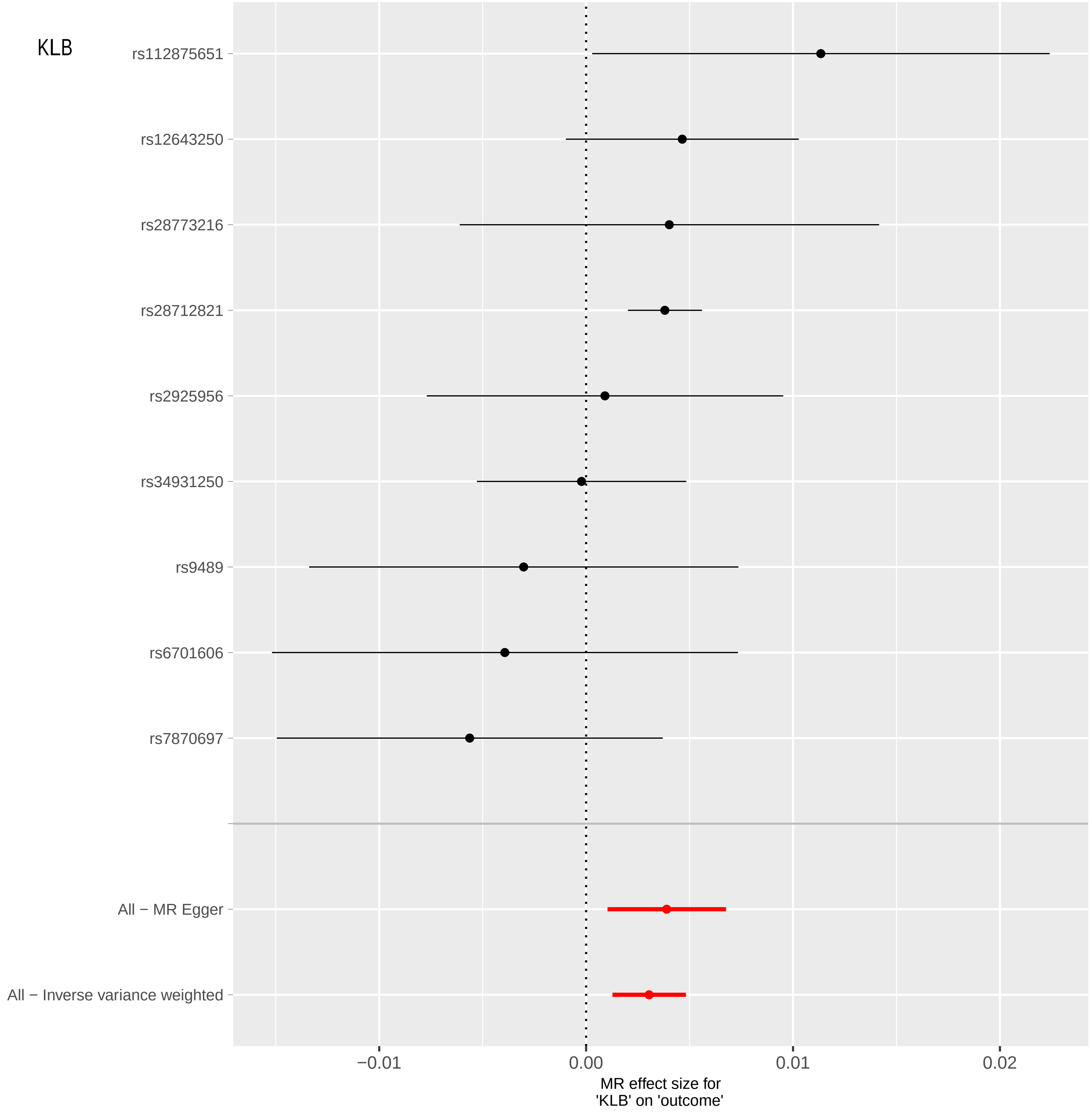
KLB
rs112875651
rs12643250
rs28773216
rs28712821
rs2925956
rs34931250
rs9489
rs6701606
rs7870697
All − MR Egger
All − Inverse variance weighted
−0.01
0.00
0.01
0.02
MR effect size for
'KLB' on 'outcome'

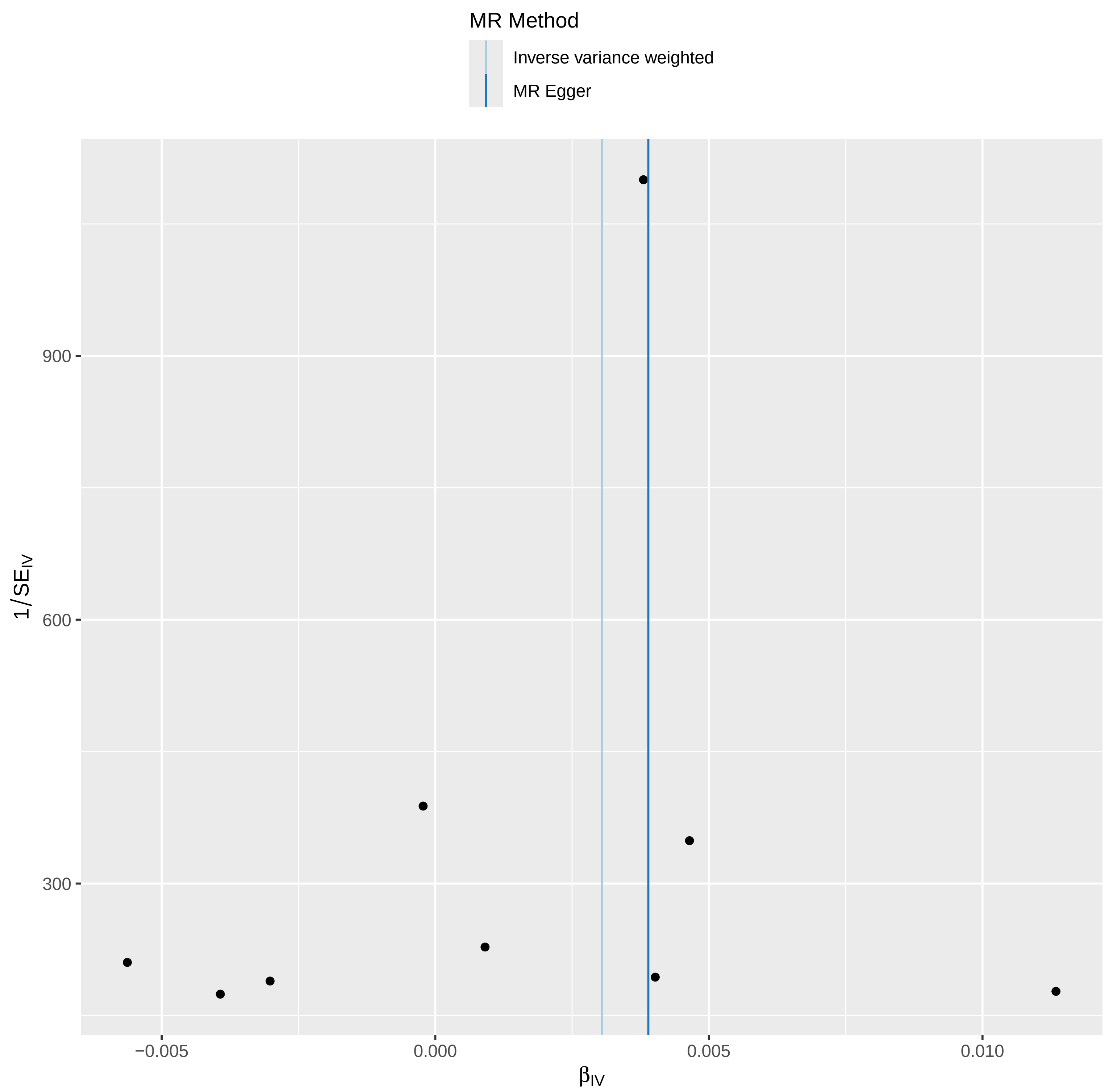
MR Method
Inverse variance weighted
MR Egger
1/SE_IV
900
600
300
−0.005
0.000
0.005
0.010
β_IV

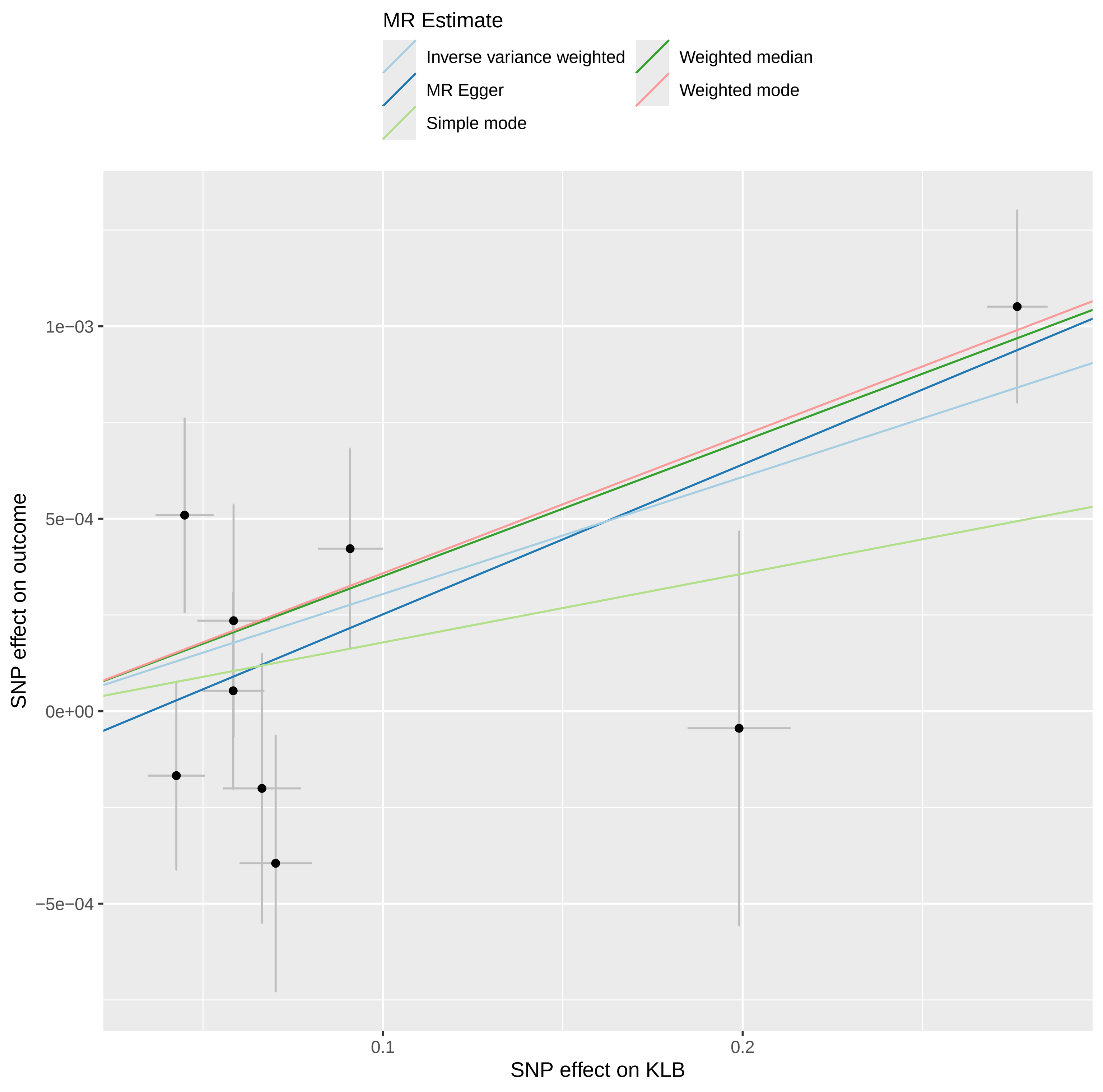
MR Estimate
Inverse variance weighted
MR Egger
Simple mode
Weighted median
Weighted mode
1e−03
5e−04
0e+00
−5e−04
SNP effect on outcome
0.1
0.2
SNP effect on KLB

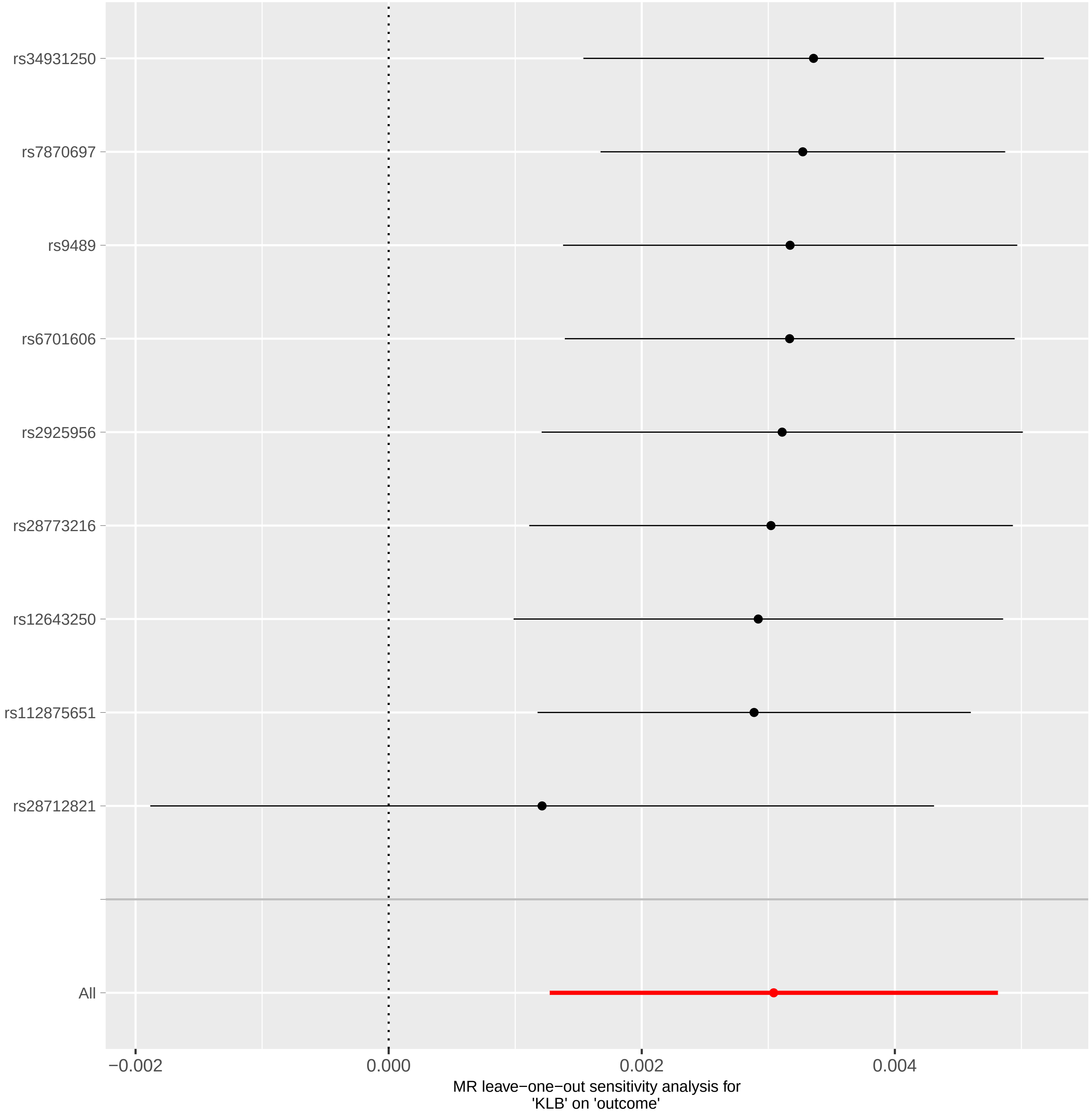

rs34931250
rs7870697
rs9489
rs6701606
rs2925956
rs28773216
rs12643250
rs112875651
rs28712821
All
−0.002
0.000
0.002
0.004
MR leave−one−out sensitivity analysis for
'KLB' on 'outcome'

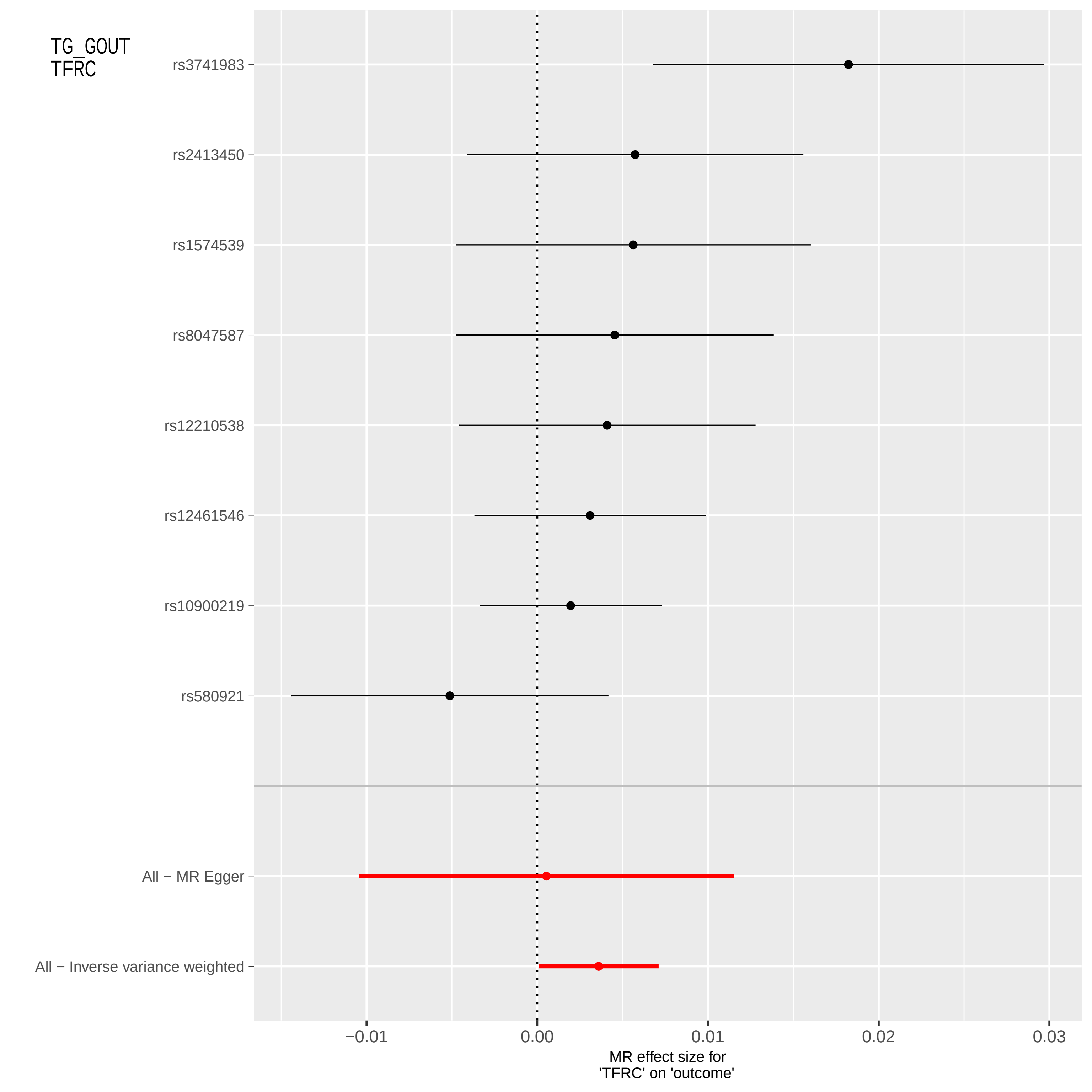

TG_GOUT
TFRC
rs3741983
rs2413450
rs1574539
rs8047587
rs12210538
rs12461546
rs10900219
rs580921
All − MR Egger
All − Inverse variance weighted
−0.01
0.00
0.01
0.02
0.03
MR effect size for
'TFRC' on 'outcome'

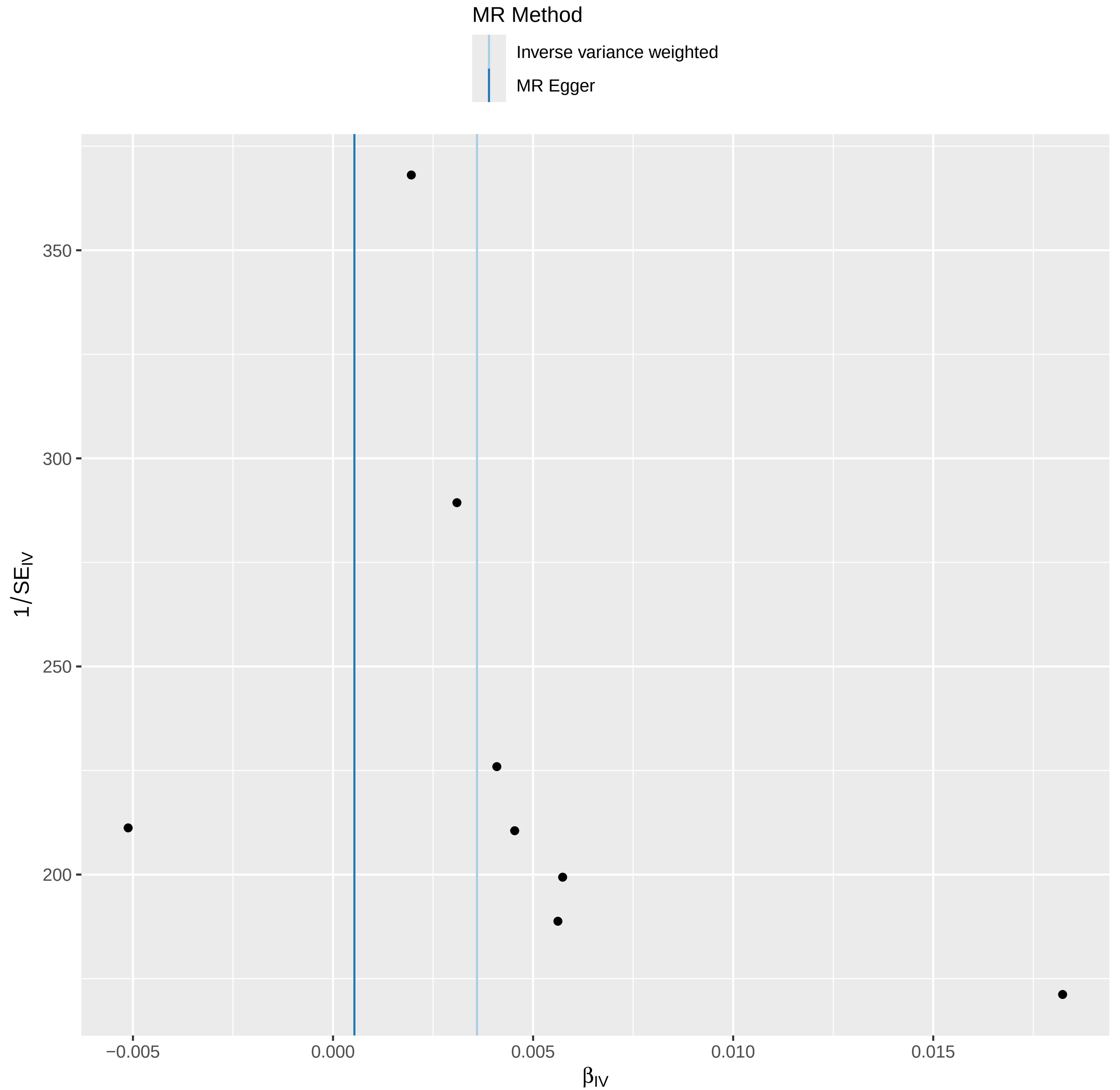

MR Method
Inverse variance weighted
MR Egger
350
300
250
200
1/SE_IV
−0.005
0.000
0.005
0.010
0.015
β_IV

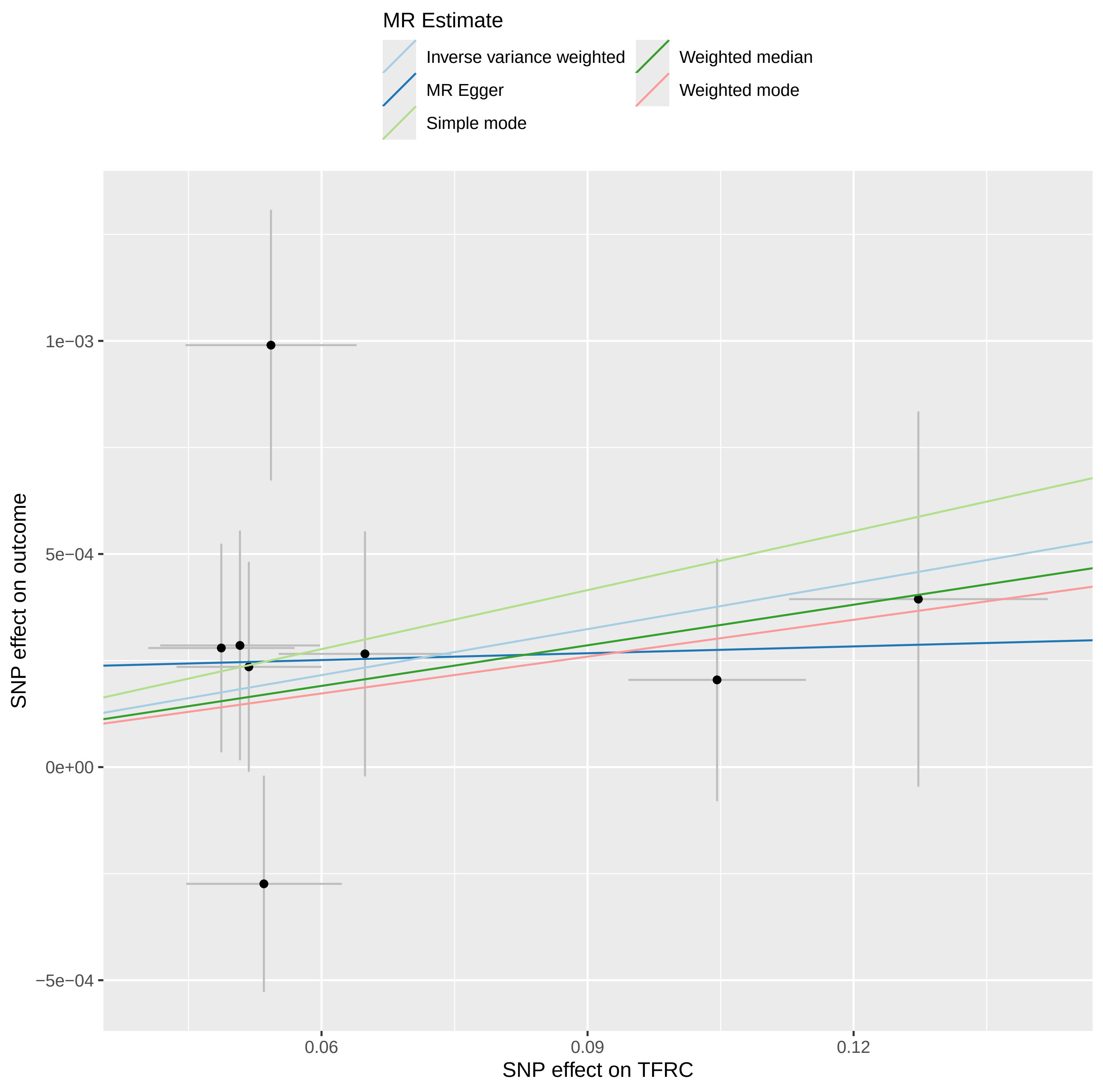

MR Estimate
Inverse variance weighted
MR Egger
Simple mode
Weighted median
Weighted mode
1e−03
5e−04
0e+00
−5e−04
SNP effect on outcome
0.06
0.09
0.12
SNP effect on TFRC

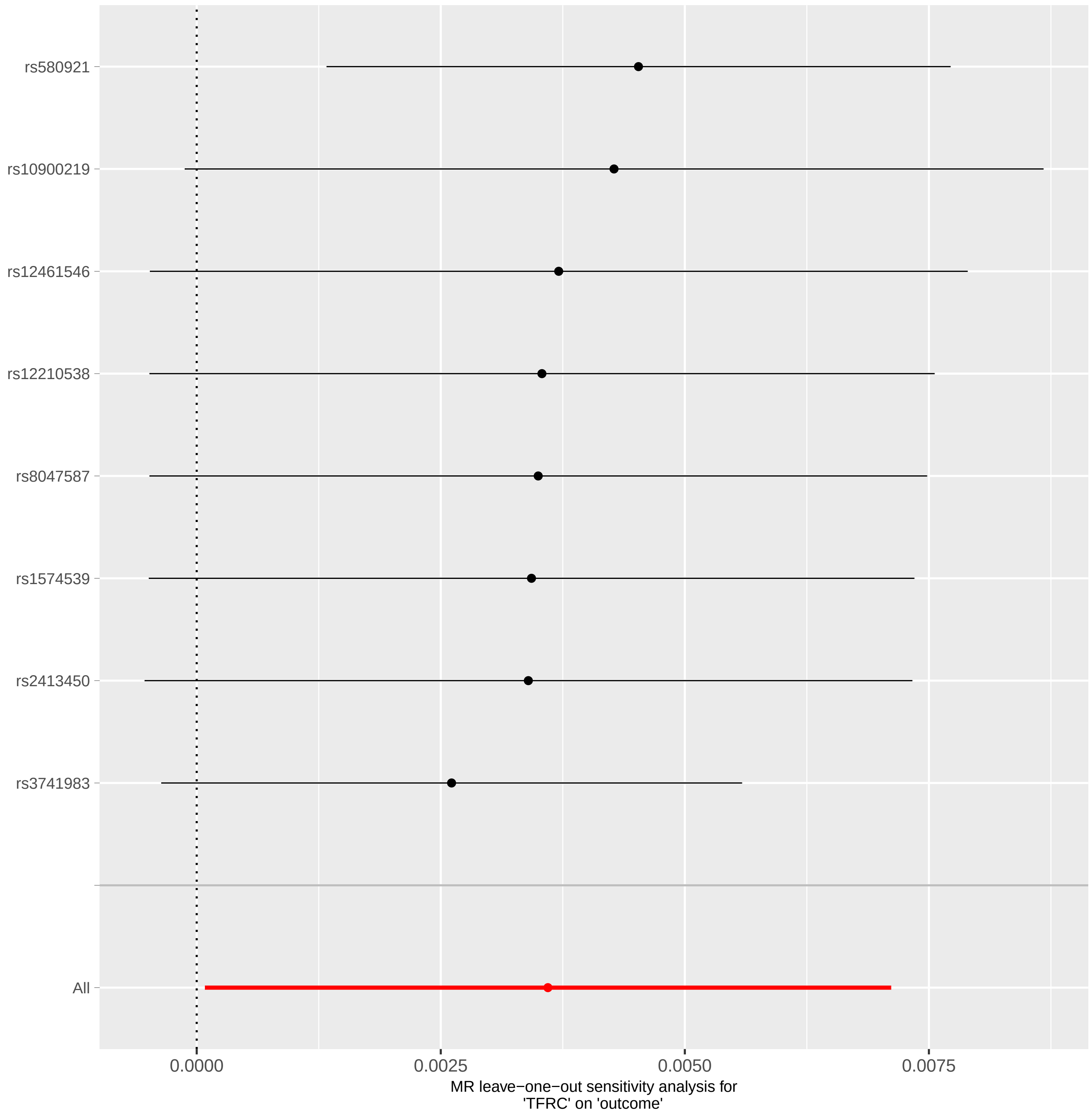
rs580921
rs10900219
rs12461546
rs12210538
rs8047587
rs1574539
rs2413450
rs3741983
All
0.0000
0.0025
0.0050
0.0075
MR leave−one−out sensitivity analysis for
'TFRC' on 'outcome'

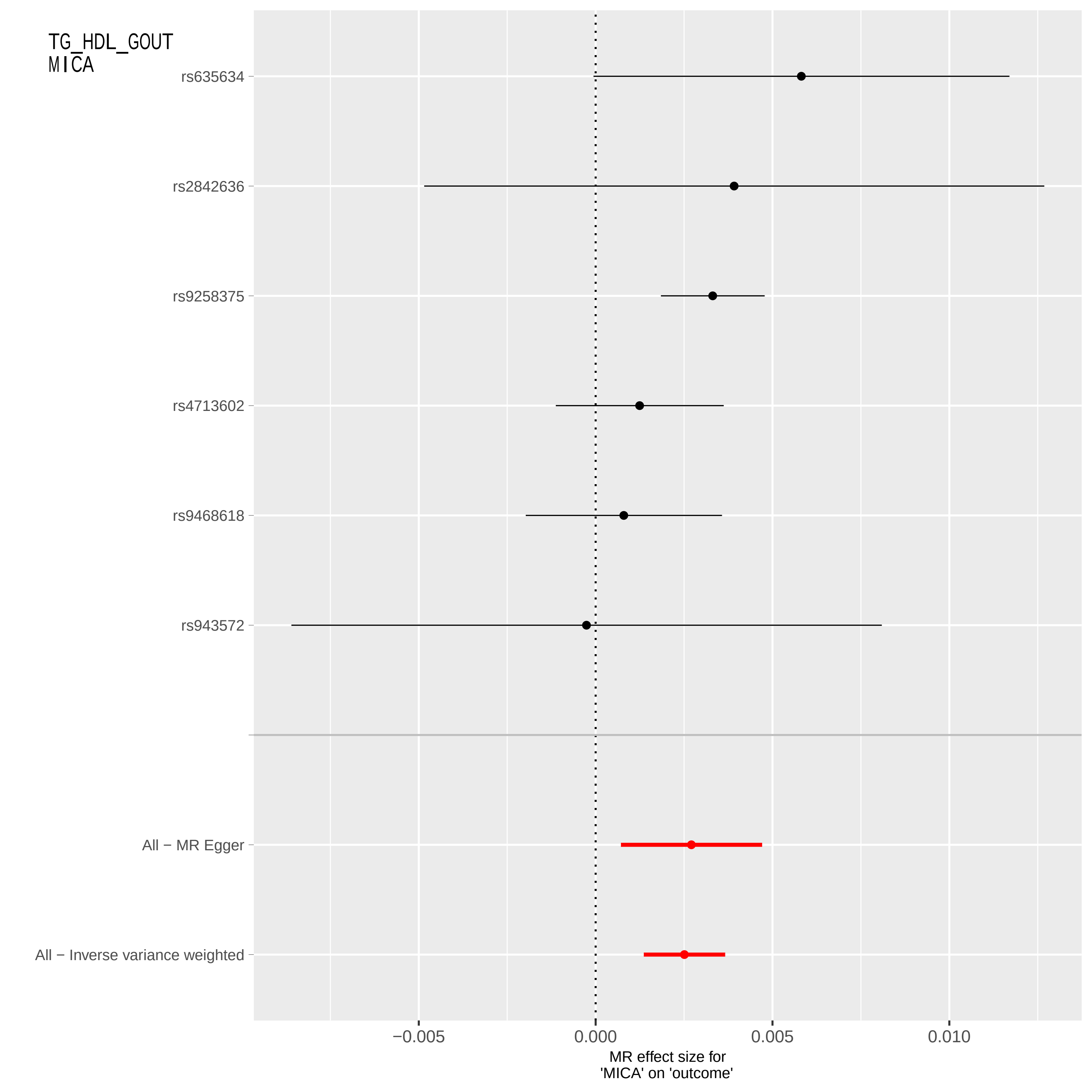
TG_HDL_GOUT
MICA
rs635634
rs2842636
rs9258375
rs4713602
rs9468618
rs943572
All − MR Egger
All − Inverse variance weighted
−0.005
0.000
0.005
0.010
MR effect size for
'MICA' on 'outcome'

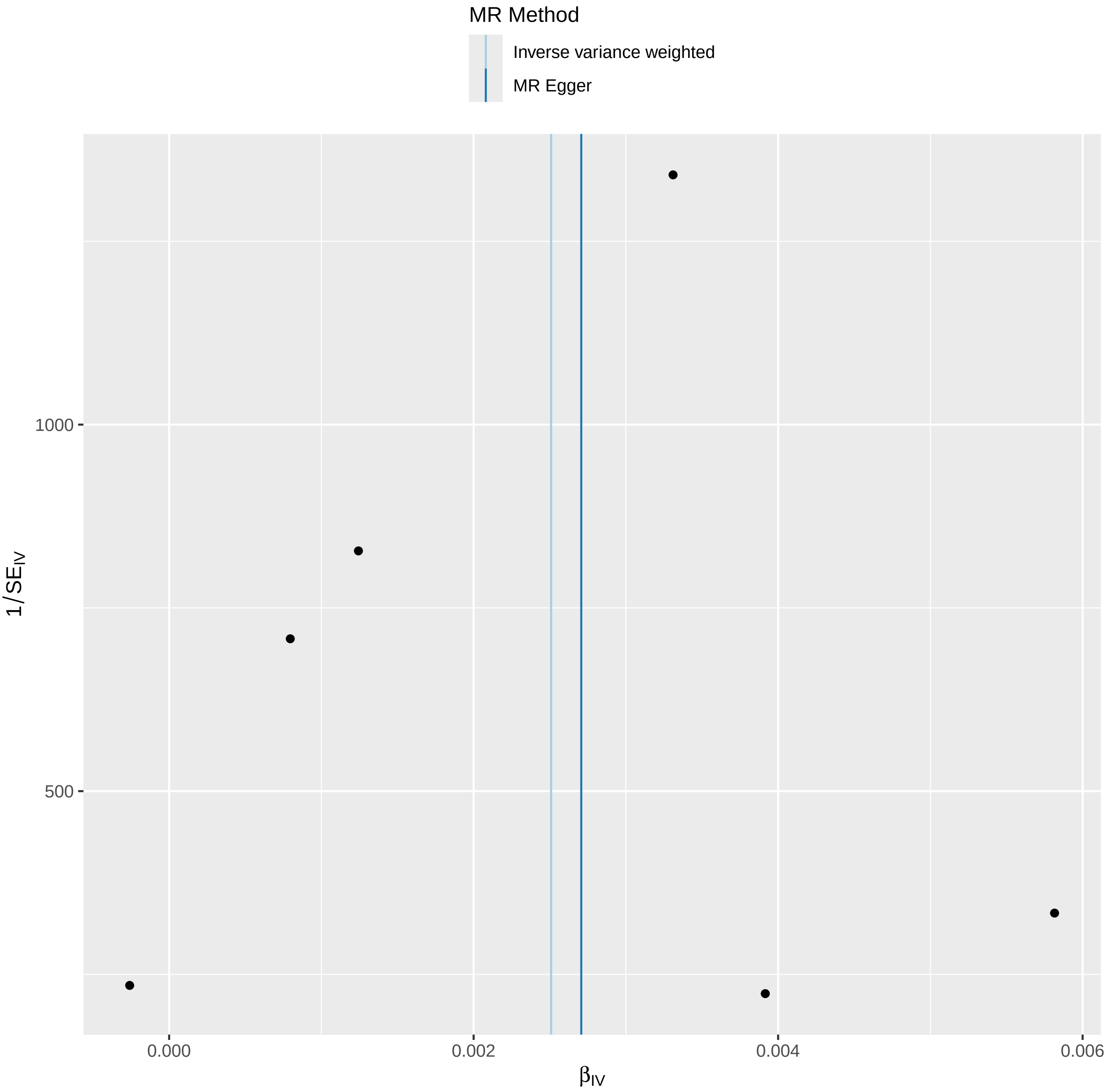

MR Method
Inverse variance weighted
MR Egger
1000
500
1/SE_IV
0.000
0.002
0.004
0.006
β_IV

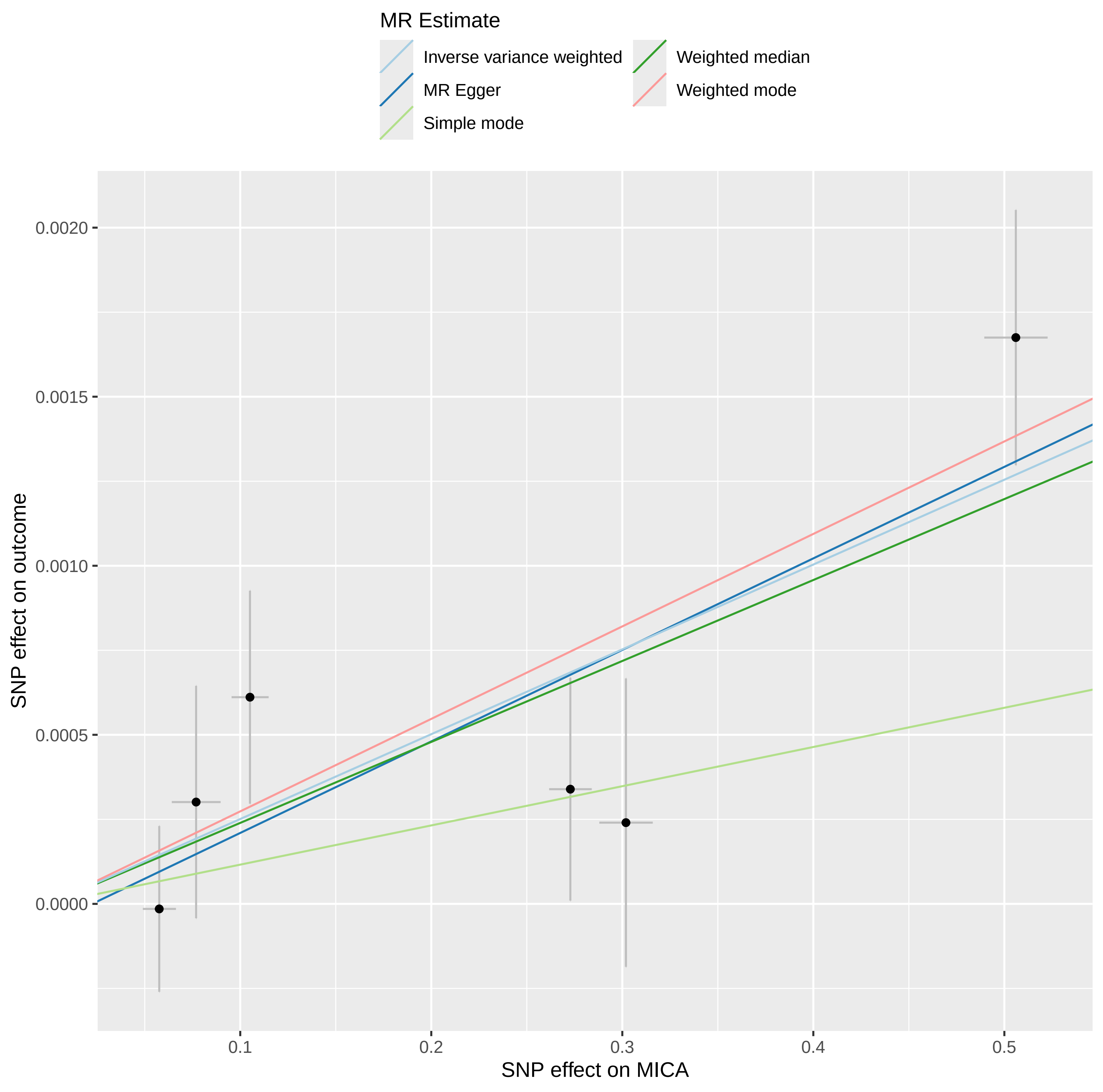
MR Estimate
Inverse variance weighted
MR Egger
Simple mode
Weighted median
Weighted mode
0.0020
0.0015
0.0010
0.0005
0.0000
SNP effect on outcome
0.1
0.2
0.3
0.4
0.5
SNP effect on MICA

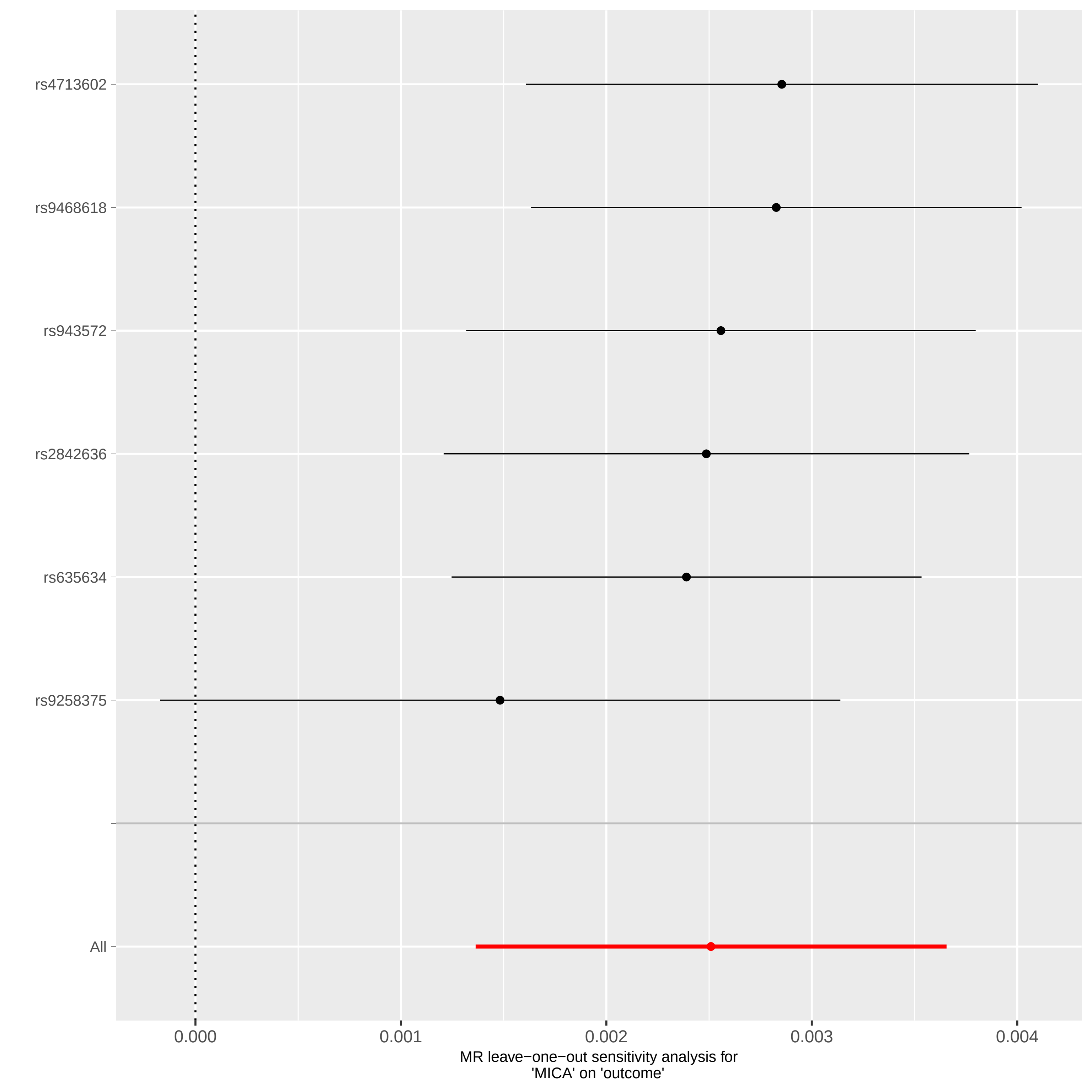
rs4713602
rs9468618
rs943572
rs2842636
rs635634
rs9258375
All
0.000
0.001
0.002
0.003
0.004
MR leave−one−out sensitivity analysis for
'MICA' on 'outcome'

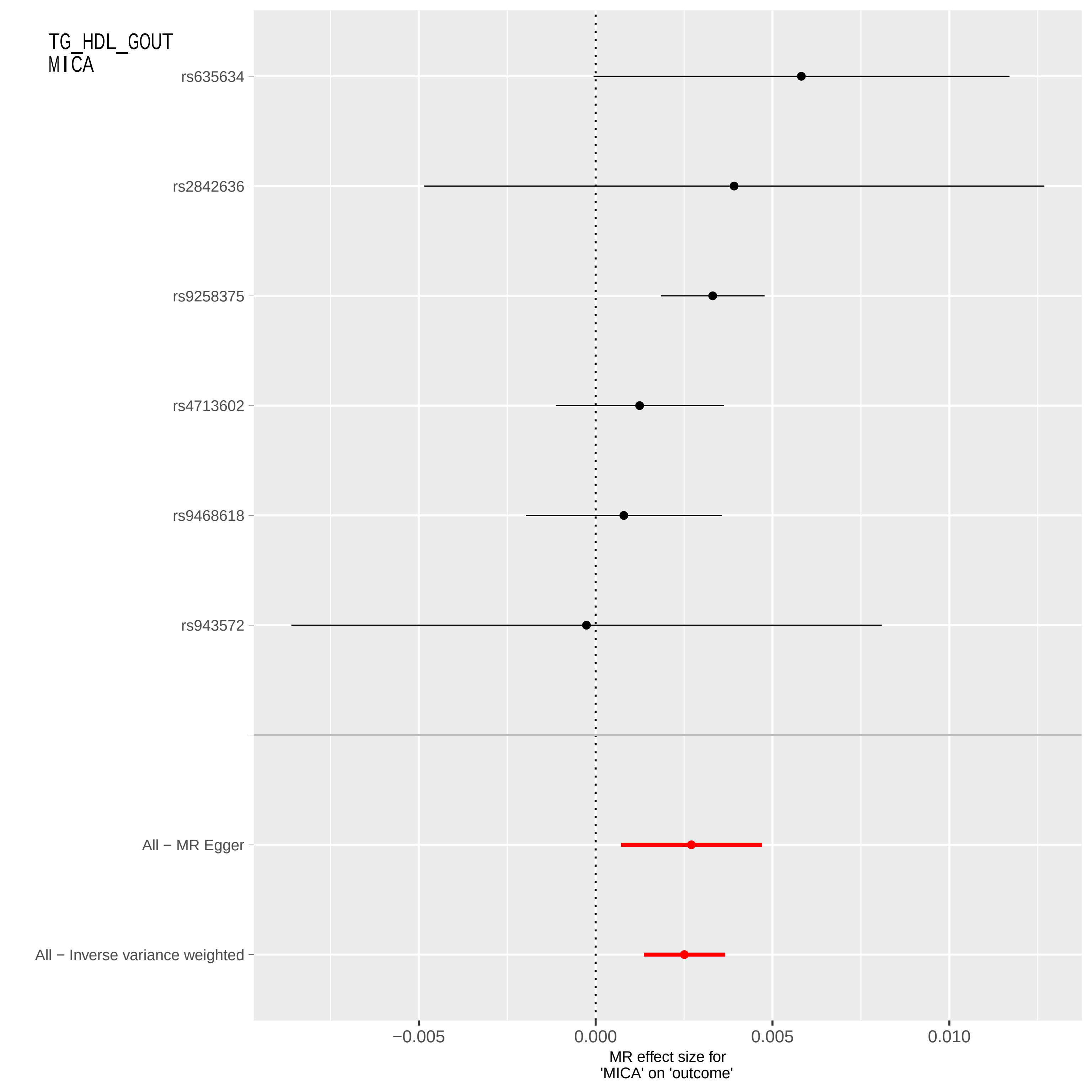

TG_HDL_GOUT
MICA
rs635634
rs2842636
rs9258375
rs4713602
rs9468618
rs943572
All − MR Egger
All − Inverse variance weighted
−0.005
0.000
0.005
0.010
MR effect size for
'MICA' on 'outcome'

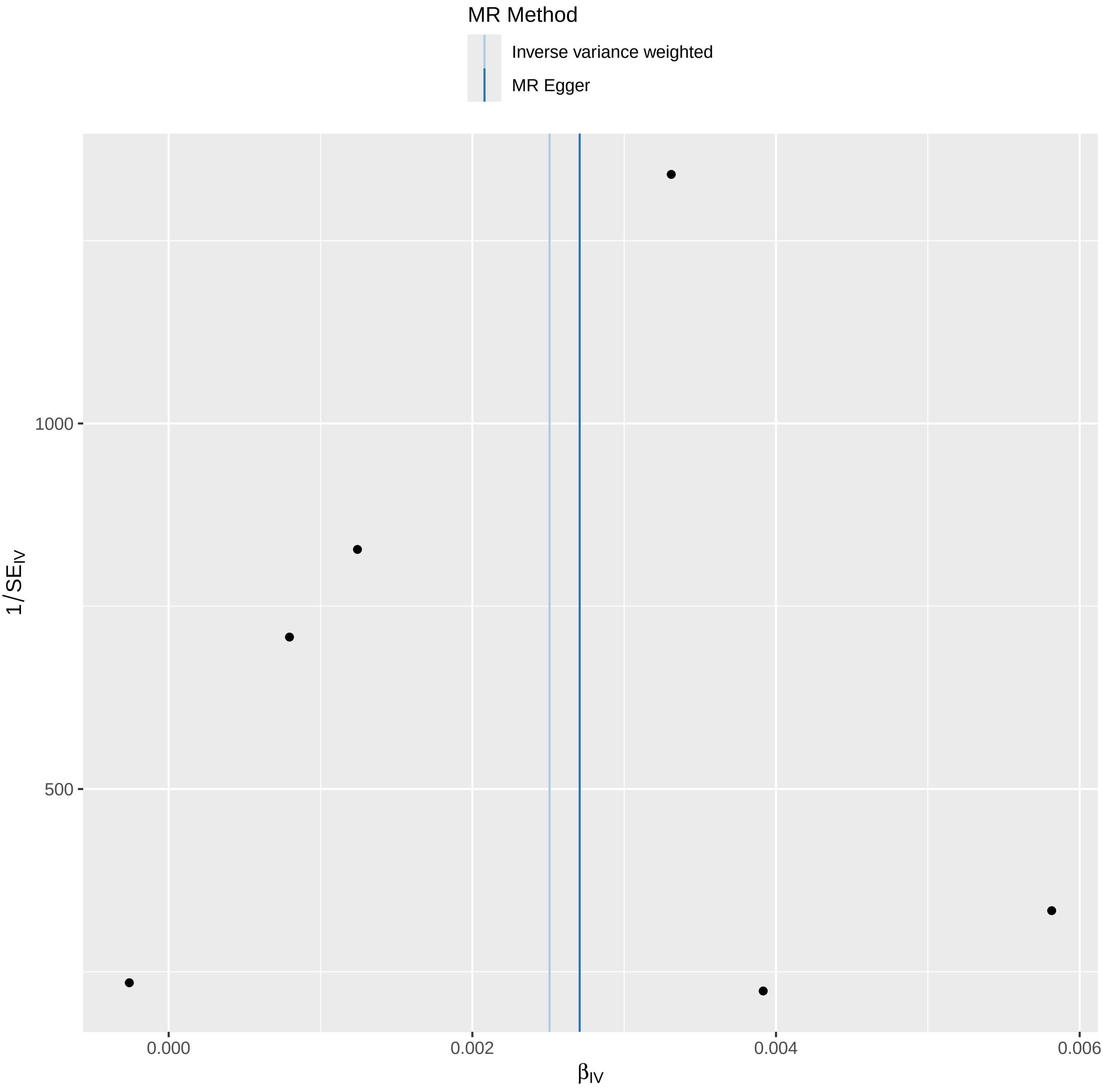
MR Method
Inverse variance weighted
MR Egger
1000
500
1/SE$_{IV}$
0.000
0.002
0.004
0.006
β_{IV}

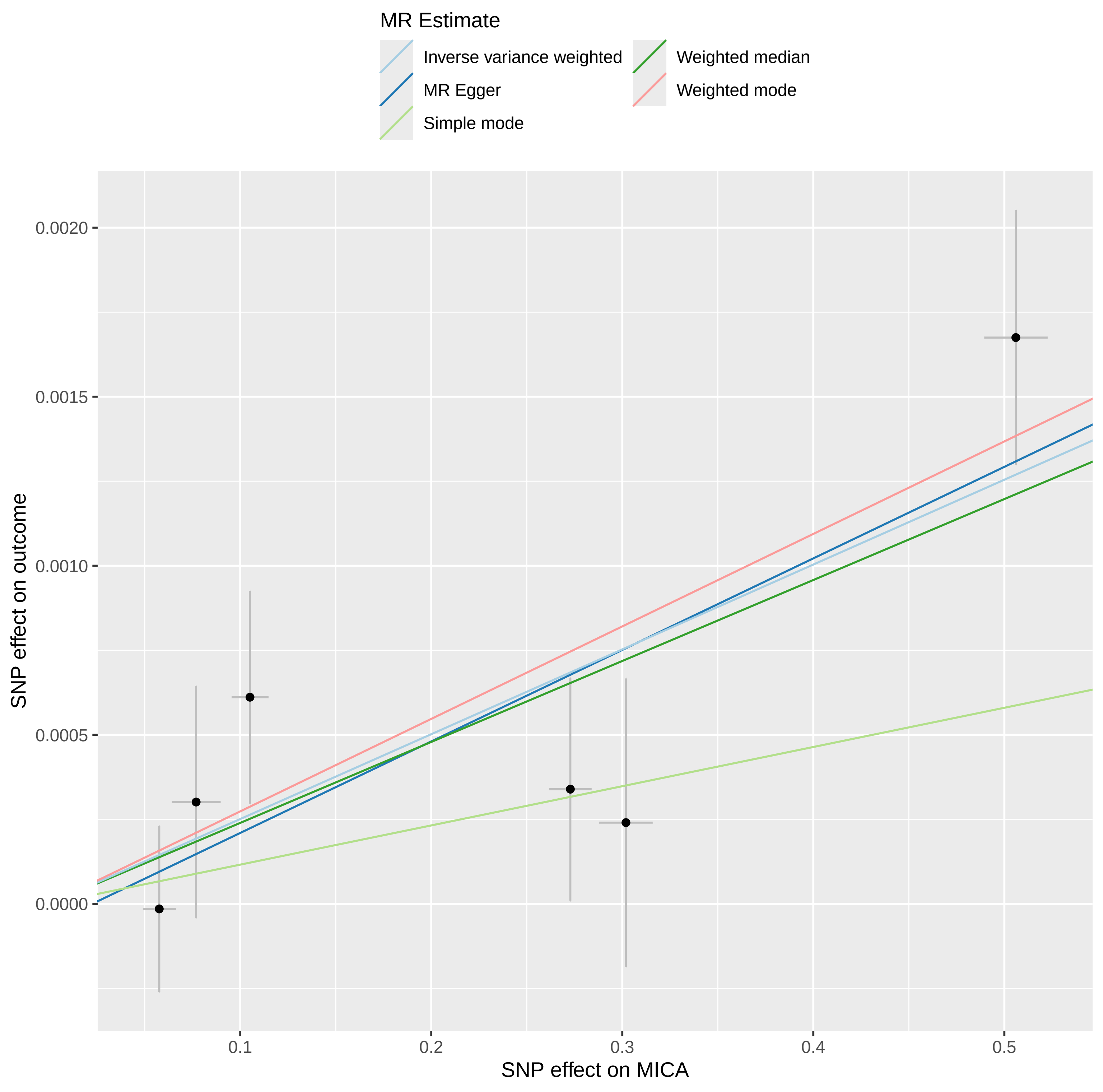
MR Estimate
Inverse variance weighted
MR Egger
Simple mode
Weighted median
Weighted mode
0.0020
0.0015
0.0010
0.0005
0.0000
SNP effect on outcome
0.1
0.2
0.3
0.4
0.5
SNP effect on MICA

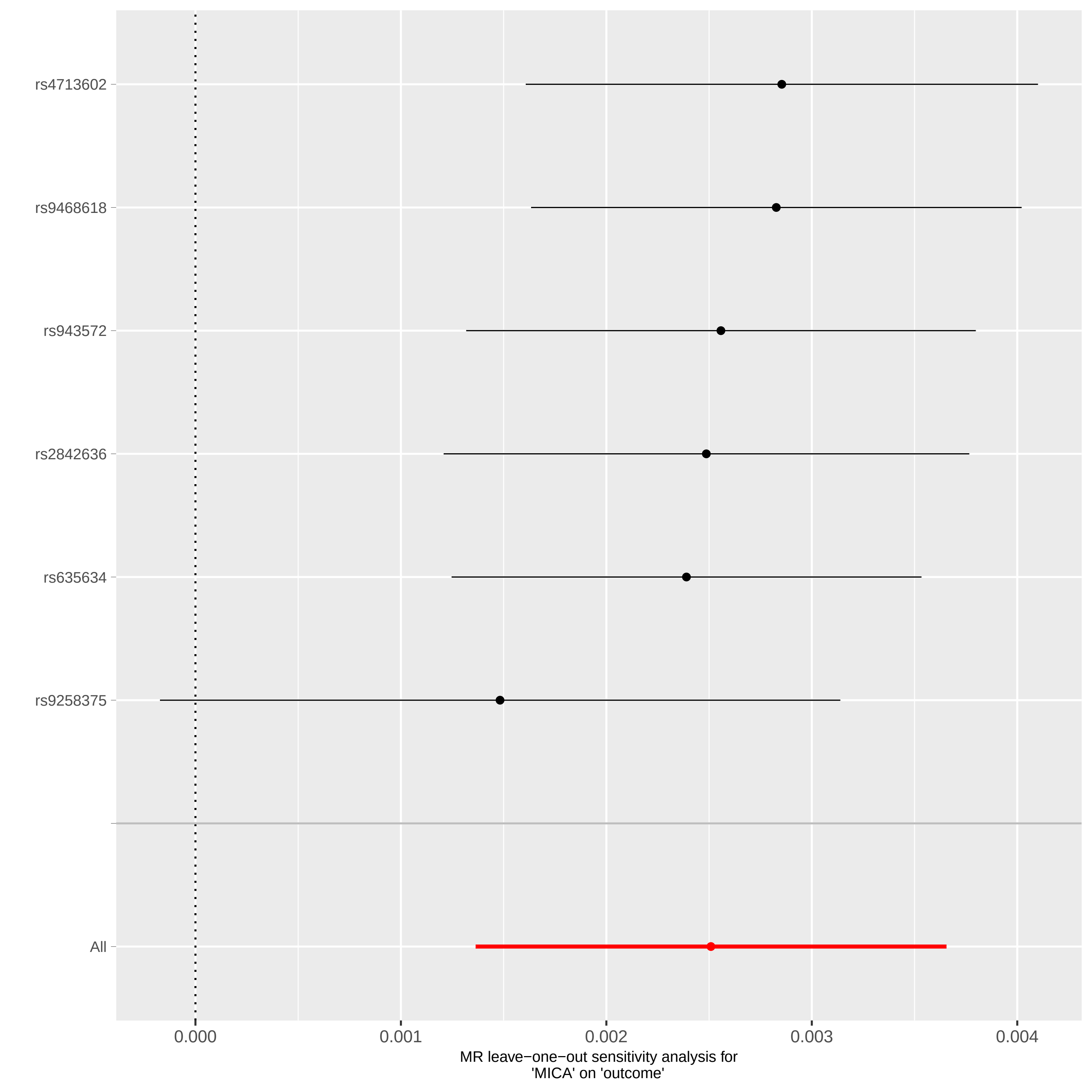

rs4713602
rs9468618
rs943572
rs2842636
rs635634
rs9258375
All
0.000
0.001
0.002
0.003
0.004
MR leave−one−out sensitivity analysis for
'MICA' on 'outcome'

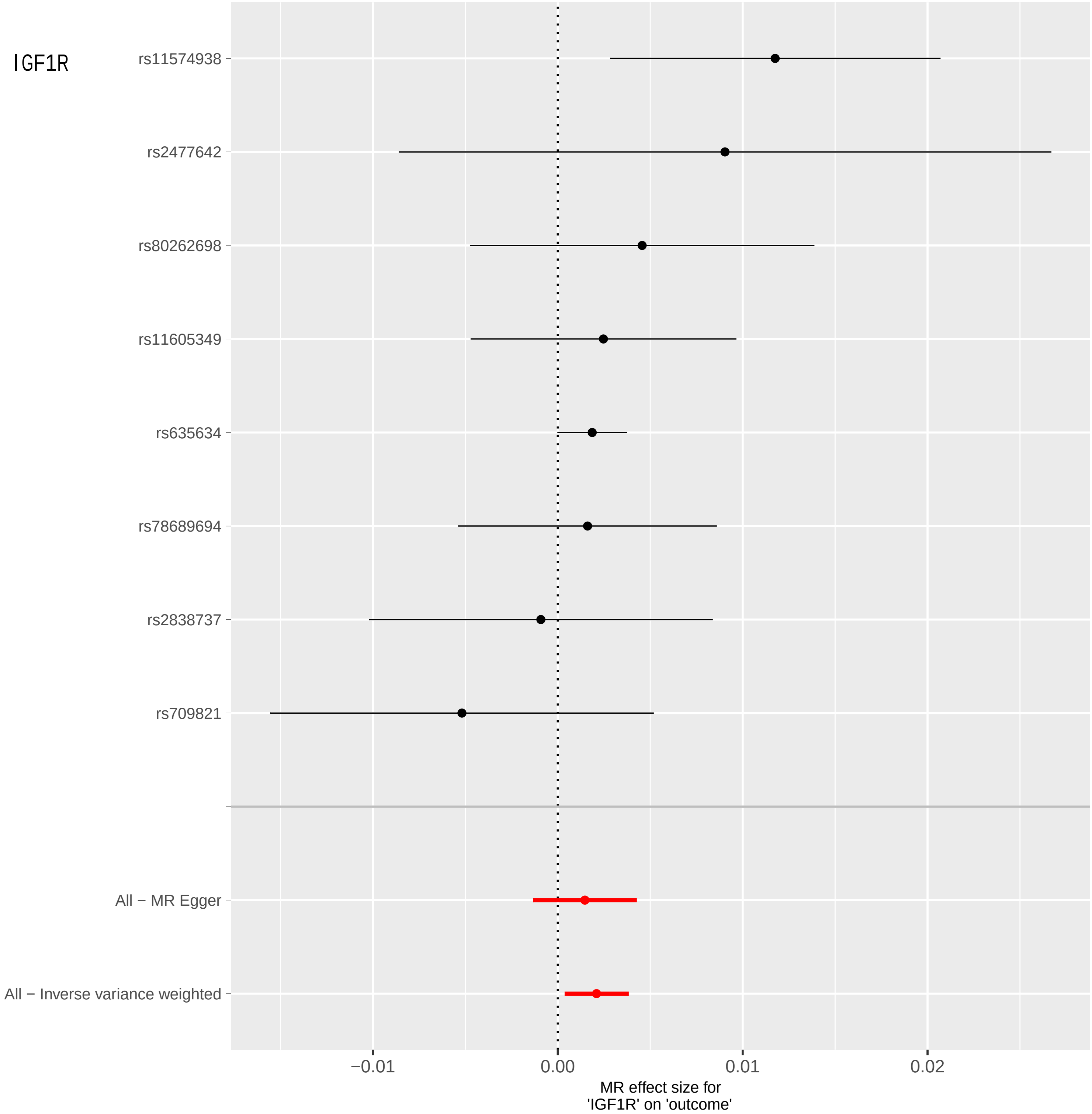
IGF1R
rs11574938
rs2477642
rs80262698
rs11605349
rs635634
rs78689694
rs2838737
rs709821
All − MR Egger
All − Inverse variance weighted
−0.01
0.00
0.01
0.02
MR effect size for
'IGF1R' on 'outcome'

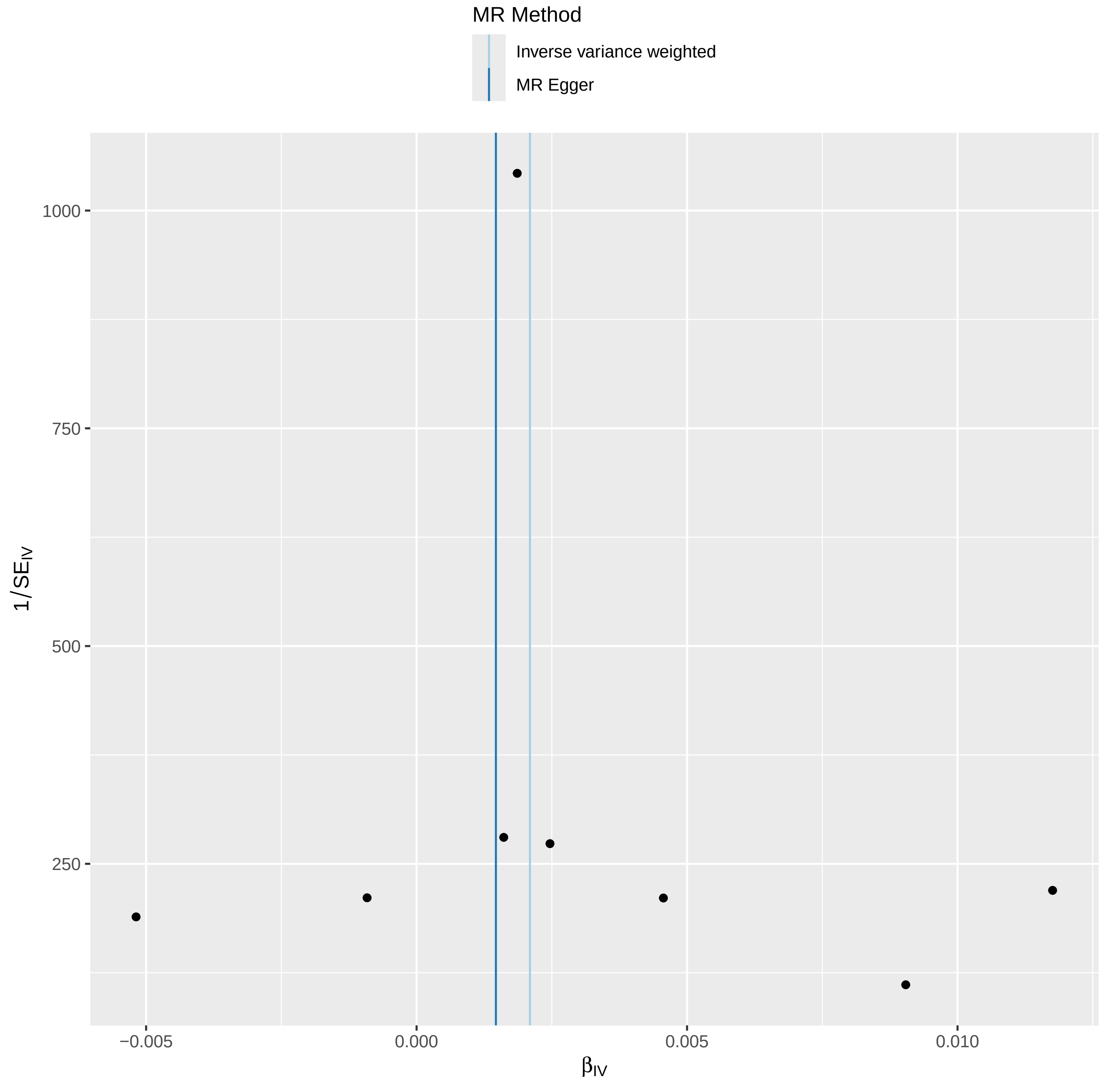

MR Method
Inverse variance weighted
MR Egger
1000
750
500
250
1/SE_IV
−0.005
0.000
0.005
0.010
β_IV

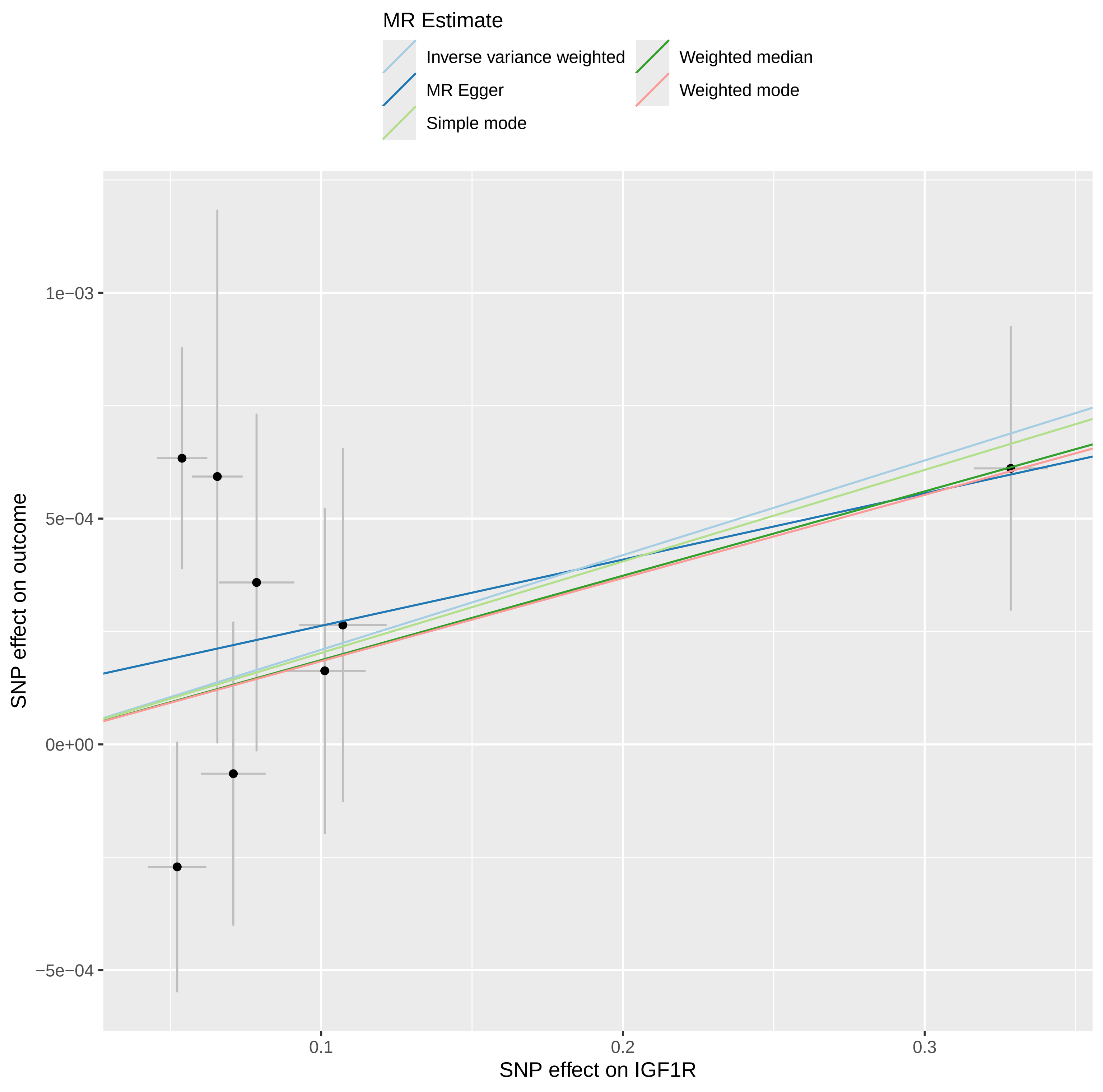
MR Estimate
Inverse variance weighted
MR Egger
Simple mode
Weighted median
Weighted mode
1e−03
5e−04
0e+00
−5e−04
0.1
0.2
0.3
SNP effect on outcome
SNP effect on IGF1R

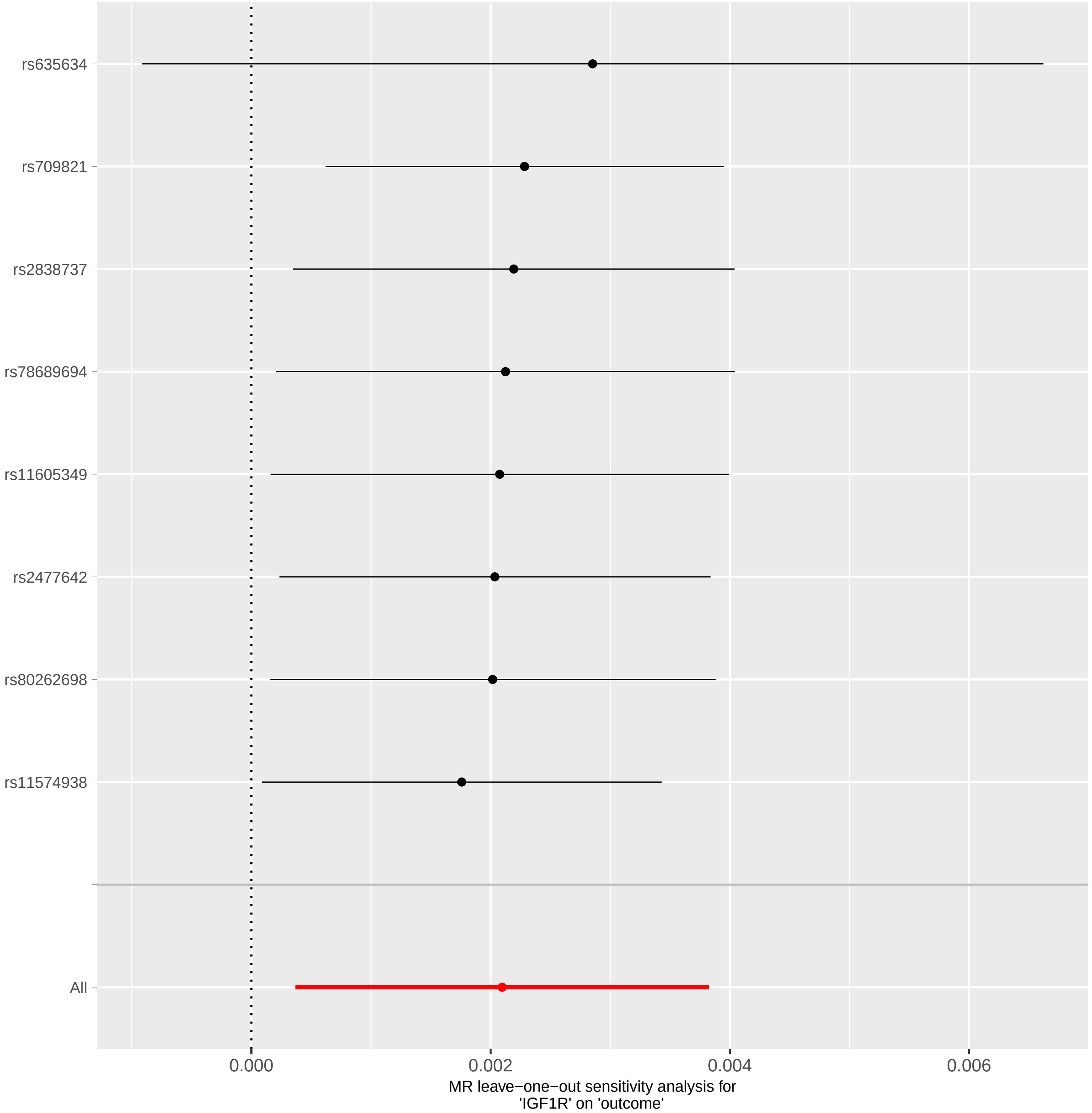
rs635634
rs709821
rs2838737
rs78689694
rs11605349
rs2477642
rs80262698
rs11574938
All
0.000
0.002
0.004
0.006
MR leave−one−out sensitivity analysis for
'IGF1R' on 'outcome'

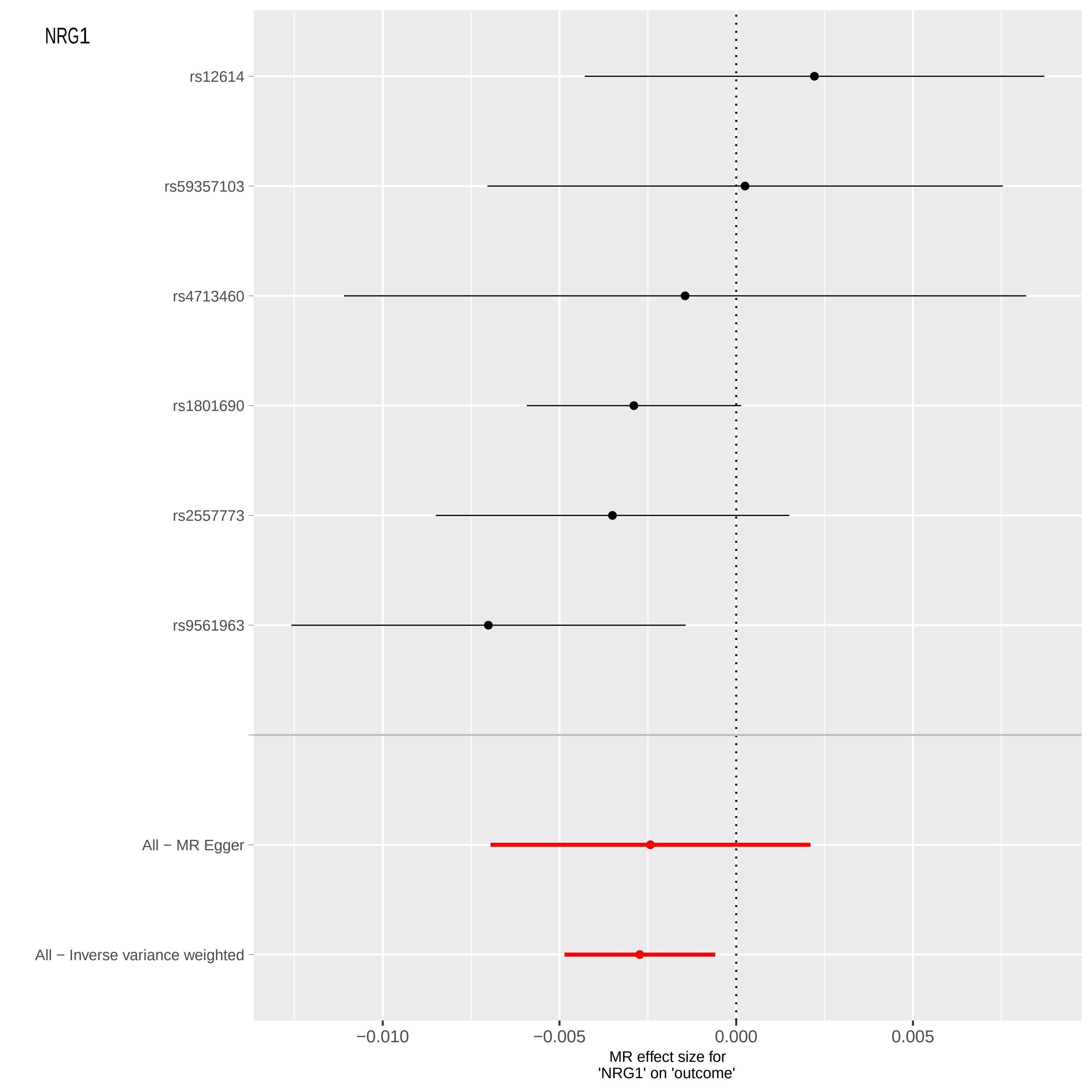

NRG1
rs12614
rs59357103
rs4713460
rs1801690
rs2557773
rs9561963
All − MR Egger
All − Inverse variance weighted
−0.010
−0.005
0.000
0.005
MR effect size for
'NRG1' on 'outcome'

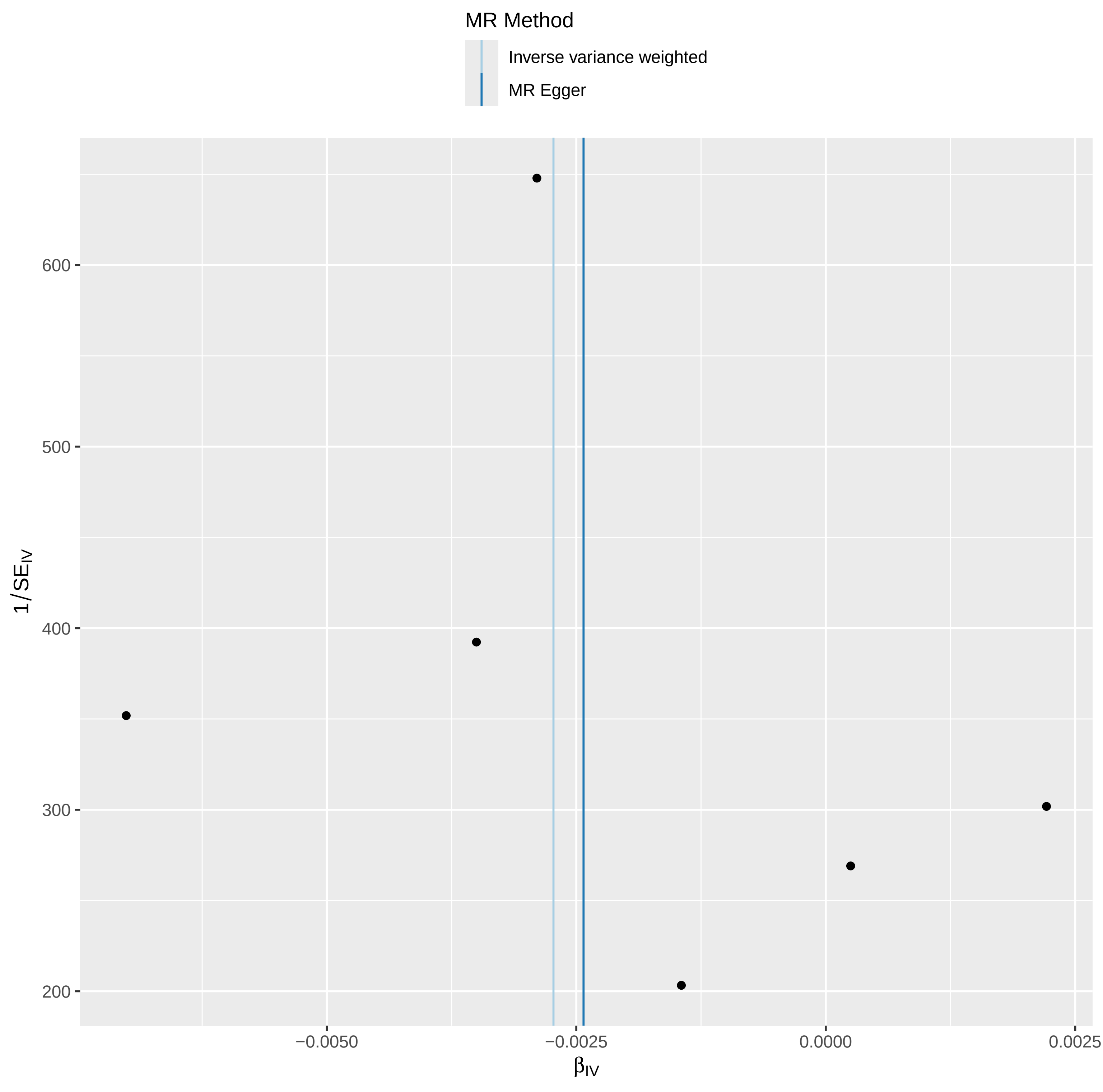
MR Method
Inverse variance weighted
MR Egger
600
500
400
300
200
1/SE_IV
−0.0050
−0.0025
0.0000
0.0025
β_IV

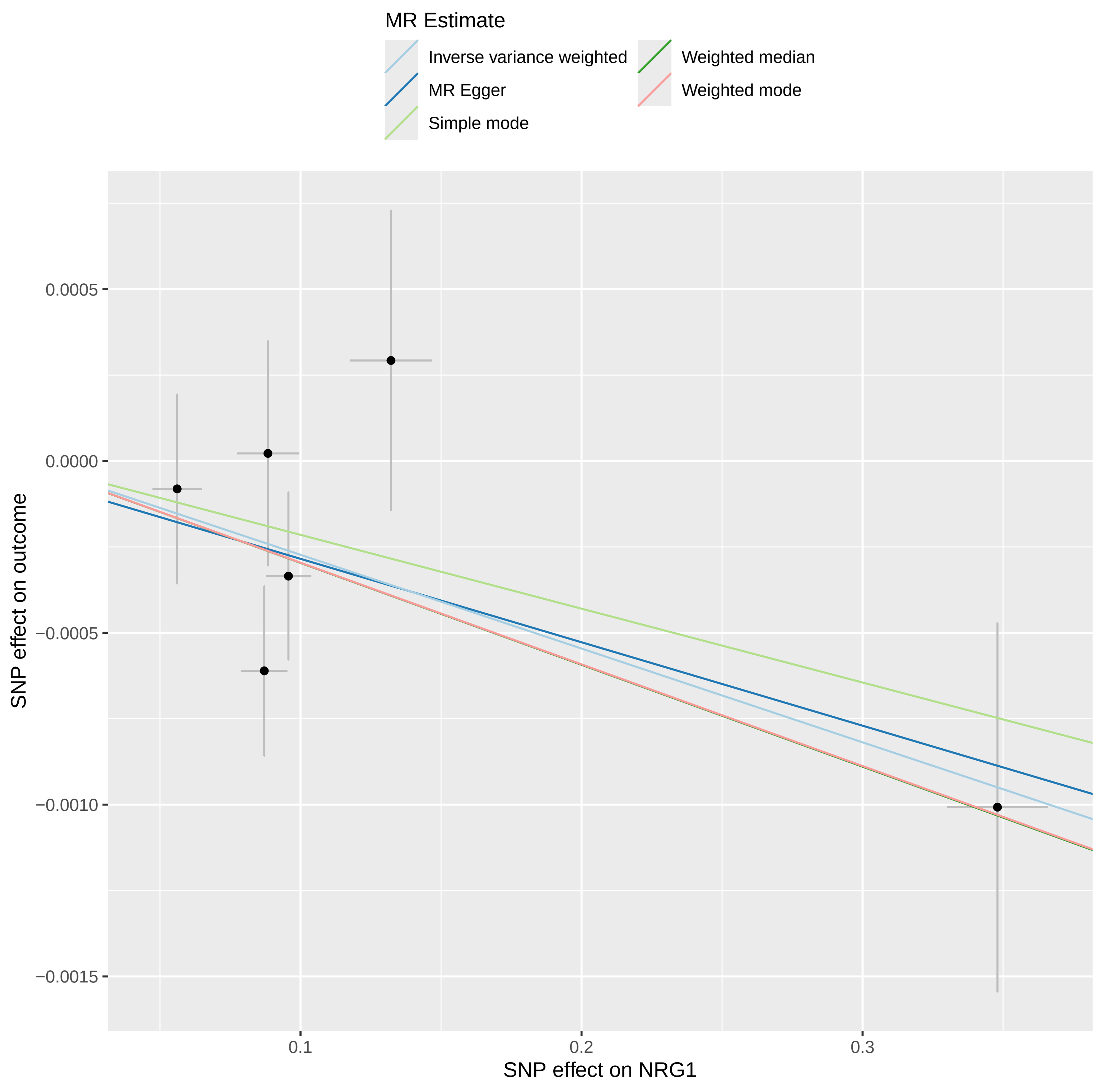
MR Estimate
Inverse variance weighted
MR Egger
Simple mode
Weighted median
Weighted mode
0.0005
0.0000
−0.0005
−0.0010
−0.0015
SNP effect on outcome
0.1
0.2
0.3
SNP effect on NRG1

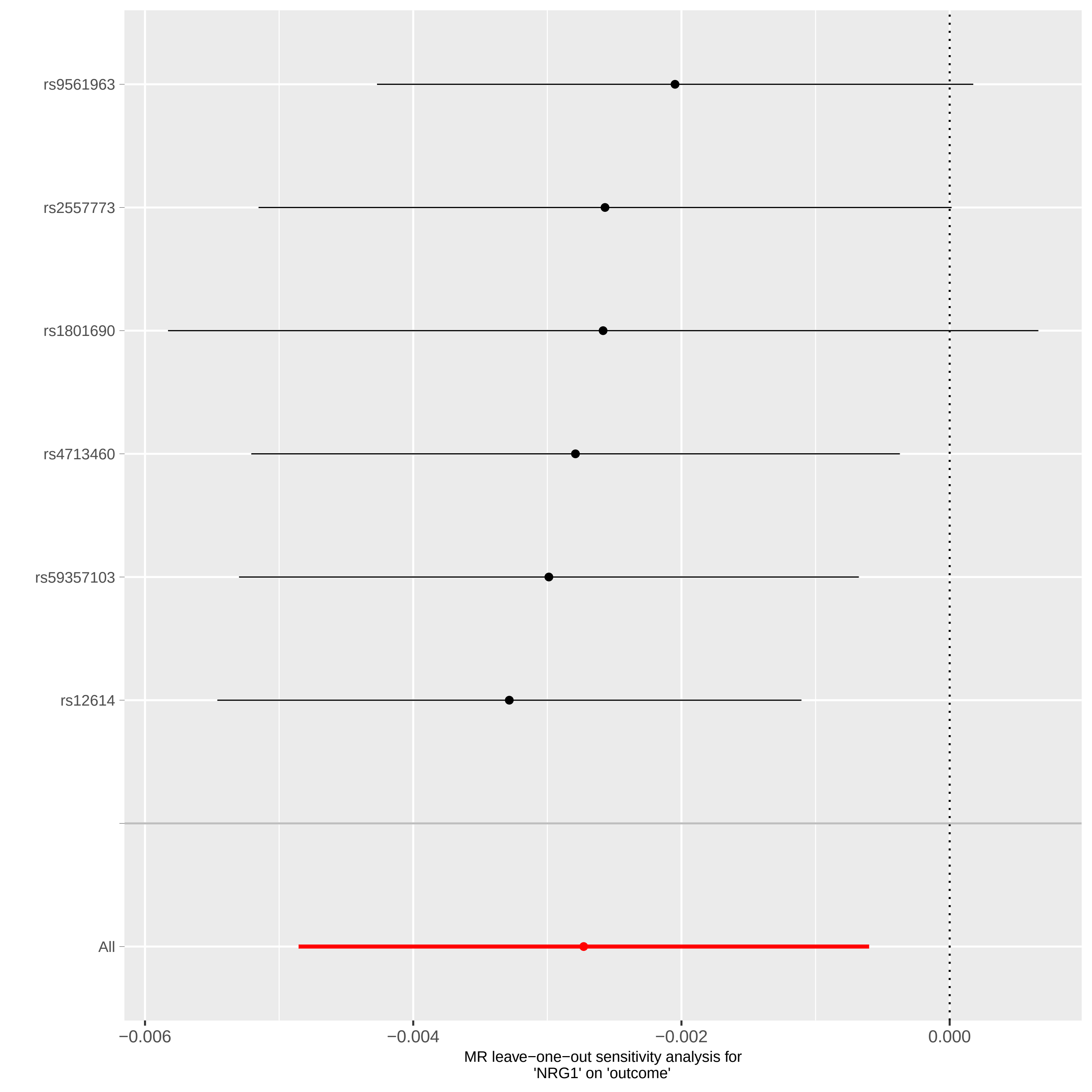

rs9561963
rs2557773
rs1801690
rs4713460
rs59357103
rs12614
All
−0.006
−0.004
−0.002
0.000
MR leave−one−out sensitivity analysis for
'NRG1' on 'outcome'

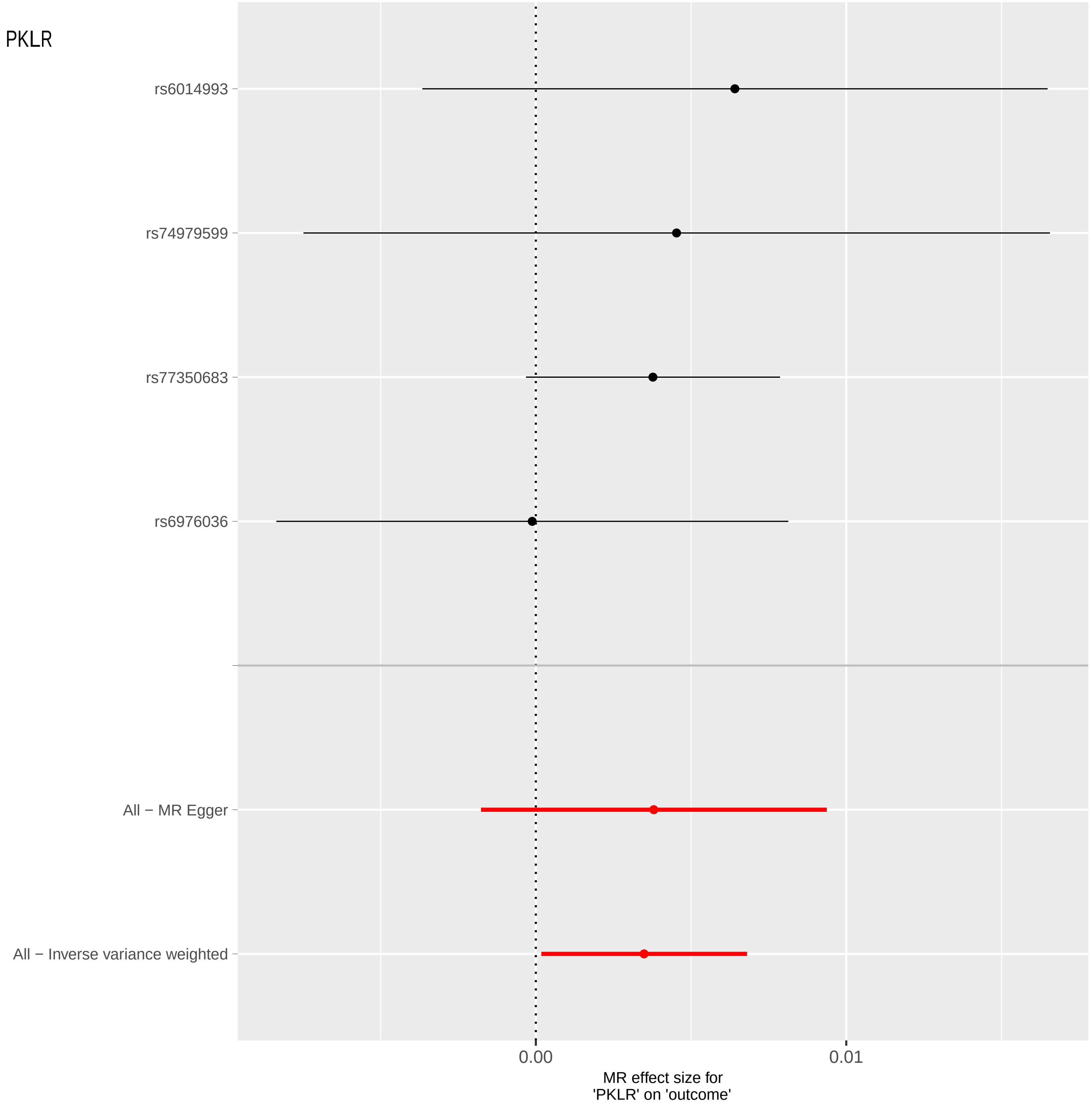
PKLR
rs6014993
rs74979599
rs77350683
rs6976036
All − MR Egger
All − Inverse variance weighted
0.00
0.01
MR effect size for
'PKLR' on 'outcome'

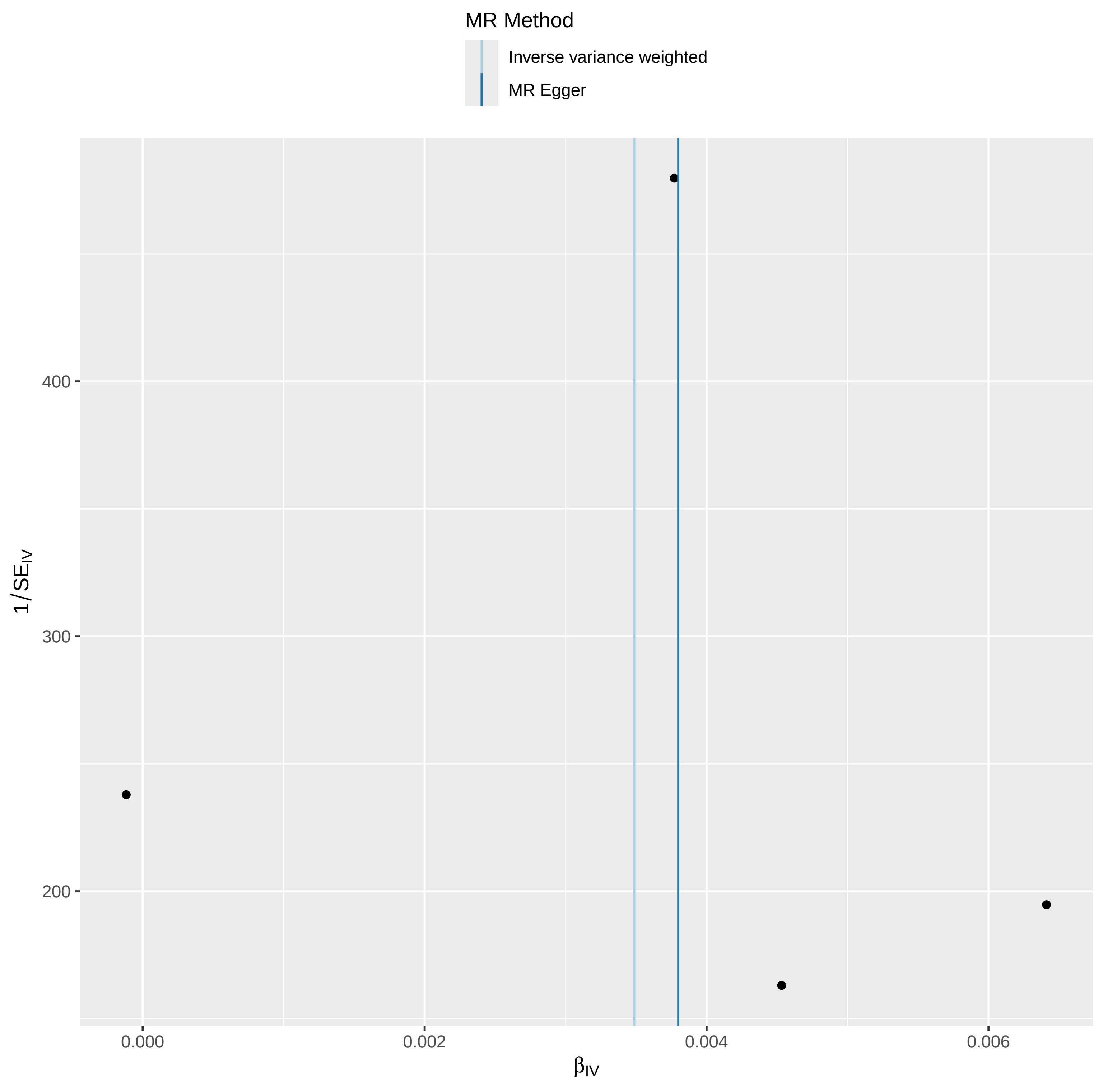

MR Method
Inverse variance weighted
MR Egger
400
300
200
0.000
0.002
0.004
0.006
1/SE_IV
β_IV

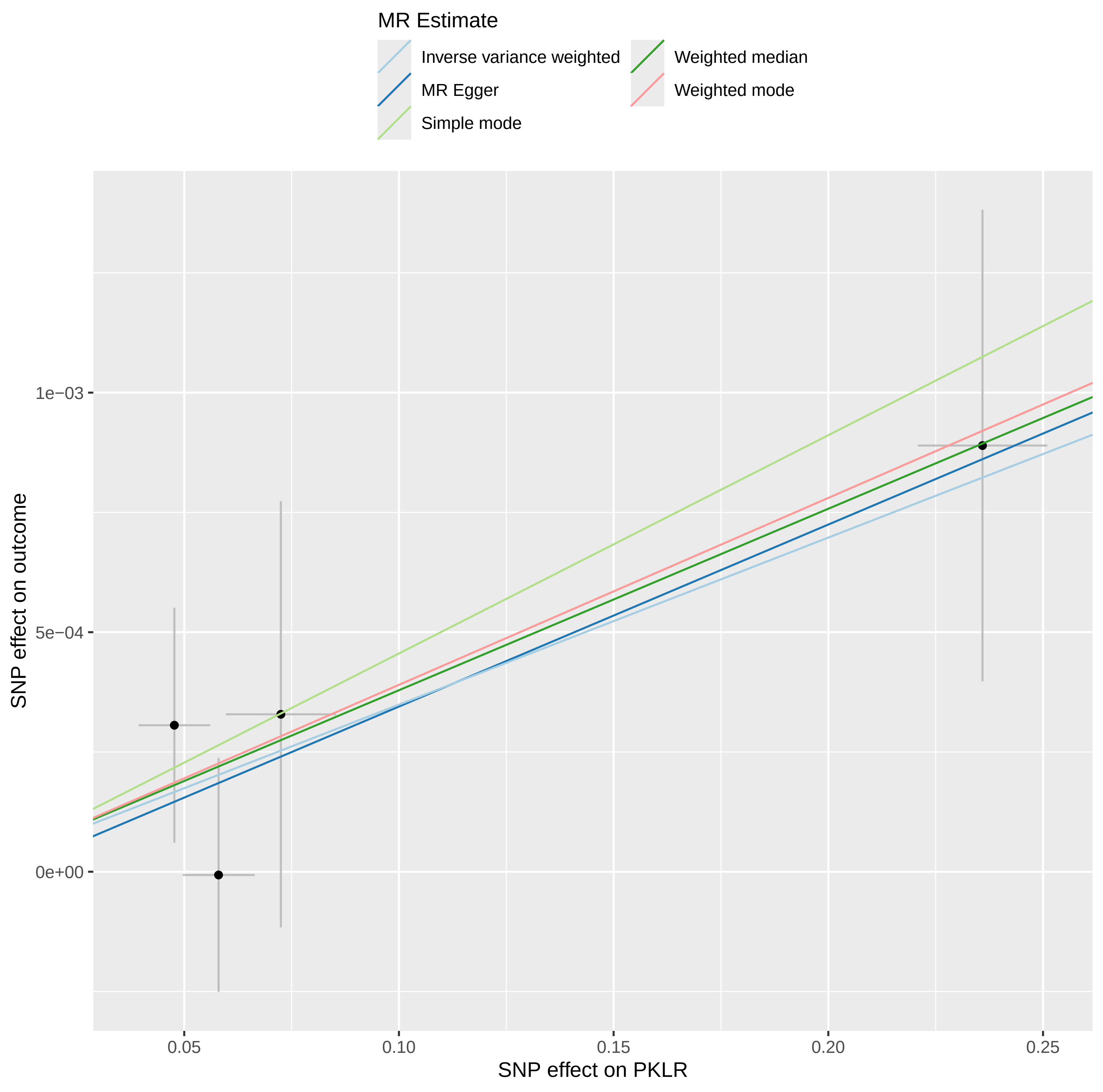

MR Estimate
Inverse variance weighted
MR Egger
Simple mode
Weighted median
Weighted mode
1e−03
5e−04
0e+00
SNP effect on outcome
0.05
0.10
0.15
0.20
0.25
SNP effect on PKLR

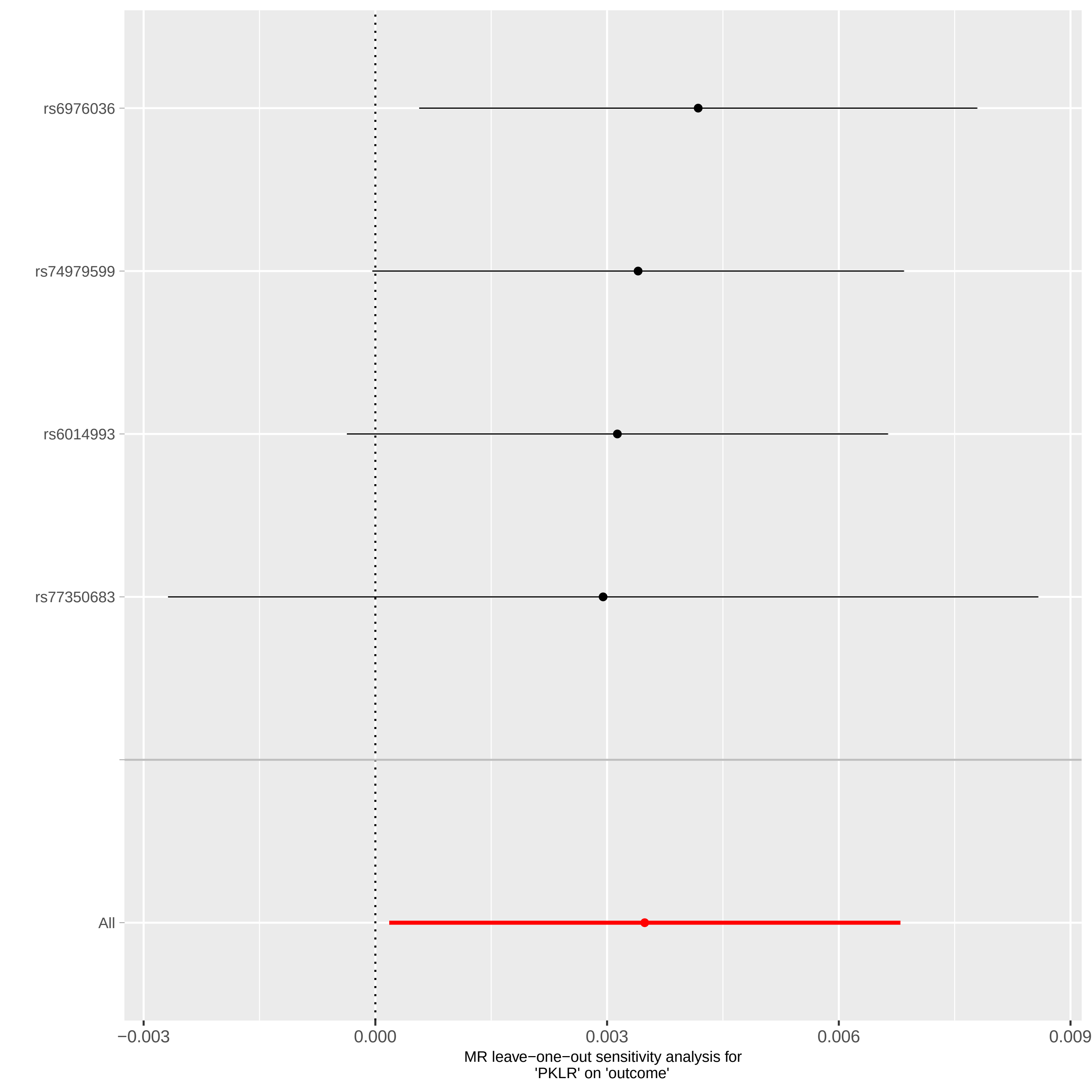

rs6976036
rs74979599
rs6014993
rs77350683
All
−0.003
0.000
0.003
0.006
0.009
MR leave−one−out sensitivity analysis for
'PKLR' on 'outcome'

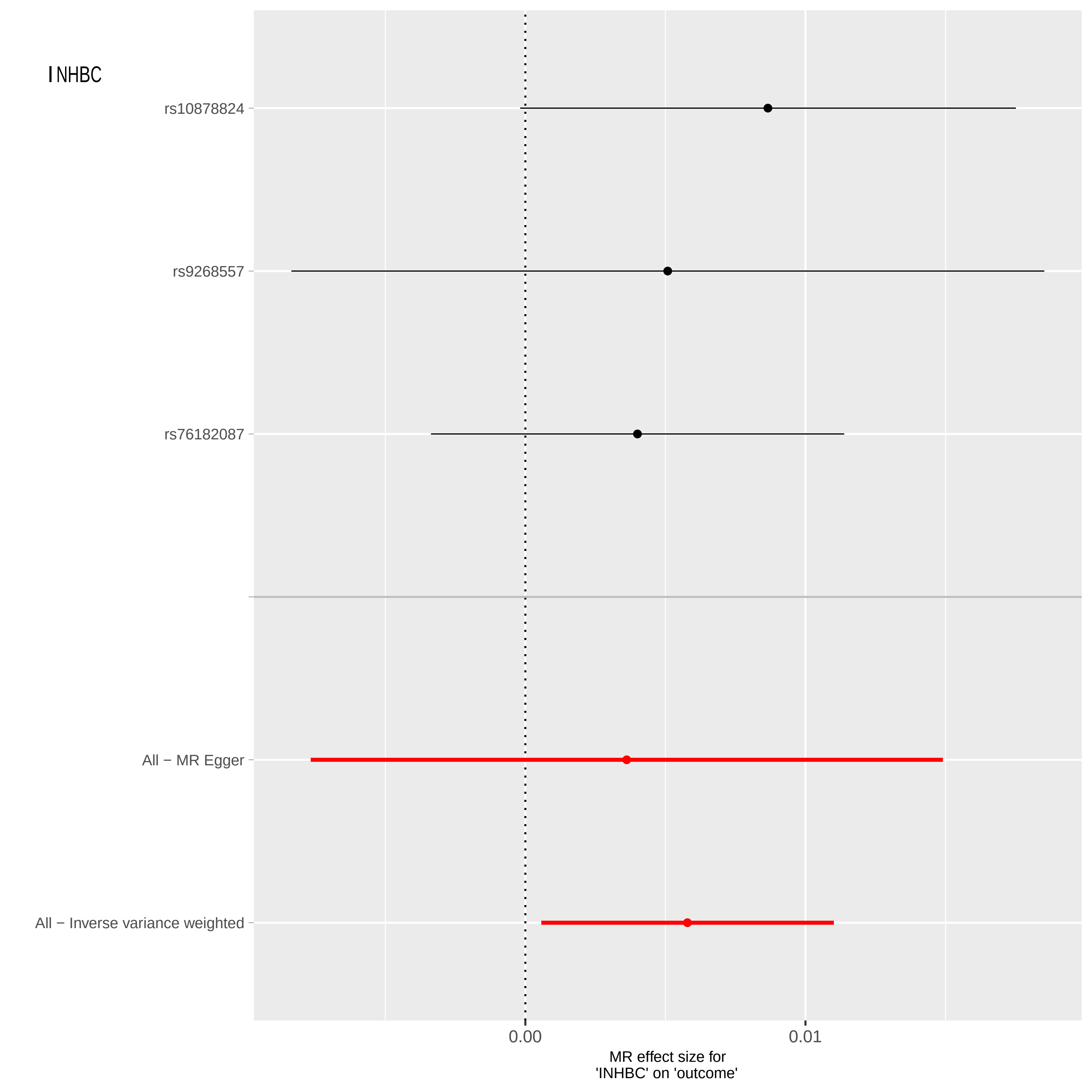
INHBC
rs10878824
rs9268557
rs76182087
All − MR Egger
All − Inverse variance weighted
0.00
0.01
MR effect size for
'INHBC' on 'outcome'

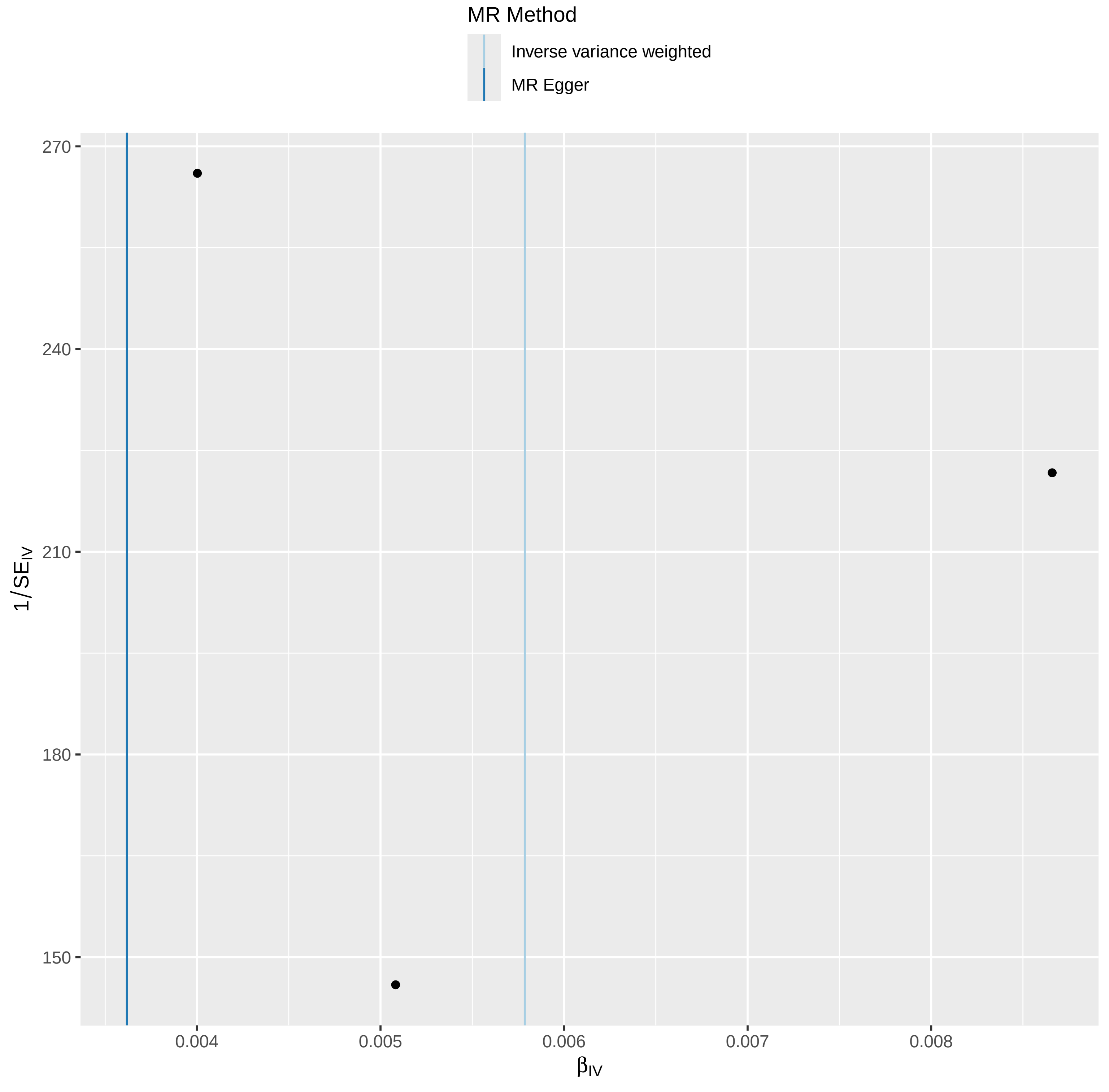
MR Method
Inverse variance weighted
MR Egger
270
240
210
180
150
1/SE_IV
0.004
0.005
0.006
0.007
0.008
β_IV

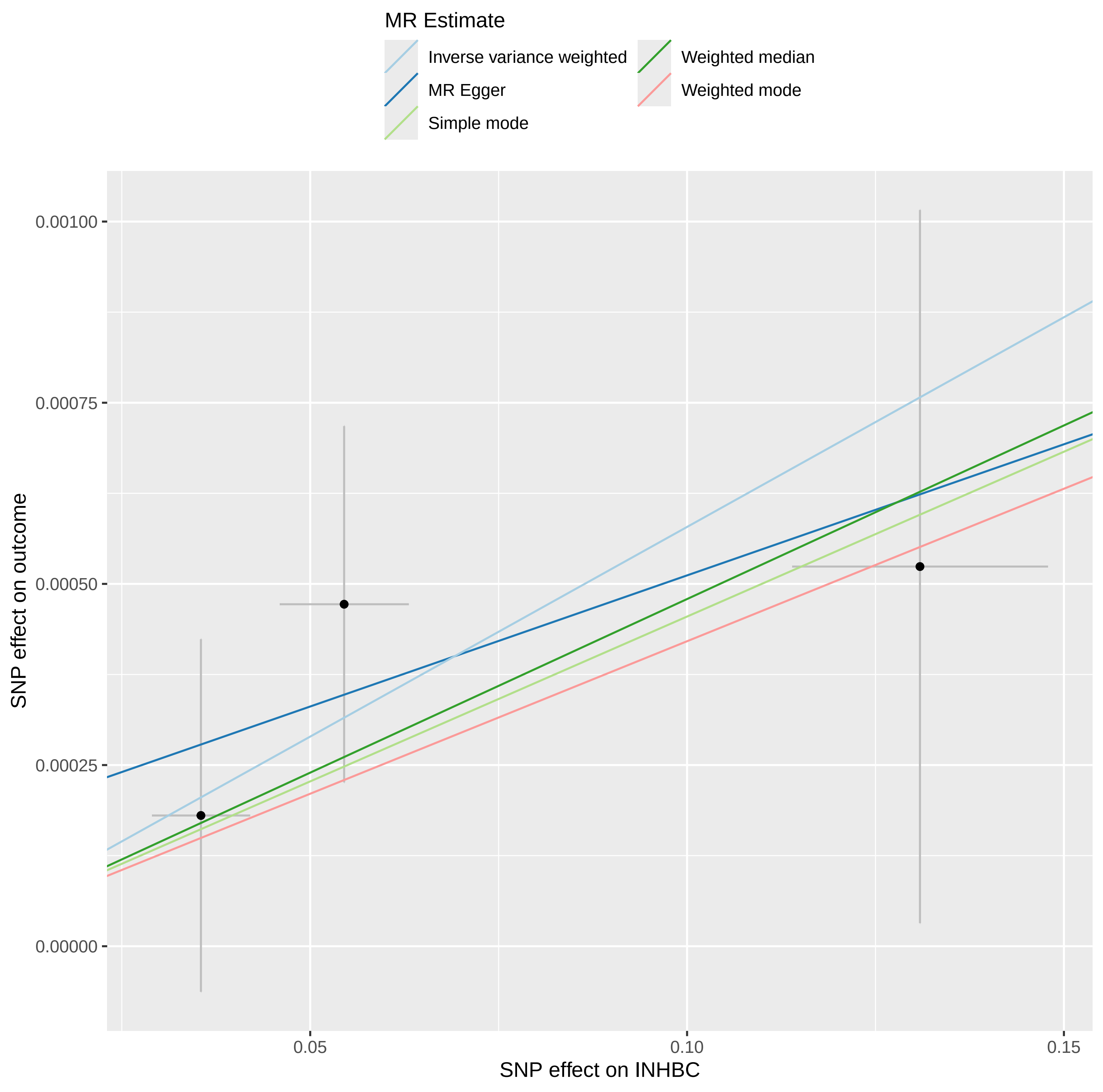

MR Estimate
Inverse variance weighted
MR Egger
Simple mode
Weighted median
Weighted mode
0.00100
0.00075
0.00050
0.00025
0.00000
SNP effect on outcome
0.05
0.10
0.15
SNP effect on INHBC

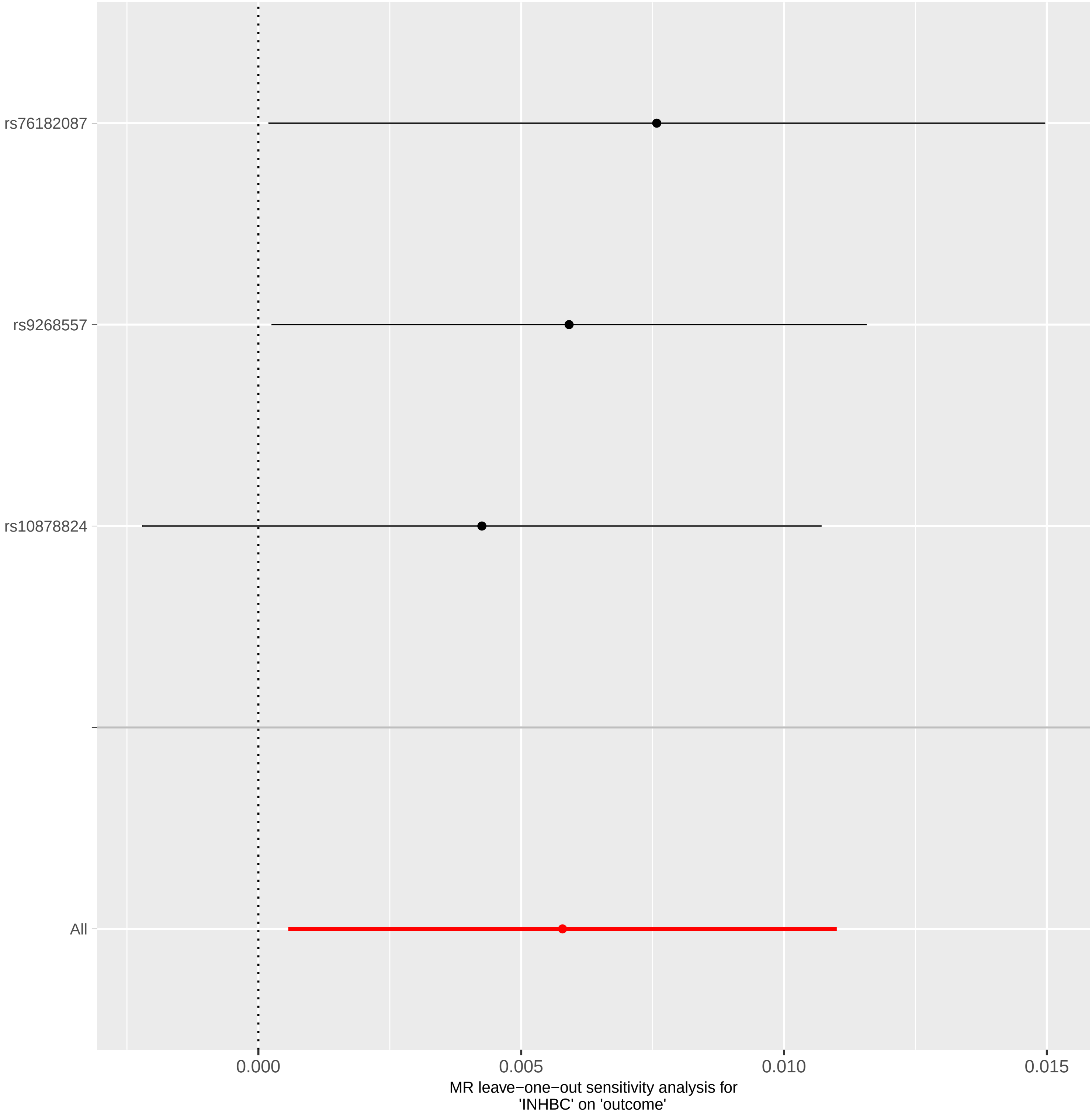

rs76182087
rs9268557
rs10878824
All
0.000
0.005
0.010
0.015
MR leave−one−out sensitivity analysis for
'INHBC' on 'outcome'

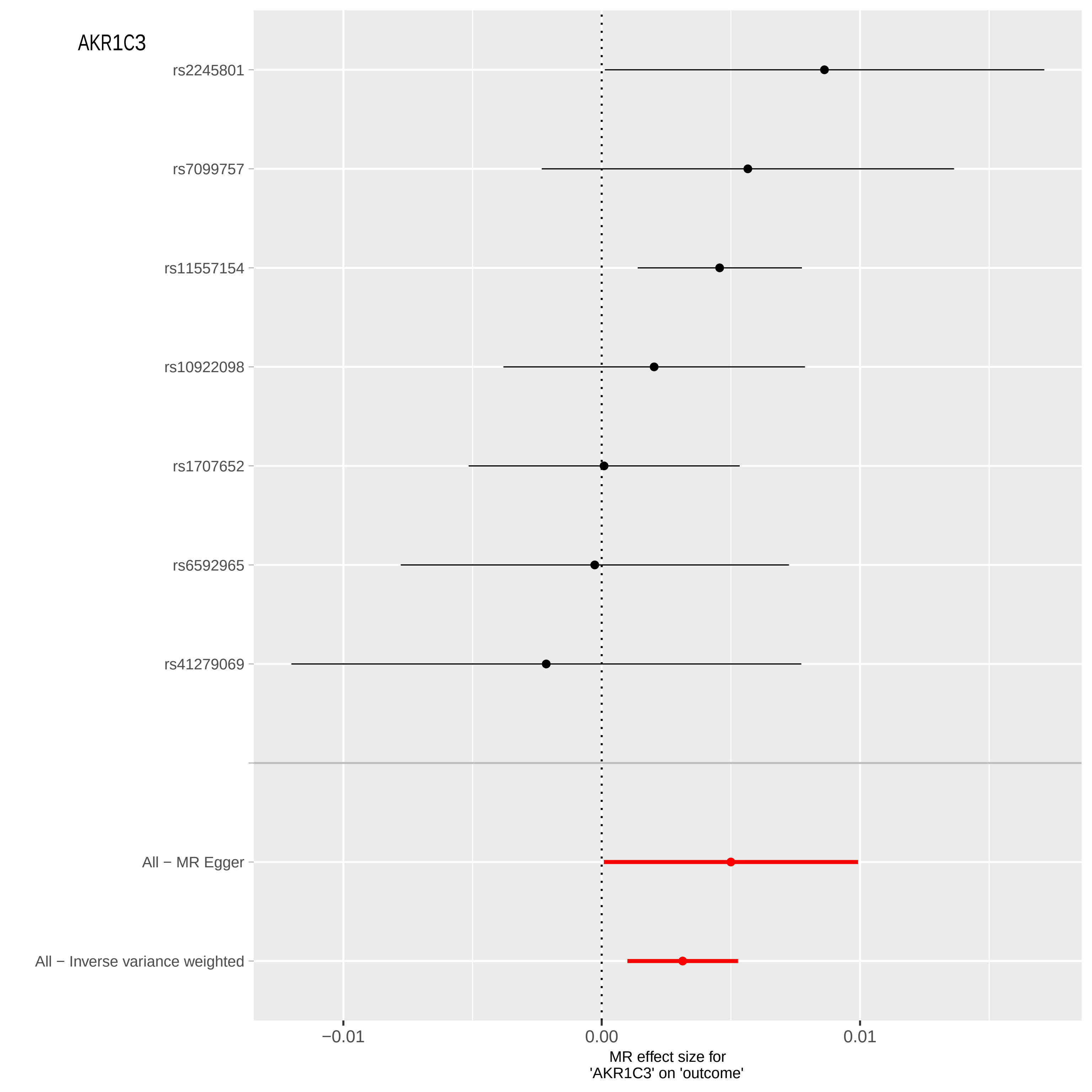
AKR1C3
rs2245801
rs7099757
rs11557154
rs10922098
rs1707652
rs6592965
rs41279069
All − MR Egger
All − Inverse variance weighted
−0.01
0.00
0.01
MR effect size for
'AKR1C3' on 'outcome'

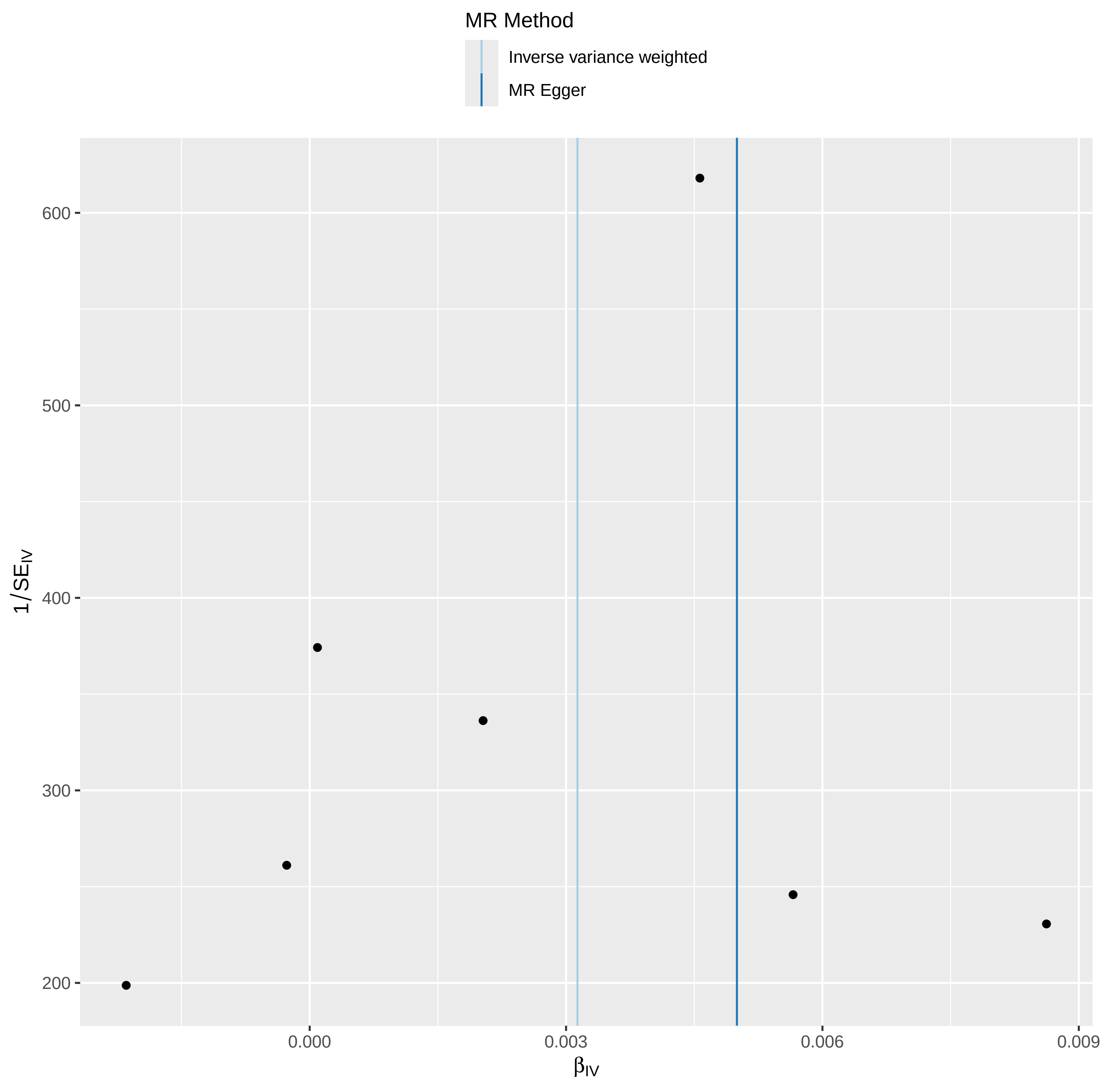

MR Method
Inverse variance weighted
MR Egger
600
500
400
300
200
1/SE_IV
0.000
0.003
0.006
0.009
β_IV

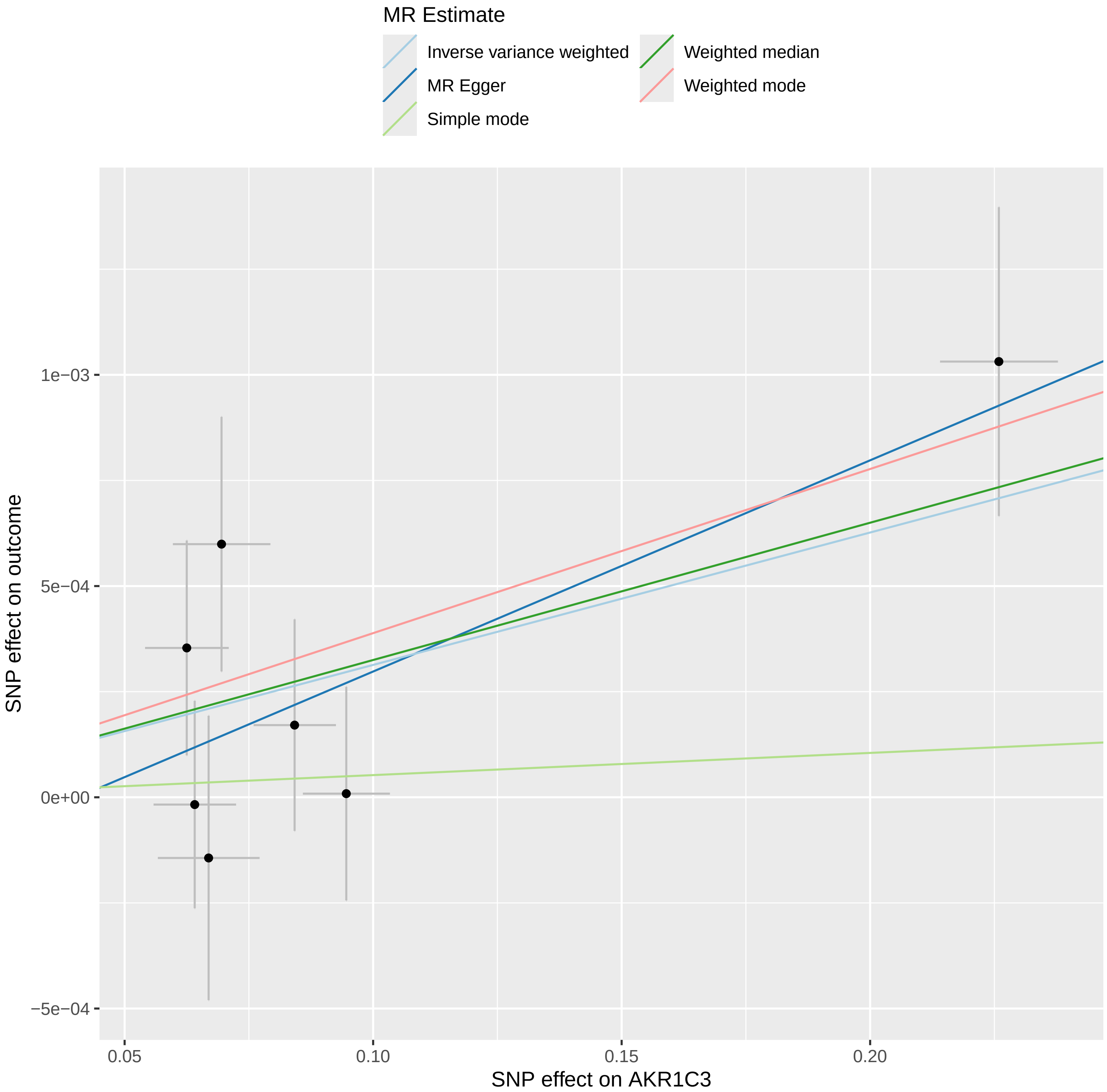
MR Estimate
Inverse variance weighted
MR Egger
Simple mode
Weighted median
Weighted mode
SNP effect on outcome
1e−03
5e−04
0e+00
−5e−04
0.05
0.10
0.15
0.20
SNP effect on AKR1C3

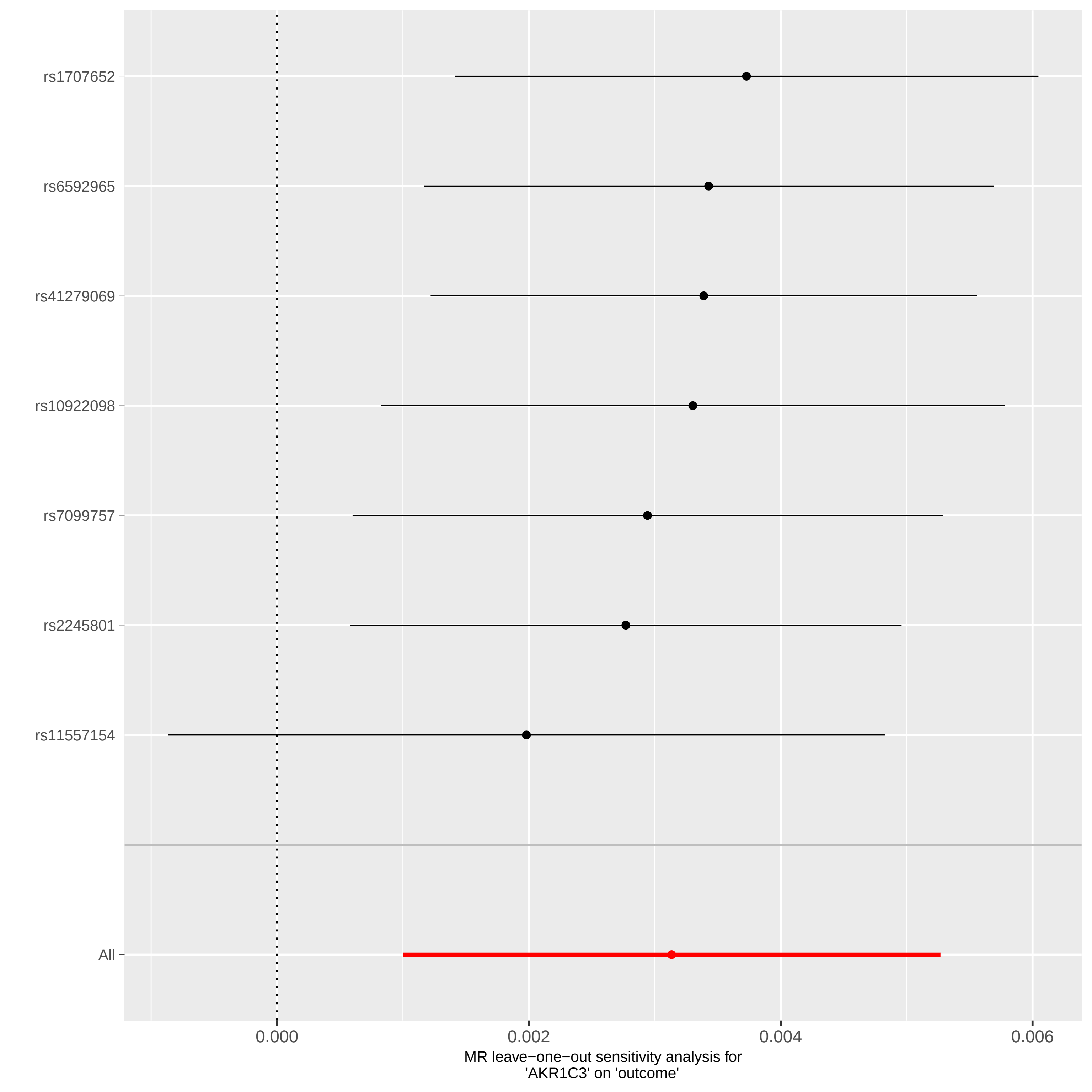

rs1707652
rs6592965
rs41279069
rs10922098
rs7099757
rs2245801
rs11557154
All
0.000
0.002
0.004
0.006
MR leave−one−out sensitivity analysis for
'AKR1C3' on 'outcome'

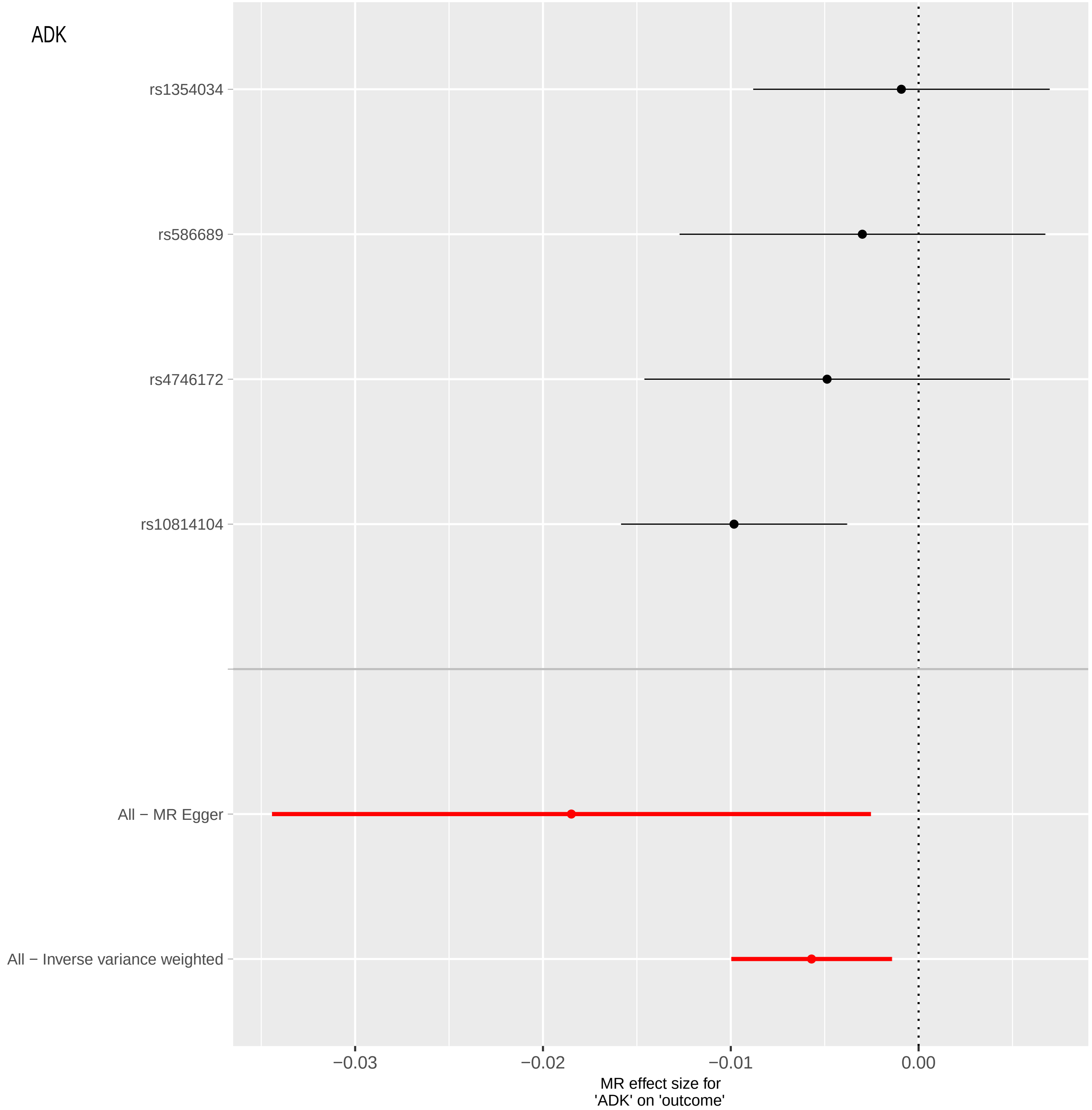
ADK
rs1354034
rs586689
rs4746172
rs10814104
All − MR Egger
All − Inverse variance weighted
−0.03
−0.02
−0.01
0.00
MR effect size for
'ADK' on 'outcome'

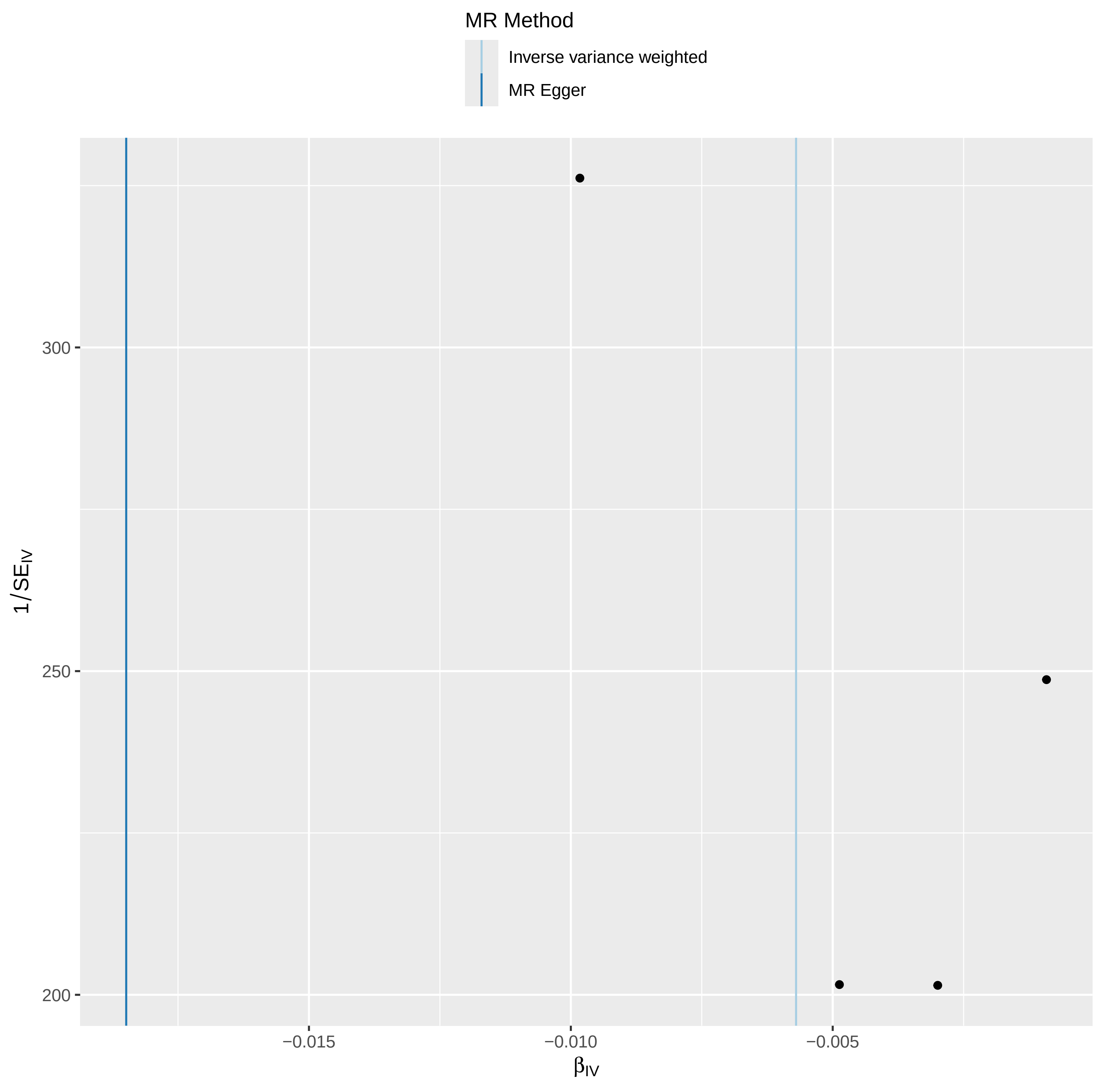
MR Method
Inverse variance weighted
MR Egger
300
250
200
1/SE_IV
−0.015
−0.010
−0.005
β_IV

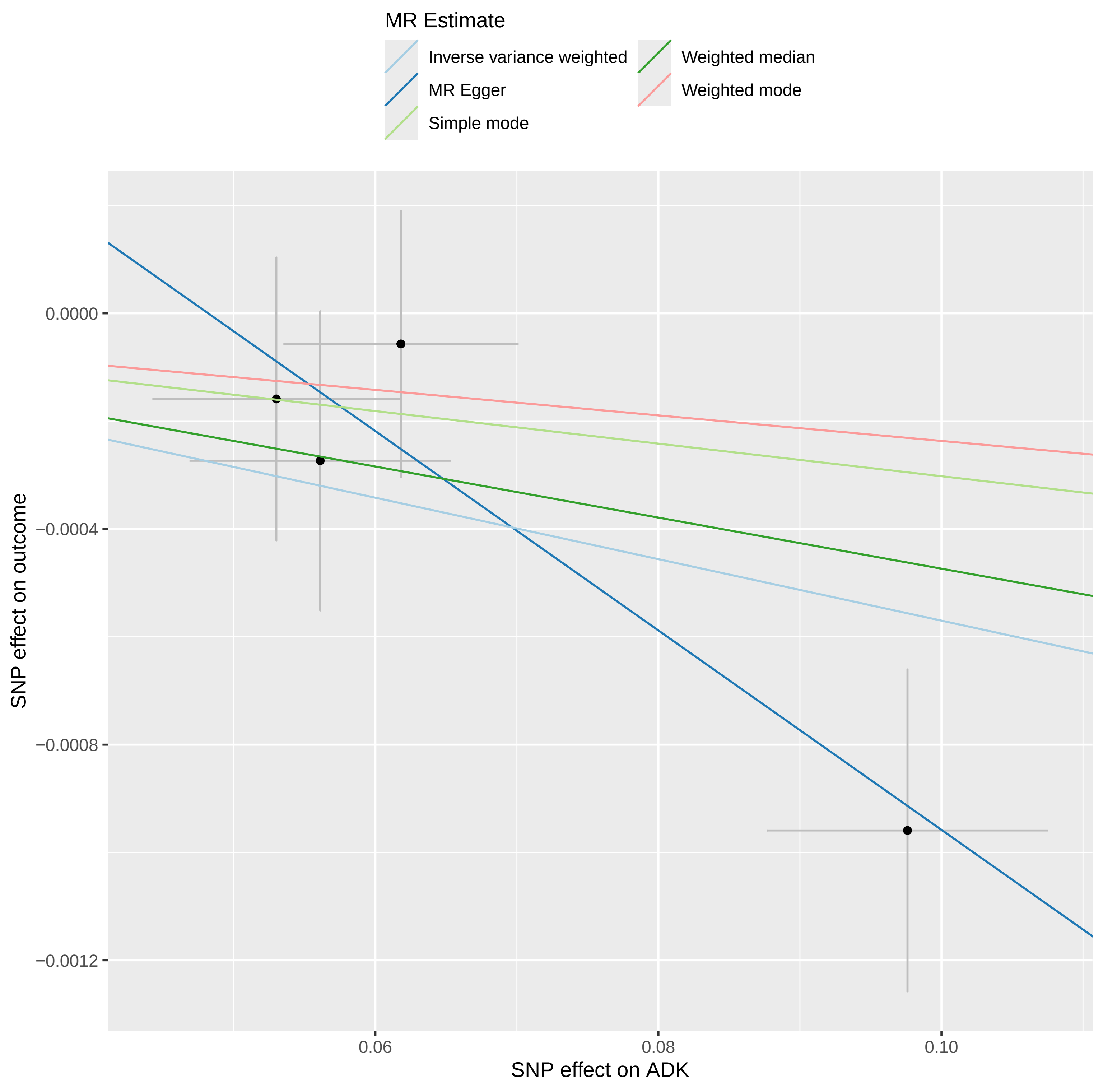

MR Estimate
Inverse variance weighted
MR Egger
Simple mode
Weighted median
Weighted mode
0.0000
−0.0004
−0.0008
−0.0012
SNP effect on outcome
0.06
0.08
0.10
SNP effect on ADK

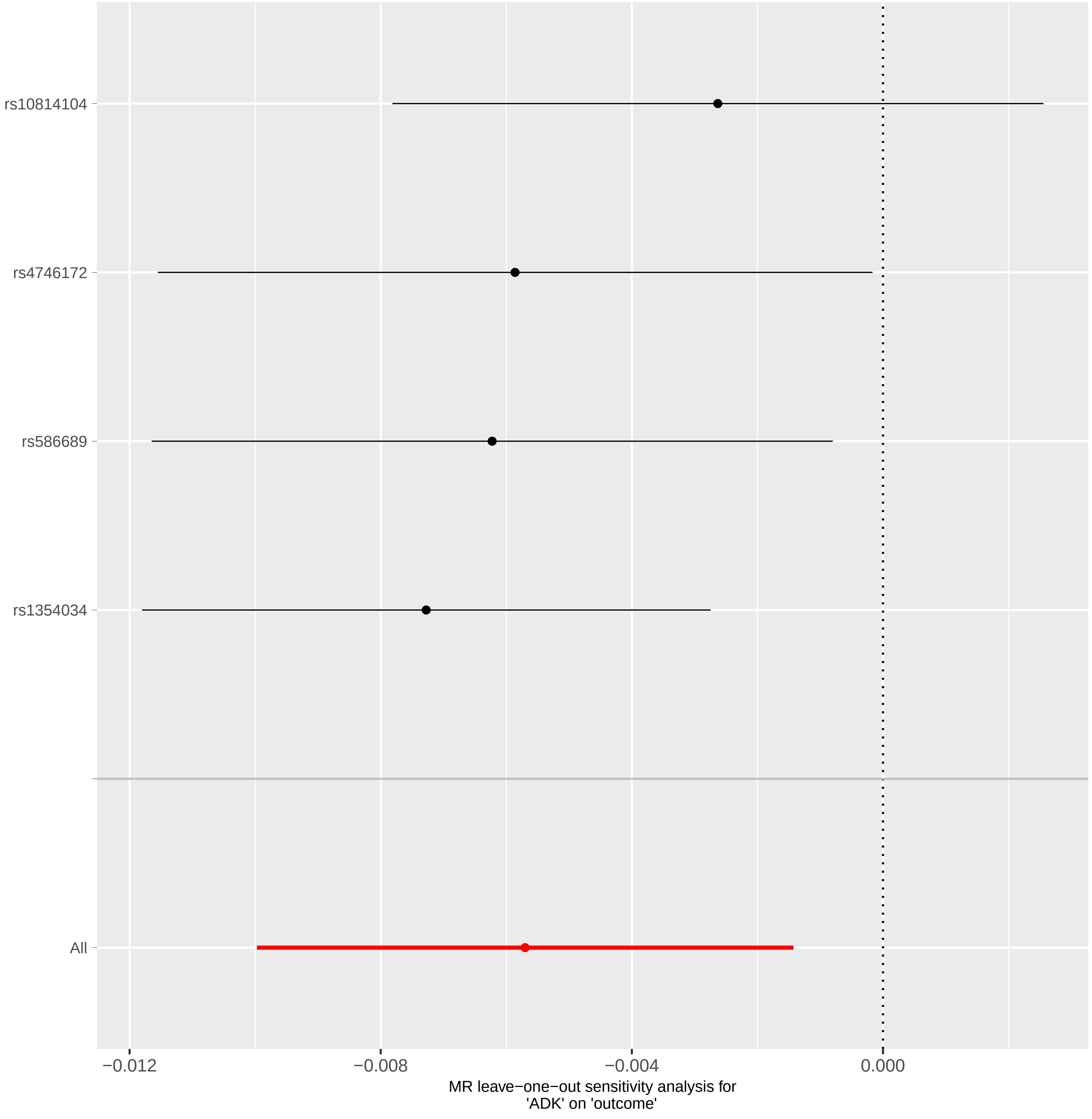

rs10814104
rs4746172
rs586689
rs1354034
All
−0.012
−0.008
−0.004
0.000
MR leave−one−out sensitivity analysis for
'ADK' on 'outcome'

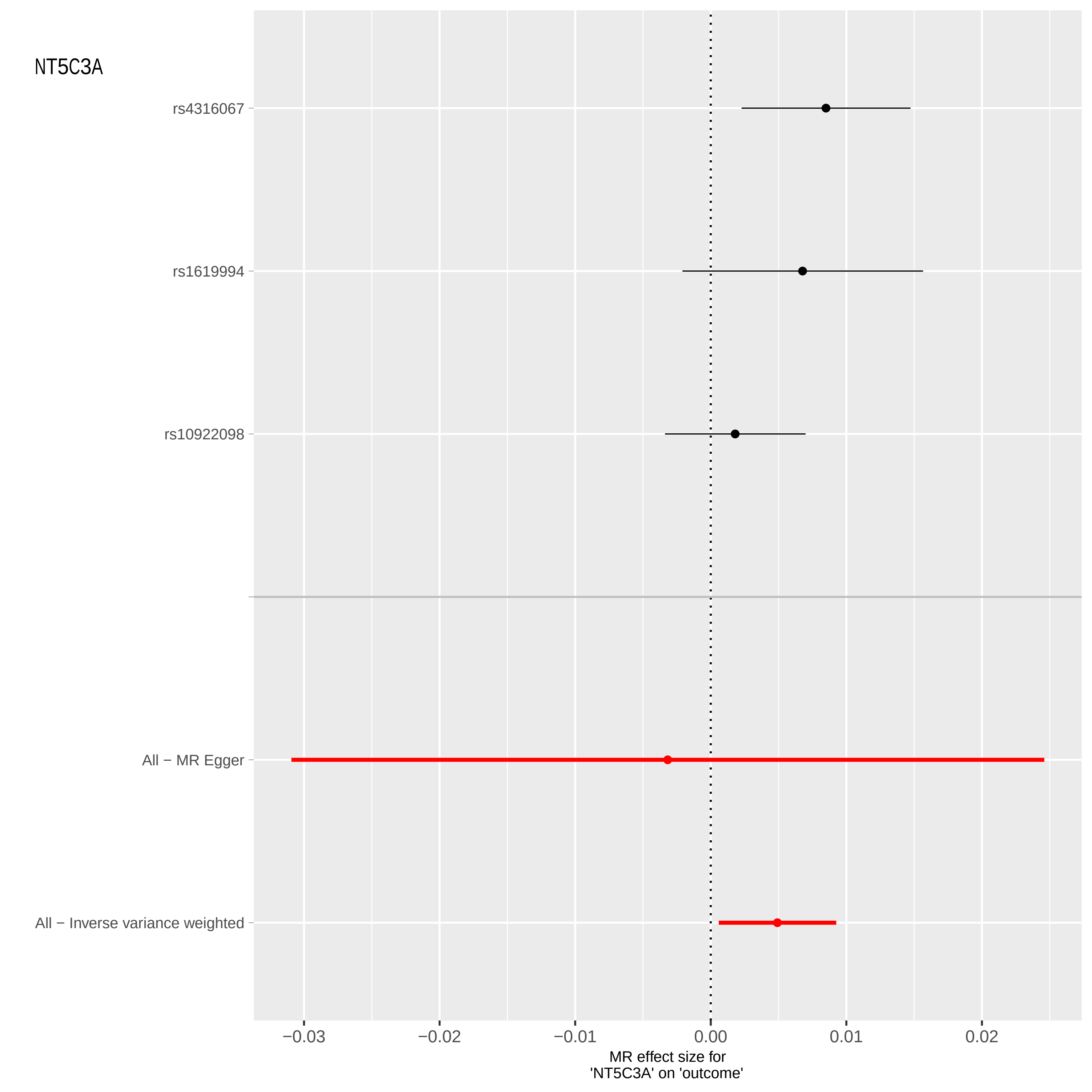
NT5C3A
rs4316067
rs1619994
rs10922098
All − MR Egger
All − Inverse variance weighted
−0.03
−0.02
−0.01
0.00
0.01
0.02
MR effect size for
'NT5C3A' on 'outcome'

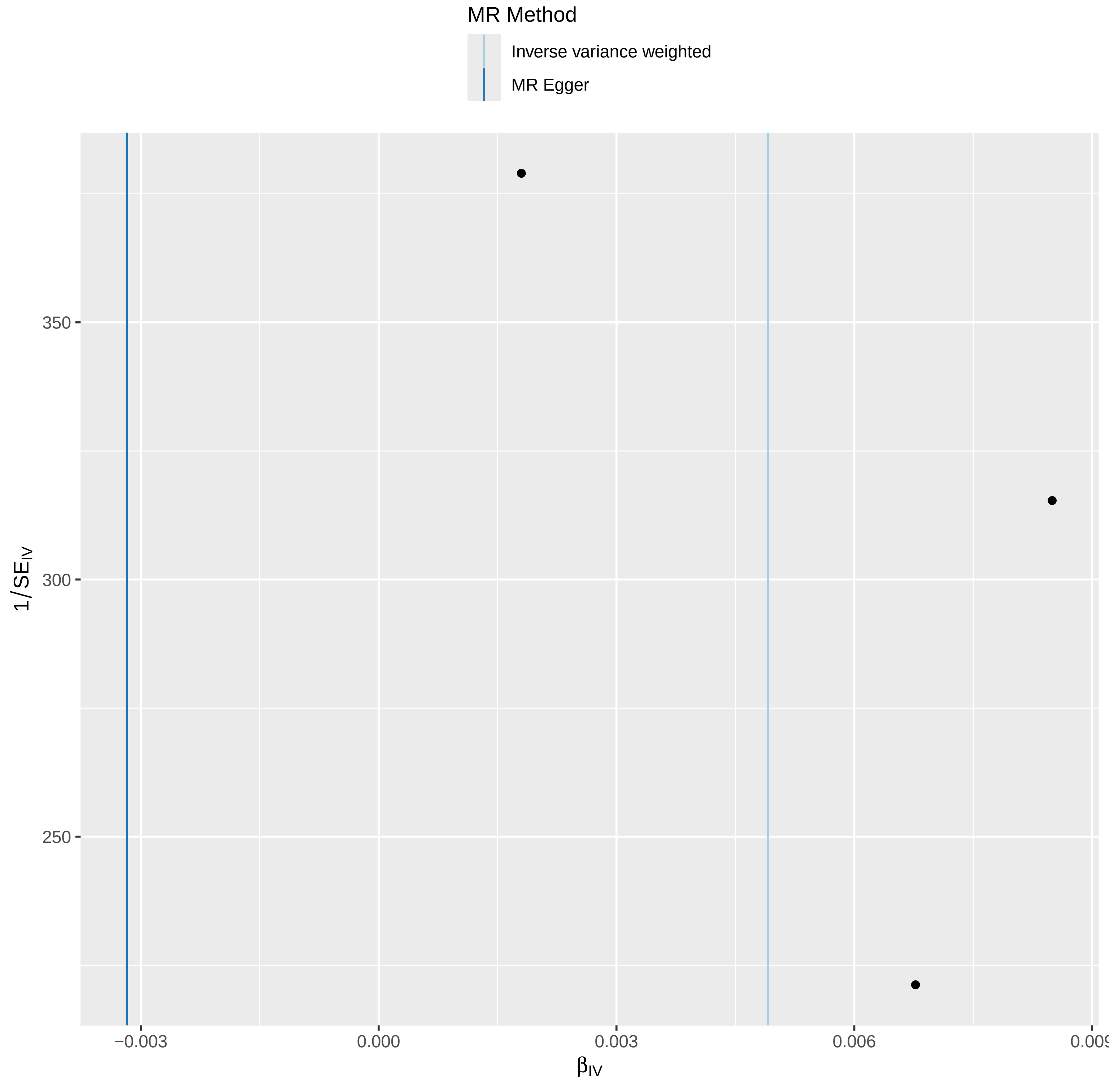
MR Method
Inverse variance weighted
MR Egger
350
300
250
$1/SE_{IV}$
−0.003
0.000
0.003
0.006
0.009
β_{IV}

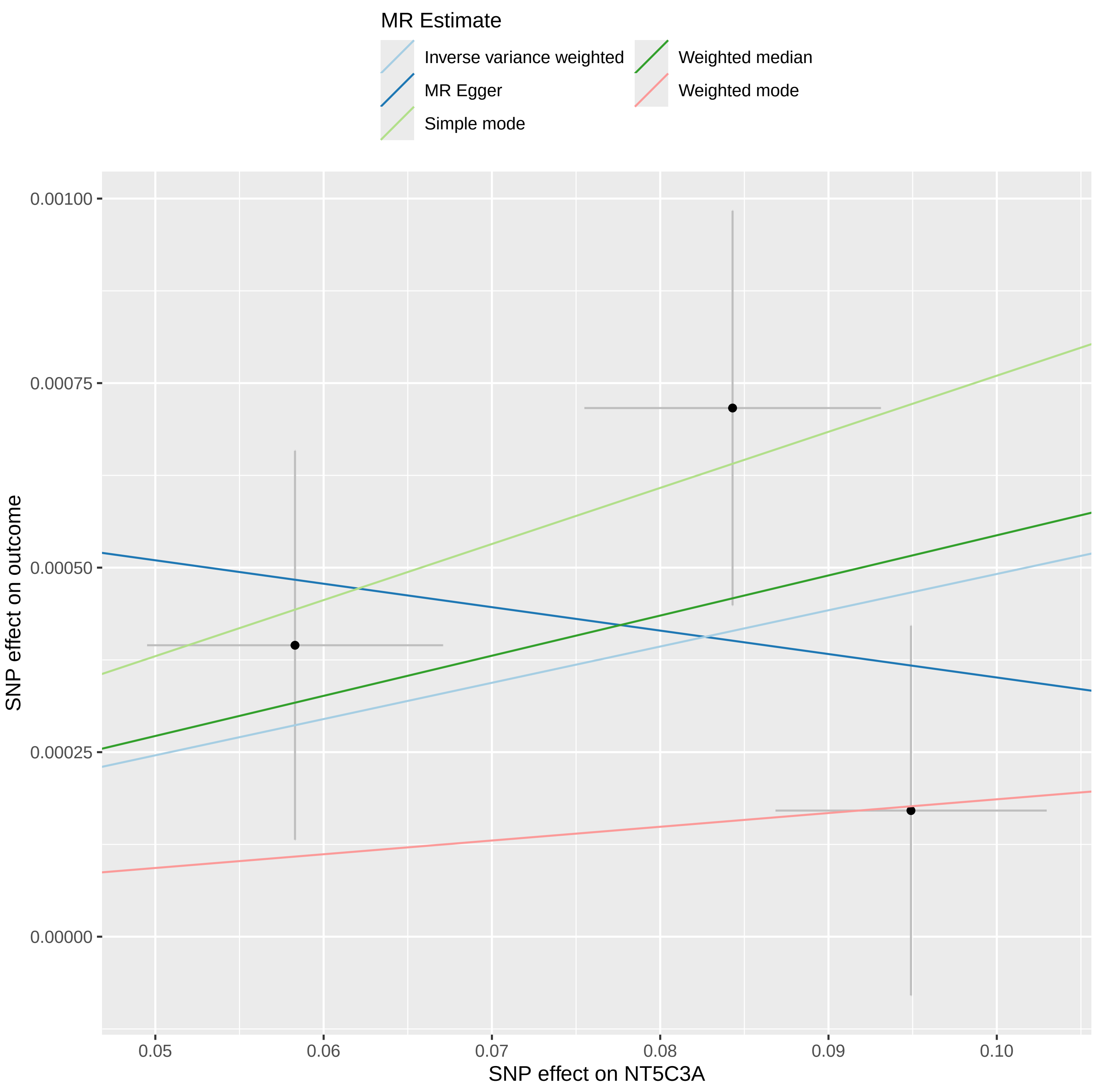

MR Estimate
Inverse variance weighted
MR Egger
Simple mode
Weighted median
Weighted mode
0.00100
0.00075
0.00050
0.00025
0.00000
SNP effect on outcome
0.05
0.06
0.07
0.08
0.09
0.10
SNP effect on NT5C3A

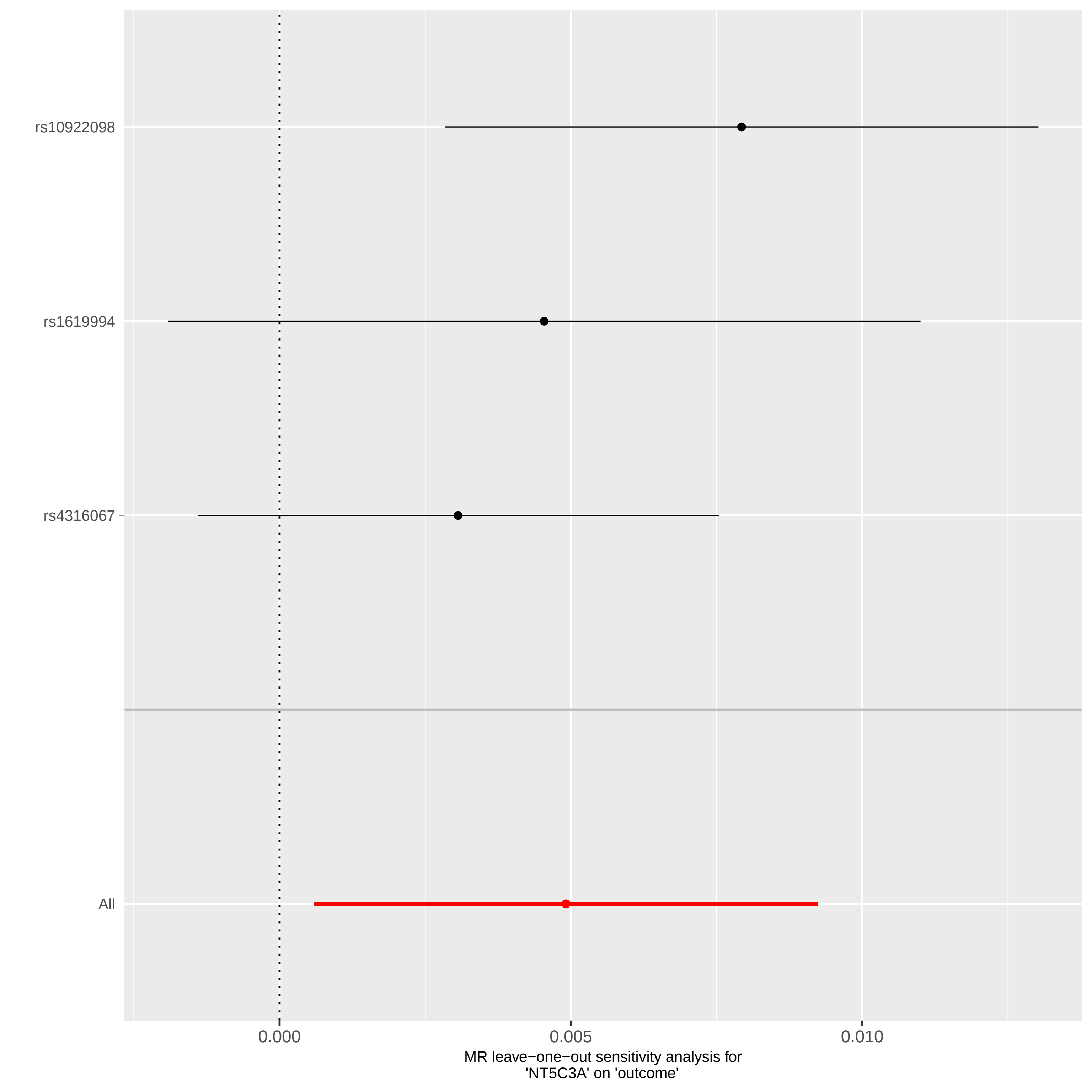
rs10922098
rs1619994
rs4316067
All
0.000
0.005
0.010
MR leave−one−out sensitivity analysis for
'NT5C3A' on 'outcome'
