# Supplementary material for: Decoding the lipid etiology of atherogenic index of plasma and gout: establishing the causal role of triglycerides through NHANES, Mendelian randomization, and network pharmacology
Source: Cardiovasc Diabetol Endocrinol Rep. 2026 Jul 13;12:40. doi: 10.1186/s40842-026-00309-0 (PMC13362044; doi:10.1186/s40842-026-00309-0)
Supplement: Supplementary file 9 — Supplementary Material 9 [file 40842_2026_309_MOESM9_ESM.pdf]

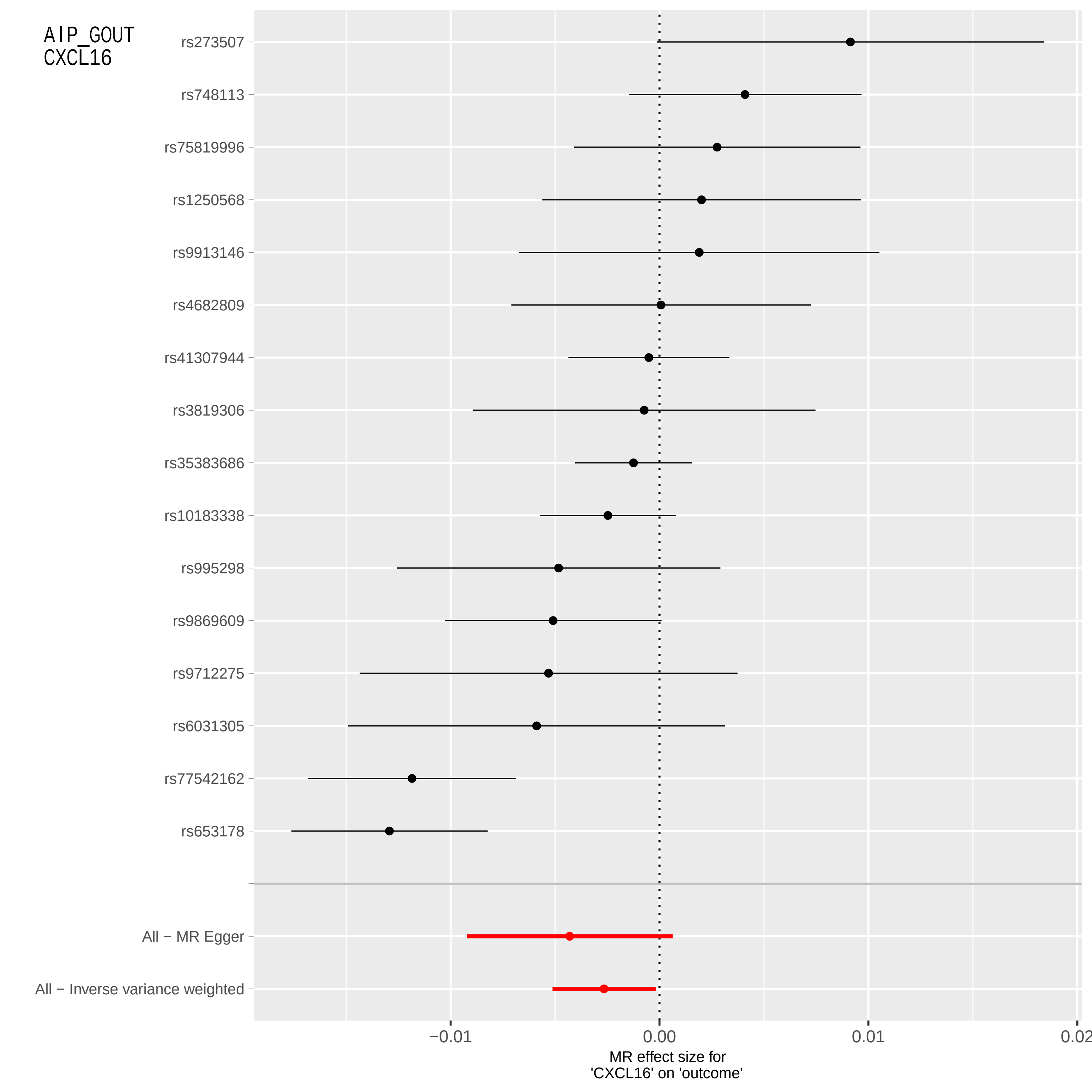

AIP_GOUT
CXCL16
rs273507
rs748113
rs75819996
rs1250568
rs9913146
rs4682809
rs41307944
rs3819306
rs35383686
rs10183338
rs995298
rs9869609
rs9712275
rs6031305
rs77542162
rs653178
All − MR Egger
All − Inverse variance weighted
−0.01
0.00
0.01
0.02
MR effect size for
'CXCL16' on 'outcome'

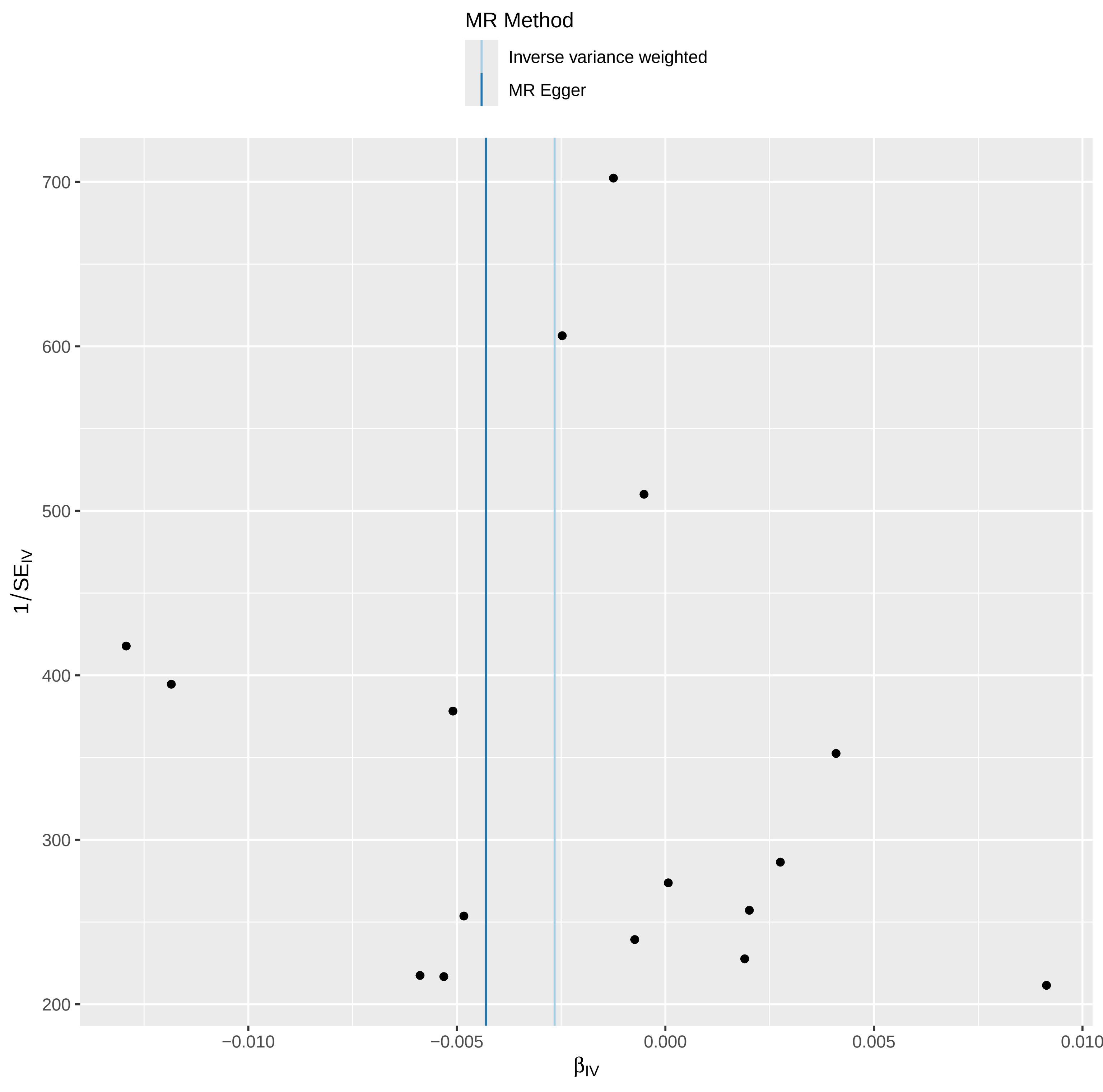

MR Method
Inverse variance weighted
MR Egger
700
600
500
400
300
200
1/SE_IV
−0.010
−0.005
0.000
0.005
0.010
β_IV

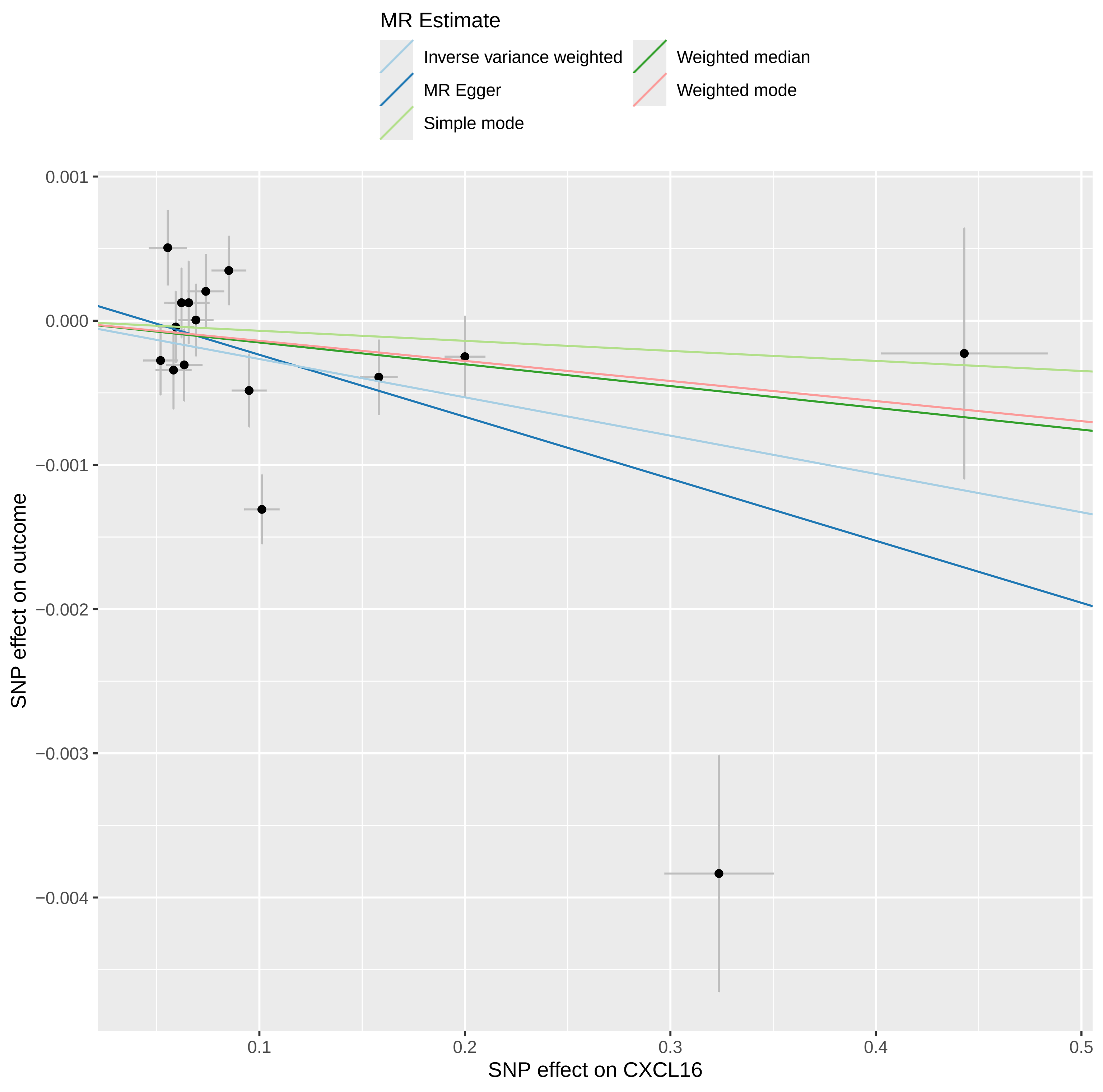

MR Estimate
Inverse variance weighted
MR Egger
Simple mode
Weighted median
Weighted mode
0.001
0.000
−0.001
−0.002
−0.003
−0.004
0.1
0.2
0.3
0.4
0.5
SNP effect on outcome
SNP effect on CXCL16

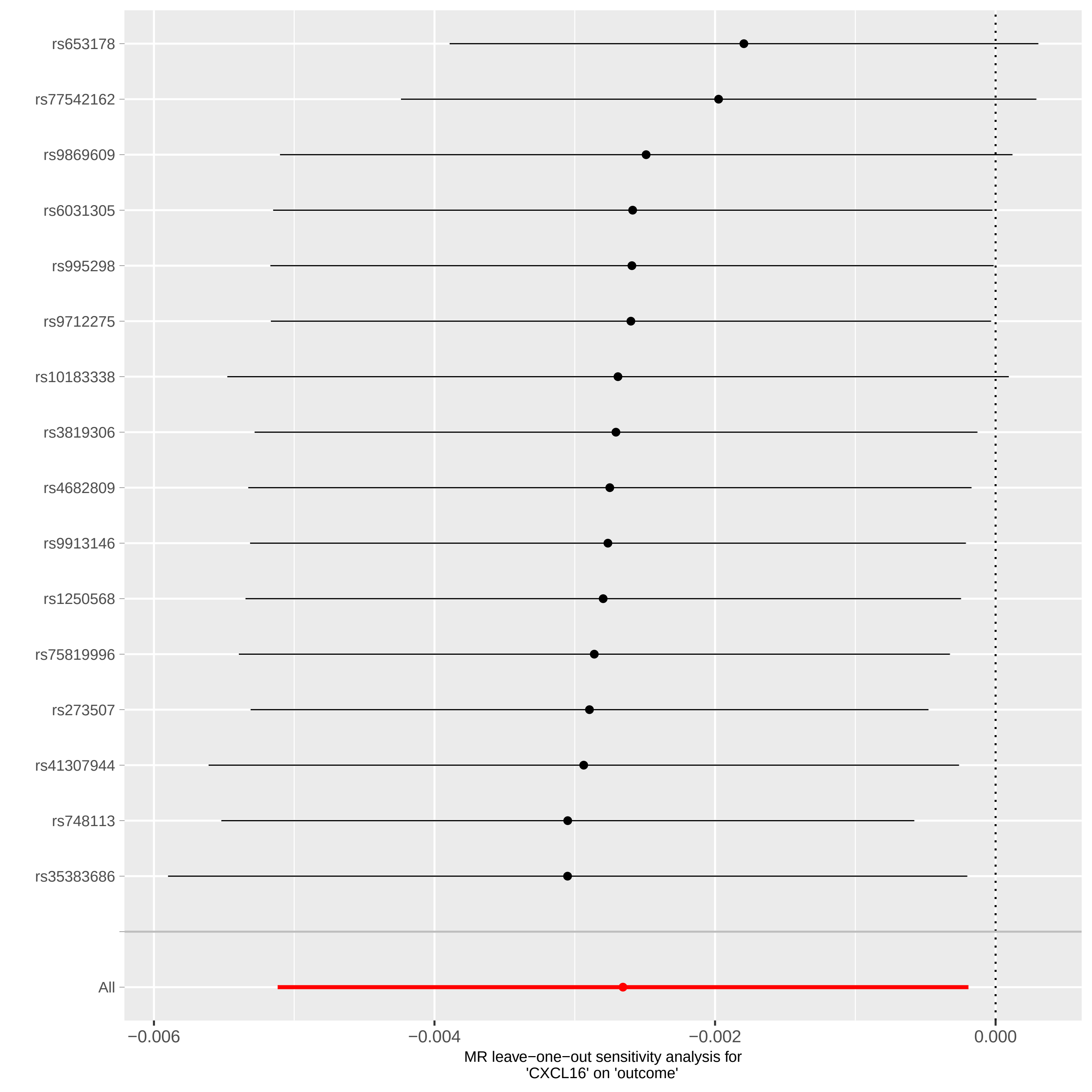
rs653178
rs77542162
rs9869609
rs6031305
rs995298
rs9712275
rs10183338
rs3819306
rs4682809
rs9913146
rs1250568
rs75819996
rs273507
rs41307944
rs748113
rs35383686
All
−0.006
−0.004
−0.002
0.000
MR leave−one−out sensitivity analysis for
'CXCL16' on 'outcome'

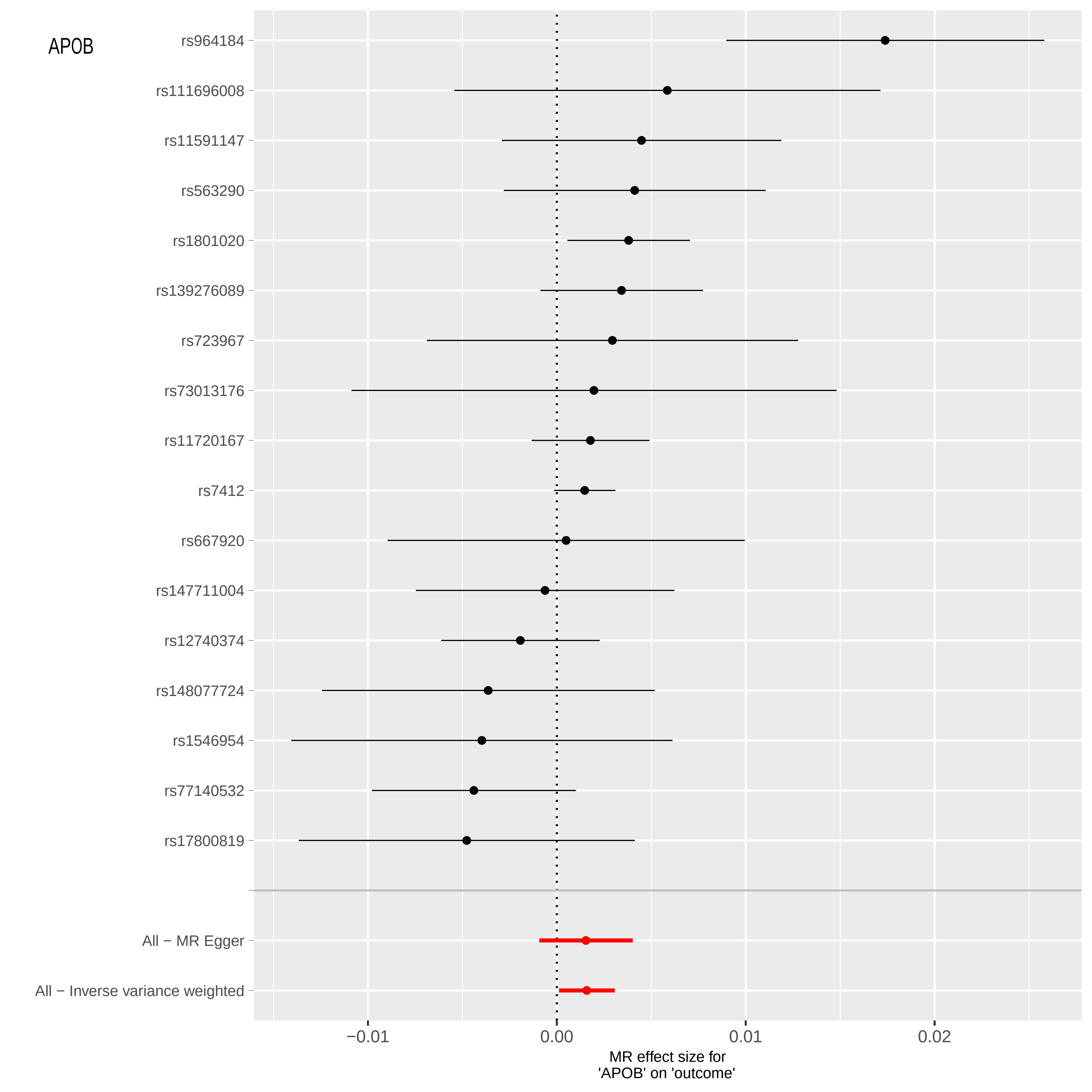
APOB
rs964184
rs111696008
rs11591147
rs563290
rs1801020
rs139276089
rs723967
rs73013176
rs11720167
rs7412
rs667920
rs147711004
rs12740374
rs148077724
rs1546954
rs77140532
rs17800819
All − MR Egger
All − Inverse variance weighted
−0.01
0.00
0.01
0.02
MR effect size for
'APOB' on 'outcome'

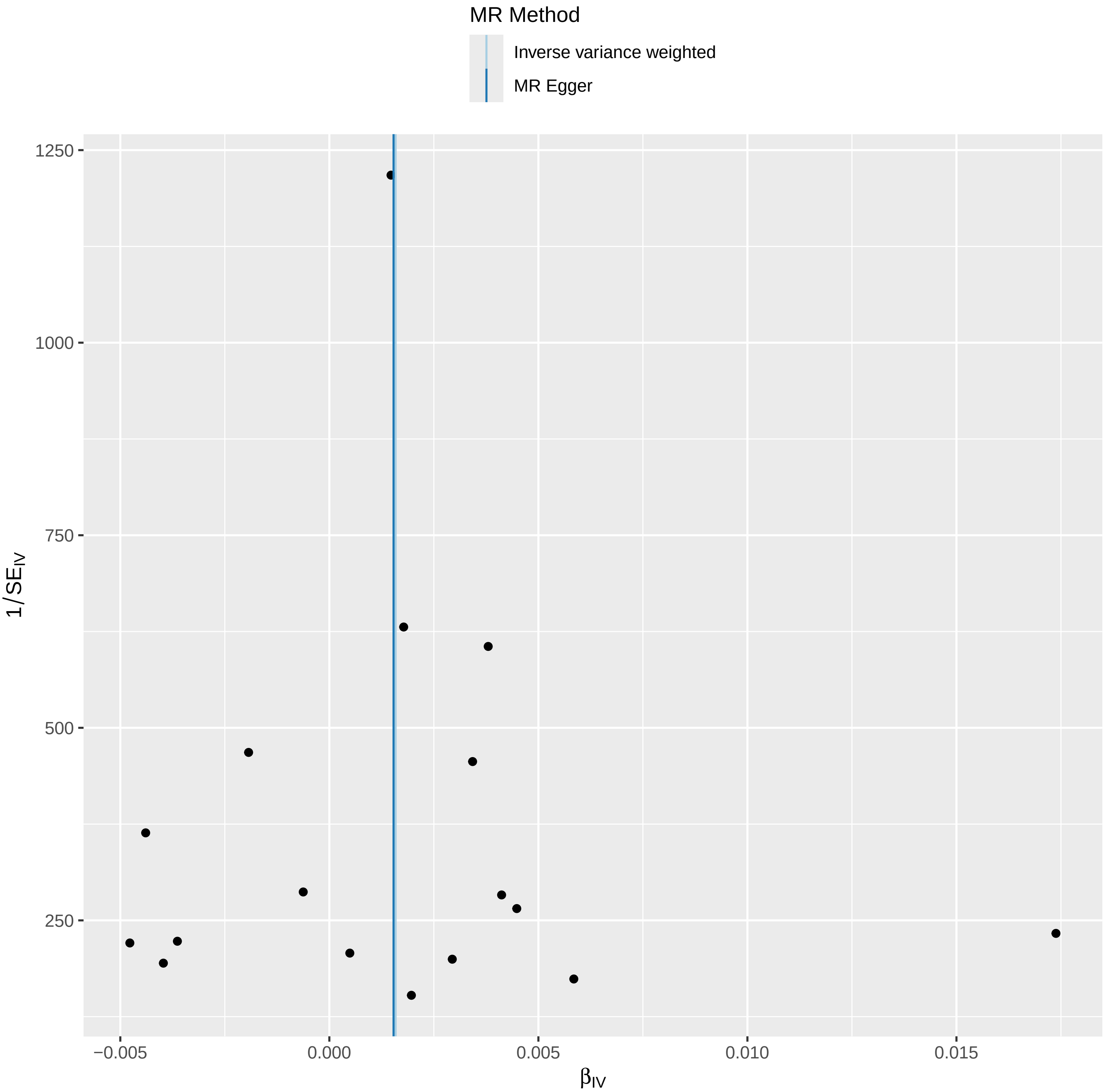
MR Method
Inverse variance weighted
MR Egger
1250
1000
750
500
250
1/SE$_{IV}$
−0.005
0.000
0.005
0.010
0.015
β$_{IV}$

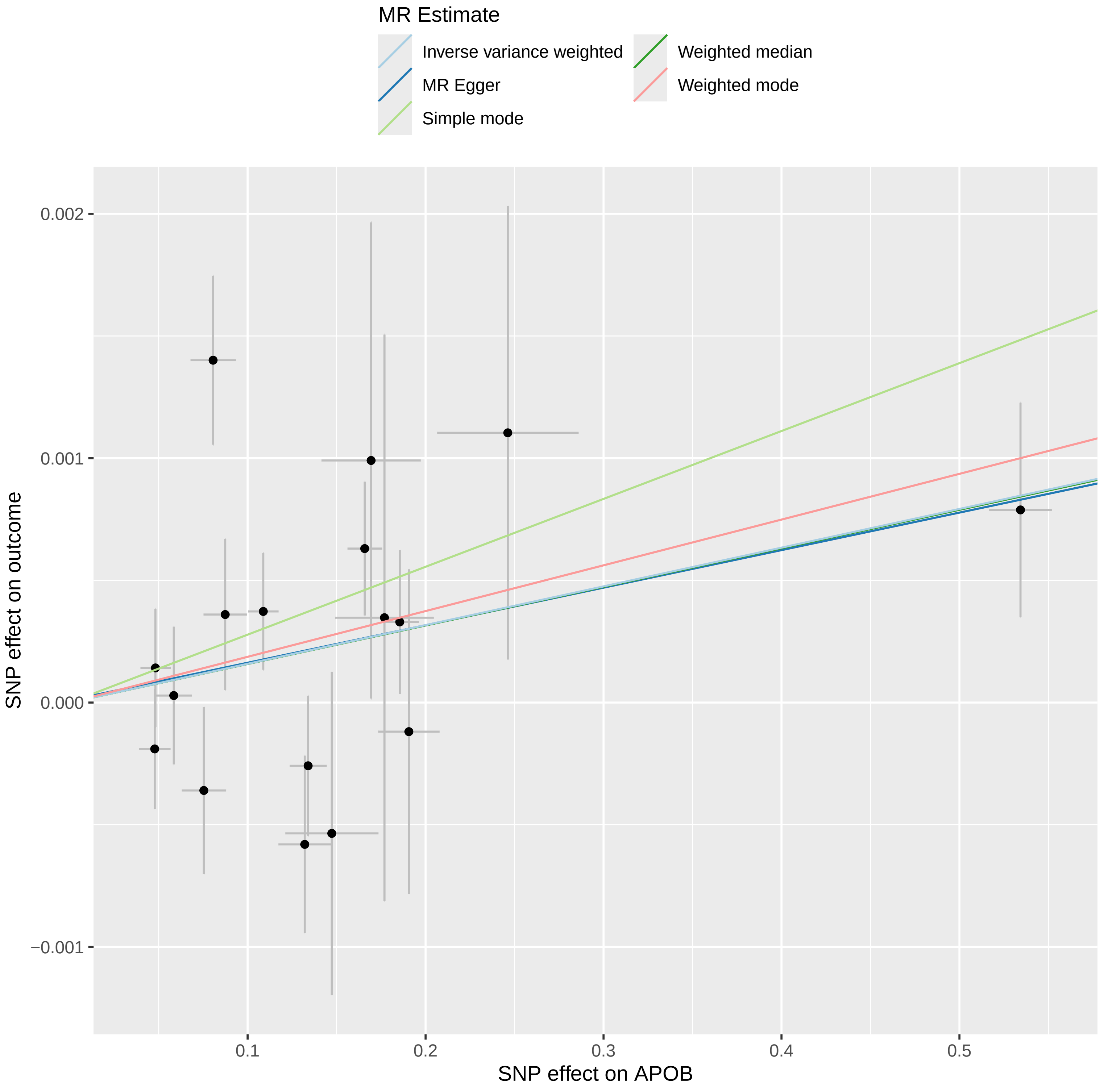
MR Estimate
Inverse variance weighted
MR Egger
Simple mode
Weighted median
Weighted mode
0.002
0.001
0.000
−0.001
SNP effect on outcome
0.1
0.2
0.3
0.4
0.5
SNP effect on APOB

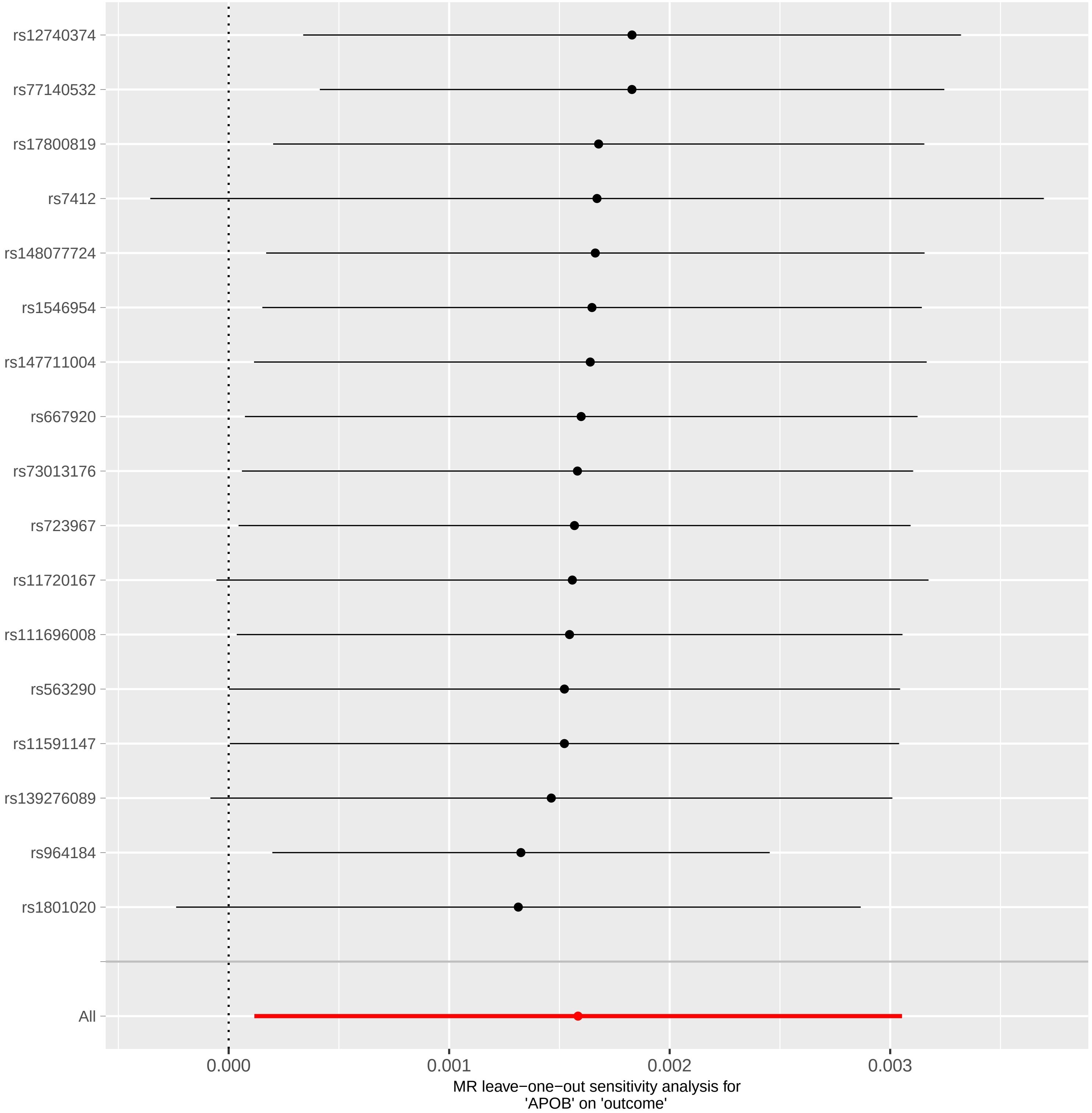
rs12740374
rs77140532
rs17800819
rs7412
rs148077724
rs1546954
rs147711004
rs667920
rs73013176
rs723967
rs11720167
rs111696008
rs563290
rs11591147
rs139276089
rs964184
rs1801020
All
0.000
0.001
0.002
0.003
MR leave−one−out sensitivity analysis for
'APOB' on 'outcome'

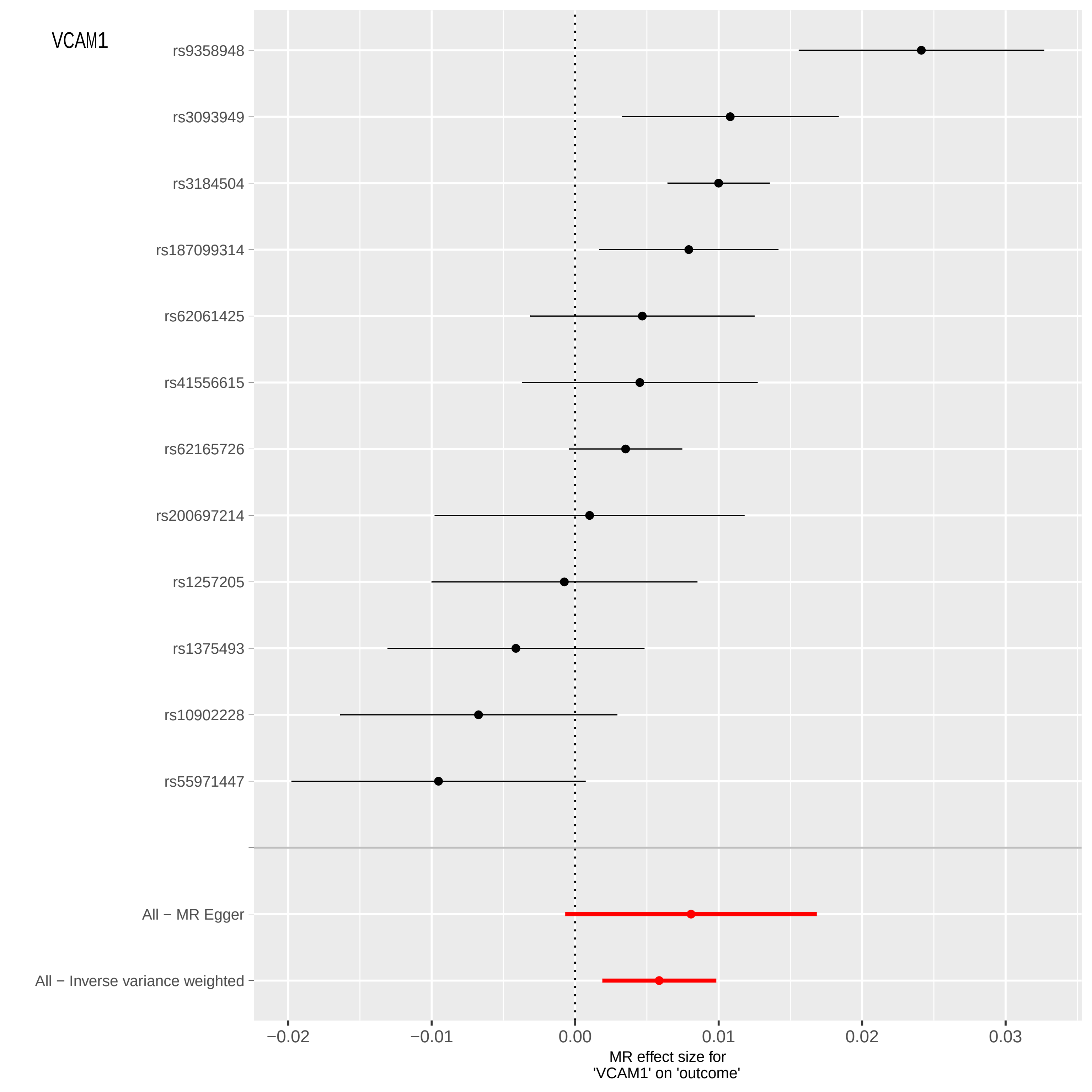

VCAM1
rs9358948
rs3093949
rs3184504
rs187099314
rs62061425
rs41556615
rs62165726
rs200697214
rs1257205
rs1375493
rs10902228
rs55971447
All − MR Egger
All − Inverse variance weighted
−0.02
−0.01
0.00
0.01
0.02
0.03
MR effect size for
'VCAM1' on 'outcome'

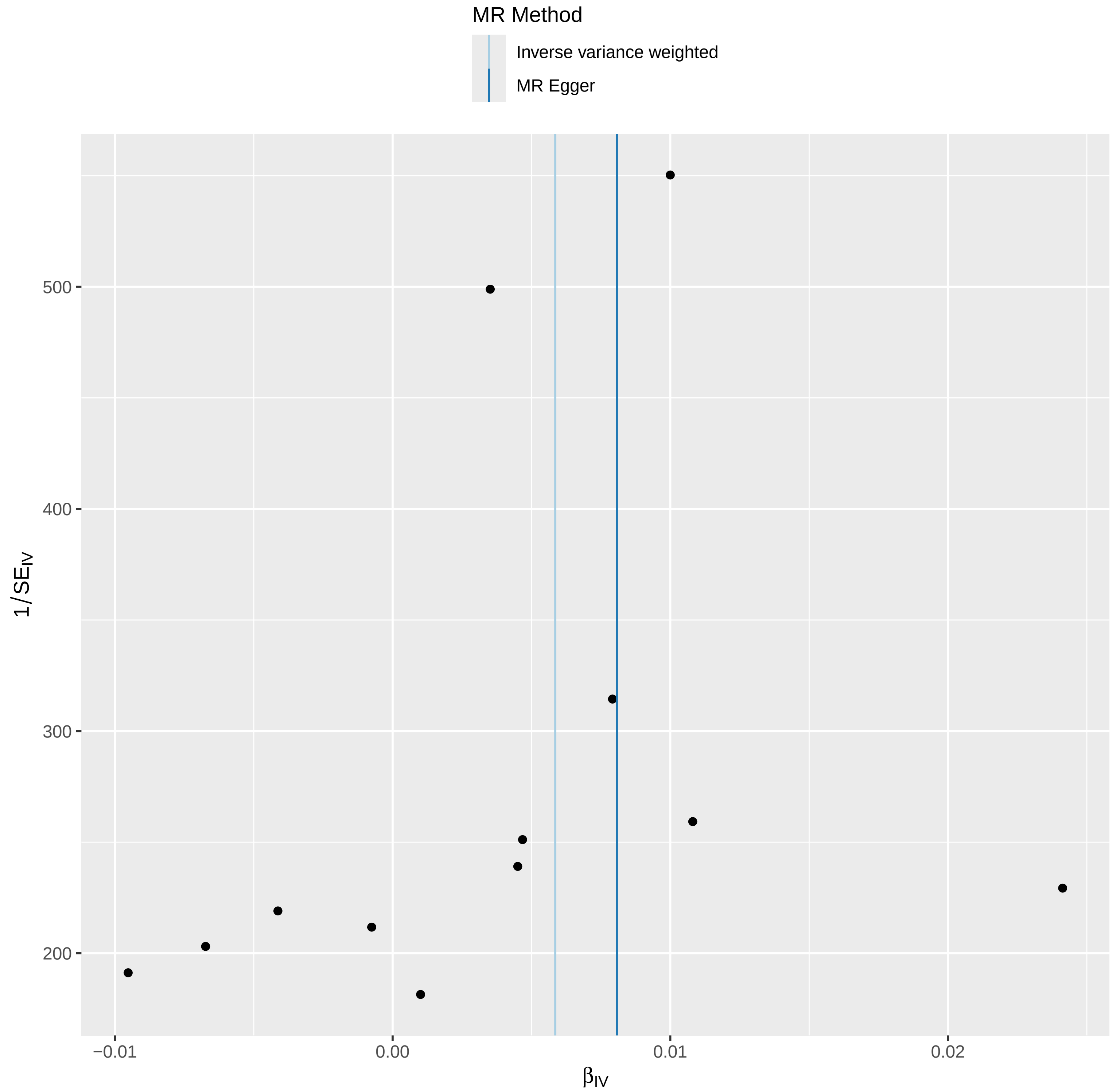

MR Method
Inverse variance weighted
MR Egger
500
400
300
200
1/SE$_{IV}$
−0.01
0.00
0.01
0.02
β$_{IV}$

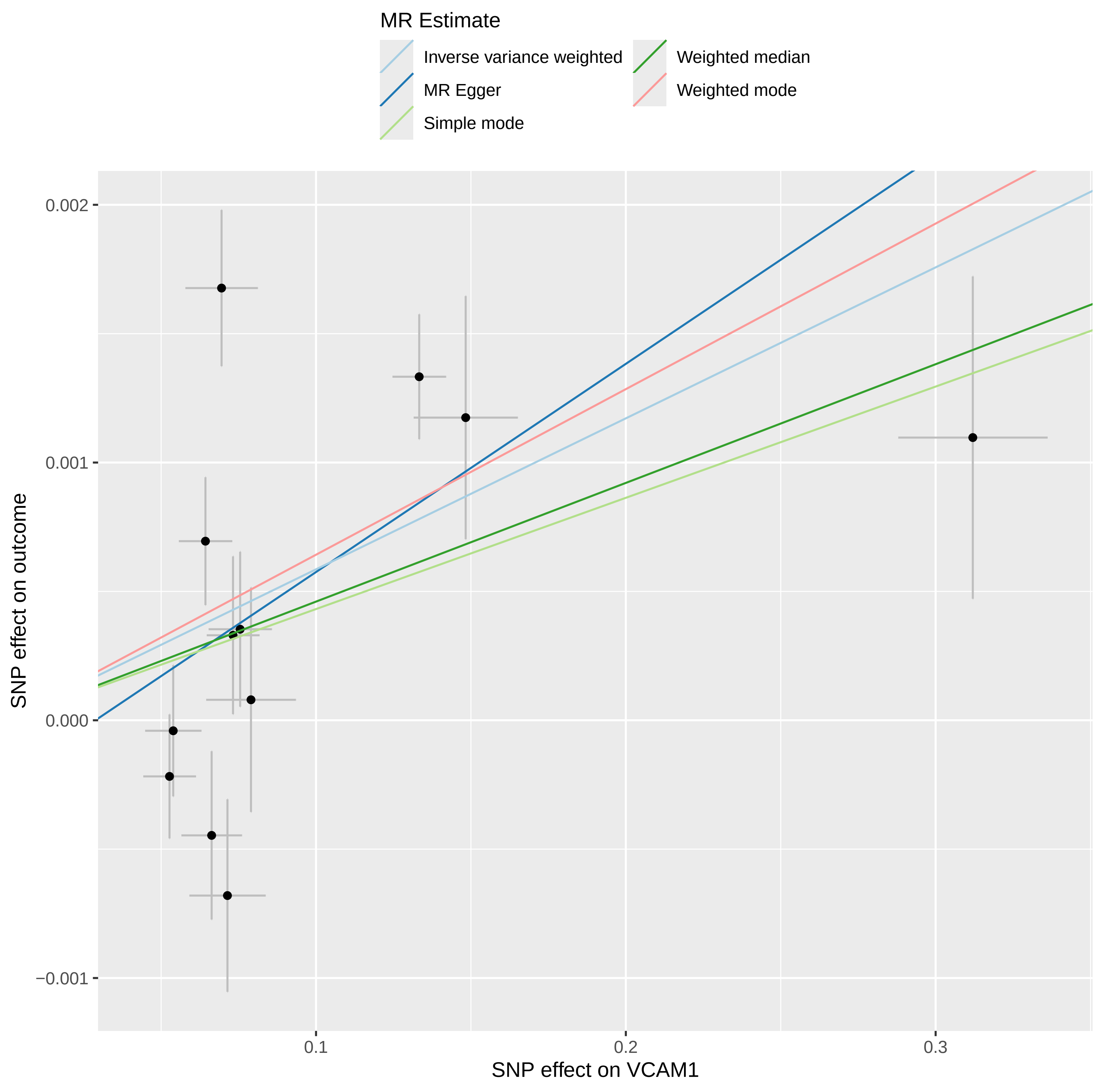

MR Estimate
Inverse variance weighted
MR Egger
Simple mode
Weighted median
Weighted mode
0.002
0.001
0.000
−0.001
SNP effect on outcome
0.1
0.2
0.3
SNP effect on VCAM1

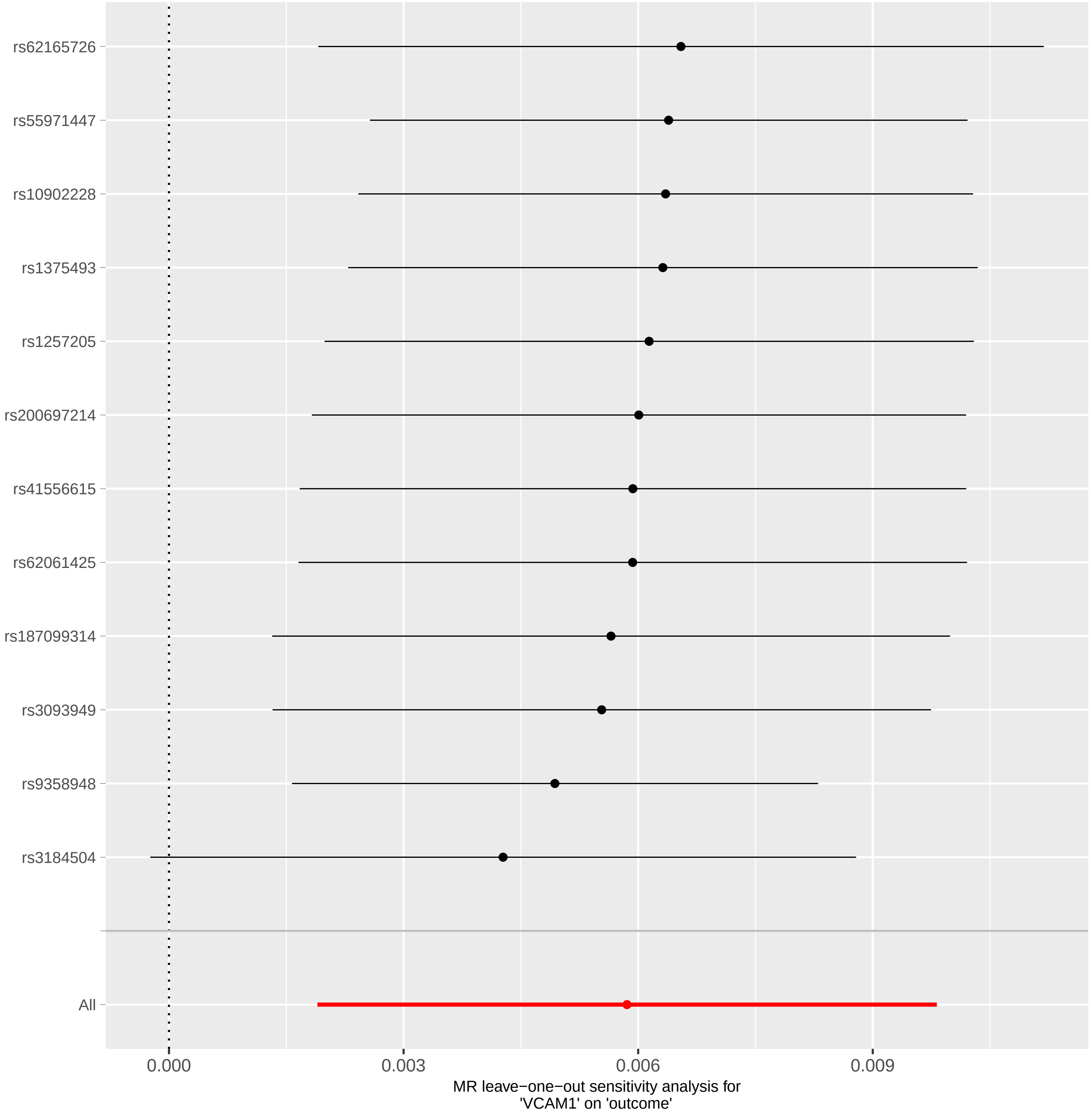
rs62165726
rs55971447
rs10902228
rs1375493
rs1257205
rs200697214
rs41556615
rs62061425
rs187099314
rs3093949
rs9358948
rs3184504
All
0.000
0.003
0.006
0.009
MR leave−one−out sensitivity analysis for
'VCAM1' on 'outcome'

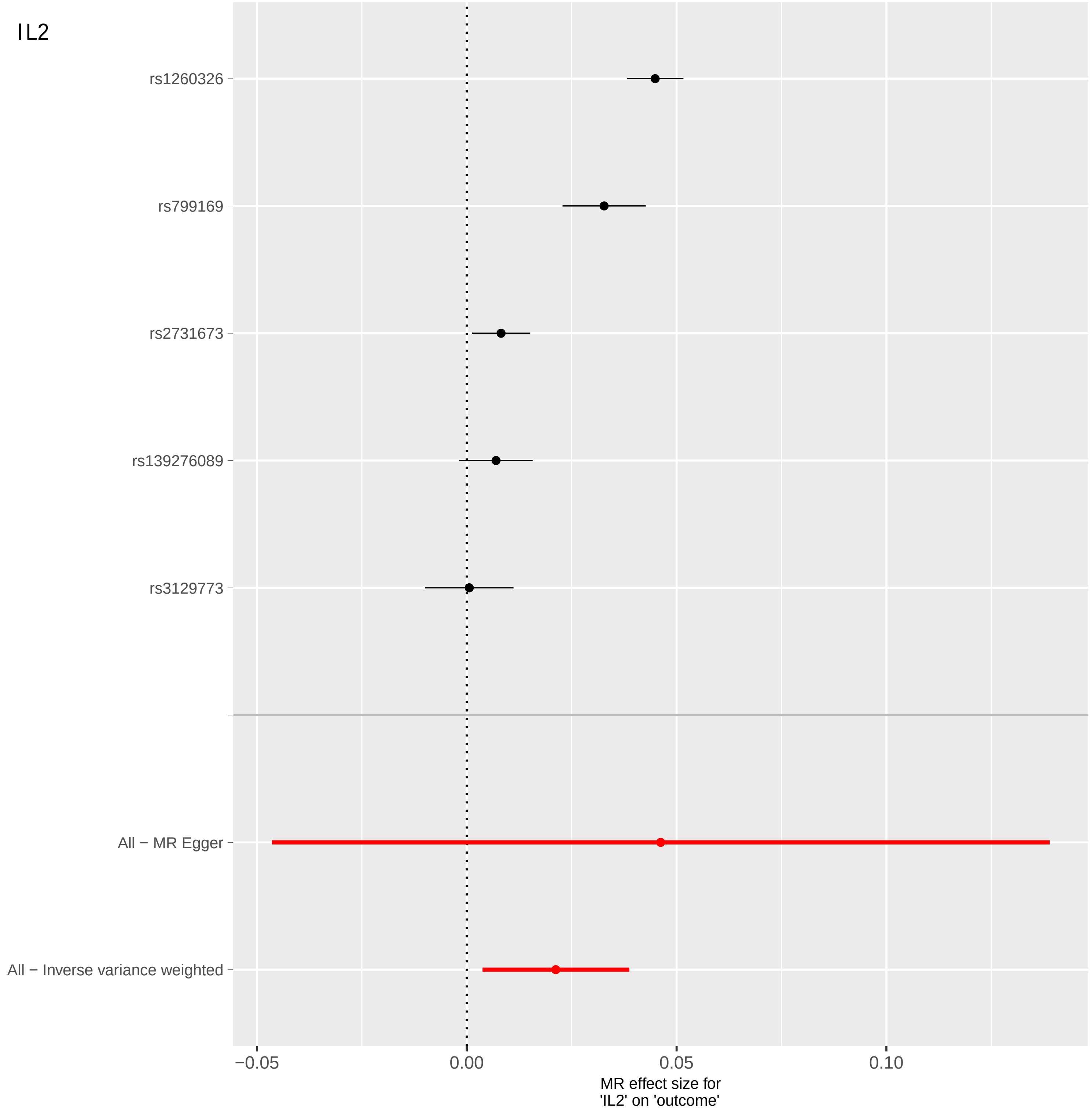
IL2
rs1260326
rs799169
rs2731673
rs139276089
rs3129773
All − MR Egger
All − Inverse variance weighted
−0.05
0.00
0.05
0.10
MR effect size for
'IL2' on 'outcome'

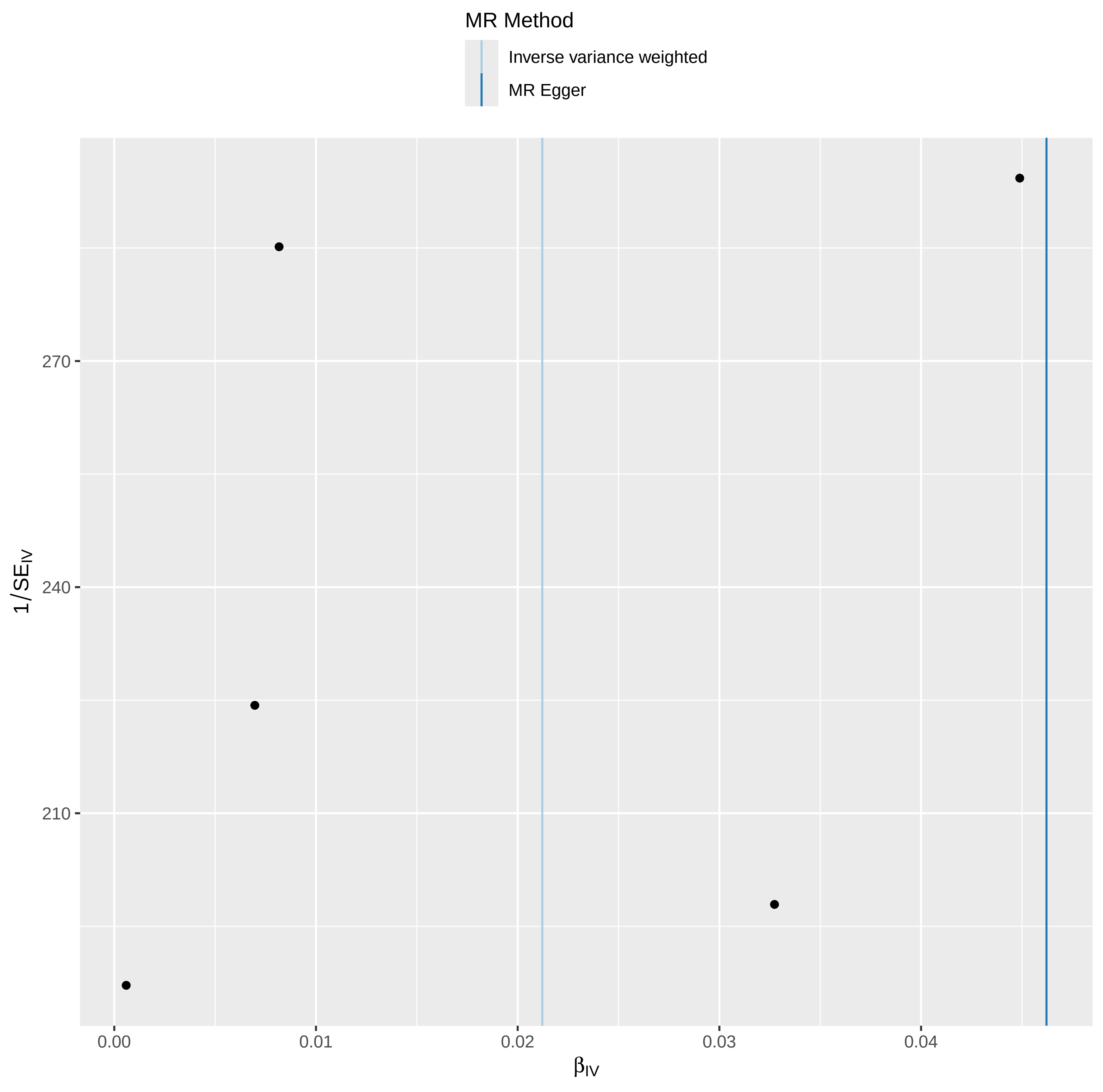

MR Method
Inverse variance weighted
MR Egger
270
240
210
1/SE_IV
0.00
0.01
0.02
0.03
0.04
β_IV

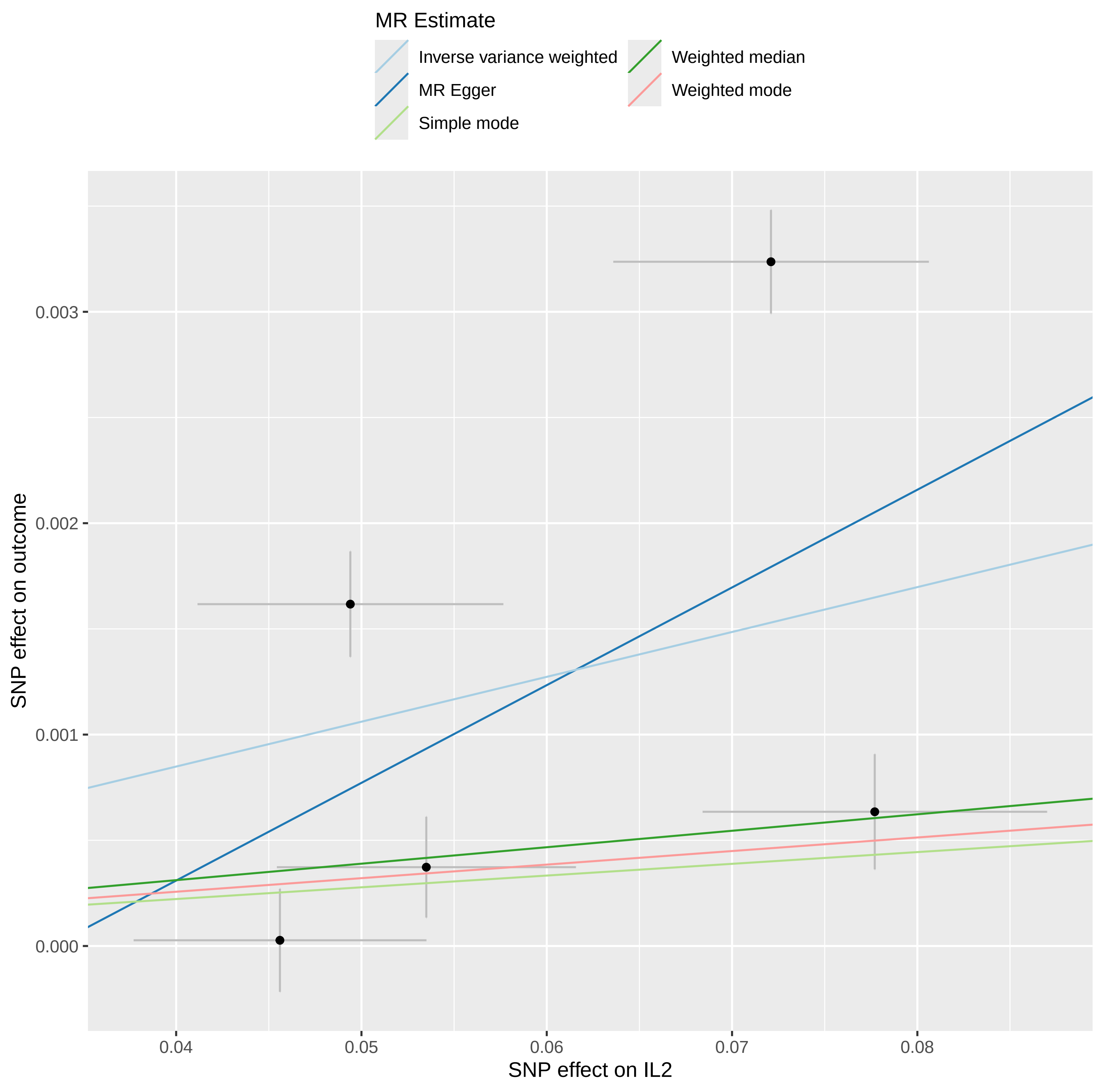
MR Estimate
Inverse variance weighted
MR Egger
Simple mode
Weighted median
Weighted mode
SNP effect on outcome
0.003
0.002
0.001
0.000
0.04
0.05
0.06
0.07
0.08
SNP effect on IL2

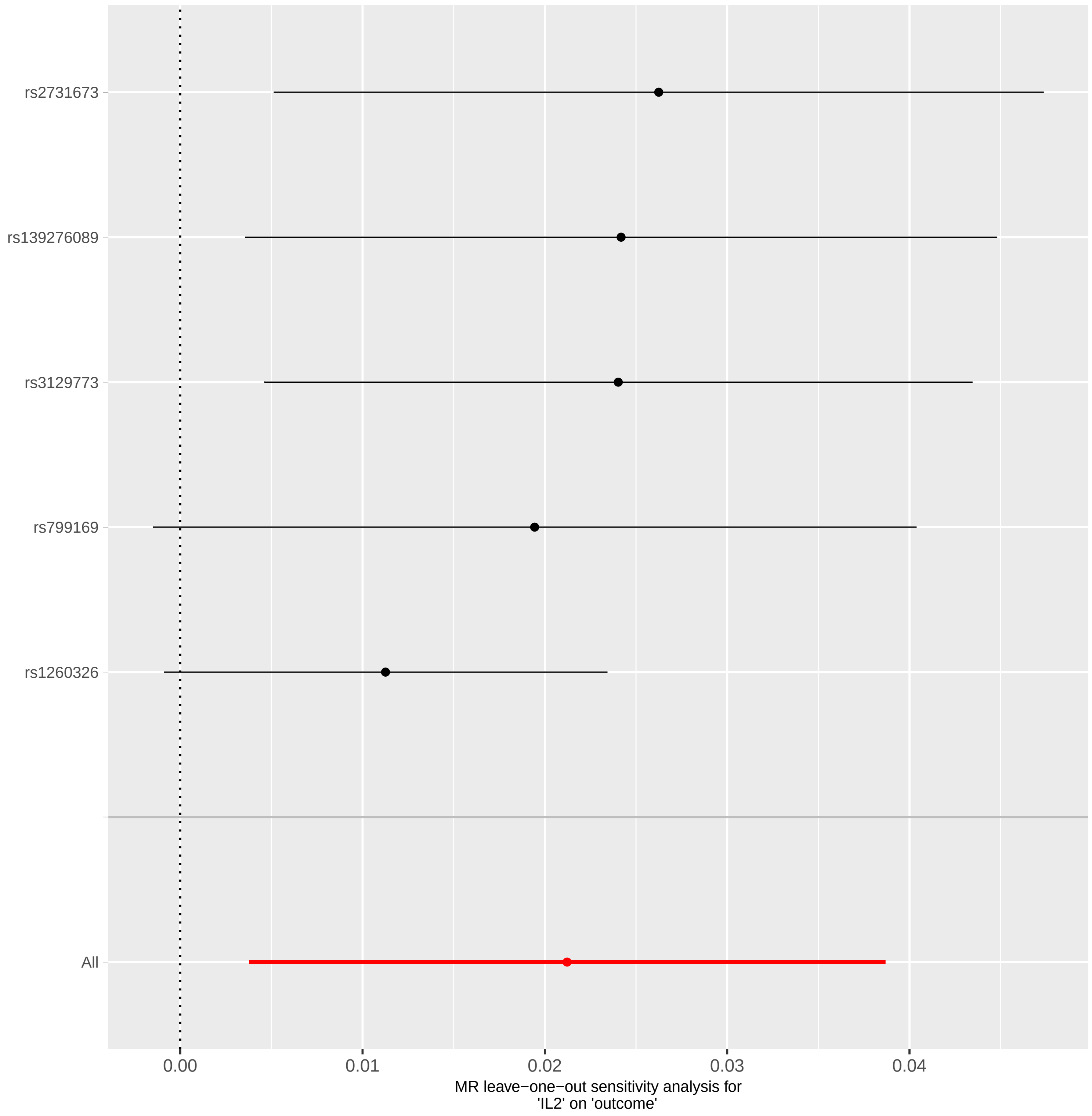

rs2731673
rs139276089
rs3129773
rs799169
rs1260326
All
0.00
0.01
0.02
0.03
0.04
MR leave−one−out sensitivity analysis for
'IL2' on 'outcome'

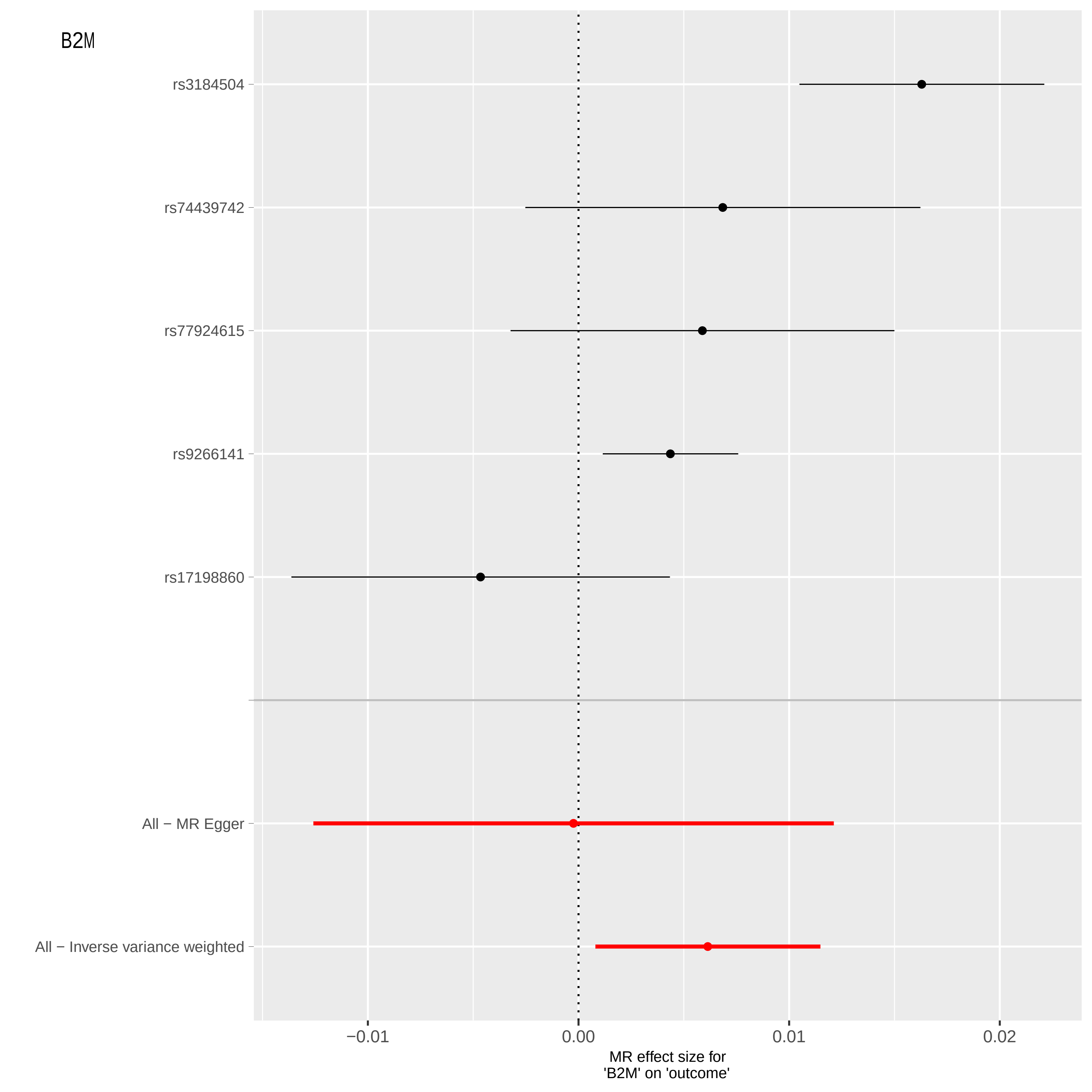
B2M
rs3184504
rs74439742
rs77924615
rs9266141
rs17198860
All − MR Egger
All − Inverse variance weighted
−0.01
0.00
0.01
0.02
MR effect size for
'B2M' on 'outcome'

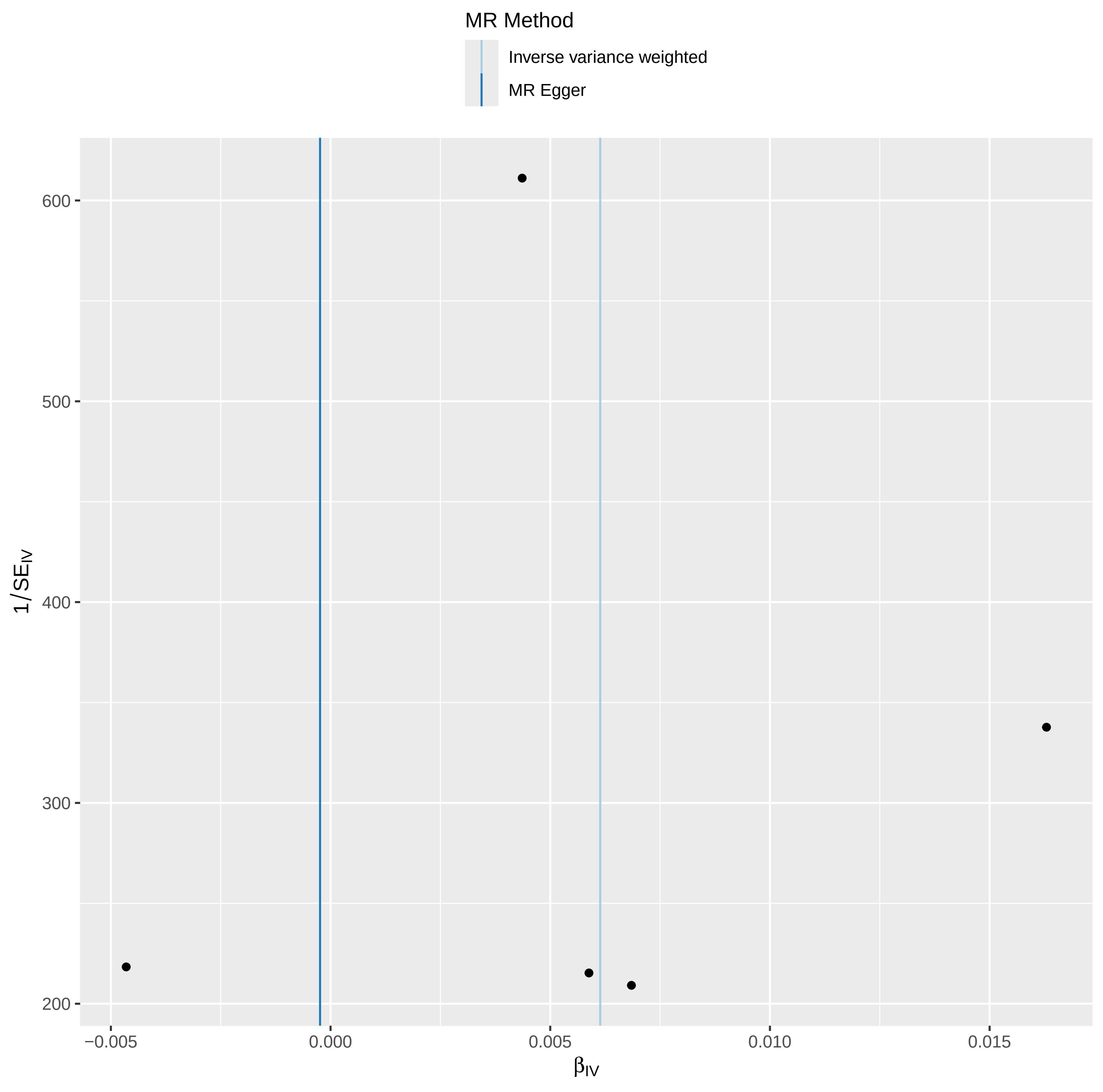

MR Method
Inverse variance weighted
MR Egger
600
500
400
300
200
1/SE$_{IV}$
−0.005
0.000
0.005
0.010
0.015
β_{IV}

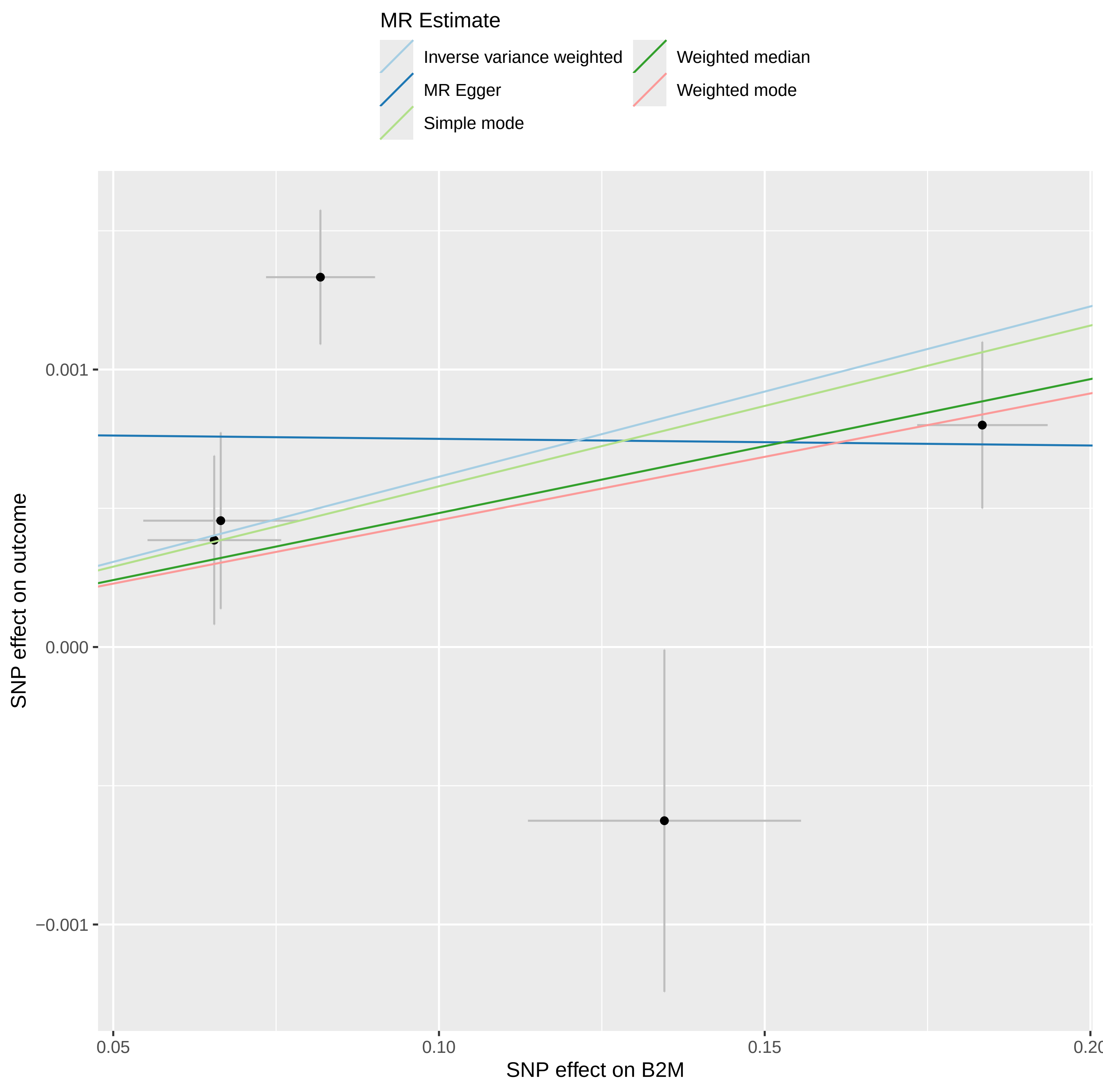
MR Estimate
Inverse variance weighted
MR Egger
Simple mode
Weighted median
Weighted mode
SNP effect on outcome
0.001
0.000
−0.001
0.05
0.10
0.15
0.20
SNP effect on B2M

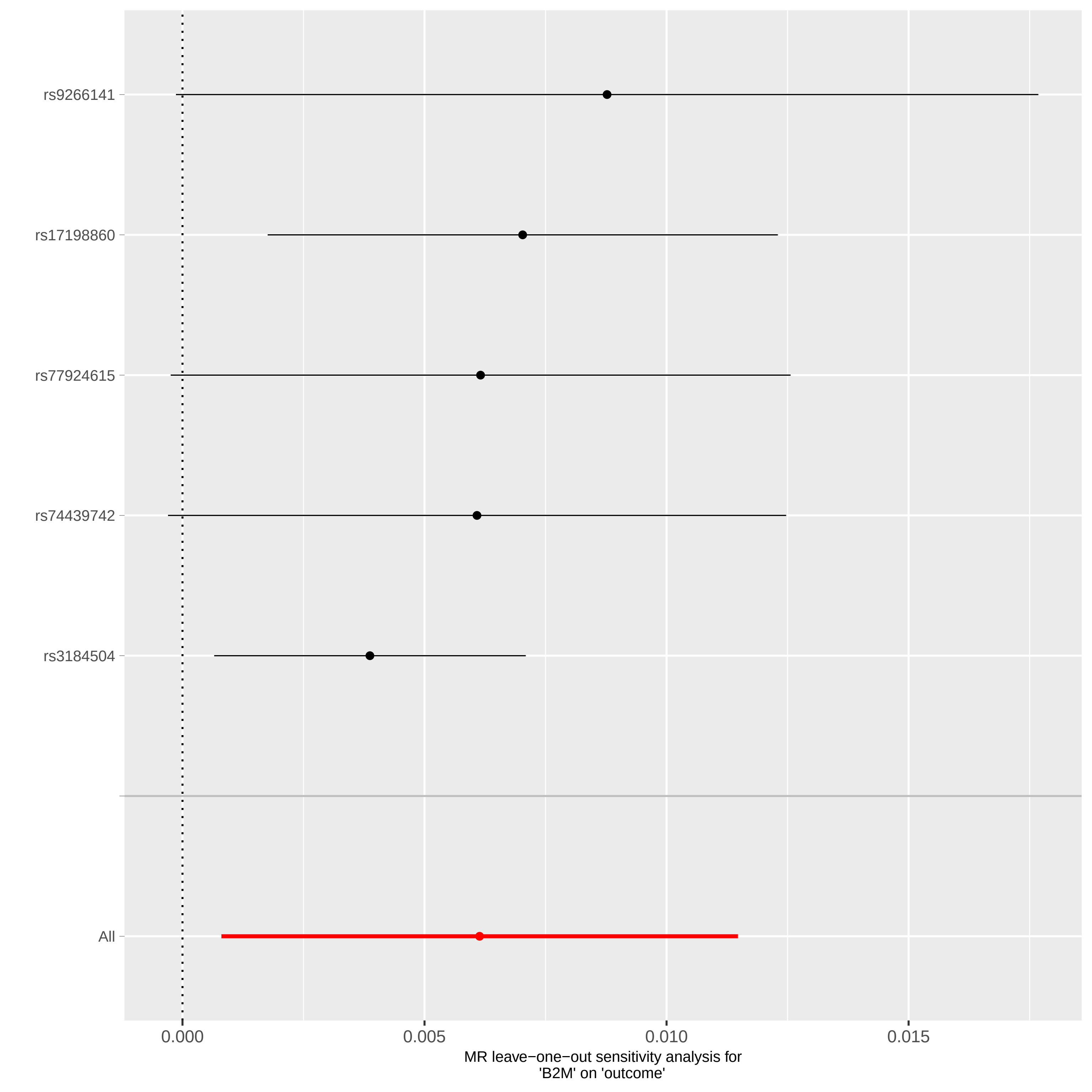

rs9266141
rs17198860
rs77924615
rs74439742
rs3184504
All
0.000
0.005
0.010
0.015
MR leave−one−out sensitivity analysis for
'B2M' on 'outcome'

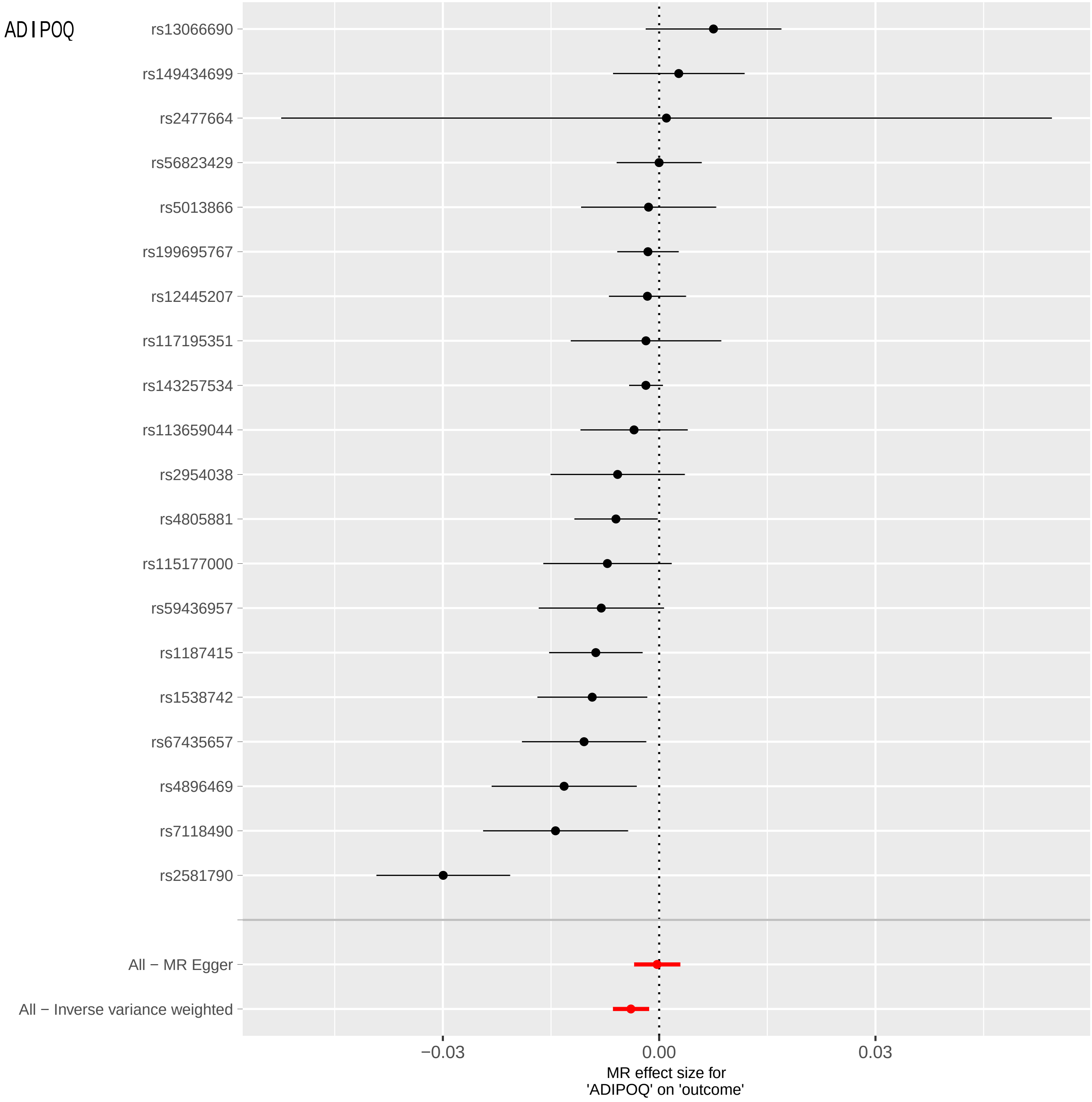
ADIPOQ
rs13066690
rs149434699
rs2477664
rs56823429
rs5013866
rs199695767
rs12445207
rs117195351
rs143257534
rs113659044
rs2954038
rs4805881
rs115177000
rs59436957
rs1187415
rs1538742
rs67435657
rs4896469
rs7118490
rs2581790
All − MR Egger
All − Inverse variance weighted
−0.03
0.00
0.03
MR effect size for
'ADIPOQ' on 'outcome'

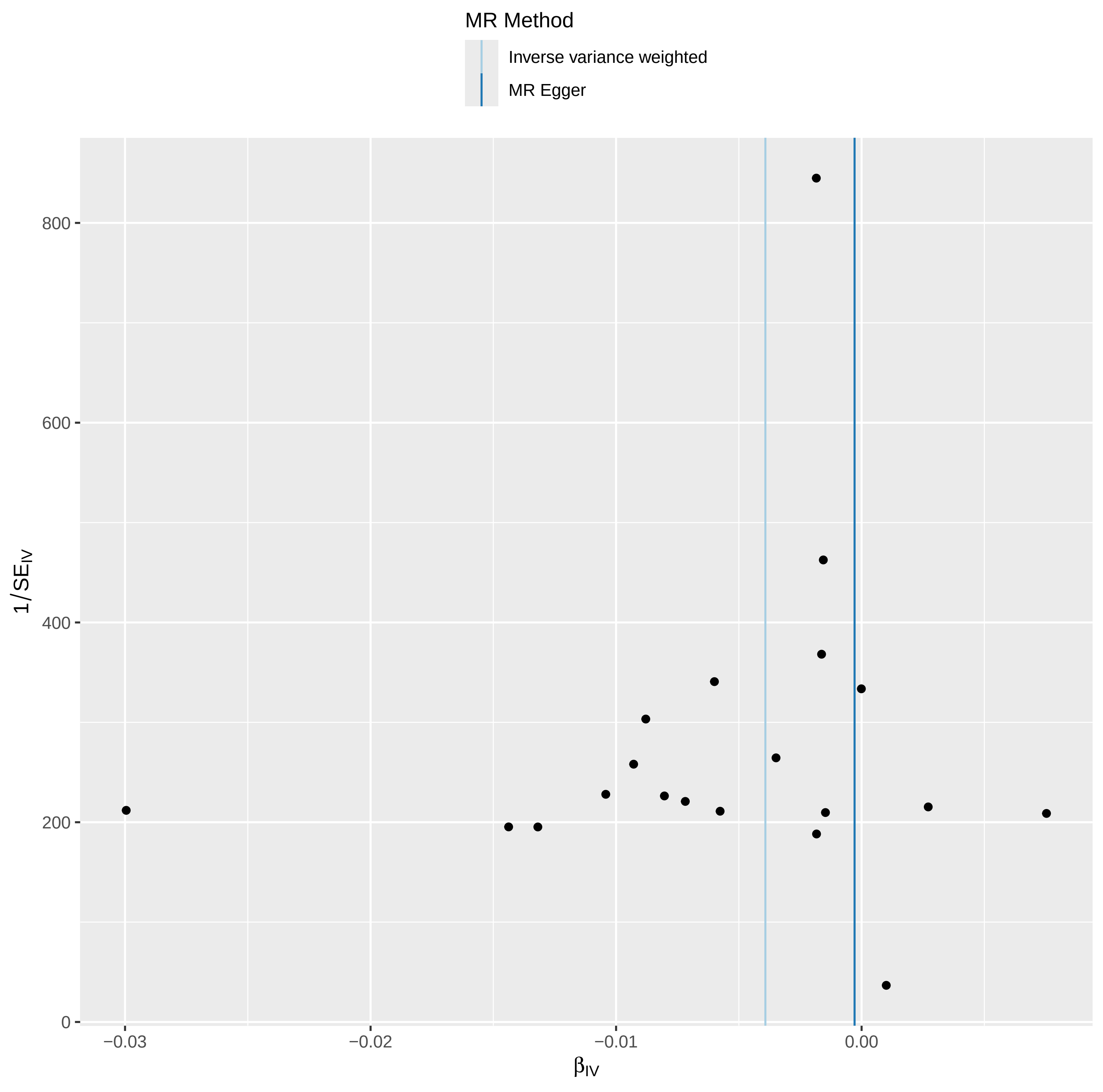
MR Method
Inverse variance weighted
MR Egger
800
600
400
200
0
1/SE_IV
−0.03
−0.02
−0.01
0.00
β_IV

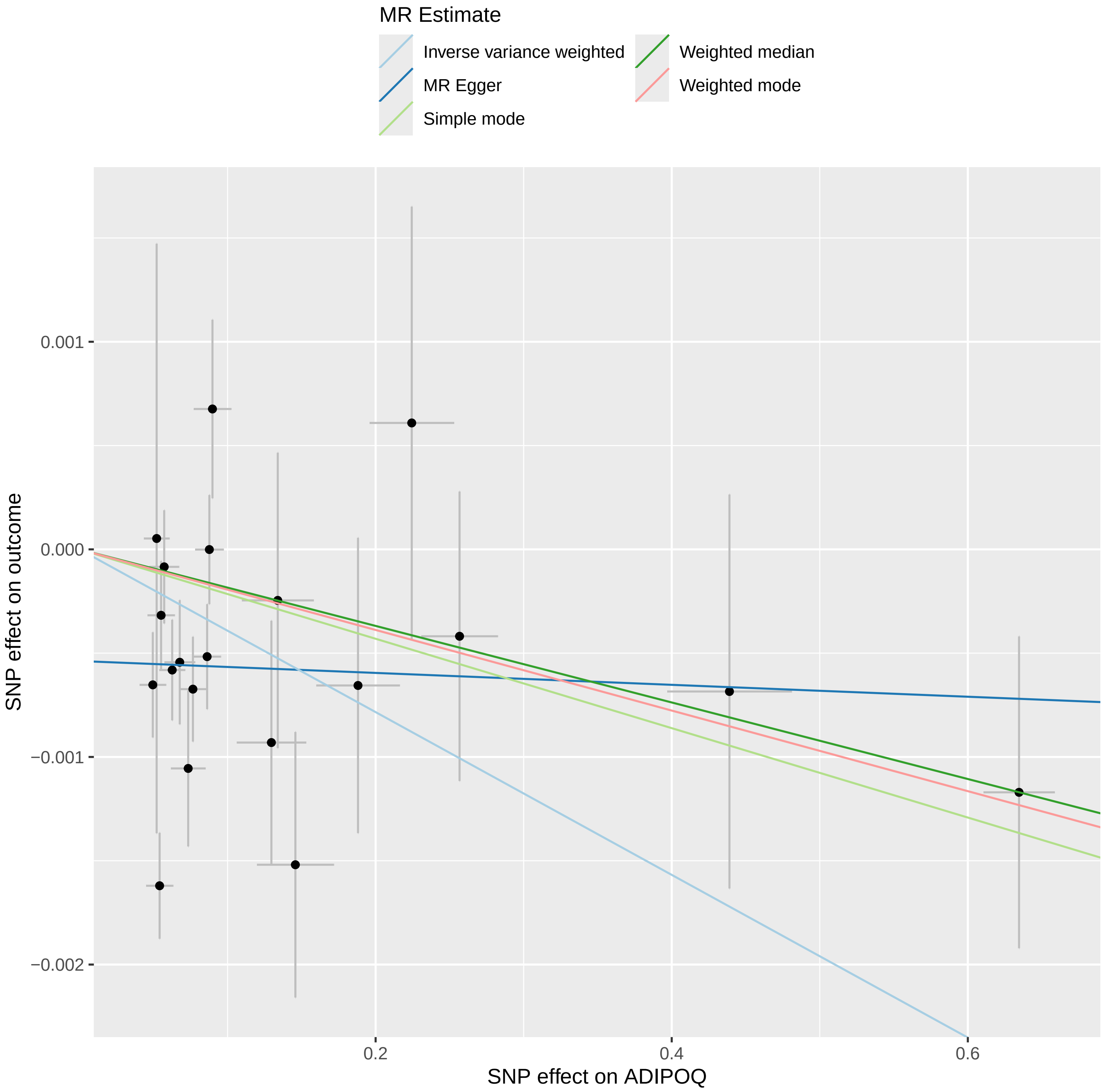
MR Estimate
Inverse variance weighted
MR Egger
Simple mode
Weighted median
Weighted mode
0.001
0.000
−0.001
−0.002
SNP effect on outcome
0.2
0.4
0.6
SNP effect on ADIPOQ

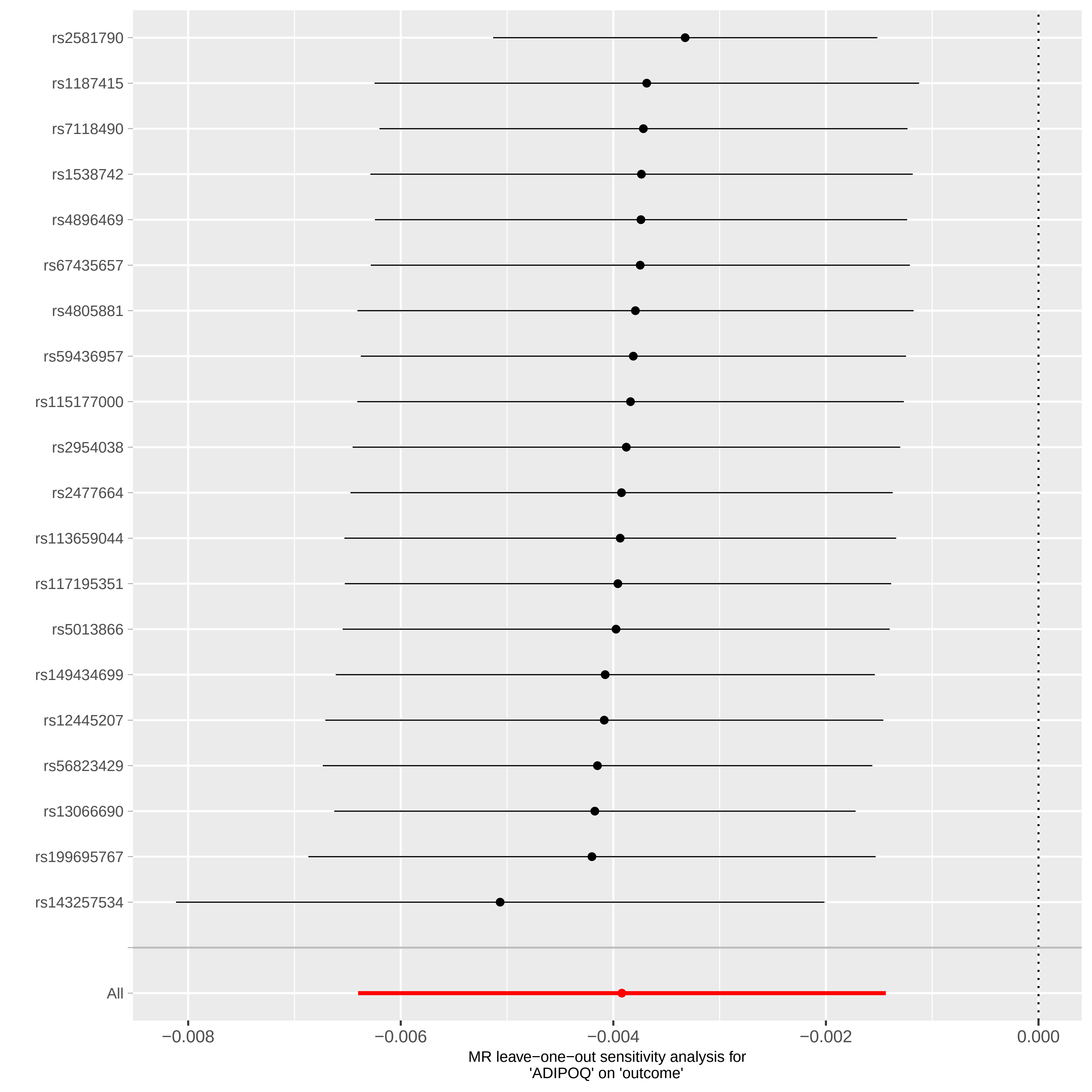

rs2581790
rs1187415
rs7118490
rs1538742
rs4896469
rs67435657
rs4805881
rs59436957
rs115177000
rs2954038
rs2477664
rs113659044
rs117195351
rs5013866
rs149434699
rs12445207
rs56823429
rs13066690
rs199695767
rs143257534
All
−0.008
−0.006
−0.004
−0.002
0.000
MR leave−one−out sensitivity analysis for
'ADIPOQ' on 'outcome'

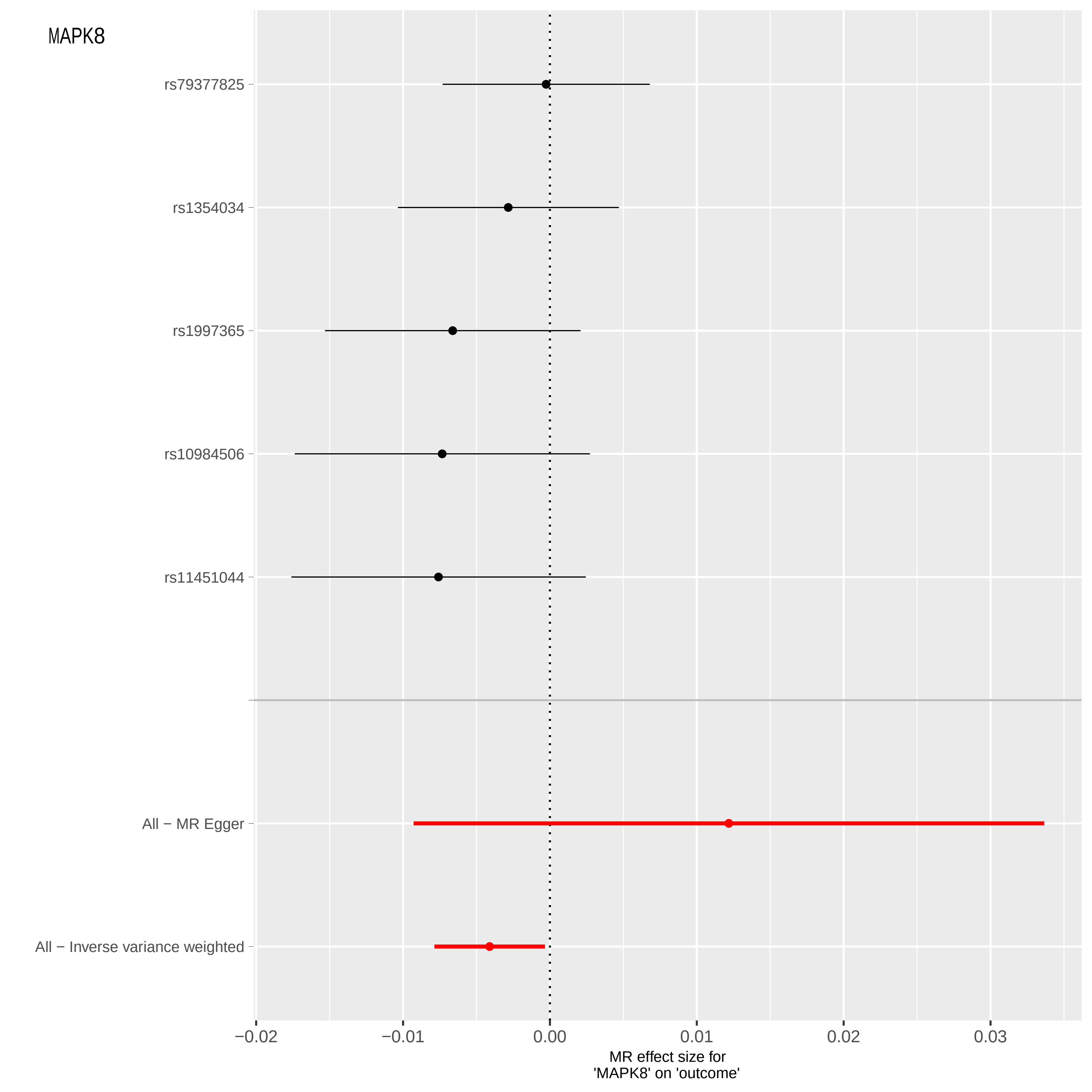

MAPK8
rs79377825
rs1354034
rs1997365
rs10984506
rs11451044
All − MR Egger
All − Inverse variance weighted
−0.02
−0.01
0.00
0.01
0.02
0.03
MR effect size for
'MAPK8' on 'outcome'

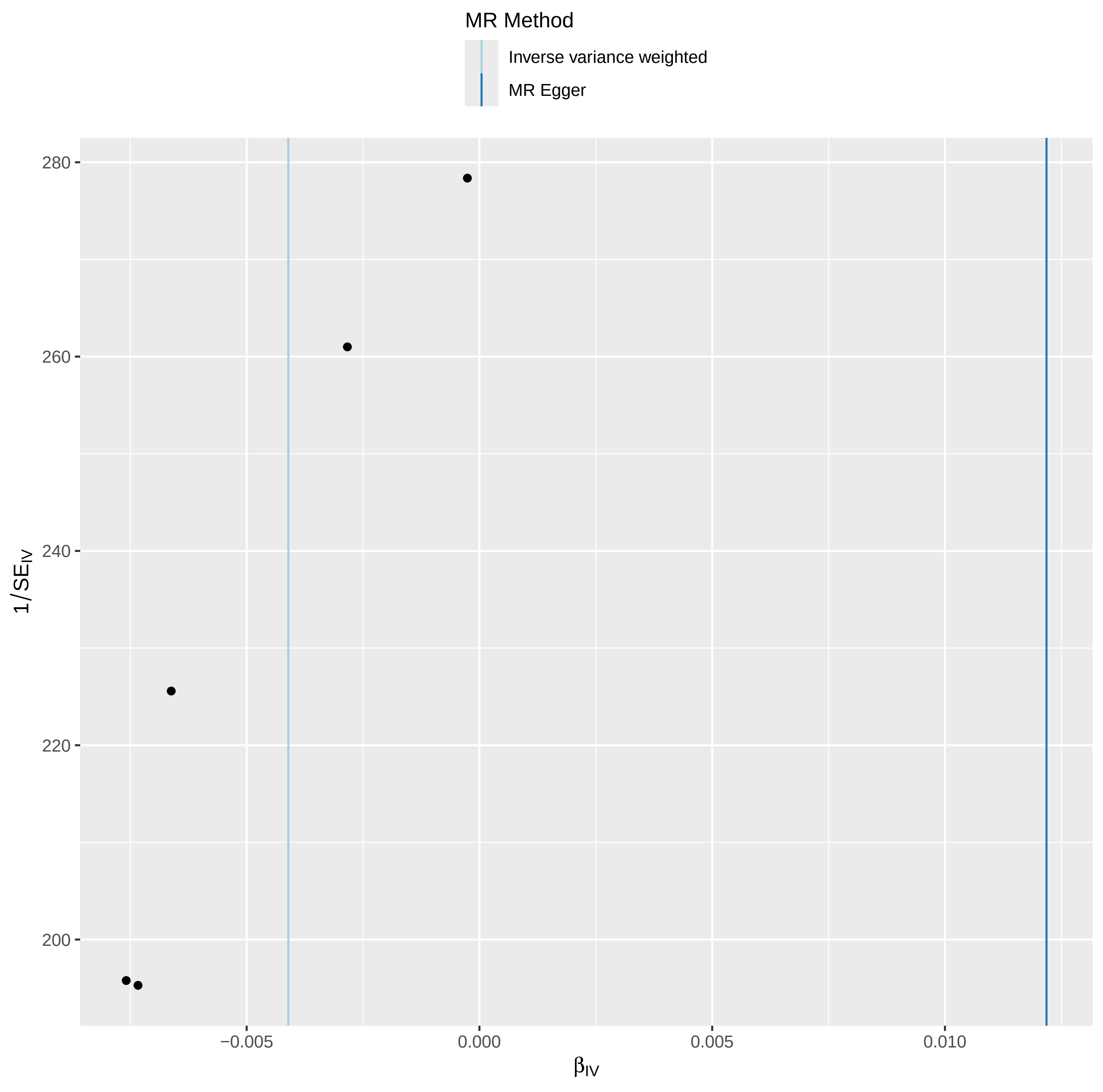

MR Method
Inverse variance weighted
MR Egger
280
260
240
220
200
1/SE$_{IV}$
−0.005
0.000
0.005
0.010
β$_{IV}$

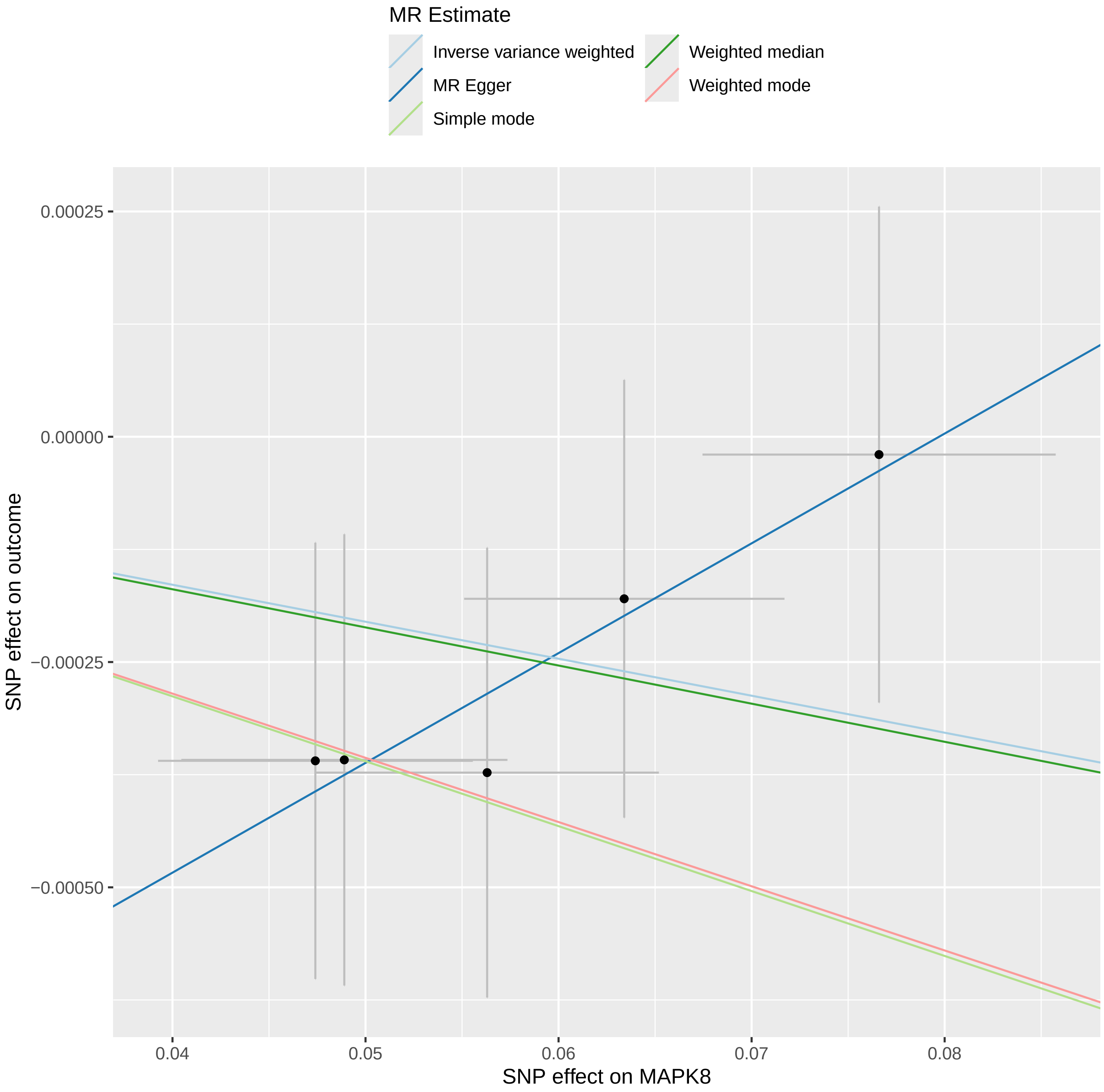

MR Estimate
Inverse variance weighted
MR Egger
Simple mode
Weighted median
Weighted mode
0.00025
0.00000
−0.00025
−0.00050
0.04
0.05
0.06
0.07
0.08
SNP effect on MAPK8
SNP effect on outcome

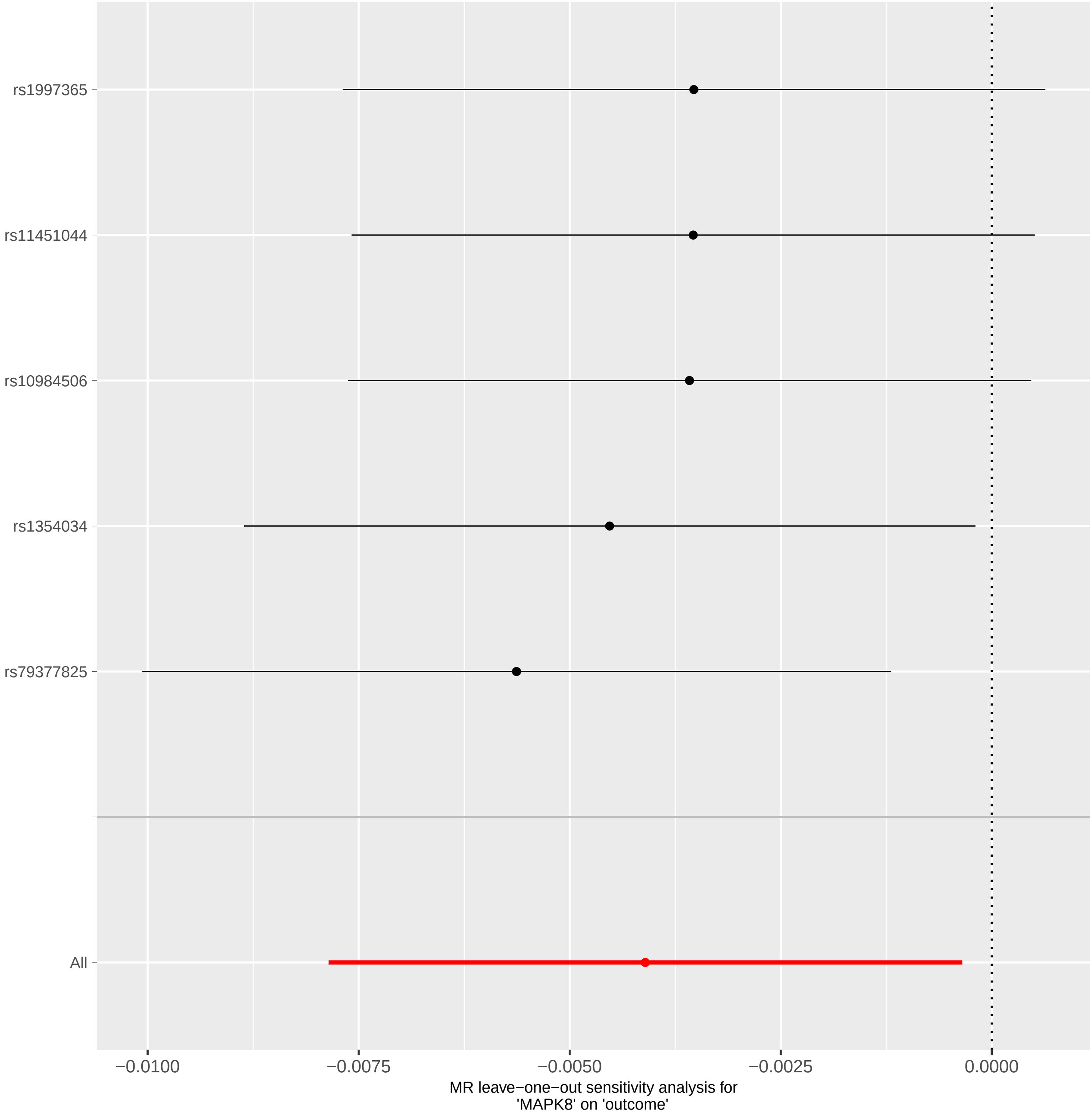

rs1997365
rs11451044
rs10984506
rs1354034
rs79377825
All
−0.0100
−0.0075
−0.0050
−0.0025
0.0000
MR leave−one−out sensitivity analysis for
'MAPK8' on 'outcome'

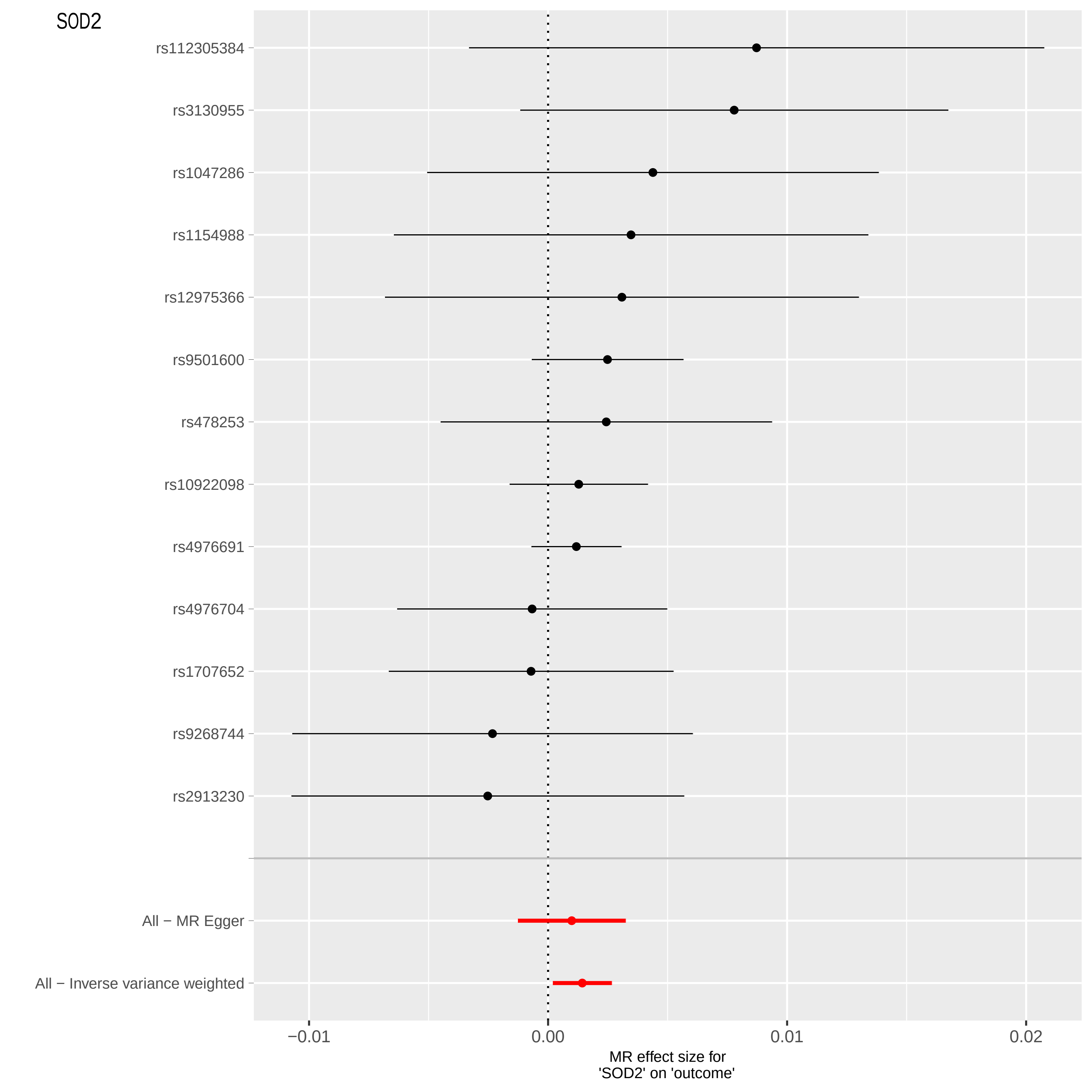
SOD2
rs112305384
rs3130955
rs1047286
rs1154988
rs12975366
rs9501600
rs478253
rs10922098
rs4976691
rs4976704
rs1707652
rs9268744
rs2913230
All − MR Egger
All − Inverse variance weighted
−0.01
0.00
0.01
0.02
MR effect size for
'SOD2' on 'outcome'

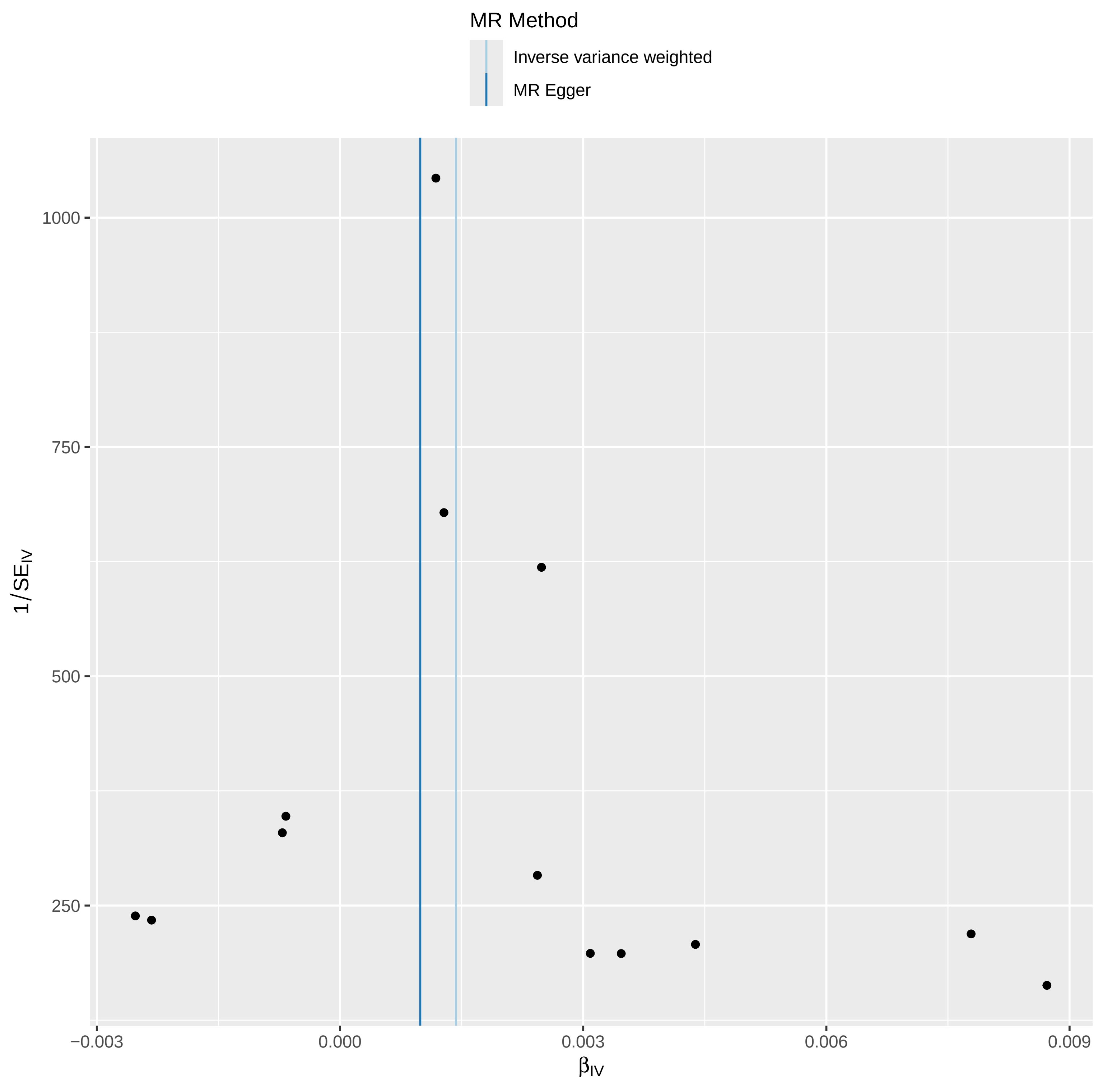
MR Method
Inverse variance weighted
MR Egger
1000
750
500
250
1/SE_IV
−0.003
0.000
0.003
0.006
0.009
β_IV

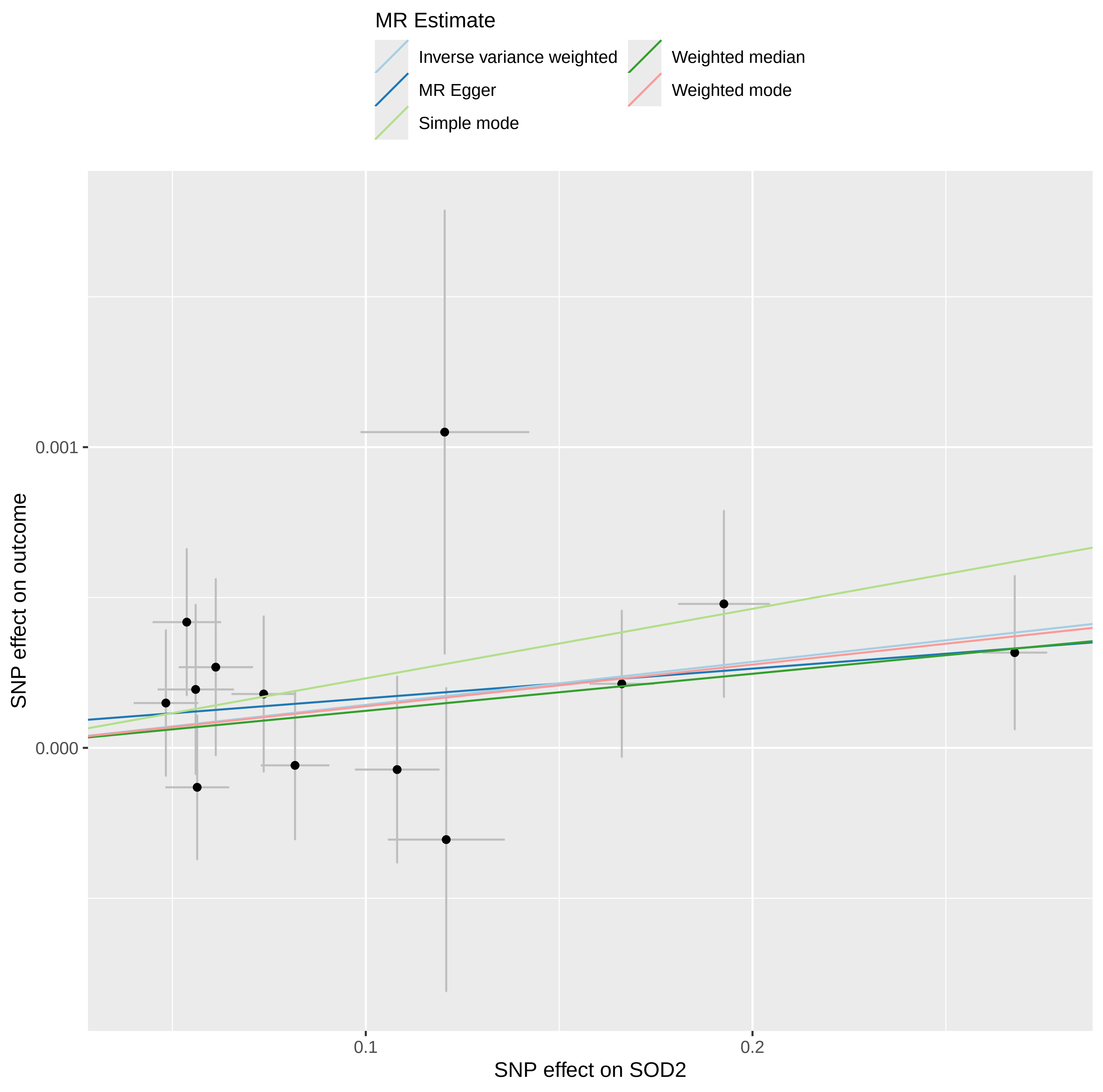
MR Estimate
Inverse variance weighted
MR Egger
Simple mode
Weighted median
Weighted mode
0.001
0.000
SNP effect on outcome
0.1
0.2
SNP effect on SOD2

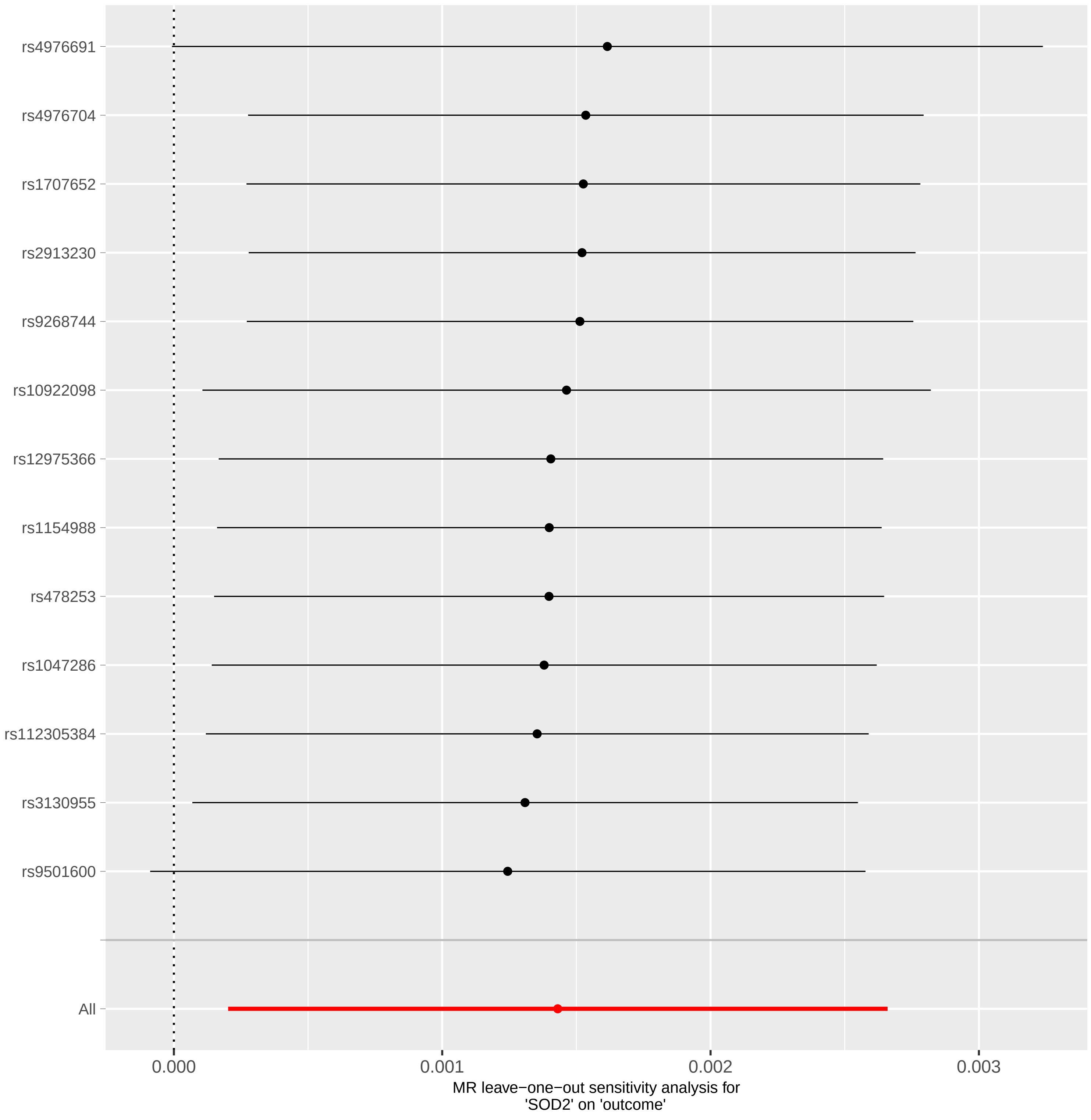
rs4976691
rs4976704
rs1707652
rs2913230
rs9268744
rs10922098
rs12975366
rs1154988
rs478253
rs1047286
rs112305384
rs3130955
rs9501600
All
0.000
0.001
0.002
0.003
MR leave−one−out sensitivity analysis for
'SOD2' on 'outcome'

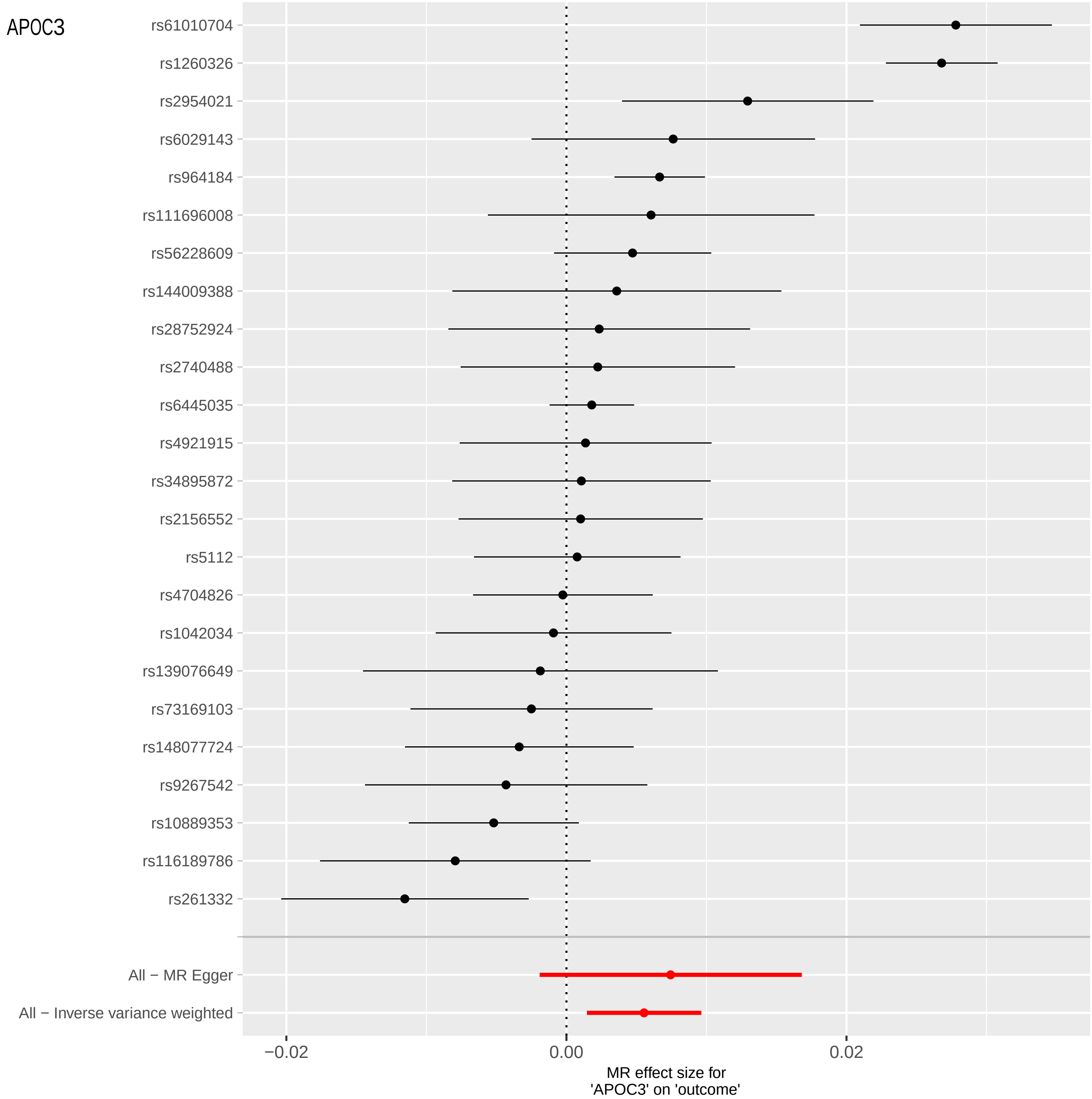

APOC3
rs61010704
rs1260326
rs2954021
rs6029143
rs964184
rs111696008
rs56228609
rs144009388
rs28752924
rs2740488
rs6445035
rs4921915
rs34895872
rs2156552
rs5112
rs4704826
rs1042034
rs139076649
rs73169103
rs148077724
rs9267542
rs10889353
rs116189786
rs261332
All − MR Egger
All − Inverse variance weighted
−0.02
0.00
0.02
MR effect size for
'APOC3' on 'outcome'

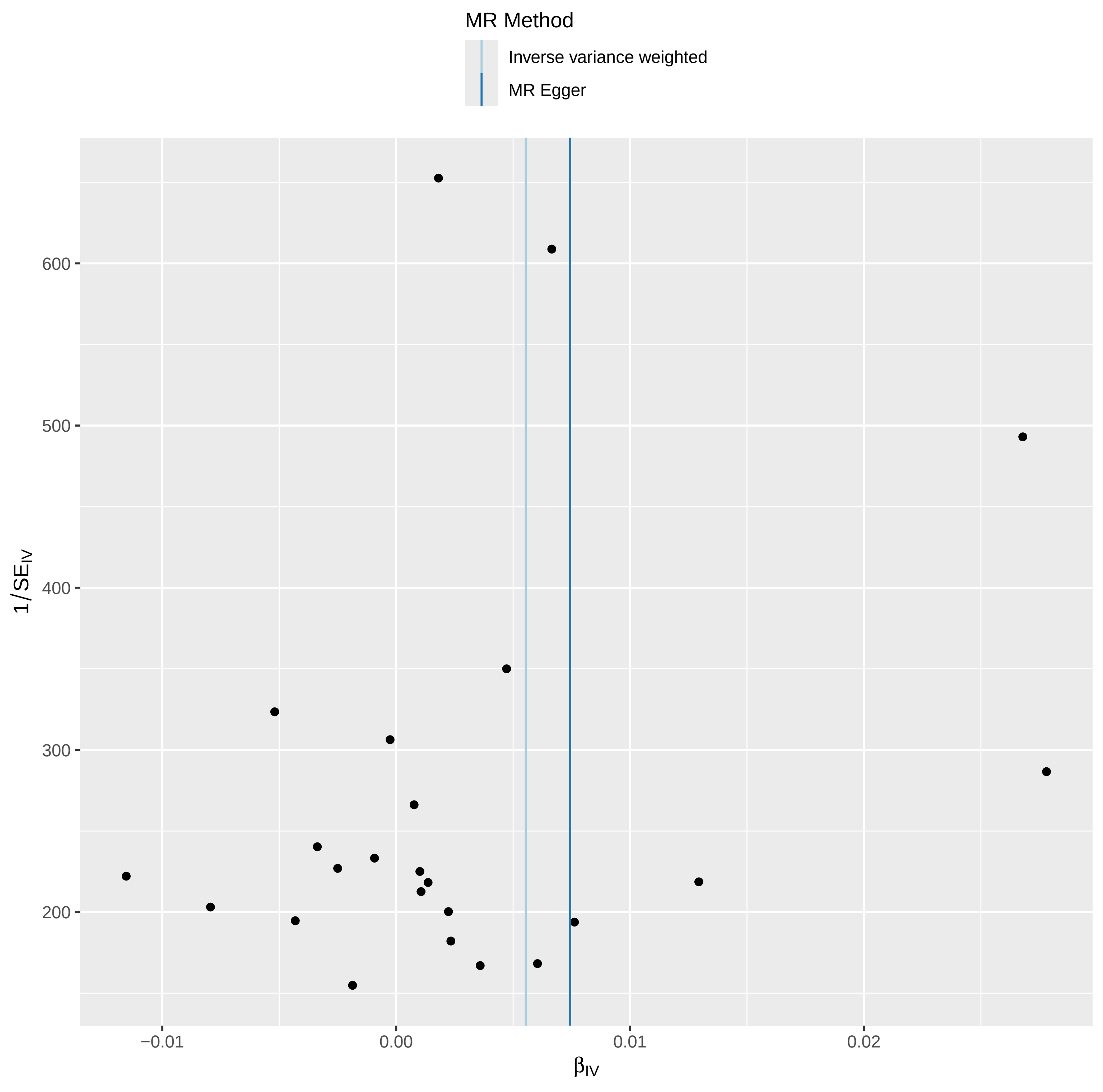
MR Method
Inverse variance weighted
MR Egger
600
500
400
300
200
1/SE_IV
−0.01
0.00
0.01
0.02
β_IV

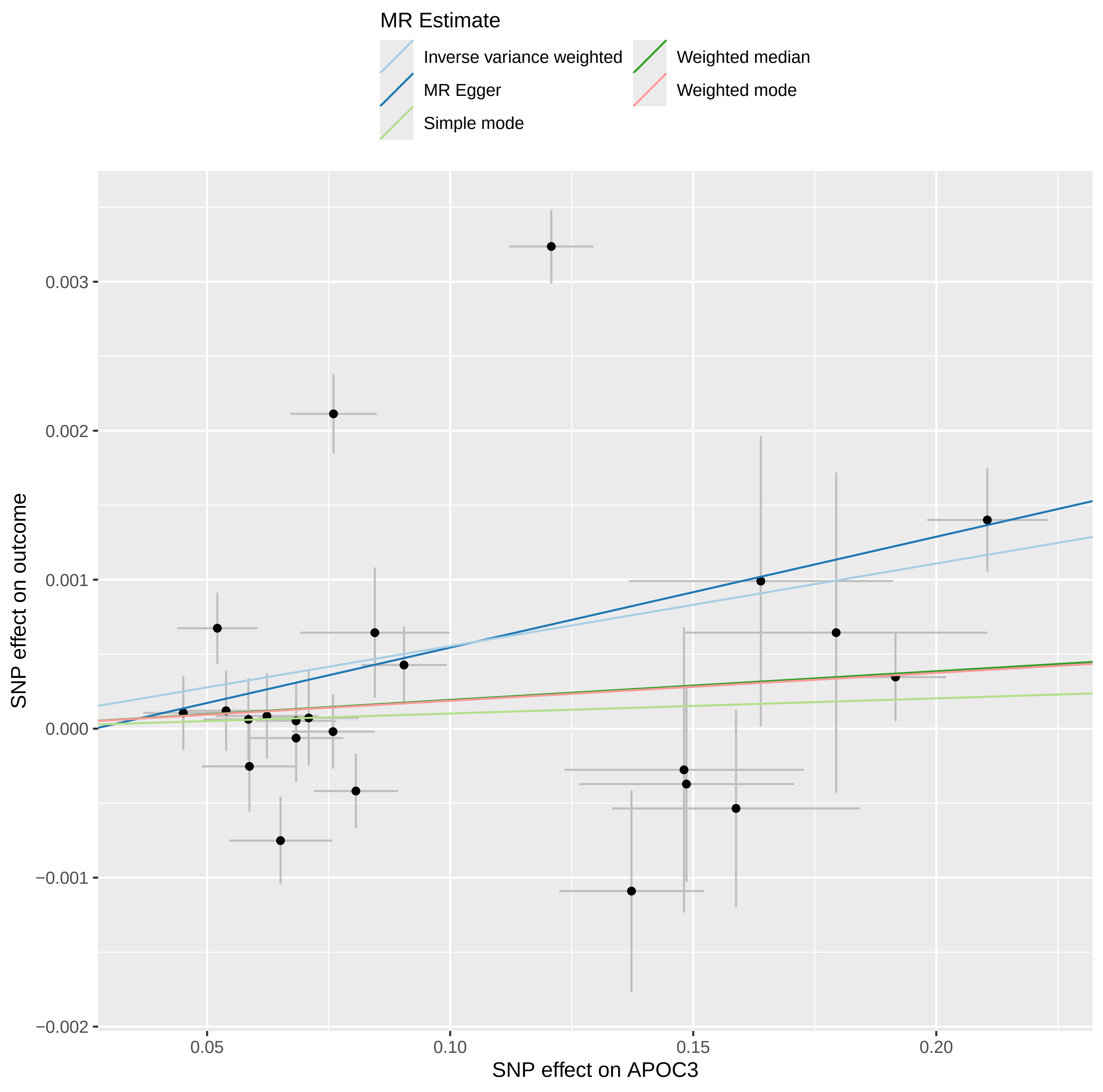
MR Estimate
Inverse variance weighted
MR Egger
Simple mode
Weighted median
Weighted mode
0.003
0.002
0.001
0.000
−0.001
−0.002
0.05
0.10
0.15
0.20
SNP effect on APOC3
SNP effect on outcome

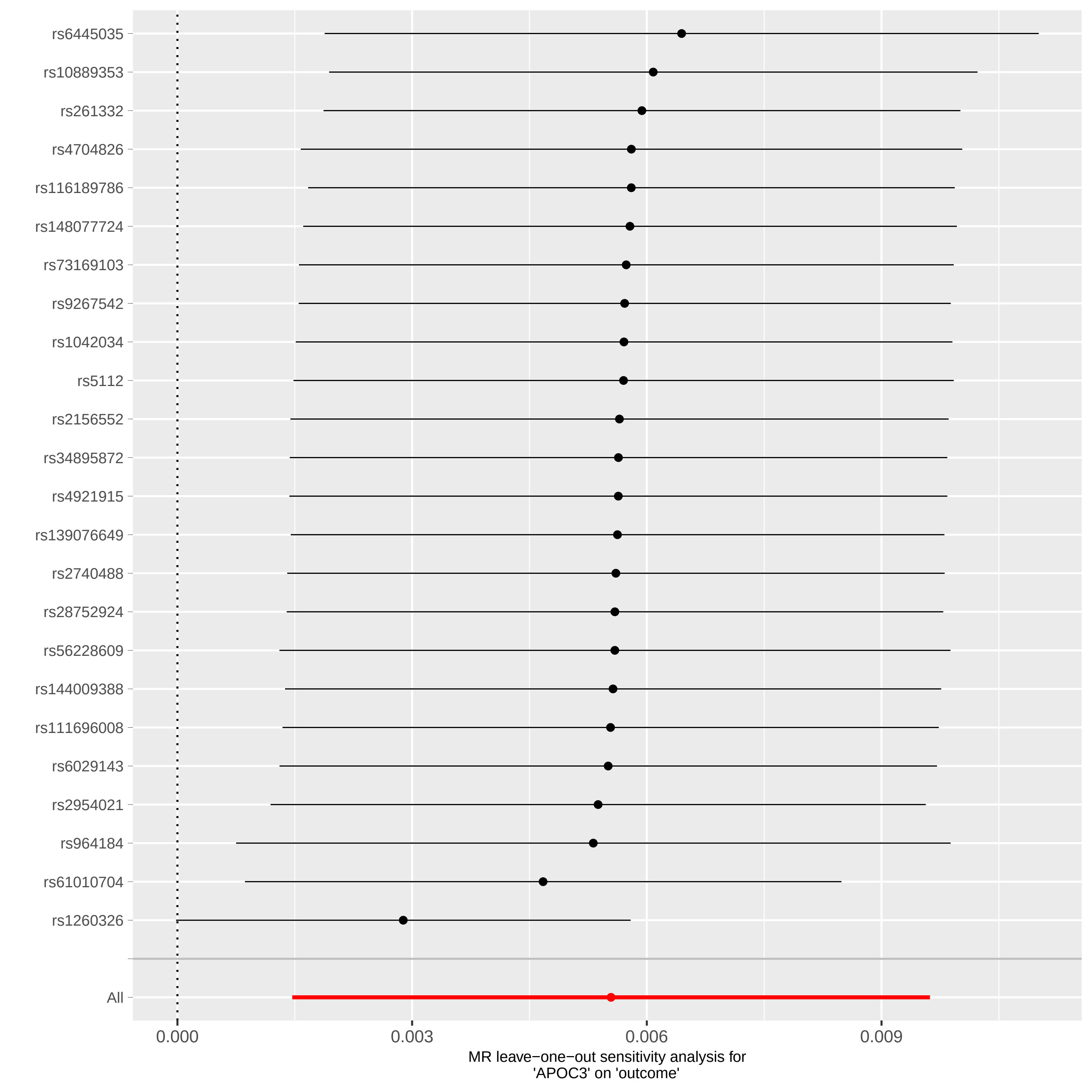

rs6445035
rs10889353
rs261332
rs4704826
rs116189786
rs148077724
rs73169103
rs9267542
rs1042034
rs5112
rs2156552
rs34895872
rs4921915
rs139076649
rs2740488
rs28752924
rs56228609
rs144009388
rs111696008
rs6029143
rs2954021
rs964184
rs61010704
rs1260326
All
0.000
0.003
0.006
0.009
MR leave−one−out sensitivity analysis for
'APOC3' on 'outcome'

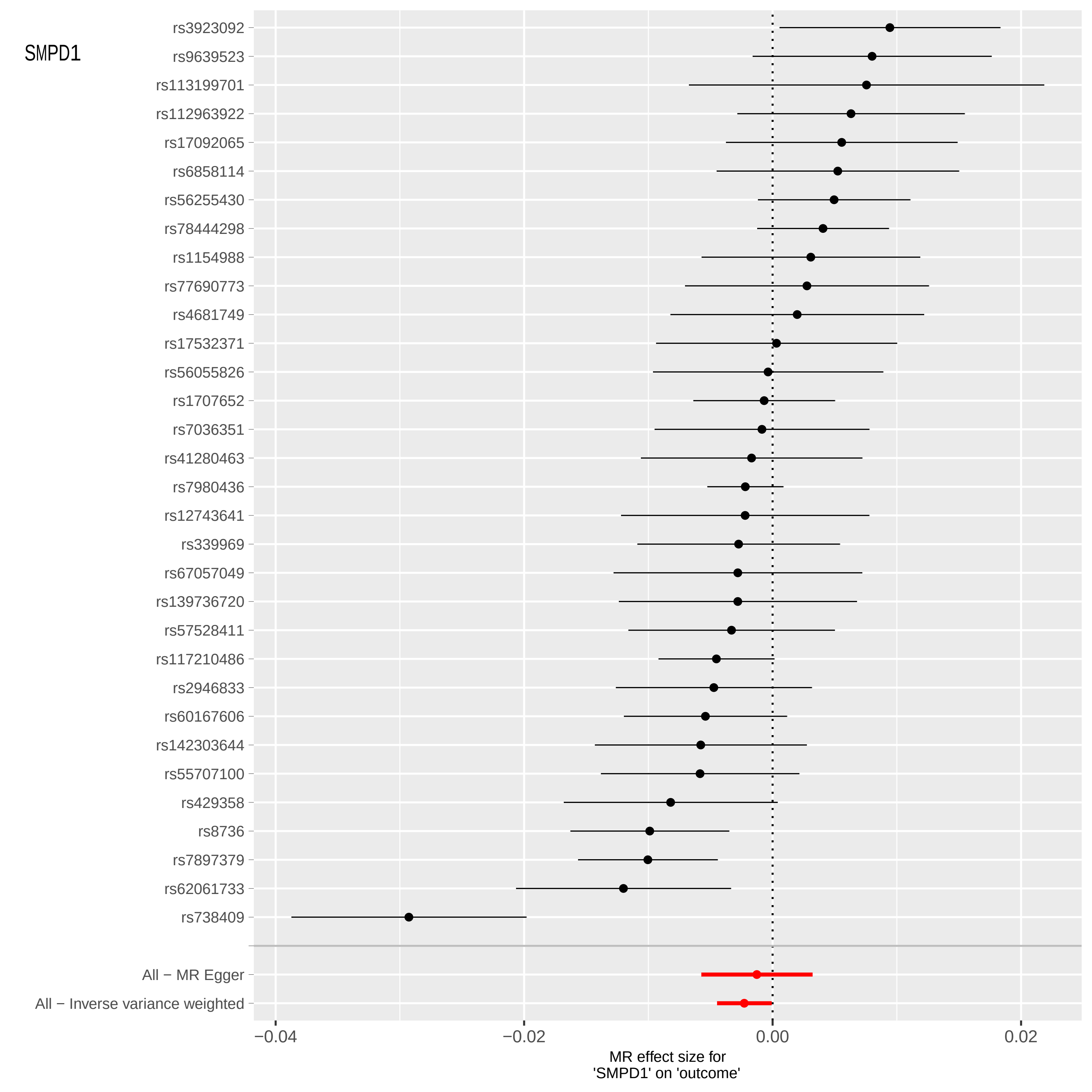
SMPD1
rs3923092
rs9639523
rs113199701
rs112963922
rs17092065
rs6858114
rs56255430
rs78444298
rs1154988
rs77690773
rs4681749
rs17532371
rs56055826
rs1707652
rs7036351
rs41280463
rs7980436
rs12743641
rs339969
rs67057049
rs139736720
rs57528411
rs117210486
rs2946833
rs60167606
rs142303644
rs55707100
rs429358
rs8736
rs7897379
rs62061733
rs738409
All − MR Egger
All − Inverse variance weighted
−0.04
−0.02
0.00
0.02
MR effect size for
'SMPD1' on 'outcome'

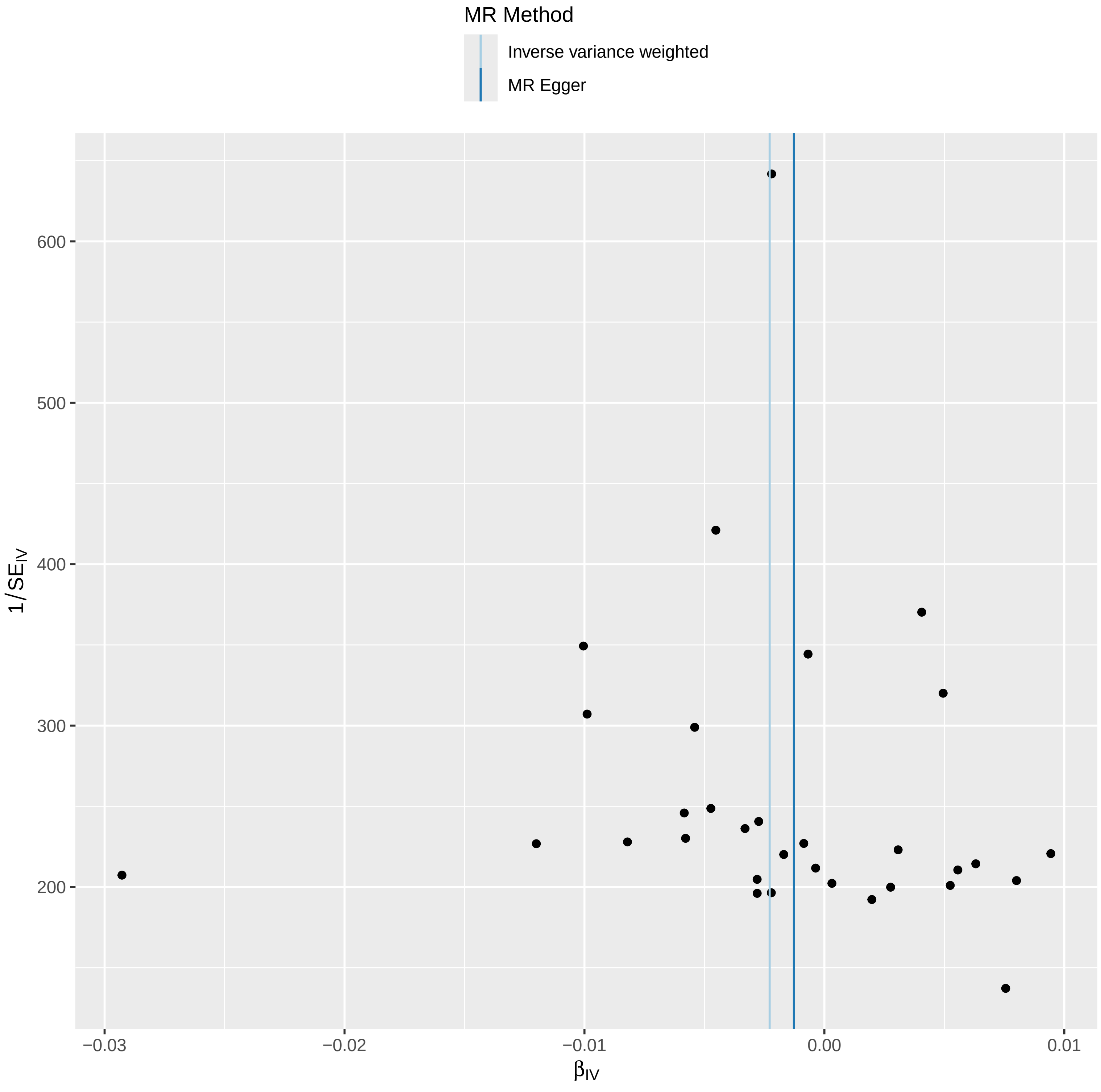
MR Method
Inverse variance weighted
MR Egger
600
500
400
300
200
1/SE_IV
−0.03
−0.02
−0.01
0.00
0.01
β_IV

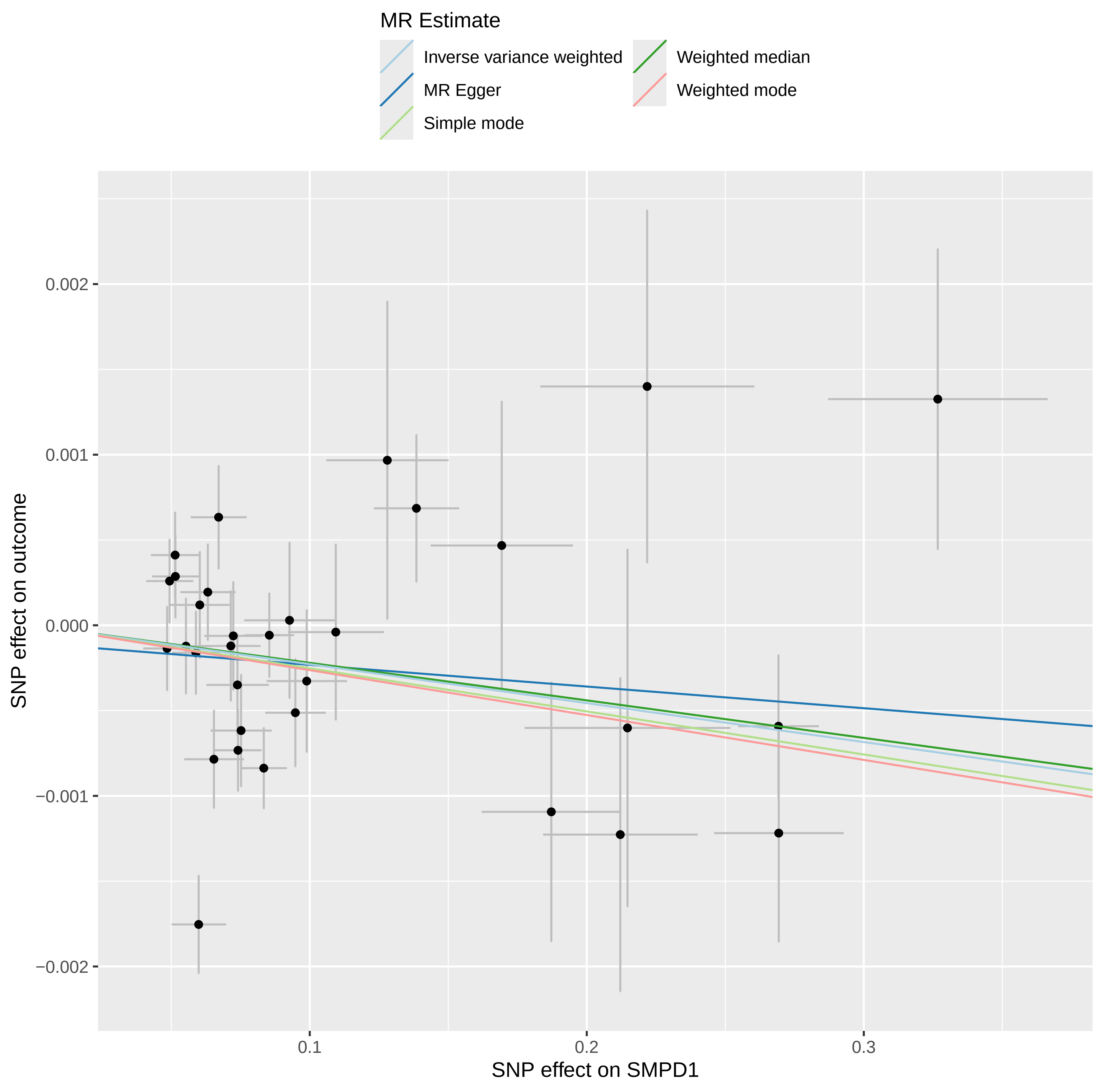

MR Estimate
Inverse variance weighted
MR Egger
Simple mode
Weighted median
Weighted mode
0.002
0.001
0.000
−0.001
−0.002
0.1
0.2
0.3
SNP effect on outcome
SNP effect on SMPD1

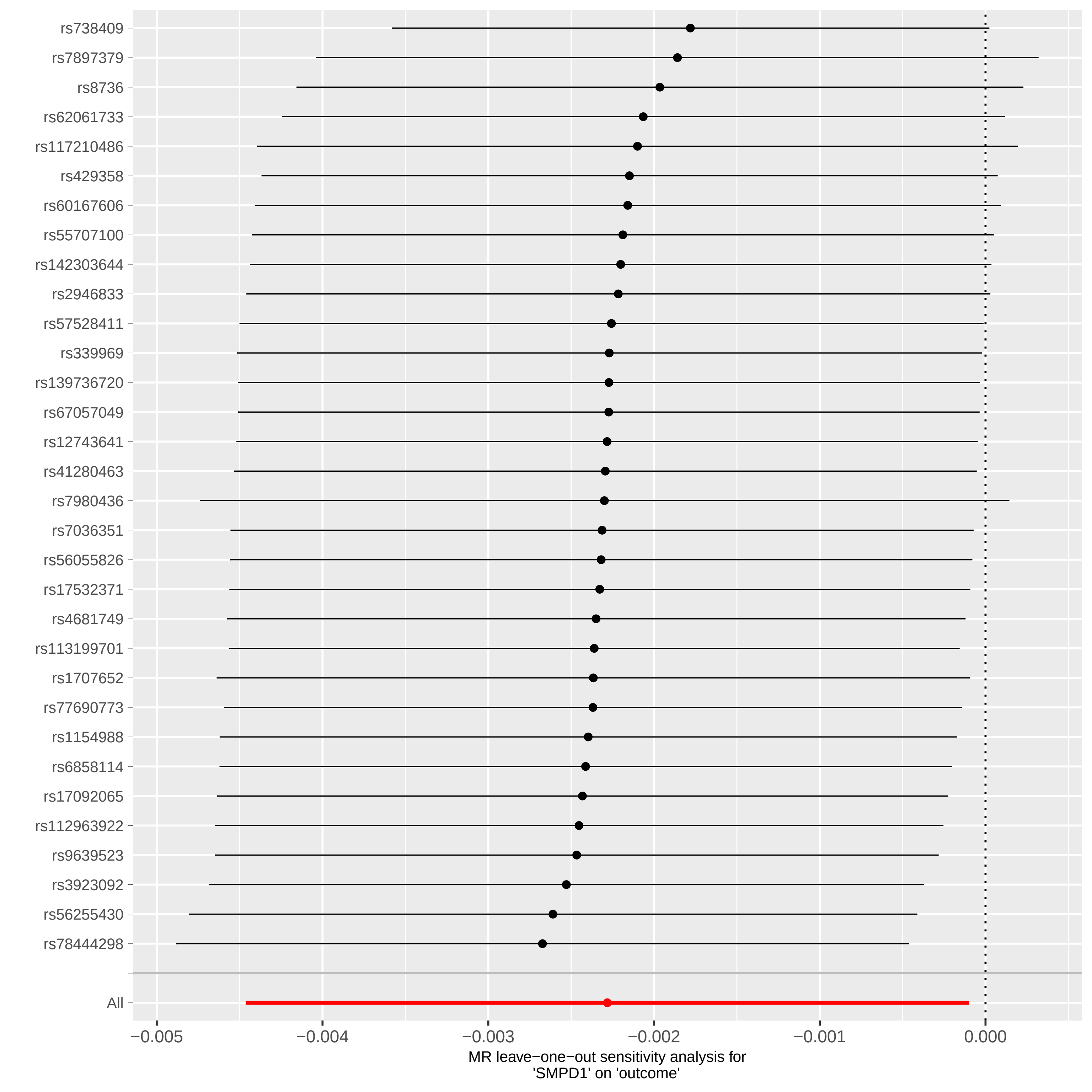
rs738409
rs7897379
rs8736
rs62061733
rs117210486
rs429358
rs60167606
rs55707100
rs142303644
rs2946833
rs57528411
rs339969
rs139736720
rs67057049
rs12743641
rs41280463
rs7980436
rs7036351
rs56055826
rs17532371
rs4681749
rs113199701
rs1707652
rs77690773
rs1154988
rs6858114
rs17092065
rs112963922
rs9639523
rs3923092
rs56255430
rs78444298
All
−0.005
−0.004
−0.003
−0.002
−0.001
0.000
MR leave−one−out sensitivity analysis for
'SMPD1' on 'outcome'

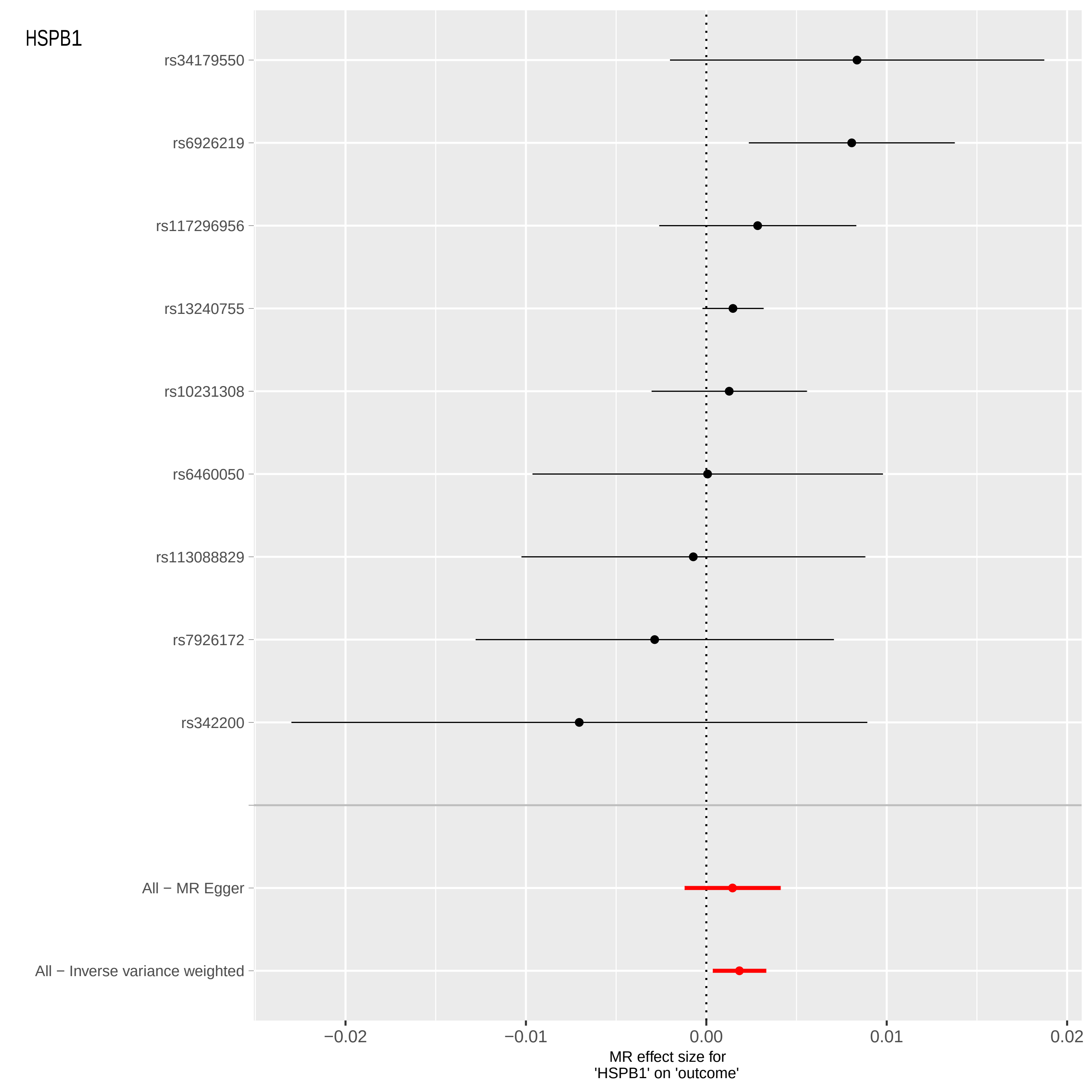

HSPB1
rs34179550
rs6926219
rs117296956
rs13240755
rs10231308
rs6460050
rs113088829
rs7926172
rs342200
All − MR Egger
All − Inverse variance weighted
−0.02
−0.01
0.00
0.01
0.02
MR effect size for
'HSPB1' on 'outcome'

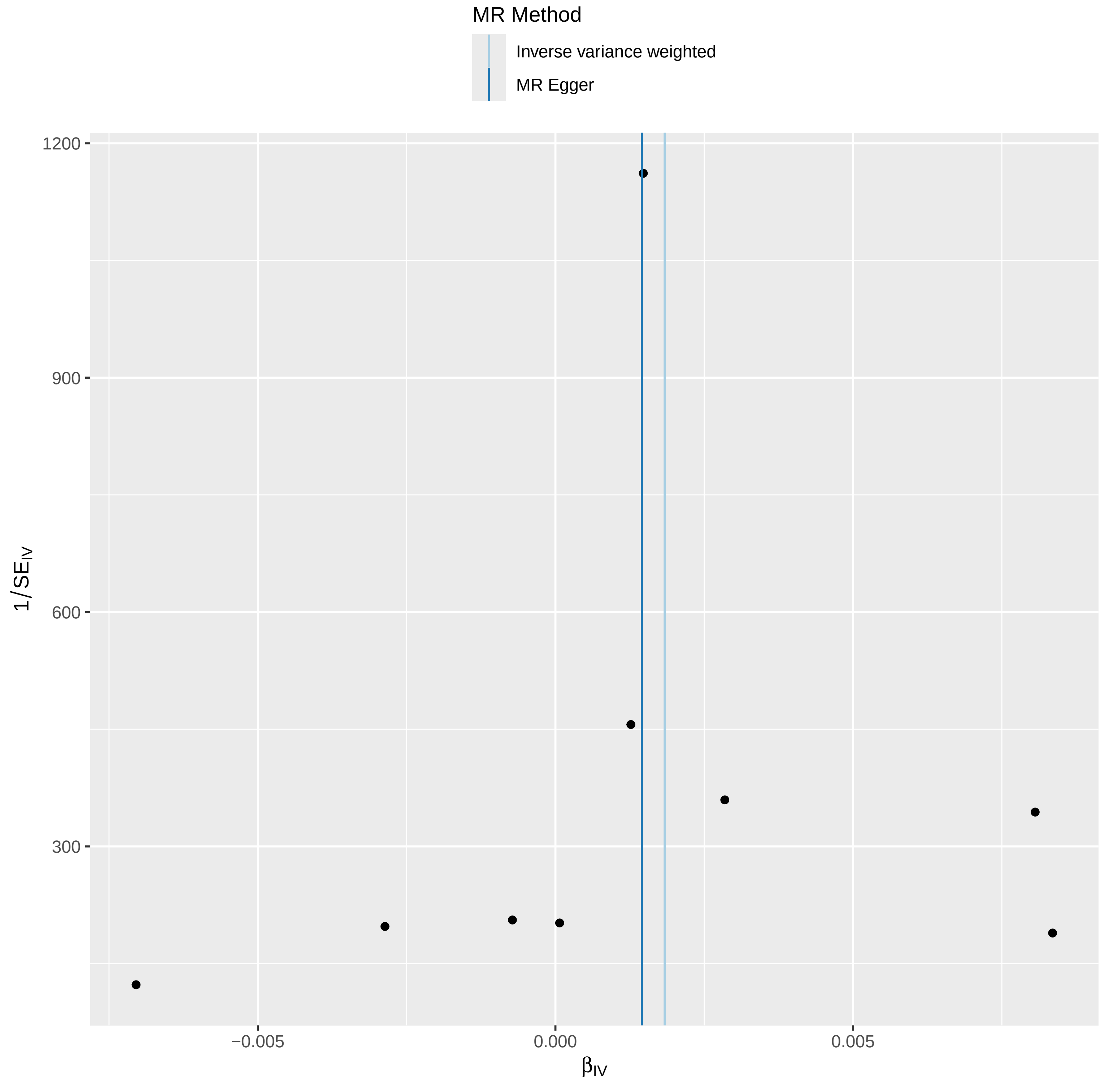

MR Method
Inverse variance weighted
MR Egger
1200
900
600
300
$1/SE_{IV}$
−0.005
0.000
0.005
β_{IV}

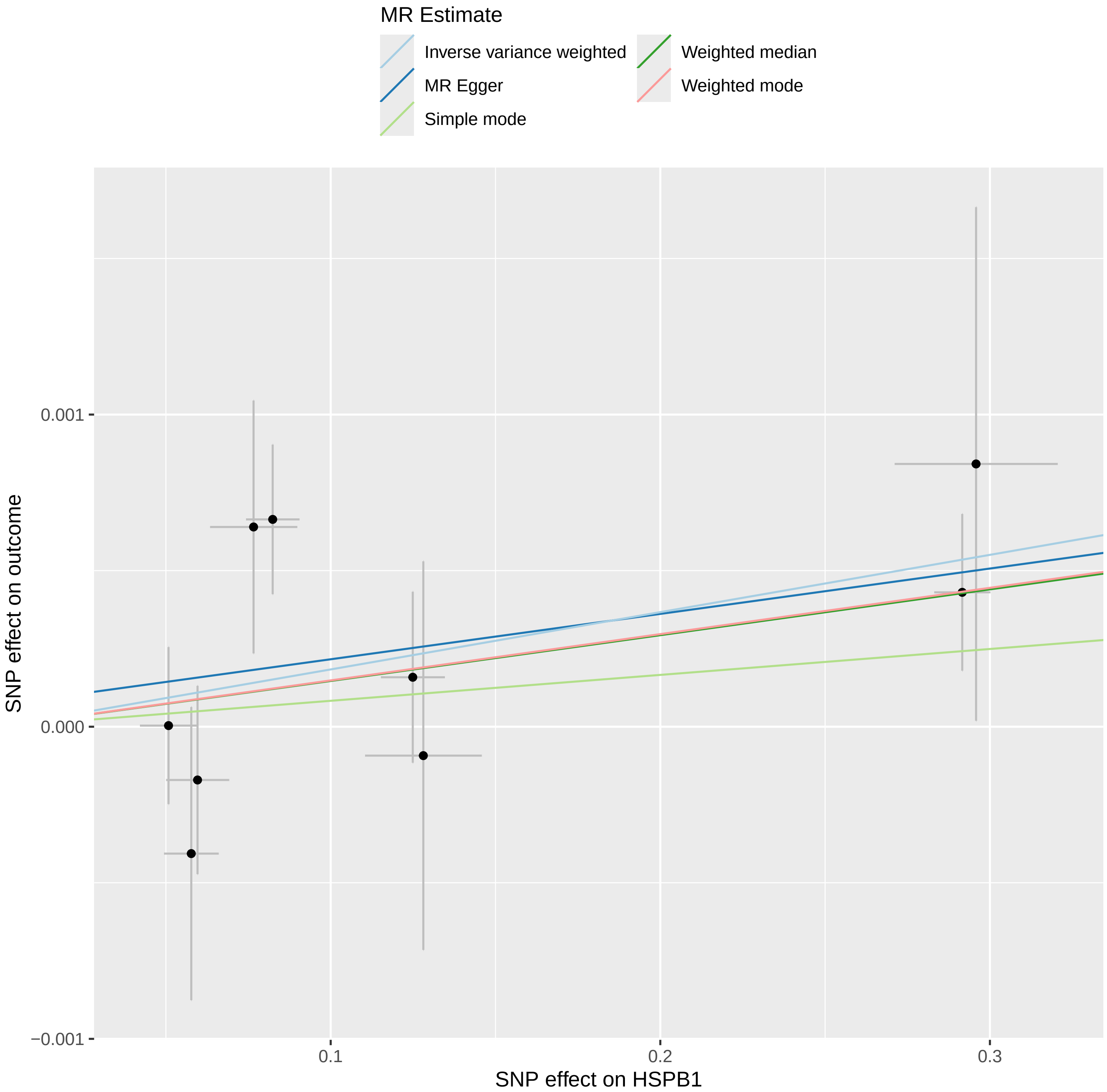
MR Estimate
Inverse variance weighted
MR Egger
Simple mode
Weighted median
Weighted mode
0.001
0.000
−0.001
0.1
0.2
0.3
SNP effect on outcome
SNP effect on HSPB1

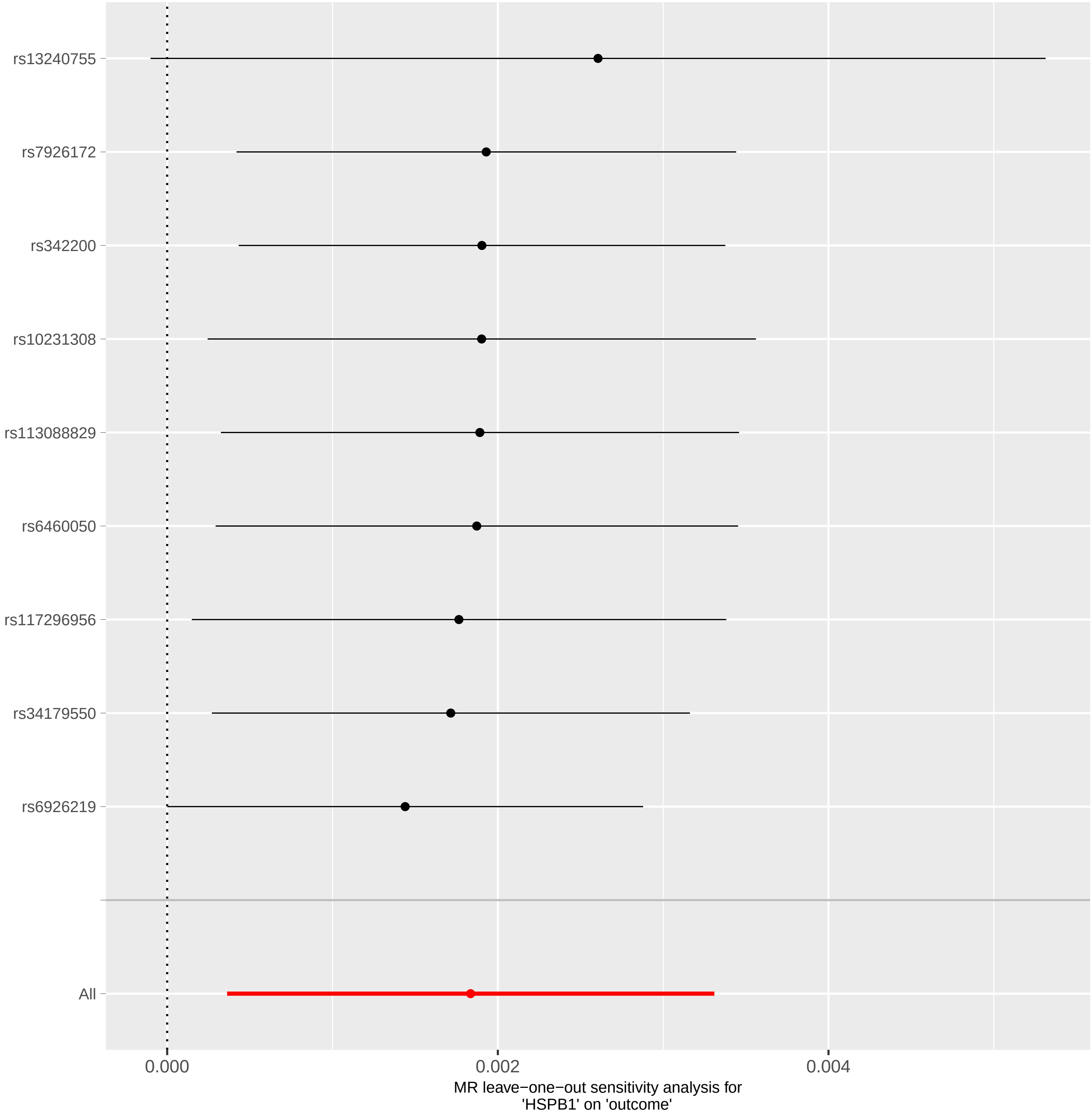

rs13240755
rs7926172
rs342200
rs10231308
rs113088829
rs6460050
rs117296956
rs34179550
rs6926219
All
0.000
0.002
0.004
MR leave−one−out sensitivity analysis for
'HSPB1' on 'outcome'

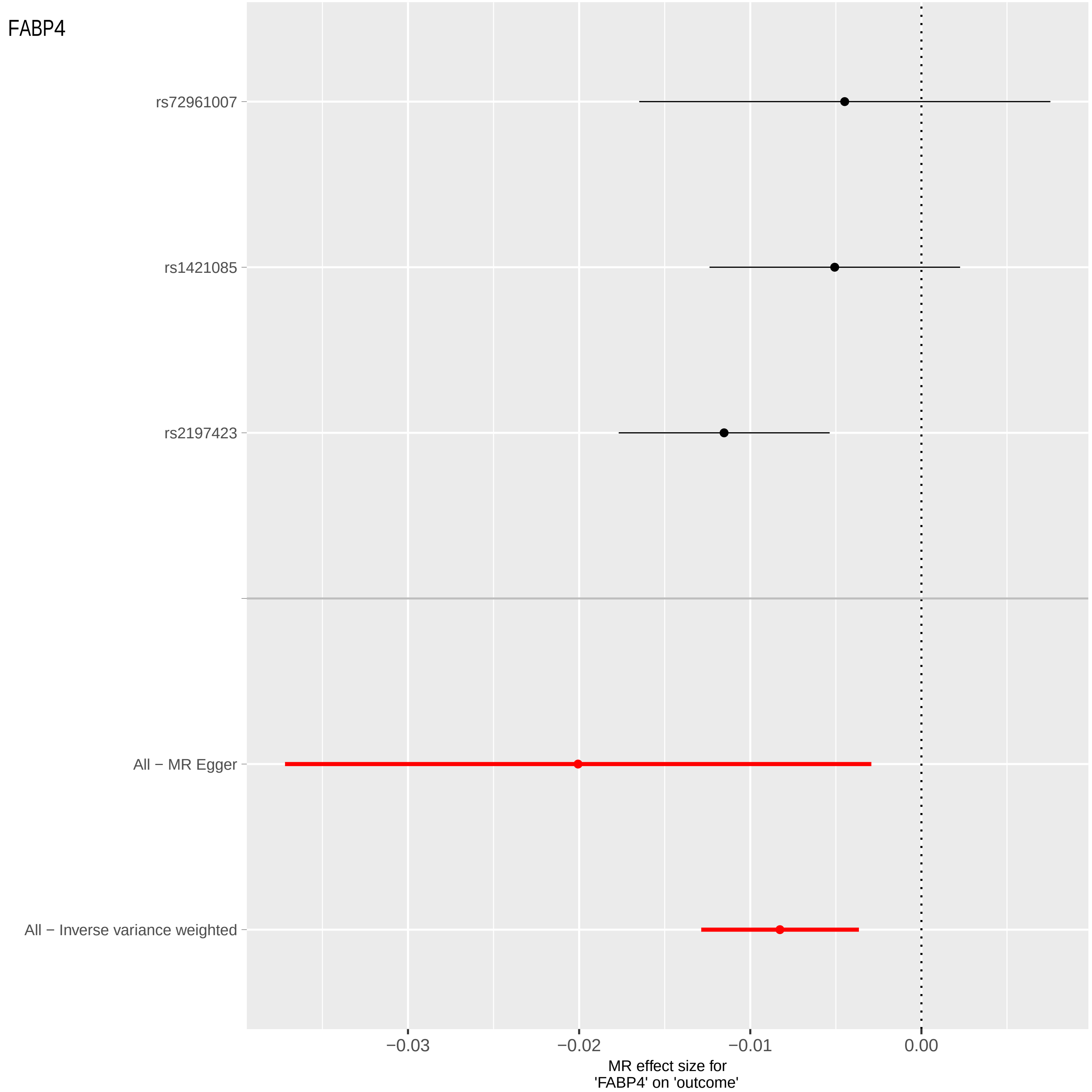
FABP4
rs72961007
rs1421085
rs2197423
All − MR Egger
All − Inverse variance weighted
−0.03
−0.02
−0.01
0.00
MR effect size for
'FABP4' on 'outcome'

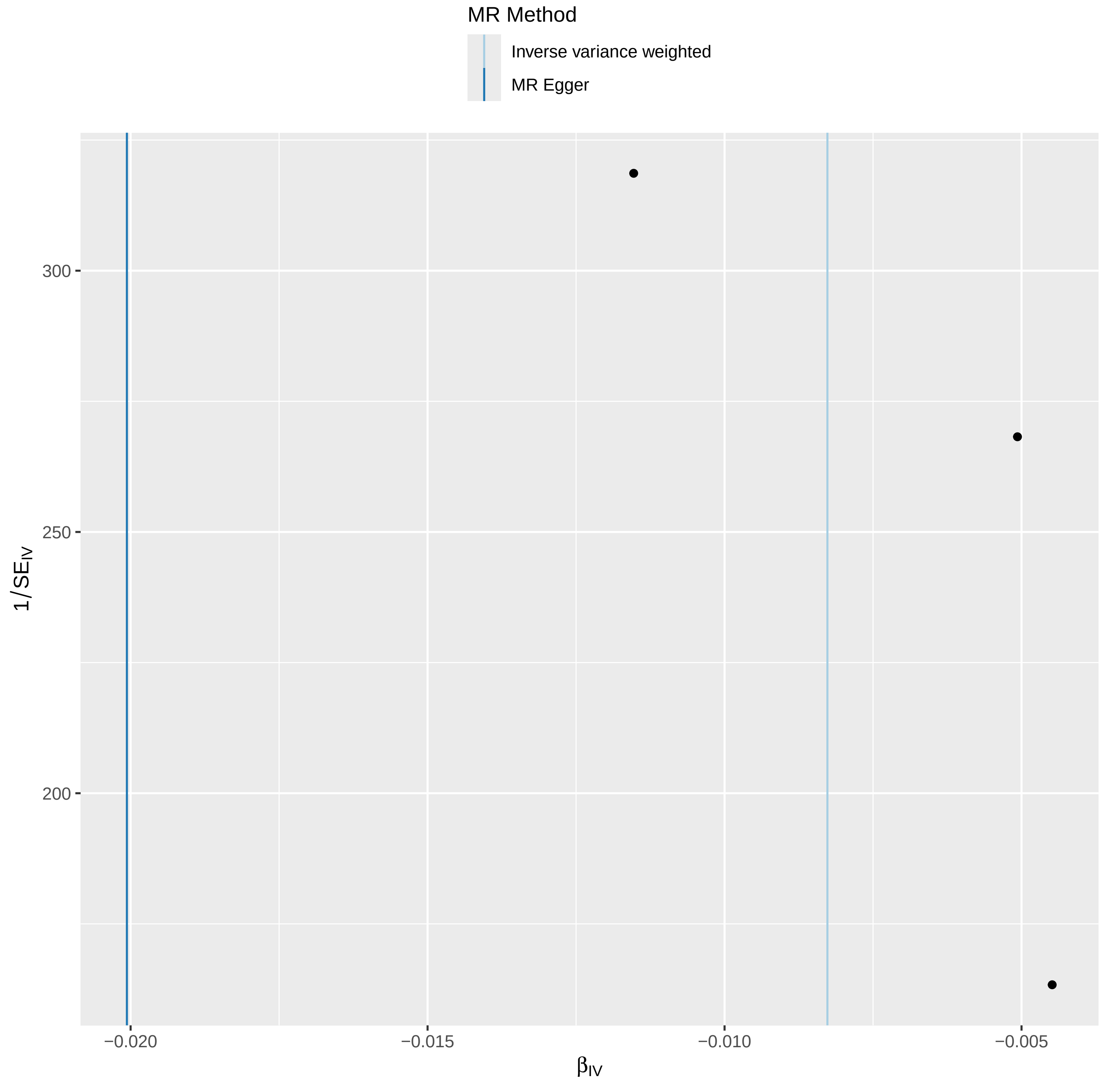
MR Method
Inverse variance weighted
MR Egger
300
250
200
1/SE_IV
−0.020
−0.015
−0.010
−0.005
β_IV

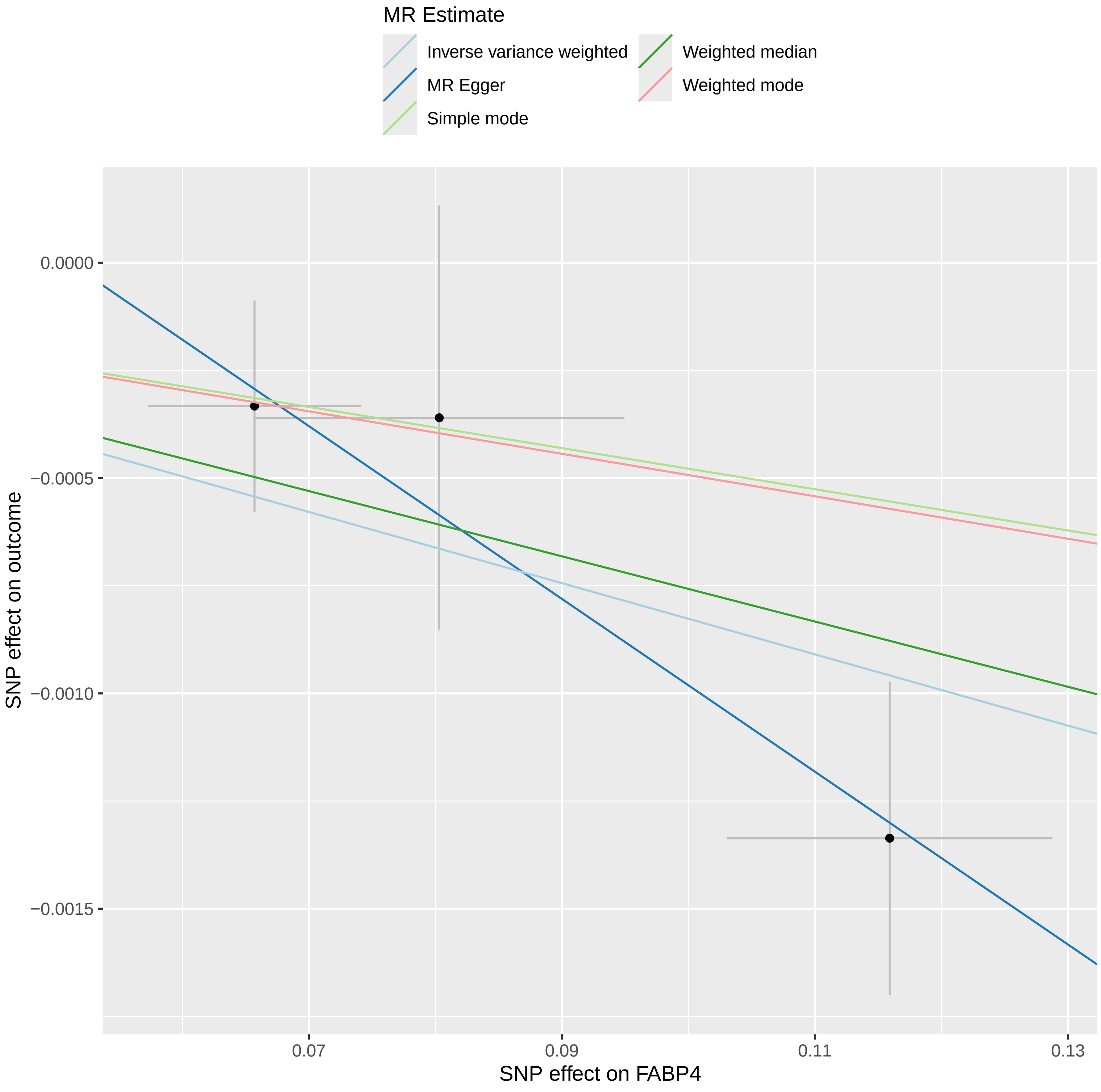
MR Estimate
Inverse variance weighted
MR Egger
Simple mode
Weighted median
Weighted mode
0.0000
−0.0005
−0.0010
−0.0015
0.07
0.09
0.11
0.13
SNP effect on outcome
SNP effect on FABP4

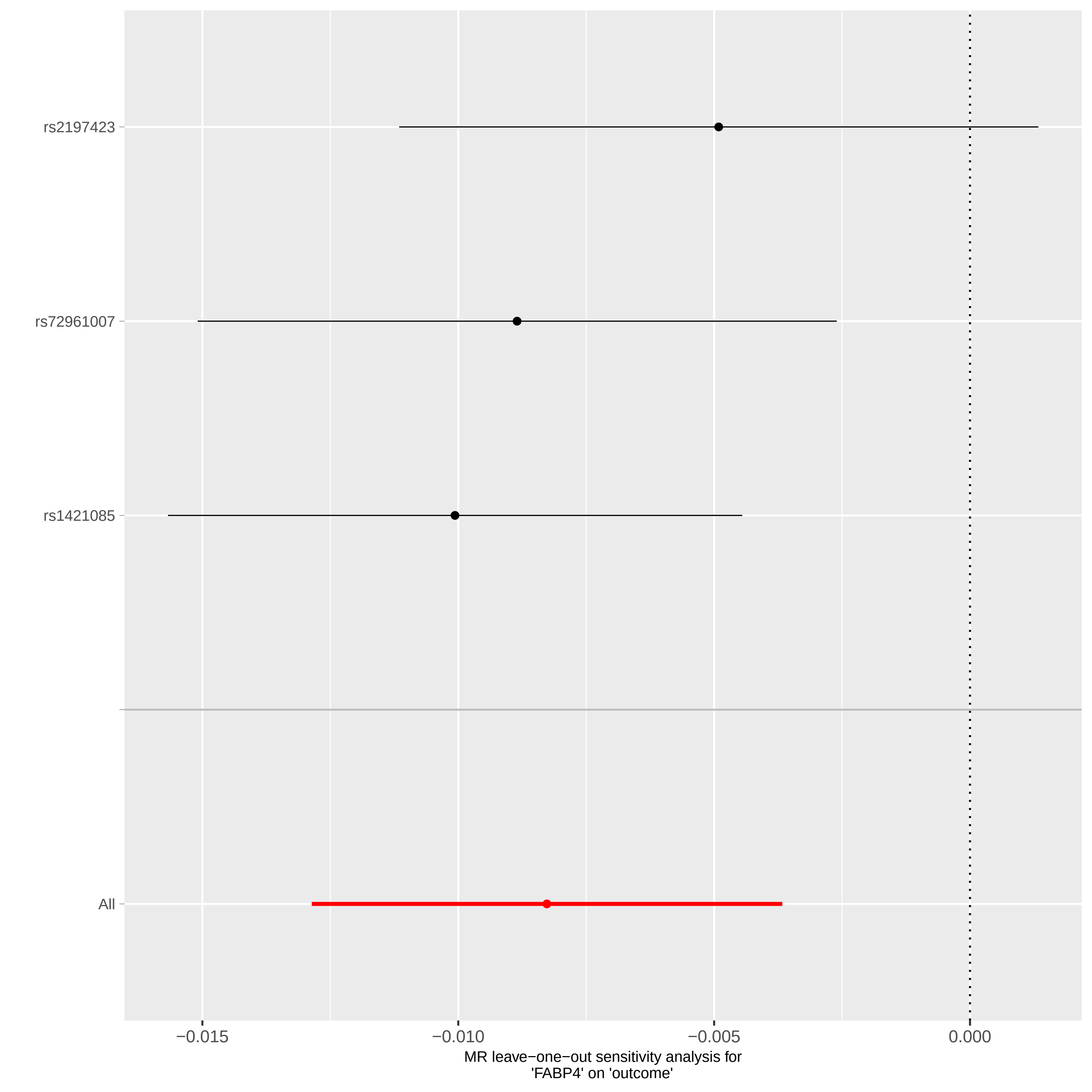
rs2197423
rs72961007
rs1421085
All
−0.015
−0.010
−0.005
0.000
MR leave−one−out sensitivity analysis for
'FABP4' on 'outcome'

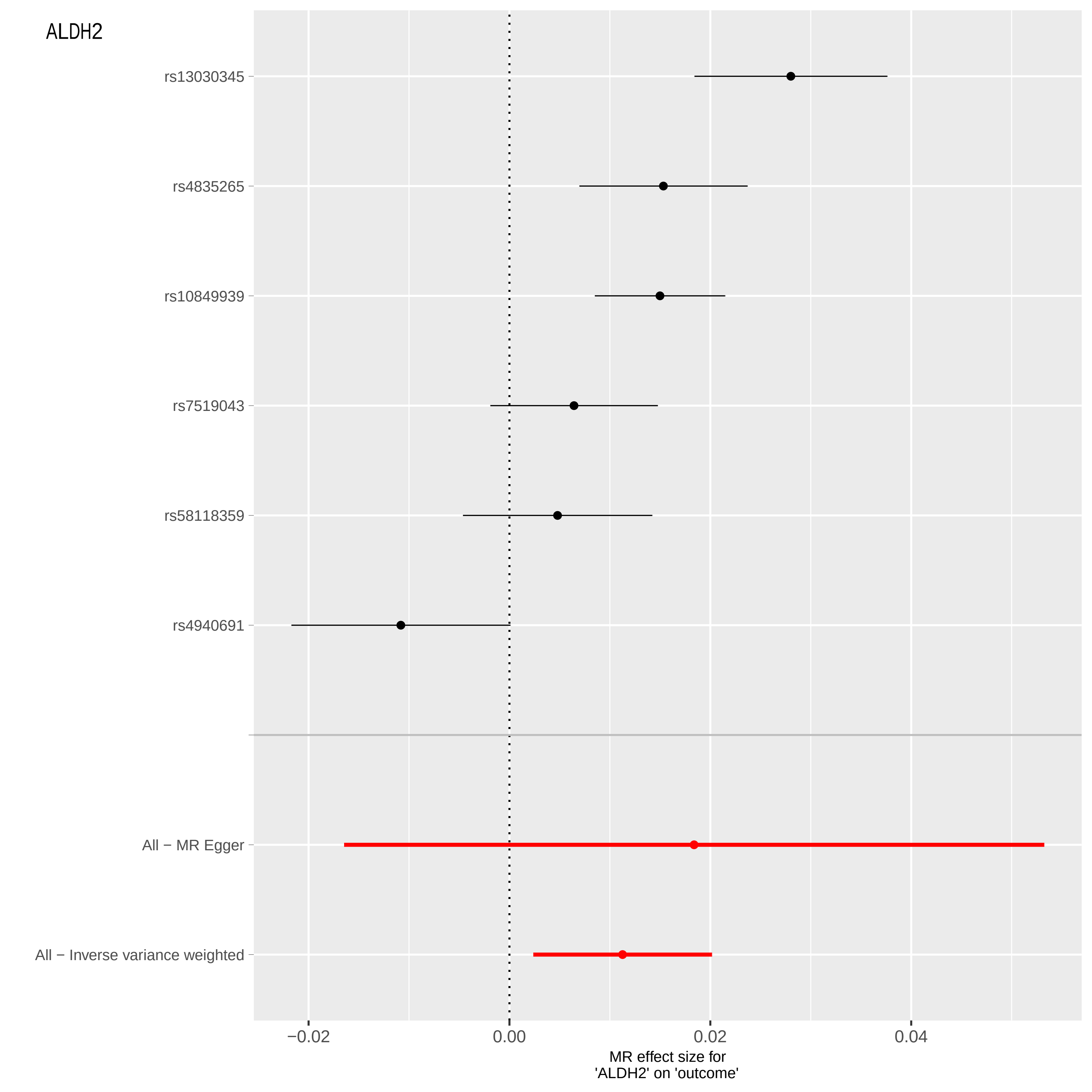

ALDH2
rs13030345
rs4835265
rs10849939
rs7519043
rs58118359
rs4940691
All − MR Egger
All − Inverse variance weighted
−0.02
0.00
0.02
0.04
MR effect size for
'ALDH2' on 'outcome'

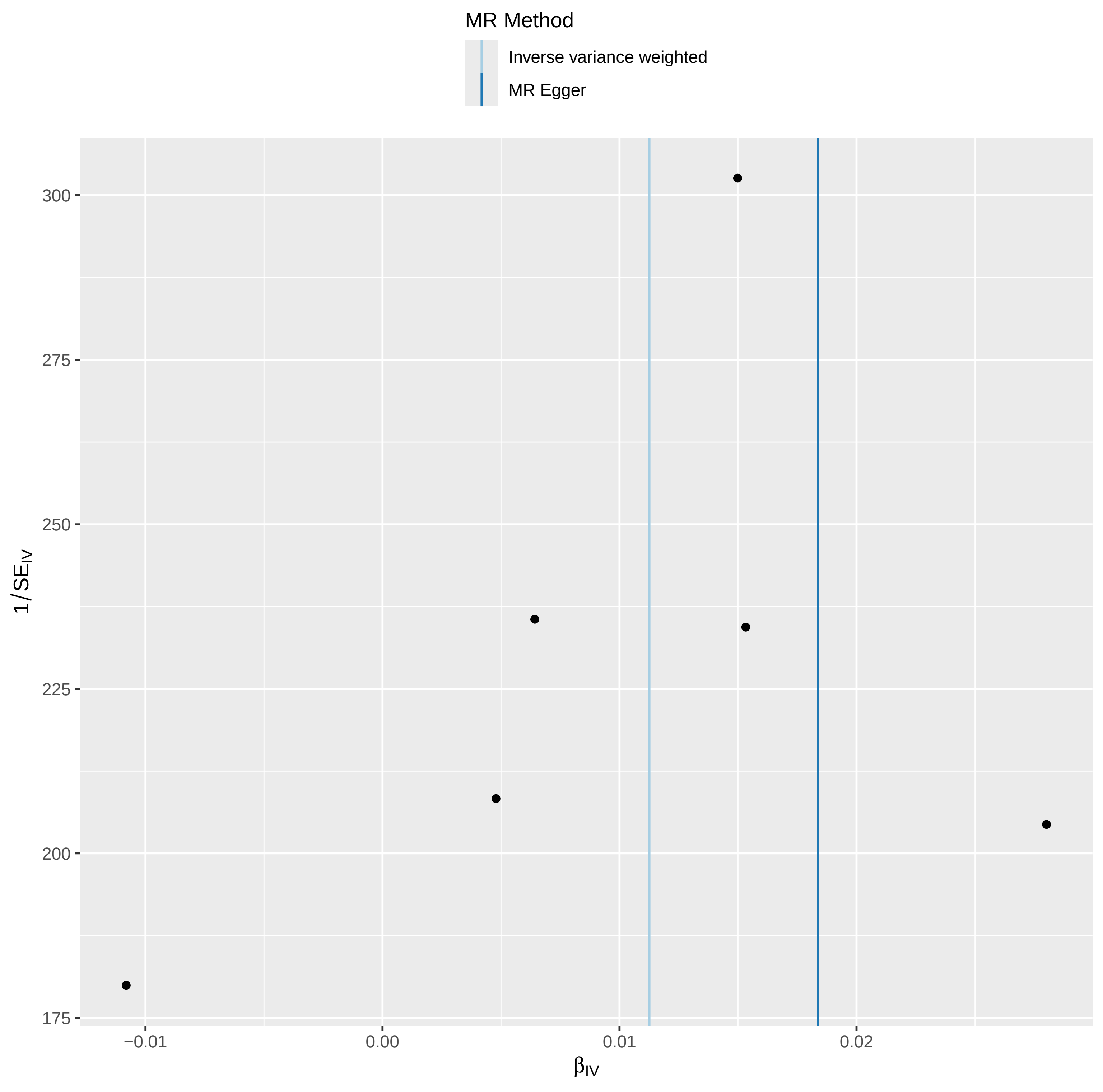

MR Method
Inverse variance weighted
MR Egger
300
275
250
225
200
175
$1/SE_{IV}$
−0.01
0.00
0.01
0.02
β_{IV}

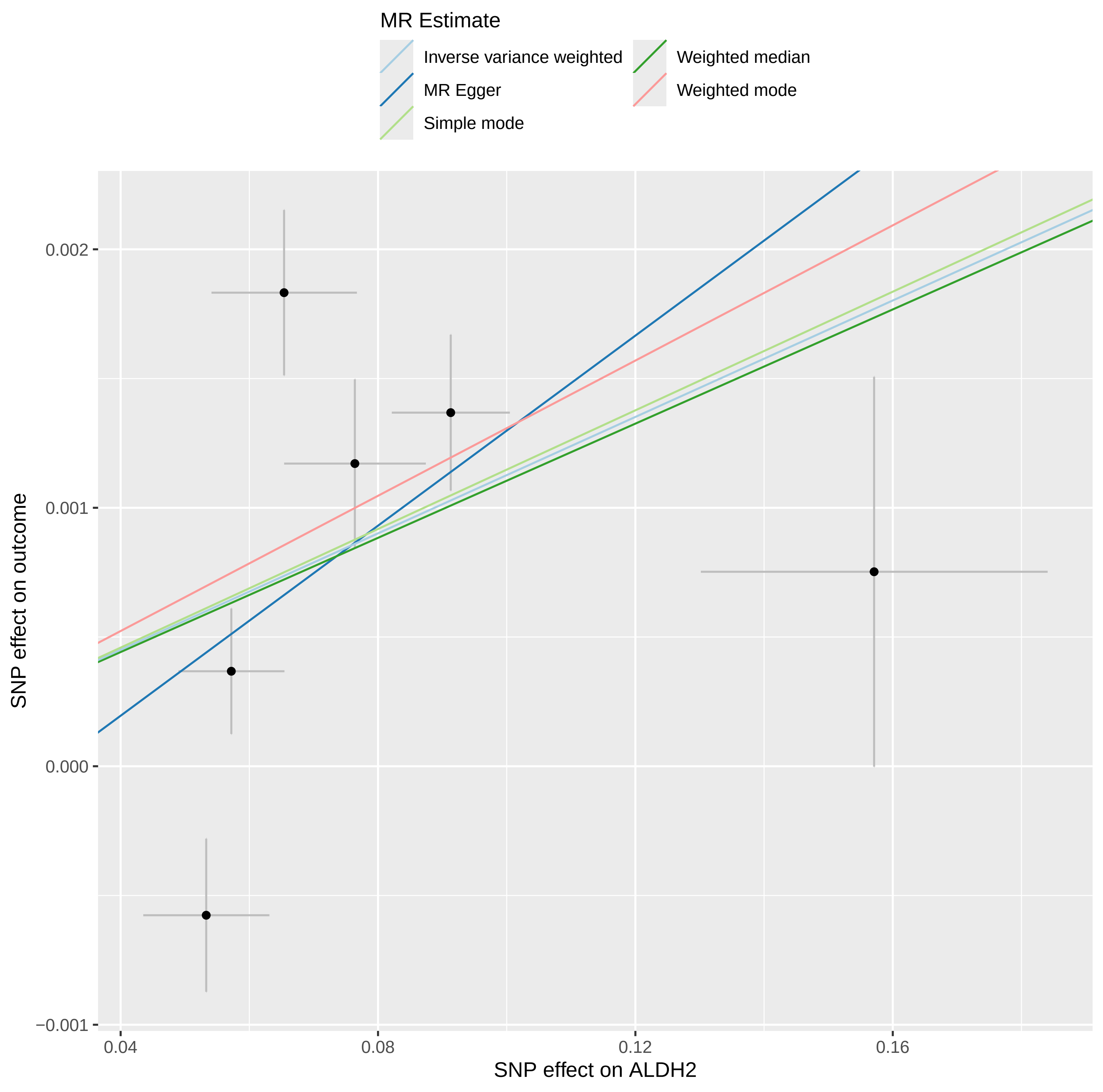

MR Estimate
Inverse variance weighted
MR Egger
Simple mode
Weighted median
Weighted mode
SNP effect on outcome
0.002
0.001
0.000
−0.001
0.04
0.08
0.12
0.16
SNP effect on ALDH2

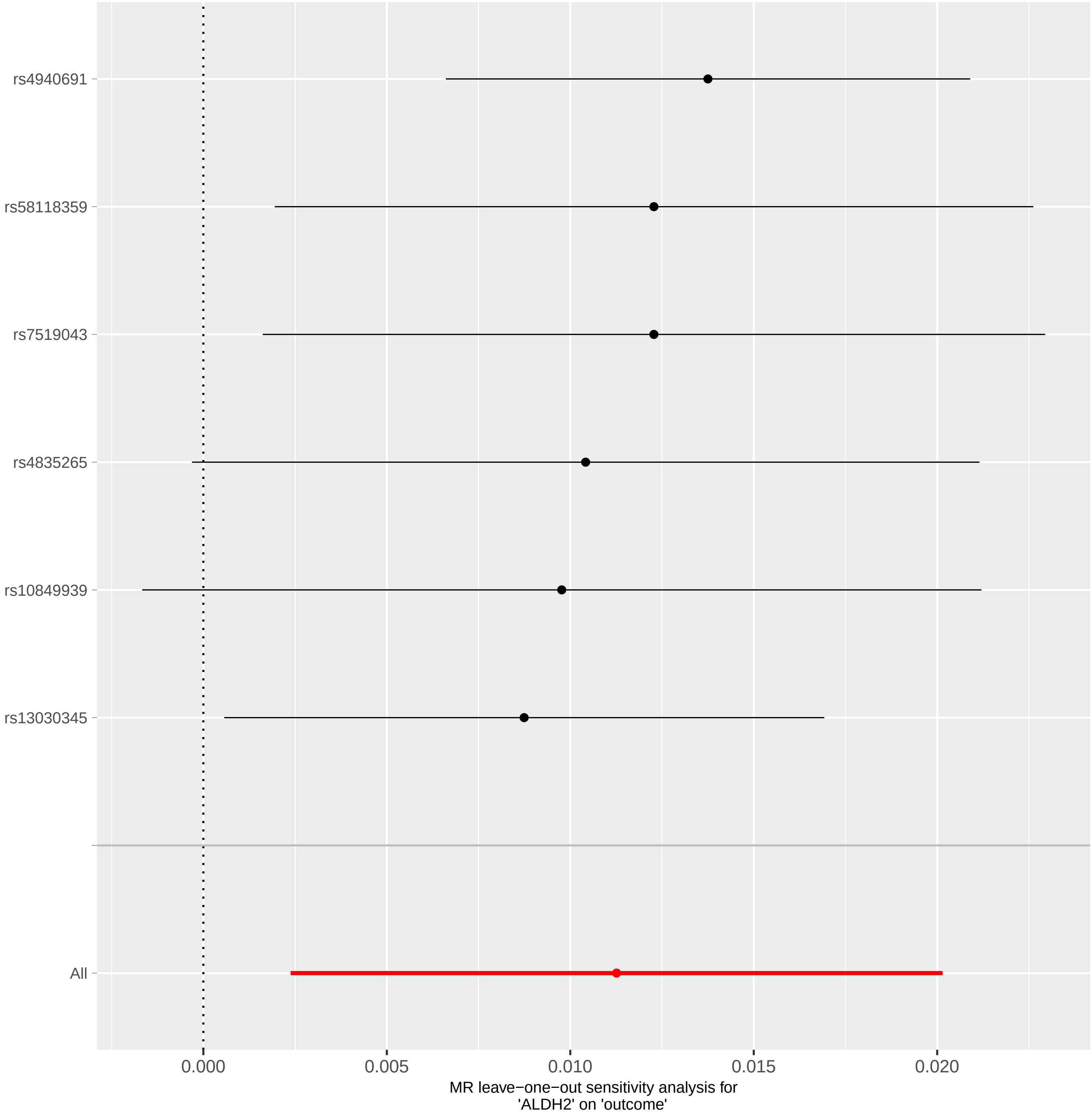

rs4940691
rs58118359
rs7519043
rs4835265
rs10849939
rs13030345
All
0.000
0.005
0.010
0.015
0.020
MR leave−one−out sensitivity analysis for
'ALDH2' on 'outcome'

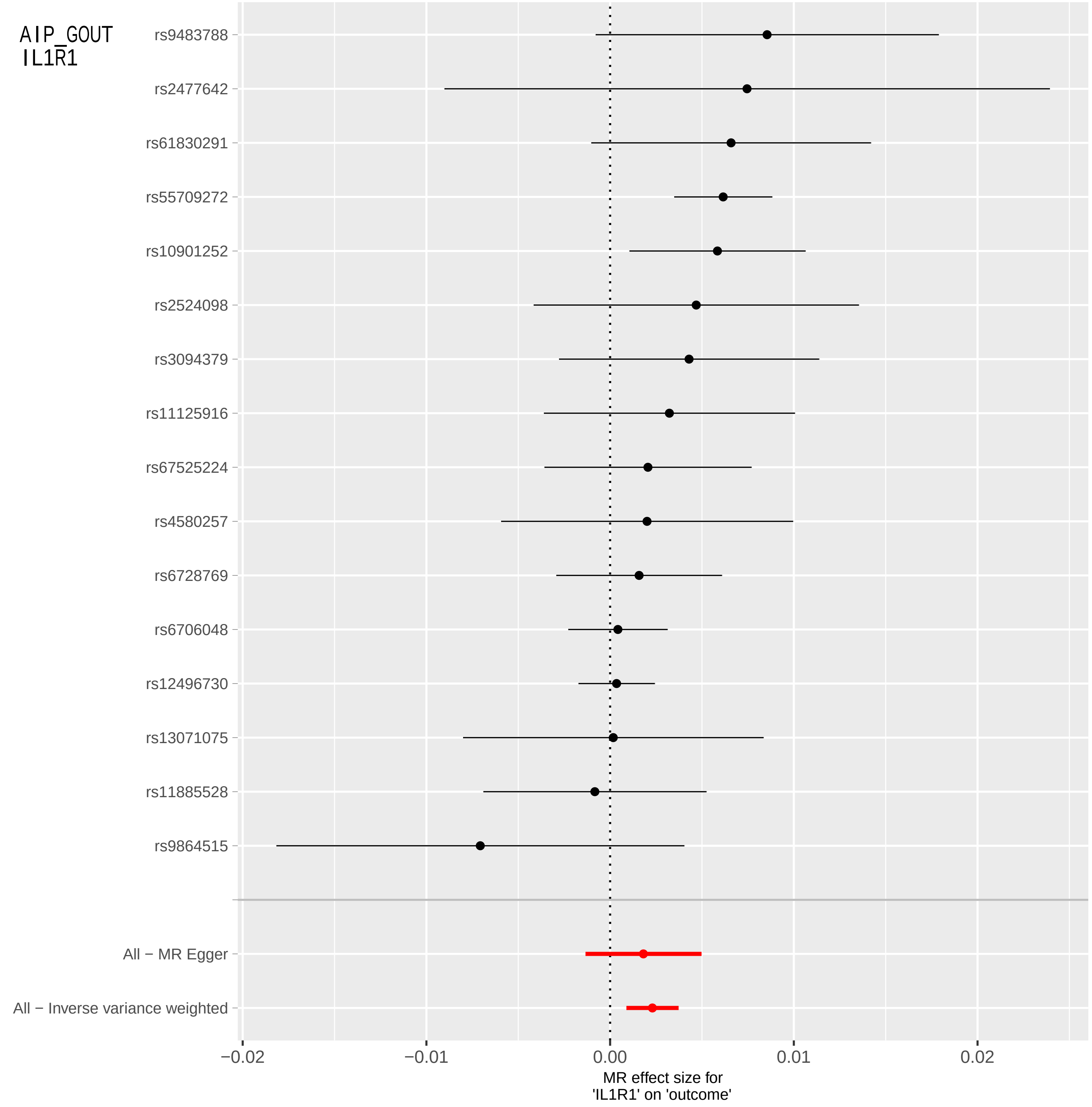

AIP_GOUT
IL1R1
rs9483788
rs2477642
rs61830291
rs55709272
rs10901252
rs2524098
rs3094379
rs11125916
rs67525224
rs4580257
rs6728769
rs6706048
rs12496730
rs13071075
rs11885528
rs9864515
All − MR Egger
All − Inverse variance weighted
−0.02
−0.01
0.00
0.01
0.02
MR effect size for
'IL1R1' on 'outcome'

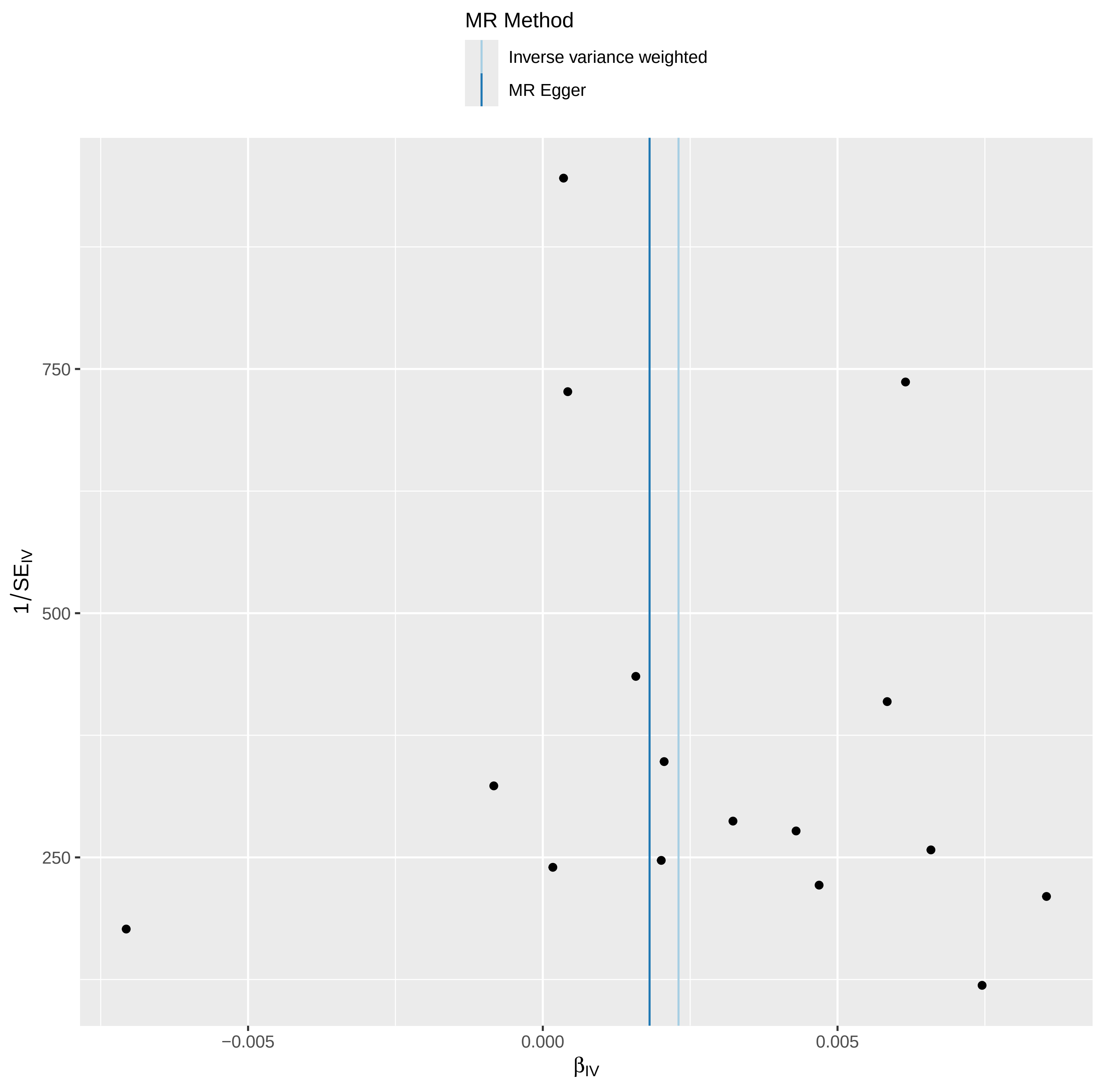
MR Method
Inverse variance weighted
MR Egger
1/SE$_{IV}$
750
500
250
−0.005
0.000
0.005
β$_{IV}$

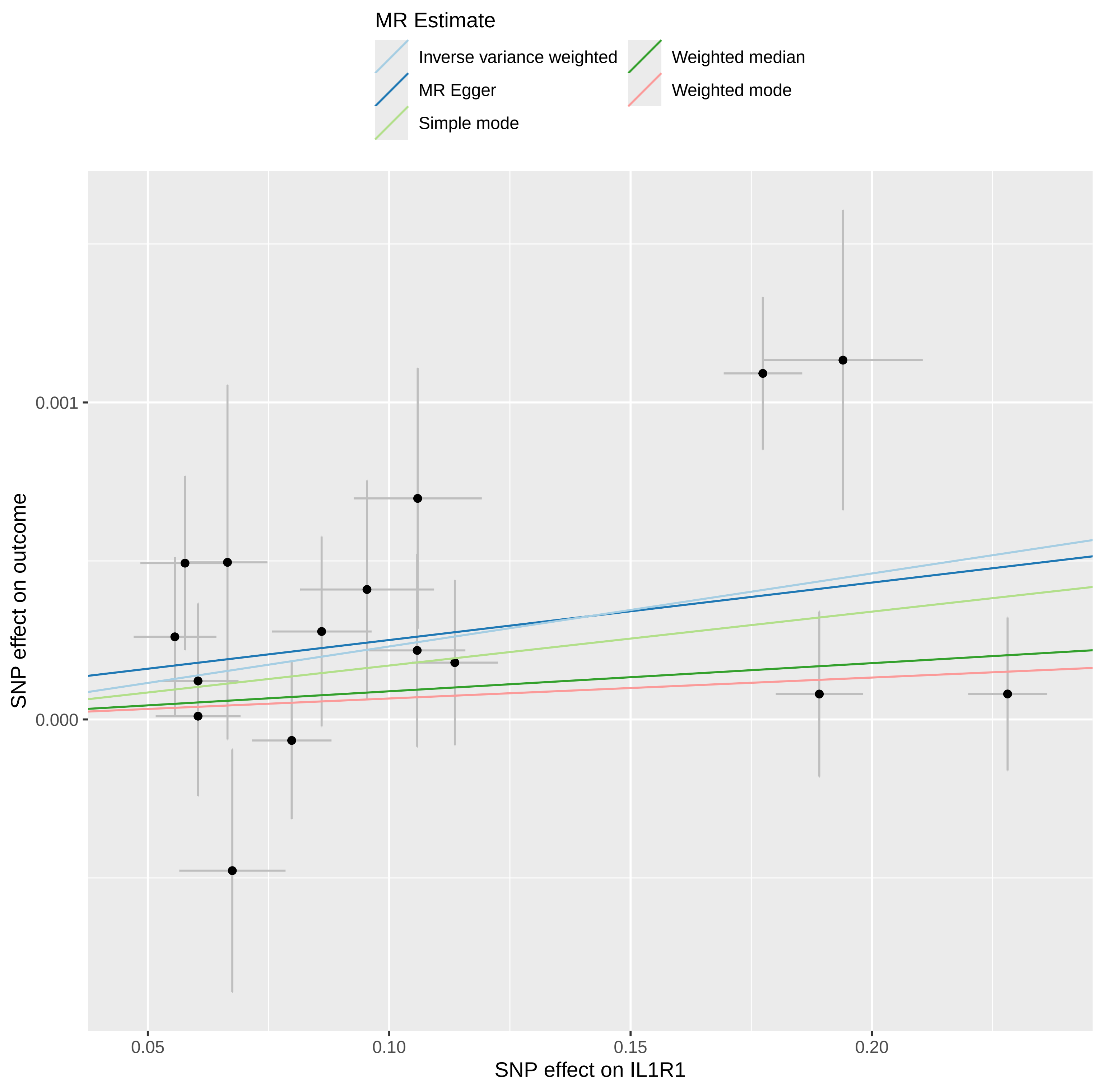
MR Estimate
Inverse variance weighted
MR Egger
Simple mode
Weighted median
Weighted mode
SNP effect on outcome
0.001
0.000
0.05
0.10
0.15
0.20
SNP effect on IL1R1

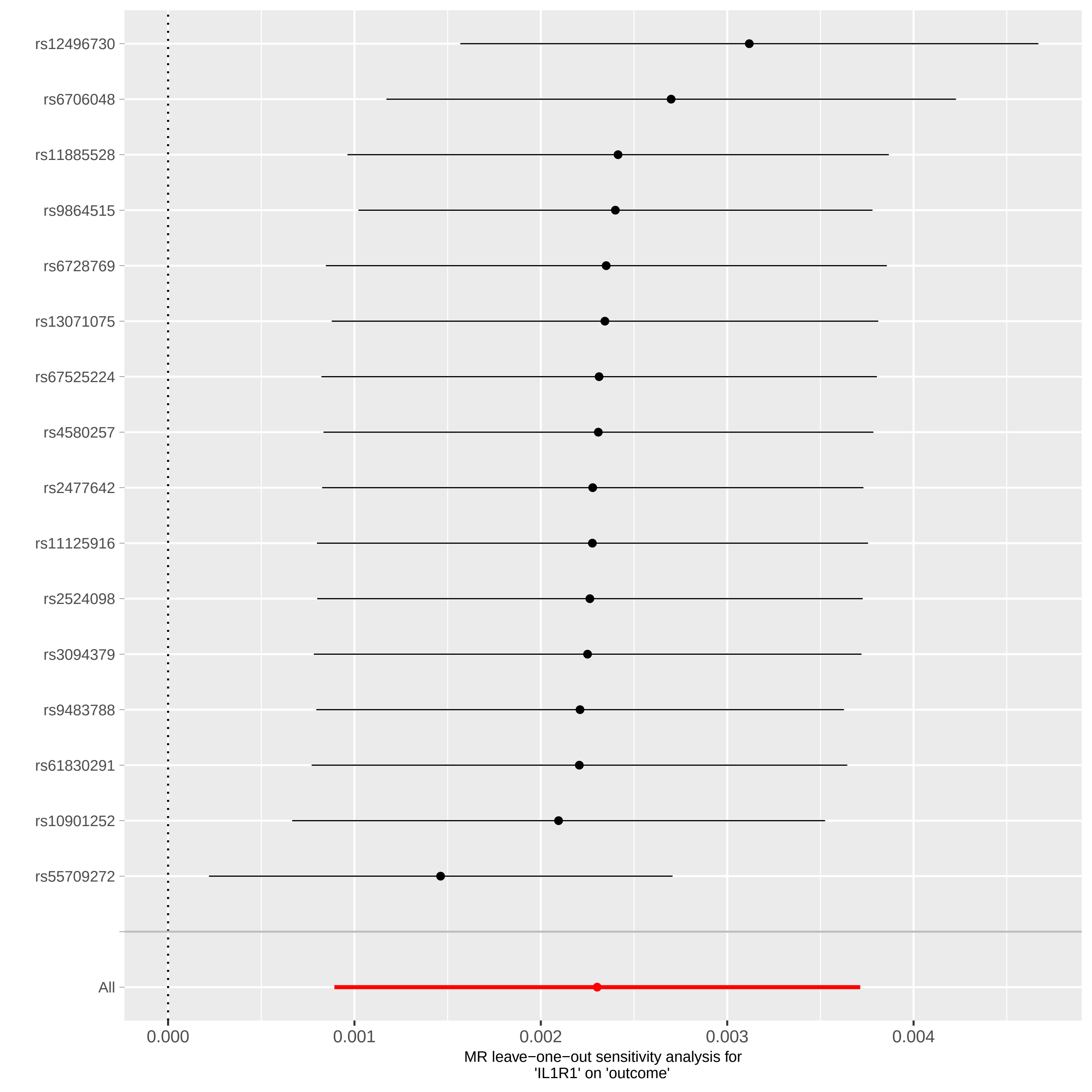
rs12496730
rs6706048
rs11885528
rs9864515
rs6728769
rs13071075
rs67525224
rs4580257
rs2477642
rs11125916
rs2524098
rs3094379
rs9483788
rs61830291
rs10901252
rs55709272
All
0.000
0.001
0.002
0.003
0.004
MR leave−one−out sensitivity analysis for
'IL1R1' on 'outcome'

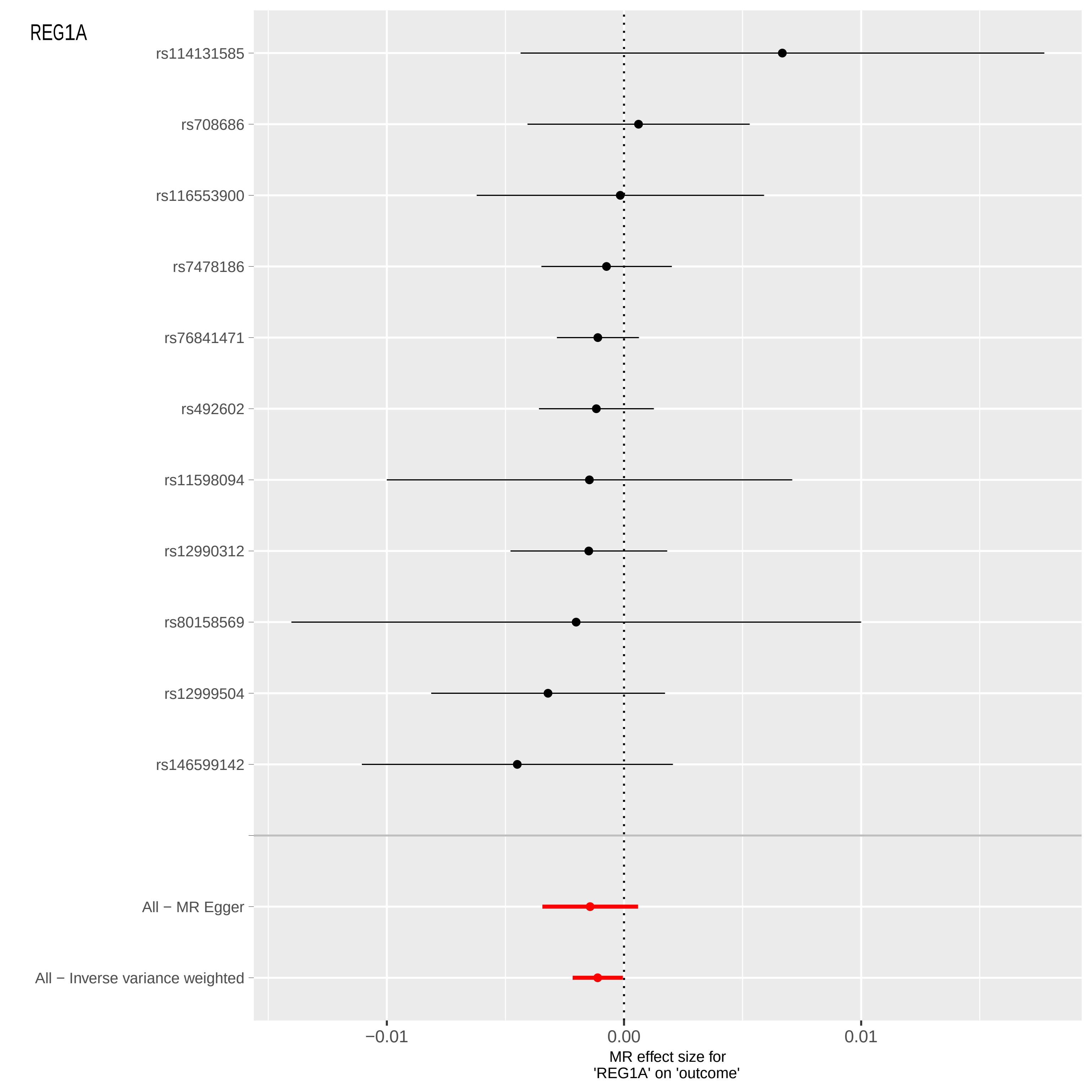

REG1A
rs114131585
rs708686
rs116553900
rs7478186
rs76841471
rs492602
rs11598094
rs12990312
rs80158569
rs12999504
rs146599142
All − MR Egger
All − Inverse variance weighted
−0.01
0.00
0.01
MR effect size for
'REG1A' on 'outcome'

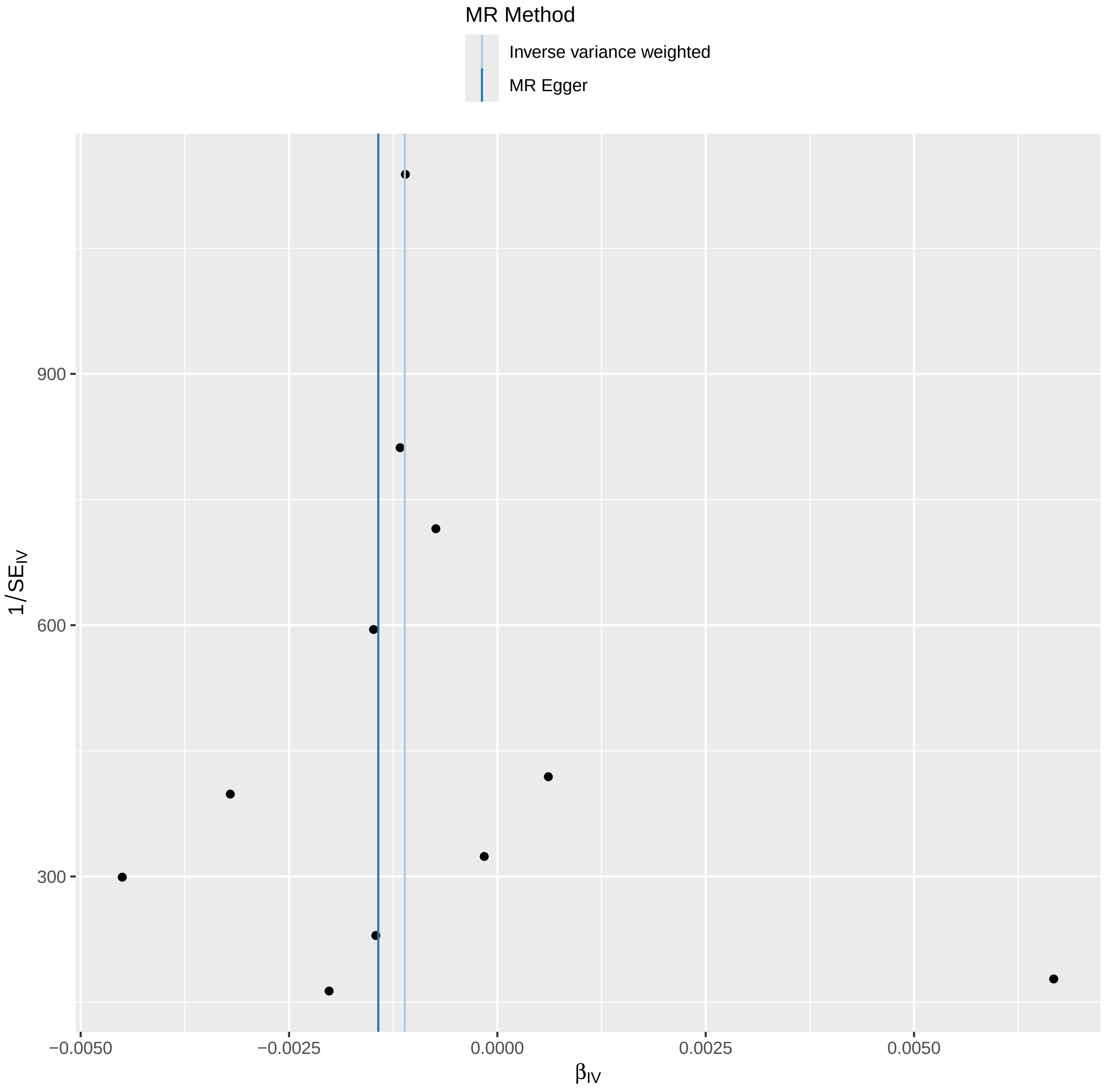

MR Method
Inverse variance weighted
MR Egger
900
600
300
1/SE_IV
−0.0050
−0.0025
0.0000
0.0025
0.0050
β_IV

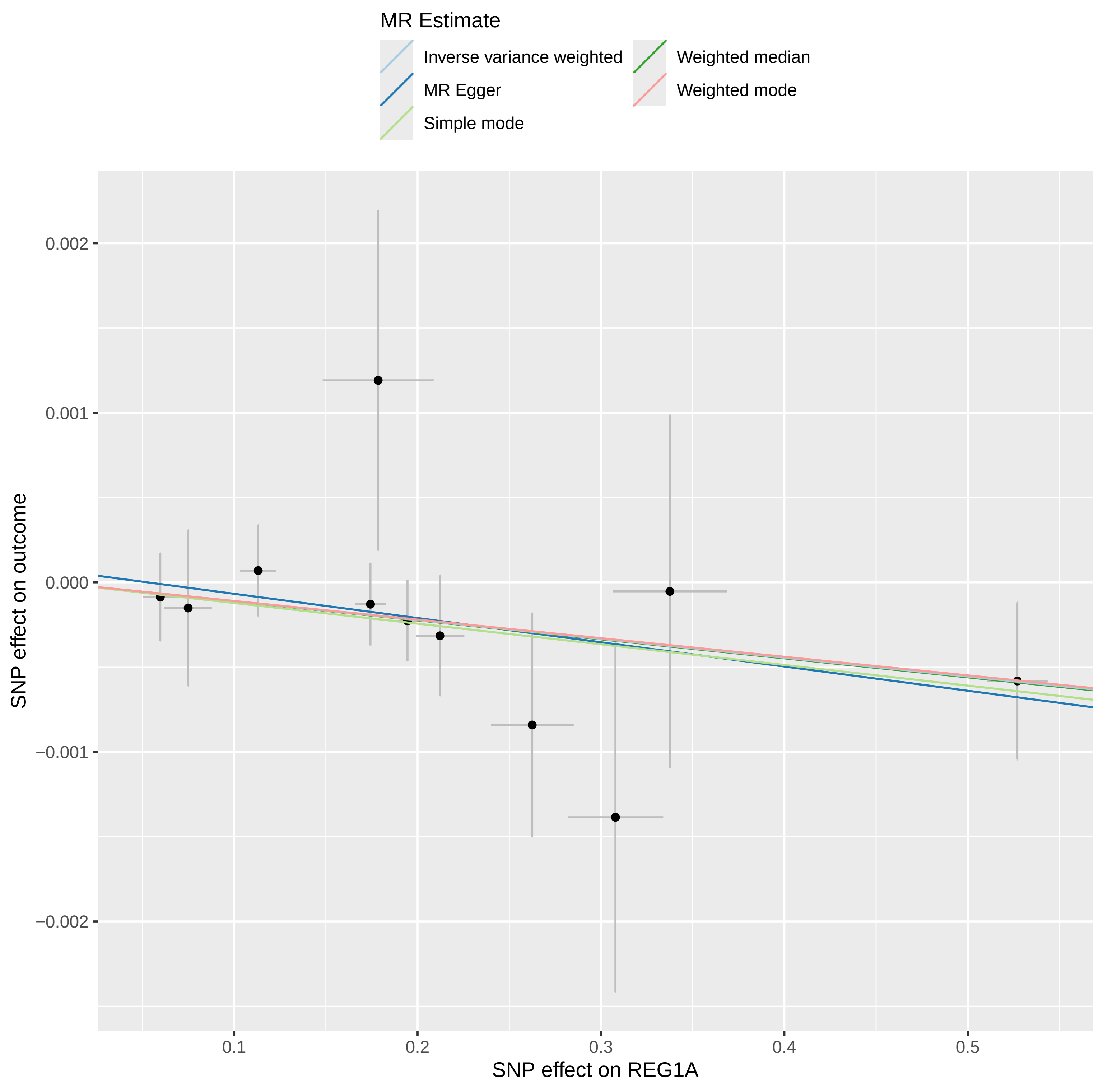

MR Estimate
Inverse variance weighted
MR Egger
Simple mode
Weighted median
Weighted mode
0.002
0.001
0.000
−0.001
−0.002
SNP effect on outcome
0.1
0.2
0.3
0.4
0.5
SNP effect on REG1A

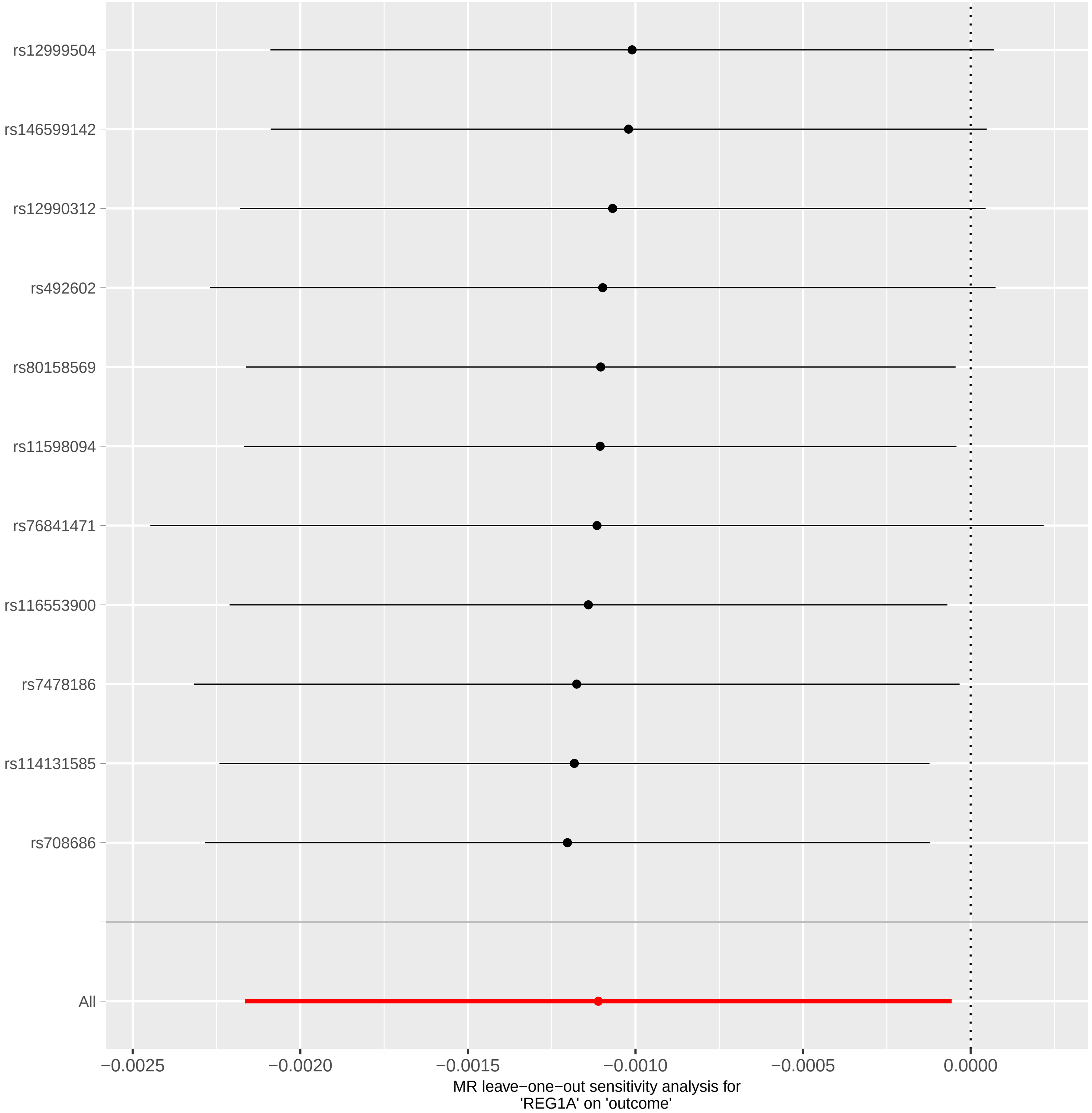
rs12999504
rs146599142
rs12990312
rs492602
rs80158569
rs11598094
rs76841471
rs116553900
rs7478186
rs114131585
rs708686
All
−0.0025
−0.0020
−0.0015
−0.0010
−0.0005
0.0000
MR leave−one−out sensitivity analysis for
'REG1A' on 'outcome'

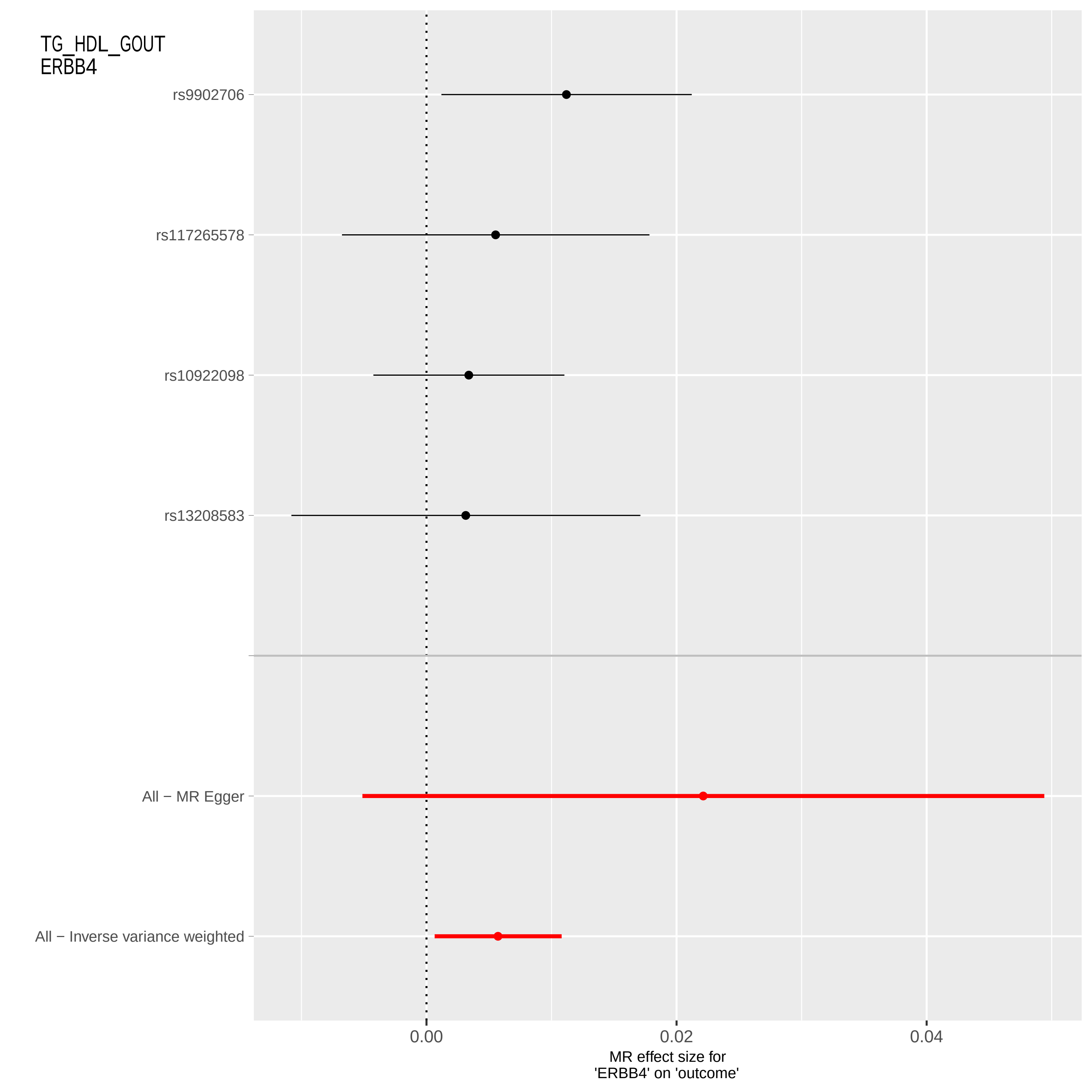

TG_HDL_GOUT
ERBB4
rs9902706
rs117265578
rs10922098
rs13208583
All − MR Egger
All − Inverse variance weighted
0.00
0.02
0.04
MR effect size for
'ERBB4' on 'outcome'

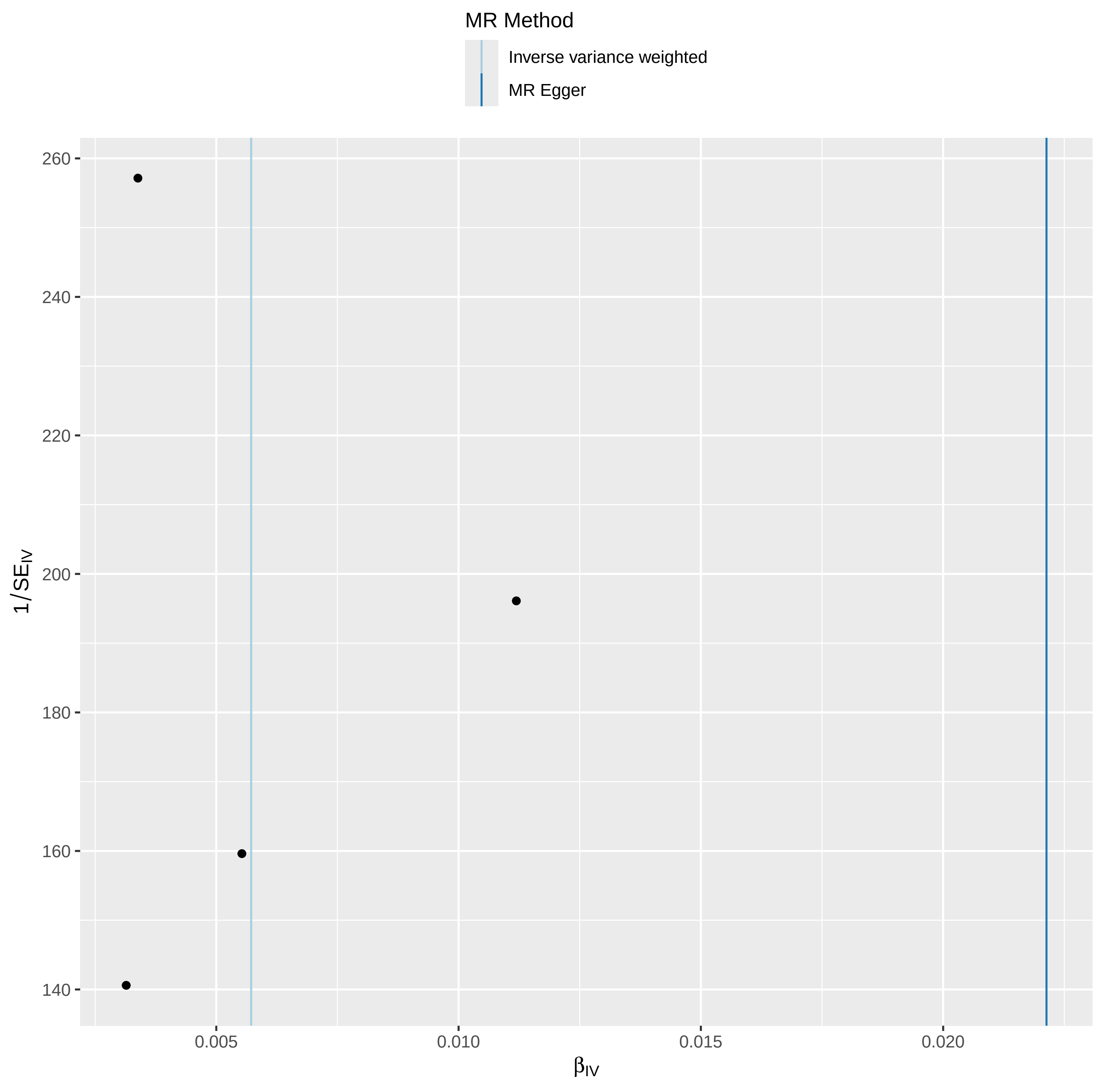

MR Method
Inverse variance weighted
MR Egger
260
240
220
200
180
160
140
1/SE_IV
0.005
0.010
0.015
0.020
β_IV

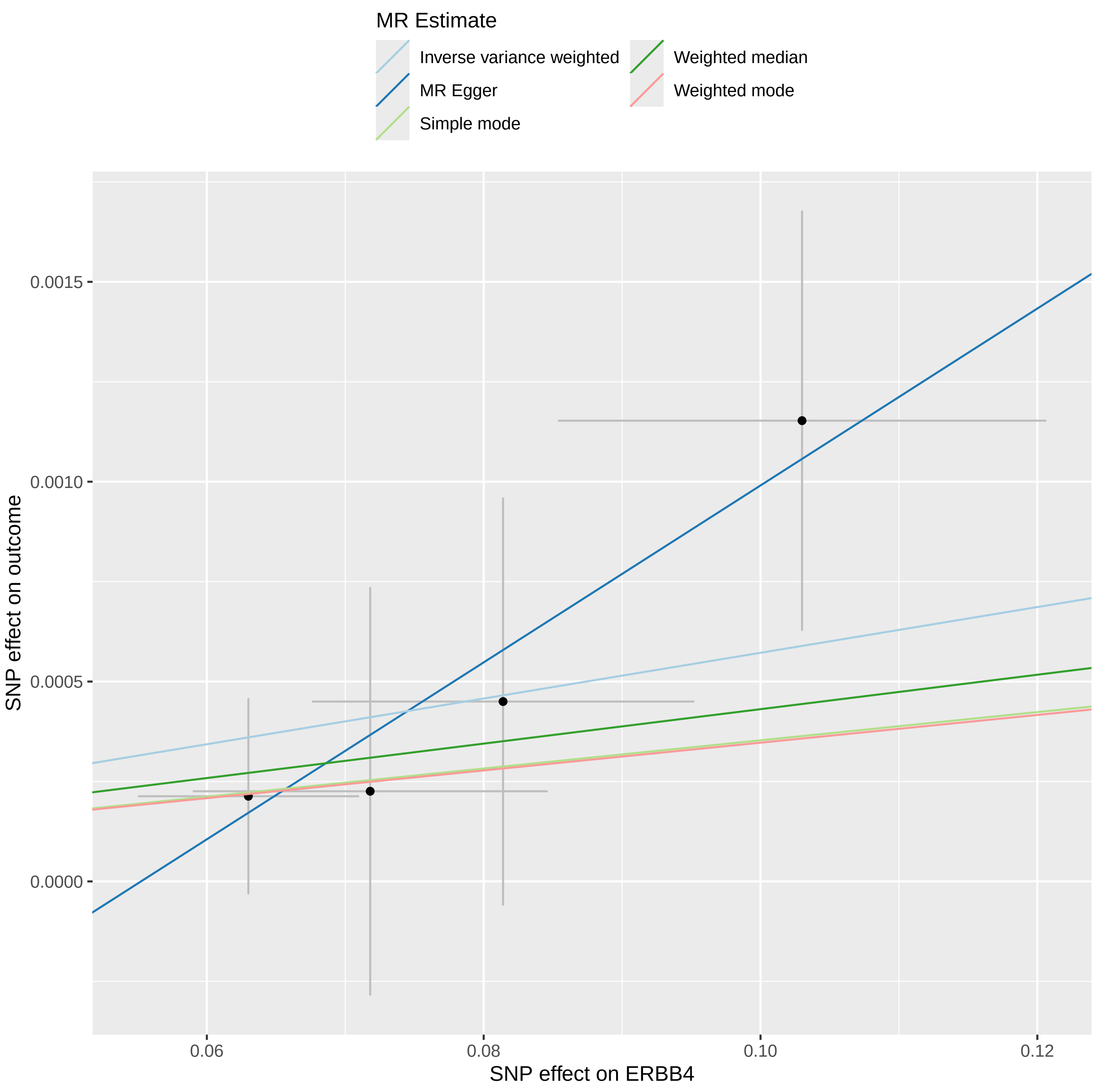
MR Estimate
Inverse variance weighted
MR Egger
Simple mode
Weighted median
Weighted mode
0.0015
0.0010
0.0005
0.0000
SNP effect on outcome
0.06
0.08
0.10
0.12
SNP effect on ERBB4

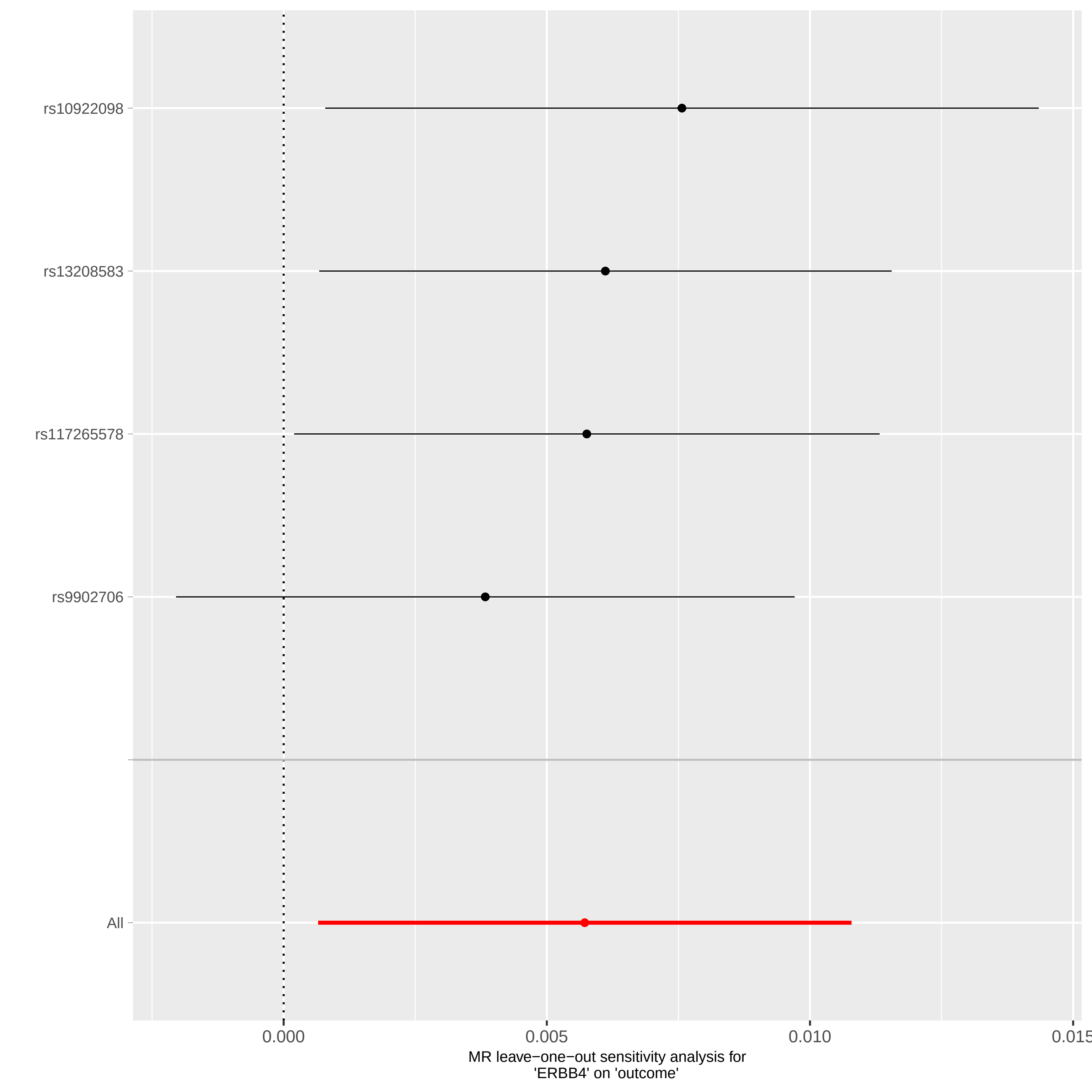
rs10922098
rs13208583
rs117265578
rs9902706
All
0.000
0.005
0.010
0.015
MR leave−one−out sensitivity analysis for
'ERBB4' on 'outcome'

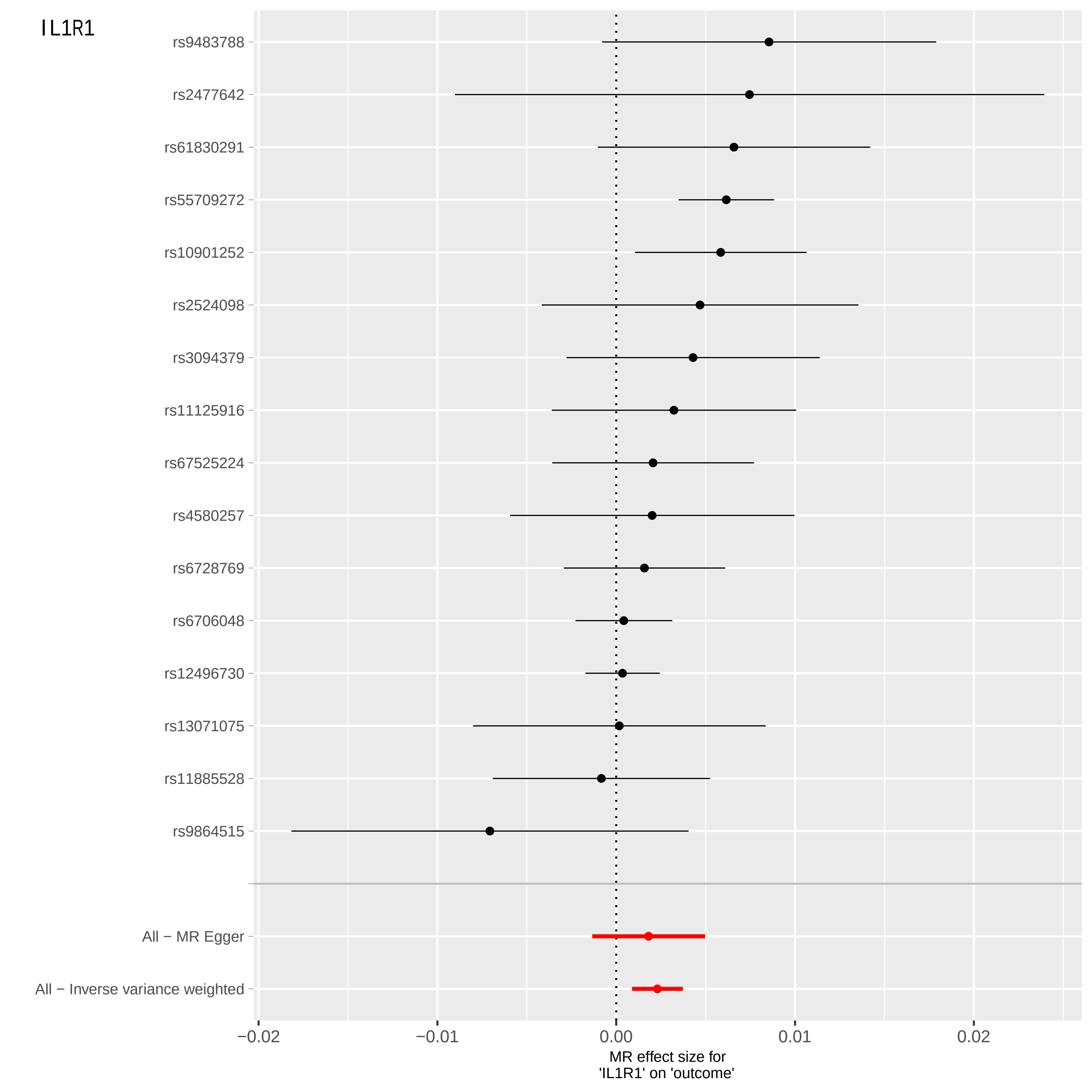
IL1R1
rs9483788
rs2477642
rs61830291
rs55709272
rs10901252
rs2524098
rs3094379
rs11125916
rs67525224
rs4580257
rs6728769
rs6706048
rs12496730
rs13071075
rs11885528
rs9864515
All − MR Egger
All − Inverse variance weighted
−0.02
−0.01
0.00
0.01
0.02
MR effect size for
'IL1R1' on 'outcome'

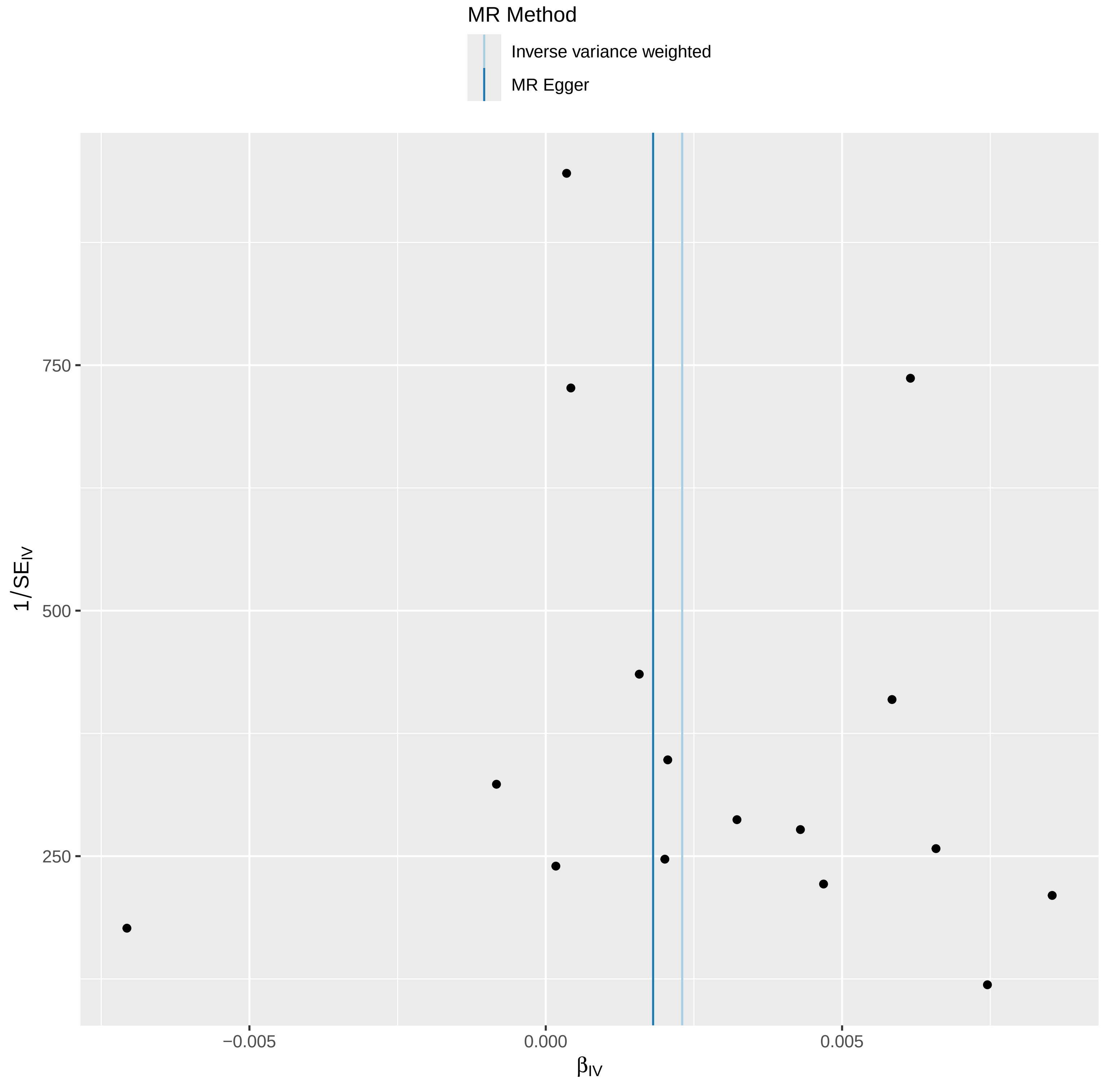

MR Method
Inverse variance weighted
MR Egger
750
500
250
$1/SE_{IV}$
−0.005
0.000
0.005
β_{IV}

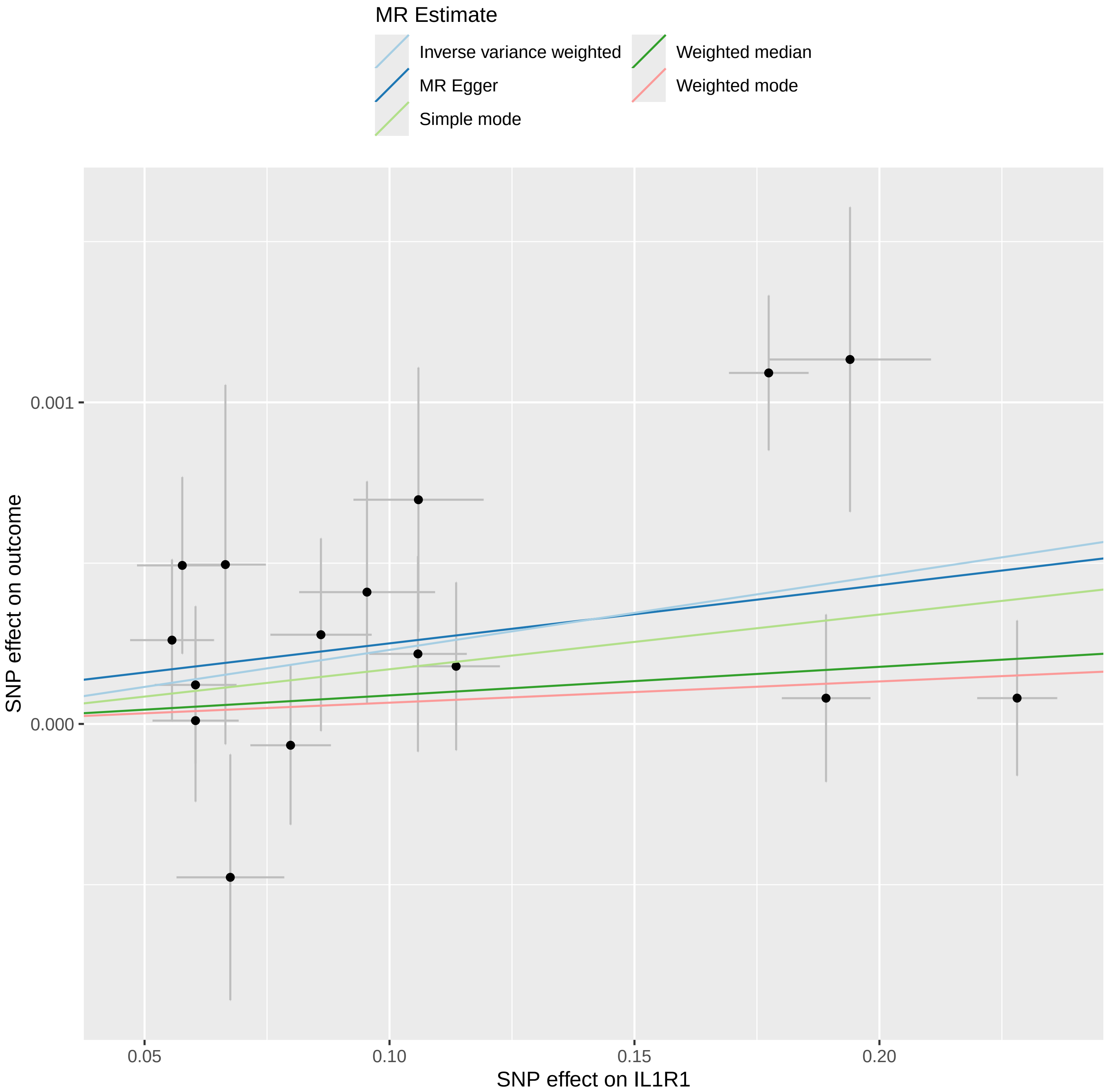
MR Estimate
Inverse variance weighted
MR Egger
Simple mode
Weighted median
Weighted mode
0.001
0.000
SNP effect on outcome
0.05
0.10
0.15
0.20
SNP effect on IL1R1

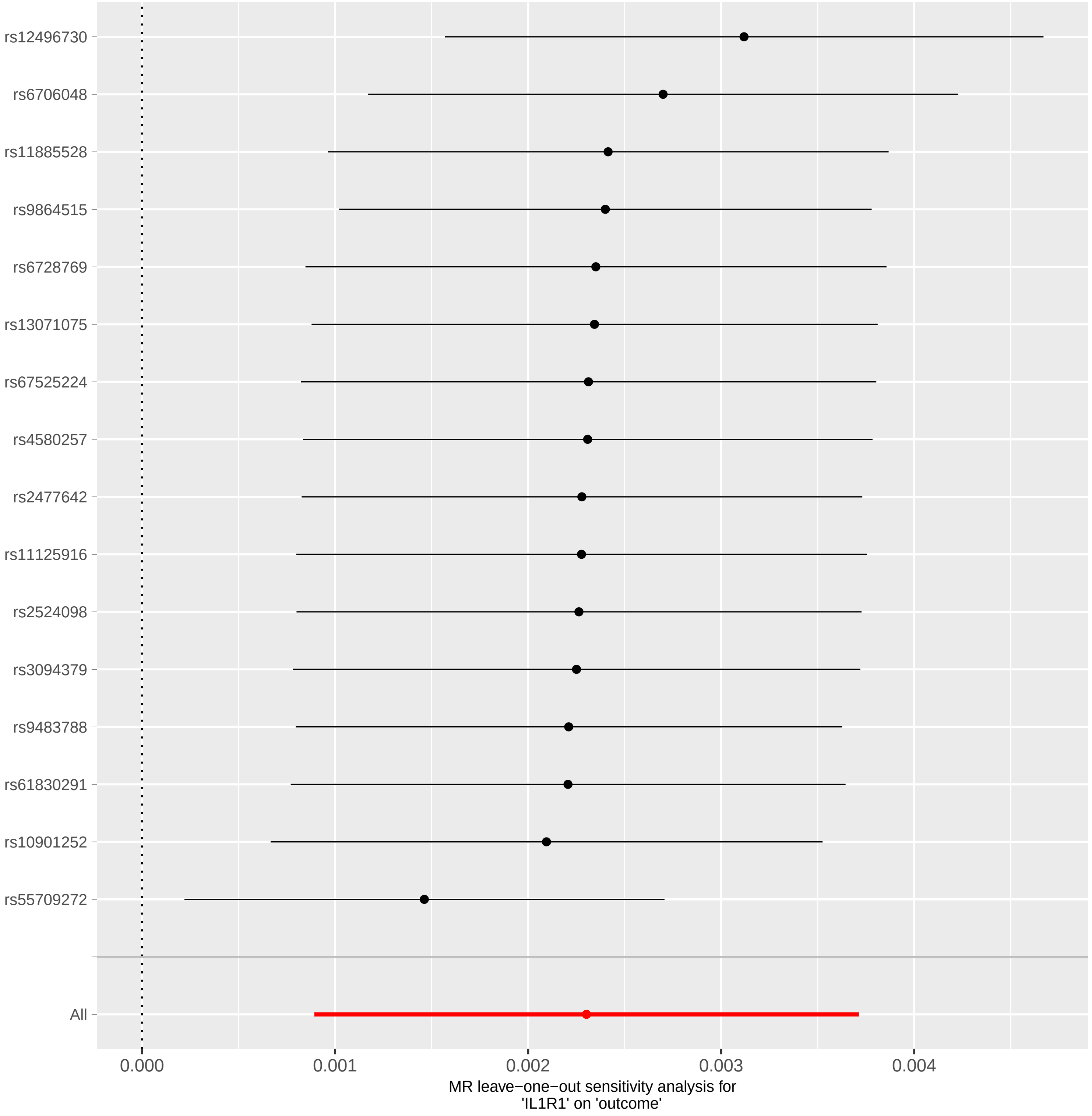

rs12496730
rs6706048
rs11885528
rs9864515
rs6728769
rs13071075
rs67525224
rs4580257
rs2477642
rs11125916
rs2524098
rs3094379
rs9483788
rs61830291
rs10901252
rs55709272
All
0.000
0.001
0.002
0.003
0.004
MR leave−one−out sensitivity analysis for
'IL1R1' on 'outcome'

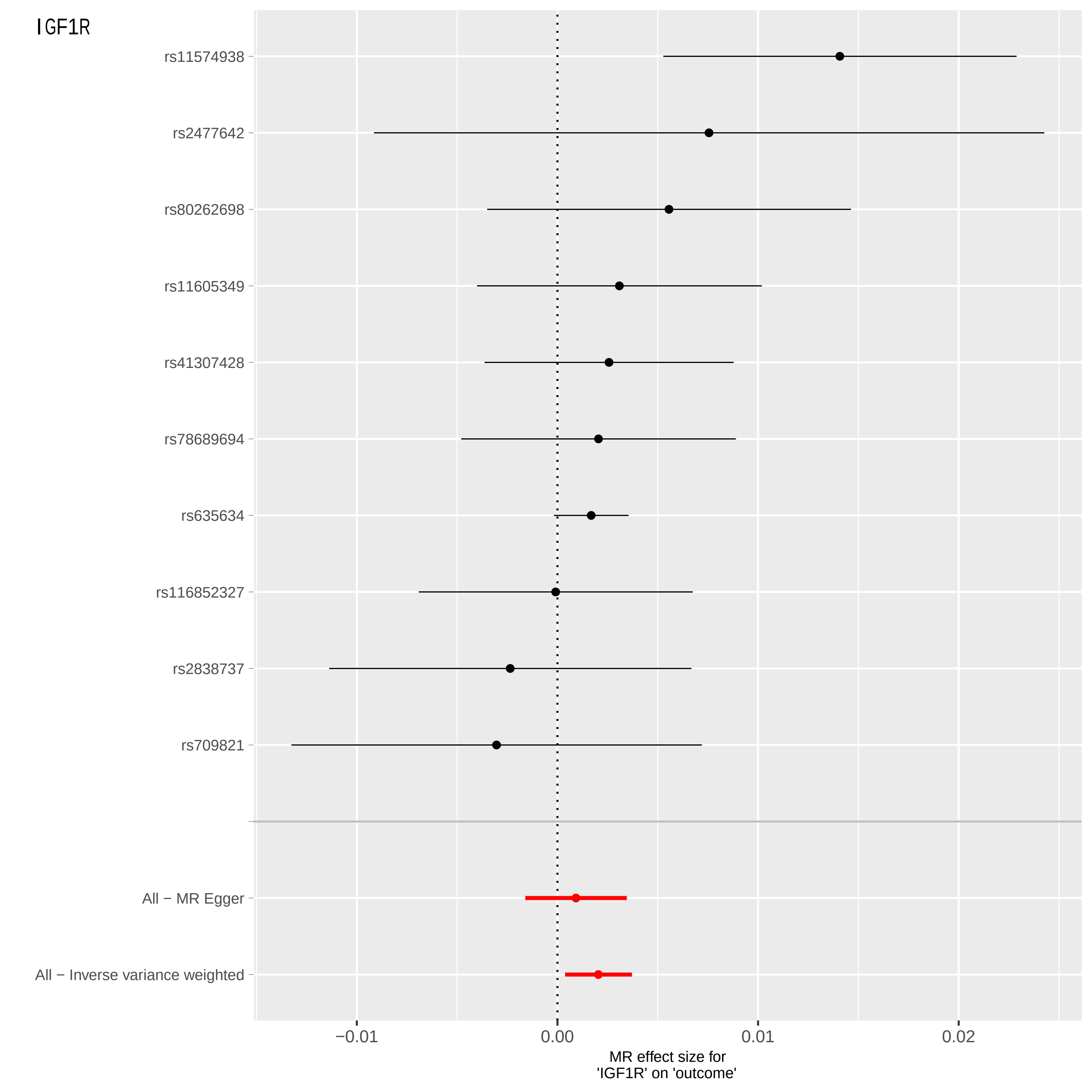

IGF1R
rs11574938
rs2477642
rs80262698
rs11605349
rs41307428
rs78689694
rs635634
rs116852327
rs2838737
rs709821
All − MR Egger
All − Inverse variance weighted
−0.01
0.00
0.01
0.02
MR effect size for
'IGF1R' on 'outcome'

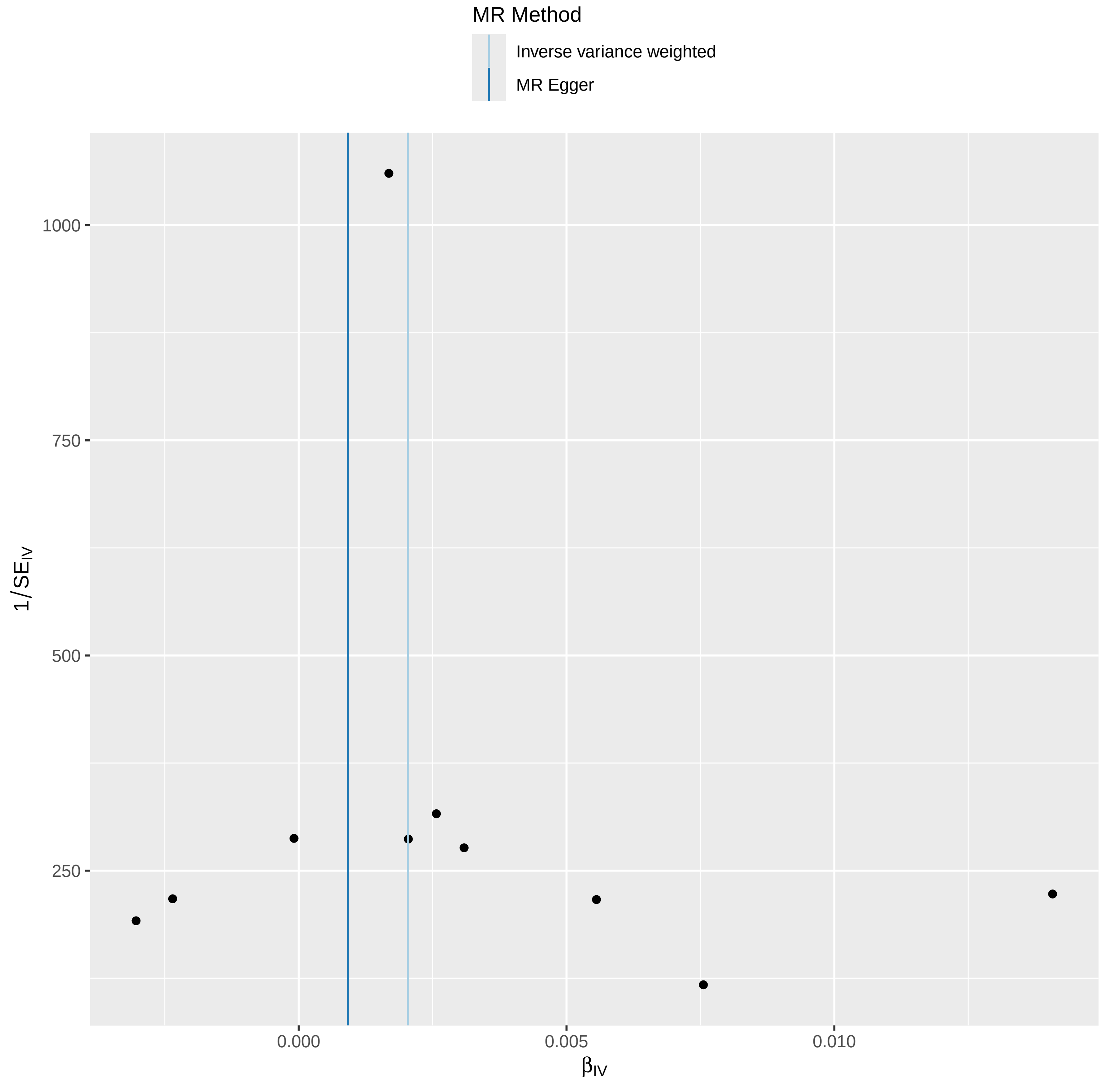
MR Method
Inverse variance weighted
MR Egger
1000
750
500
250
0.000
0.005
0.010
1/SE_IV
β_IV

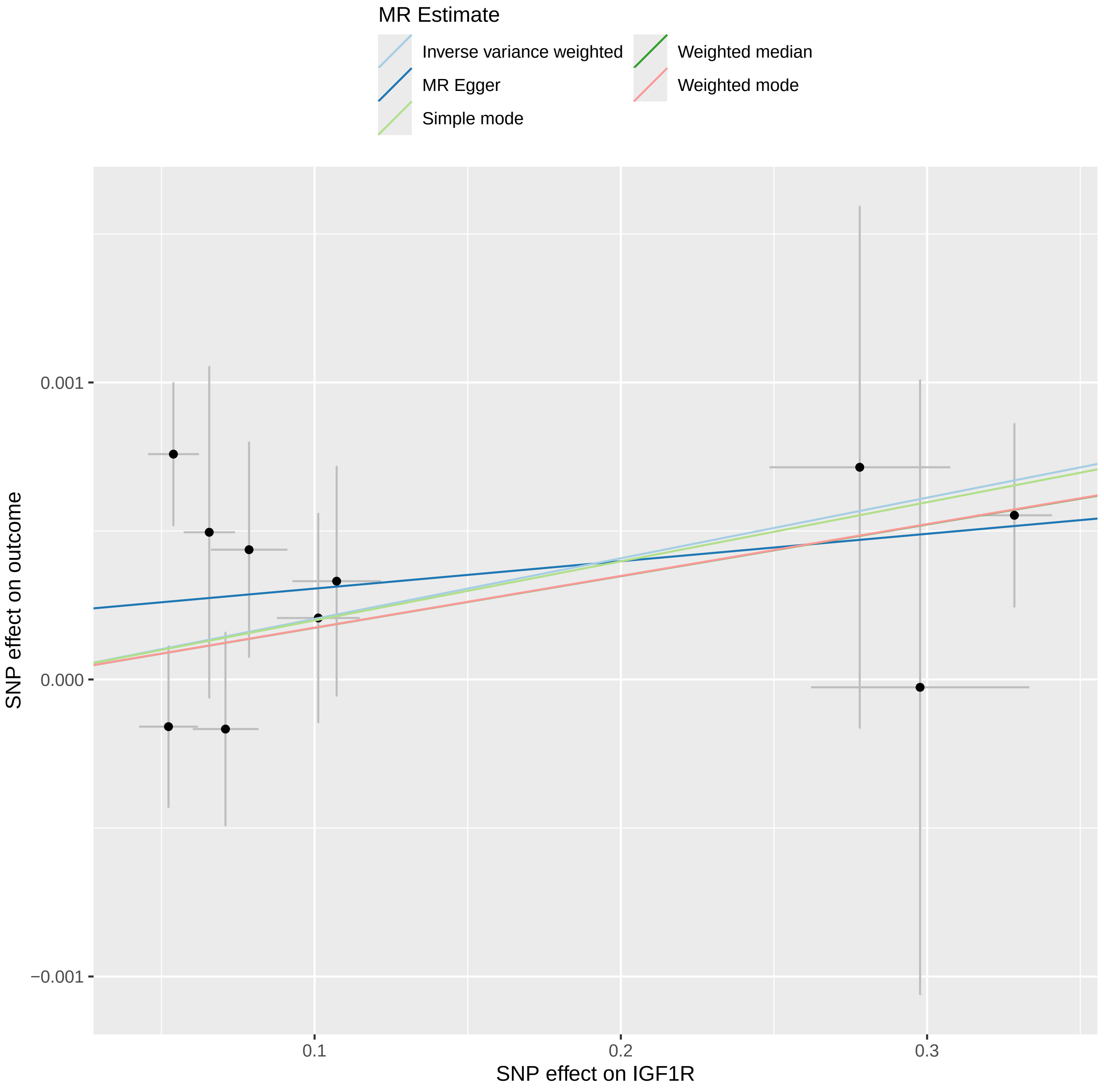

MR Estimate
Inverse variance weighted
MR Egger
Simple mode
Weighted median
Weighted mode
SNP effect on outcome
0.001
0.000
−0.001
0.1
0.2
0.3
SNP effect on IGF1R

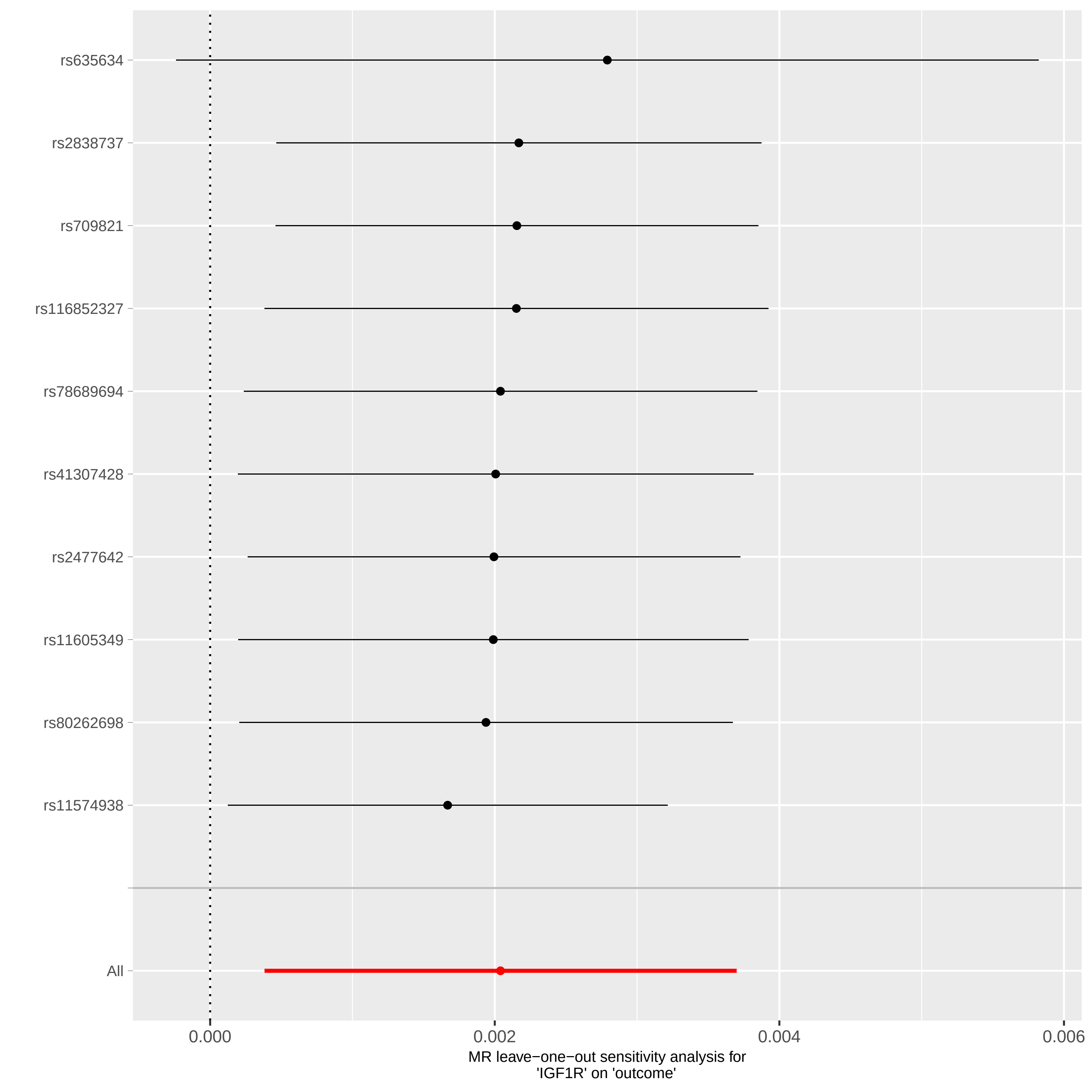

rs635634
rs2838737
rs709821
rs116852327
rs78689694
rs41307428
rs2477642
rs11605349
rs80262698
rs11574938
All
0.000
0.002
0.004
0.006
MR leave−one−out sensitivity analysis for
'IGF1R' on 'outcome'

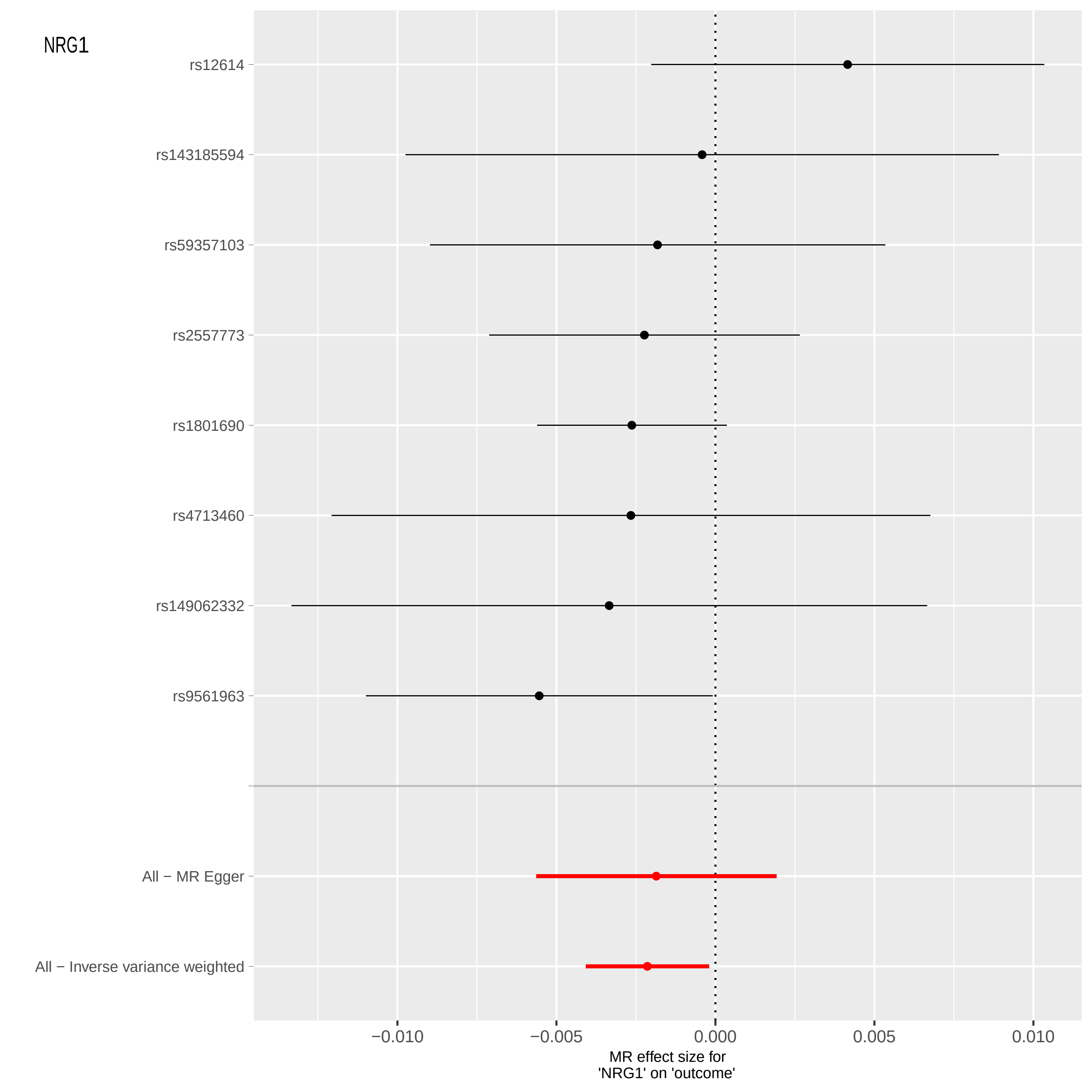

NRG1
rs12614
rs143185594
rs59357103
rs2557773
rs1801690
rs4713460
rs149062332
rs9561963
All − MR Egger
All − Inverse variance weighted
−0.010
−0.005
0.000
0.005
0.010
MR effect size for
'NRG1' on 'outcome'

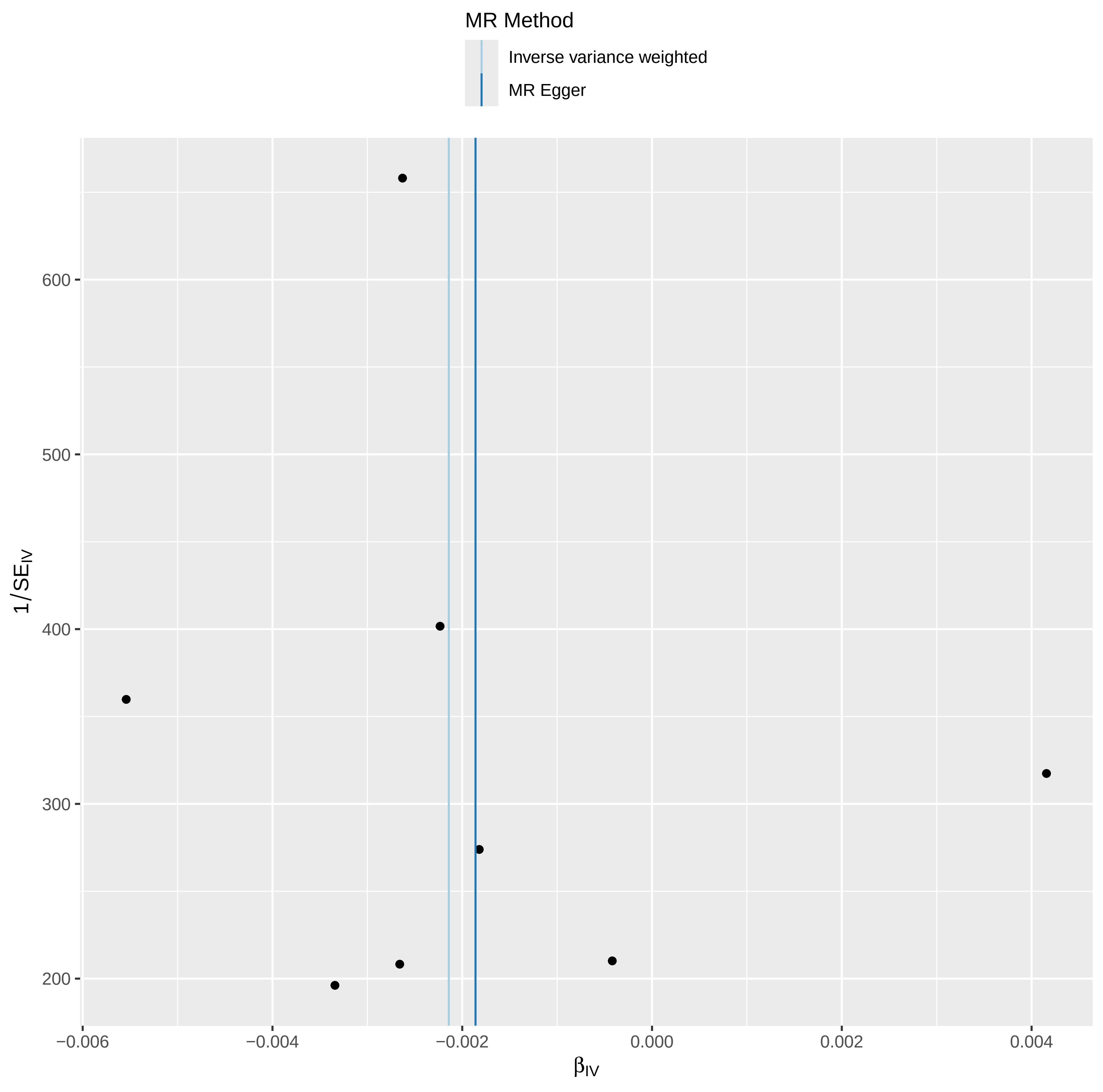

MR Method
Inverse variance weighted
MR Egger
600
500
400
300
200
1/SE$_{IV}$
−0.006
−0.004
−0.002
0.000
0.002
0.004
β$_{IV}$

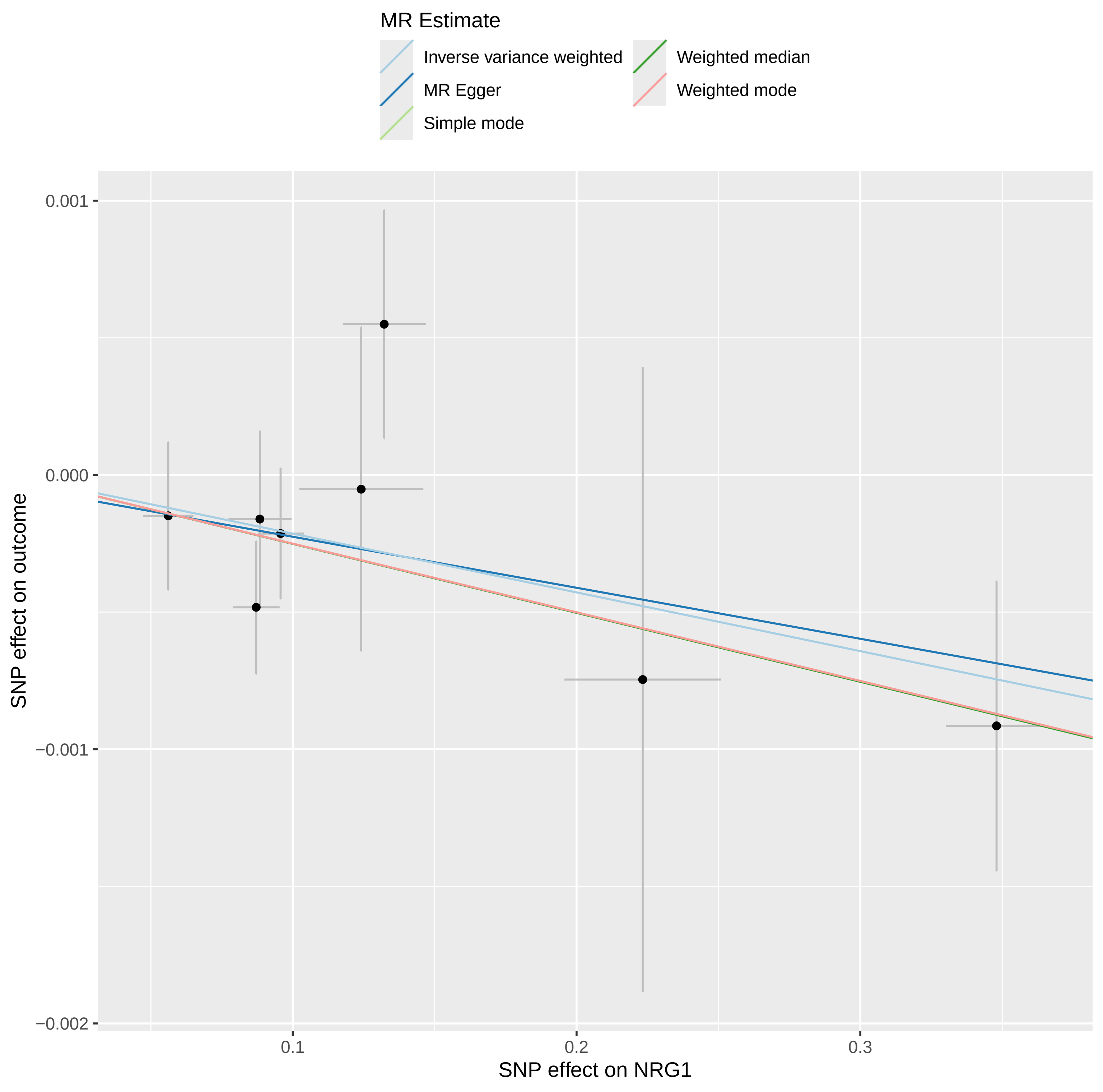

MR Estimate
Inverse variance weighted
MR Egger
Simple mode
Weighted median
Weighted mode
0.001
0.000
−0.001
−0.002
SNP effect on outcome
0.1
0.2
0.3
SNP effect on NRG1

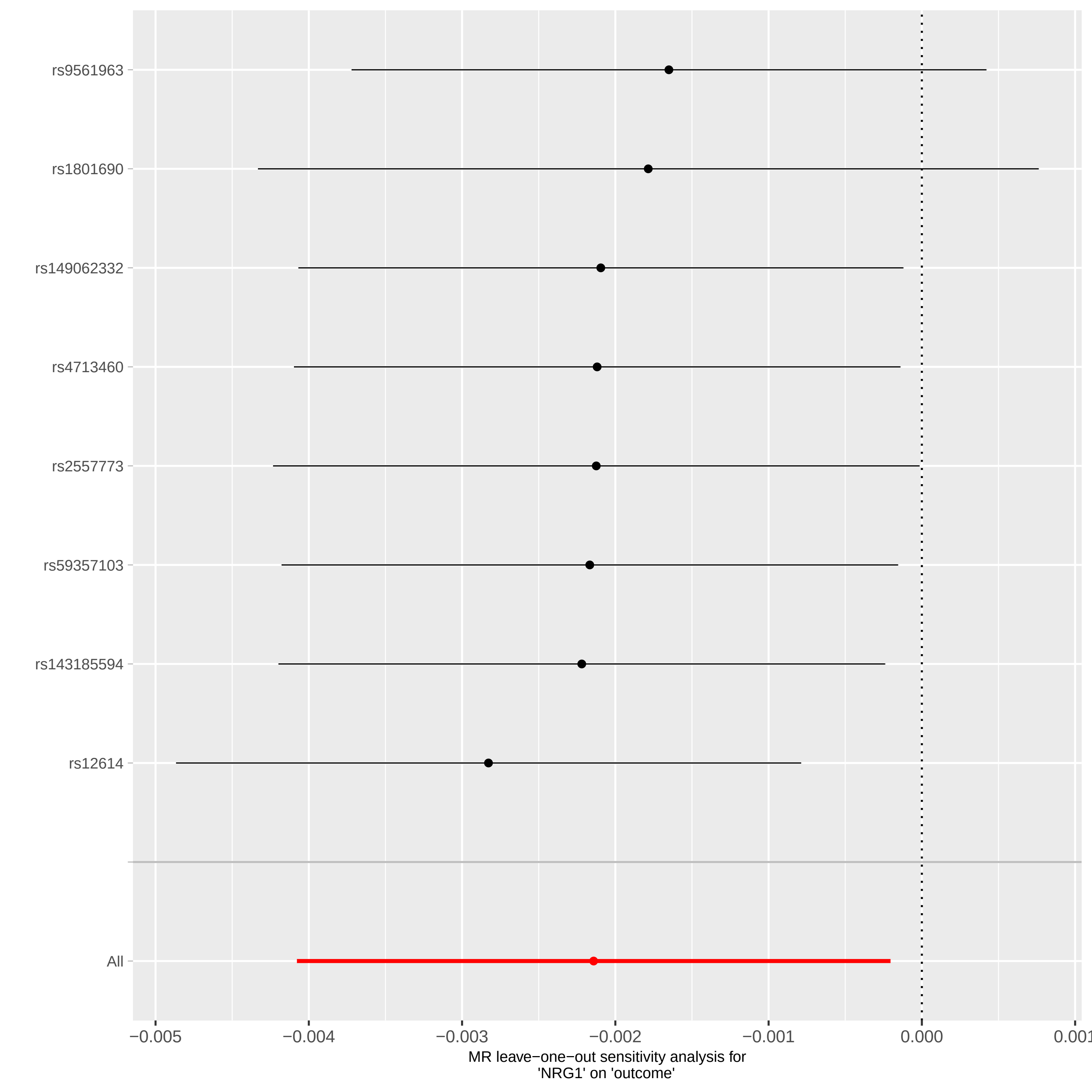
rs9561963
rs1801690
rs149062332
rs4713460
rs2557773
rs59357103
rs143185594
rs12614
All
−0.005
−0.004
−0.003
−0.002
−0.001
0.000
0.001
MR leave−one−out sensitivity analysis for
'NRG1' on 'outcome'

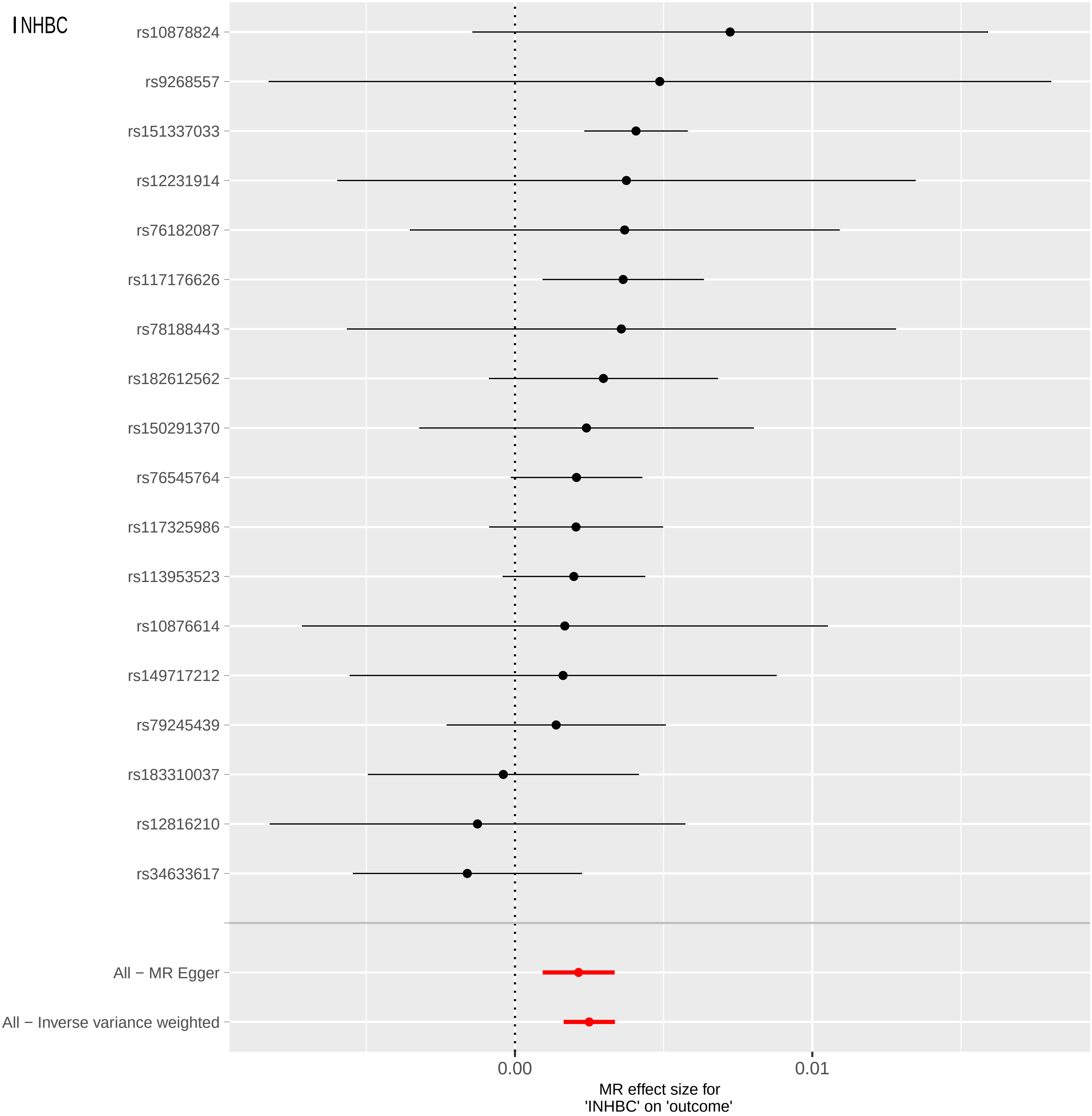
INHBC
rs10878824
rs9268557
rs151337033
rs12231914
rs76182087
rs117176626
rs78188443
rs182612562
rs150291370
rs76545764
rs117325986
rs113953523
rs10876614
rs149717212
rs79245439
rs183310037
rs12816210
rs34633617
All − MR Egger
All − Inverse variance weighted
0.00
0.01
MR effect size for
'INHBC' on 'outcome'

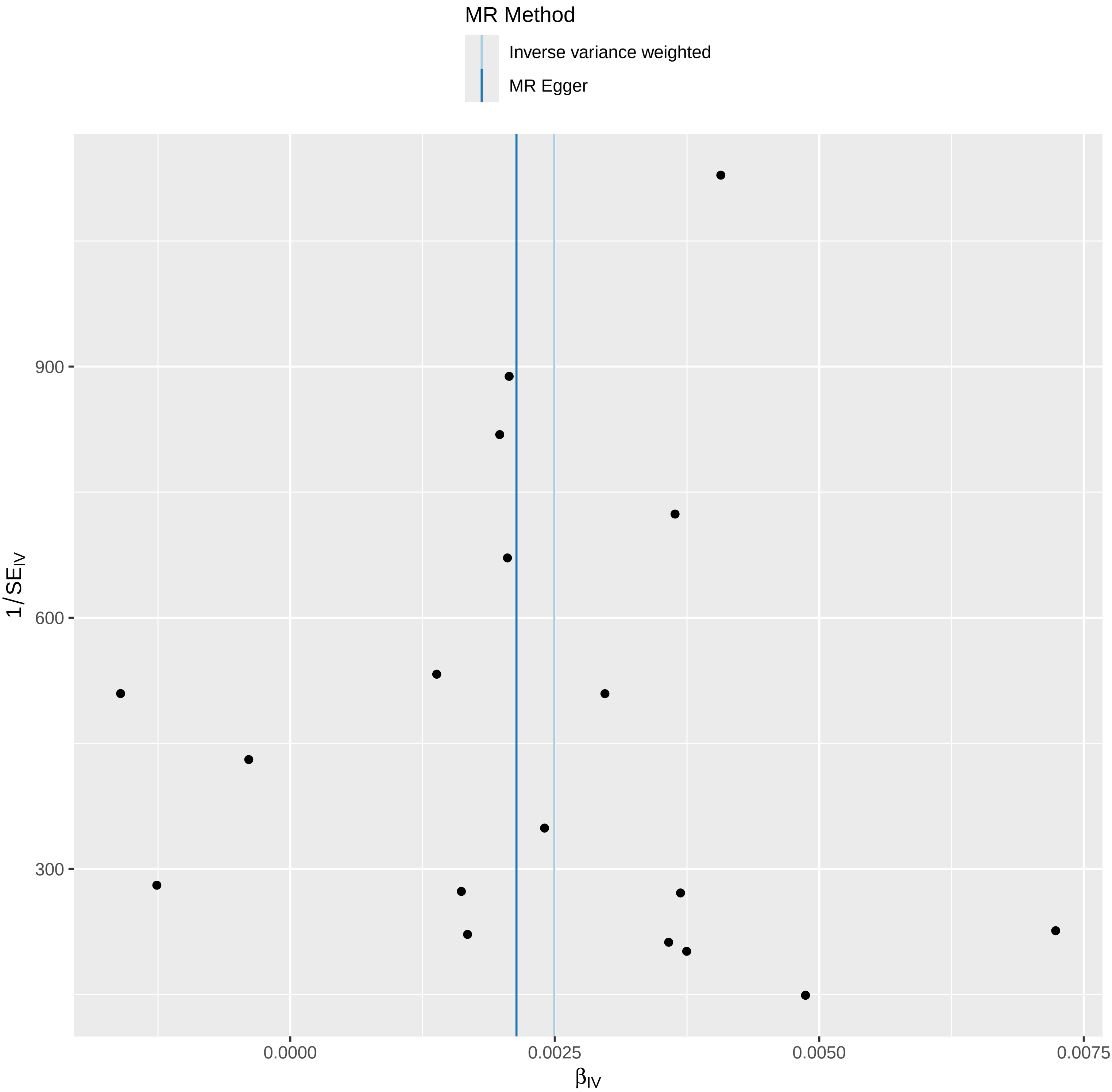

MR Method
Inverse variance weighted
MR Egger
1/SE_IV
900
600
300
0.0000
0.0025
0.0050
0.0075
β_IV

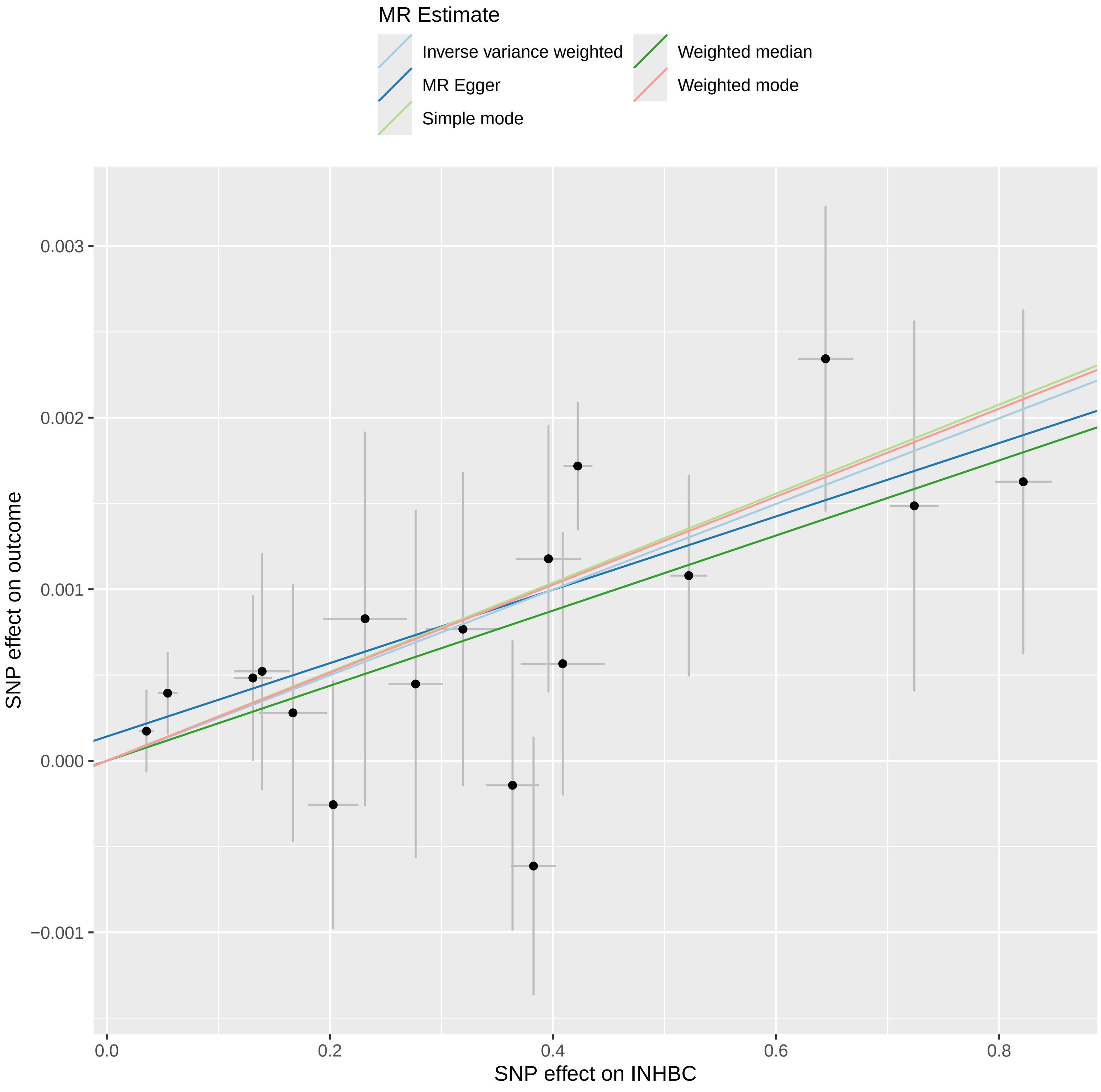
MR Estimate
Inverse variance weighted
MR Egger
Simple mode
Weighted median
Weighted mode
0.003
0.002
0.001
0.000
−0.001
SNP effect on outcome
0.0
0.2
0.4
0.6
0.8
SNP effect on INHBC

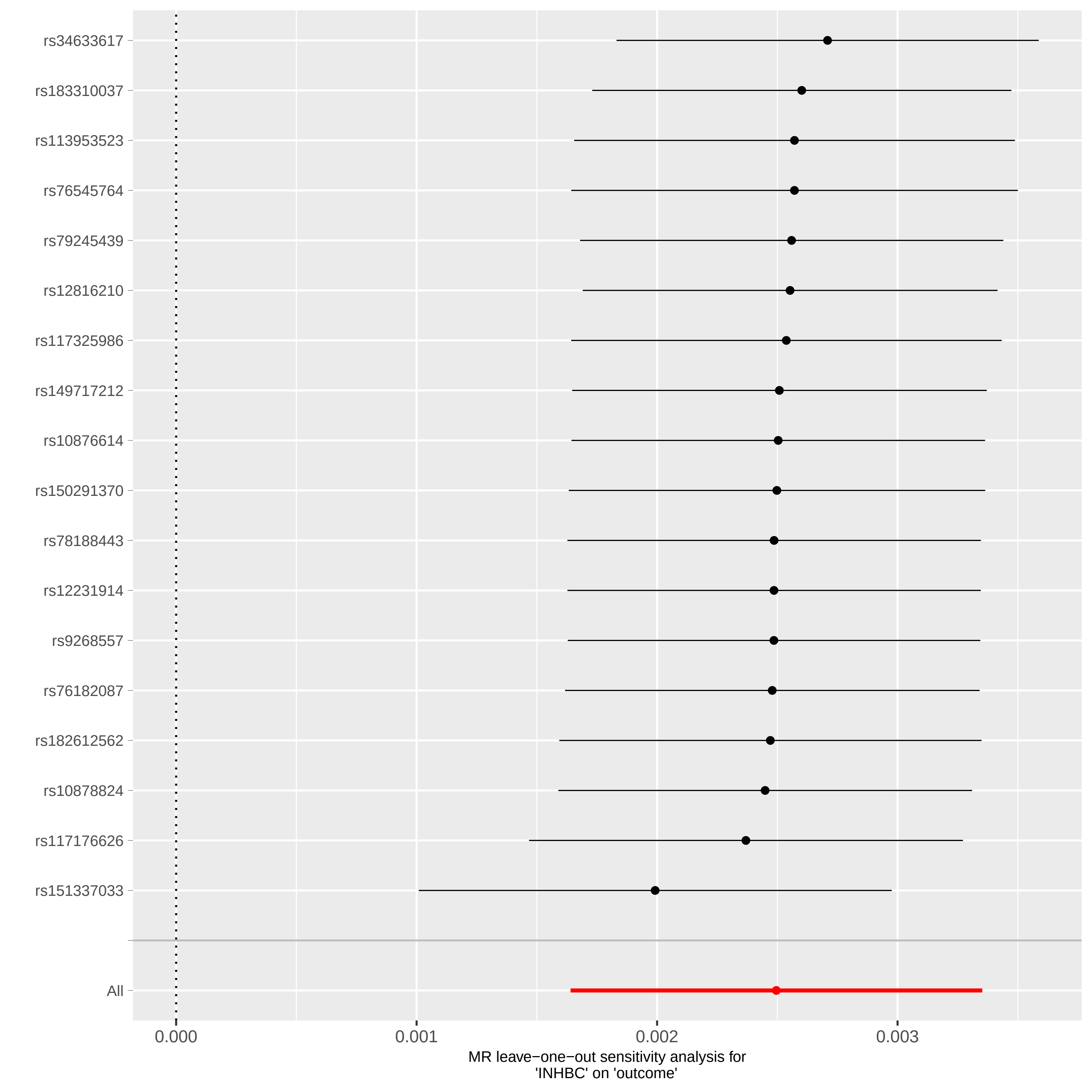

rs34633617
rs183310037
rs113953523
rs76545764
rs79245439
rs12816210
rs117325986
rs149717212
rs10876614
rs150291370
rs78188443
rs12231914
rs9268557
rs76182087
rs182612562
rs10878824
rs117176626
rs151337033
All
0.000
0.001
0.002
0.003
MR leave−one−out sensitivity analysis for
'INHBC' on 'outcome'

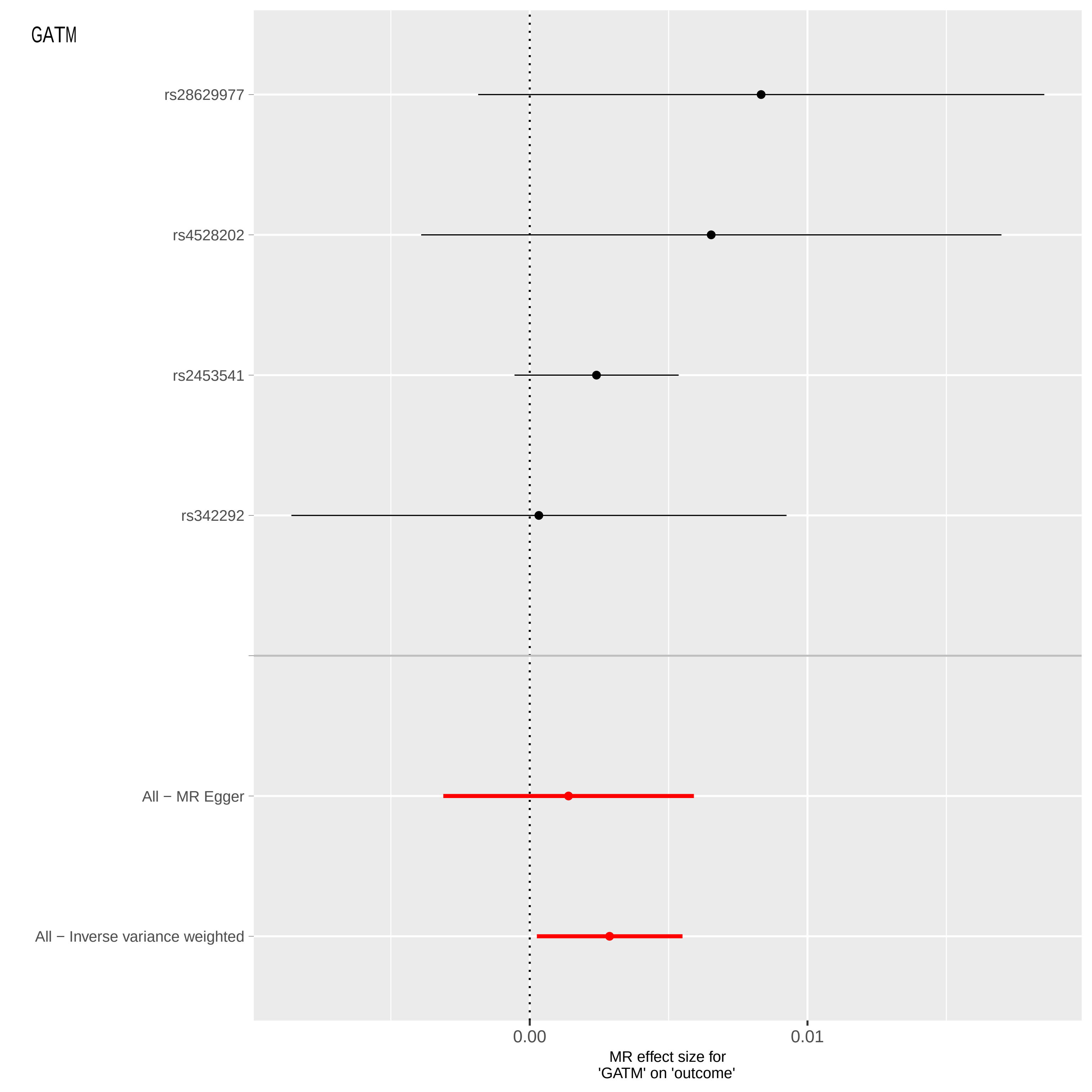

GATM
rs28629977
rs4528202
rs2453541
rs342292
All − MR Egger
All − Inverse variance weighted
0.00
0.01
MR effect size for
'GATM' on 'outcome'

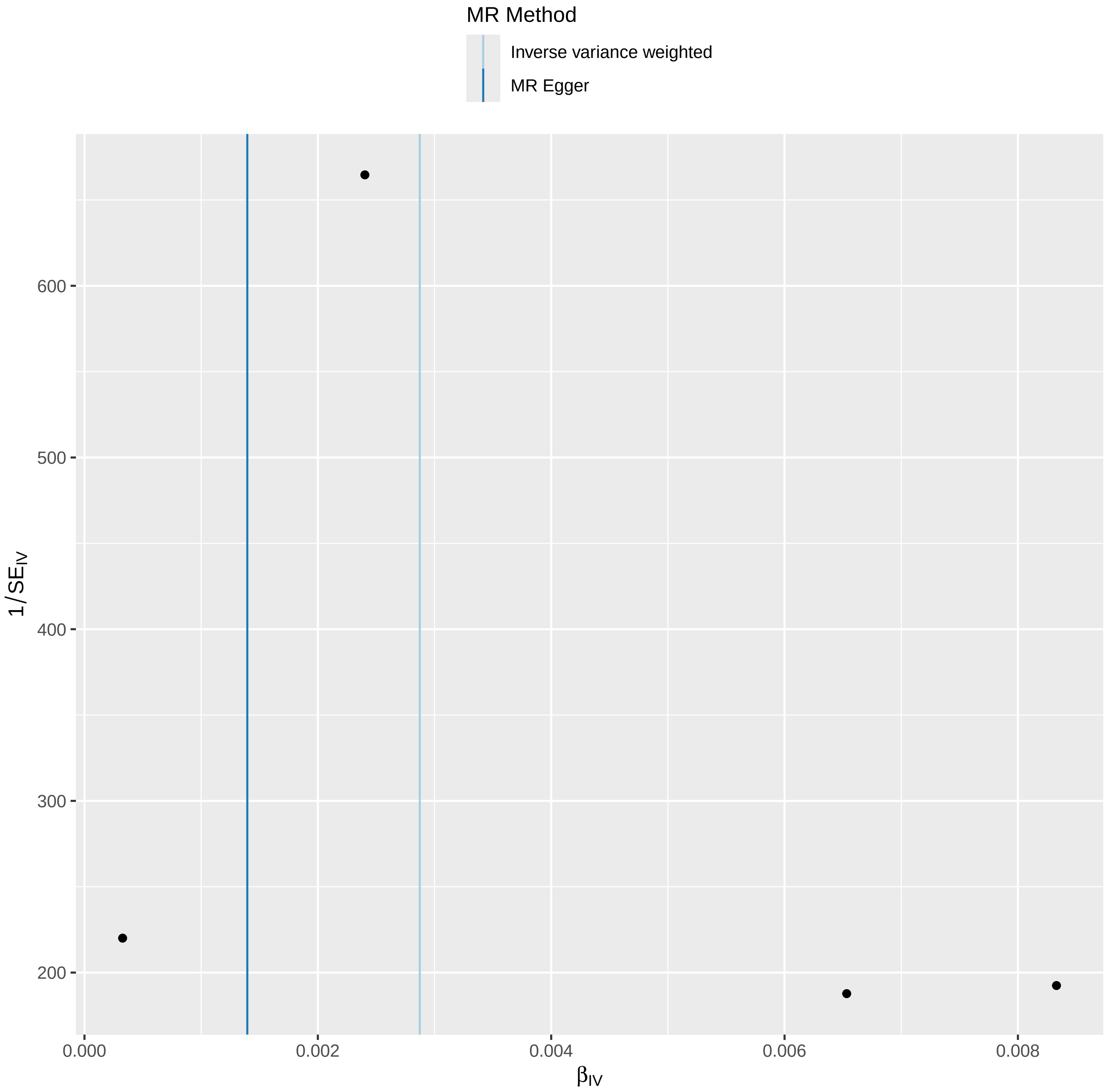
MR Method
Inverse variance weighted
MR Egger
600
500
400
300
200
$1/SE_{IV}$
0.000
0.002
0.004
0.006
0.008
β_{IV}

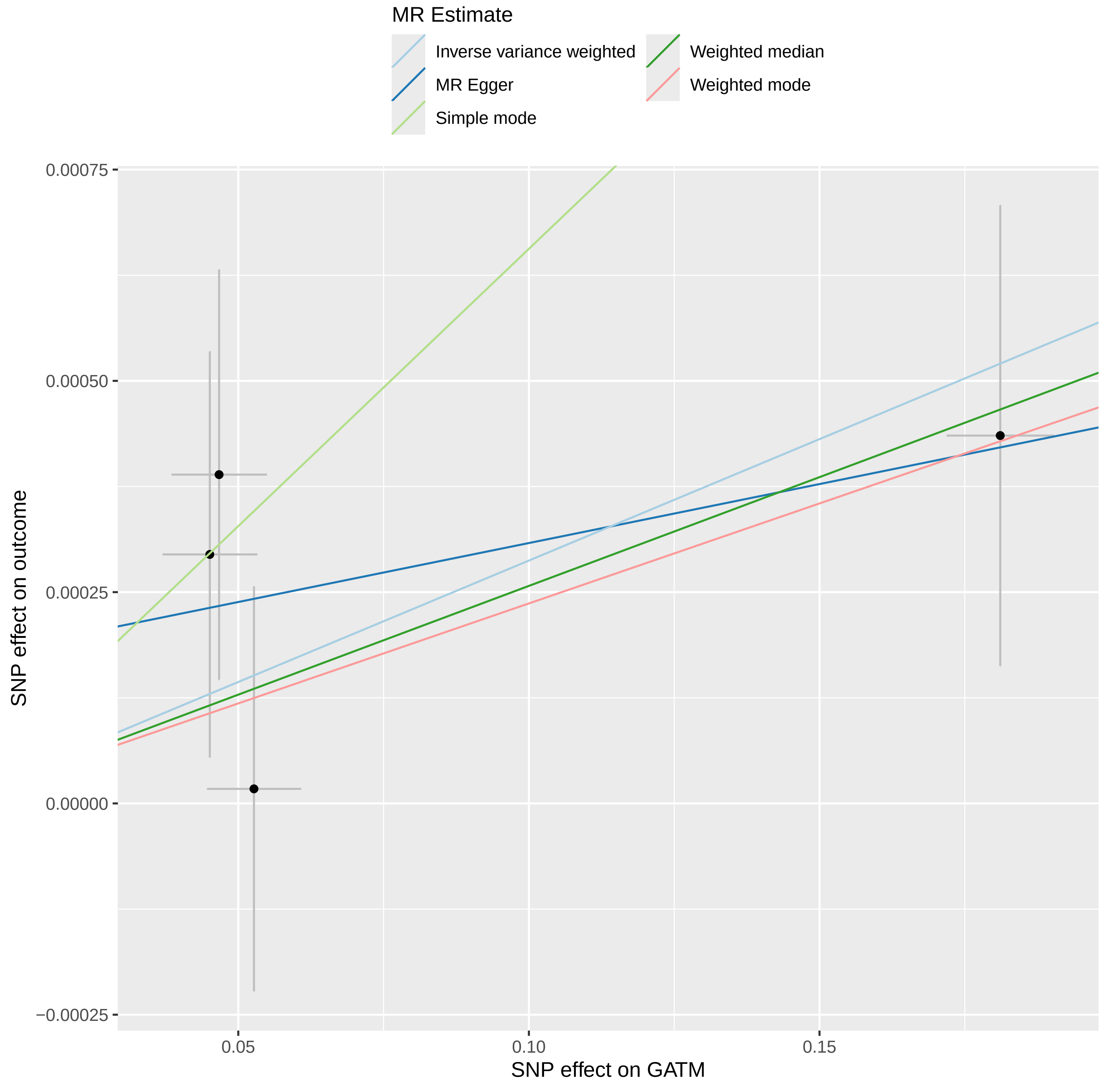

MR Estimate
Inverse variance weighted
MR Egger
Simple mode
Weighted median
Weighted mode
0.00075
0.00050
0.00025
0.00000
−0.00025
SNP effect on outcome
0.05
0.10
0.15
SNP effect on GATM

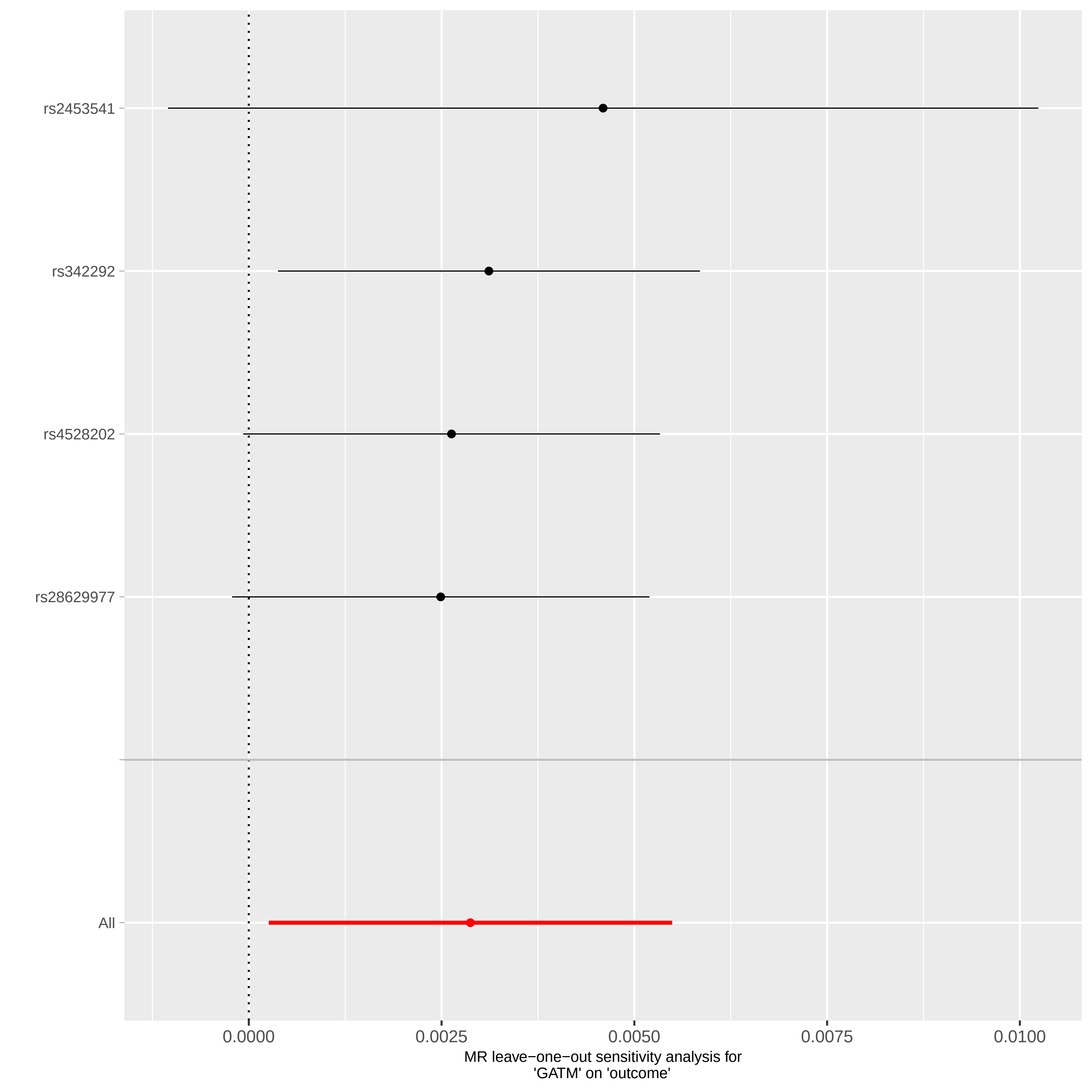
rs2453541
rs342292
rs4528202
rs28629977
All
0.0000
0.0025
0.0050
0.0075
0.0100
MR leave−one−out sensitivity analysis for
'GATM' on 'outcome'

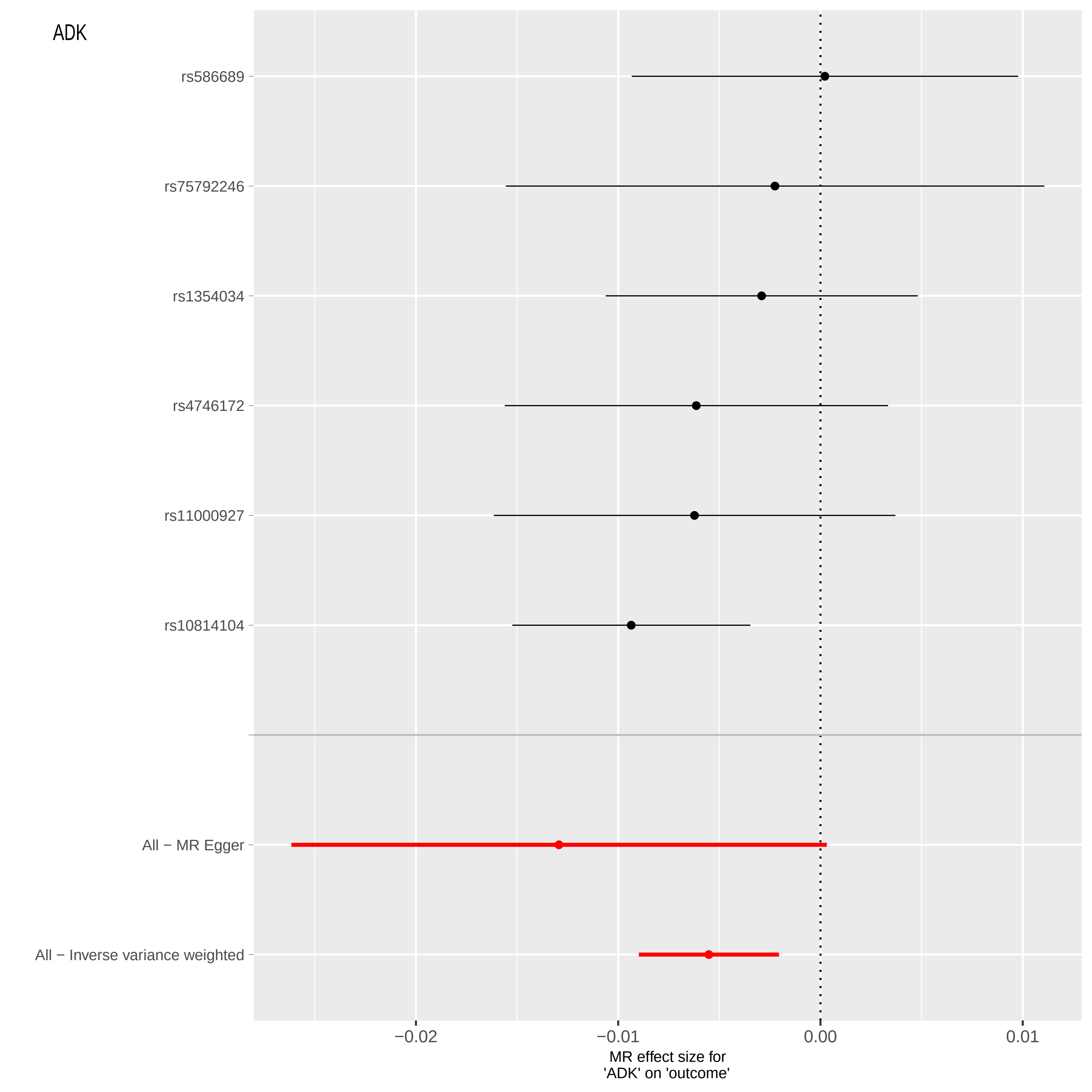

ADK
rs586689
rs75792246
rs1354034
rs4746172
rs11000927
rs10814104
All − MR Egger
All − Inverse variance weighted
−0.02
−0.01
0.00
0.01
MR effect size for
'ADK' on 'outcome'

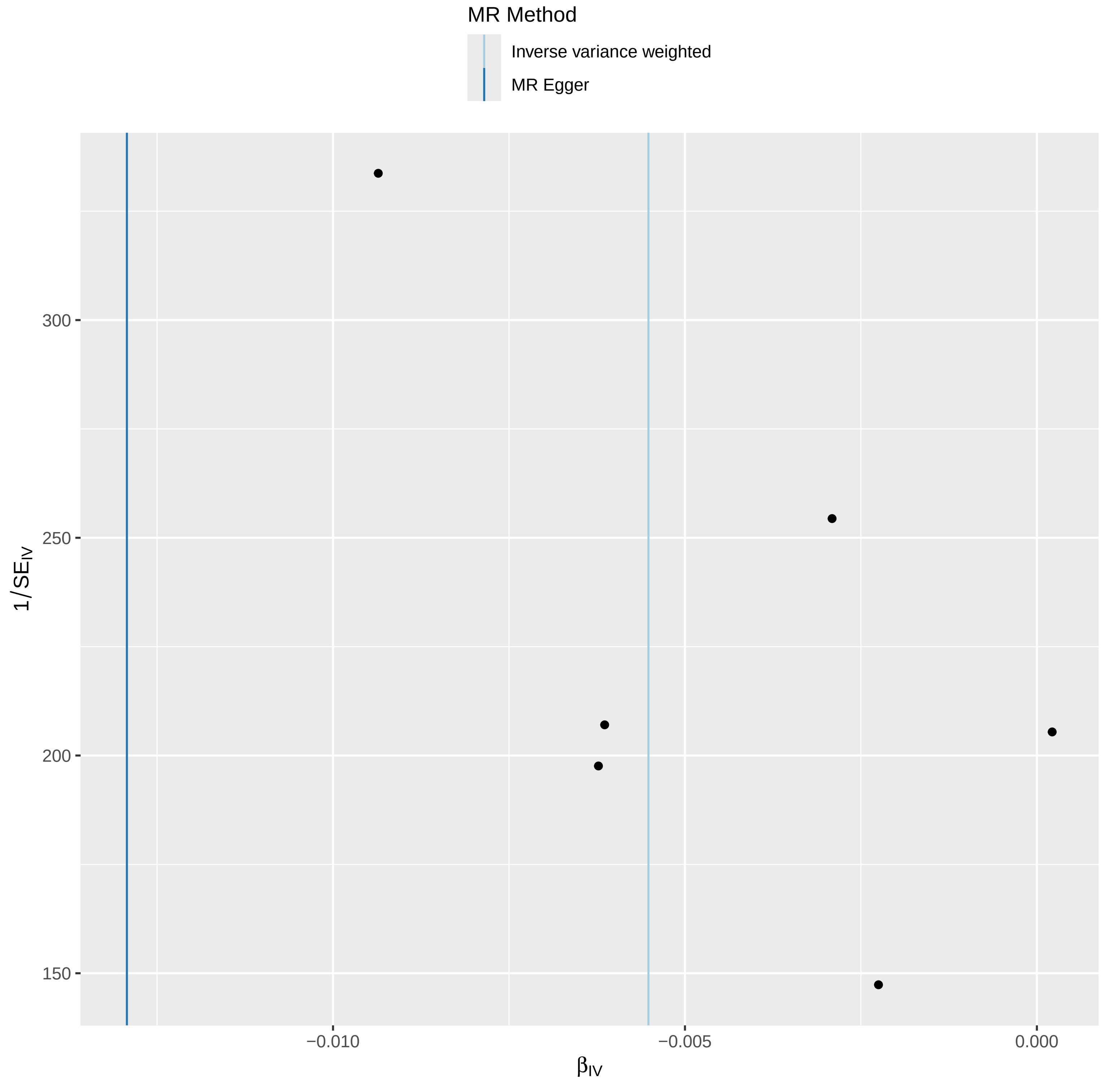

MR Method
Inverse variance weighted
MR Egger
300
250
200
150
1/SE_IV
−0.010
−0.005
0.000
β_IV

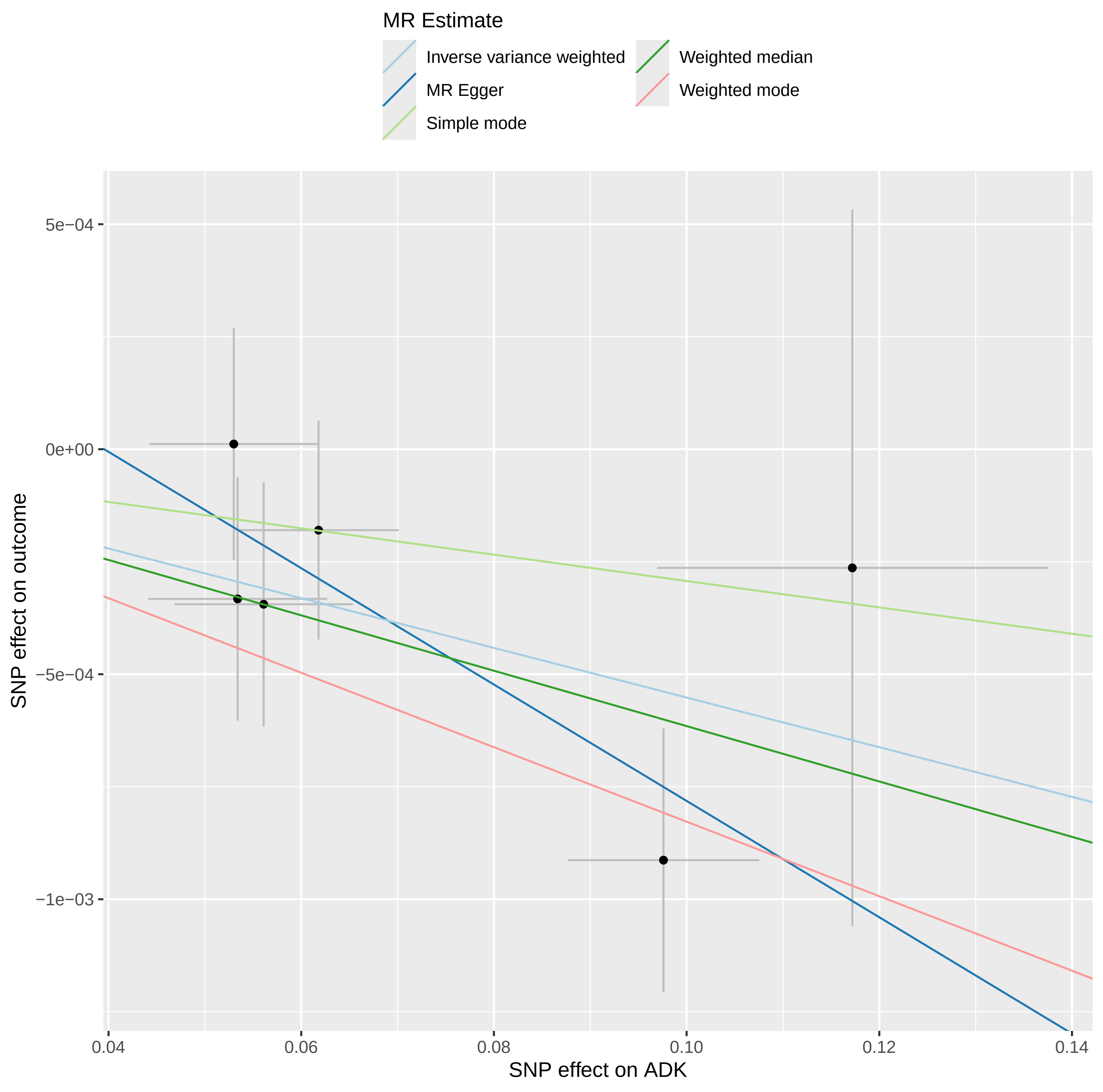

MR Estimate
Inverse variance weighted
MR Egger
Simple mode
Weighted median
Weighted mode
5e−04
0e+00
−5e−04
−1e−03
SNP effect on outcome
0.04
0.06
0.08
0.10
0.12
0.14
SNP effect on ADK

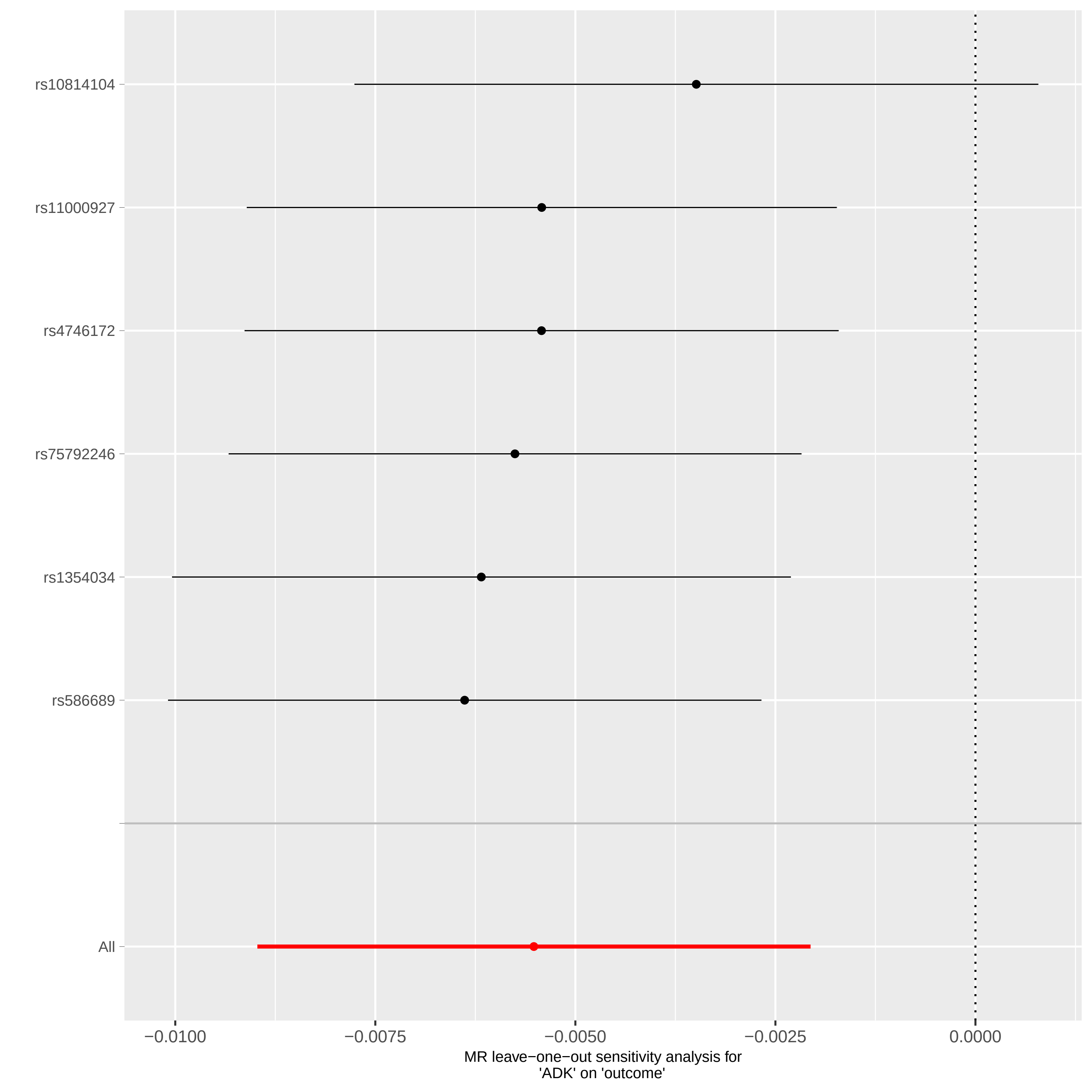

rs10814104
rs11000927
rs4746172
rs75792246
rs1354034
rs586689
All
−0.0100
−0.0075
−0.0050
−0.0025
0.0000
MR leave−one−out sensitivity analysis for
'ADK' on 'outcome'

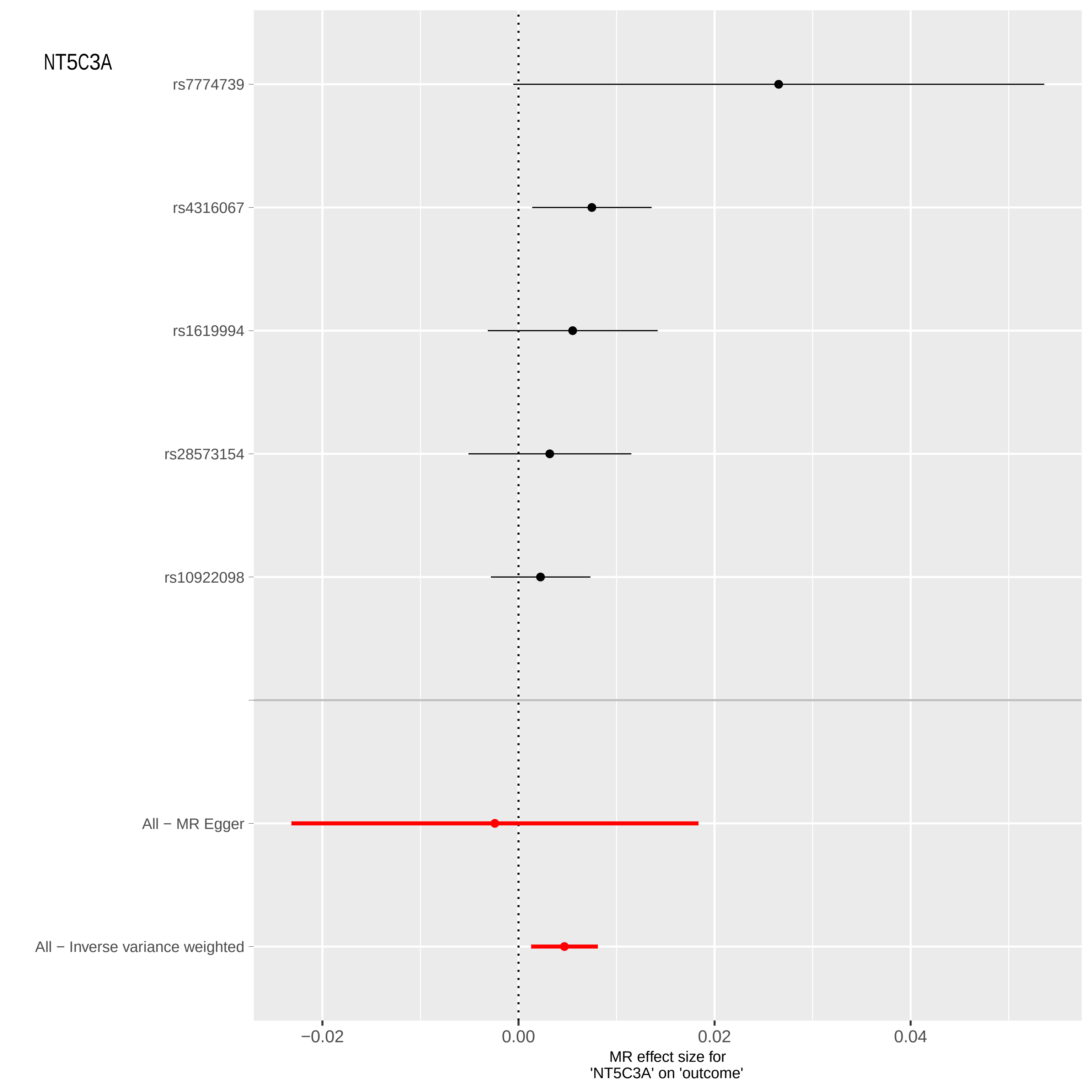

NT5C3A
rs7774739
rs4316067
rs1619994
rs28573154
rs10922098
All − MR Egger
All − Inverse variance weighted
−0.02
0.00
0.02
0.04
MR effect size for
'NT5C3A' on 'outcome'

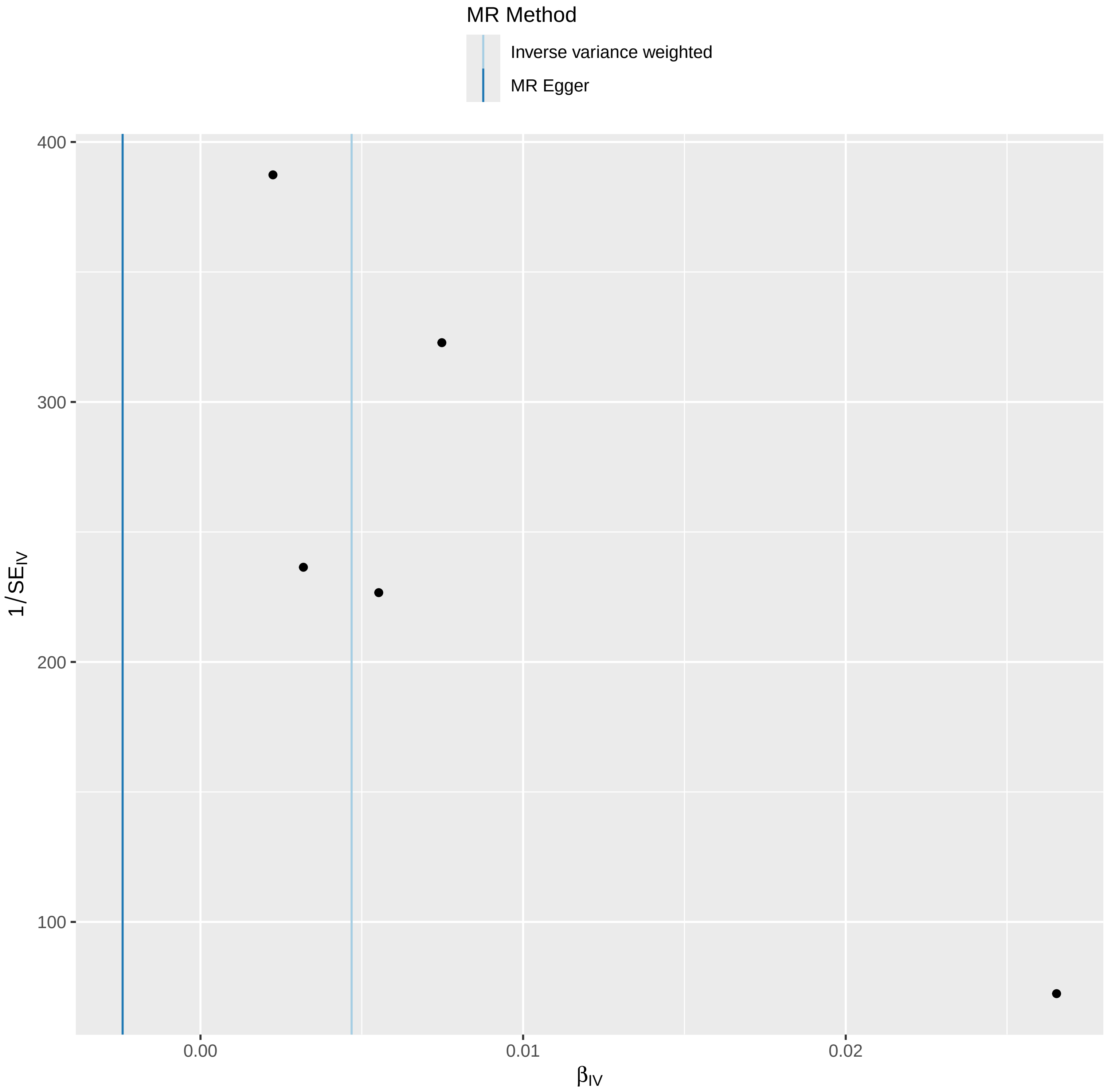
MR Method
Inverse variance weighted
MR Egger
400
300
200
100
$1/SE_{IV}$
0.00
0.01
0.02
β_{IV}

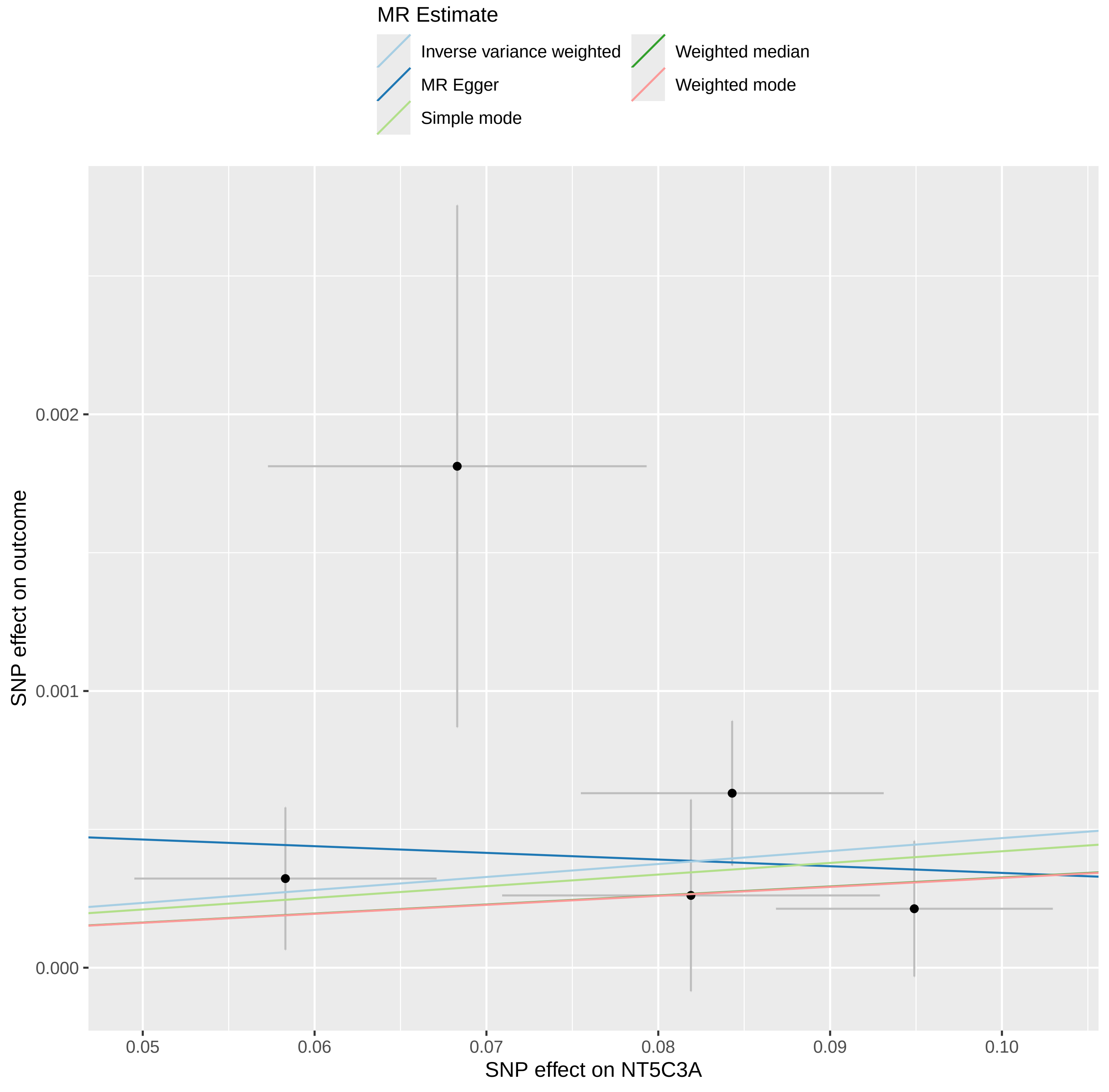

MR Estimate
Inverse variance weighted
MR Egger
Simple mode
Weighted median
Weighted mode
0.002
0.001
0.000
SNP effect on outcome
0.05
0.06
0.07
0.08
0.09
0.10
SNP effect on NT5C3A

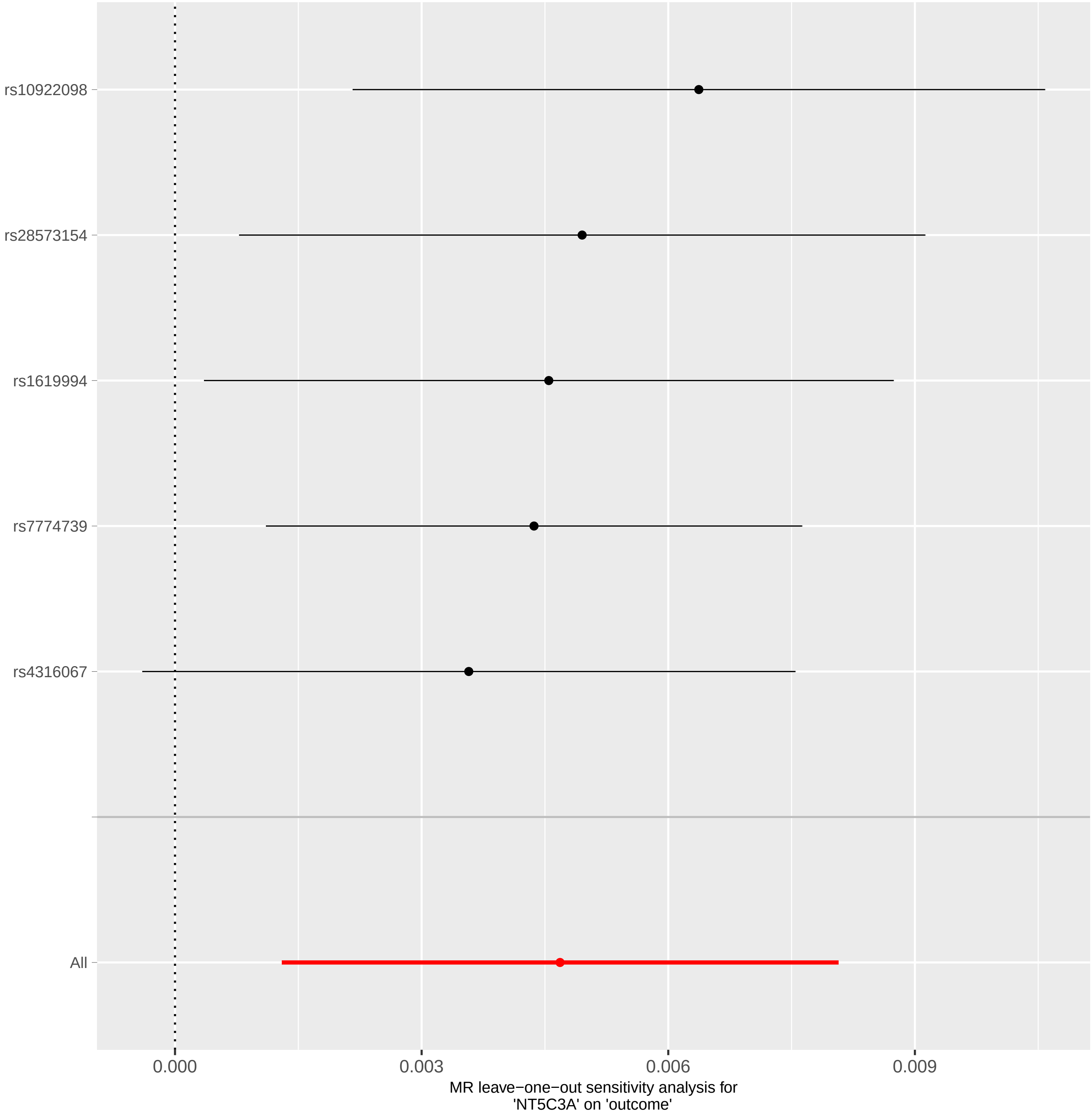
rs10922098
rs28573154
rs1619994
rs7774739
rs4316067
All
0.000
0.003
0.006
0.009
MR leave−one−out sensitivity analysis for
'NT5C3A' on 'outcome'
